# Integrated transcriptomics and metabolomics analysis reveals the biomolecular mechanisms associated to the antitumoral potential of a novel silver-based core@shell nanosystem

**DOI:** 10.1007/s00604-023-05712-3

**Published:** 2023-03-13

**Authors:** Guillermo Aragoneses-Cazorla, María Vallet-Regí, Ma. Milagros Gómez-Gómez, Blanca González, Jose L. Luque-Garcia

**Affiliations:** 1 Department of Analytical Chemistry, Faculty of Chemical Sciences, Complutense University of Madrid, 28040 Madrid, Spain; 2 Department of Chemistry in Pharmaceutical Sciences, Faculty of Pharmacy, Complutense University of Madrid, Instituto de Investigación Sanitaria Hospital, 12 de Octubre (I+12), 28040 Madrid, Spain; 3 Centro de Investigación Biomédica en Red de Bioingeniería, Biomateriales Y Nanomedicina (CIBER-BBN), Saragossa, Spain

**Keywords:** Metabolomics, Transcriptomics, Mesoporous silica nanoparticles, Cancer treatment, Electron transport chain complex, ATP synthesis

## Abstract

A combination of omics techniques (transcriptomics and metabolomics) has been used to elucidate the mechanisms responsible for the antitumor action of a nanosystem based on a Ag core coated with mesoporous silica on which transferrin has been anchored as a targeting ligand against tumor cells (Ag@MSNs-Tf). Transcriptomics analysis has been carried out by gene microarrays and RT-qPCR, while high-resolution mass spectrometry has been used for metabolomics. This multi-omics strategy has enabled the discovery of the effect of this nanosystem on different key molecular pathways including the glycolysis, the pentose phosphate pathway, the oxidative phosphorylation and the synthesis of fatty acids, among others.

**Graphical Abstract:**

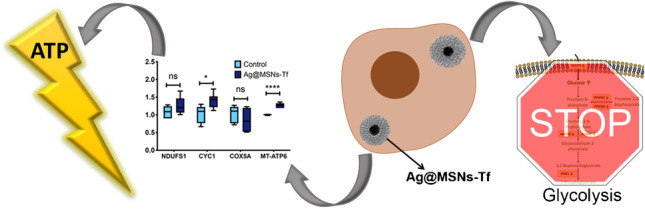

**Supplementary Information:**

The online version contains supplementary material available at 10.1007/s00604-023-05712-3.

## Introduction

The main drawbacks of traditional cancer treatments are the associated side effects and the presence of multidrug-resistant cancer cells, making it necessary to develop new approaches for cancer treatment [1–3]. In this sense, the use of nanosystems as drug delivery systems is a promising way for cancer treatment [4]. Mesoporous silica nanoparticle–based (MSNs) nanosystems have been widely employed with this aim thanks to their physico-chemical properties, such as high surface area, possibility of surface functionalization, or tunable size, among others, which confer these nanosystems a great versatility [5–9]. Apart from improving the pharmaceutical properties of therapeutic compounds, these nanoplatforms allow the co-delivery of different therapeutic agents, constituting an alternative to overcome cancer cell drug resistance [10–12] or even providing dual antimicrobial and osteogenic effects in the case of bone infection [13]. Taking this into account, the evaluation of the biomolecular mechanisms by which the nanosystems exert their antitumoral effects is crucial to identify possible cellular defense mechanisms activated after nanosystem exposure, thus allowing the improvement of the functionality of these nanoplatforms to increase their therapeutic action. In this context, the application of -omic techniques, such as proteomics, metabolomics, or transcriptomics, helps to evaluate impaired molecular pathways after exposure to cytotoxic agents [14, 15]. While metabolomics allows the identification of low molecular weight compounds involved in essential cellular functions in a wide variety of samples including cells, tissues, or body fluids [16, 17], transcriptomics profiling provides information about the expression levels of different genes comprising the transcriptome of a biological sample at a certain moment, since gene expression is very sensitive to external perturbations, such as the exposure to antitumoral agents [18]. The combination of these two powerful discovery tools (metabolomics and transcriptomics) allows for the identification of complete biochemical pathways involved in the biological process under study [19].

In our previous study, a hemocompatible transferrin-decorated core–shell nanosystem based on a silver nanoparticle core covered by a mesoporous silica coating (Ag@MSNs-Tf) was developed and extensively characterized. This nanosystem was proved to be selectively internalized by HepG2 cells, which overexpress their transferrin receptors; then, it was able to significantly reduce cellular viability and induce cellular death. In that work, it was demonstrated that Ag@MSNs-Tf was able to induce cellular apoptosis via the activation of the caspase cascade and through the interaction of different cell cycle components with membrane apoptotic receptors [20]. However, much of the biochemical mechanisms by which this nanosystem exerts its antitumor potential remain to be elucidated. In this work, in order to further reveal the molecular mechanisms involved, both targeted and untargeted metabolomics have been combined with transcriptomic profiling. The results obtained have allowed us to obtain a much more complete picture of how the Ag@MSNs-Tf nanosystem exerts its antitumor action, therefore providing useful information for the design of new, more complex, and more effective nanosystems.

## Materials and methods

### Ag@MSNs-Tf synthesis and characterization

Ag@MSNs-Tf nanosystem was synthesized as previously described [20]. Briefly, AgNPs were synthesized in a CTAB-containing medium prior to mesoporous silica shell formation. Then, Ag@MSNs were functionalized to display carboxylic acid groups (-COOH) onto their surface (Ag@MSNs-COOH). Finally, transferrin (Tf) was covalently grafted on the nanosystem via carbodiimide chemistry (Ag@MSNs-Tf). The nanosystem preparation was monitored after each synthetic step by thermogravimetric and differential thermal analysis (TGA and DTA) in a PerkinElmer Pyris Diamond TG/DTA analyzer, Fourier transform infrared (FTIR) spectroscopy using a Thermo Nicolet Nexus spectrometer, electrophoretic mobility measurements to calculate the values of the zeta-potential (*ζ*), and dynamic light scattering (DLS) performed in a Zetasizer Nano ZS Malvern instrument and transmission electron microscopy acquired in a JEOL JEM 1400 microscope.

### Cell culture

Human hepatocellular carcinoma cell line (HepG2) was maintained in Dubelcco’s modified Eagle’s medium (DMEM) supplemented with 10% fetal bovine serum (FBS) and 1% penicillin/streptomycin at 37 °C and 5% CO_2_.

### Cytotoxicity assays

For the cell viability assay, HepG2 cells were seeded on 96-well plates 24 h prior to the experiment. After cell attachment, cells were exposed to 10, 15, and 25 µg/mL of Ag@MSNs-Tf for 72 h. Then, 20 µL of MTT reagent (3-(4,5-dimethyl-thiazol-2-yl)2,5-diphenyl tetrazolium bromide) was added to each well and incubated for 4 h at 37 °C. The media was then removed, and the generated formazan crystals were dissolved in 100 mL of dimethyl sulfoxide. Absorbance was measured at 595 nm using a microplate reader (Sunrise, Tecan).

### Targeted metabolomics

HepG2 cells were seeded into P100 plates and exposed to 10 µg/mL of the Ag@MSNs-Tf nanosystem for 72 h. After the exposure time, cells were placed on ice and washed with 10 mL of 0.9% NaCl. Before harvesting, 100 µL of methanol at − 20 °C was added. Then, 400 µL of an ice-cold 0.4% formic acid (v/v) solution was used to resuspend the cells, which were transferred to a 1.5-mL Eppendorf tube and vortexed for 1 min. After 3 min of incubation on ice, 45 µL of 15% NH_4_HCO_3_ (w/v) was added to neutralize the pH of the samples. Samples were then vortexed for 1 min and incubated for 20 min on ice. Prior to the LC–MS analysis, samples were centrifuged at 16,000 g and 4 °C for 10 min, and the supernatant was filtered through a PTFE membrane (pore size 0.22 µm). Total protein concentration of each sample was calculated by means of the Bradford assay, thus allowing further normalization of the concentration of each metabolite. The LC–MS analysis was carried out in multiple reaction monitoring (MRM) mode as previously described [21].

Differences in ATP, ADP, NADH, and NAD^+^ contents between control and cells exposed to Ag@MSNs-Tf were assessed by ANOVA statistical analysis at a 95% confidence level (*p* value < 0.05), followed by the Bonferroni test.

### Untargeted metabolomics

Seven replicates (10^7^ cells approx.) of each studied condition, HepG2 cells exposed to 10 µg/mL of Ag@MSNs-Tf nanosystem for 72 h and control untreated cells, were prepared. For the extraction of intracellular metabolites, cells were washed with 10 mL of 0.9% NaCl and placed on ice. The metabolic cycle was quenched by adding 400 µL of methanol at − 20 °C and 400 µL of ice-cold water. Then, cells were scraped and transferred into 1.5-mL tubes. Four hundred microliters of CHCl_3_ at − 20 °C was added to each sample; the tubes were incubated in a shaker for 20 min at 1400 rpm and 4 °C, and then centrifuged at 16,000 g and 4 °C for 5 min. After the separation of the polar and non-polar phases, 300 µL from each phase was transferred to different glass vials. The protein interphase was removed and the total protein concentration of each sample was estimated using the Bradford assay. 50 mg/L of 4-chloroplenylalanine was added to each sample as an internal standard. Then, the extracts were evaporated under a N_2_ stream. Prior to GC–MS analysis, the extracted metabolites were chemically derivatized by adding 30 µL of 50 mg/L methoxyamine hydrochloride in pyridine. The mixture was incubated at 500 rpm for 90 min at 37 °C. Subsequently, 60 µL of *N*,*O*-bis(trimethylsilyl)trifluoroacetamide (BTSFA) containing 1% trimethylsilyl chloride (TMCS) was added and incubated at 500 rpm for 1 h at 60 °C [22]. Samples were analyzed in a gas chromatograph (7890A, Agilent) coupled to a time-of-flight (TOF) high-resolution mass spectrometer (GCT premier Micromass, Waters) using the conditions previously described [21].

Chromatographic data were analyzed with Mass Lynx software and the peak area of each metabolite was normalized with the corresponding internal standard area. Metabolites were identified based on both the mass spectra and the accurate mass value using the NIST MS search 2.0 library.

### Transcriptome analysis

To evaluate potential alteration in the mRNA expression levels of HepG2 cells exposed to the proposed nanosystem, a microarray-based transcriptome profiling was carried out. HepG2 cells were seeded in P100 plates for 24 h, and then exposed to 10 µg/mL of Ag@MNSs-Tf for 72 h. The total mRNA was extracted from each sample using a commercial kit (PureLink® Invitrogen). Briefly, cells were harvested and centrifuged and the supernatant was removed. Cells were lysed with a buffer containing 2-mercaptoethanol. Lysates were then centrifuged and one volume of 70% ethanol was added to the samples. Seven hundred microliters of this volume was transferred to a spin cartridge and centrifuged. Afterwards, samples were rinsed with washing buffer and RNase-free water was added to the spin cartridge prior to centrifugation. The purified RNA was stored at − 80 °C for further analysis. Samples were processed with GeneChip® WT PLUS Reagent Kit (Applied Biosystems) hybridized with Clariom™ D array and Human (Applied Biosystems) and scanned with a GeneChip® Scanner 3000 7G (Applied Biosystems). Raw data were processed with RMA algorithm included in the Transcriptome Analysis Console (Applied Biosystems) for normalization and gene levels analysis. For each experimental condition (control and Ag@MSNs-Tf exposed cells), three microarray experiments corresponding to three independent RNA replicates were processed and analyzed. Fold changes between both experimental conditions were calculated as a quotient between the mean of the gene expression signals. Statistical analysis was performed with ebayes limma included in the Transcriptome Analysis Console.

### RT-qPCR analysis of the electron transport chain complexes

The mRNA levels of the genes involved in the electron transport complexes were measured in HepG2 cells after exposure to 10 μg/mL of Ag@MSNs-Tf for 72 h. Total RNA was isolated using the TRIzol® reagent (Invitrogen) according to the manufacturer’s instructions. The quantity of extracted RNA was measured using a NanoDrop One (Thermo Fisher Scientific). The synthesis of cDNA with integrated removal of genomic DNA contamination was performed using a Quantitec reverse transcription kit (Qiagen) employing 1 μg of total RNA. RT-qPCR analysis was carried out using TaqMan gene expression assays (Thermo Fisher Scientific) and TaqMan Fast advance master mix (Thermo Fisher Scientific) according to the manufacturer’s instructions. The references of the TaqMan gene expression assays used are listed in Table S1 in Supplementary data. All reactions were performed in a final volume of 10 μL. The reaction protocol was 2 min at 50 °C, 10 min at 95 °C for activating the polymerase, and 40 cycles of 15 s at 95 °C and 1 min at 60 °C. Relative expression of genes was normalized using GAPDH as the endogenous control. Gene expression in each sample was calculated as 2^−ΔΔCt^.

## Results

### Effect of the nanosystem on the energy metabolism of tumoral cells

Before evaluating the effect of the proposed nanosystem on the energy metabolism of exposed tumoral cells, an analytical characterization of the material (Figs. S1 and S2 and Table S2 in Supplementary data) as well as assessment of its cytotoxicity (Fig. S3 in Supplementary data) has been carried out.

**Fig. 1:**
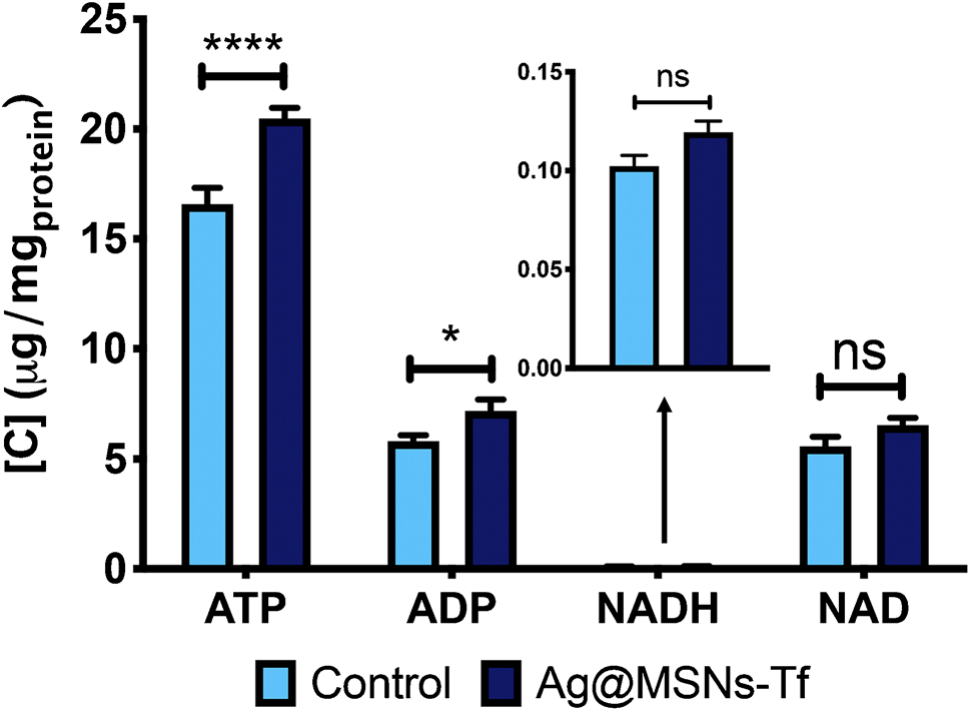
Concentration levels of ATP, ADP, NADH, and NAD^+^ metabolites in HepG2 cells after exposure to 10 µg/mL of Ag@MSNs-Tf for 72 h. Data were analyzed by ANOVA followed by Bonferroni’s multiple-comparison test. Statistical significance: *p < 0.05; ****p < 0.0001

An important aspect when testing the anti-tumor potential of a drug is to check that it affects the energy metabolism of tumor cells, which is normally increased. For this reason, we evaluated the levels of the four key metabolites in energy metabolism (ATP, ADP, NADH, and NAD^+^) in cells exposed to the nanosystem (Ag@MSNs-Tf) in comparison to control cells. For this purpose, a targeted metabolomics method based on the MRM mode was employed. As shown in Fig. 1, the levels of both ATP and ADP were significantly increased after exposure to 10 µg/mL Ag@MSNs-Tf for 72 h, thus showing alteration of the energy-related machinery in treated cells. On the contrary, the levels of NADH and NAD^+^ remained unchanged at the tested exposure condition.

**Fig. 2:**
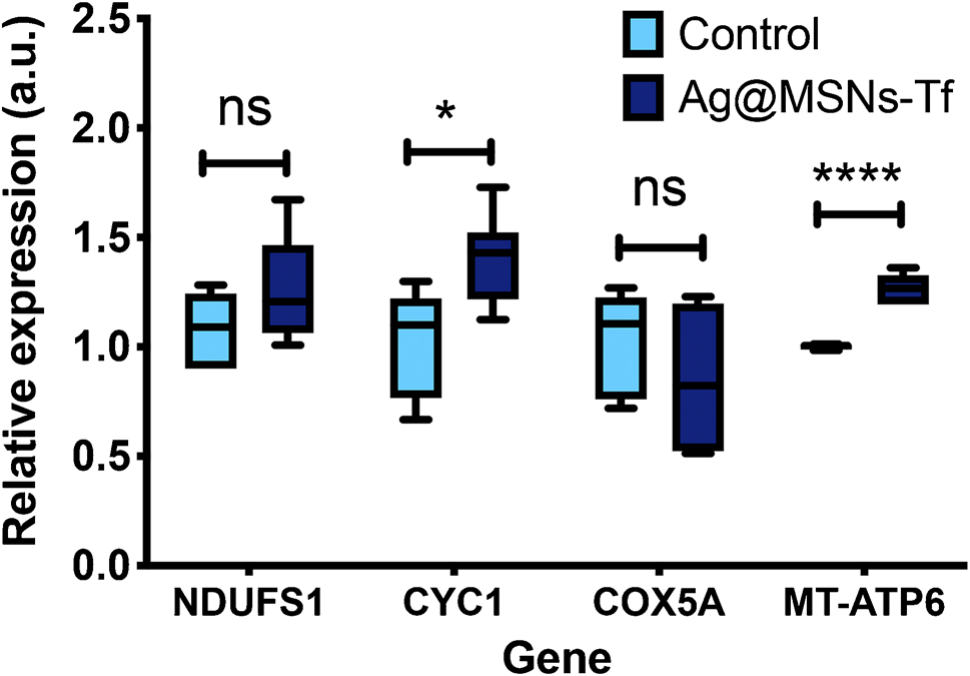
mRNA levels of genes involved in the electron transport chain complexes in HepG2 cells exposed to 10 µg/mL of Ag@MSNs-Tf for 72 h. Data were analyzed by ANOVA followed by Bonferroni’s multiple-comparison test. Statistical significance: *p < 0.05; ****p < 0.0001

Since ATP levels were found to be significantly increased in cells exposed to the nanosystem, it was decided to evaluate whether this effect was related to an alteration in the expression levels of genes involved in the electron transport chain (ETC). For this purpose, four transcripts (NDUFS1, CYC1, COX5A, and MT-ATP6) were analyzed by RT-qPCR. CYC1 and MT-ATP6 were found significantly overexpressed in HepG2 cells after exposure to the nanosystem (Fig. 2); thus confirming the enhanced activity of the ETC. Furthermore, the overexpression of these two transcripts is also related to an increase in ATP synthesis, which can be well related to the results obtained by targeted metabolomics, where ATP levels were found to be significantly increased in cells treated with the nanosystem.

### Untargeted metabolomics results

Metabolites with a NIST Rmatch higher than 700 were considered in order to guarantee the reliability of the identification. Based on this criterion, 26 metabolites present in both samples (control and cells exposed to the nanosystem) were quantified, including fatty acids, sugars, and amino acids, among other types of molecules (Table S3 in Supplementary material). To evaluate the similarity between the concentration levels of the metabolites, the Pearson correlation index (*r*) was used [23], which for 95% confidence would be 0.334 and 0.312 for *n* = 25 and *n* = 40, respectively. However, applying a stricter criterion, only correlations with an *r* value greater than 0.6 were taken into account (Table S4 in Supplementary material). Among these data, malic acid and isocitric acid (*r* = 0.8716); malic acid and ribitol (*r* = 0.8323); malic acid and phosphoric acid (*r* = 0.8149); malic acid and glutamine (*r* = 0.8026); isocitric acid and galactopyranose (*r* = 0.8232); *D*-ribofuranose and *L*-threonine (*r* = 0.8121); glutamine and *D*-ribofuranose (*r* = 0.8633); glutamine and erythritol (*r* = 0.8563); and *L*-threonine and erythritol (*r* = 0.8979) are worth noting since they showed a high positive correlation (> 0.8), which means that if the levels of one increased, the levels of the other increased similarly.

**Fig. 3:**
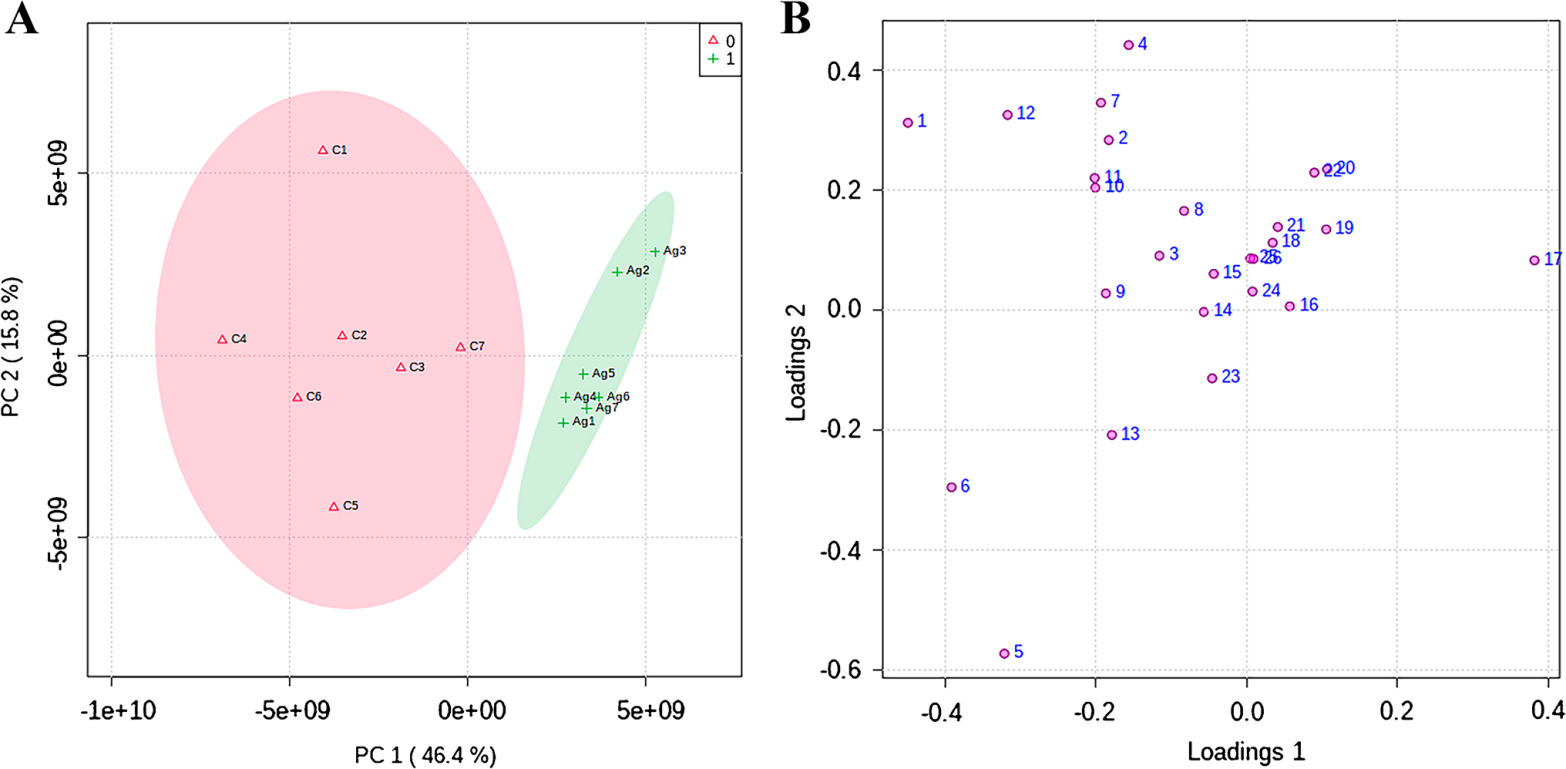
PCA results from the GC–MS data. **A** 2D scores plot of the first principal component (PC1) versus the second principal component (PC2) for control (*n* = 7) (red area) and cells treated with 10 µg/mL of Ag@MSNs-Tf for 72 h (*n* = 7) (green area). **B** Loading plot of PC1 versus PC2 for the 26 quantified metabolites (the number of each metabolite correlates with the identified metabolites as shown in Supplemental Table S3)

Subsequently, a principal component analysis (PCA) was performed on the quantified metabolites in order to detect metabolic alterations and patterns. This analysis allowed the identification of two distinct groups based on the first two principal components (Fig. 3A), which comprised 62.2% of the variance explained. On the other hand, principal component 1 (PC1), which explained 46% of the variance explained, was able to clearly separate the two experimental groups. The contribution of the variables (metabolites) represented by the loadings plot (Fig. 3B) showed the metabolites contributing the most to the separation of the groups: *L*- serine (23), rythonic acid (13), phosphoric acid (6), *L*-aspartic acid (5), and lauric acid (17).

Finally, using Student’s *t*-test (*p*-value < 0.05), it was found that 19 of the 26 quantified analytes showed significant differences in terms of concentration level when comparing control cells and cells treated with Ag@MSNs-Tf. Table 1 shows the 19 altered metabolites and includes their chromatographic retention time and Rmatch, confirming their correct identification. The table also includes the *R*_M_ for each metabolite, which is defined as the ratio of the mean of the peak areas for each compound found in cells exposed to the nanosystem to the mean of the peak areas for the same compounds in control cells.

**Table 1:** Altered metabolites (*p*-value < 0.05) in HepG2 cells exposed to 10 µg/mL of Ag@MSNs-Tf for 72 h

Compound	Retention time (min)	NIST Rmatch	*R*_M_
Lauric acid	21.802	788	8.03
β-*D*-Glucopyranose	27.187	828	2.76
Myo-Inositol	27.982	757	2.56
Stearic acid	33.362	813	2.14
Palmitic acid	28.350	727	2.00
*D*-Glucose	25.947	710	1.76
Pantothenic acid	27.239	719	0.63
Isocitric acid	24.247	789	0.36
Galactopyranose	24.747	778	0.33
Glutamine	20.587	735	0.30
*D*-Ribofuranose	20.427	884	0.19
Phosphoric acid	23.244	825	0.17
*L*-Threonine	15.787	860	0.17
Ribitol	22.521	805	0.12
Erythritol	18.376	732	0.12
*L*-Serine	14.190	848	0.09
*L*-Aspartic acid	18.598	845	0.05
Malic acid	17.988	765	0.03
Rythonic acid	19.063	777	0.02

### Transcriptome profiling

Transcriptomic analysis allowed the evaluation of more than 20,000 well-annotated human genes (Table S5 in Supplementary data). Table 2 lists the genes that were considered differentially expressed between control samples and cells exposed to Ag@MSNs-Tf. Genes with a log2 fold change above 1.2 (11 genes) or below 0.8 (11 genes) were considered altered at a significance level of 95%. The altered genes were found to be involved in different processes such as glycolysis, pentose phosphate pathway (PPP), electron transport chain (ETC), oxidative phosphorylation (OXPHOS), and amino acids transformation.

**Table 2:** Altered transcripts found in HepG2 cells exposed to 10 µg/mL of Ag@MSNs-Tf for 72 h

Gene name	Gene code	FC
Glucokinase (hexokinase 4) regulator	GCKR	1.79
Family with sequence similarity 173, member B	FAM173B	1.44
ATPase, Cu + + transporting, alpha polypeptide	ATP7A	1.42
Cystathionine gamma-lyase	CSE	1.39
Growth arrest and DNA-damage-inducible, gamma interacting	GADD45GIP1	1.38
Cystathionine-beta-synthase	CBS	1.35
Asparagine synthetase (glutamine-hydrolyzing)	ASNS	1.33
DnaJ (Hsp40) homolog, subfamily C, member 30	DNAJC30	1.28
Acetyl-CoA carboxylase alpha	ACACA	1.26
Glutaminase 2 (liver, mitochondrial)	GLS2	1.24
Ribose 5-phosphate isomerase A	RPIA	1.24
Aldolase C, fructose-bisphosphate	ALDOC	0.79
Solute carrier family 2 (facilitated glucose transporter), member 10	SLC2A10	0.79
Phosphoglycerate kinase 1	PGK1	0.78
Solute carrier family 2 (facilitated glucose/fructose transporter), member 5	SLC2A5	0.78
Aldolase B, fructose-bisphosphate	ALDOB	0.77
Tafazzin	TAZ	0.75
6-phosphofructo-2-kinase/fructose-2,6-biphosphatase 3	PFKFB3	0.65
Solute carrier family 2 (facilitated glucose transporter), member 14	SLC2A14	0.54
6-phosphofructo-2-kinase/fructose-2,6-biphosphatase 4; microRNA 6823	PFKFB4	0.50
Solute carrier family 2 (facilitated glucose transporter), member 3	SLC2A3	0.50
Glucose-6-phosphatase, catalytic subunit	G6PC	0.42

## Discussion

In the present study, the biomolecular mechanisms responsible for the antitumor action observed for the new Ag@MSNs-Tf nanosystem have been studied in depth, using a multi-omic approach based on transcriptomics and both targeted and untargeted metabolomics.

In the first experiment, the state of the cellular energy machinery after treatment has been evaluated by means of targeted metabolomics, showing a deregulation based on a significant increase in ATP and ADP levels in the treated cells (Fig. 1). This alteration in ATP and ADP levels is also supported by the overexpression of CYC1 and MT-ATP6 genes found by RT-qPCR, since both genes are related to ATP synthesis. While ATP is the major source of energy for tumoral and non-tumoral cells for maintaining their viability [24], and it is known that ATP levels are increased in most tumors to satisfy the increased energy demand due to its fast growth, it has also been shown that ATP is also needed for apoptosis to occur, since processes as the apoptosome complex formation or the processing of pro-caspase 9 are ATP-dependent [25]. Such processes were previously proved by protein expression analysis to be induced in cells after treatment with Ag@MSNs-Tf [14, 20], which would explain the elevated levels of ATP found. As for the increased ADP levels induced by the nanosystem, it is known that the levels of this metabolite are usually increased as a consequence of ATP consumption, so higher ATP levels could lead to an increase in ADP levels. In addition, ADP levels have been reported to be related to the rate of oxidative phosphorylation (OXPHOS), since an increase in ADP levels produces a rise in the activity of this process [26]. However, since cancer cells reprogram their energetic metabolism to obtain ATP mostly from glycolysis, in a process known as the “Warburg effect” [27], it is still necessary to establish the source of the increase in ATP levels found in cells treated with Ag@MSNs-Tf nanosystem.

In this sense, the untargeted metabolomics analysis has allowed the acquisition of information about the status of the pathways involved in ATP production and other metabolic pathways. Metabolites of different natures such as carbohydrates, amino acids, tricarboxylic acid (TCA) cycle intermediates, fatty acids, and inorganic compounds have been identified. However, only 26 of these metabolites met the quantification criteria.

**Fig. 4:**
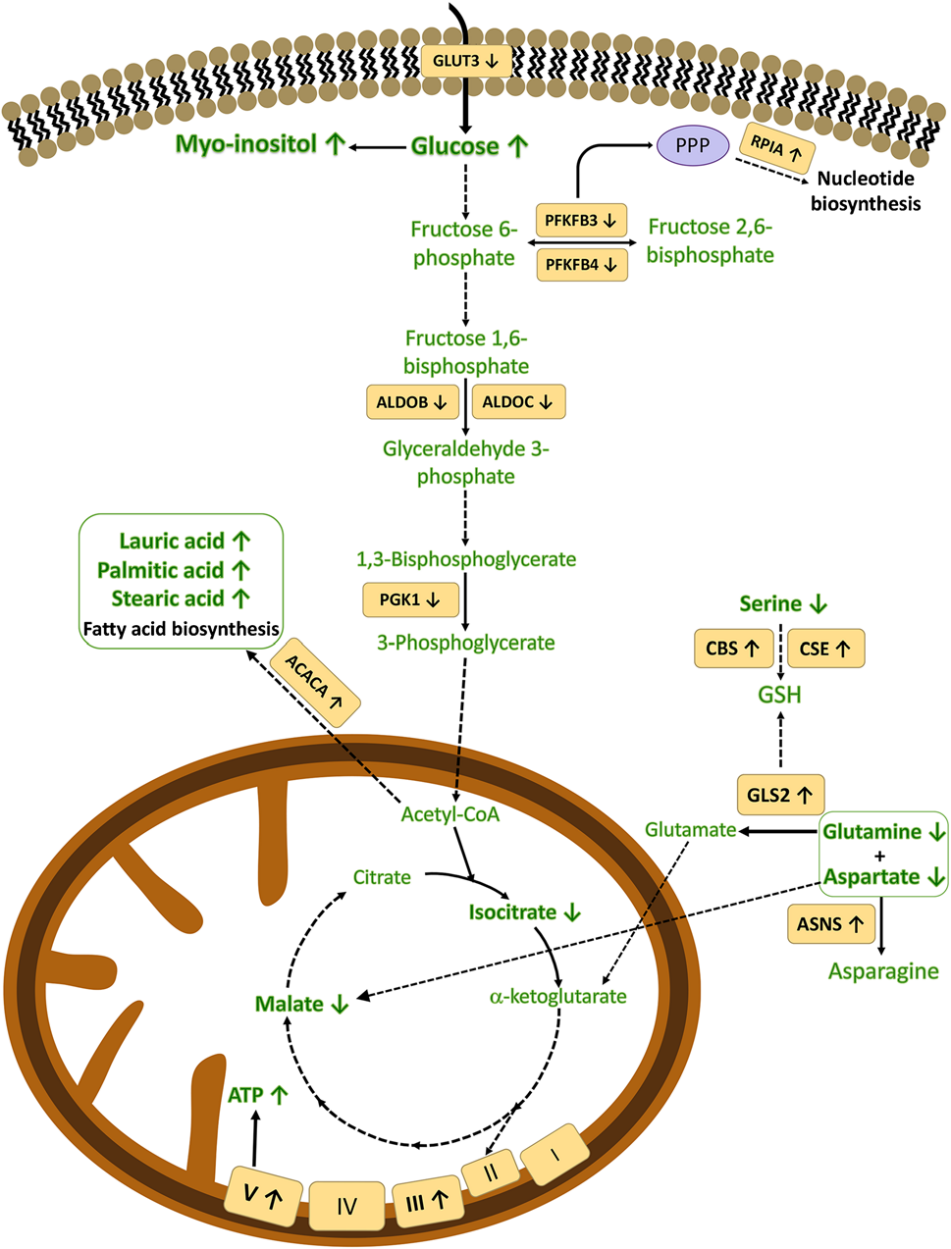
Illustration of the main biochemical pathways responsible for the antitumoral potential of Ag@MSNs-Tf

Out of these 26 metabolites, 19 were found to be significantly deregulated in HepG2 cells after exposure to the Ag@MSNs-Tf nanosystem (Table 1). Regarding the transcriptome profiling, 22 transcripts codifying enzymes involved in different metabolic pathways (Table 2) were found altered upon treatment with Ag@MSNs-Tf. Considering the set of metabolites and transcripts found by the multi-omics approach and that appear altered in HepG2 cells after treatment with the proposed nanosystem, it is possible to conclude that there are 4 main mechanisms by which this nanosystem exerts its anti-tumor action: the glycolysis, the pentose phosphate pathway (PPP), the oxidative phosphorylation, and the fatty acid biogenesis (Fig. 4). These mechanisms will be discussed below, including the pieces of evidence found in the omics analysis carried out.

### Effect on glycolysis

As commented before, tumoral cells usually reprogram their energetic metabolism through a shift from OXPHOS to glycolysis due to the Warburg effect [28, 29]. Out of all the metabolites found deregulated, the increased levels of 2 isoforms of glucose (D-glucose (*R*_M_ = 1.76) and D-glucopyranose (*R*_M_ = 2.76)) are worth noting in this context. This high glucose level could be a symptom of the increased uptake of glucose and the activation of the glycolysis pathway as part of the Warburg effect, since it is known that cancer cells increase the uptake of glucose to generate glycolytic intermediates required for the synthesis of carbohydrates, fatty acids, and proteins, and to increase ATP production, as it has been observed after the exposure to Ag@MSNs-Tf (Fig. 1) [30, 31]. Taking this into account, the mRNA expression levels of all members of the glucose transporters SLC2A family were assessed after exposure to the nanosystem, finding 4 of them deregulated (SLC2A3, SLC2A5, SLC2A10, and SLC2A14). In the case of SLC2A5 (FC = 0.78) and SLC2A10 (FC = 0.79), these two transporters have low affinity for glucose as they are mainly involved in the transport of fructose in the case of SLC2A5 (also known as GLUT5) [32], and dehydroascorbic acid in the case of SLC2A10 (known as GLUT10) [33, 34]. Despite this fact, the downregulation of both transporters might have important effects on cancer cells, since the knockdown of GLUT5 has been proved to deplete fructose uptake and decrease cell proliferation [32]. As for GLUT10, this transporter is implicated in the maintenance of ascorbic acid homeostasis under oxidative stress conditions, so the downregulation of GLUT10 may promote the proliferation of ROS [33, 34]. Of special importance is the depletion of mRNA levels of SLC2A3 (or GLUT3) (FC = 0.50) because of the high affinity of this transporter towards glucose [35]. It has been demonstrated that hyperglycemia (as it has been observed in the study) induces the downregulation of GLUT3, and that the inhibition of this transporter was able to suppress the tumorigenic potential of hepatocarcinoma cells, since GLUT3 is involved in different pathways such as cAMP, NF-κβ, and p53 signaling [36, 37]. The transporter SLC2A14 (GLUT14), which has a strong similarity to GLUT3, was also found downregulated (FC = 0.54) [38, 39]. Thus, after analyzing the expression levels of the glucose transporters, it can be hypothesized that the observed accumulation of glucose was not due to an increase in the uptake of this metabolite, but because of an impairment in the glycolytic degradation of glucose. To support this hypothesis, the expression levels of all the transcripts codifying the different enzymes involved in the glycolysis pathway were additionally assessed. Among all the evaluated transcripts, two isoforms of the fructose-bisphosphate aldolase transcript, ALDOB (FC = 0.77) and ALDOC (FC = 0.79), which catalyzes the transformation of fructose 1,6-bisphosphate to glyceraldehyde 3-phosphate, were found downregulated. Nevertheless, the effect of the impairment of these isoforms on glycolysis is not clear since the downregulation of ALDOC produces contradictory effects on cell proliferation and migration in glioblastoma cells and in vivo [40]. Moreover, ALDOs have been demonstrated not to be the rate-controlling enzymes of the glycolysis pathway, since ALDOA downregulation does not affect intracellular ATP levels [41]. Another glycolytic transcript found deregulated after exposure to Ag@MSNs-Tf was the phosphoglycerate kinase 1 (PGK1, FC = 0.78) which catalyzes the transformation of 1,3-bisphosphoglycerate to 3-phosphoglycerate. Interestingly, overexpression of PGK1 has been observed in different types of tumors including liver cancer, and downregulation of PGK1 has been proved to reduce cell proliferation and tumorigenesis. Additionally, the knockdown of PGK1 has been demonstrated to inhibit glycolysis [42, 43]. In addition, the results pointed out a strong downregulation of the transcripts PFKFB3 (FC = 0.65) and PFKFB4 (FC = 0.50), which are two isoforms that catalyze the reversible transformation between fructose 2,6-bisphosphate (F2,6BP) and fructose-6-phosphate (F6P). PFKFB3 is a rate-limiting enzyme that has a strong kinase activity promoting the formation of F2,6BP and is usually upregulated in a wide variety of tumors, including hepatocellular carcinoma, where F2,6BP acts as an allosteric modulator promoting the activation of the glycolytic flux [44–46]. Thus, the downregulation of PFKFB3 leads to a decrease in the glycolytic flux resulting in a reduction in the cell growth of tumoral cells [44]. Moreover, PFKFB3 inhibition can be due to the presence of ROS (whose generation has been shown to be induced by exposure to AgNPs [45]) and redirects the glucose flux from glycolysis to the pentose phosphate pathway to increase nucleotide biosynthesis, regeneration of glutathione (GSH), and ROS scavenging [46, 47]. PFKFB4 has a more active kinase activity than phosphatase, and it is also overexpressed in several human cancers. PFKFB4 inhibition induces a decrease in F2,6BP levels and thus reduces the glycolytic flux and glucose uptake [48]. Other transcripts involved in the glycolysis pathway regulation were also found deregulated. Such is the case of glucose-6-phosphatase (G6PC) and the hexokinase 4 regulator (GCKR). The downregulation of G6PC as it was observed (FC = 0.42) permits tumoral cells to overcome glycolytic inhibition, acting as a survival mechanism [49]; although it has also been reported that loss of G6PC is implicated in the reduction of glucose levels [50]. In addition, the glucokinase regulator (GCKR) was found overexpressed (FC = 1.79) since its transcription is induced by high glucose levels to avoid the accumulation of glucose-6 phosphate [51].

### Effect on the pentose phosphate pathway (PPP) and other minor pathways

In addition to all the above, different metabolites implicated in the PPP were found deregulated in HepG2 cells after exposure to Ag@MSNs-Tf. Among all of them, the downregulation of *D*-ribose (R_M_ = 0.19) should be pointed out since it is an essential component of key molecules such as DNA, RNA, acetyl-CoA, NADPH, and ATP [52, 53]. Ribose levels are increased in tumoral cells to support several cellular processes, so the depletion of this metabolite might have important effects on different metabolic processes [52, 54]. The transcript ribose 5-phosphate isomerase (RPIA), which was found slightly upregulated (FC = 1.24), is related to ribose metabolism. RPIA is usually upregulated after the activation of mTORC1 signaling, which redirects the glycolytic flux to generate ribose 5-phosphate and thus increases nucleotide synthesis [55, 56]. Apart from ribose, other byproducts of the PPP were found downregulated after treatment with Ag@MSNs-Tf such as ribitol (*R*_M_ = 0.12), erythritol (*R*_M_ = 0.12), or erythronic acid (*R*_M_ = 0.02), what might be a symptom of the shift of the PPP towards the synthesis of ribose 5-phosphate to increase the nucleotide synthesis.

On another hand, the levels of myo-inositol (*R*_M_ = 2.56) were found increased after exposure to the nanosystem. Since myo-inositol is endogenously synthesized from glucose, this result was expected due to the high levels of glucose found in the experiment as commented before [57]. Additionally, this result is particularly interesting because it has been described that myo-inositol might contribute to the inhibition of carcinogenesis in different organs such as the liver or lungs, since it is able to disrupt the PI3K/Akt survival pathway of tumoral cells and thus the cellular growth and cell cycle progression of such cells [57, 58]. Furthermore, galactose levels have been shown to be depleted in our study (*R*_M_ = 0.33), what might involve the alteration of the galactose metabolism. The impairment of the galactose metabolism induces the downregulation of the PI3K/Akt pathway as well, and the reduction of galactose uptake might force the cells to use *L*-glutamine to obtain energy [59, 60].

### Effect on amino acid–related pathways

Among others, the amino acid *L*-glutamine was found highly downregulated (*R*_M_ = 0.30) after exposure to Ag@MSNs-Tf. It has been reported that glutamine depletion induces cell cycle arrest, as this metabolite plays an important role in cancer growth since, alongside glycine and aspartate, it is involved in the biosynthesis of purine and pyrimidine [28, 61]. Besides, glutamine is an important TCA anaplerosis metabolite, since it can generate α-ketoglutarate via its previous conversion to glutamate [61, 62]. The transformation of glutamine into glutamate is catalyzed by the enzyme glutaminase (GLS2), whose transcript was found somewhat upregulated (FC = 1.24). Thus, the overexpression of this transcript could respond to the need of the cells to replenish the levels of some metabolites involved in the TCA cycle. Moreover, the overexpression of GLS2 has been proved to have antiproliferative and tumor suppression properties in tumor cells since it also downregulates the PI3K/Akt signaling pathway [63, 64]. In addition, and besides its anti-proliferative activity, GLS2 overexpression is related to oxidative stress by contributing to the reduction of the intracellular levels of ROS through the production of glutathione (GSH) [63–65]. Apart from its role in TCA anaplerosis, glutamine is also involved in the biosynthesis of asparagine, acting as an amide group donor which is accepted by aspartate [61, 62]. This reaction is catalyzed by the enzyme asparaginase (ASNS), whose transcript was found upregulated after exposure to Ag@MSNs-Tf (FC = 1.33). Moreover, ASNS acts as a tumor growth suppressor and increases the sensibility of tumoral cells to apoptosis, since its depletion produces the accumulation of aspartate and glutamine [66]. Thus, the upregulation of GLS2 along with the overexpression of ASNS may explain the reduced levels of glutamine observed in the experiment. As in the case of glutamine, aspartate (*R*_M_ = 0.05) also contributes to TCA cycle anaplerosis by generating citrate and malate, while it also takes part in nucleotide biosynthesis [67]. Although a strong decrease in aspartate levels was found in cells exposed to the nanosystem, none of the transcripts involved in neither the TCA cycle nor the nucleotide biosynthesis were found deregulated. This fact may mean that, like glutamine, aspartate is being consumed to produce arginine. Also, aspartate has been suggested to be a limiting metabolite for tumor growth and cell proliferation under hypoxic conditions, and reduced aspartate levels increase the dependence on glutaminase to replenish the TCA cycle [67, 68]. Despite the role of both glutamine and aspartate in TCA cycle anaplerosis, depleted levels of malate (*R*_M_ = 0.03) and isocitrate (*R*_M_ = 0.36) were found after exposure to the nanosystem, what can be due to the diversion of the glycolysis towards the PPP, since pyruvate may not be produced at the same level, or because of an increased activity of the TCA cycle and OXPHOS (pathway related to TCA cycle) to produce higher amounts of ATP.

Other amino acids such as threonine (*R*_M_ = 0.17) or serine (*R*_M_ = 0.09) were also found significantly deregulated. After exposure to Ag@MSNs-Tf, threonine levels were reduced, what may lead to cell death [69]. Moreover, it has been reported that depleted threonine levels affect S-adenosyl-homocysteine levels, which is a molecule necessary for tumor survival [61]. Even more interesting is the observed reduction in the serine levels which might have an impact on different processes. On one hand, serine is involved in the biosynthesis of GSH, acting as a substrate in the formation of cysteine, the limiting reagent in this process [61, 69, 70]. The reduction of serine levels has been related to the overexpression of cystathionine β-synthase (CBS), an enzyme that, together with cystathionine λ-lyase (CSE), is involved in the synthesis of GSH [29, 71, 72] and in cell proliferation and tumor growth [70]. Interestingly, the transcriptomics experiment confirmed this fact, since the transcripts encoding for both enzymes, CBS and CSE, were found upregulated (FC = 1.35 and FC = 1.39, respectively) upon Ag@MSNs-Tf exposure. On the other hand, serine is implicated in the biosynthesis of nucleotides (via the folate cycle) through its conversion to glycine in a reaction catalyzed by the enzyme serine hydroxymethyltransferase (SHMT2, FC = 1.08), and in the biosynthesis of other key biomolecules such as phospholipids. For this reason, a reduction in serine levels has been proved to inhibit tumor growth [73, 74]. Furthermore, upon serine starvation, a decrease in pyruvate kinase (PKM2) activity is induced, and consequently pyruvate is diverted to the TCA cycle [69, 75]. In the transcriptomics study, only the expression levels of the PKM transcript (FC = 0.88) were found slightly downregulated (below the established level). Under this situation, OXPHOS could acquire an important role in supporting cellular growth [75], what might explain the increased ATP levels observed in the targeted metabolomics assay (Fig. 1).

### Effect on oxidative phosphorylation (OXPHOS)

As commented above, the glycolytic flux has been proved to be impaired in HepG2 cells exposed to Ag@MSNs-Tf; thus, the observed increase in the ATP levels might be due to an activation of oxidative phosphorylation (OXPHOS). Taking this into account, the expression levels of different transcripts involved in the ATP biosynthetic process and the OXPHOS were evaluated. Among all the deregulated transcripts, the upregulation of the ATP synthase subunit C lysine *N*-methyltransferase (FAM173B, FC = 1.44) should be highlighted, since it is involved in the assembly of the ATP synthase complex. Its expression is also related to the ATP synthesis process through the OXPHOS and to the mitochondrial respiration activity [76]. Additionally, the transcript encoding the DnaJ heat shock protein family (Hsp40) member C30 (DNAJC30), which was also found slightly upregulated (FC = 1.28), has been related to the ATP synthase complex. In this sense, it has been reported that the decreased expression of DNAJC30 results in reduced ATP levels [77, 78]. In addition, the gamma interacting protein 1 (GADD45GIP1), which is essential for the formation of the OXPHOS complexes, was found overexpressed (FC = 1.38) after exposure to Ag@MSNs-Tf, thus confirming the induced OXPHOS activity and the increased ATP production [79]. Interestingly, the copper-transporting ATPase 1 (ATP7A), which is implicated in the regulation of the OXPHOS, was also found upregulated (FC = 1.42). Although this ATPase is implicated in the transport of copper, it has been reported that ATP7A is able to carry silver ions and that the excess of both binding metals can increase the expression levels of this transporter [80, 81]. Thus, the increased ATP levels found in the experiment might also be a response of the cells to expel the silver ions generated from the Ag core of the nanosystem. In the same direction, we can highlight the downregulation found for the transcript encoding tafazzin (TAZ) (FC = 0.75), which is a protein involved in the maintenance of the mitochondrial membrane potential, thus indicating the impairment of the mitochondrial membrane potential after the exposure to the nanosystem [82].

Based on the above and to confirm the effect of Ag@MSNs-Tf on the electron transport chain (ETC) and the ATP production through the OXPHOS in treated cells, a RT-qPCR analysis was carried out to evaluate the expression levels of the following transcripts: NDUFS1, COX5A, CYC1, and MT-ATP6 (Fig. 2). The results showed that both CYC1 and MT-ATP6 were found significantly overexpressed in cells exposed to the nanosystem, confirming the enhanced activity of the ETC. Furthermore, the overexpression of these two transcripts is also related to an increase in ATP synthesis [83–85]. Thus, the previous hypothesis about the source of the increased ATP levels observed after exposure to the nanosystem can be confirmed and attributed to an enhanced activity of the OXPHOS, and specifically of the ETC complexes III and V.

### Effect on fatty acid metabolism

Apart from all the above, we observed the accumulation of different fatty acids such as lauric acid (*R*_M_ = 8.03), palmitic acid (*R*_M_ = 2.00), and stearic acid (*R*_M_ = 2.14) after exposure to Ag@MSNs-Tf. High levels of lauric acid have been proved to have toxic effects in different cell lines, including HepG2 cells [86]. The toxic properties of lauric acid are exerted through the induction of apoptosis due to the generation of ROS and the activation of the EGFR/ERK transduction pathway [87]. In the same way, the accumulation of palmitic and stearic acid is related to an increase in cellular apoptosis through different mechanisms in hepatocytes [88, 89]. In the case of palmitic acid, the apoptotic action is displayed through the activation of PPARα, the production of ceramide, the generation of NO and ROS, the induction of endoplasmic reticulum stress, or the activation of the factor κβ [90]. On another hand, stearic acid exerts its apoptotic action similarly to lauric acid, through the inhibition of the EGFR [91]. In order to explain the accumulation of these fatty acids, the expression levels of several transcripts involved in the biosynthesis and the β-oxidation of the fatty acids were evaluated. The most interesting transcript found slightly overexpressed was the acetyl-CoA carboxylase 1 (ACACA) (FC = 1.26), which is thought to be a fatty acid–controlling isoform. Its overexpression is related to the enhanced capacity of tumoral cells for de novo lipogenesis, what might explain the increased levels of the different fatty acids found upon Ag@MSNs-Tf exposure [92, 93]. Additionally, the levels of the coenzyme A precursor, the pantothenic acid, were depleted (*R*_M_ = 0.63) after exposure to the nanosystem. This molecule leads to the formation of CoA with ATP, so it can be thought that the depleted levels of this precursor, along with the high levels of ATP found, mean a greater formation of CoA, needed for the synthesis of fatty acids and the operation of the TCA cycle [94, 95].

## Conclusions

This study has unravelled some of the biomolecular mechanisms by which the hematocompatible Tf decorated core–shell Ag@MSNs hybrid nanosystem exerts its antitumor action. For this purpose, targeted and untargeted metabolomics analyses have been carried out in combination with a microarray transcriptomics analysis. The targeted metabolomics analysis has shown an alteration of the energy metabolism, with increased levels of ATP and ADP in cells exposed to the nanosystem. Additionally, the untargeted metabolomics analysis has identified a series of metabolites of different natures (carbohydrates, amino acids, TCA intermediates, fatty acids, and other minor molecules) significantly deregulated in hepatocarcinoma cells treated with the anti-tumoral nanosystem. The results of the metabolomics study were supported by the results derived from the transcriptomics assay. Overall, and considering the indications and targets found in both experiments, it has been deduced that the Ag@MSNs-Tf nanosystem exerts its antitumor action through different mechanisms. On one hand, exposure to the nanosystem impairs the glycolytic pathway, reducing the glycolytic flux and the uptake of glucose through the downregulation of some membrane glucose transporters. On the other hand, it has been confirmed that treated cells activate the oxidative phosphorylation pathway to increase the ATP levels as a defense mechanism to expel silver cations and to favor the formation of the apoptosome complex, necessary to induce cellular apoptosis. These findings have provided essential information on the biomolecular mechanisms of action of this potential antitumor nanosystem. Furthermore, these outcomes open the door to new research in which additional drugs loaded into the pores of the nanosystem could target the defense systems that were found to be activated in tumor cells exposed to the unloaded Ag@MSNs-Tf nanosystem, thereby increasing the effectiveness of the treatment. We are currently carrying out research in this direction.

## Supplementary Information

Below is the link to the electronic supplementary material.

Supplementary file1 (DOCX 1453 KB)

Supplementary file2 (PDF 4498 KB)
