# Supplementary material for: Integrated transcriptomics and metabolomics analysis reveals the biomolecular mechanisms associated to the antitumoral potential of a novel silver-based core@shell nanosystem
Source: Mikrochim Acta. 2023 Mar 13;190(4):132. doi: 10.1007/s00604-023-05712-3 (PMC10011303; doi:10.1007/s00604-023-05712-3)

**Supplementary Information**

**Integrated transcriptomics and metabolomics analysis reveals the biomolecular mechanisms associated to the antitumoral potential of a novel silver-based core@shell nanosystem**

*Guillermo Aragoneses-Cazorla,^a^ María Vallet-Regí,^b,c^ Mª Milagros Gómez-Gómez,^a^*

*Blanca González,^b,c^ Jose L. Luque-Garcia^a,^**

^a^ Department of Analytical Chemistry, Faculty of Chemical Sciences, Complutense University of Madrid, 28040, Madrid, Spain.

^b^ Department of Chemistry in Pharmaceutical Sciences, Faculty of Pharmacy, Complutense University of Madrid, Instituto de Investigación Sanitaria Hospital 12 de Octubre (i+12), 28040, Madrid, Spain.

^c^ Centro de Investigación Biomédica en Red de Bioingeniería, Biomateriales y Nanomedicina (CIBER-BBN), Spain.

*Corresponding author

*E-mail address*: [jlluque@ucm.es](mailto:jlluque@ucm.es) (J.L. Luque-Garcia)

**Table S1.** References of TaqMan gene expression assays used for the RT-qPCR analysis.

| **Gene** | **Codified protein** | **Assay ID** |
| --- | --- | --- |
| **NDUFS1** | CI-75kD | Hs00192297_m1 |
| **COX5A** | Cytochrome c oxidase subunit 5A, mitochondrial | Hs00362067_m1 |
| **CYC1** | Cytochrome c-1 | Hs00357717_m1 |
| **MT-ATP6** | ATP synthase subunit a | Hs02596862_g1 |

**
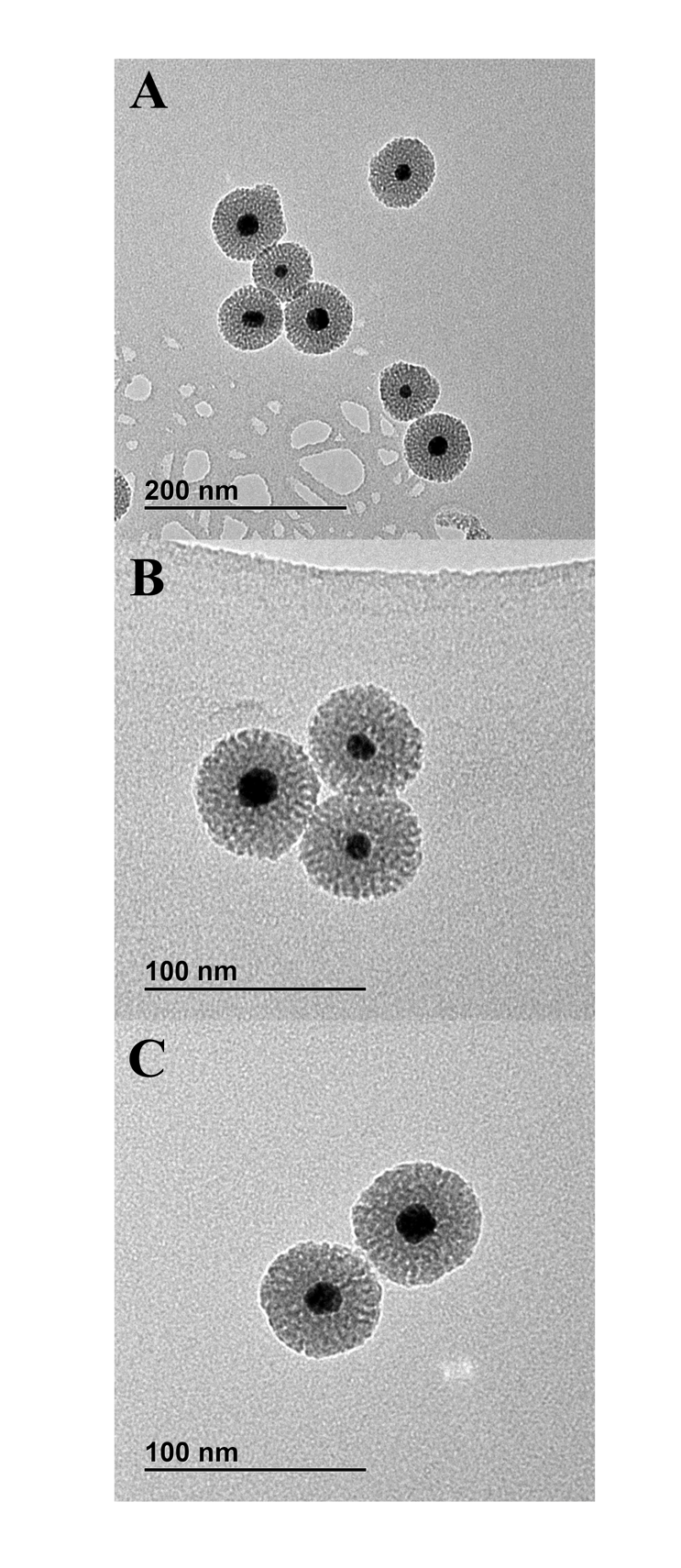
**

**Figure S1.** TEM micrographs of Ag@MSNs (A), Ag@MSNs-COOH (B) and Ag@MSNs-Tf (C) at different magnifications.

**Table S2.** Organic content from thermogravimetric analysis, ζ-potential values and hydrodynamic particle size in water medium of Ag@MSNs and functionalized Ag@MSNs materials.

| Material | Organic content (wt %) | ζ-Potential ^c^ (mV) | Hydrodynamic size ^c,d^ (nm) |
| --- | --- | --- | --- |
| Ag@MSNs | 4.7 ^a^ | −15 ± 1 | 66 ± 5 |
| Ag@MSNs-COOHext | 8.3 ^a,b^ | −24 ± 1 | 58 ± 9 |
| Ag@MSNs-Tf | 16.7 ^a,b^ | −19 ± 3 | 74 ± 8 |

^a^ Organic content (wt%) is determined from the TGA weight losses, excluding the weight loss due to the desorption of water (up to 125 ºC) and ^b^ further corrected by the weight loss of the remaining alkoxysilanes after the sol-gel reaction (surfactant extracted unmodified Ag@MSNs). ^c^ Samples were measured in quintuplicate (n = 5). ^d^ Maximum of the size distribution measured by dynamic light scattering.


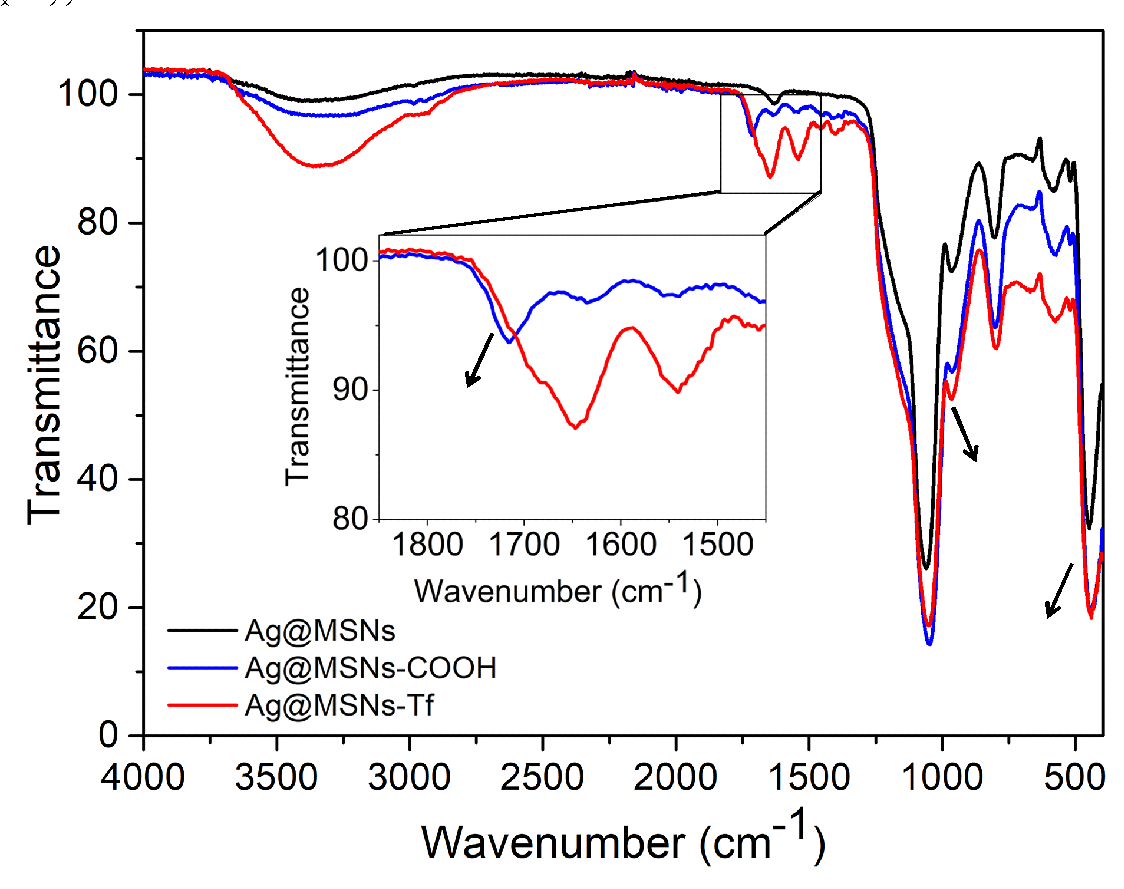


**Figure S2.** Fourier transform infrared (FTIR) spectra of Ag@MSNs, Ag@MSNs-COOH and Ag@MSNs-Tf nanosystems.


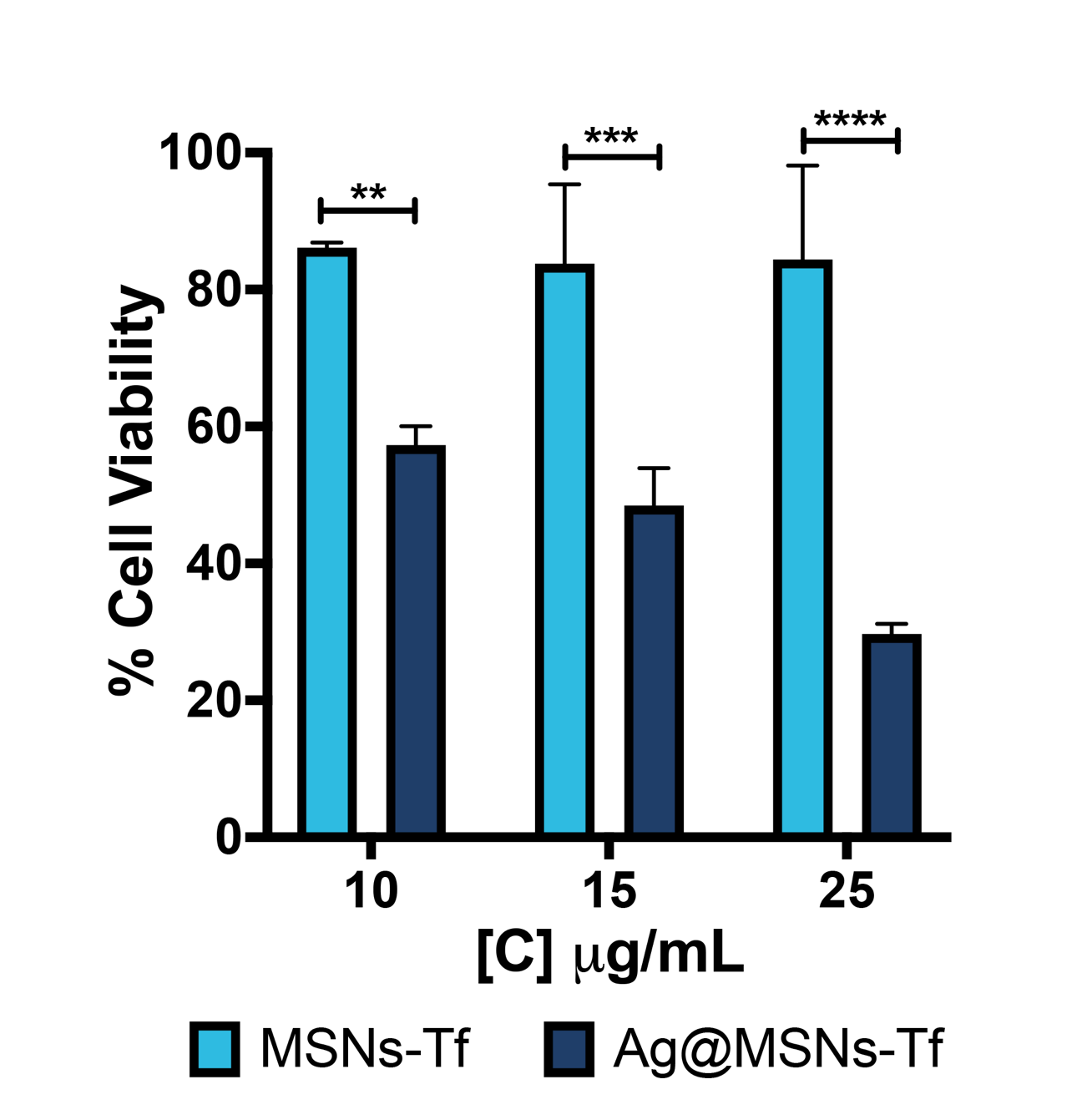


**Figure S3.** Cell viability of HepG2 cells exposed to different concentrations (10, 15 and 25 μg/mL) of either MSNs-Tf orAg@MSNs-Tf for 72 h. Statistical significance: ** p<0.01; *** p<0.001; **** p<0.0001.

**Table S3.** Quantified metabolites in HepG2 cells with a match factor up to 700.

| **Compound** | **Retention time (min)** | **NIST Rmatch** |
| --- | --- | --- |
| 1. Malic acid | 17.988 | 765 |
| 2. Isocitric acid | 24.247 | 789 |
| 3. Ribitol | 22.521 | 805 |
| 4. Galactopyranose | 24.747 | 778 |
| 5. *L*-Aspartic acid | 18.598 | 845 |
| 6. Phosphoric acid | 23.244 | 825 |
| 7. *L*-Proline | 18.702 | 880 |
| 8. Gluconic acid | 27.337 | 829 |
| 9. *D*-Ribofuranose | 20.427 | 884 |
| 10. Glutamine | 20.587 | 735 |
| 11. *L*-Threonine | 15.787 | 860 |
| 12. Erythritol | 18.376 | 732 |
| 13. Rythonic acid | 19.063 | 777 |
| 14. Myristic acid | 24.865 | 885 |
| 15. Palmitelaidic acid | 27.936 | 791 |
| 16. *L*-Lysine | 26.055 | 763 |
| 17. Lauric acid | 21.802 | 788 |
| 18. Oleic acid | 32.194 | 751 |
| 19. *β*-*D*-Glucopyranose | 27.187 | 828 |
| 20. Myo-Inositol | 27.982 | 757 |
| 21. Palmitic acid | 28.350 | 727 |
| 22. Stearic acid | 33.362 | 813 |
| 23. *L*-Serine | 14.190 | 848 |
| 24. Pantothenic acid | 27.239 | 719 |
| 25. *D*-Glucose | 25.947 | 710 |
| 26. *L*-Tyrosine | 26.293 | 826 |
| 4-Chlorophenylalanine (internal standard) | 23.756 |  |

**Table S4.** Pearson’s correlation matrix data for the 26 quantified metabolites.


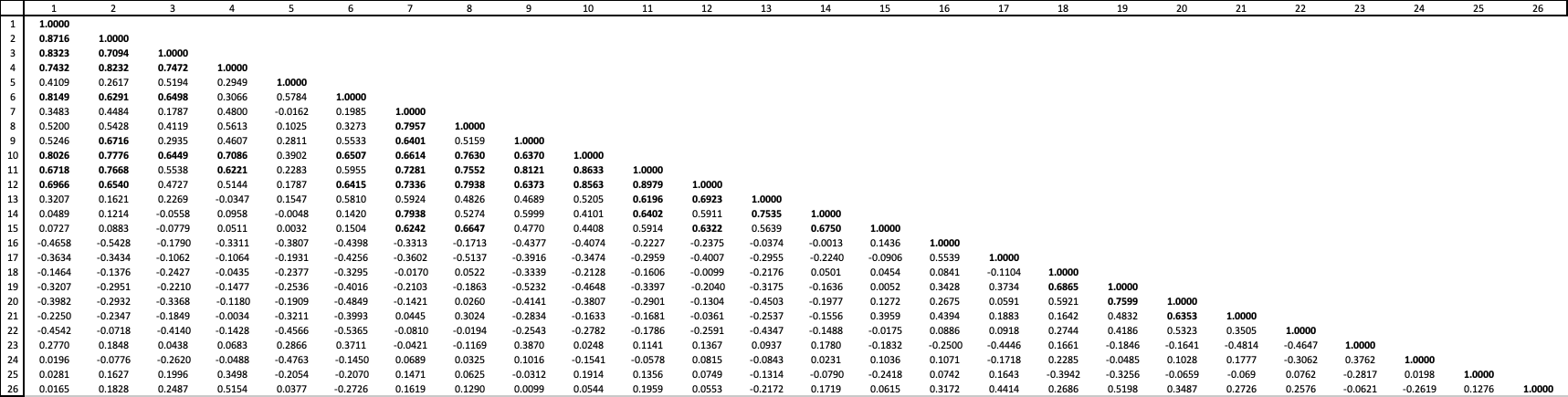

Supplement: Supplementary file 1 — Supplementary file1 (DOCX 1453 KB) [file 604_2023_5712_MOESM1_ESM.docx]
