# Supplementary material for: Integrated transcriptomics and metabolomics analysis reveals the biomolecular mechanisms associated to the antitumoral potential of a novel silver-based core@shell nanosystem
Source: Mikrochim Acta. 2023 Mar 13;190(4):132. doi: 10.1007/s00604-023-05712-3 (PMC10011303; doi:10.1007/s00604-023-05712-3)
Supplement: Supplementary file 2 — Supplementary file2 (PDF 4498 KB) [file 604_2023_5712_MOESM2_ESM.pdf]

Table S5. Whole transcriptome analysis of HepG2 cells exposed to Ag@MSNs-Tf

Gene name	Gene code	FC
Transcript Identified by AceView, Entrez Gene ID(s) 124936	CYB5D2	2,39
Transcript Identified by AceView, Entrez Gene ID(s) 3684	ITGAM	2,09
Memczak2013 ALT_ACCEPTOR, ALT_DONOR, coding, INTERNAL, intronic best transcript NM_021141	XRCC5	2,09
Transcript Identified by AceView, Entrez Gene ID(s) 10673	TNFSF13B	2,05
Transcript Identified by AceView, Entrez Gene ID(s) 64062	RBM26	2,04
HSPB (heat shock 27kDa) associated protein 1	HSPBAP1	2,03
Memczak2013 ANTISENSE, CDS, coding, INTERNAL best transcript NM_174956	ATP2A3	2,00
Transcript Identified by AceView, Entrez Gene ID(s) 54870	QRICH1	1,95
Memczak2013 ANTISENSE, coding, INTERNAL, intronic best transcript NM_001018	RPS15	1,94
Transcript Identified by AceView, Entrez Gene ID(s) 26275	HIBCH	1,93
Transcript Identified by AceView, Entrez Gene ID(s) 10915	TCERG1	1,93
long intergenic non-protein coding RNA 654	LINC00654	1,92
Memczak2013 ALT_ACCEPTOR, ALT_DONOR, coding, INTERNAL, intronic best transcript NM_017841	SDHAF2	1,89
Transcript Identified by AceView, Entrez Gene ID(s) 6660	SOX5	1,89
DEAD (Asp-Glu-Ala-Asp) box polypeptide 60-like	DDX60L	1,86
Transcript Identified by AceView, Entrez Gene ID(s) 123355	LRRC28	1,85
Jeck2013 ALT_ACCEPTOR, ALT_DONOR, coding, INTERNAL, intronic best transcript NM_015589	SAMD4A	1,84
Memczak2013 ALT_ACCEPTOR, ALT_DONOR, INTERNAL, intronic, ncRNA best transcript NR_023348	GAS8	1,83
Na+/K+ transporting ATPase interacting 2	NKAIN2	1,83
calcium channel, voltage-dependent, P/Q type, alpha 1A subunit	CACNA1A	1,83
pre-B-cell leukemia homeobox 4	PBX4	1,81
mab-21-like 2 (C. elegans)	MAB21L2	1,81
Transcript Identified by AceView, Entrez Gene ID(s) 2646	GCKR	1,79
Transcript Identified by AceView, Entrez Gene ID(s) 55278	QRSL1	1,79
Transcript Identified by AceView, Entrez Gene ID(s) 60468	BACH2	1,78
Memczak2013 ALT_ACCEPTOR, ALT_DONOR, coding, INTERNAL, intronic best transcript NM_170721	MSI2	1,76
cytochrome b-245, beta polypeptide	CYBB	1,76
neuronal calcium sensor 1	NCS1	1,76
coiled-coil domain containing 179	CCDC179	1,74
spermidine/spermine N1-acetyl transferase-like 1	SATL1	1,73
glucocorticoid modulatory element binding protein 1	GMEB1	1,72
Memczak2013 ANTISENSE, coding, INTERNAL, intronic best transcript NM_003335	UBA7	1,70
Transcript Identified by AceView, Entrez Gene ID(s) 64327	LMBR1	1,70
EPH receptor A1	EPHA1	1,70
Transcript Identified by AceView, Entrez Gene ID(s) 5257	PHKB	1,69

5-hydroxytryptamine (serotonin) receptor 3B, ionotropic	HTR3B	1,69
protein tyrosine phosphatase, receptor type, f polypeptide (PTPRF), interacting protein (liprin), alpha 4	PPFIA4	1,68
taste receptor, type 2, member 19	TAS2R19	1,68
arachidonate 15-lipoxygenase	ALOX15	1,68
family with sequence similarity 189, member A2	FAM189A2	1,68
Transcript Identified by AceView, Entrez Gene ID(s) 23670	TMEM2	1,67
EF-hand calcium binding domain 13	EFCAB13	1,67
caudal type homeobox 1	CDX1	1,67
Memczak2013 ALT_ACCEPTOR, ALT_DONOR, coding, INTERNAL, intronic best transcript NM_172373	ELF1	1,66
keratin associated protein 5-2	KRTAP5-2	1,66
tektin 3	TEKT3	1,66
UDP glycosyltransferase 3 family, polypeptide A1	UGT3A1	1,65
microtubule-associated protein, RP/EB family, member 3	MAPRE3	1,65
zinc finger, DHHC-type containing 11	ZDHHC11	1,65
Transcript Identified by AceView, Entrez Gene ID(s) 23126	POGZ	1,65
PROP paired-like homeobox 1	PROP1	1,64
EF-hand calcium binding domain 1	EFCAB1	1,64
zinc finger with KRAB and SCAN domains 7	ZKSCAN7	1,64
EGF-like repeats and discoidin I-like domains 3	EDIL3	1,63
dispatched homolog 1 (Drosophila)	DISP1	1,63
HPN antisense RNA 1	HPN-AS1	1,63
Transcript Identified by AceView, Entrez Gene ID(s) 196527	ANO6	1,63
musashi RNA binding protein 1	MSI1	1,62
Transcript Identified by AceView, Entrez Gene ID(s) 92691	TMEM169	1,62
centrosomal protein 72kDa	CEP72	1,61
outer dense fiber of sperm tails 2-like	ODF2L	1,61
sperm antigen with calponin homology and coiled-coil domains 1	SPECC1	1,61
l(3)mbt-like 1 (Drosophila)	L3MBTL1	1,61
Transcript Identified by AceView, Entrez Gene ID(s) 23126	POGZ	1,61
Memczak2013 ALT_ACCEPTOR, ALT_DONOR, coding, INTERNAL, intronic best transcript NM_001040033	CD53	1,60
olfactory receptor, family 2, subfamily B, member 11	OR2B11	1,60
integrin alpha FG-GAP repeat containing 2	ITFG2	1,59
MANSC domain containing 1	MANSC1	1,59
HEAT repeat containing 4	HEATR4	1,59
Memczak2013 ALT_ACCEPTOR, ALT_DONOR, coding, INTERNAL, intronic best transcript NM_032792	ZBTB45	1,58
PR domain containing 1, with ZNF domain	PRDM1	1,58
Memczak2013 ALT_ACCEPTOR, ALT_DONOR, coding, INTERNAL, intronic best transcript NM_021937	EEFSEC	1,58
cancer/testis antigen family 45, member A7; cancer/testis antigen family 45, member A6	CT45A7; CT45A6	1,58

chymotrypsin-like	CTRL	1,58
Memczak2013 ANTISENSE, coding, INTERNAL, intronic best transcript NM_018023	YEATS2	1,57
anaplastic lymphoma receptor tyrosine kinase	ALK	1,57
adenylate kinase 8	AK8	1,57
chloride channel accessory 4	CLCA4	1,57
zinc finger family member 767, pseudogene	ZNF767P	1,57
purinergic receptor P2X, ligand gated ion channel, 5	P2RX5	1,56
Zhang2013 ALT_ACCEPTOR, ALT_DONOR, coding, INTERNAL, intronic best transcript NM_001127	AP1B1	1,56
Memczak2013 ALT_ACCEPTOR, ALT_DONOR, coding, INTERNAL, intronic best transcript NM_001031711	ERGIC1	1,56
tubulin, epsilon 1	TUBE1	1,56
Transcript Identified by AceView, Entrez Gene ID(s) 220972	MARCH8	1,56
olfactory receptor, family 51, subfamily E, member 1	OR51E1	1,56
interleukin 20	IL20	1,55
abhydrolase domain containing 16B	ABHD16B	1,55
SPANX family, member B1	SPANXB1	1,55
olfactory receptor, family 10, subfamily V, member 1	OR10V1	1,55
uncharacterized LOC100129940; Transcript Identified by AceView; novel transcript, antisense to HMGA2	LOC100129940; flanu; RP11-366L20.2	1,55
regulator of G-protein signaling 20	RGS20	1,55
leucine rich repeat containing 37, member A4, pseudogene	LRRC37A4P	1,55
interleukin 21	IL21	1,55
CSAG family, member 3	CSAG3	1,55
chemokine (C-C motif) ligand 7	CCL7	1,55
glycoprotein Ib (platelet), beta polypeptide; septin 5	GP1BB; SEPT5	1,54
Memczak2013 ALT_ACCEPTOR, ALT_DONOR, coding, INTERNAL, intronic best transcript NM_203487	PCDH9	1,54
threonine synthase-like 2	THNSL2	1,54
nucleosome assembly protein 1-like 5	NAP1L5	1,54
tetra-peptide repeat homeobox 1	TPRX1	1,54
matrix metallopeptidase 10	MMP10	1,54
tumor necrosis factor receptor superfamily, member 18	TNFRSF18	1,54
vimentin-type intermediate filament associated coiled-coil protein	VMAC	1,54
Transcript Identified by AceView, Entrez Gene ID(s) 8613	PPAP2B	1,53
chromosome 9 open reading frame 106	C9orf106	1,53
SH2B adaptor protein 3	SH2B3	1,53
membrane-spanning 4-domains, subfamily A, member 10	MS4A10	1,53
antizyme inhibitor 2	AZIN2	1,53
TBC1 domain family, member 27	TBC1D27	1,53
defensin, beta 128	DEFB128	1,53
coiled-coil domain containing 168	CCDC168	1,53

TAF1 RNA polymerase II, TATA box binding protein (TBP)-associated factor, 210kDa-like	TAF1L	1,53
gremlin 1, DAN family BMP antagonist [Source:HGNC Symbol;Acc:HGNC:2001]	GREM1	1,53
monoacylglycerol O-acyltransferase 1	MOGAT1	1,52
suppressor of cytokine signaling 2	SOCS2	1,52
nidogen 2 (osteonidogen)	NID2	1,52
tubulin tyrosine ligase-like family member 1	TTLL1	1,52
coiled-coil domain containing 73	CCDC73	1,52
glycerophosphocholine phosphodiesterase 1	GPCPD1	1,52
VPS11, CORVET/HOPS core subunit [Source:HGNC Symbol;Acc:HGNC:14583]	VPS11	1,52
Zhang2013 ALT_ACCEPTOR, ALT_DONOR, coding, INTERNAL, intronic best transcript NM_002953	RPS6KA1	1,52
transmembrane protein 171	TMEM171	1,52
protein phosphatase 1, regulatory subunit 13B	PPP1R13B	1,52
RAD9 checkpoint clamp component B	RAD9B	1,52
myosin light chain kinase 3	MYLK3	1,51
aldo-keto reductase family 1, member B10 (aldose reductase)	AKR1B10	1,51
NLR family, apoptosis inhibitory protein	NAIP	1,51
tubulin polymerization-promoting protein family member 2	TPPP2	1,51
NK6 homeobox 3	NKX6-3	1,51
DAZ interacting zinc finger protein 1-like	DZIP1L	1,51
E74-like factor 4 (ets domain transcription factor)	ELF4	1,51
desmocollin 3	DSC3	1,51
COX19 cytochrome c oxidase assembly factor	COX19	1,50
kelch-like family member 38	KLHL38	1,50
nephronectin	NPNT	1,50
solute carrier family 5 (sodium/inositol cotransporter), member 11	SLC5A11	1,50
serpin peptidase inhibitor, clade E (nexin, plasminogen activator inhibitor type 1), member 3	SERPINE3	1,50
Transcript Identified by AceView, Entrez Gene ID(s) 5747	PTK2	1,50
Transcript Identified by AceView, Entrez Gene ID(s) 79875	THSD4	1,50
Salzman2013 ANNOTATED, coding, OVEXON, UTR5 best transcript NM_001077664	URGCP	1,50
keratin associated protein 2-1	KRTAP2-1	1,50
coiled-coil domain containing 84	CCDC84	1,50
gamma-aminobutyric acid (GABA) A receptor, beta 2	GABRB2	1,50
ameloblastin	AMBN	1,50
actin-like 7B	ACTL7B	1,50
titin-cap	TCAP	1,50
chromosome 1 open reading frame 220	C1orf220	1,50
synuclein gamma	SNCG	1,49
V-set and transmembrane domain containing 4	VSTM4	1,49

solute carrier family 16, member 4	SLC16A4	1,49
kelch repeat and BTB (POZ) domain containing 12	KBTBD12	1,49
A kinase (PRKA) anchor protein 14	AKAP14	1,49
caspase recruitment domain family, member 16	CARD16	1,49
potassium channel, two pore domain subfamily K, member 17	KCNK17	1,49
Transcript Identified by AceView, Entrez Gene ID(s) 6845	VAMP7	1,49
FYVE and coiled-coil domain containing 1	FYCO1	1,49
stromal cell-derived factor 2-like 1	SDF2L1	1,49
Memczak2013 ANTISENSE, coding, INTERNAL, intronic best transcript NM_139057	ADAMTS17	1,49
coagulation factor VIII, procoagulant component	F8	1,49
Memczak2013 ANTISENSE, coding, INTERNAL, intronic best transcript NM_018150	RNF220	1,49
uncharacterized protein MGC27345	MGC27345	1,49
zinc finger protein 501 [Source:HGNC Symbol;Acc:HGNC:23717]	ZNF501	1,49
radial spoke head 4 homolog A (Chlamydomonas)	RSPH4A	1,48
DDB1 and CUL4 associated factor 8-like 2	DCAF8L2	1,48
torsin family 3, member A	TOR3A	1,48
WD repeat domain 63	WDR63	1,48
histatin 1	HTN1	1,48
F-box and leucine-rich repeat protein 2	FBXL2	1,48
chromosome 12 open reading frame 71	C12orf71	1,48
olfactory receptor, family 1, subfamily F, member 1	OR1F1	1,48
PRAME family member 18	PRAMEF18	1,48
long intergenic non-protein coding RNA 482	LINC00482	1,48
zinc finger protein 519	ZNF519	1,48
RAD52 motif containing 1	RDM1	1,48
Transcript Identified by AceView, Entrez Gene ID(s) 10896	OCLM	1,48
heparan sulfate 6-O-sulfotransferase 2	HS6ST2	1,48
MAGE family member B16	MAGEB16	1,48
proteasome 26S subunit, ATPase 4 [Source:HGNC Symbol;Acc:HGNC:9551]	PSMC4	1,48
solute carrier family 17 (organic anion transporter), member 3; histone cluster 1, H2a, pseudogene 2	SLC17A3; HIST1H2APS2	1,47
Transcript Identified by AceView, Entrez Gene ID(s) 1174	AP1S1	1,47
mesenteric estrogen-dependent adipogenesis	MEDAG	1,47
ubiquitin conjugating enzyme E2E 2	UBE2E2	1,47
STAM binding protein-like 1	STAMBPL1	1,47
chitinase 3-like 2	CHI3L2	1,47
SH3-domain binding protein 2	SH3BP2	1,47
archaelysin family metallopeptidase 1	AMZ1	1,47
oxoglutarate (alpha-ketoglutarate) receptor 1	OXGR1	1,47
zinc finger protein 718	ZNF718	1,47
fatty acyl-CoA reductase 2 pseudogene 1	FAR2P1	1,47

erythroblast membrane-associated protein (Scianna blood group)	ERMAP	1,47
hemopexin	HPX	1,47
SFI1 centrin binding protein	SFI1	1,47
baculoviral IAP repeat containing 3	BIRC3	1,47
sialic acid binding Ig-like lectin 16 (gene/pseudogene)	SIGLEC16	1,47
Transcript Identified by AceView, Entrez Gene ID(s) 64478	CSMD1	1,47
threonine synthase-like 1	THNSL1	1,46
keratin associated protein 2-2	KRTAP2-2	1,46
alkaline phosphatase, placental like 2	ALPPL2	1,46
breast carcinoma amplified sequence 3	BCAS3	1,46
mitochondrial amidoxime reducing component 1	MARC1	1,46
myosin, heavy chain 11, smooth muscle	MYH11	1,46
Memczak2013 ANTISENSE, coding, INTERNAL, intronic best transcript NM_006947	SRP72	1,46
heat shock 27kDa protein 2; chromosome 11 open reading frame 52; HSPB2-C11orf52 readthrough (NMD candidate)	HSPB2; C11orf52; HSPB2-C11orf52	1,46
serpin peptidase inhibitor, clade I (pancpin), member 2	SERPINI2	1,46
submaxillary gland androgen regulated protein 3A	SMR3A	1,46
solute carrier family 16, member 14	SLC16A14	1,46
spermatogenesis associated 1	SPATA1	1,46
aprataxin	APTX	1,46
MAGE family member A2	MAGEA2	1,46
zinc finger protein 780A	ZNF780A	1,46
reticulon 4 interacting protein 1	RTN4IP1	1,46
small integral membrane protein 11A	SMIM11A	1,46
spermatogenesis associated 13; C1q and tumor necrosis factor related protein 9	SPATA13; C1QTNF9	1,46
polypeptide N-acetylgalactosaminyltransferase-like 5	GALNTL5	1,45
gremlin 1, DAN family BMP antagonist	GREM1	1,45
cone-rod homeobox	CRX	1,45
indoleamine 2,3-dioxygenase 1	IDO1	1,45
calcium release activated channel regulator 2A	CRACR2A	1,45
proline-rich protein HaeIII subfamily 1; taste receptor, type 2, member 14; proline rich 4 (lacrimal)	PRH1; TAS2R14; PRR4	1,45
mitogen-activated protein kinase 8 interacting protein 3	MAPK8IP3	1,45
Jeck2013 ALT_ACCEPTOR, ALT_DONOR, coding, INTERNAL, intronic best transcript NM_000314; novel transcript, sense intronic to PTEN	RP11-380G5.2; PTEN	1,45
chromosome 3 open reading frame 67	C3orf67	1,45
transmembrane protein 14E, pseudogene	TMEM14EP	1,45
methyltransferase like 18	METTL18	1,45

Zhang2013 ALT_ACCEPTOR, ALT_DONOR, coding, INTERNAL, intronic best transcript NM_052909	PLEKHG4B	1,45
hydroxymethylbilane synthase	HMBS	1,45
B and T lymphocyte associated	BTLA	1,45
ADAM metallopeptidase with thrombospondin type 1 motif 1	ADAMTS1	1,45
small integral membrane protein 9	SMIM9	1,44
fibroblast growth factor 9	FGF9	1,44
retinal G protein coupled receptor	RGR	1,44
chemokine (C-C motif) ligand 1	CCL1	1,44
leucine rich repeat containing 32	LRRC32	1,44
aldehyde dehydrogenase 1 family, member L2	ALDH1L2	1,44
major histocompatibility complex, class II, DM alpha	HLA-DMA	1,44
saitohin	STH	1,44
THAP domain containing, apoptosis associated protein 3	THAP3	1,44
granzyme A	GZMA	1,44
deleted in azoospermia 2; deleted in azoospermia 4	DAZ2; DAZ4	1,44
interferon, alpha 13	IFNA13	1,44
Memczak2013 ALT_ACCEPTOR, ALT_DONOR, coding, INTERNAL, intronic best transcript NM_000332	ATXN1	1,44
coiled-coil domain containing 74A	CCDC74A	1,44
calcium channel, voltage-dependent, gamma subunit 6	CACNG6	1,44
testis-specific serine kinase 2	TSSK2	1,44
UDP-Gal:betaGlcNAc beta 1,4- galactosyltransferase, polypeptide 4	B4GALT4	1,44
trafficking protein particle complex 4	TRAPPC4	1,44
zinc finger protein 605	ZNF605	1,44
inhibitor of CDK, cyclin A1 interacting protein 1	INCA1	1,44
Transcript Identified by AceView, Entrez Gene ID(s) 134145	FAM173B	1,44
Transcript Identified by AceView, Entrez Gene ID(s) 10580	SORBS1	1,44
coiled-coil domain containing 173	CCDC173	1,44
transmembrane protein 67	TMEM67	1,44
Memczak2013 ANTISENSE, coding, INTERNAL, intronic best transcript NM_012399	PITPNB	1,44
Jeck2013 ALT_ACCEPTOR, ALT_DONOR, coding, INTERNAL, intronic best transcript NM_001145418	TTC28	1,43
long intergenic non-protein coding RNA 1599	LINC01599	1,43
transmembrane protein 136	TMEM136	1,43
cat eye syndrome chromosome region, candidate 5	CECR5	1,43
proline-rich transmembrane protein 2	PRRT2	1,43
Transcript Identified by AceView, Entrez Gene ID(s) 285596	FAM153A	1,43
transmembrane protein 102	TMEM102	1,43
small integral membrane protein 5	SMIM5	1,43
polypeptide N-acetylgalactosaminyltransferase 10	GALNT10	1,43
glutaredoxin, cysteine rich 2	GRXCR2	1,43
ribosomal RNA processing 36	RRP36	1,43

centrosomal protein 83kDa	CEP83	1,43
FERM and PDZ domain containing 2B, pseudogene; FERM and PDZ domain containing 2	FRMPD2B; FRMPD2	1,43
calcium channel, voltage-dependent, gamma subunit 3	CACNG3	1,43
ER membrane protein complex subunit 1	EMC1	1,43
dual specificity tyrosine-(Y)-phosphorylation regulated kinase 1B	DYRK1B	1,43
PDZ domain containing 9	PDZD9	1,43
transmembrane 4 L six family member 19	TM4SF19	1,43
taste receptor, type 2, member 10	TAS2R10	1,43
R3H domain and coiled-coil containing 1-like	R3HCC1L	1,43
coiled-coil domain containing 170	CCDC170	1,43
G antigen 10	GAGE10	1,43
family with sequence similarity 220, member A	FAM220A	1,43
CREB regulated transcription coactivator 1	CRTC1	1,43
PRELI domain containing 3A	PRELID3A	1,43
transcription factor 15 (basic helix-loop-helix)	TCF15	1,43
DNA damage inducible transcript 4	DDIT4	1,43
small integral membrane protein 11A	SMIM11A	1,43
RAB34, member RAS oncogene family	RAB34	1,43
DMRT-like family C1B; DMRT-like family C1	DMRTC1B; DMRTC1	1,43
signal peptide peptidase like 2C	SPPL2C	1,43
heat shock transcription factor, Y-linked 2	HSFY2	1,43
Memczak2013 ALT_DONOR, coding, INTERNAL, intronic best transcript NM_001080437	SNED1	1,43
C-type lectin domain family 4, member D	CLEC4D	1,43
carbohydrate (N-acetylglucosamine-6-O) sulfotransferase 2	CHST2	1,43
contactin 1	CNTN1	1,43
autophagy related 16-like 1	ATG16L1	1,42
zinc finger and BTB domain containing 17	ZBTB17	1,42
maltase-glucoamylase 2 (putative)	MGAM2	1,42
ATPase, Cu++ transporting, alpha polypeptide	ATP7A	1,42
gamma-aminobutyric acid (GABA) A receptor, alpha 5	GABRA5	1,42
zinc finger protein 618	ZNF618	1,42
cytochrome P450, family 1, subfamily B, polypeptide 1	CYP1B1	1,42
family with sequence similarity 133, member A	FAM133A	1,42
enhancer of zeste 1 polycomb repressive complex 2 subunit	EZH1	1,42
zinc finger protein 30	ZNF30	1,42
muscular LMNA-interacting protein	MLIP	1,42
WAP four-disulfide core domain 11	WFDC11	1,42
coiled-coil domain containing 84	CCDC84	1,42
lactate dehydrogenase A-like 6B	LDHAL6B	1,42
cytohesin 4	CYTH4	1,42
Transcript Identified by AceView, Entrez Gene ID(s) 116840	CNTROB	1,42
CD58 molecule	CD58	1,42

interleukin 11 receptor, alpha	IL11RA	1,42
T-box 18	TBX18	1,42
lipase, gastric	LIPF	1,42
KIAA1755	KIAA1755	1,41
plexin domain containing 1	PLXDC1	1,41
cytochrome P450, family 2, subfamily A, polypeptide 7	CYP2A7	1,41
serum amyloid A1	SAA1	1,41
chromosome 6 open reading frame 1	C6orf1	1,41
calcium channel, voltage-dependent, N type, alpha 1B subunit	CACNA1B	1,41
HCK proto-oncogene, Src family tyrosine kinase	HCK	1,41
phosphoinositide-3-kinase, regulatory subunit 3 (gamma)	PIK3R3	1,41
interleukin 13	IL13	1,41
acyl-CoA synthetase medium-chain family member 3	ACSM3	1,41
Memczak2013 ANTISENSE, CDS, coding, INTERNAL, intronic best transcript NM_001178056	PARP8	1,41
sema domain, immunoglobulin domain (Ig), transmembrane domain (TM) and short cytoplasmic domain, (semaphorin) 4A	SEMA4A	1,41
BarH-like homeobox 2	BARHL2	1,41
transmembrane protein 25	TMEM25	1,41
theg spermatid protein-like	THEGL	1,41
lectin, galactoside-binding, soluble, 7B	LGALS7B	1,41
NADH dehydrogenase (ubiquinone) 1 alpha subcomplex, 10, 42kDa	NDUFA10	1,41
family with sequence similarity 219, member A	FAM219A	1,41
Memczak2013 ALT_ACCEPTOR, ALT_DONOR, coding, INTERNAL, intronic best transcript NM_212471	PRKAR1A	1,41
helicase (DNA) B	HELB	1,41
Transcript Identified by AceView, Entrez Gene ID(s) 50804	MYEF2	1,40
Memczak2013 ANTISENSE, coding, INTERNAL, UTR3 best transcript NM_002727	SRGN	1,40
SLP adaptor and CSK interacting membrane protein	SCIMP	1,40
ankyrin repeat domain 16	ANKRD16	1,40
glutathione S-transferase alpha 1	GSTA1	1,40
neuromedin U	NMU	1,40
cancer/testis antigen 83	CT83	1,40
SET domain containing (lysine methyltransferase) 7	SETD7	1,40
leucine-rich repeats and guanylate kinase domain containing	LRGUK	1,40
IGF like family member 4	IGFL4	1,40
lymphotoxin beta receptor (TNFR superfamily, member 3)	LTBR	1,40
palladin, cytoskeletal associated protein	PALLD	1,40
chromosome 7 open reading frame 69	C7orf69	1,40
ankyrin repeat domain 13B	ANKRD13B	1,40
zinc finger protein 329	ZNF329	1,40
Transcript Identified by AceView, Entrez Gene ID(s) 10082	GPC6	1,40
5-nucleotidase domain containing 4	NT5DC4	1,40

ATPase, Na+/K+ transporting, alpha 2 polypeptide	ATP1A2	1,40
ras homolog family member J	RHOJ	1,40
dual specificity tyrosine-(Y)-phosphorylation regulated kinase 1B	DYRK1B	1,40
polycystic kidney and hepatic disease 1 (autosomal recessive)	PKHD1	1,40
Memczak2013 ANTISENSE, coding, INTERNAL, intronic best transcript NM_006990	WASF2	1,40
IQ motif containing D	IQCD	1,40
tripartite motif containing 69	TRIM69	1,40
diacylglycerol kinase gamma	DGKG	1,40
protocadherin beta 2	PCDHB2	1,40
small proline-rich protein 2A	SPRR2A	1,40
RNA binding region (RNP1, RRM) containing 3	RNPC3	1,40
phospholipid phosphatase 4	PLPP4	1,40
thrombospondin 3	THBS3	1,40
cytoglobin	CYGB	1,40
vesicle amine transport 1-like	VAT1L	1,40
surfeit 2 [Source:HGNC Symbol;Acc:HGNC:11475]	SURF2	1,39
activating transcription factor 3	ATF3	1,39
family with sequence similarity 92, member B	FAM92B	1,39
LIM and calponin homology domains 1	LIMCH1	1,39
neurotrophic tyrosine kinase, receptor, type 2	NTRK2	1,39
Transcript Identified by AceView, Entrez Gene ID(s) 51105	PHF20L1	1,39
zinc finger protein 624	ZNF624	1,39
proprotein convertase subtilisin/kexin type 4	PCSK4	1,39
RIB43A domain with coiled-coils 2	RIBC2	1,39
CUGBP, Elav-like family member 3	CELF3	1,39
keratin associated protein 19-4	KRTAP19-4	1,39
protease, serine, 12 (neurotrypsin, motopsin)	PRSS12	1,39
cementum protein 1	CEMP1	1,39
homeobox B3; homeobox B4; microRNA 10a	HOXB3; HOXB4; MIR10A	1,39
zinc finger and SCAN domain containing 2	ZSCAN2	1,39
SET domain containing (lysine methyltransferase) 7	SETD7	1,39
zinc finger protein 230	ZNF230	1,39
cystathionine gamma-lyase	CTH	1,39
zinc finger protein 423	ZNF423	1,39
zinc finger protein 583	ZNF583	1,39
coiled-coil domain containing 181	CCDC181	1,39
POTE ankyrin domain family, member F	POTEF	1,39
jumonji domain containing 8	JMJD8	1,39
integrin alpha X	ITGAX	1,39
ATP binding cassette subfamily A member 2	ABCA2	1,39
small nuclear ribonucleoprotein D2 pseudogene 2	SNRPD2P2	1,39
phosphodiesterase 3B, cGMP-inhibited	PDE3B	1,39

tetratricopeptide repeat domain 6	TTC6	1,39
TRAF3 interacting protein 3	TRAF3IP3	1,39
neuroblastoma breakpoint family, member 3	NBPF3	1,39
family with sequence similarity 197, Y-linked, member 1	FAM197Y1	1,39
olfactory receptor, family 4, subfamily D, member 1	OR4D1	1,39
FSHD region gene 2	FRG2	1,39
Transcript Identified by AceView, Entrez Gene ID(s) 93663; 253582; Jeck2013 ALT_ACCEPTOR, ALT_DONOR, downstream_end, ncRNA, OVEXON, upstream_start best transcript TCONS_l2_00025470; Jeck2013 ALT_ACCEPTOR, ncRNA, OVEXON, upstream_start best transcript TCONS_l2_00024911; Salzman2013 ANTISENSE, CDS, coding, downstream_end, intronic, OVCODE, OVEXON, UTR3 best transcript NM_000426; Salzman2013 ANNOTATED, INTERNAL, ncRNA, OVEXON best transcript TCONS_l2_00025470; putative novel transcript; novel transcript	C6orf191andARHGAP18; RP1-69D17.4; RP1-69D17.3; TCONS_l2_00025470; TCONS_l2_00024911; LAMA2	1,39
coiled-coil domain containing 33	CCDC33	1,39
G protein-coupled receptor 162	GPR162	1,38
zinc finger with KRAB and SCAN domains 7	ZKSCAN7	1,38
zinc finger protein 236	ZNF236	1,38
ras homolog family member Q	RHOQ	1,38
Fc fragment of IgG, low affinity IIIb, receptor (CD16b)	FCGR3B	1,38
carbonic anhydrase XIII	CA13	1,38
caspase recruitment domain family, member 11	CARD11	1,38
chromosome 7 open reading frame 25	C7orf25	1,38
protease, serine, 33	PRSS33	1,38
Transcript Identified by AceView, Entrez Gene ID(s) 8997	KALRN	1,38
SEC14-like lipid binding 6	SEC14L6	1,38
N-myc (and STAT) interactor	NMI	1,38
ZNF252P antisense RNA 1	ZNF252P-AS1	1,38
Transcript Identified by AceView, Entrez Gene ID(s) 8148	TAF15	1,38
Transcript Identified by AceView, Entrez Gene ID(s) 6502	SKP2	1,38
SUMO-interacting motifs containing 1	SIMC1	1,38
cancer/testis antigen family 45, member A6; cancer/testis antigen family 45, member A7	CT45A6; CT45A7	1,38
HCLS1 associated protein X-1	HAX1	1,38
tetratricopeptide repeat domain 30A	TTC30A	1,38
sestrin 2	SESN2	1,38
heat shock 70kDa protein 4-like	HSPA4L	1,38
diacylglycerol kinase, zeta	DGKZ	1,38
chromosome 19 open reading frame 33	C19orf33	1,38
EPH receptor A7	EPHA7	1,38
eva-1 homolog C (C. elegans)	EVA1C	1,38
proline rich 21	PRR21	1,38
CD274 molecule	CD274	1,38

microfibrillar associated protein 4	MFAP4	1,38
defensin, beta 118	DEFB118	1,38
protein tyrosine phosphatase, non-receptor type 21	PTPN21	1,38
KIAA1328	KIAA1328	1,38
trafficking protein particle complex 4	TRAPPC4	1,38
growth arrest and DNA-damage-inducible, gamma interacting protein 1	GADD45GIP1	1,38
DEAH (Asp-Glu-Ala-His) box polypeptide 36	DHX36	1,38
zinc finger protein 518B	ZNF518B	1,38
Jeck2013 ALT_ACCEPTOR, ALT_DONOR, coding, INTERNAL, intronic best transcript NM_004249	RAB28	1,38
ATP binding cassette subfamily A member 3	ABCA3	1,38
regulator of G-protein signaling 22	RGS22	1,38
Ras association (RalGDS/AF-6) domain family member 5	RASSF5	1,38
keratin 9, type I	KRT9	1,38
Memczak2013 ANTISENSE, CDS, coding, INTERNAL best transcript NM_006623	PHGDH	1,38
WNT1 inducible signaling pathway protein 3	WISP3	1,38
protocadherin beta 6	PCDHB6	1,37
EF-hand domain family, member B	EFHB	1,37
proline rich 4 (lacrimal)	PRR4	1,37
keratin associated protein 21-2	KRTAP21-2	1,37
Transcript Identified by AceView, Entrez Gene ID(s) 4179	CD46	1,37
ribosome biogenesis regulator homolog	RRS1	1,37
P2RX5-TAX1BP3 readthrough (NMD candidate)	P2RX5-TAX1BP3	1,37
ring finger protein 113B	RNF113B	1,37
ankyrin repeat domain 34C	ANKRD34C	1,37
ALX homeobox 3	ALX3	1,37
UL16 binding protein 1	ULBP1	1,37
heat shock protein, alpha-crystallin-related, B6	HSPB6	1,37
spermatogenesis associated 6-like	SPATA6L	1,37
protein phosphatase 1, regulatory subunit 3E	PPP1R3E	1,37
NME/NM23 family member 8	NME8	1,37
beta-1,3-N-acetylgalactosaminyltransferase 1 (globoside blood group)	B3GALNT1	1,37
uncharacterized LOC284365; uncharacterized LOC284365 [Source:EntrezGene;Acc:284365]; novel transcript	MGC45922; CTD-2568A17.5	1,37
RAB39B, member RAS oncogene family	RAB39B	1,37
annexin A9	ANXA9	1,37
ankyrin repeat and SOCS box containing 5	ASB5	1,37
tryptophanyl-tRNA synthetase	WARS	1,37
wingless-type MMTV integration site family, member 2B	WNT2B	1,37
retinitis pigmentosa GTPase regulator	RPGR	1,37
Bardet-Biedl syndrome 1	BBS1	1,37

Memczak2013 ALT_ACCEPTOR, ALT_DONOR, coding, INTERNAL, intronic best transcript NM_001204883	RAB43	1,37
calcium channel, voltage-dependent, P/Q type, alpha 1A subunit	CACNA1A	1,37
Transcript Identified by AceView, Entrez Gene ID(s) 56890	MDM1	1,37
3-hydroxyacyl-CoA dehydratase 1	HACD1	1,37
Sp6 transcription factor	SP6	1,37
C-type lectin domain family 4, member M	CLEC4M	1,37
Na+/K+ transporting ATPase interacting 3	NKAIN3	1,37
endoplasmic reticulum protein 27	ERP27	1,37
septin 1	sept-01	1,37
formin like 1	FMNL1	1,37
opsin 5	OPN5	1,37
B-cell CLL/lymphoma 11A (zinc finger protein)	BCL11A	1,37
aminoadipate aminotransferase	AADAT	1,37
long intergenic non-protein coding RNA 266-1	LINC00266-1	1,37
neurofilament, heavy polypeptide	NEFH	1,37
N-acetylglutamate synthase	NAGS	1,37
RAB39A, member RAS oncogene family	RAB39A	1,37
Rho GTPase activating protein 4	ARHGAP4	1,37
TraB domain containing 2A	TRABD2A	1,37
PBX/knotted 1 homeobox 2	PKNOX2	1,37
GULP, engulfment adaptor PTB domain containing 1	GULP1	1,37
interleukin 2 receptor, gamma	IL2RG	1,37
fatty acid binding protein 2, intestinal	FABP2	1,37
phosphotyrosine interaction domain containing 1	PID1	1,37
RAB24, member RAS oncogene family	RAB24	1,37
DEAD (Asp-Glu-Ala-Asp) box helicase 25	DDX25	1,36
late cornified envelope 3D	LCE3D	1,36
potassium channel, two pore domain subfamily K, member 16	KCNK16	1,36
keratin associated protein 27-1	KRTAP27-1	1,36
transmembrane protein 173	TMEM173	1,36
collectin sub-family member 12	COLEC12	1,36
basic helix-loop-helix and HMG box domain containing 1	BHMG1	1,36
5-oxoprolinase (ATP-hydrolysing)	OPLAH	1,36
peptidase inhibitor 16	PI16	1,36
Zhang2013 ALT_ACCEPTOR, ALT_DONOR, coding, INTERNAL, intronic best transcript NM_001164446	C6orf132	1,36
solute carrier family 27 (fatty acid transporter), member 5	SLC27A5	1,36
oxysterol binding protein 2	OSBP2	1,36
tyrosine hydroxylase	TH	1,36
glycerol-3-phosphate acyltransferase 3	GPAT3	1,36
transmembrane protein 88	TMEM88	1,36
major facilitator superfamily domain containing 4	MFSD4	1,36
inhibitor of growth family member 2	ING2	1,36

POTE ankyrin domain family, member M; POTE ankyrin domain family, member G	POTEM; POTEG	1,36
maelstrom spermatogenic transposon silencer	MAEL	1,36
Transcript Identified by AceView, Entrez Gene ID(s) 339500	ZNF678	1,36
Charcot-Leyden crystal protein pseudogene	LGALS17A	1,36
neurexophilin 2	NXPH2	1,36
Memczak2013 ALT_ACCEPTOR, ALT_DONOR, coding, INTERNAL, intronic best transcript NM_001098512	PRKG1	1,36
testis-specific serine kinase 3	TSSK3	1,36
MLX interacting protein	MLXIP	1,36
corticotropin releasing hormone receptor 2	CRHR2	1,36
interleukin 6	IL6	1,36
epididymal protein 3A	EDDM3A	1,36
zinc finger protein 709	ZNF709	1,36
kelch repeat and BTB (POZ) domain containing 8	KBTBD8	1,36
spire-type actin nucleation factor 2	SPIRE2	1,36
kelch repeat and BTB (POZ) domain containing 3	KBTBD3	1,36
Transcript Identified by AceView, Entrez Gene ID(s) 22874	PLEKHA6	1,36
NIMA-related kinase 11	NEK11	1,36
cadherin 18, type 2	CDH18	1,36
Transcript Identified by AceView, Entrez Gene ID(s) 11193	WBP4	1,36
leucine rich repeat containing 4C	LRRC4C	1,36
Transcript Identified by AceView, Entrez Gene ID(s) 7227	TRPS1	1,36
actin-related protein T1	ACTRT1	1,36
Transcript Identified by AceView, Entrez Gene ID(s) 23358	USP24	1,36
ISL LIM homeobox 2	ISL2	1,36
dipeptidyl-peptidase 10 (inactive)	DPP10	1,36
family with sequence similarity 124 member B	FAM124B	1,36
deltex 3, E3 ubiquitin ligase	DTX3	1,36
RAB3B, member RAS oncogene family	RAB3B	1,36
olfactory receptor, family 10, subfamily Z, member 1	OR10Z1	1,36
ZBED6 C-terminal like	ZBED6CL	1,36
MOK protein kinase	MOK	1,36
cytochrome P450, family 11, subfamily A, polypeptide 1	CYP11A1	1,36
killer cell lectin-like receptor subfamily B, member 1	KLRB1	1,36
testis specific 10	TSGA10	1,36
NACHT and WD repeat domain containing 2	NWD2	1,36
alpha kinase 2	ALPK2	1,36
zinc finger protein 251	ZNF251	1,36
hect domain and RLD 2 pseudogene 3	HERC2P3	1,36
Rho GTPase activating protein 21	ARHGAP21	1,36
small vasohibin binding protein	SVBP	1,36
apoptosis, caspase activation inhibitor	AVEN	1,36
ubiquitin-conjugating enzyme E2F (putative)	UBE2F	1,35
X-ray radiation resistance associated 1	XRRA1	1,35

cystathionine-beta-synthase	CBS	1,35
solute carrier family 16 (monocarboxylate transporter), member 1	SLC16A1	1,35
olfactory receptor, family 2, subfamily AK, member 2	OR2AK2	1,35
mediator complex subunit 30	MED30	1,35
chromosome 20 open reading frame 96	C20orf96	1,35
synaptotagmin XII	SYT12	1,35
K(lysine) acetyltransferase 6B	KAT6B	1,35
SRSF protein kinase 2	SRPK2	1,35
CD3e molecule, epsilon (CD3-TCR complex)	CD3E	1,35
guanine nucleotide binding protein (G protein), gamma 8	GNG8	1,35
T-cell leukemia translocation altered	TCTA	1,35
S100 calcium binding protein A8	S100A8	1,35
X antigen family, member 5	XAGE5	1,35
calcium binding protein 5	CABP5	1,35
chromosome 5 open reading frame 58	C5orf58	1,35
deoxyribonuclease II beta	DNASE2B	1,35
chemokine (C motif) ligand 1	XCL1	1,35
heterogeneous nuclear ribonucleoprotein A1-like 2	HNRNPA1L2	1,35
TELO2 interacting protein 2	TTI2	1,35
cullin 9	CUL9	1,35
PRP40 homolog, pre-mRNA processing factor B	PRPF40B	1,35
KIAA1109	KIAA1109	1,35
atlastin GTPase 1	ATL1	1,35
KIAA0753	KIAA0753	1,35
plexin domain containing 2	PLXDC2	1,35
RAS p21 protein activator 2	RASA2	1,35
family with sequence similarity 153, member A	FAM153A	1,35
small proline-rich protein 1A	SPRR1A	1,35
periplakin	PPL	1,35
synaptogyrin 3	SYNGR3	1,35
Zhang2013 ALT_ACCEPTOR, ALT_DONOR, coding, INTERNAL, intronic best transcript NM_004110	FDXR	1,35
gremlin 2, DAN family BMP antagonist	GREM2	1,35
elongator acetyltransferase complex subunit 4	ELP4	1,35
transforming, acidic coiled-coil containing protein 2	TACC2	1,35
unc-51 like kinase 4	ULK4	1,35
solute carrier family 4 (anion exchanger), member 3	SLC4A3	1,35
taste receptor, type 2, member 39	TAS2R39	1,35
transgelin 3	TAGLN3	1,35
taste receptor, type 2, member 7	TAS2R7	1,35
zinc finger and BTB domain containing 20; microRNA 568	ZBTB20; MIR568	1,35
collagen, type VI, alpha 1	COL6A1	1,35
zinc finger protein 563	ZNF563	1,35

RIB43A domain with coiled-coils 1	RIBC1	1,35
HRAS-like suppressor 2	HRASLS2	1,35
sodium channel and clathrin linker 1	SCLT1	1,35
AF4/FMR2 family, member 2	AFF2	1,35
killer cell immunoglobulin-like receptor, two domains, short cytoplasmic tail, 5; killer cell immunoglobulin-like receptor, three domains, long cytoplasmic tail, 2; killer cell immunoglobulin-like receptor, two domains, long cytoplasmic tail, 1; killer cell immunoglobulin-like receptor, three domains, long cytoplasmic tail, 3; killer cell immunoglobulin-like receptor, three domains, long cytoplasmic tail, 1; killer cell immunoglobulin-like receptor, two domains, short cytoplasmic tail, 4; killer cell immunoglobulin-like receptor, two domains, long cytoplasmic tail, 3; killer cell immunoglobulin-like receptor, three domains, short cytoplasmic tail, 1; killer cell immunoglobulin-like receptor, two domains, short cytoplasmic tail, 2; killer cell immunoglobulin-like receptor, two domains, pseudogene 1; killer cell immunoglobulin-like receptor, three domains, pseudogene 1	KIR2DS5; KIR3DL2; KIR2DL1; KIR3DL3; KIR3DL1; KIR2DS4; KIR2DL3; KIR3DS1; KIR2DS2; KIR2DP1; KIR3DP1	1,35
zinc finger with KRAB and SCAN domains 7	ZKSCAN7	1,35
slit guidance ligand 1; ARHGAP19-SLIT1 readthrough (NMD candidate)	SLIT1; ARHGAP19-SLIT1	1,35
nucleotide-binding oligomerization domain containing 1	NOD1	1,35
potassium channel, voltage gated eag related subfamily H, member 3	KCNH3	1,35
GRAM domain containing 1C	GRAMD1C	1,35
Down syndrome critical region 10 (non-protein coding)	DSCR10	1,35
metallo-beta-lactamase domain containing 2	MBLAC2	1,35
Transcript Identified by AceView, Entrez Gene ID(s) 23527; ACAP2 intronic transcript 1 (non-protein coding) [Source:HGNC Symbol;Acc:HGNC:41426]; novel transcript; ACAP2 intronic transcript 1 [Source:HGNC Symbol;Acc:HGNC:41426]	ACAP2; ACAP2-IT1; AC090018.3	1,35
sperm acrosome associated 5B	SPACA5B	1,35
zona pellucida glycoprotein 3 (sperm receptor)	ZP3	1,34
Zhang2013 ALT_ACCEPTOR, ALT_DONOR, coding, INTERNAL, intronic best transcript NM_001122823	GTF3C5	1,34
leucine rich repeat containing 37, member A2	LRRC37A2	1,34
olfactory receptor, family 4, subfamily N, member 5	OR4N5	1,34
4-aminobutyrate aminotransferase	ABAT	1,34
olfactory receptor, family 10, subfamily P, member 1	OR10P1	1,34
phospholipase C, beta 2	PLCB2	1,34
KIAA0040	KIAA0040	1,34
NLR family, CARD domain containing 3	NLRC3	1,34
calmodulin-like 3	CALML3	1,34
ArfGAP with dual PH domains 1	ADAP1	1,34

killer cell lectin-like receptor subfamily G, member 2	KLRG2	1,34
NME/NM23 family member 9	NME9	1,34
transmembrane protein 244	TMEM244	1,34
olfactory receptor, family 51, subfamily Q, member 1 (gene/pseudogene)	OR51Q1	1,34
myocardin	MYOCD	1,34
KRIT1, ankyrin repeat containing	KRIT1	1,34
PHD finger protein 11	PHF11	1,34
5-nucleotidase, cytosolic IIIA	NT5C3A	1,34
deltex 3 like, E3 ubiquitin ligase	DTX3L	1,34
serine hydrolase-like 2	SERHL2	1,34
nuclear protein 1, transcriptional regulator	NUPR1	1,34
fibulin 5	FBLN5	1,34
olfactory receptor, family 2, subfamily M, member 3	OR2M3	1,34
Memczak2013 ANTISENSE, CDS, coding, INTERNAL, intronic best transcript NM_004579	MAP4K2	1,34
leucine rich repeat transmembrane neuronal 4	LRRTM4	1,34
zinc finger protein 669	ZNF669	1,34
MAD1 mitotic arrest deficient-like 1 (yeast)	MAD1L1	1,34
myosin X	MYO10	1,34
mutS homolog 3	MSH3	1,34
LIM domain containing 2	LIMD2	1,34
nuclear apoptosis inducing factor 1	NAIF1	1,34
praja ring finger 1, E3 ubiquitin protein ligase	PJA1	1,34
TAM41 mitochondrial translocator assembly and maintenance homolog	TAMM41	1,34
RNA binding protein, fox-1 homolog (C. elegans) 1	RBFOX1	1,34
aspartic peptidase, retroviral-like 1; PCBP1 antisense RNA 1	ASPRV1; PCBP1-AS1	1,34
golgin A6 family, member C	GOLGA6C	1,34
lysosomal protein transmembrane 5	LAPTM5	1,34
glucosaminyl (N-acetyl) transferase 1, core 2	GCNT1	1,34
deleted in azoospermia 2; deleted in azoospermia 3	DAZ2; DAZ3	1,34
carbonic anhydrase VB, mitochondrial	CA5B	1,34
olfactory receptor, family 6, subfamily C, member 1	OR6C1	1,34
N-deacetylase/N-sulfotransferase (heparan glucosaminyl) 2	NDST2	1,34
keratin associated protein 4-12	KRTAP4-12	1,34
protease, serine, 48	PRSS48	1,34
solute carrier family 28 (concentrative nucleoside transporter), member 2	SLC28A2	1,34
WBP2 N-terminal like	WBP2NL	1,34
chemokine (C-C motif) receptor-like 2	CCRL2	1,34
ATPase, H+ transporting, lysosomal 13kDa, V1 subunit G3	ATP6V1G3	1,34
adenosine A2a receptor	ADORA2A	1,34
olfactory receptor, family 52, subfamily B, member 2	OR52B2	1,34

tripartite motif containing 74	TRIM74	1,34
KN motif and ankyrin repeat domains 3	KANK3	1,34
syntrophin, alpha 1	SNTA1	1,34
claudin 2	CLDN2	1,34
biliverdin reductase B	BLVRB	1,34
syntaxin 1A (brain)	STX1A	1,34
solute carrier family 7 (anionic amino acid transporter light chain, xc- system), member 11	SLC7A11	1,34
pregnancy specific beta-1-glycoprotein 11	PSG11	1,34
Transcript Identified by AceView; Zhang2013 ALT_ACCEPTOR, ALT_DONOR, coding, INTERNAL, intronic best transcript NM_006548	dorsnarby; IGF2BP2	1,34
phosphoenolpyruvate carboxykinase 2 (mitochondrial)	PCK2	1,34
melanoma associated antigen (mutated) 1-like 1	MUM1L1	1,34
polypeptide N-acetylgalactosaminyltransferase 11	GALNT11	1,33
ankyrin repeat and SOCS box containing 7	ASB7	1,33
tumor protein p73	TP73	1,33
WD repeat domain 31	WDR31	1,33
SRY box 2	SOX2	1,33
klotho	KL	1,33
phosphatidylinositol transfer protein, membrane-associated 2	PITPNM2	1,33
A kinase (PRKA) anchor protein 3	AKAP3	1,33
Rho/Rac guanine nucleotide exchange factor 2	ARHGEF2	1,33
post-GPI attachment to proteins 1	PGAP1	1,33
dystrophin related protein 2	DRP2	1,33
Zhang2013 ALT_ACCEPTOR, ALT_DONOR, coding, INTERNAL, intronic best transcript NM_001033549	BABAM1	1,33
MAX network transcriptional repressor	MNT	1,33
long intergenic non-protein coding RNA 1089	LINC01089	1,33
microphthalmia-associated transcription factor	MITF	1,33
cadherin 22, type 2	CDH22	1,33
dickkopf WNT signaling pathway inhibitor 2	DKK2	1,33
lymphoid-restricted membrane protein	LRMP	1,33
SPATA31 subfamily E, member 1	SPATA31E1	1,33
caspase 12 (gene/pseudogene)	CASP12	1,33
protein tyrosine kinase 2 beta	PTK2B	1,33
leucine rich repeat containing 28	LRRC28	1,33
Transcript Identified by AceView, Entrez Gene ID(s) 728833	FAM72D	1,33
elongation factor RNA polymerase II-like 3	ELL3	1,33
programmed cell death 5	PDCD5	1,33
5-hydroxytryptamine (serotonin) receptor 3C, ionotropic	HTR3C	1,33
embigin	EMB	1,33
uroplakin 1B	UPK1B	1,33
syntrophin, gamma 1	SNTG1	1,33
DEAD (Asp-Glu-Ala-Asp) box polypeptide 59	DDX59	1,33

lymphocyte antigen 6 complex, locus D	LY6D	1,33
olfactory receptor, family 10, subfamily H, member 2	OR10H2	1,33
long intergenic non-protein coding RNA 1219	LINC01219	1,33
long intergenic non-protein coding RNA 1387	LINC01387	1,33
Memczak2013 ANTISENSE, CDS, coding, INTERNAL best transcript NM_004145	MYO9B	1,33
cytoplasmic polyadenylation element binding protein 3	CPEB3	1,33
interaction protein for cytohesin exchange factors 1	IPCEF1	1,33
colony stimulating factor 1 receptor	CSF1R	1,33
proline rich, Y-linked	PRORY	1,33
U2 small nuclear RNA auxiliary factor 1-like 4	U2AF1L4	1,33
nucleoporin 205kDa	NUP205	1,33
zinc finger, CW type with PWWP domain 2	ZCWPW2	1,33
ecto-NOX disulfide-thiol exchanger 1	ENOX1	1,33
basic leucine zipper transcription factor, ATF-like	BATF	1,33
Transcript Identified by AceView, Entrez Gene ID(s) 27332	ZNF638	1,33
BEN domain containing 6	BEND6	1,33
olfactory receptor, family 4, subfamily M, member 1	OR4M1	1,33
defensin, beta 136	DEFB136	1,33
chromosome 1 open reading frame 189	C1orf189	1,33
asparagine synthetase (glutamine-hydrolyzing)	ASNS	1,33
immunity-related GTPase family, M	IRGM	1,33
zinc finger protein 77	ZNF77	1,33
Rab interacting lysosomal protein-like 1	RILPL1	1,33
keratin associated protein 5-3	KRTAP5-3	1,33
flavin containing monooxygenase 2	FMO2	1,33
aminopeptidase-like 1	NPEPL1	1,33
DENN/MADD domain containing 2A	DENND2A	1,33
coiled-coil domain containing 83	CCDC83	1,33
histone cluster 1, H2aa	HIST1H2AA	1,33
solute carrier family 8 (sodium/lithium/calcium exchanger), member B1	SLC8B1	1,33
sparc/osteonectin, cwcv and kazal-like domains proteoglycan (testican) 3	SPOCK3	1,33
S-phase cyclin A-associated protein in the ER	SCAPER	1,33
glutamate receptor, ionotropic, AMPA 2	GRIA2	1,33
RUN and FYVE domain containing 3	RUFY3	1,33
Zhang2013 ALT_ACCEPTOR, ALT_DONOR, coding, INTERNAL, intronic best transcript NM_002520	NPM1	1,33
ferrochelatase	FECH	1,33
sortilin-related VPS10 domain containing receptor 1	SORCS1	1,33
phosphodiesterase 1B, calmodulin-dependent	PDE1B	1,33
Transcript Identified by AceView, Entrez Gene ID(s) 7520	XRCC5	1,33
haptoglobin	HP	1,33
chromosome 9 open reading frame 116	C9orf116	1,33

zinc finger protein 211	ZNF211	1,33
neurexophilin and PC-esterase domain family, member 3	NXPE3	1,33
guanine nucleotide binding protein (G protein), alpha inhibiting activity polypeptide 1	GNAI1	1,33
ring finger protein 44	RNF44	1,33
CD300a molecule	CD300A	1,33
cytochrome P450, family 39, subfamily A, polypeptide 1	CYP39A1	1,33
cadherin 15, type 1, M-cadherin (myotubule)	CDH15	1,33
caudal type homeobox 4	CDX4	1,32
hyperpolarization activated cyclic nucleotide gated potassium channel 3	HCN3	1,32
chromosome 20 open reading frame 27	C20orf27	1,32
keratin 76, type II	KRT76	1,32
lymphocyte antigen 9	LY9	1,32
tubulin, beta 6 class V	TUBB6	1,32
CTS telomere maintenance complex component 1	CTC1	1,32
inositol polyphosphate-5-phosphatase E	INPP5E	1,32
Kruppel-like factor 13	KLF13	1,32
TBC1 domain family, member 10C	TBC1D10C	1,32
family with sequence similarity 53, member A	FAM53A	1,32
signal-regulatory protein alpha	SIRPA	1,32
Jeck2013 ALT_ACCEPTOR, ALT_DONOR, coding, INTERNAL, intronic best transcript NM_016836	RBMS1	1,32
gremlin 1, DAN family BMP antagonist [Source:HGNC Symbol;Acc:HGNC:2001]	GREM1	1,32
chromosome 8 open reading frame 88	C8orf88	1,32
chromosome 12 open reading frame 54	C12orf54	1,32
ribonuclease L (2,5-oligoisoadenylate synthetase-dependent)	RNASEL	1,32
Memczak2013 ANTISENSE, coding, INTERNAL, intronic best transcript NM_001195144	ANKRD44	1,32
LSM family member 14B	LSM14B	1,32
ceroid-lipofuscinosis, neuronal 8	CLN8	1,32
Transcript Identified by AceView, Entrez Gene ID(s) 63892	THADA	1,32
DEXH (Asp-Glu-X-His) box polypeptide 58	DHX58	1,32
synovial sarcoma, X breakpoint 2; synovial sarcoma, X breakpoint 2B	SSX2; SSX2B	1,32
POU class 4 homeobox 1	POU4F1	1,32
nudix hydrolase 13	NUDT13	1,32
CD52 molecule	CD52	1,32
Memczak2013 ANTISENSE, coding, INTERNAL, intronic best transcript NM_181893	UBE2D3	1,32
long intergenic non-protein coding RNA 602	LINC00602	1,32
double zinc ribbon and ankyrin repeat domains 1	DZANK1	1,32
protocadherin 12	PCDH12	1,32
laminin, alpha 3	LAMA3	1,32

polycystic kidney disease 1-like 3	PKD1L3	1,32
long intergenic non-protein coding RNA 1189	LINC01189	1,32
S100 calcium binding protein A11	S100A11	1,32
vacuolar protein sorting 11 homolog (S. cerevisiae)	VPS11	1,32
ferredoxin 1-like	FDX1L	1,32
oligodendrocyte myelin glycoprotein	OMG	1,32
lipoma HMGIC fusion partner-like 4	LHFPL4	1,32
LHX4 antisense RNA 1; acyl-CoA binding domain containing 6	LHX4-AS1; ACBD6	1,32
Memczak2013 ALT_ACCEPTOR, ALT_DONOR, coding, INTERNAL, intronic best transcript NM_022492	TTC31	1,32
ubiquitin fusion degradation 1 like (yeast)	UFD1L	1,32
acyl-CoA dehydrogenase, long chain	ACADL	1,32
six transmembrane epithelial antigen of the prostate 1	STEAP1	1,32
lin-28 homolog A (C. elegans)	LIN28A	1,32
Transcript Identified by AceView, Entrez Gene ID(s) 9215	LARGE	1,32
Memczak2013 ALT_ACCEPTOR, ALT_DONOR, coding, INTERNAL, intronic best transcript NM_003369	UVRAG	1,32
kinesin family member 15	KIF15	1,32
mitogen-activated protein kinase kinase 6	MAP2K6	1,32
calcium binding protein 1	CABP1	1,32
Jeck2013 ALT_ACCEPTOR, ALT_DONOR, coding, INTERNAL, intronic best transcript NM_017774	CDKAL1	1,32
zinc finger protein 775	ZNF775	1,32
proteoglycan 4	PRG4	1,32
DNA (cytosine-5-)-methyltransferase 3 beta	DNMT3B	1,32
Jeck2013 ALT_ACCEPTOR, ALT_DONOR, coding, INTERNAL, intronic best transcript NM_006909	RASGRF2	1,32
solute carrier family 51, alpha subunit	SLC51A	1,31
Transcript Identified by AceView, Entrez Gene ID(s) 375248	ANKRD36	1,31
Rho guanine nucleotide exchange factor 4	ARHGEF4	1,31
Transcript Identified by AceView, Entrez Gene ID(s) 148753; novel transcript	RP11-12M5.1; FAM163A	1,31
UDP glycosyltransferase 3 family, polypeptide A2	UGT3A2	1,31
zinc finger protein 528	ZNF528	1,31
solute carrier family 14 (urea transporter), member 2	SLC14A2	1,31
Transcript Identified by AceView; family with sequence similarity 25, member D [Source:HGNC Symbol;Acc:23588]; family with sequence similarity 25, member E [Source:HGNC Symbol;Acc:HGNC:23587]	buskee; FAM25D; FAM25E; zawskaw	1,31
V-set and transmembrane domain containing 2B	VSTM2B	1,31
junctophilin 3	JPH3	1,31
tocopherol (alpha) transfer protein	TTPA	1,31
Transcript Identified by AceView, Entrez Gene ID(s) 9320	TRIP12	1,31
testis-specific serine kinase substrate	TSKS	1,31

runt-related transcription factor 1	RUNX1	1,31
MAS-related GPR, member F	MRGPRF	1,31
EPH receptor B1	EPHB1	1,31
vesicle associated membrane protein 1	VAMP1	1,31
protein tyrosine phosphatase, receptor type, C	PTPRC	1,31
gem nuclear organelle associated protein 5	GEMIN5	1,31
N-acetyltransferase 14 (GCN5-related, putative)	NAT14	1,31
zinc finger protein 584	ZNF584	1,31
Transcript Identified by AceView, Entrez Gene ID(s) 4798	NFRKB	1,31
nebulette	NEBL	1,31
integrator complex subunit 8	INTS8	1,31
cysteine-rich, angiogenic inducer, 61	CYR61	1,31
hes family bHLH transcription factor 5	HES5	1,31
progestin and adipoQ receptor family member VI	PAQR6	1,31
plexin C1	PLXNC1	1,31
neuromedin U receptor 2	NMUR2	1,31
taste receptor, type 2, member 31	TAS2R31	1,31
NOP2/Sun domain family, member 7	NSUN7	1,31
keratin associated protein 20-1	KRTAP20-1	1,31
regulator of G-protein signaling 11	RGS11	1,31
Transcript Identified by AceView, Entrez Gene ID(s) 57498; 100216337	KIDINS220	1,31
FXYD domain containing ion transport regulator 5	FXYD5	1,31
discs, large homolog 5 (Drosophila)	DLG5	1,31
long intergenic non-protein coding RNA 337	LINC00337	1,31
BEN domain containing 5	BEND5	1,31
family with sequence similarity 221, member A	FAM221A	1,31
chloride intracellular channel 6	CLIC6	1,31
BCL2-like 10 (apoptosis facilitator)	BCL2L10	1,31
selenoprotein V; selenoprotein V [Source:EntrezGene;Acc:348303]; Transcript Identified by AceView, Entrez Gene ID(s) 348303, RefSeq ID(s) NM_182704	SELV	1,31
ethanolamine kinase 2	ETNK2	1,31
ELL associated factor 2	EAF2	1,31
tripartite motif containing 16-like	TRIM16L	1,31
A kinase (PRKA) interacting protein 1	AKIP1	1,31
stannin	SNN	1,31
BCL2-like 14 (apoptosis facilitator)	BCL2L14	1,31
zinc finger protein 812, pseudogene	ZNF812P	1,31
thymocyte selection associated	THEMIS	1,31
Transcript Identified by AceView, Entrez Gene ID(s) 22873	DZIP1	1,31
COX14 cytochrome c oxidase assembly factor	COX14	1,31
chromosome 10 open reading frame 11	C10orf11	1,31
Memczak2013 ANTISENSE, CDS, coding, INTERNAL best transcript NM_006901	MYO9A	1,31

Zhang2013 ALT_ACCEPTOR, ALT_DONOR, coding, INTERNAL, intronic best transcript NM_002482	NASP	1,31
developmental pluripotency associated 4	DPPA4	1,31
claudin 5	CLDN5	1,31
mucosal vascular addressin cell adhesion molecule 1	MADCAM1	1,31
prolyl-tRNA synthetase 2, mitochondrial (putative)	PARS2	1,31
uncharacterized LOC441178; novel tanscript	LOC441178; RP11-351J23.1	1,31
pregnancy specific beta-1-glycoprotein 9	PSG9	1,31
membrane-spanning 4-domains, subfamily A, member 6E	MS4A6E	1,31
chromosome 3 open reading frame 56	C3orf56	1,31
atypical chemokine receptor 1 (Duffy blood group)	ACKR1	1,31
synaptotagmin II	SYT2	1,31
GDNF family receptor alpha 1	GFRA1	1,31
calcitonin receptor	CALCR	1,31
proline rich 5 (renal)	PRR5	1,31
bladder cancer associated protein	BLCAP	1,31
olfactory receptor, family 4, subfamily C, member 6	OR4C6	1,31
protein phosphatase, EF-hand calcium binding domain 2	PPEF2	1,31
transmembrane channel like 6	TMC6	1,31
Transcript Identified by AceView, Entrez Gene ID(s) 23175	LPIN1	1,31
uveal autoantigen with coiled-coil domains and ankyrin repeats	UACA	1,31
microfibrillar associated protein 3 like	MFAP3L	1,31
retinal pigment epithelium-derived rhodopsin homolog	RRH	1,31
sperm associated antigen 16	SPAG16	1,31
zinc finger, BED-type containing 3	ZBED3	1,31
family with sequence similarity 65, member C	FAM65C	1,31
zinc finger protein 197	ZNF197	1,31
unc-13 homolog C (C. elegans)	UNC13C	1,31
kalirin, RhoGEF kinase	KALRN	1,31
Transcript Identified by AceView, Entrez Gene ID(s) 640	BLK	1,31
diacylglycerol kinase, delta 130kDa	DGKD	1,31
prune homolog 2 (Drosophila)	PRUNE2	1,31
protein phosphatase, Mg2+/Mn2+ dependent, 1J	PPM1J	1,31
ephrin-A4	EFNA4	1,31
component of oligomeric golgi complex 1	COG1	1,31
SPANX family, member N3	SPANXN3	1,31
MICAL-like 1	MICALL1	1,30
chromosome 8 open reading frame 34	C8orf34	1,30
serum amyloid A2; SAA2-SAA4 readthrough; serum amyloid A4, constitutive	SAA2; SAA2-SAA4; SAA4	1,30
IZUMO family member 2	IZUMO2	1,30
ankyrin repeat domain 34A	ANKRD34A	1,30
importin 4	IPO4	1,30
olfactory receptor, family 5, subfamily M, member 11	OR5M11	1,30

ubiquitin specific peptidase 6	USP6	1,30
chondrosarcoma associated gene 1	CSAG1	1,30
olfactory receptor, family 4, subfamily F, member 3	OR4F3	1,30
fibrillarin	FBL	1,30
trafficking protein particle complex 4	TRAPPC4	1,30
RAB11 family interacting protein 3 (class II)	RAB11FIP3	1,30
N-acetyltransferase 1 (arylamine N-acetyltransferase)	NAT1	1,30
serine/threonine kinase-like domain containing 1 [Source:HGNC Symbol;Acc:HGNC:28669]	STKLD1	1,30
Zhang2013 ALT_ACCEPTOR, ALT_DONOR, coding, INTERNAL, intronic best transcript NM_004559	YBX1	1,30
thyrotropin-releasing hormone degrading enzyme	TRHDE	1,30
MAP/microtubule affinity-regulating kinase 1	MARK1	1,30
MLX interacting protein	MLXIP	1,30
ring finger protein 146	RNF146	1,30
interferon regulatory factor 2 binding protein 1	IRF2BP1	1,30
sciellin	SCEL	1,30
Memczak2013 ALT_ACCEPTOR, ALT_DONOR, coding, INTERNAL, intronic best transcript NM_015275	KIAA1033	1,30
inositol polyphosphate-5-phosphatase D	INPP5D	1,30
centrosomal protein 128kDa	CEP128	1,30
ZNF582 antisense RNA 1 (head to head)	ZNF582-AS1	1,30
olfactory receptor, family 2, subfamily M, member 5	OR2M5	1,30
glycine-N-acyltransferase	GLYAT	1,30
solute carrier family 25 (S-adenosylmethionine carrier), member 26	SLC25A26	1,30
arylsulfatase F	ARSF	1,30
CAP-GLY domain containing linker protein family, member 4	CLIP4	1,30
potassium channel, inwardly rectifying subfamily J, member 11	KCNJ11	1,30
keratin associated protein 2-4	KRTAP2-4	1,30
reticulocalbin 3, EF-hand calcium binding domain	RCN3	1,30
zinc finger protein 212	ZNF212	1,30
Memczak2013 ALT_ACCEPTOR, ALT_DONOR, coding, INTERNAL, intronic best transcript NM_005534	IFNGR2	1,30
family with sequence similarity 209, member B	FAM209B	1,30
kelch-like family member 10	KLHL10	1,30
keratin 24, type I	KRT24	1,30
glial cells missing homolog 2 (Drosophila)	GCM2	1,30
fibrinogen-like 2	FGL2	1,30
folate hydrolase (prostate-specific membrane antigen) 1	FOLH1	1,30
carcinoembryonic antigen-related cell adhesion molecule 19	CEACAM19	1,30
coiled-coil domain containing 84	CCDC84	1,30
v-rel avian reticuloendotheliosis viral oncogene homolog B	RELB	1,30
ADAM metallopeptidase with thrombospondin type 1 motif 12	ADAMTS12	1,30
tumor protein p63	TP63	1,30

RAB15, member RAS oncogene family	RAB15	1,30
immunoglobulin-like domain containing receptor 2	ILDR2	1,30
Zhang2013 ALT_ACCEPTOR, ALT_DONOR, coding, INTERNAL, intronic, OVERLAPTX best transcript NM_014501	UBE2S	1,30
integrin alpha 7	ITGA7	1,30
Transcript Identified by AceView, Entrez Gene ID(s) 80230	RUFY1	1,30
double C2-like domains, alpha	DOC2A	1,30
CD40 molecule, TNF receptor superfamily member 5	CD40	1,30
elastin	ELN	1,30
Memczak2013 ANTISENSE, coding, INTERNAL, intronic best transcript NM_175900; novel transcript, antisense to C16orf54	AC009133.17; C16orf54	1,30
glucocorticoid induced 1	GLCCI1	1,30
chemokine (C-X-C motif) ligand 8	CXCL8	1,30
FERM and PDZ domain containing 2	FRMPD2	1,30
diacylglycerol kinase alpha	DGKA	1,30
chromosome 17 open reading frame 67	C17orf67	1,30
HIG1 hypoxia inducible domain family, member 1B	HIGD1B	1,30
Transcript Identified by AceView, Entrez Gene ID(s) 26269	FBXO8	1,30
zinc finger, DHHC-type containing 3	ZDHHC3	1,30
LIM homeobox transcription factor 1, beta	LMX1B	1,30
AHNAK nucleoprotein	AHNAK	1,30
pregnancy specific beta-1-glycoprotein 5	PSG5	1,30
brain expressed X-linked 1	BEX1	1,30
KIAA2012	KIAA2012	1,30
zinc finger protein 385A	ZNF385A	1,30
G protein-coupled receptor 153	GPR153	1,30
suprabasin	SBSN	1,30
C-type lectin domain family 4, member E	CLEC4E	1,30
placenta expressed transcript 1	PLET1	1,30
relaxin 3	RLN3	1,30
S-antigen; retina and pineal gland (arrestin)	SAG	1,30
ring finger protein 152	RNF152	1,30
glypican 4	GPC4	1,30
transmembrane channel like 8	TMC8	1,30
potassium channel, voltage gated eag related subfamily H, member 8	KCNH8	1,30
chromosome 6 open reading frame 229	C6orf229	1,30
potassium channel, two pore domain subfamily K, member 15	KCNK15	1,30
centromere protein C	CENPC	1,30
Transcript Identified by AceView, Entrez Gene ID(s) 79875	THSD4	1,30
defensin, beta 114	DEFB114	1,30
transmembrane protein 196	TMEM196	1,30
amylase, alpha 2B (pancreatic); actin gamma 1 pseudogene 4	AMY2B; ACTG1P4	1,30
glutathione S-transferase alpha 2	GSTA2	1,30

L antigen family, member 3	LAGE3	1,30
golgin A8 family, member F; golgin A8 family, member G	GOLGA8F; GOLGA8G	1,30
olfactory receptor, family 52, subfamily E, member 2	OR52E2	1,30
golgin A8 family, member K; ULK4 pseudogene 1	GOLGA8K; ULK4P1	1,30
G protein-coupled receptor 158	GPR158	1,30
FES proto-oncogene, tyrosine kinase	FES	1,30
late endosomal/lysosomal adaptor, MAPK and MTOR activator 1	LAMTOR1	1,30
G antigen 2B; G antigen 2A; G antigen 2C	GAGE2B; GAGE2A; GAGE2C	1,30
calreticulin 3	CALR3	1,30
FERM domain containing 7	FRMD7	1,30
complexin 3	CPLX3	1,30
echinoderm microtubule associated protein like 6	EML6	1,30
KH domain containing, RNA binding, signal transduction associated 3	KHDRBS3	1,30
Fanconi anemia complementation group B	FANCB	1,29
carnitine O-octanoyltransferase	CROT	1,29
zinc finger protein 773	ZNF773	1,29
growth associated protein 43	GAP43	1,29
dorsal root ganglia homeobox	DRGX	1,29
actin-like 6B	ACTL6B	1,29
collagen, type VIII, alpha 1	COL8A1	1,29
olfactory receptor, family 52, subfamily E, member 1 (gene/pseudogene)	OR52E1	1,29
Transcript Identified by AceView, Entrez Gene ID(s) 149840	C20orf196	1,29
sterile alpha motif domain containing 4A	SAMD4A	1,29
olfactory receptor, family 3, subfamily A, member 4 pseudogene; olfactory receptor, family 3, subfamily A, member 5 pseudogene	OR3A4P; OR3A5P	1,29
brain-derived neurotrophic factor	BDNF	1,29
protein kinase D1	PRKD1	1,29
small integral membrane protein 22	SMIM22	1,29
family with sequence similarity 229, member A	FAM229A	1,29
secreted frizzled-related protein 5	SFRP5	1,29
leucine rich repeat containing 37, member A3	LRRC37A3	1,29
maestro heat-like repeat family member 2B	MROH2B	1,29
zinc finger protein 71	ZNF71	1,29
ZNF625-ZNF20 readthrough (NMD candidate); zinc finger protein 625	ZNF625-ZNF20; ZNF625	1,29
endo-beta-N-acetylglucosaminidase	ENGASE	1,29
family with sequence similarity 179, member B	FAM179B	1,29

Transcript Identified by AceView, Entrez Gene ID(s) 54758; 100129637	KLHDC4	1,29
cylicin, basic protein of sperm head cytoskeleton 2	CYLC2	1,29
cytochrome P450, family 4, subfamily F, polypeptide 11	CYP4F11	1,29
malic enzyme 2, NAD(+)-dependent, mitochondrial	ME2	1,29
synaptonemal complex central element protein 2	SYCE2	1,29
zinc finger protein 786	ZNF786	1,29
RAB27B, member RAS oncogene family	RAB27B	1,29
Memczak2013 ALT_ACCEPTOR, ALT_DONOR, coding, INTERNAL, intronic best transcript NM_001076787	TP53I11	1,29
plasminogen activator, urokinase receptor	PLAUR	1,29
small integral membrane protein 8	SMIM8	1,29
Memczak2013 ANTISENSE, CDS, coding, INTERNAL best transcript NM_001080453	INTS1	1,29
RNA binding protein with multiple splicing 2	RBPMS2	1,29
olfactory receptor, family 13, subfamily D, member 1	OR13D1	1,29
zinc finger protein 780A	ZNF780A	1,29
egl-9 family hypoxia-inducible factor 2	EGLN2	1,29
centrosomal protein 112kDa	CEP112	1,29
histidine-rich glycoprotein	HRG	1,29
Purkinje cell protein 4	PCP4	1,29
calpain 10	CAPN10	1,29
coiled-coil domain containing 97	CCDC97	1,29
piggyBac transposable element derived 5	PGBD5	1,29
zinc finger protein 660	ZNF660	1,29
WD repeat domain 53	WDR53	1,29
zinc finger protein 595	ZNF595	1,29
ST3 beta-galactoside alpha-2,3-sialyltransferase 3; microRNA 6079	ST3GAL3; MIR6079	1,29
MX dynamin-like GTPase 1	MX1	1,29
frizzled class receptor 1	FZD1	1,29
ATP binding cassette subfamily C member 6	ABCC6	1,29
calcitonin receptor like receptor	CALCRL	1,29
zinc finger protein 416	ZNF416	1,29
PWWP domain containing 2B	PWWP2B	1,29
collagen-like tail subunit (single strand of homotrimer) of asymmetric acetylcholinesterase	COLQ	1,29
general transcription factor IIH subunit 4	GTF2H4	1,29
solute carrier family 5 (glucose activated ion channel), member 4	SLC5A4	1,29
spermatogenesis associated 5	SPATA5	1,29
KAT8 regulatory NSL complex subunit 1 like	KANSL1L	1,29
carbonic anhydrase I	CA1	1,29
CD200 receptor 1	CD200R1	1,29
progestagen-associated endometrial protein	PAEP	1,29

SH3 domain binding glutamate-rich protein like 3	SH3BGRL3	1,29
relaxin 1	RLN1	1,29
vomeronasal 1 receptor 10 pseudogene	VN1R10P	1,29
NRDE-2, necessary for RNA interference, domain containing	NRDE2	1,29
von Willebrand factor A domain containing 7	VWA7	1,29
serine peptidase inhibitor, Kazal type 1	SPINK1	1,29
tropomodulin 4 (muscle)	TMOD4	1,29
growth hormone receptor	GHR	1,29
transcription factor Dp family, member 3	TFDP3	1,29
triggering receptor expressed on myeloid cells 1	TREM1	1,29
zinc finger, DHHC-type containing 1	ZDHHC1	1,29
phospholipid scramblase 3; transmembrane protein 256; TMEM256-PLSCR3 readthrough (NMD candidate)	PLSCR3; TMEM256; TMEM256-PLSCR3	1,29
insulin-like 4 (placenta)	INSL4	1,29
glutamate rich 5	ERICH5	1,29
echinoderm microtubule associated protein like 2; microRNA 330	EML2; MIR330	1,29
MOB kinase activator 3C	MOB3C	1,29
dual specificity phosphatase 27 (putative)	DUSP27	1,29
gem nuclear organelle associated protein 2	GEMIN2	1,29
olfactory receptor, family 1, subfamily S, member 1 (gene/pseudogene)	OR1S1	1,29
coiled-coil domain containing 64	CCDC64	1,29
transmembrane protein 105	TMEM105	1,29
ALDH1L1 antisense RNA 1	ALDH1L1-AS1	1,29
ATPase, aminophospholipid transporter, class I, type 8A, member 2	ATP8A2	1,29
nuclear factor of kappa light polypeptide gene enhancer in B-cells inhibitor, zeta	NFKBIZ	1,29
neurotrophic tyrosine kinase, receptor, type 1	NTRK1	1,29
dermatopontin	DPT	1,29
asporin	ASPN	1,29
calcium-sensing receptor	CASR	1,29
leucine-rich, glioma inactivated 1	LGI1	1,29
glycine receptor beta	GLRB	1,29
FYVE, RhoGEF and PH domain containing 4	FGD4	1,29
tripartite motif containing 73	TRIM73	1,29
tetraspanin 32	TSPAN32	1,29
protease, serine, 22	PRSS22	1,29
Rh family, B glycoprotein (gene/pseudogene)	RHBG	1,29
linker for activation of T-cells family member 2	LAT2	1,29
peptidyl arginine deiminase, type IV	PADI4	1,29
solute carrier family 35, member G4	SLC35G4	1,29

protein inhibitor of activated STAT 2	PIAS2	1,29
chromosome 19 open reading frame 81	C19orf81	1,29
Transcript Identified by AceView, Entrez Gene ID(s) 23126	POGZ	1,29
Ras association (RalGDS/AF-6) domain family (N-terminal) member 10	RASSF10	1,29
family with sequence similarity 131, member B	FAM131B	1,28
seryl-tRNA synthetase 2, mitochondrial	SARS2	1,28
pleckstrin homology domain containing, family O member 2	PLEKHO2	1,28
proline rich Gla (G-carboxyglutamic acid) 2	PRRG2	1,28
tetratricopeptide repeat, ankyrin repeat and coiled-coil containing 2	TANC2	1,28
polymerase (DNA directed), lambda	POLL	1,28
ribosomal protein S6 kinase, 90kDa, polypeptide 5	RPS6KA5	1,28
adhesion G protein-coupled receptor G1	ADGRG1	1,28
natural killer cell granule protein 7	NKG7	1,28
ribosomal L24 domain containing 1	RSL24D1	1,28
potassium channel subfamily M regulatory beta subunit 3	KCNMB3	1,28
retinol dehydrogenase 12 (all-trans/9-cis/11-cis)	RDH12	1,28
olfactory receptor, family 9, subfamily K, member 2	OR9K2	1,28
zinc finger protein 57	ZNF57	1,28
fetal and adult testis expressed 1	FATE1	1,28
DEAD (Asp-Glu-Ala-Asp) box polypeptide 52	DDX52	1,28
tubulointerstitial nephritis antigen	TINAG	1,28
S100 calcium binding protein A7	S100A7	1,28
histone cluster 1, H1d	HIST1H1D	1,28
NDRG family member 4	NDRG4	1,28
novel transcript; Transcript Identified by AceView, Entrez Gene ID(s) 55737	RP11-93O14.2; VPS35	1,28
janus kinase and microtubule interacting protein 2	JAKMIP2	1,28
death associated protein like 1; olfactory receptor, family 7, subfamily E, member 89 pseudogene	DAPL1; OR7E89P	1,28
doublecortin-like kinase 3	DCLK3	1,28
sarcoglycan alpha	SGCA	1,28
leukocyte receptor cluster (LRC) member 8	LENG8	1,28
defensin, beta 125	DEFB125	1,28
F-box protein 8	FBXO8	1,28
CD47 molecule	CD47	1,28
sorting nexin 18	SNX18	1,28
synaptoporin	SYNPR	1,28
RNA binding motif protein 23	RBM23	1,28
leucine rich repeat containing 74A	LRRC74A	1,28
thyroid hormone receptor interactor 6; microRNA 6875	TRIP6; MIR6875	1,28
acyl-CoA synthetase medium-chain family member 1	ACSM1	1,28
melanophilin	MLPH	1,28

S100 calcium binding protein B	S100B	1,28
DnaJ (Hsp40) homolog, subfamily C, member 30	DNAJC30	1,28
olfactory receptor, family 5, subfamily T, member 2	OR5T2	1,28
tripartite motif containing 59	TRIM59	1,28
glutamate decarboxylase like 1	GADL1	1,28
transient receptor potential cation channel, subfamily M, member 2	TRPM2	1,28
HECT, C2 and WW domain containing E3 ubiquitin protein ligase 2	HECW2	1,28
zinc finger protein 579	ZNF579	1,28
chromosome 1 open reading frame 229	C1orf229	1,28
agouti signaling protein	ASIP	1,28
PIH1 domain containing 2	PIH1D2	1,28
G protein-coupled receptor 52	GPR52	1,28
enolase superfamily member 1	ENOSF1	1,28
trace amine associated receptor 9 (gene/pseudogene)	TAAR9	1,28
BEAN1 antisense RNA 1	BEAN1-AS1	1,28
solute carrier family 7 (amino acid transporter light chain, bo,+ system), member 9	SLC7A9	1,28
protein phosphatase 1, regulatory subunit 42	PPP1R42	1,28
neuron navigator 3	NAV3	1,28
signal-regulatory protein delta	SIRPD	1,28
dual specificity phosphatase 28	DUSP28	1,28
regulator of telomere elongation helicase 1; tumor necrosis factor receptor superfamily, member 6b, decoy; RTEL1-TNFRSF6B readthrough (NMD candidate)	RTEL1; TNFRSF6B; RTEL1-TNFRSF6B	1,28
D-amino acid oxidase activator	DAOA	1,28
zinc finger protein 141	ZNF141	1,28
H2.0-like homeobox	HLX	1,28
CD276 molecule	CD276	1,28
coiled-coil domain containing 28B	CCDC28B	1,28
family with sequence similarity 3, member A	FAM3A	1,28
novel transcript, antisense to KCNIP4; Transcript Identified by AceView, Entrez Gene ID(s) 133015	RP11-362J17.1; PACRGL	1,28
brain expressed X-linked 2	BEX2	1,28
stromal interaction molecule 2	STIM2	1,28
formin homology 2 domain containing 3	FHOD3	1,28
bradykinin receptor B1	BDKRB1	1,28
phospholipase A2, group XVI	PLA2G16	1,28
sorting nexin 32	SNX32	1,28
centrosomal protein 76kDa	CEP76	1,28
small proline-rich protein 2D	SPRR2D	1,28
delta/notch like EGF repeat containing	DNER	1,28
tudor domain containing 12	TDRD12	1,28

tumor suppressor candidate 2	TUSC2	1,28
sulfotransferase family 1C member 3	SULT1C3	1,28
zinc finger homeobox 2	ZFHX2	1,28
chemokine (C-C motif) receptor 2	CCR2	1,28
frequently rearranged in advanced T-cell lymphomas 1	FRAT1	1,28
synaptopodin	SYNPO	1,28
NUAK family, SNF1-like kinase, 1	NUAK1	1,28
plasminogen activator, urokinase	PLAU	1,28
coiled-coil domain containing 17	CCDC17	1,28
tRNA methyltransferase 2 homolog B	TRMT2B	1,28
integrin beta 3	ITGB3	1,28
zinc finger, NFX1-type containing 1	ZNFX1	1,28
granulysin	GNLY	1,28
G antigen 1	GAGE1	1,28
distal-less homeobox 6	DLX6	1,28
zinc finger, CW type with PWWP domain 1	ZCWPW1	1,28
CCNT2 antisense RNA 1	CCNT2-AS1	1,28
fucokinase	FUK	1,28
G elongation factor, mitochondrial 2	GFM2	1,28
Josephin domain containing 2	JOSD2	1,28
Transcript Identified by AceView, Entrez Gene ID(s) 80205	CHD9	1,27
leucine rich repeat containing 17	LRRC17	1,27
GSG1-like 2	GSG1L2	1,27
transmembrane protein 17	TMEM17	1,27
ubiquitin interaction motif containing 1	UIMC1	1,27
ankyrin repeat and MYND domain containing 1	ANKMY1	1,27
K(lysine) acetyltransferase 6B	KAT6B	1,27
Transcript Identified by AceView, Entrez Gene ID(s) 64895	PAPOLG	1,27
follistatin-like 5	FSTL5	1,27
tweety family member 1	TTYH1	1,27
chromosome 14 open reading frame 180	C14orf180	1,27
Tctex1 domain containing 1	TCTEX1D1	1,27
tumor necrosis factor receptor superfamily, member 25	TNFRSF25	1,27
fibroblast growth factor 20	FGF20	1,27
tripartite motif containing 77	TRIM77	1,27
small VCP/p97-interacting protein	SVIP	1,27
retinoic acid receptor responder (tazarotene induced) 3	RARRES3	1,27
DENN/MADD domain containing 4B	DENND4B	1,27
core-binding factor, runt domain, alpha subunit 2; translocated to, 3	CBFA2T3	1,27
T-cell activation RhoGTPase activating protein	TAGAP	1,27
zinc finger protein 354A	ZNF354A	1,27
solute carrier family 25 (mitochondrial carrier; adenine nucleotide translocator), member 31	SLC25A31	1,27
synaptotagmin-like 2	SYTL2	1,27

keratin associated protein 5-6	KRTAP5-6	1,27
chromosome 16 open reading frame 89	C16orf89	1,27
uncharacterized LOC389199; Transcript Identified by AceView, Entrez Gene ID(s) 389199; novel transcript	LOC389199; AC097381.1	1,27
v-akt murine thymoma viral oncogene homolog 3	AKT3	1,27
chondroitin sulfate proteoglycan 4 pseudogene 1, Y-linked	CSPG4P1Y	1,27
gonadotropin releasing hormone receptor	GNRHR	1,27
uncharacterized LOC100130691; Transcript Identified by AceView, Entrez Gene ID(s) 100130691; 4780, RefSeq ID(s) NR_026966; novel transcript	LOC100130691; AC074286.1; NFE2L2	1,27
zinc finger protein 627	ZNF627	1,27
guanine nucleotide binding protein (G protein), beta polypeptide 3	GNB3	1,27
delta-like 4 (Drosophila)	DLL4	1,27
spermatogenesis associated 45	SPATA45	1,27
Memczak2013 ALT_ACCEPTOR, ALT_DONOR, coding, INTERNAL, intronic best transcript NM_006586	CNPY3	1,27
p21 protein (Cdc42/Rac)-activated kinase 1	PAK1	1,27
clathrin, light chain B	CLTB	1,27
CUGBP, Elav-like family member 1	CELF1	1,27
peptidoglycan recognition protein 4	PGLYRP4	1,27
chromosome 2 open reading frame 73	C2orf73	1,27
neuropeptide S receptor 1	NPSR1	1,27
family with sequence similarity 65, member A	FAM65A	1,27
zinc finger protein 385C	ZNF385C	1,27
receptor (chemosensory) transporter protein 4	RTP4	1,27
FSHD region gene 2 family, member B	FRG2B	1,27
dishevelled-binding antagonist of beta-catenin 2	DACT2	1,27
testis expressed 9	TEX9	1,27
Memczak2013 ANTISENSE, coding, INTERNAL, intronic best transcript NM_175061	JAZF1	1,27
SHC (Src homology 2 domain containing) family, member 4	SHC4	1,27
cartilage intermediate layer protein 2	CILP2	1,27
SEC14-like lipid binding 4	SEC14L4	1,27
defensin, beta 106A	DEFB106A	1,27
PDZ domain containing 2	PDZD2	1,27
sodium channel, non voltage gated 1 alpha subunit	SCNN1A	1,27
EMG1 N1-specific pseudouridine methyltransferase	EMG1	1,27
urotensin 2 receptor	UTS2R	1,27
stabilizer of axonemal microtubules 2	SAXO2	1,27
aprataxin and PNKP like factor	APLF	1,27
sphingomyelin phosphodiesterase 2, neutral membrane (neutral sphingomyelinase)	SMPD2	1,27
proline rich 14-like	PRR14L	1,27
defensin, beta 134	DEFB134	1,27

zinc finger protein, FOG family member 2	ZFPM2	1,27
trace amine associated receptor 3 (gene/pseudogene)	TAAR3	1,27
transmembrane protein 238	TMEM238	1,27
chromosome 8 open reading frame 49	C8orf49	1,27
Memczak2013 ANTISENSE, coding, INTERNAL, UTR3 best transcript NM_080677	DYNLL2	1,27
zinc finger and SCAN domain containing 10	ZSCAN10	1,27
ectonucleoside triphosphate diphosphohydrolase 8	ENTPD8	1,27
coiled-coil domain containing 84	CCDC84	1,27
synaptonemal complex protein 3	SYCP3	1,27
transglutaminase 4	TGM4	1,27
olfactory receptor, family 51, subfamily G, member 1 (gene/pseudogene)	OR51G1	1,27
Fc fragment of IgA receptor	FCAR	1,27
contactin associated protein 1	CNTNAP1	1,27
defensin, beta 103A	DEFB103A	1,27
testis-specific serine kinase 3	TSSK3	1,27
pleckstrin homology domain containing, family F (with FYVE domain) member 1	PLEKHF1	1,27
ectodysplasin A receptor	EDAR	1,27
zinc finger protein 649	ZNF649	1,27
DIRAS family, GTP-binding RAS-like 2	DIRAS2	1,27
phosphofurin acidic cluster sorting protein 1	PACS1	1,27
testis expressed sequence 13-like	LOC100129520	1,27
ets variant 7	ETV7	1,27
vesicle associated membrane protein 4	VAMP4	1,27
chromosome 12 open reading frame 74	C12orf74	1,27
family with sequence similarity 9, member C	FAM9C	1,27
cyclin-dependent kinase 5, regulatory subunit 2 (p39)	CDK5R2	1,27
protein kinase C, alpha	PRKCA	1,27
trimethylguanosine synthase 1	TGS1	1,27
TP53 target 5	TP53TG5	1,27
barrier to autointegration factor 2	BANF2	1,27
fibroblast growth factor (acidic) intracellular binding protein	FIBP	1,27
collagen, type IV, alpha 4	COL4A4	1,27
integrator complex subunit 1	INTS1	1,27
taste receptor, type 2, member 14	TAS2R14	1,27
secreted frizzled-related protein 4	SFRP4	1,27
dynein, cytoplasmic 1, intermediate chain 1	DYNC1I1	1,27
platelet endothelial aggregation receptor 1	PEAR1	1,27
C-type lectin domain family 5, member A	CLEC5A	1,27
transketolase-like 1	TKTL1	1,27
Memczak2013 ANTISENSE, coding, INTERNAL, UTR3 best transcript NM_014310	RASD2	1,27
coiled-coil domain containing 70	CCDC70	1,27

defensin, beta 116	DEFB116	1,27
5-phosphohydroxy-L-lysine phospho-lyase	PHYKPL	1,27
moesin	MSN	1,27
olfactory receptor, family 5, subfamily AN, member 1	OR5AN1	1,27
poliovirus receptor-related 4	PVRL4	1,27
transmembrane protein 81	TMEM81	1,27
proline/arginine-rich end leucine-rich repeat protein	PRELP	1,27
pregnancy specific beta-1-glycoprotein 3	PSG3	1,27
3-phosphoinositide dependent protein kinase 1	PDPK1	1,27
chemokine (C-C motif) ligand 22	CCL22	1,26
Transcript Identified by AceView, Entrez Gene ID(s) 54715	A2BP1	1,26
GATA binding protein 1 (globin transcription factor 1)	GATA1	1,26
arylacetamide deacetylase-like 2	AADACL2	1,26
Transcript Identified by AceView, Entrez Gene ID(s) 729475	RAD51AP2	1,26
G protein-coupled receptor 85	GPR85	1,26
ubiquitin specific peptidase 30	USP30	1,26
zinc finger protein 568	ZNF568	1,26
RGD motif, leucine rich repeats, tropomodulin domain and proline-rich containing	RLTPR	1,26
potassium channel, voltage gated Shaw related subfamily C, member 2	KCNC2	1,26
uncharacterized LOC100131315; novel transcript	LOC100131315; RP11-758N13.1	1,26
UDP-Gal:betaGlcNAc beta 1,3-galactosyltransferase 2	B3GALT2	1,26
Jeck2013 ALT_ACCEPTOR, ALT_DONOR, coding, INTERNAL, intronic best transcript NM_002841	PTPRG	1,26
katanin p60 subunit A-like 2	KATNAL2	1,26
MYB antisense RNA 1 [Source:HGNC Symbol;Acc:HGNC:37457]; Memczak2013 ANTISENSE, coding, INTERNAL, intronic best transcript NM_005375; putative novel transcript; Transcript Identified by AceView	MYB-AS1; MYB; RP1-32B1.3; rawjer	1,26
chromosome 8 open reading frame 86	C8orf86	1,26
fibroblast growth factor 14; FGF14 intronic transcript 1	FGF14; FGF14-IT1	1,26
protocadherin beta 14	PCDHB14	1,26
family with sequence similarity 86, member B2	FAM86B2	1,26
aldo-keto reductase family 1, member B15	AKR1B15	1,26
Sp8 transcription factor	SP8	1,26
neutral sphingomyelinase activation associated factor	NSMAF	1,26
olfactory receptor, family 5, subfamily M, member 1	OR5M1	1,26
acetyl-CoA carboxylase alpha	ACACA	1,26
olfactory receptor, family 5, subfamily L, member 1 (gene/pseudogene)	OR5L1	1,26
solute carrier family 35, member F4	SLC35F4	1,26

FERM domain containing 5	FRMD5	1,26
leucine rich repeat containing 49	LRRC49	1,26
transmembrane protein 40	TMEM40	1,26
tumor necrosis factor receptor superfamily, member 10a	TNFRSF10A	1,26
chromosome 15 open reading frame 54	C15orf54	1,26
olfactory receptor, family 5, subfamily K, member 4	OR5K4	1,26
RAS-like, estrogen-regulated, growth inhibitor	RERG	1,26
phosphatidylinositol-5-phosphate 4-kinase, type II, alpha	PIP4K2A	1,26
carbonic anhydrase III	CA3	1,26
BPI fold containing family A, member 2	BPIFA2	1,26
polymerase (RNA) II (DNA directed) polypeptide G	POLR2G	1,26
coiled-coil and C2 domain containing 1A	CC2D1A	1,26
galanin-like peptide	GALP	1,26
fatty acid binding protein 12	FABP12	1,26
ribosomal modification protein rimK-like family member B	RIMKLB	1,26
Transcript Identified by AceView, Entrez Gene ID(s) 8453; novel transcript	CUL2; RP11-297A16.2	1,26
heat shock 70kDa protein 1-like	HSPA1L	1,26
3-oxoacyl-ACP synthase, mitochondrial	OXSM	1,26
cyclin-dependent kinase-like 5	CDKL5	1,26
RWD domain containing 3; transmembrane protein 56; TMEM56-RWDD3 readthrough	RWDD3; TMEM56; TMEM56-RWDD3	1,26
selenocysteine lyase	SCLY	1,26
kallikrein related peptidase 13	KLK13	1,26
UBX domain protein 2B	UBXN2B	1,26
spermatogenesis associated 5-like 1	SPATA5L1	1,26
leucine zipper, putative tumor suppressor 2	LZTS2	1,26
transmembrane and coiled-coil domain family 2	TMCC2	1,26
interleukin 36, gamma	IL36G	1,26
methyltransferase like 1	METTL1	1,26
protein tyrosine phosphatase, non-receptor type 14	PTPN14	1,26
small integral membrane protein 19	SMIM19	1,26
progestin and adipoQ receptor family member III	PAQR3	1,26
interleukin 15 receptor, alpha	IL15RA	1,26
zinc finger protein 487	ZNF487	1,26
retinitis pigmentosa 1-like 1	RP1L1	1,26
transcription elongation factor A (SII)-like 5	TCEAL5	1,26
PITH (C-terminal proteasome-interacting domain of thioredoxin-like) domain containing 1	PITHD1	1,26
Jeck2013 ANTISENSE, CDS, coding, INTERNAL, intronic, OVCODE, OVEXON best transcript NM_000089	COL1A2	1,26
glycerol-3-phosphate acyltransferase 4	GPAT4	1,26
chromosome 12 open reading frame 76	C12orf76	1,26

GrpE-like 2, mitochondrial (E. coli)	GRPEL2	1,26
uncharacterized protein ENSP00000383407-like; Transcript Identified by AceView; HCG1818297; Protein LOC388813 [Source:UniProtKB/TrEMBL;Acc:A0A087WSY0]	LOC388813; bafor; AF165138.7	1,26
keratin 10, type I	KRT10	1,26
pleckstrin and Sec7 domain containing 2	PSD2	1,26
trafficking protein particle complex 3-like	TRAPPC3L	1,26
sulfide quinone reductase-like (yeast)	SQRDL	1,26
G protein-coupled receptor 137B	GPR137B	1,26
leucine rich repeat containing 8 family, member B	LRRC8B	1,26
hematopoietic cell-specific Lyn substrate 1	HCLS1	1,26
tensin 1	TNS1	1,26
shisa family member 9	SHISA9	1,26
armadillo repeat containing, X-linked 1	ARMCX1	1,26
chromosome 1 open reading frame 54	C1orf54	1,26
polyhomeotic homolog 1 (Drosophila)	PHC1	1,26
fucosyltransferase 7 (alpha (1,3) fucosyltransferase)	FUT7	1,26
ecotropic viral integration site 2A; ecotropic viral integration site 2B	EVI2A; EVI2B	1,26
P antigen family, member 1 (prostate associated)	PAGE1	1,26
hemochromatosis type 2 (juvenile)	HFE2	1,26
RNA binding motif protein 44	RBM44	1,26
solute carrier family 24 (sodium/potassium/calcium exchanger), member 2	SLC24A2	1,26
hypermethylated in cancer 2	HIC2	1,26
activin A receptor type I	ACVR1	1,26
long intergenic non-protein coding RNA 663	LINC00663	1,26
von Willebrand factor A domain containing 3A	VWA3A	1,26
KIAA1024-like	KIAA1024L	1,26
microRNA 4738; H3 histone, family 3B (H3.3B)	MIR4738; H3F3B	1,26
cat eye syndrome chromosome region, candidate 1	CECR1	1,26
GIPC PDZ domain containing family, member 3	GIPC3	1,26
zinc finger protein 90	ZNF90	1,26
paraneoplastic Ma antigen family-like 2	PNMAL2	1,26
BH3 interacting domain death agonist	BID	1,26
VPS50 EARP/GARPII complex subunit	VPS50	1,26
chromosome 7 open reading frame 31	C7orf31	1,26
meteorin, glial cell differentiation regulator	METRN	1,26
fibroblast growth factor 4	FGF4	1,26
retinoic acid receptor, alpha	RARA	1,26
interferon, alpha 21	IFNA21	1,26
ZFP62 zinc finger protein	ZFP62	1,26
regulator of G-protein signaling 9	RGS9	1,26
testis-specific serine kinase 1B	TSSK1B	1,26

regenerating islet-derived 1 beta	REG1B	1,26
pyrin and HIN domain family, member 1	PYHIN1	1,26
protein phosphatase 1, regulatory subunit 17	PPP1R17	1,26
aquaporin 3 (Gill blood group)	AQP3	1,26
myosin, light chain 3, alkali; ventricular, skeletal, slow	MYL3	1,26
cyclin-dependent kinase inhibitor 1C (p57, Kip2)	CDKN1C	1,26
potassium channel tetramerization domain containing 1	KCTD1	1,26
Jeck2013 ALT_DONOR, coding, INTERNAL, intronic best transcript NM_152227	SNX5	1,26
follicular dendritic cell secreted protein	FDCSP	1,26
D site of albumin promoter (albumin D-box) binding protein	DBP	1,26
NIMA-related kinase 1	NEK1	1,26
C-type lectin domain family 4, member A	CLEC4A	1,26
adenylate cyclase activating polypeptide 1 (pituitary)	ADCYAP1	1,26
colony stimulating factor 2 receptor, beta, low-affinity (granulocyte-macrophage)	CSF2RB	1,25
long intergenic non-protein coding RNA 1342	LINC01342	1,25
calcium channel, voltage-dependent, L type, alpha 1D subunit	CACNA1D	1,25
zinc finger, BED-type containing 9	ZBED9	1,25
phosphodiesterase 10A	PDE10A	1,25
chloride channel, voltage-sensitive 4	CLCN4	1,25
homeobox A5	HOXA5	1,25
ring finger protein 148	RNF148	1,25
phosphatidylinositol-4-phosphate 3-kinase, catalytic subunit type 2 beta	PIK3C2B	1,25
potassium channel, inwardly rectifying subfamily J, member 5	KCNJ5	1,25
NADH dehydrogenase (ubiquinone) complex I, assembly factor 6	NDUFAF6	1,25
tumor protein D52-like 3	TPD52L3	1,25
adaptor-related protein complex 1 sigma 2 subunit	AP1S2	1,25
leucine rich repeat containing 16B	LRRC16B	1,25
ZXD family zinc finger C	ZXDC	1,25
katanin p80 (WD repeat containing) subunit B 1	KATNB1	1,25
glutamate receptor, ionotropic, AMPA 1	GRIA1	1,25
Transcript Identified by AceView, Entrez Gene ID(s) 9246	UBE2L6	1,25
lipase, family member K	LIPK	1,25
T brachyury transcription factor	T	1,25
Leber congenital amaurosis 5-like	LCA5L	1,25
Transcript Identified by AceView, Entrez Gene ID(s) 114876	OSBPL1A	1,25
bone morphogenetic protein 6	BMP6	1,25
ilvB (bacterial acetolactate synthase)-like	ILVBL	1,25
centrosomal protein 290kDa	CEP290	1,25
odorant binding protein 2B	OBP2B	1,25
chromosome 17 open reading frame 89	C17orf89	1,25
family with sequence similarity 205, member C	FAM205C	1,25
TBC1 domain family, member 28	TBC1D28	1,25

arginine and glutamate rich 1	ARGLU1	1,25
high mobility group nucleosome binding domain 5	HMGN5	1,25
glucosaminyl (N-acetyl) transferase 3, mucin type	GCNT3	1,25
coiled-coil domain containing 88B	CCDC88B	1,25
MER proto-oncogene, tyrosine kinase	MERTK	1,25
arrestin domain containing 3	ARRDC3	1,25
G-patch domain containing 4	GPATCH4	1,25
POTE ankyrin domain family, member B	POTEB	1,25
aquaporin 10	AQP10	1,25
zinc finger protein 641	ZNF641	1,25
ubiquitin specific peptidase 17-like family member 18; ubiquitin specific peptidase 17-like family member 11	USP17L18; USP17L11	1,25
phosphodiesterase 6C, cGMP-specific, cone, alpha prime	PDE6C	1,25
bridging integrator 3	BIN3	1,25
megalencephalic leukoencephalopathy with subcortical cysts 1	MLC1	1,25
CD80 molecule	CD80	1,25
FRMPD3 antisense RNA 1	FRMPD3-AS1	1,25
CD4 molecule	CD4	1,25
ATP binding cassette subfamily C member 9	ABCC9	1,25
forkhead-associated (FHA) phosphopeptide binding domain 1	FHAD1	1,25
CD5 molecule	CD5	1,25
spermatogenesis associated, serine-rich 1	SPATS1	1,25
matrix extracellular phosphoglycoprotein	MEPE	1,25
MMP25 antisense RNA 1	MMP25-AS1	1,25
dehydrogenase/reductase (SDR family) member 7C	DHRS7C	1,25
mitogen-activated protein kinase kinase kinase 15	MAP3K15	1,25
cAMP responsive element binding protein 3-like 3	CREB3L3	1,25
cysteine dioxygenase type 1	CDO1	1,25
cell division cycle associated 7	CDCA7	1,25
RIMS binding protein 2	RIMBP2	1,25
LRP2 binding protein	LRP2BP	1,25
family with sequence similarity 20, member A	FAM20A	1,25
Zhang2013 ALT_ACCEPTOR, ALT_DONOR, coding, INTERNAL, intronic best transcript NM_001146018	TJAP1	1,25
CXADR-like membrane protein	CLMP	1,25
ADAM metallopeptidase with thrombospondin type 1 motif 18	ADAMTS18	1,25
ZFP57 zinc finger protein	ZFP57	1,25
THAP domain containing 10	THAP10	1,25
Rho guanine nucleotide exchange factor 3	ARHGEF3	1,25
diacylglycerol kinase, delta 130kDa	DGKD	1,25
pitrilysin metallopeptidase 1	PITRM1	1,25
basic helix-loop-helix family, member e23	BHLHE23	1,25
thrombospondin type 1 domain containing 4	THSD4	1,25
leukocyte immunoglobulin-like receptor, subfamily A (with TM domain), member 6	LILRA6	1,25

amphiphysin	AMPH	1,25
striatin interacting protein 2	STRIP2	1,25
mucin 12, cell surface associated	MUC12	1,25
sperm-tail PG-rich repeat containing 1	STPG1	1,25
tuftelin interacting protein 11	TFIP11	1,25
solute carrier family 12 (potassium/chloride transporter), member 5	SLC12A5	1,25
actin filament associated protein 1	AFAP1	1,25
STX16-NPEPL1 readthrough (NMD candidate)	STX16-NPEPL1	1,25
TSC22 domain family, member 3	TSC22D3	1,25
Homo sapiens CD99 molecule (CD99), transcript variant 2, mRNA.; Homo sapiens CD99 molecule (CD99), transcript variant 3, mRNA.; Homo sapiens CD99 molecule (CD99), transcript variant 1, mRNA.; CD99 molecule [Source:HGNC Symbol;Acc:HGNC:7082]; Homo sapiens CD99 molecule, mRNA (cDNA clone MGC:2282 IMAGE:3161673), complete cds.; Homo sapiens CD99 molecule, mRNA (cDNA clone MGC:3938 IMAGE:2906143), complete cds.; Homo sapiens CD99 molecule, mRNA (cDNA clone MGC:19734 IMAGE:3606974), complete cds.; Homo sapiens CD99 molecule, mRNA (cDNA clone MGC:4214 IMAGE:2957883), complete cds.; Homo sapiens CD99 molecule, mRNA (cDNA clone MGC:14521 IMAGE:4094268), complete cds.; Salzman2013 ANNOTATED, CDS, coding, INTERNAL, OVCODE, OVEXON best transcript NM_002414; Salzman2013 ANNOTATED, CDS, coding, OVCODE, OVEXON, UTR3 best transcript NM_002414	CD99	1,25
upstream transcription factor 2, c-fos interacting	USF2	1,25
chromosome 10 open reading frame 107	C10orf107	1,25
histone deacetylase 7	HDAC7	1,25
calponin 1, basic, smooth muscle	CNN1	1,25
CUB domain containing protein 1	CDCP1	1,25
misato 1, mitochondrial distribution and morphology regulator; misato family member 2, pseudogene	MSTO1; MSTO2P	1,25
potassium channel, inwardly rectifying subfamily J, member 10	KCNJ10	1,25
chromosome 11 open reading frame 73	C11orf73	1,25
pregnancy specific beta-1-glycoprotein 1	PSG1	1,25
one cut homeobox 3	ONECUT3	1,25
MAGEA10-MAGEA5 readthrough; novel transcript	MAGEA10-MAGEA5; RP11-1007I13.4	1,25
	LRIG1	1,25
ribonuclease P/MRP 25kDa subunit-like	RPP25L	1,25
keratin associated protein 21-3	KRTAP21-3	1,25
S-antigen; retina and pineal gland (arrestin)	SAG	1,25
centrosomal protein 89kDa	CEP89	1,25

heat shock 70kDa protein 12A	HSPA12A	1,25
ribosomal protein L10-like	RPL10L	1,25
immediate early response 2	IER2	1,25
FYVE, RhoGEF and PH domain containing 6	FGD6	1,25
fibroblast growth factor receptor substrate 3	FRS3	1,25
G antigen 2D; G antigen 13; G antigen 2E; G antigen 8; G antigen 2B; G antigen 2A; G antigen 2C	GAGE2D; GAGE13; GAGE2E; GAGE8; GAGE2B; GAGE2A; GAGE2C	1,25
keratin 38, type I	KRT38	1,25
zinc finger protein 687	ZNF687	1,25
oncostatin M	OSM	1,25
ring finger protein 135	RNF135	1,25
zinc finger protein 556	ZNF556	1,25
sodium channel modifier 1; tumor necrosis factor, alpha-induced protein 8-like 2	SCNM1; TNFAIP8L2	1,25
ankyrin repeat domain 36	ANKRD36	1,25
VIM antisense RNA 1	VIM-AS1	1,25
angiopoietin like 5	ANGPTL5	1,25
Memczak2013 ALT_ACCEPTOR, ALT_DONOR, coding, INTERNAL, intronic best transcript NM_001006634	ARHGAP17	1,25
histidyl-tRNA synthetase 2, mitochondrial	HARS2	1,25
TNF receptor-associated factor 6, E3 ubiquitin protein ligase	TRAF6	1,25
intersectin 2	ITSN2	1,25
growth factor receptor bound protein 10	GRB10	1,25
crystallin lambda 1	CRYL1	1,25
ficolin (collagen/fibrinogen domain containing lectin) 2	FCN2	1,25
peroxisomal biogenesis factor 11 alpha	PEX11A	1,25
chromosome 9 open reading frame 114	C9orf114	1,25
receptor (G protein-coupled) activity modifying protein 1	RAMP1	1,25
cancer/testis antigen family 45, member A5	CT45A5	1,25
carbonic anhydrase X	CA10	1,25
RAB6C, member RAS oncogene family	RAB6C	1,25
poly(A) binding protein, cytoplasmic 1-like	PABPC1L	1,25
acyl-CoA binding domain containing 7	ACBD7	1,25
microtubule associated protein 6	MAP6	1,25
pseudopodium-enriched atypical kinase 1	PEAK1	1,25
chromosome X open reading frame 65	CXorf65	1,25
CHRNA7 (cholinergic receptor, nicotinic, alpha 7, exons 5-10) and FAM7A (family with sequence similarity 7A, exons A-E) fusion	CHRFAM7A	1,25
zinc finger protein 165	ZNF165	1,25

TraB domain containing 2B	TRABD2B	1,25
homeobox D3	HOXD3	1,25
ADP-ribosylation factor like GTPase 4C	ARL4C	1,25
arginase 2	ARG2	1,25
RNA binding motif protein 15B	RBM15B	1,25
TM4SF19-TCTEX1D2 readthrough (NMD candidate)	TM4SF19-TCTEX1D2	1,25
cylindromatosis (turban tumor syndrome)	CYLD	1,25
complement factor H-related 4	CFHR4	1,25
polymerase (RNA) mitochondrial (DNA directed)	POLRMT	1,25
GATA binding protein 3	GATA3	1,25
coagulation factor II (thrombin) receptor-like 2	F2RL2	1,24
cation channel, sperm associated 2	CATSPER2	1,24
TatD DNase domain containing 2; ghrelin opposite strand/antisense RNA	TATDN2; GHRLOS	1,24
KRAB-A domain containing 1	KRBA1	1,24
Memczak2013 ALT_ACCEPTOR, ALT_DONOR, coding, INTERNAL, intronic best transcript NM_033071	SYNE1	1,24
Memczak2013 ALT_ACCEPTOR, ALT_DONOR, coding, INTERNAL, intronic best transcript NM_001098207	HNRNPF	1,24
ectonucleotide pyrophosphatase/phosphodiesterase 2	ENPP2	1,24
ubiquitin specific peptidase 51	USP51	1,24
inositol polyphosphate-5-phosphatase D	INPP5D	1,24
CD14 molecule	CD14	1,24
poly(A) binding protein interacting protein 2B	PAIP2B	1,24
neurobeachin	NBEA	1,24
family with sequence similarity 118, member A	FAM118A	1,24
junctional adhesion molecule 2	JAM2	1,24
ribosomal protein L3-like	RPL3L	1,24
microfibrillar associated protein 5	MFAP5	1,24
glypican 2	GPC2	1,24
chromosome 12 open reading frame 50	C12orf50	1,24
dynein, axonemal, heavy chain 2	DNAH2	1,24
vascular endothelial growth factor A	VEGFA	1,24
unc-13 homolog B (C. elegans)	UNC13B	1,24
BRCA1 associated protein	BRAP	1,24
NADH dehydrogenase (ubiquinone) 1 alpha subcomplex, 12	NDUFA12	1,24
Mov10 RISC complex RNA helicase like 1	MOV10L1	1,24
sec1 family domain containing 2	SCFD2	1,24
kelch-like family member 25	KLHL25	1,24
zinc finger protein 546	ZNF546	1,24
N-acetylneuraminic acid synthase	NANS	1,24
PIF1 5-to-3 DNA helicase	PIF1	1,24
MAGE family member A10	MAGEA10	1,24
chromosome 15 open reading frame 39	C15orf39	1,24

RAD17 checkpoint clamp loader component	RAD17	1,24
GLI pathogenesis-related 2	GLIPR2	1,24
HemK methyltransferase family member 1	HEMK1	1,24
family with sequence similarity 173, member B	FAM173B	1,24
chemokine (C-X-C motif) ligand 14	CXCL14	1,24
late cornified envelope 3E	LCE3E	1,24
Shwachman-Bodian-Diamond syndrome	SBDS	1,24
solute carrier family 1 (glutamate/neutral amino acid transporter), member 4	SLC1A4	1,24
chromosome 1 open reading frame 194	C1orf194	1,24
mucin 21, cell surface associated	MUC21	1,24
ribose 5-phosphate isomerase A	RPIA	1,24
olfactory receptor, family 10, subfamily Q, member 1	OR10Q1	1,24
zinc finger protein 551	ZNF551	1,24
family with sequence similarity 65, member B	FAM65B	1,24
DENN/MADD domain containing 1A	DENND1A	1,24
NHS-like 1	NHSL1	1,24
psoriasis susceptibility 1 candidate 2	PSORS1C2	1,24
ring finger protein 113A	RNF113A	1,24
MPL proto-oncogene, thrombopoietin receptor	MPL	1,24
IQ motif and Sec7 domain 3	IQSEC3	1,24
rotatin	RTTN	1,24
PITPNM family member 3	PITPNM3	1,24
cadherin-related family member 1	CDHR1	1,24
G-protein signaling modulator 2	GPSM2	1,24
SPATA31 subfamily D, member 4	SPATA31D4	1,24
oral cancer overexpressed 1	ORAOV1	1,24
adenosine monophosphate deaminase 1	AMPD1	1,24
homer scaffolding protein 3	HOMER3	1,24
3-hydroxymethyl-3-methylglutaryl-CoA lyase-like 1	HMGCLL1	1,24
G-patch domain containing 3	GPATCH3	1,24
gap junction protein beta 5	GJB5	1,24
ataxia, cerebellar, Cayman type	ATCAY	1,24
two pore segment channel 2	TPCN2	1,24
armadillo repeat containing 4	ARMC4	1,24
cilia and flagella associated protein 52	CFAP52	1,24
iduronidase, alpha-L-	IDUA	1,24
ABRA C-terminal like	ABRACL	1,24
coiled-coil domain containing 38	CCDC38	1,24
leucine rich repeat and fibronectin type III domain containing 3	LRFN3	1,24
cyclin D2	CCND2	1,24
StAR-related lipid transfer domain containing 10	STARD10	1,24
major intrinsic protein of lens fiber	MIP	1,24
secretogranin V	SCG5	1,24
PRAME family member 8	PRAMEF8	1,24

interleukin 7 receptor	IL7R	1,24
leucine, glutamate and lysine rich 1	LEKR1	1,24
ArfGAP with dual PH domains 2	ADAP2	1,24
aspartate beta-hydroxylase domain containing 2	ASPHD2	1,24
keratin 25, type I	KRT25	1,24
decapping exoribonuclease	DXO	1,24
leucine rich repeat (in FLII) interacting protein 2	LRRFIP2	1,24
glycogenin 1	GYG1	1,24
PHD finger protein 12	PHF12	1,24
ovochymase 2 (gene/pseudogene)	OVCH2	1,24
olfactory receptor, family 1, subfamily D, member 4 (gene/pseudogene)	OR1D4	1,24
olfactory receptor, family 4, subfamily C, member 45	OR4C45	1,24
sulfite oxidase	SUOX	1,24
arginine-fifty homeobox	ARGFX	1,24
crystallin beta A2	CRYBA2	1,24
ankyrin repeat domain 57 pseudogene	LOC389834; AL354822.1	1,24
transglutaminase 4	TGM4	1,24
sema domain, immunoglobulin domain (Ig), short basic domain, secreted, (semaphorin) 3G	SEMA3G	1,24
Transcript Identified by AceView, Entrez Gene ID(s) 285074; 5343	PLGLB1	1,24
cubilin (intrinsic factor-cobalamin receptor)	CUBN	1,24
mucin 20, cell surface associated; succinate dehydrogenase complex subunit A, flavoprotein pseudogene 2; microRNA 570; long intergenic non-protein coding RNA 969	MUC20; SDHAP2; MIR570; LINC00969	1,24
glutathione S-transferase alpha 5	GSTA5	1,24
cancer/testis antigen family 45, member A3; cancer/testis antigen family 45, member A4; cancer/testis antigen family 45, member A5	CT45A3; CT45A4; CT45A5	1,24
canopy FGF signaling regulator 1	CNPY1	1,24
FSHD region gene 2 family, member C	FRG2C	1,24
haloacid dehalogenase-like hydrolase domain containing 3	HDHD3	1,24
neutrophil cytosolic factor 1	NCF1	1,24
mesoderm induction early response 1, family member 2	MIER2	1,24
cerebellin 3 precursor	CBLN3	1,24
golgin A8 family, member B	GOLGA8B	1,24
secretagogin, EF-hand calcium binding protein	SCGN	1,24
novel transcript; Transcript Identified by AceView, Entrez Gene ID(s) 389432	RP11-307P5.1; SAMD5	1,24
phosphoribosyl pyrophosphate synthetase-associated protein 2	PRPSAP2	1,24
beclin 2	BECN2	1,24
shroom family member 1	SHROOM1	1,24

tubulin, alpha 4b	TUBA4B	1,24
WD repeat domain 86	WDR86	1,24
formin 1	FMN1	1,24
Norrie disease (pseudoglioma)	NDP	1,24
Rho GTPase activating protein 42	ARHGAP42	1,24
olfactory receptor, family 10, subfamily A, member 2	OR10A2	1,24
short chain dehydrogenase/reductase family 42E, member 1	SDR42E1	1,24
A kinase (PRKA) anchor protein 13; microRNA 7706	AKAP13; MIR7706	1,24
uncharacterized LOC101928327; novel transcript	LOC101928327; AC007557.1	1,24
codanin 1	CDAN1	1,24
glutaminase 2 (liver, mitochondrial)	GLS2	1,24
F-box and WD repeat domain containing 7, E3 ubiquitin protein ligase	FBXW7	1,24
TNFAIP3 interacting protein 2	TNIP2	1,24
CD300e molecule	CD300E	1,24
NLR family, apoptosis inhibitory protein	NAIP	1,24
DEAD (Asp-Glu-Ala-Asp) box helicase 24	DDX24	1,24
uncharacterized LOC100130451; novel transcript (LOC100130451); novel transcript	LOC100130451; AC079610.2; RP11-105N14.2	1,24
flavin containing monooxygenase 1	FMO1	1,24
SPATA31 subfamily A, member 5; SPATA31 subfamily A, member 7	SPATA31A5; SPATA31A7	1,23
odorant binding protein 2B	OBP2B	1,23
Transcript Identified by AceView, Entrez Gene ID(s) 8220	DGCR14	1,23
C-type lectin domain family 7, member A	CLEC7A	1,23
NADPH oxidase 3	NOX3	1,23
tetraspanin 19	TSPAN19	1,23
solute carrier family 6 (neurotransmitter transporter), member 13	SLC6A13	1,23
acyl-CoA synthetase short-chain family member 1	ACSS1	1,23
INO80 complex subunit E	INO80E	1,23
ATP binding cassette subfamily C member 8	ABCC8	1,23
RAR-related orphan receptor B	RORB	1,23
chromosome 8 open reading frame 87	C8orf87	1,23
slingshot protein phosphatase 3	SSH3	1,23
VPS11, CORVET/HOPS core subunit [Source:HGNC Symbol;Acc:HGNC:14583]	VPS11	1,23
ectonucleotide pyrophosphatase/phosphodiesterase 2	ENPP2	1,23
Transcript Identified by AceView, Entrez Gene ID(s) 55222	LRRC20	1,23
vitamin D (1,25- dihydroxyvitamin D3) receptor	VDR	1,23
Src-like-adaptor	SLA	1,23

zinc finger protein 577	ZNF577	1,23
zinc finger protein 280D	ZNF280D	1,23
attractin-like 1	ATRNL1	1,23
MANSC domain containing 1	MANSC1	1,23
MLX interacting protein	MLXIP	1,23
intermediate filament family orphan 2	IFFO2	1,23
cytochrome b5 domain containing 2	CYB5D2	1,23
N-acylsphingosine amidohydrolase (non-lysosomal ceramidase) 2	ASAH2	1,23
zinc finger protein 780A	ZNF780A	1,23
FAT atypical cadherin 3	FAT3	1,23
testis expressed 38	TEX38	1,23
SEC16 homolog B, endoplasmic reticulum export factor	SEC16B	1,23
UBA domain containing 1	UBAC1	1,23
zinc finger protein 501	ZNF501	1,23
family with sequence similarity 218, member A	FAM218A	1,23
keratin 79, type II	KRT79	1,23
mediator complex subunit 21	MED21	1,23
ring finger protein 25	RNF25	1,23
glutamyl-prolyl-tRNA synthetase	EPRS	1,23
piggyBac transposable element derived 1	PGBD1	1,23
protein phosphatase 1, regulatory subunit 26	PPP1R26	1,23
cAMP responsive element binding protein 5	CREB5	1,23
brain protein I3	BRI3	1,23
methylenetetrahydrofolate dehydrogenase (NADP+ dependent) 1-like	MTHFD1L	1,23
ATP binding cassette subfamily C member 4	ABCC4	1,23
poly(rC) binding protein 3	PCBP3	1,23
T-cell leukemia homeobox 2	TLX2	1,23
transmembrane protein 47	TMEM47	1,23
zinc finger protein 133	ZNF133	1,23
sodium channel, voltage gated, type VIII alpha subunit	SCN8A	1,23
nanos homolog 2 (Drosophila)	NANOS2	1,23
wingless-type MMTV integration site family, member 5B	WNT5B	1,23
G protein-coupled receptor 17	GPR17	1,23
pregnancy up-regulated nonubiquitous CaM kinase	PNCK	1,23
keratin 7, type II	KRT7	1,23
potassium channel, two pore domain subfamily K, member 3	KCNK3	1,23
transient receptor potential cation channel, subfamily M, member 4	TRPM4	1,23
Transcript Identified by AceView, Entrez Gene ID(s) 6596	HLTF	1,23
Transcript Identified by AceView, Entrez Gene ID(s) 8878	SQSTM1	1,23
zinc finger, CCCH-type with G-patch domain; Lck interacting transmembrane adaptor 1	ZGPAT; LIME1	1,23
spindle and kinetochore associated complex subunit 1	SKA1	1,23

ATPase family, AAA domain containing 3B	ATAD3B	1,23
S-antigen; retina and pineal gland (arrestin)	SAG	1,23
chromosome 11 open reading frame 16	C11orf16	1,23
HERV-H LTR-associating 2	HHLA2	1,23
DPH2 homolog	DPH2	1,23
Memczak2013 ALT_ACCEPTOR, ALT_DONOR, coding, INTERNAL, intronic best transcript NM_001163435	TBCK	1,23
keratin 73, type II	KRT73	1,23
potassium channel subfamily M regulatory beta subunit 4	KCNMB4	1,23
CD9 molecule	CD9	1,23
deleted in primary ciliary dyskinesia homolog (mouse)	DPCD	1,23
family with sequence similarity 227, member B	FAM227B	1,23
ligand of numb-protein X 1, E3 ubiquitin protein ligase	LNX1	1,23
homeobox A2	HOXA2	1,23
PR domain containing 2, with ZNF domain	PRDM2	1,23
actin-like 10	ACTL10	1,23
coiled-coil domain containing 80; long intergenic non-protein coding RNA 1279	CCDC80; LINC01279	1,23
Memczak2013 ANTISENSE, coding, INTERNAL, intronic best transcript NM_001166373	MARCH1	1,23
Ras and Rab interactor like	RINL	1,23
very low density lipoprotein receptor	VLDLR	1,23
ATPase, Ca++ transporting, cardiac muscle, fast twitch 1	ATP2A1	1,23
salt-inducible kinase 1	SIK1	1,23
autophagy related 16-like 1	ATG16L1	1,23
Ras association (RalGDS/AF-6) domain family member 3	RASSF3	1,23
VPS11, CORVET/HOPS core subunit [Source:HGNC Symbol;Acc:HGNC:14583]	VPS11	1,23
matrix metallopeptidase 14 (membrane-inserted)	MMP14	1,23
tumor necrosis factor (ligand) superfamily, member 11	TNFSF11	1,23
fibroblast growth factor 2 (basic)	FGF2	1,23
POU class 3 homeobox 1	POU3F1	1,23
G protein-coupled receptor 55	GPR55	1,23
Memczak2013 ANTISENSE, coding, INTERNAL, intronic best transcript NM_181985	LILRA5	1,23
inositol-trisphosphate 3-kinase B	ITPKB	1,23
tubulin tyrosine ligase-like family member 8	TTLL8	1,23
biorientation of chromosomes in cell division 1-like 2	BOD1L2	1,23
zinc finger protein 548	ZNF548	1,23
single-stranded DNA binding protein 2	SSBP2	1,23
growth arrest-specific 2 like 2	GAS2L2	1,23
zinc finger protein 711	ZNF711	1,23
zinc finger protein 501 [Source:HGNC Symbol;Acc:HGNC:23717]	ZNF501	1,23
chemokine (C-C motif) ligand 17	CCL17	1,23
allograft inflammatory factor 1-like	AIF1L	1,23

protease, serine, 8	PRSS8	1,23
5-nucleotidase domain containing 4	NT5DC4	1,23
coiled-coil domain containing 159	CCDC159	1,23
Jeck2013 ALT_ACCEPTOR, ALT_DONOR, coding, INTERNAL, intronic best transcript NM_005270	GLI2	1,23
WD repeat domain 97	WDR97	1,23
synovial sarcoma, X breakpoint 8	SSX8	1,23
grainyhead-like transcription factor 3	GRHL3	1,23
exocyst complex component 3-like 2	EXOC3L2	1,23
ZPR1 zinc finger	ZPR1	1,23
family with sequence similarity 174, member B	FAM174B	1,23
GTF2I repeat domain containing 1	GTF2IRD1	1,23
collagen, type IX, alpha 1	COL9A1	1,23
Transcript Identified by AceView, Entrez Gene ID(s) 49855	SCAPER	1,23
chemokine (C-C motif) ligand 28	CCL28	1,23
tubulin tyrosine ligase-like family member 7	TTLL7	1,23
solute carrier family 6 (neurotransmitter transporter, glycine), member 9	SLC6A9	1,23
rhomboid, veinlet-like 3 (Drosophila)	RHBDL3	1,23
AXL receptor tyrosine kinase	AXL	1,23
protein phosphatase 1, regulatory subunit 10	PPP1R10	1,23
endothelin receptor type A	EDNRA	1,23
RPARP antisense RNA 1	RPARP-AS1	1,23
ATP binding cassette subfamily A member 10	ABCA10	1,23
uncharacterized LOC100130370; novel transcript	LOC100130370; RP11-1055B8.3	1,23
chemokine (C-X-C motif) ligand 3	CXCL3	1,23
engulfment and cell motility 3	ELMO3	1,23
zinc finger with KRAB and SCAN domains 7	ZKSCAN7	1,23
alanyl-tRNA synthetase	AARS	1,23
zinc finger protein 550	ZNF550	1,23
signal-regulatory protein beta 1	SIRPB1	1,23
LY6/PLAUR domain containing 1	LYPD1	1,23
polypeptide N-acetylgalactosaminyltransferase 18	GALNT18	1,23
Transcript Identified by AceView, Entrez Gene ID(s) 84954; novel transcript, antisense to STAP2	AC007292.7; MPND	1,23
LSM10, U7 small nuclear RNA associated	LSM10	1,23
Transcript Identified by AceView, Entrez Gene ID(s) 26147	PHF19	1,23
cyclin-dependent kinase 2 interacting protein	CINP	1,23
otoconin 90	OC90	1,23
glutaredoxin, cysteine rich 1	GRXCR1	1,23
Rho guanine nucleotide exchange factor 11	ARHGEF11	1,23
family with sequence similarity 167, member B	FAM167B	1,23
Transcript Identified by AceView, Entrez Gene ID(s) 6660	SOX5	1,23

glycoprotein 2 (zymogen granule membrane)	GP2	1,23
pre-B-cell leukemia homeobox 1	PBX1	1,23
leucine-rich repeats and transmembrane domains 2	LRTM2	1,23
Myb/SANT-like DNA-binding domain containing 2	MSANTD2	1,23
centromere protein M	CENPM	1,23
zinc finger protein 485	ZNF485	1,23
GTPase, IMAP family member 8	GIMAP8	1,23
ripply transcriptional repressor 3	RIPPLY3	1,23
zinc finger and BTB domain containing 3	ZBTB3	1,23
Transcript Identified by AceView, Entrez Gene ID(s) 7265	TTC1	1,23
phospholipase C-like 2; microRNA 3714	PLCL2; MIR3714	1,23
tripartite motif containing 25; microRNA 3614	TRIM25; MIR3614	1,23
Rho GTPase activating protein 36	ARHGAP36	1,23
activator of basal transcription 1	ABT1	1,23
solute carrier family 48 (heme transporter), member 1	SLC48A1	1,23
CASK interacting protein 1	CASKIN1	1,23
ankyrin repeat domain 39	ANKRD39	1,23
potassium channel, two pore domain subfamily K, member 2	KCNK2	1,23
zinc finger protein 662	ZNF662	1,23
BTB (POZ) domain containing 10	BTBD10	1,23
S100 calcium binding protein A2	S100A2	1,23
chromosome 1 open reading frame 168	C1orf168	1,23
KIAA1644	KIAA1644	1,23
olfactory receptor, family 2, subfamily B, member 6	OR2B6	1,23
Meis homeobox 2	MEIS2	1,23
ATPase type 13A4	ATP13A4	1,23
transmembrane protein 100	TMEM100	1,23
zinc finger protein 878	ZNF878	1,23
ribonuclease, RNase A family, 7	RNASE7	1,23
GRIK1 antisense RNA 2; BTB and CNC homology 1, basic leucine zipper transcription factor 1	GRIK1-AS2; BACH1	1,23
thiosulfate sulfurtransferase (rhodanese)-like domain containing 3	TSTD3	1,23
metallophosphoesterase domain containing 2	MPPED2	1,23
chromodomain protein, Y-linked, 2B; chromodomain protein, Y-linked, 2A	CDY2B; CDY2A	1,23
transmembrane protein 120B	TMEM120B	1,23
transcription elongation factor A (SII) N-terminal and central domain containing	TCEANC	1,23
Transcript Identified by AceView, Entrez Gene ID(s) 1997; 100128628	ELF1	1,22
autophagy related 16-like 1	ATG16L1	1,22
olfactory receptor, family 5, subfamily P, member 2	OR5P2	1,22

chemokine (C-X-C motif) receptor 3	CXCR3	1,22
T-box 4	TBX4	1,22
PYD and CARD domain containing	PYCARD	1,22
transmembrane protein 42	TMEM42	1,22
uncharacterized LOC102723701; novel transcript, antisense to ERLIN2	LOC102723701; RP11-863K10.7	1,22
mitogen-activated protein kinase kinase kinase 12	MAP3K12	1,22
gastric inhibitory polypeptide	GIP	1,22
family with sequence similarity 27, member E3	FAM27E3	1,22
AT rich interactive domain 5A (MRF1-like)	ARID5A	1,22
long intergenic non-protein coding RNA 598	LINC00598	1,22
thyroid hormone receptor interactor 11	TRIP11	1,22
Memczak2013 ALT_ACCEPTOR, ALT_DONOR, coding, INTERNAL, intronic best transcript NM_015032	PDS5B	1,22
ring finger protein 32	RNF32	1,22
proenkephalin	PENK	1,22
epoxide hydrolase 4	EPHX4	1,22
complement component 5a receptor 2	C5AR2	1,22
chimerin 1	CHN1	1,22
AE binding protein 1; microRNA 4649	AEBP1; MIR4649	1,22
chromosome 12 open reading frame 45	C12orf45	1,22
peroxisomal biogenesis factor 2	PEX2	1,22
coactivator-associated arginine methyltransferase 1	CARM1	1,22
brain and reproductive organ-expressed (TNFRSF1A modulator)	BRE	1,22
growth arrest-specific 8	GAS8	1,22
mitogen-activated protein kinase 8	MAPK8	1,22
coiled-coil domain containing 79	CCDC79	1,22
uncharacterized LOC100131496; novel transcript, antisense to ZMYND8	LOC100131496; RP4-569M23.5	1,22
papilin, proteoglycan-like sulfated glycoprotein	PAPLN	1,22
oculocerebrorenal syndrome of Lowe	OCRL	1,22
coiled-coil domain containing 84	CCDC84	1,22
kinesin family member C2	KIFC2	1,22
Jeck2013 ALT_ACCEPTOR, ALT_DONOR, coding, INTERNAL, intronic best transcript NM_012316	KPNA6	1,22
arachidonate 15-lipoxygenase, type B	ALOX15B	1,22
G antigen 4; G antigen 7; G antigen 12G	GAGE4; GAGE7; GAGE12G	1,22
MTOR associated protein, LST8 homolog	MLST8	1,22
optic atrophy 3 (autosomal recessive, with chorea and spastic paraplegia)	OPA3	1,22
protein kinase, Y-linked, pseudogene	PRKY	1,22

adrenergic, beta, receptor kinase 1	ADRBK1	1,22
RAB44, member RAS oncogene family	RAB44	1,22
tripeptidyl peptidase II	TPP2	1,22
coiled-coil domain containing 74B	CCDC74B	1,22
calcium/calmodulin-dependent protein kinase II gamma	CAMK2G	1,22
SHQ1, H/ACA ribonucleoprotein assembly factor	SHQ1	1,22
taste receptor, type 2, member 9	TAS2R9	1,22
cornifelin	CNFN	1,22
uncharacterized LOC100130449; novel transcript, antisense to GPC1; Transcript Identified by AceView, Entrez Gene ID(s) 100130449; uncharacterized LOC100130449 [Source:EntrezGene;Acc:100130449]	PP14571; AC110619.2	1,22
PRAME family member 25	PRAMEF25	1,22
signal transducer and activator of transcription 5B	STAT5B	1,22
cytochrome P450, family 3, subfamily A, polypeptide 7; CYP3A7-CYP3A51P readthrough	CYP3A7; CYP3A7-CYP3A51P	1,22
Zic family member 1	ZIC1	1,22
NK1 homeobox 1	NKX1-1	1,22
TRHDE antisense RNA 1	TRHDE-AS1	1,22
tryptophan rich basic protein	WRB	1,22
Memczak2013 ANTISENSE, coding, intronic, upstream_start, UTR5 best transcript NM_001161357	FCHO1	1,22
R3H domain and coiled-coil containing 1	R3HCC1	1,22
membrane associated ring finger 1	MARCH1	1,22
carbonyl reductase 1	CBR1	1,22
family with sequence similarity 114, member A2	FAM114A2	1,22
GATA binding protein 6	GATA6	1,22
cytochrome P450, family 2, subfamily D, polypeptide 6	CYP2D6	1,22
defensin, beta 107A; defensin, beta 107B	DEFB107A; DEFB107B	1,22
ATPase, H+ transporting, lysosomal 50/57kDa, V1 subunit H	ATP6V1H	1,22
autophagy related 16-like 1	ATG16L1	1,22
cartilage intermediate layer protein, nucleotide pyrophosphohydrolase	CILP	1,22
guanosine monophosphate reductase	GMPR	1,22
centrosomal protein 164kDa	CEP164	1,22
olfactory receptor, family 4, subfamily A, member 15	OR4A15	1,22
peptidyl arginine deiminase, type IV	PADI4	1,22
solute carrier family 47 (multidrug and toxin extrusion), member 2	SLC47A2	1,22
regulating synaptic membrane exocytosis 4	RIMS4	1,22
serum/glucocorticoid regulated kinase family, member 3	SGK3	1,22
CKLF-like MARVEL transmembrane domain containing 7	CMTM7	1,22
olfactory receptor, family 4, subfamily M, member 2	OR4M2	1,22

prostaglandin E synthase	PTGES	1,22
lymphatic vessel endothelial hyaluronan receptor 1	LYVE1	1,22
double homeobox 4	DUX4	1,22
proline rich Gla (G-carboxyglutamic acid) 4 (transmembrane)	PRRG4	1,22
nuclear receptor binding protein 2; microRNA 6845	NRBP2; MIR6845	1,22
branched chain keto acid dehydrogenase E1, alpha polypeptide	BCKDHA	1,22
solute carrier family 34 (type II sodium/phosphate cotransporter), member 3	SLC34A3	1,22
adenylate cyclase activating polypeptide 1 (pituitary) receptor type I	ADCYAP1R1	1,22
potassium channel, inwardly rectifying subfamily J, member 2	KCNJ2	1,22
DEP domain containing 7	DEPDC7	1,22
kallikrein related peptidase 7	KLK7	1,22
cell division cycle 14A	CDC14A	1,22
left-right determination factor 2	LEFTY2	1,22
basic helix-loop-helix family, member a15	BHLHA15	1,22
zinc finger protein 14	ZNF14	1,22
radical S-adenosyl methionine domain containing 1	RSAD1	1,22
pleiotrophin	PTN	1,22
adaptor-related protein complex 1, sigma 2 subunit pseudogene	LOC653653; AC025048.1	1,22
centrosomal protein 170B	CEP170B	1,22
RNA binding motif protein 5	RBM5	1,22
SPATA31 subfamily D, member 3	SPATA31D3	1,22
dorsal inhibitory axon guidance protein	DRAXIN	1,22
keratin 77, type II	KRT77	1,22
sialic acid acetylesterase	SIAE	1,22
chromosome 12 open reading frame 75	C12orf75	1,22
ligase I, DNA, ATP-dependent	LIG1	1,22
Memczak2013 ANTISENSE, CDS, coding, INTERNAL best transcript NM_002087	GRN	1,22
neuropilin 1	NRP1	1,22
myosin binding protein H-like	MYBPHL	1,22
kinesin family member 2B	KIF2B	1,22
queuine tRNA-ribosyltransferase domain containing 1	QTRTD1	1,22
glycoprotein IX (platelet)	GP9	1,22
arrestin domain containing 4	ARRDC4	1,22
spermatogenesis and oogenesis specific basic helix-loop-helix 1	SOHLH1	1,22
geminin coiled-coil domain containing	GMNC	1,22
taste receptor, type 2, member 30; taste receptor, type 2, member 43; proline rich 4 (lacrimal)	TAS2R30; TAS2R43; PRR4	1,22
lacritin	LACRT	1,22
polypeptide N-acetylgalactosaminyltransferase 6	GALNT6	1,22

STE20-like kinase	SLK	1,22
WAS/WASL interacting protein family, member 3	WIPF3	1,22
dickkopf WNT signaling pathway inhibitor 4	DKK4	1,22
zinc finger protein 780A	ZNF780A	1,22
UDP-Gal:betaGlcNAc beta 1,4- galactosyltransferase, polypeptide 5	B4GALT5	1,22
chemokine (C-C motif) ligand 5	CCL5	1,22
zinc finger protein 501 [Source:HGNC Symbol;Acc:HGNC:23717]	ZNF501	1,22
apolipoprotein B mRNA editing enzyme, catalytic polypeptide-like 3A	APOBEC3A	1,22
sigma non-opioid intracellular receptor 1	SIGMAR1	1,22
Memczak2013 ALT_ACCEPTOR, ALT_DONOR, coding, INTERNAL, intronic best transcript NM_022720	DGCR8	1,22
cyclin-dependent kinase 11A	CDK11A	1,22
ADAM metallopeptidase with thrombospondin type 1 motif 20	ADAMTS20	1,22
solute carrier family 45, member 2	SLC45A2	1,22
NFS1 cysteine desulfurase	NFS1	1,22
nuclear VCP-like	NVL	1,22
YTH N(6)-methyladenosine RNA binding protein 1	YTHDF1	1,22
xylosyltransferase II	XYLT2	1,22
EFR3 homolog A	EFR3A	1,22
mucin 7, secreted	MUC7	1,22
potassium channel, voltage gated modifier subfamily V, member 2	KCNV2	1,22
leucine rich repeat containing 14	LRRC14	1,22
TSPY-like 4	TSPYL4	1,22
BAH domain and coiled-coil containing 1	BAHCC1	1,22
coiled-coil domain containing 102B	CCDC102B	1,22
tetraspanin 18	TSPAN18	1,22
NPHP3-ACAD11 readthrough (NMD candidate)	NPHP3-ACAD11	1,22
zinc finger protein 546	ZNF546	1,22
annexin A3	ANXA3	1,22
NADH dehydrogenase (ubiquinone) 1 alpha subcomplex, 3, 9kDa	NDUFA3	1,22
inositol 1,4,5-trisphosphate receptor, type 2	ITPR2	1,22
seizure threshold 2 homolog (mouse)	SZT2	1,22
zinc finger protein 804A	ZNF804A	1,22
Transcript Identified by AceView, Entrez Gene ID(s) 2098	ESD	1,22
NADH dehydrogenase (ubiquinone) 1 alpha subcomplex, 10, 42kDa	NDUFA10	1,22
taste receptor, type 1, member 1	TAS1R1	1,22
proline rich, lacrimal 1	PROL1	1,21
organic solute carrier partner 1	OSCP1	1,21
MAP/microtubule affinity-regulating kinase 3	MARK3	1,21

G antigen 12H; G antigen 12B; G antigen 12C	GAGE12H; GAGE12B; GAGE12C	1,21
G antigen 12D	GAGE12D	1,21
G antigen 12G; G antigen 12E	GAGE12G; GAGE12E	1,21
chromosome 9 open reading frame 153	C9orf153	1,21
SAM domain, SH3 domain and nuclear localization signals 1	SAMSN1	1,21
carboxypeptidase M	CPM	1,21
family with sequence similarity 25, member A	FAM25A	1,21
clarin 2	CLRN2	1,21
zinc finger protein 235	ZNF235	1,21
glutathione reductase	GSR	1,21
TSSK6 activating co-chaperone	TSACC	1,21
CD22 molecule; microRNA 5196	CD22; MIR5196	1,21
KLRC4-KLRK1 readthrough; killer cell lectin-like receptor subfamily K, member 1; killer cell lectin-like receptor subfamily C, member 4	KLRC4-KLRK1; KLRK1; KLRC4	1,21
chromosome X open reading frame 56	CXorf56	1,21
neuregulin 1	NRG1	1,21
deiodinase, iodothyronine, type II	DIO2	1,21
zinc finger protein 805	ZNF805	1,21
carbohydrate (N-acetylgalactosamine 4-0) sulfotransferase 14	CHST14	1,21
Transcript Identified by AceView, Entrez Gene ID(s) 8939	FUBP3	1,21
BARX homeobox 2	BARX2	1,21
methylenetetrahydrofolate dehydrogenase (NADP+ dependent) 2-like	MTHFD2L	1,21
prostate cancer susceptibility candidate 1	PRAC1	1,21
ATP binding cassette subfamily C member 11	ABCC11	1,21
ataxin 1-like	ATXN1L	1,21
solute carrier family 38, member 4	SLC38A4	1,21
solute carrier family 22 (organic anion/urate transporter), member 13	SLC22A13	1,21
coiled-coil domain containing 151	CCDC151	1,21
cyclin and CBS domain divalent metal cation transport mediator 1	CNNM1	1,21
dynein regulatory complex subunit 3	DRC3	1,21
EP400 N-terminal like	EP400NL	1,21
peptidylprolyl isomerase A (cyclophilin A)-like 4A	PPIAL4A	1,21
Rho GTPase activating protein 6	ARHGAP6	1,21
MAS-related GPR, member X1	MRGPRX1	1,21
ADAM metallopeptidase with thrombospondin type 1 motif 15	ADAMTS15	1,21
sushi, von Willebrand factor type A, EGF and pentraxin domain containing 1	SVEP1	1,21
sterile alpha motif domain containing 15	SAMD15	1,21

iron/zinc purple acid phosphatase-like protein; Iron/zinc purple acid phosphatase-like protein [Source:UniProtKB/Swiss-Prot;Acc:Q6ZNF0]; Transcript Identified by AceView, Entrez Gene ID(s) 390928	PAPL	1,21
carboxylesterase 4A	CES4A	1,21
zinc finger protein 608	ZNF608	1,21
microtubule-associated protein 1 light chain 3 beta 2	MAP1LC3B2	1,21
glutamate receptor interacting protein 2	GRIP2	1,21
gamma-aminobutyric acid (GABA) B receptor, 2	GABBR2	1,21
solute carrier family 22 (organic cation/carnitine transporter), member 5	SLC22A5	1,21
proline rich 23 domain containing 2	PRR23D2	1,21
adhesion G protein-coupled receptor G3	ADGRG3	1,21
doublesex and mab-3 related transcription factor 2	DMRT2	1,21
homeobox A3	HOXA3	1,21
Down syndrome cell adhesion molecule like 1	DSCAML1	1,21
interferon, alpha 5	IFNA5	1,21
protein phosphatase 1, regulatory subunit 16A	PPP1R16A	1,21
suppression of tumorigenicity 7 like	ST7L	1,21
X-box binding protein 1	XBP1	1,21
mitochondrial ribosomal protein L11	MRPL11	1,21
protein disulfide isomerase-like, testis expressed	PDILT	1,21
double homeobox 4 like 6	DUX4L6	1,21
double homeobox 4 like 5	DUX4L5	1,21
double homeobox 4 like 1	DUX4L1	1,21
double homeobox 4 like 3	DUX4L3	1,21
double homeobox 4 like 2	DUX4L2	1,21
patched domain containing 1	PTCHD1	1,21
Fc receptor-like 4	FCRL4	1,21
NECAP endocytosis associated 2	NECAP2	1,21
aquaporin 2 (collecting duct)	AQP2	1,21
U-box domain containing 5; FAST kinase domains 5	UBOX5; FASTKD5	1,21
calpain 3	CAPN3	1,21
Salzman2013 ANTISENSE, CDS, coding, INTERNAL, intronic, OVCODE, OVERLAPTX, OVEXON best transcript NM_001014809	CRMP1	1,21
persephin	PSPN	1,21
InaF-motif containing 1	INAFM1	1,21
olfactory receptor, family 2, subfamily A, member 25	OR2A25	1,21
extracellular matrix protein 1	ECM1	1,21
storkhead box 1	STOX1	1,21
armadillo repeat containing 10	ARMC10	1,21
Spi-1 proto-oncogene	SPI1	1,21
M-phase phosphoprotein 6	MPHOSPH6	1,21
DnaJ (Hsp40) homolog, subfamily C, member 13	DNAJC13	1,21

huntingtin	HTT	1,21
linker for activation of T-cells	LAT	1,21
proprotein convertase subtilisin/kexin type 6	PCSK6	1,21
dual specificity phosphatase 23	DUSP23	1,21
lactate dehydrogenase D	LDHD	1,21
tolloid like 1	TLL1	1,21
coiled-coil domain containing 134	CCDC134	1,21
exocyst complex component 8	EXOC8	1,21
mirror-image polydactyly 1	MIPOL1	1,21
butyrophilin-like 2	BTNL2	1,21
SH3 and cysteine rich domain	STAC	1,21
glutamate decarboxylase 2	GAD2	1,21
upstream transcription factor family member 3	USF3	1,21
zinc finger protein 700	ZNF700	1,21
mitochondrial rRNA methyltransferase 1	MRM1	1,21
zinc finger protein 708	ZNF708	1,21
serine peptidase inhibitor, Kunitz type 1	SPINT1	1,21
TAF4b RNA polymerase II, TATA box binding protein (TBP)-associated factor, 105kDa	TAF4B	1,21
CD163 molecule	CD163	1,21
small integral membrane protein 14	SMIM14	1,21
lectin, galactoside-binding, soluble, 16	LGALS16	1,21
olfactory receptor, family 1, subfamily L, member 1	OR1L1	1,21
T-cell acute lymphocytic leukemia 2	TAL2	1,21
sema domain, seven thrombospondin repeats (type 1 and type 1-like), transmembrane domain (TM) and short cytoplasmic domain, (semaphorin) 5B	SEMA5B	1,21
spectrin, alpha, erythrocytic 1	SPTA1	1,21
pecanex homolog (Drosophila)	PCNX	1,21
Transcript Identified by AceView, Entrez Gene ID(s) 3030	HADHA	1,21
HORMA domain containing 1	HORMAD1	1,21
ankyrin repeat and SOCS box containing 2	ASB2	1,21
intestine-specific homeobox	ISX	1,21
parvalbumin	PVALB	1,21
hydroxysteroid (11-beta) dehydrogenase 2	HSD11B2	1,21
Meckel syndrome, type 1	MKS1	1,21
small nuclear RNA activating complex polypeptide 1	SNAPC1	1,21
activin A receptor type IL	ACVRL1	1,21
chromosome 3 open reading frame 80	C3orf80	1,21
synaptophysin	SYP	1,21
sacsin molecular chaperone	SACS	1,21
ADAM metallopeptidase with thrombospondin type 1 motif 17	ADAMTS17	1,21
aldehyde oxidase 1	AOX1	1,21
zinc finger protein 80	ZNF80	1,21

Memczak2013 ANTISENSE, coding, INTERNAL, intronic best transcript NM_001098633	AKT1S1	1,21
formiminotransferase cyclodeaminase N-terminal like	FTCDNL1	1,21
zinc finger protein 433	ZNF433	1,21
protocadherin beta 15	PCDHB15	1,21
tyrosyl-tRNA synthetase	YARS	1,21
zinc finger protein 491	ZNF491	1,21
PTEN induced putative kinase 1; microRNA 6084	PINK1; MIR6084	1,21
ataxin 1	ATXN1	1,21
calpain 5	CAPN5	1,21
spastic paraplegia 11 (autosomal recessive)	SPG11	1,21
proline rich 23A	PRR23A	1,21
prostaglandin I2 (prostacyclin) receptor (IP)	PTGIR	1,21
chromosome 15 open reading frame 41	C15orf41	1,21
dynein, cytoplasmic 2, heavy chain 1	DYNC2H1	1,21
solute carrier family 22 (organic cation transporter), member 3	SLC22A3	1,21
Memczak2013 ALT_ACCEPTOR, ALT_DONOR, coding, INTERNAL, intronic best transcript NM_020772; Transcript Identified by AceView	NUFIP2; rerdy	1,21
NADH dehydrogenase (ubiquinone) 1 alpha subcomplex, 10, 42kDa	NDUFA10	1,21
mannose-6-phosphate receptor (cation dependent)	M6PR	1,21
5-aminolevulinate synthase 2	ALAS2	1,21
SP140 nuclear body protein	SP140	1,21
coiled-coil domain containing 129	CCDC129	1,21
prostate transmembrane protein, androgen induced 1	PMEPA1	1,21
leucine-rich repeats and immunoglobulin-like domains 3	LRIG3	1,21
T-box 2	TBX2	1,21
centrosomal protein 126kDa	CEP126	1,21
glioma tumor suppressor candidate region gene 1	GLTSCR1	1,21
kelch-like family member 28	KLHL28	1,21
cyclin-dependent kinase 11B	CDK11B	1,21
single-pass membrane protein with coiled-coil domains 3	SMCO3	1,21
olfactory receptor, family 2, subfamily AG, member 1 (gene/pseudogene)	OR2AG1	1,21
phosphate cytidylyltransferase 1, choline, alpha	PCYT1A	1,21
spastic paraplegia 7 (pure and complicated autosomal recessive)	SPG7	1,21
dystrobrevin beta	DTNB	1,21
keratin 19, type I	KRT19	1,21
paired-like homeodomain 1	PITX1	1,21
ladinin 1	LAD1	1,21
Memczak2013 ANTISENSE, coding, INTERNAL, intronic best transcript NM_001040442	FABP6	1,21

semenogelin I	SEMG1	1,21
zinc finger protein 114	ZNF114	1,21
endothelin 2	EDN2	1,21
receptor (chemosensory) transporter protein 2	RTP2	1,21
myosin IA	MYO1A	1,21
defensin, alpha 4, corticostatin	DEFA4	1,21
EYA transcriptional coactivator and phosphatase 2	EYA2	1,21
coiled-coil domain containing 149	CCDC149	1,21
RNA binding motif, single stranded interacting protein 2	RBMS2	1,21
X antigen family, member 2	XAGE2	1,21
asparaginyl-tRNA synthetase	NARS	1,21
SRY box 12	SOX12	1,21
microtubule associated monooxygenase, calponin and LIM domain containing 2	MICAL2	1,21
testis expressed 13B	TEX13B	1,21
chromosome 2 open reading frame 48	C2orf48	1,21
kallikrein related peptidase 12	KLK12	1,21
anoctamin 8	ANO8	1,21
receptor accessory protein 1	REEP1	1,21
sodium channel, voltage gated, type IV alpha subunit	SCN4A	1,21
LDL receptor related protein 2	LRP2	1,21
notch 2 N-terminal like	NOTCH2NL	1,21
Transcript Identified by AceView, Entrez Gene ID(s) 153339	TMEM167A	1,21
hypoxia up-regulated 1	HYOU1	1,21
phenylalanyl-tRNA synthetase 2, mitochondrial	FARS2	1,21
sterol regulatory element binding transcription factor 1	SREBF1	1,21
cadherin 3, type 1, P-cadherin (placental)	CDH3	1,21
keratin 37, type I	KRT37	1,21
Rap guanine nucleotide exchange factor 2	RAPGEF2	1,21
zinc finger with KRAB and SCAN domains 2	ZKSCAN2	1,21
glutamate receptor, ionotropic, N-methyl D-aspartate 1	GRIN1	1,21
Transcript Identified by AceView, Entrez Gene ID(s) 7037	TFRC	1,21
membrane-spanning 4-domains, subfamily A, member 1	MS4A1	1,21
zinc finger protein 607	ZNF607	1,21
deoxyribonuclease I	DNASE1	1,21
family with sequence similarity 43, member A	FAM43A	1,21
DNA-damage-inducible transcript 3	DDIT3	1,21
family with sequence similarity 102, member B	FAM102B	1,21
protein kinase, cGMP-dependent, type II	PRKG2	1,20
TIMP metallopeptidase inhibitor 1	TIMP1	1,20
olfactory receptor, family 2, subfamily T, member 1	OR2T1	1,20
Transcript Identified by AceView, Entrez Gene ID(s) 143279	HECTD2	1,20
transmembrane protein 132D	TMEM132D	1,20

COMMD3-BMI1 readthrough; BMI1 proto-oncogene, polycomb ring finger; COMM domain containing 3	COMMD3-BMI1; BMI1; COMMD3	1,20
ZFP69 zinc finger protein B	ZFP69B	1,20
SMG6 nonsense mediated mRNA decay factor	SMG6	1,20
vesicle transport through interaction with t-SNAREs 1A	VTI1A	1,20
calcium channel, voltage-dependent, gamma subunit 1	CACNG1	1,20
methylenetetrahydrofolate dehydrogenase (NADP+ dependent) 2, methenyltetrahydrofolate cyclohydrolase	MTHFD2	1,20
family with sequence similarity 196, member A	FAM196A	1,20
diacylglycerol kinase, delta 130kDa	DGKD	1,20
lysyl oxidase-like 4	LOXL4	1,20
adenosine A1 receptor	ADORA1	1,20
arginine vasopressin receptor 2	AVPR2	1,20
Zhang2013 ALT_ACCEPTOR, ALT_DONOR, coding, INTERNAL, intronic best transcript NM_002473	MYH9	1,20
large 60S subunit nuclear export GTPase 1	LSG1	1,20
zinc finger protein 501 [Source:HGNC Symbol;Acc:HGNC:23717]	ZNF501	1,20
translocase of inner mitochondrial membrane 22 homolog (yeast)	TIMM22	1,20
WWC family member 3	WWC3	1,20
CD209 molecule	CD209	1,20
ribosomal protein S6 kinase, 90kDa, polypeptide 1	RPS6KA1	1,20
C1q and tumor necrosis factor related protein 3	C1QTNF3	1,20
p21 protein (Cdc42/Rac)-activated kinase 3	PAK3	1,20
aspartate-rich 1	DRICH1	1,20
Rho guanine nucleotide exchange factor 1	ARHGEF1	1,20
insulinoma-associated 1	INSM1	1,20
cadherin 26	CDH26	1,20
zinc finger protein 81	ZNF81	1,20
Memczak2013 ANTISENSE, CDS, coding, INTERNAL best transcript NM_001456	FLNA	1,20
chromosome 4 open reading frame 48	C4orf48	1,20
mediator complex subunit 7	MED7	1,20
olfactory receptor, family 4, subfamily S, member 2	OR4S2	1,20
Memczak2013 ALT_ACCEPTOR, ALT_DONOR, coding, INTERNAL, intronic best transcript NM_014661	FAM53B	1,20
adenylate kinase 7	AK7	1,20
cytochrome c oxidase subunit VIIIC	COX8C	1,20
ATP synthase mitochondrial F1 complex assembly factor 2	ATPAF2	1,20
glycophorin A (MNS blood group)	GYPA	1,20
phosphodiesterase 6G, cGMP-specific, rod, gamma	PDE6G	1,20
SLAM family member 9	SLAMF9	1,20

GIMAP1-GIMAP5 readthrough; GTPase, IMAP family member 5; GTPase, IMAP family member 1	GIMAP1-GIMAP5; GIMAP5; GIMAP1	1,20
TM2 domain containing 3	TM2D3	1,20
formin 1	FMN1	1,20
MAS1 proto-oncogene like, G protein-coupled receptor	MAS1L	1,20
peptidyl arginine deiminase, type IV	PADI4	1,20
transmembrane protein 159	TMEM159	1,20
elastin microfibril interfacer 2	EMILIN2	1,20
MAGE family member B5	MAGEB5	1,20
lectin, galactoside-binding, soluble, 2	LGALS2	1,20
secreted protein, acidic, cysteine-rich (osteonectin)	SPARC	1,20
tripartite motif containing 39	TRIM39	1,20
calcium and integrin binding family member 4	CIB4	1,20
ankyrin repeat and sterile alpha motif domain containing 3	ANKS3	1,20
BTB (POZ) domain containing 9	BTBD9	1,20
nexilin (F actin binding protein)	NEXN	1,20
G protein-coupled receptor, class C, group 5, member B	GPRC5B	1,20
6-phosphofructo-2-kinase/fructose-2,6-biphosphatase 1	PFKFB1	1,20
sortilin-related VPS10 domain containing receptor 2	SORCS2	1,20
CREB regulated transcription coactivator 3	CRTC3	1,20
fibrillarin-like 1	FBLL1	1,20
glyoxalase domain containing 5	GLOD5	1,20
ribonuclease, RNase A family, 2 (liver, eosinophil-derived neurotoxin)	RNASE2	1,20
RNA binding motif protein 6	RBM6	1,20
crystallin gamma B	CRYGB	1,20
solute carrier family 10, member 3	SLC10A3	1,20
RNA binding motif protein 20	RBM20	1,20
MIS18 kinetochore protein A	MIS18A	1,20
caspase recruitment domain family, member 17	CARD17	1,20
embryonic ectoderm development; microRNA 6755	EED; MIR6755	1,20
folliculogenesis specific bHLH transcription factor	FIGLA	1,20
chromosome 4 open reading frame 22	C4orf22	1,20
zinc finger protein 432	ZNF432	1,20
peptidyl arginine deiminase, type I	PADI1	1,20
colorectal cancer associated 2	COLCA2	1,20
zinc finger protein 397	ZNF397	1,20
Memczak2013 ALT_ACCEPTOR, ALT_DONOR, coding, INTERNAL, intronic best transcript NM_001136017	CCND3	1,20
HUS1 checkpoint clamp component	HUS1	1,20
collagen, type VI, alpha 5	COL6A5	1,20
olfactory receptor, family 51, subfamily E, member 2	OR51E2	1,20
A kinase (PRKA) anchor protein 10	AKAP10	1,20

microtubule associated protein tau	MAPT	1,20
regulator of G-protein signaling 10	RGS10	1,20
discs, large (Drosophila) homolog-associated protein 2	DLGAP2	1,20
ankyrin repeat domain 20 family, member A4	ANKRD20A4	1,20
calcium channel, voltage-dependent, gamma subunit 8; microRNA 935	CACNG8; MIR935	1,20
defensin, beta 124	DEFB124	1,20
laeverin	LVRN	1,20
ROR1 antisense RNA 1	ROR1-AS1	1,20
sema domain, immunoglobulin domain (Ig), transmembrane domain (TM) and short cytoplasmic domain, (semaphorin) 4D	SEMA4D	1,20
potassium channel tetramerization domain containing 15	KCTD15	1,20
chromosome 1 open reading frame 141	C1orf141	1,20
olfactory receptor, family 10, subfamily A, member 3	OR10A3	1,20
inositol polyphosphate-5-phosphatase D	INPP5D	1,20
RAS (RAD and GEM)-like GTP binding 2	REM2	1,20
solute carrier family 24 (sodium/potassium/calcium exchanger), member 5	SLC24A5	1,20
zinc finger protein 626	ZNF626	1,20
PTPRF interacting protein, binding protein 1 (liprin beta 1)	PPFIBP1	1,20
interleukin 10 receptor, alpha	IL10RA	1,20
CD34 molecule	CD34	1,20
leucine rich repeat containing 6	LRRC6	1,20
DnaJ (Hsp40) homolog, subfamily B, member 2	DNAJB2	1,20
spermatogenesis associated 18	SPATA18	1,20
uncharacterized serine/threonine-protein kinase SgK494; Synthetic construct Homo sapiens clone IMAGE:100064171, MGC:193254 uncharacterized serine/threonine-protein kinase SgK494 (FLJ25006) mRNA, encodes complete protein.; Salzman2013 ANNOTATED, CDS, coding, INTERNAL, OVCODE, OVERLAPTX, OVEXON best transcript NM_001174103; Transcript Identified by AceView, Entrez Gene ID(s) 124923; 9703; 645851; 10615; uncharacterized serine/threonine-protein kinase SgK494 [Source:EntrezGene;Acc:124923]; Uncharacterized protein [Source:UniProtKB/TrEMBL;Acc:J3KTE0]	SGK494; FLJ25006; KIAA0100andSGK494andSPAG5; RP11-192H23.4	1,20
Transcript Identified by AceView, Entrez Gene ID(s) 83941	TM2D1	1,20
sperm protein associated with the nucleus, X-linked, family member A1	SPANXA1	1,20
F-box and WD repeat domain containing 8	FBXW8	1,20
kelch-like family member 2	KLHL2	1,20
Rh blood group, CcEe antigens	RHCE	1,20
TP73 antisense RNA 1	TP73-AS1	1,20
lymphocyte transmembrane adaptor 1	LAX1	1,20
dpy-19-like 4 (C. elegans)	DPY19L4	1,20

Transcript Identified by AceView, Entrez Gene ID(s) 8502	PKP4	1,20
zinc finger protein 780A	ZNF780A	1,20
CUE domain containing 1	CUEDC1	1,20
SET domain containing 4	SETD4	1,20
chromosome 5 open reading frame 42	C5orf42	1,20
CKLF-like MARVEL transmembrane domain containing 4	CMTM4	1,20
ubiquitin domain containing 2	UBTD2	1,20
microtubule-actin crosslinking factor 1; KIAA0754	MACF1; KIAA0754	1,20
cyclic nucleotide gated channel beta 3	CNGB3	1,20
EF-hand calcium binding domain 7; deleted in lymphocytic leukemia 2-like	EFCAB7; DLEU2L	1,20
potassium channel, voltage gated shaker related subfamily A, member 5	KCNA5	1,20
teashirt zinc finger homeobox 2	TSHZ2	1,20
Transcript Identified by AceView, Entrez Gene ID(s) 54443	ANLN	1,20
glycerol kinase 2	GK2	1,20
membrane protein, palmitoylated 2	MPP2	1,20
spermine oxidase	SMOX	1,20
pleckstrin homology domain containing, family J member 1; microRNA 6789	PLEKHJ1; MIR6789	1,20
SPRY domain containing 4	SPRYD4	1,20
HECT and RLD domain containing E3 ubiquitin protein ligase 2	HERC2	1,20
REX1, RNA exonuclease 1 homolog; microRNA 1909	REXO1; MIR1909	1,20
retinoblastoma binding protein 5	RBBP5	1,20
SH3-domain GRB2-like (endophilin) interacting protein 1	SGIP1	1,20
zinc finger protein 660	ZNF660	1,20
olfactory receptor, family 52, subfamily A, member 1	OR52A1	1,20
melatonin receptor 1B	MTNR1B	1,20
chromosome 19 open reading frame 73	C19orf73	1,20
keratin associated protein 5-11	KRTAP5-11	1,20
doublesex and mab-3 related transcription factor 3	DMRT3	1,20
SH3-domain kinase binding protein 1	SH3KBP1	1,20
kelch domain containing 1	KLHDC1	1,20
diacylglycerol kinase theta	DGKQ	1,20
proopiomelanocortin	POMC	1,20
Kell blood group, metallo-endopeptidase	KEL	1,20
defensin, beta 130	DEFB130	1,20
defensin, beta 130	DEFB130	1,20
adhesion molecule with Ig-like domain 2	AMIGO2	1,20
olfactory receptor, family 8, subfamily U, member 1; olfactory receptor, family 8, subfamily U, member 8	OR8U1; OR8U8	1,20
TBC1 domain family, member 2B pseudogene; Transcript Identified by AceView, Entrez Gene ID(s) 646938	LOC646938	1,20

Memczak2013 ANTISENSE, coding, INTERNAL, intronic best transcript NM_015995	KLF13	1,20
erythrocyte membrane protein band 4.2	EPB42	1,20
dehydrogenase/reductase (SDR family) member 9	DHRS9	1,20
diphthamide biosynthesis 5	DPH5	1,20
mex-3 RNA binding family member D	MEX3D	1,20
integrin linked kinase	ILK	1,20
WNT1 inducible signaling pathway protein 2	WISP2	1,20
myosin, heavy chain 8, skeletal muscle, perinatal	MYH8	1,20
jumonji domain containing 7; phospholipase A2, group IVB (cytosolic); JMJD7-PLA2G4B readthrough	JMJD7; PLA2G4B; JMJD7-PLA2G4B	1,20
calcium channel, voltage-dependent, T type, alpha 1I subunit	CACNA1I	1,20
cyclin-dependent kinase 5	CDK5	1,20
alkaline phosphatase, placental	ALPP	1,20
paroxysmal nonkinesigenic dyskinesia; microRNA 6810	PNKD; MIR6810	1,20
chymotrypsin-like elastase family, member 2B	CELA2B	1,20
ankyrin repeat domain 18B	ANKRD18B	1,20
major facilitator superfamily domain containing 14B	MFSD14B	1,20
rhophilin, Rho GTPase binding protein 1	RHPN1	1,20
transmembrane (C-terminal) protease, serine 12	TMPRSS12	1,20
olfactory receptor, family 4, subfamily N, member 4	OR4N4	1,20
chromosome 10 open reading frame 62	C10orf62	1,20
neuroblastoma breakpoint family, member 19	NBPF19	1,20
kinesin light chain 1	KLC1	1,20
testis expressed 30	TEX30	1,20
trafficking protein particle complex 10	TRAPPC10	1,20
tripartite motif containing 49C	TRIM49C	1,20
TAF1 RNA polymerase II, TATA box binding protein (TBP)-associated factor, 250kDa	TAF1	1,20
craniofacial development protein 1	CFDP1	1,20
neurofibromin 1	NF1	1,20
coiled-coil domain containing 60	CCDC60	1,20
serine active site containing 1	SERAC1	1,20
nuclear receptor subfamily 2, group F, member 1	NR2F1	1,20
ATP5S-like	ATP5SL	1,20
apoptosis inhibitor 5	API5	1,20
CD99 molecule	CD99	1,20
phosphatidylinositol-5-phosphate 4-kinase, type II, beta	PIP4K2B	1,20
cholinergic receptor, nicotinic alpha 7	CHRNA7	1,20
EPH receptor A3	EPHA3	1,20
Transcript Identified by AceView, Entrez Gene ID(s) 257415	FAM133B	1,20
SH3 domain containing 21	SH3D21	1,20

tripartite motif containing 55	TRIM55	1,20
hypocretin (orexin) receptor 2	HCRTR2	1,20
solute carrier family 22 (organic cation transporter), member 1	SLC22A1	1,20
olfactory receptor, family 6, subfamily J, member 1 (gene/pseudogene)	OR6J1	1,20
uncharacterized LOC338797; novel transcript	LOC338797; RP13-507P19.2; RP13-507P19.1	1,20
potassium channel, voltage gated eag related subfamily H, member 6	KCNH6	1,20
interleukin 17 receptor D	IL17RD	1,20
Ras-related GTP binding B	RRAGB	1,20
olfactory receptor, family 11, subfamily H, member 7 (gene/pseudogene)	OR11H7	1,20
fructosamine 3 kinase related protein	FN3KRP	1,20
transmembrane and coiled-coil domains 5A	TMCO5A	1,20
MAGE family member C3	MAGEC3	1,20
Fanconi anemia complementation group M	FANCM	1,20
TBC1 domain family, member 7	TBC1D7	1,20
polycomb group ring finger 3	PCGF3	1,20
ankyrin repeat domain 20 family, member A5, pseudogene; ras homolog family member T1 pseudogene 1	ANKRD20A5P; RHOT1P1	1,20
calneuron 1	CALN1	1,20
leukocyte immunoglobulin-like receptor, subfamily B (with TM and ITIM domains), member 3	LILRB3	1,20
keratinocyte differentiation-associated protein	KRTDAP	1,20
Down syndrome cell adhesion molecule	DSCAM	1,20
zinc finger protein 586	ZNF586	1,20
tetratricopeptide repeat domain 26	TTC26	1,20
POU class 6 homeobox 1	POU6F1	1,20
mitochondrial transcription termination factor 4	MTERF4	1,20
solute carrier family 6 (neurotransmitter transporter), member 11	SLC6A11	1,20
tryptophanyl tRNA synthetase 2, mitochondrial	WARS2	1,20
DiGeorge syndrome critical region gene 6	DGCR6	1,20
transmembrane BAX inhibitor motif containing 6	TMBIM6	1,20
RUN domain containing 3A	RUNDC3A	1,20
syntabulin (syntaxin-interacting)	SYBU	1,20
zinc finger protein 326	ZNF326	1,20
dual specificity phosphatase and pro isomerase domain containing 1	DUPD1	1,20
chromosome 8 open reading frame 46	C8orf46	1,20
ATP/GTP binding protein-like 2	AGBL2	1,20
lectin, galactoside-binding, soluble, 9B	LGALS9B	1,20

transcription factor AP-4 (activating enhancer binding protein 4)	TFAP4	1,20
uncharacterized LOC730159; putative novel transcript	LOC730159; RP11-360D2.1	1,20
DnaJ (Hsp40) homolog, subfamily C, member 6	DNAJC6	1,20
ubinuclein 2	UBN2	1,20
sarcospan	SSPN	1,20
mannosidase, alpha, class 2B, member 2	MAN2B2	1,20
O-sialoglycoprotein endopeptidase	OSGEP	1,20
family with sequence similarity 174, member A	FAM174A	1,20
suppressor APC domain containing 2	SAPCD2	1,20
keratin associated protein 5-8	KRTAP5-8	1,20
zinc finger protein 23	ZNF23	1,20
kallikrein related peptidase 5	KLK5	1,20
beta-1,3-glucuronyltransferase 1	B3GAT1	1,20
lipocalin 12	LCN12	1,20
GLI pathogenesis-related 1	GLIPR1	1,20
lymphoid-restricted membrane protein	LRMP	1,19
corin, serine peptidase	CORIN	1,19
zinc finger protein 189	ZNF189	1,19
SIX homeobox 1	SIX1	1,19
kinesin family member 5A	KIF5A	1,19
trafficking protein particle complex 4	TRAPPC4	1,19
fibronectin type III and SPRY domain containing 1-like	FSD1L	1,19
NHS-like 2	NHSL2	1,19
like-glycosyltransferase	LARGE	1,19
glutamate receptor, ionotropic, kainate 2	GRIK2	1,19
nucleolar and coiled-body phosphoprotein 1	NOLC1	1,19
calcium channel, voltage-dependent, T type, alpha 1G subunit	CACNA1G	1,19
G protein-coupled receptor 146	GPR146	1,19
tripartite motif containing 11; microRNA 6742	TRIM11; MIR6742	1,19
coiled-coil domain containing 89	CCDC89	1,19
regulatory factor X, 4 (influences HLA class II expression)	RFX4	1,19
GRIP1 associated protein 1	GRIPAP1	1,19
schlafen family member 5	SLFN5	1,19
small ubiquitin-like modifier 4	SUMO4	1,19
leucine rich repeat containing 27	LRRC27	1,19
aprataxin and PNKP like factor	APLF	1,19
kelch repeat and BTB (POZ) domain containing 13	KBTBD13	1,19
inositol polyphosphate-5-phosphatase D	INPP5D	1,19
zinc finger protein 623	ZNF623	1,19
zinc finger protein 343	ZNF343	1,19
cystatin S	CST4	1,19
uncharacterized LOC102724238; uncharacterized LOC554249	LOC102724238; LOC554249	1,19

olfactory receptor, family 14, subfamily C, member 36	OR14C36	1,19
bone morphogenetic protein 8a	BMP8A	1,19
transmembrane protein 79	TMEM79	1,19
tRNA phosphotransferase 1	TRPT1	1,19
dickkopf WNT signaling pathway inhibitor 1	DKK1	1,19
cystatin D	CST5	1,19
interleukin 27 receptor, alpha	IL27RA	1,19
RUN and FYVE domain containing 1	RUFY1	1,19
chromosome 18 open reading frame 8	C18orf8	1,19
zinc finger protein 627	ZNF627	1,19
Zhang2013 ALT_ACCEPTOR, ALT_DONOR, coding, INTERNAL, intronic, OVERLAPTX best transcript NM_002140	HNRNPK	1,19
myosin, heavy chain 6, cardiac muscle, alpha	MYH6	1,19
proteasome 26S subunit, ATPase 4 [Source:HGNC Symbol;Acc:HGNC:9551]	PSMC4	1,19
myelin regulatory factor-like	MYRFL	1,19
chromosome 3 open reading frame 22	C3orf22	1,19
actin binding LIM protein family, member 2	ABLIM2	1,19
methyltransferase like 16	METTL16	1,19
Transcript Identified by AceView, Entrez Gene ID(s) 238	ALK	1,19
solute carrier family 3 (amino acid transporter heavy chain), member 2	SLC3A2	1,19
sterile alpha motif domain containing 5	SAMD5	1,19
mesencephalic astrocyte-derived neurotrophic factor	MANF	1,19
potassium channel, voltage gated KQT-like subfamily Q, member 3	KCNQ3	1,19
nuclear pore associated protein 1	NPAP1	1,19
killer cell immunoglobulin-like receptor, three domains, X1	KIR3DX1	1,19
cleft lip and palate associated transmembrane protein 1	CLPTM1	1,19
huntingtin interacting protein 1	HIP1	1,19
transmembrane protein 116	TMEM116	1,19
F-box and leucine-rich repeat protein 16	FBXL16	1,19
alanine-glyoxylate aminotransferase	AGXT	1,19
lipase, endothelial	LIPG	1,19
EF-hand calcium binding domain 5	EFCAB5	1,19
cadherin 10, type 2 (T2-cadherin)	CDH10	1,19
microRNA 659; ankyrin repeat domain 54	MIR659; ANKRD54	1,19
growth hormone releasing hormone	GHRH	1,19
protein kinase, cAMP-dependent, regulatory, type I, alpha; arylsulfatase G	PRKAR1A; ARSG	1,19
Memczak2013 ANTISENSE, CDS, coding, INTERNAL, intronic best transcript NM_001077401	ACVRL1	1,19
Transcript Identified by AceView, Entrez Gene ID(s) 63899	NSUN3	1,19
keratin 82, type II	KRT82	1,19

ADP-ribosylation factor like GTPase 17B; ADP-ribosylation factor like GTPase 17A	ARL17B; ARL17A	1,19
Homo sapiens uncharacterized LOC79999 (LOC79999), mRNA.; Homo sapiens uncharacterized protein ENSP00000382042 (LOC388436), mRNA.; novel transcript antisense to GRAPL	LOC79999; LOC388436; AC007952.6	1,19
otoferlin	OTOF	1,19
family with sequence similarity 83, member A	FAM83A	1,19
RAB3A, member RAS oncogene family	RAB3A	1,19
ferritin, heavy polypeptide 1 pseudogene 18	FTH1P18	1,19
Memczak2013 ALT_ACCEPTOR, ALT_DONOR, coding, INTERNAL, intronic best transcript NM_006454	MXD4	1,19
keratin 7, type II	KRT7	1,19
chromogranin B	CHGB	1,19
ALG1, chitobiosyldiphosphodolichol beta-mannosyltransferase-like 2	ALG1L2	1,19
supervillin	SVIL	1,19
Memczak2013 ANTISENSE, coding, INTERNAL, intronic best transcript NM_001105192	TLE3	1,19
nuclear transcription factor Y subunit alpha	NFYA	1,19
serpin peptidase inhibitor, clade E (nexin, plasminogen activator inhibitor type 1), member 2	SERPINE2	1,19
protein kinase, membrane associated tyrosine/threonine 1	PKMYT1	1,19
bone morphogenetic protein receptor type II	BMPR2	1,19
midasin AAA ATPase 1	MDN1	1,19
ring finger protein 223	RNF223	1,19
defensin, beta 113	DEFB113	1,19
YLP motif containing 1	YLPM1	1,19
Homo sapiens synaptotagmin XV (SYT15), transcript variant a, mRNA.; Homo sapiens synaptotagmin XV (SYT15), transcript variant b, mRNA.; Homo sapiens synaptotagmin XV, mRNA (cDNA clone MGC:164845 IMAGE:40147906), complete cds.; Synaptotagmin-15 [Source:UniProtKB/Swiss-Prot;Acc:Q9BQS2]	SYT15; ABC7-42404400C24.1	1,19
copper metabolism (Murr1) domain containing 1	COMMD1	1,19
olfactory receptor, family 4, subfamily F, member 4; olfactory receptor, family 4, subfamily F, member 17	OR4F4; OR4F17	1,19
TSEN2 tRNA splicing endonuclease subunit	TSEN2	1,19
stromal antigen 3-like 1 (pseudogene)	STAG3L1	1,19
Rho GTPase activating protein 25	ARHGAP25	1,19
long intergenic non-protein coding RNA 521	LINC00521	1,19
chromosome 12 open reading frame 77	C12orf77	1,19
serpin peptidase inhibitor, clade B (ovalbumin), member 5	SERPINB5	1,19
CDK5 regulatory subunit associated protein 3	CDK5RAP3	1,19
cofilin 1 (non-muscle)	CFL1	1,19
von Willebrand factor A domain containing 8; microRNA 5006	VWA8; MIR5006	1,19

placenta specific 1	PLAC1	1,19
LIM and senescent cell antigen-like domains 3-like	LIMS3L	1,19
phosphoglycerate mutase 2 (muscle)	PGAM2	1,19
inhibin beta E	INHBE	1,19
plasminogen activator, tissue	PLAT	1,19
5-azacytidine induced 2	AZI2	1,19
nuclear factor of activated T-cells, cytoplasmic, calcineurin-dependent 2	NFATC2	1,19
zinc finger protein 426	ZNF426	1,19
surfeit 6	SURF6	1,19
heat shock transcription factor family, X-linked 2; heat shock transcription factor family, X-linked 1	HSFX2; HSFX1	1,19
blood vessel epicardial substance	BVES	1,19
galanin receptor 2	GALR2	1,19
peroxisomal biogenesis factor 14	PEX14	1,19
multiple C2 domains, transmembrane 2	MCTP2	1,19
family with sequence similarity 73, member A	FAM73A	1,19
shisa family member 8	SHISA8	1,19
tRNA methyltransferase 11 homolog	TRMT11	1,19
olfactory receptor, family 4, subfamily F, member 6	OR4F6	1,19
Rho GTPase activating protein 30	ARHGAP30	1,19
interferon, alpha 4	IFNA4	1,19
guanine nucleotide binding protein (G protein), alpha activating activity polypeptide, olfactory type	GNAL	1,19
semaphorin 7A, GPI membrane anchor (John Milton Hagen blood group)	SEMA7A	1,19
trichoplein, keratin filament binding	TCHP	1,19
cripto, FRL-1, cryptic family 1B; cripto, FRL-1, cryptic family 1	CFC1B; CFC1	1,19
transmembrane protein 213	TMEM213	1,19
troponin T type 3 (skeletal, fast)	TNNT3	1,19
VPS11, CORVET/HOPS core subunit [Source:HGNC Symbol;Acc:HGNC:14583]	VPS11	1,19
ankyrin repeat domain 23	ANKRD23	1,19
aarF domain containing kinase 1	ADCK1	1,19
lymphocyte antigen 6 complex, locus G5C	LY6G5C	1,19
leucine rich repeat containing 42	LRRC42	1,19
mediator complex subunit 31	MED31	1,19
programmed cell death 6	PDCD6	1,19
late cornified envelope 1C	LCE1C	1,19
proline-rich transmembrane protein 4	PRRT4	1,19
angiomotin; microRNA 4329	AMOT; MIR4329	1,19
cystatin 9-like	CST9L	1,19
programmed cell death 11	PDCD11	1,19
family with sequence similarity 110, member C	FAM110C	1,19

PIH1 domain containing 3	PIH1D3	1,19
mitochondrial inner membrane organizing system 1	MINOS1	1,19
gonadotropin releasing hormone 1	GNRH1	1,19
ring finger protein (C3H2C3 type) 6	RNF6	1,19
cornichon family AMPA receptor auxiliary protein 1	CNIH1	1,19
THAP7 antisense RNA 1	THAP7-AS1	1,19
KIAA1456	KIAA1456	1,19
Transcript Identified by AceView, Entrez Gene ID(s) 135112	NCOA7	1,19
murine retrovirus integration site 1 homolog	MRVI1	1,19
anaphase promoting complex subunit 15	ANAPC15	1,19
prolactin-induced protein	PIP	1,19
coiled-coil domain containing 53	CCDC53	1,19
egl-9 family hypoxia-inducible factor 3	EGLN3	1,19
dynactin 4 (p62)	DCTN4	1,19
T-cell lymphoma invasion and metastasis 2	TIAM2	1,19
Transcript Identified by AceView, Entrez Gene ID(s) 5747	PTK2	1,19
cytochrome P450, family 7, subfamily A, polypeptide 1	CYP7A1	1,19
olfactory receptor, family 4, subfamily D, member 6	OR4D6	1,19
T-cell leukemia homeobox 1	TLX1	1,19
Memczak2013 ALT_ACCEPTOR, ALT_DONOR, coding, INTERNAL, intronic best transcript NM_031453	FAM107B	1,19
GRB2-related adaptor protein-like	GRAPL	1,19
serpin peptidase inhibitor, clade C (antithrombin), member 1	SERPINC1	1,19
FCH and double SH3 domains 1	FCHSD1	1,19
Transcript Identified by AceView, Entrez Gene ID(s) 5136	PDE1A	1,19
keratin 85, type II	KRT85	1,19
GLI family zinc finger 1	GLI1	1,19
HLA complex group 22	HCG22	1,19
uncharacterized LOC102724323; Transcript Identified by AceView, Entrez Gene ID(s) 220972; novel transcript	LOC102724323; RP11-67C2.2; MARCH8	1,19
Bardet-Biedl syndrome 12	BBS12	1,19
UHRF1 binding protein 1	UHRF1BP1	1,19
pleckstrin homology domain containing, family G (with RhoGef domain) member 6	PLEKHG6	1,19
chromosome 2 open reading frame 81	C2orf81	1,19
schlafen family member 11	SLFN11	1,19
exportin, tRNA	XPOT	1,19
retina and anterior neural fold homeobox	RAX	1,19
cyclin B1	CCNB1	1,19
collagen, type XXI, alpha 1	COL21A1	1,19
LPS-responsive vesicle trafficking, beach and anchor containing	LRBA	1,19
polynucleotide kinase 3-phosphatase	PNKP	1,19
dedicator of cytokinesis 5	DOCK5	1,19
ATP binding cassette subfamily C member 1	ABCC1	1,19

aconitase 1, soluble	ACO1	1,19
prostaglandin I2 (prostacyclin) synthase	PTGIS	1,19
chromosome 7 open reading frame 34	C7orf34	1,19
DDB1 and CUL4 associated factor 8	DCAF8	1,19
gigaxonin; microRNA 4720	GAN; MIR4720	1,19
olfactory receptor, family 4, subfamily D, member 5	OR4D5	1,19
IGF like family member 3	IGFL3	1,19
tripartite motif containing 68	TRIM68	1,19
adenomatous polyposis coli	APC	1,19
MYST/Esa1-associated factor 6	MEAF6	1,19
threonyl-tRNA synthetase-like 2	TARSL2	1,19
cytochrome P450, family 4, subfamily F, polypeptide 3	CYP4F3	1,19
lactate dehydrogenase B	LDHB	1,19
egl-9 family hypoxia-inducible factor 3	EGLN3	1,19
Jeck2013 ALT_ACCEPTOR, ALT_DONOR, coding, INTERNAL, intronic best transcript NM_152900	MAGI3	1,19
olfactomedin like 3	OLFML3	1,19
calcium channel, voltage-dependent, L type, alpha 1C subunit	CACNA1C	1,19
keratin 18 pseudogene 55	KRT18P55	1,19
Memczak2013 ANTISENSE, CDS, coding, INTERNAL best transcript NM_181786	HKR1	1,19
Zhang2013 ALT_ACCEPTOR, ALT_DONOR, coding, INTERNAL, intronic best transcript NM_001007	RPS4X	1,19
mitogen-activated protein kinase kinase kinase 6	MAP3K6	1,19
regenerating islet-derived family, member 4	REG4	1,19
zinc finger protein 345	ZNF345	1,19
olfactory receptor, family 2, subfamily T, member 8	OR2T8	1,19
ATPase, H+ transporting, lysosomal 56/58kDa, V1 subunit B1	ATP6V1B1	1,19
polymerase (RNA) III (DNA directed) polypeptide K, 12.3 kDa	POLR3K	1,19
chromatin target of PRMT1	CHTOP	1,19
defensin, beta 4B	DEFB4B	1,19
cancer/testis antigen family 47, member A1	CT47A1	1,19
adenosine deaminase, tRNA-specific 1	ADAT1	1,19
lectin, galactoside-binding, soluble, 13	LGALS13	1,19
metallothionein 1M	MT1M	1,19
GRB2-associated binding protein 1	GAB1	1,19
family with sequence similarity 122B	FAM122B	1,19
myeloperoxidase	MPO	1,19
uncharacterized LOC102724279; Transcript Identified by AceView; novel transcript	LOC102724279; AC004791.2; nimure	1,19
cyclic nucleotide binding domain containing 2	CNBD2	1,19
cytochrome P450, family 24, subfamily A, polypeptide 1	CYP24A1	1,19
calcium channel flower domain containing 1	CACFD1	1,19

leucine-rich repeats and calponin homology (CH) domain containing 2	LRCH2	1,19
neuroblastoma breakpoint family, member 7	NBPF7	1,19
family with sequence similarity 3, member D	FAM3D	1,19
solute carrier family 10 (sodium/bile acid cotransporter), member 6	SLC10A6	1,19
fibroblast growth factor 12	FGF12	1,19
GTF2I repeat domain containing 2; GTF2I repeat domain containing 2B	GTF2IRD2; GTF2IRD2B	1,19
cytochrome P450, family 4, subfamily V, polypeptide 2	CYP4V2	1,19
G antigen 12F; G antigen 12J; G antigen 12D; G antigen 5; G antigen 6; G antigen 12B; G antigen 4; G antigen 2E	GAGE12F; GAGE12J; GAGE12D; GAGE5; GAGE6; GAGE12B; GAGE4; GAGE2E	1,19
ankyrin repeat domain 36B	ANKRD36B	1,19
Transcript Identified by AceView, Entrez Gene ID(s) 158219	TTC39B	1,19
protocadherin beta 10	PCDHB10	1,19
Memczak2013 ANTISENSE, coding, INTERNAL, intronic best transcript NM_021931	DHX35	1,19
Memczak2013 ANTISENSE, coding, INTERNAL, intronic best transcript NM_005308	GRK5	1,19
NOTCH-regulated ankyrin repeat protein	NRARP	1,19
gamma-aminobutyric acid (GABA) A receptor, rho 3 (gene/pseudogene)	GABRR3	1,19
GDNF antisense RNA 1 (head to head)	GDNF-AS1	1,19
C-type lectin domain family 10, member A	CLEC10A	1,19
transmembrane protein 163	TMEM163	1,18
schlafen family member 12-like	SLFN12L	1,18
free fatty acid receptor 1	FFAR1	1,18
interleukin 18 receptor accessory protein	IL18RAP	1,18
sulfotransferase family 2B member 1	SULT2B1	1,18
zinc finger protein 333	ZNF333	1,18
synaptonemal complex protein 2	SYCP2	1,18
alkaline phosphatase, intestinal	ALPI	1,18
nuclear pore complex interacting protein family, member A8	NPIPA8	1,18
POU class 2 homeobox 2	POU2F2	1,18
cancer susceptibility candidate 10	CASC10	1,18
myosin VA	MYO5A	1,18
acyl-CoA synthetase long-chain family member 1	ACSL1	1,18
ADP-ribosyltransferase 5	ART5	1,18
forkhead box D3	FOXD3	1,18

multiple EGF-like-domains 8	MEGF8	1,18
transmembrane protein 54	TMEM54	1,18
interferon regulatory factor 1	IRF1	1,18
inositol polyphosphate-1-phosphatase	INPP1	1,18
PRAME family member 5	PRAMEF5	1,18
speedy/RINGO cell cycle regulator family member E6; speedy/RINGO cell cycle regulator family member E2	SPDYE6; SPDYE2	1,18
solute carrier family 25 (carnitine/acylcarnitine translocase), member 20	SLC25A20	1,18
nuclear export mediator factor	NEMF	1,18
Sad1 and UNC84 domain containing 3	SUN3	1,18
nucleotide binding protein-like	NUBPL	1,18
chromosome 1 open reading frame 127	C1orf127	1,18
golgin A6 family-like 9	GOLGA6L9	1,18
parathyroid hormone-like hormone	PTHLH	1,18
WAP four-disulfide core domain 1	WFDC1	1,18
F11 antisense RNA 1	F11-AS1	1,18
Memczak2013 ANTISENSE, CDS, coding, INTERNAL best transcript NM_014931	PPP6R1	1,18
TSC22 domain family, member 1	TSC22D1	1,18
tubulin, gamma complex associated protein 6	TUBGCP6	1,18
POTE ankyrin domain family, member J	POTEJ	1,18
synaptotagmin XIV	SYT14	1,18
troponin I type 3 (cardiac)	TNNI3	1,18
CCAAT/enhancer binding protein (C/EBP), beta	CEBPB	1,18
beclin 1, autophagy related	BECN1	1,18
cadherin-related family member 4	CDHR4	1,18
rhomboid, veinlet-like 1 (Drosophila)	RHBDL1	1,18
chromosome 20 open reading frame 194	C20orf194	1,18
hematopoietic prostaglandin D synthase	HPGDS	1,18
ankyrin repeat and EF-hand domain containing 1	ANKEF1	1,18
zinc finger, CCHC domain containing 4	ZCCHC4	1,18
poly(ADP-ribose) polymerase family member 11	PARP11	1,18
C-type lectin domain family 4, member F	CLEC4F	1,18
integrator complex subunit 4 pseudogene 2	INTS4P2	1,18
peroxisome proliferator-activated receptor alpha	PPARA	1,18
cytokine-dependent hematopoietic cell linker	CLNK	1,18
G protein-coupled receptor 148	GPR148	1,18
N-acylethanolamine acid amidase	NAAA	1,18
secretoglobin, family 2B, member 2	SCGB2B2	1,18
phosducin like 2	PDCL2	1,18
family with sequence similarity 13, member B	FAM13B	1,18
ST8 alpha-N-acetyl-neuraminide alpha-2,8-sialyltransferase 2	ST8SIA2	1,18
chromosome X open reading frame 58	CXorf58	1,18
general transcription factor IIH subunit 1	GTF2H1	1,18

meiosis/spermiogenesis associated 1	MEIG1	1,18
PRAME family member 6 [Source:HGNC Symbol;Acc:HGNC:30583]	PRAMEF6	1,18
PRAME family member 6 [Source:HGNC Symbol;Acc:HGNC:30583]	PRAMEF6	1,18
acyl-CoA binding domain containing 6	ACBD6	1,18
golgin A6 family-like 5, pseudogene; golgin A6 family-like 17, pseudogene	GOLGA6L5P; GOLGA6L17P	1,18
osteopetrosis associated transmembrane protein 1	OSTM1	1,18
zinc finger, FYVE domain containing 26	ZFYVE26	1,18
regulator of cell cycle	RGCC	1,18
DBF4 zinc finger B	DBF4B	1,18
toll-like receptor 5	TLR5	1,18
myocilin, trabecular meshwork inducible glucocorticoid response	MYOC	1,18
solute carrier family 16, member 9	SLC16A9	1,18
glycolipid transfer protein	GLTP	1,18
protein-O-mannosyltransferase 1	POMT1	1,18
calpain 12	CAPN12	1,18
crystallin beta-gamma domain containing 3	CRYBG3	1,18
relaxin 2	RLN2	1,18
EEF1E1-BLOC1S5 readthrough (NMD candidate)	EEF1E1-BLOC1S5	1,18
chondrolectin	CHODL	1,18
Transcript Identified by AceView, Entrez Gene ID(s) 22843	PPM1E	1,18
SH2 domain containing 6	SH2D6	1,18
TBC1 domain family member-like; TBC1 domain family, member 3D [Source:HGNC Symbol;Acc:HGNC:28944]	LOC101060389; TBC1D3D	1,18
hydroxysteroid (11-beta) dehydrogenase 1-like; ribosomal protein L36	HSD11B1L; RPL36	1,18
Transcript Identified by AceView, Entrez Gene ID(s) 123016	TTC8	1,18
Ly1 antibody reactive	LYAR	1,18
chromosome 15 open reading frame 48; microRNA 147b	C15orf48; MIR147B	1,18
NADH dehydrogenase (ubiquinone) complex I, assembly factor 1	NDUFAF1	1,18
CXXC finger protein 1	CXXC1	1,18
autophagy related 16-like 1	ATG16L1	1,18
Jeck2013 ALT_ACCEPTOR, ALT_DONOR, coding, INTERNAL, intronic best transcript NM_014629	ARHGEF10	1,18
F-box and WD repeat domain containing 2	FBXW2	1,18
dishevelled associated activator of morphogenesis 2	DAAM2	1,18
C-type lectin domain family 9, member A	CLEC9A	1,18
leukotriene B4 receptor; leukotriene B4 receptor 2	LTB4R; LTB4R2	1,18
transketolase-like 2	TKTL2	1,18
chromosome 14 open reading frame 169	C14orf169	1,18

nei-like DNA glycosylase 1	NEIL1	1,18
polymerase (RNA) III (DNA directed) polypeptide H (22.9kD)	POLR3H	1,18
claudin 34	CLDN34	1,18
glucocorticoid modulatory element binding protein 2	GMEB2	1,18
homeobox C8	HOXC8	1,18
keratin associated protein 12-2	KRTAP12-2	1,18
transient receptor potential cation channel, subfamily V, member 3	TRPV3	1,18
SET domain containing 1B	SETD1B	1,18
zinc finger protein 543	ZNF543	1,18
crumbs family member 3	CRB3	1,18
zinc finger and BTB domain containing 42	ZBTB42	1,18
variable charge, Y-linked; variable charge, Y-linked 1B	VCY; VCY1B	1,18
family with sequence similarity 157, member A	FAM157A	1,18
jumonji domain containing 4	JMJD4	1,18
CHKB-CPT1B readthrough (NMD candidate)	CHKB-CPT1B	1,18
cytochrome P450, family 4, subfamily F, polypeptide 12	CYP4F12	1,18
zinc finger protein 75D	ZNF75D	1,18
RAP1 GTPase activating protein 2	RAP1GAP2	1,18
adaptor-related protein complex 2, alpha 2 subunit	AP2A2	1,18
MAU2 sister chromatid cohesion factor	MAU2	1,18
SCAN domain containing 2 pseudogene	SCAND2P	1,18
calcium binding protein 39-like	CAB39L	1,18
testis expressed 15	TEX15	1,18
proline-rich protein BstNI subfamily 1	PRB1	1,18
collagen, type XII, alpha 1	COL12A1	1,18
Transcript Identified by AceView, Entrez Gene ID(s) 153562	MARVELD2	1,18
choline phosphotransferase 1	CHPT1	1,18
neuropeptide Y receptor Y5	NPY5R	1,18
claudin 14	CLDN14	1,18
syntrophin, gamma 2	SNTG2	1,18
RAB28, member RAS oncogene family	RAB28	1,18
failed axon connections homolog	FAXC	1,18
leptin	LEP	1,18
Memczak2013 ANTISENSE, CDS, coding, INTERNAL best transcript NM_133502	ZNF274	1,18
chromosome 2 open reading frame 15	C2orf15	1,18
transmembrane protein 183A	TMEM183A	1,18
olfactory receptor, family 56, subfamily B, member 4	OR56B4	1,18
IQ motif containing GTPase activating protein 3	IQGAP3	1,18
retinoblastoma binding protein 4	RBBP4	1,18
WW domain containing oxidoreductase	WWOX	1,18
v-maf avian musculoaponeurotic fibrosarcoma oncogene homolog B	MAFB	1,18
uracil phosphoribosyltransferase (FUR1) homolog (S. cerevisiae)	UPRT	1,18

gamma-aminobutyric acid (GABA) B receptor, 1	GABBR1	1,18
PRAME family member 11	PRAMEF11	1,18
RNA 3-terminal phosphate cyclase	RTCA	1,18
recombination signal binding protein for immunoglobulin kappa J region-like	RBPJL	1,18
carbonic anhydrase IV	CA4	1,18
family with sequence similarity 43, member B	FAM43B	1,18
zinc finger, DHHC-type containing 16	ZDHHC16	1,18
tetratricopeptide repeat domain 36	TTC36	1,18
tripartite motif containing 65	TRIM65	1,18
family with sequence similarity 21, member C	FAM21C	1,18
GRB2-associated binding protein family, member 4	GAB4	1,18
immunoglobulin lambda-like polypeptide 1	IGLL1	1,18
Hermansky-Pudlak syndrome 4	HPS4	1,18
URB1 ribosome biogenesis 1 homolog (S. cerevisiae)	URB1	1,18
CDKN2A interacting protein	CDKN2AIP	1,18
nuclear respiratory factor 1	NRF1	1,18
neuroguidin, EIF4E binding protein	NGDN	1,18
transmembrane protein 233	TMEM233	1,18
endogenous Bornavirus-like nucleoprotein 1	EBLN1	1,18
family with sequence similarity 134, member B	FAM134B	1,18
G protein-coupled receptor 33 (gene/pseudogene)	GPR33	1,18
cytochrome P450, family 27, subfamily C, polypeptide 1	CYP27C1	1,18
taxilin beta	TXLNB	1,18
KH homology domain containing 1-like	KHDC1L	1,18
PQ loop repeat containing 2-like	PQLC2L	1,18
chromosome 1 open reading frame 146	C1orf146	1,18
cystatin A (stefin A)	CSTA	1,18
cholinergic receptor, nicotinic gamma	CHRNG	1,18
transmembrane protein 74B	TMEM74B	1,18
family with sequence similarity 83, member C	FAM83C	1,18
RNA binding motif protein, X-linked-like 3	RBMXL3	1,18
membrane bound O-acyltransferase domain containing 1	MBOAT1	1,18
cortactin binding protein 2	CTTNBP2	1,18
coiled-coil domain containing 136	CCDC136	1,18
endogenous retrovirus group W, member 1	ERVW-1	1,18
jade family PHD finger 1	JADE1	1,18
Eukaryotic translation initiation factor 3 subunit F [Source:UniProtKB/Swiss-Prot;Acc:O00303]	EIF3F	1,18
minichromosome maintenance 9 homologous recombination repair factor	MCM9	1,18
zinc finger protein 322	ZNF322	1,18
syntaxin 8	STX8	1,18
neutrophil cytosolic factor 2	NCF2	1,18
solute carrier family 6, member 16	SLC6A16	1,18

toll-like receptor 7	TLR7	1,18
interferon-induced protein 35	IFI35	1,18
long intergenic non-protein coding RNA 656	LINC00656	1,18
POTE ankyrin domain family, member I	POTEI	1,18
chromosome 21 open reading frame 2	C21orf2	1,18
Transcript Identified by AceView, Entrez Gene ID(s) 4731	NDUFV3	1,18
migration and invasion enhancer 1	MIEN1	1,18
potassium channel tetramerization domain containing 18	KCTD18	1,18
exocyst complex component 6B	EXOC6B	1,18
fibrillin 2	FBN2	1,18
transcription elongation factor A (SII)-like 1	TCEAL1	1,18
mediator complex subunit 8	MED8	1,18
glutamate-cysteine ligase, catalytic subunit	GCLC	1,18
fibroblast growth factor 6	FGF6	1,18
CDC like kinase 4	CLK4	1,18
N-acetylated alpha-linked acidic dipeptidase-like 2	NAALADL2	1,18
chromosome 20 open reading frame 144	C20orf144	1,18
elaC ribonuclease Z 1	ELAC1	1,18
cathepsin L family member 3, pseudogene	CTSL3P	1,18
grainyhead-like transcription factor 1	GRHL1	1,18
SLAM family member 7	SLAMF7	1,18
dynein, cytoplasmic 1, heavy chain 1	DYNC1H1	1,18
death-associated protein kinase 2	DAPK2	1,18
PRAME family member 5	PRAMEF5	1,18
adenomatosis polyposis coli 2	APC2	1,18
sema domain, immunoglobulin domain (Ig), transmembrane domain (TM) and short cytoplasmic domain, (semaphorin) 4B	SEMA4B	1,18
olfactory receptor, family 1, subfamily D, member 5	OR1D5	1,18
family with sequence similarity 71, member C	FAM71C	1,18
Memczak2013 ALT_ACCEPTOR, ALT_DONOR, coding, INTERNAL, intronic best transcript NM_181558	RFC3	1,18
AMMECR1 like	AMMECR1L	1,18
drebrin 1	DBN1	1,18
ubiquitin specific peptidase 17-like family member 1	USP17L1	1,18
KIAA0907; small Cajal body-specific RNA 4	KIAA0907; SCARNA4	1,18
olfactory receptor, family 52, subfamily W, member 1	OR52W1	1,18
checkpoint kinase 2	CHEK2	1,18
dehydrogenase/reductase (SDR family) member 7	DHRS7	1,18
transmembrane and coiled-coil domains 2	TMCO2	1,18
adenylate kinase 9	AK9	1,18
N-deacetylase/N-sulfotransferase (heparan glucosaminyl) 1	NDST1	1,18
Transcript Identified by AceView, Entrez Gene ID(s) 9107	MTMR6	1,18
Rho GTPase activating protein 28	ARHGAP28	1,18
orthopedia homeobox	OTP	1,18

FERM and PDZ domain containing 1	FRMPD1	1,18
ATPase, Na+/K+ transporting, alpha 1 polypeptide	ATP1A1	1,18
Ras association (RalGDS/AF-6) and pleckstrin homology domains 1	RAPH1	1,18
zinc binding alcohol dehydrogenase domain containing 2	ZADH2	1,18
acid sensing ion channel 1	ASIC1	1,18
upstream binding transcription factor, RNA polymerase I	UBTF	1,18
EP300 interacting inhibitor of differentiation 2	EID2	1,18
alkaline ceramidase 2	ACER2	1,18
ChaC, cation transport regulator homolog 2 (E. coli)	CHAC2	1,18
poly(rC) binding protein 3	PCBP3	1,18
ets variant 6	ETV6	1,18
leupaxin	LPXN	1,18
thyroid hormone responsive	THRSP	1,18
coiled-coil domain containing 113	CCDC113	1,18
PRAME family member 18	PRAMEF18	1,18
SID1 transmembrane family, member 1	SIDT1	1,18
chromosome 17 open reading frame 105	C17orf105	1,18
Memczak2013 ALT_ACCEPTOR, ALT_DONOR, coding, INTERNAL, intronic best transcript NM_001164162	PPP6R3	1,18
Memczak2013 ANTISENSE, CDS, coding, INTERNAL best transcript NM_001174167	SYK	1,18
ubiquitin specific peptidase 17-like family member 23 [Source:HGNC Symbol;Acc:HGNC:44451]	USP17L23	1,18
tsukushi, small leucine rich proteoglycan	TSKU	1,18
thioredoxin domain containing 16	TXNDC16	1,18
potassium channel, voltage gated Shal related subfamily D, member 3	KCND3	1,18
GPR158 antisense RNA 1	GPR158-AS1	1,18
gamma-glutamylcyclotransferase	GGCT	1,18
ring finger protein 217	RNF217	1,18
sorbin and SH3 domain containing 2	SORBS2	1,17
piezo-type mechanosensitive ion channel component 2	PIEZO2	1,17
zona pellucida-like domain containing 1	ZPLD1	1,17
unc-45 myosin chaperone B	UNC45B	1,17
late cornified envelope 1B	LCE1B	1,17
Transcript Identified by AceView, Entrez Gene ID(s) 342184	FMN1	1,17
keratin associated protein 16-1	KRTAP16-1	1,17
phosphatidylinositol-5-phosphate 4-kinase, type II, alpha	PIP4K2A	1,17
methyl-CpG binding domain protein 5	MBD5	1,17
dermatan sulfate epimerase-like	DSEL	1,17
membrane bound O-acyltransferase domain containing 2	MBOAT2	1,17
adenylate cyclase 3	ADCY3	1,17
anterior gradient 2, protein disulphide isomerase family member	AGR2	1,17

potassium channel, inwardly rectifying subfamily J, member 8	KCNJ8	1,17
betaine--homocysteine S-methyltransferase 2	BHMT2	1,17
neurolysin (metallopeptidase M3 family)	NLN	1,17
paxillin	PXN	1,17
olfactory receptor, family 5, subfamily B, member 3	OR5B3	1,17
coiled-coil domain containing 59	CCDC59	1,17
dedicator of cytokinesis 4	DOCK4	1,17
chromosome 1 open reading frame 106	C1orf106	1,17
chromosome 10 open reading frame 128	C10orf128	1,17
NOP2/Sun domain family, member 6	NSUN6	1,17
claudin 19	CLDN19	1,17
uridine phosphorylase 1	UPP1	1,17
apolipoprotein B mRNA editing enzyme, catalytic polypeptide-like 2	APOBEC2	1,17
Jeck2013 ANTISENSE, CDS, coding, INTERNAL, OVCODE, OVEXON best transcript NM_001110556	FLNA	1,17
cyclin-dependent kinase-like 4	CDKL4	1,17
dopachrome tautomerase	DCT	1,17
phenylethanolamine N-methyltransferase	PNMT	1,17
STEAP family member 3, metalloreductase	STEAP3	1,17
double homeobox 4 like 7	DUX4L7	1,17
serpin peptidase inhibitor, clade I (neuroserpin), member 1	SERPINI1	1,17
alcohol dehydrogenase 1C (class I), gamma polypeptide	ADH1C	1,17
protein kinase, AMP-activated, gamma 1 non-catalytic subunit	PRKAG1	1,17
ISY1-RAB43 readthrough	ISY1-RAB43	1,17
neuroblastoma breakpoint family, member 9	NBPF9	1,17
unc-51 like kinase 3	ULK3	1,17
long intergenic non-protein coding RNA 1359	LINC01359	1,17
GTP-binding protein 8 (putative)	GTPBP8	1,17
RIMS binding protein 3C; RIMS binding protein 3B	RIMBP3C; RIMBP3B	1,17
putative deoxyuridine 5-triphosphate nucleotidohydrolase-like protein FLJ16323; novel transcript; Transcript Identified by AceView	LOC100506422; RP11-80I3.1; tayamu	1,17
stonin 2	STON2	1,17
zinc finger protein 736	ZNF736	1,17
solute carrier family 12 (potassium/chloride transporter), member 7	SLC12A7	1,17
tandem C2 domains, nuclear	TC2N	1,17
H6 family homeobox 1	HMX1	1,17
olfactory receptor, family 52, subfamily Z, member 1 (gene/pseudogene)	OR52Z1	1,17
isthmin 1, angiogenesis inhibitor	ISM1	1,17
trophinin	TRO	1,17
sperm associated antigen 7	SPAG7	1,17

caspase 5	CASP5	1,17
RALBP1 associated Eps domain containing 2	REPS2	1,17
peroxisomal, testis specific 1	PXT1	1,17
mucin 13, cell surface associated	MUC13	1,17
S-antigen; retina and pineal gland (arrestin)	SAG	1,17
N-ethylmaleimide-sensitive factor attachment protein, beta	NAPB	1,17
LYR motif containing 4	LYRM4	1,17
joining chain of multimeric IgA and IgM	JCHAIN	1,17
phosphatidylinositol glycan anchor biosynthesis class L	PIGL	1,17
Homo sapiens acetylserotonin O-methyltransferase (ASMT), transcript variant 2, mRNA.	ASMT	1,17
kelch domain containing 7A	KLHDC7A	1,17
KIAA1671	KIAA1671	1,17
calmin (calponin-like, transmembrane)	CLMN	1,17
olfactory receptor, family 4, subfamily K, member 15	OR4K15	1,17
AHNAK nucleoprotein 2	AHNAK2	1,17
undifferentiated embryonic cell transcription factor 1	UTF1	1,17
family with sequence similarity 212, member B	FAM212B	1,17
ectopic P-granules autophagy protein 5 homolog (C. elegans)	EPG5	1,17
CCR4-NOT transcription complex subunit 10	CNOT10	1,17
chromosome 22 open reading frame 23	C22orf23	1,17
hexokinase 3 (white cell)	HK3	1,17
hook microtubule-tethering protein 1	HOOK1	1,17
epithelial membrane protein 3	EMP3	1,17
myelin associated glycoprotein	MAG	1,17
Zhang2013 ALT_ACCEPTOR, ALT_DONOR, coding, INTERNAL, intronic best transcript NM_000976	RPL12	1,17
INO80 complex subunit D	INO80D	1,17
IQ motif containing J; schwannomin interacting protein 1; IQCJ-SCHIP1 readthrough	IQCJ; SCHIP1; IQCJ-SCHIP1	1,17
thiamine triphosphatase	THTPA	1,17
testis expressed 37	TEX37	1,17
PHD finger protein 6	PHF6	1,17
GCN1 eIF2 alpha kinase activator homolog; microRNA 4498	GCN1; MIR4498	1,17
leukocyte immunoglobulin-like receptor, subfamily A (with TM domain), member 2	LILRA2	1,17
folate receptor 2 (fetal)	FOLR2	1,17
cytochrome P450, family 21, subfamily A, polypeptide 2	CYP21A2	1,17
endoplasmic reticulum protein 29	ERP29	1,17
adaptor-related protein complex 2 sigma 1 subunit	AP2S1	1,17
transmembrane protein 63A	TMEM63A	1,17
microseminoprotein, beta-	MSMB	1,17
neurexin 1	NRXN1	1,17
tenomodulin	TNMD	1,17

SLX4 interacting protein	SLX4IP	1,17
fibroblast growth factor 23	FGF23	1,17
small nuclear ribonucleoprotein, U1 70kDa subunit	SNRNP70	1,17
mitogen-activated protein kinase kinase kinase 13	MAP3K13	1,17
serine-rich single-pass membrane protein 1	SSMEM1	1,17
ring finger protein 8, E3 ubiquitin protein ligase	RNF8	1,17
SAP domain containing ribonucleoprotein	SARNP	1,17
Memczak2013 ANTISENSE, CDS, coding, INTERNAL, intronic, UTR3 best transcript NM_203305	FAM102A	1,17
solute carrier organic anion transporter family, member 1C1	SLCO1C1	1,17
NOP14 nucleolar protein	NOP14	1,17
ubiquitin specific peptidase 17-like family member 15	USP17L15	1,17
zinc finger protein 619	ZNF619	1,17
Memczak2013 ANTISENSE, CDS, coding, INTERNAL best transcript NM_002265	KPNB1	1,17
meteorin, glial cell differentiation regulator-like	METRNL	1,17
olfactory receptor, family 2, subfamily T, member 35	OR2T35	1,17
coiled-coil domain containing 51	CCDC51	1,17
leucine-rich repeats and transmembrane domains 1	LRTM1	1,17
NCK-associated protein 5-like	NCKAP5L	1,17
thymosin beta 4, Y-linked	TMSB4Y	1,17
GINS complex subunit 4 (Sld5 homolog)	GINS4	1,17
opioid binding protein/cell adhesion molecule-like	OPCML	1,17
TBC1 domain family, member 19	TBC1D19	1,17
artemin	ARTN	1,17
Cdc42 guanine nucleotide exchange factor 9; ARHGEF9 intronic transcript 1	ARHGEF9; ARHGEF9-IT1	1,17
X-linked Kx blood group related, Y-linked 2; X-linked Kx blood group related, Y-linked	XKRY2; XKRY	1,17
vestigial-like family member 4	VGLL4	1,17
discoidin domain receptor tyrosine kinase 2	DDR2	1,17
guanine nucleotide binding protein (G protein), alpha z polypeptide	GNAZ	1,17
migration and invasion inhibitory protein	MIIP	1,17
zinc finger protein 865	ZNF865	1,17
D4, zinc and double PHD fingers, family 3	DPF3	1,17
membrane metallo-endopeptidase-like 1	MMEL1	1,17
ATPase, H+ transporting, lysosomal accessory protein 1-like	ATP6AP1L	1,17
transmembrane protein 155	TMEM155	1,17
death-domain associated protein	DAXX	1,17
F-box protein 25	FBXO25	1,17
heat shock 27kDa protein family, member 7 (cardiovascular)	HSPB7	1,17
holocarboxylase synthetase (biotin-(proprionyl-CoA-carboxylase (ATP-hydrolysing)) ligase)	HLCS	1,17
M-phase specific PLK1 interacting protein	MPLKIP	1,17

actin-related protein T3	ACTRT3	1,17
chromosome 16 open reading frame 86	C16orf86	1,17
charged multivesicular body protein 6	CHMP6	1,17
BUD13 homolog	BUD13	1,17
surfactant protein D	SFTPD	1,17
microtubule associated monooxygenase, calponin and LIM domain containing 2	MICAL2	1,17
protein tyrosine phosphatase type IVA, member 3	PTP4A3	1,17
Transcript Identified by AceView, Entrez Gene ID(s) 84620	ST6GAL2	1,17
transglutaminase 6	TGM6	1,17
variable charge, Y-linked 1B	VCY1B	1,17
potassium channel, voltage gated shaker related subfamily A, member 3	KCNA3	1,17
breakpoint cluster region	BCR	1,17
stress-associated endoplasmic reticulum protein family member 2	SERP2	1,17
bone morphogenetic protein 10	BMP10	1,17
retinol binding protein 1, cellular	RBP1	1,17
chromosome 2 open reading frame 71	C2orf71	1,17
solute carrier family 41, member 3	SLC41A3	1,17
malate dehydrogenase 1B	MDH1B	1,17
GRAM domain containing 1B	GRAMD1B	1,17
acyl-CoA synthetase bubblegum family member 1	ACSBG1	1,17
olfactory receptor, family 51, subfamily B, member 4	OR51B4	1,17
glucagon-like peptide 1 receptor	GLP1R	1,17
Harvey rat sarcoma viral oncogene homolog	HRAS	1,17
D-aspartate oxidase	DDO	1,17
chorionic somatomammotropin hormone 2	CSH2	1,17
oxoglutarate dehydrogenase-like	OGDHL	1,17
olfactory receptor, family 5, subfamily M, member 3	OR5M3	1,17
ESX homeobox 1	ESX1	1,17
coiled-coil domain containing 8	CCDC8	1,17
SECIS binding protein 2-like	SECISBP2L	1,17
MORC family CW-type zinc finger 4	MORC4	1,17
THAP domain containing 7	THAP7	1,17
acyl-CoA synthetase short-chain family member 3	ACSS3	1,17
MRGPRG antisense RNA 1	MRGPRG-AS1	1,17
keratin associated protein 5-10	KRTAP5-10	1,17
pyrroline-5-carboxylate reductase-like	PYCRL	1,17
exonuclease 3-5 domain containing 3	EXD3	1,17
CD74 molecule, major histocompatibility complex, class II invariant chain	CD74	1,17
myomesin 3	MYOM3	1,17
RAS-like, estrogen-regulated, growth inhibitor	RERG	1,17
eukaryotic translation initiation factor 3, subunit K	EIF3K	1,17

cancer/testis antigen family 45, member A10	CT45A10	1,17
long intergenic non-protein coding RNA 483	LINC00483	1,17
glutamate receptor, ionotropic, AMPA 3	GRIA3	1,17
ecdysoneless homolog (Drosophila)	ECD	1,17
N-acetyltransferase 2 (arylamine N-acetyltransferase)	NAT2	1,17
transmembrane protein 38B	TMEM38B	1,17
solute carrier family 6 (neurotransmitter transporter), member 6	SLC6A6	1,17
OTU deubiquitinase, ubiquitin aldehyde binding 2	OTUB2	1,17
uncharacterized LOC100506388; Transcript Identified by AceView; novel transcript, atisense to RPH3AL	LOC100506388; RP11-1260E13.4; hosaru	1,17
methyltransferase like 8	METTL8	1,17
unc-5 family C-terminal like	UNC5CL	1,17
family with sequence similarity 71, member D	FAM71D	1,17
periostin, osteoblast specific factor	POSTN	1,17
major histocompatibility complex, class II, DQ alpha 1	HLA-DQA1	1,17
coiled-coil domain containing 110	CCDC110	1,17
family with sequence similarity 161, member B	FAM161B	1,17
TBC1 domain family, member 9 (with GRAM domain)	TBC1D9	1,17
uncharacterized LOC101928093; novel transcript	LOC101928093; RP11-779O18.1	1,17
chromodomain protein, Y-like 2	CDYL2	1,17
olfactory receptor, family 8, subfamily B, member 2	OR8B2	1,17
nitric oxide synthase 3 (endothelial cell)	NOS3	1,17
NADPH oxidase, EF-hand calcium binding domain 5; sperm equatorial segment protein 1	NOX5; SPESP1	1,17
coiled-coil domain containing 88A	CCDC88A	1,17
protease, serine, 16 (thymus)	PRSS16	1,17
synaptosome associated protein 47kDa	SNAP47	1,17
REX4 homolog, 3-5 exonuclease	REXO4	1,17
SPT5 homolog, DSIF elongation factor subunit	SUPT5H	1,17
prefoldin subunit 1	PFDN1	1,17
small proline-rich protein 2B	SPRR2B	1,17
chromosome 12 open reading frame 40	C12orf40	1,17
centrin 1	CETN1	1,17
secreted frizzled-related protein 1	SFRP1	1,17
T-cell immunoglobulin and mucin domain containing 4	TIMD4	1,17
receptor (chemosensory) transporter protein 1	RTP1	1,17
KIAA0100	KIAA0100	1,17
nucleophosmin/nucleoplasmin 2	NPM2	1,17
small G protein signaling modulator 2	SGSM2	1,17
haloacid dehalogenase-like hydrolase domain containing 2	HDHD2	1,17

fibrous sheath interacting protein 2	FSIP2	1,17
mannose receptor, C type 1	MRC1	1,17
zinc finger protein 197	ZNF197	1,17
fibronectin type III and ankyrin repeat domains 1	FANK1	1,17
Kruppel-like factor 11	KLF11	1,17
VPS11, CORVET/HOPS core subunit [Source:HGNC Symbol;Acc:HGNC:14583]	VPS11	1,17
proteasome subunit alpha 1	PSMA1	1,17
transmembrane inner ear	TMIE	1,17
B-cell CLL/lymphoma 7A	BCL7A	1,17
NME/NM23 family member 7	NME7	1,17
solute carrier family 12, member 9	SLC12A9	1,17
solute carrier organic anion transporter family, member 5A1	SLCO5A1	1,17
prion protein 2 (dublet)	PRND	1,17
trinucleotide repeat containing 6B	TNRC6B	1,17
splA/ryanodine receptor domain and SOCS box containing 1	SPSB1	1,17
lamin tail domain containing 2	LMNTD2	1,17
apelin receptor early endogenous ligand	APELA	1,17
chromodomain helicase DNA binding protein 8	CHD8	1,17
Rho guanine nucleotide exchange factor 15	ARHGEF15	1,17
fragile histidine triad	FHIT	1,17
BAALC antisense RNA 2	BAALC-AS2	1,17
proteasome subunit beta 3	PSMB3	1,17
bromodomain containing 8	BRD8	1,17
chromosome 19 open reading frame 71	C19orf71	1,17
zinc finger and BTB domain containing 41	ZBTB41	1,17
Jeck2013 ANTISENSE, coding, INTERNAL, OVEXON, UTR3 best transcript NM_001007098	SCP2	1,17
AF4/FMR2 family, member 3	AFF3	1,17
phospholipase A1 member A	PLA1A	1,17
olfactory receptor, family 1, subfamily J, member 2	OR1J2	1,17
zinc finger protein 526	ZNF526	1,17
Transcript Identified by AceView, Entrez Gene ID(s) 8502	PKP4	1,17
family with sequence similarity 26, member D	FAM26D	1,17
oleoyl-ACP hydrolase	OLAH	1,17
uncharacterized LOC100130880; novel transcript	LOC100130880; AC022173.2	1,17
K(lysine) acetyltransferase 7	KAT7	1,17
zinc finger protein 660	ZNF660	1,17
kallikrein related peptidase 15	KLK15	1,17
killer cell lectin-like receptor subfamily F, member 1	KLRF1	1,17
RAD52 homolog, DNA repair protein	RAD52	1,17
solute carrier family 39 (zinc transporter), member 3	SLC39A3	1,17
vasoactive intestinal peptide receptor 1	VIPR1	1,17
interferon stimulated exonuclease gene 20kDa like 2	ISG20L2	1,17

ubiquitin-conjugating enzyme E2D 4 (putative)	UBE2D4	1,17
family with sequence similarity 182, member B	FAM182B	1,17
small proline-rich protein 2E	SPRR2E	1,17
zinc finger protein 544	ZNF544	1,17
dCMP deaminase	DCTD	1,17
coiled-coil domain containing 144 family, N-terminal like	CCDC144NL	1,17
elaC ribonuclease Z 2	ELAC2	1,17
BLOC1S1-RDH5 readthrough; Uncharacterized protein [Source:UniProtKB/TrEMBL;Acc:F8VNQ1]	BLOC1S1-RDH5; RP11-644F5.10	1,17
ubiquitin-like 7	UBL7	1,17
zinc finger, DHHC-type containing 11B	ZDHHC11B	1,17
polymerase (DNA directed), epsilon, catalytic subunit	POLE	1,17
tektin 4	TEKT4	1,17
chromobox homolog 4	CBX4	1,17
solute carrier family 17 (vesicular glutamate transporter), member 6	SLC17A6	1,17
dihydrouridine synthase 3-like	DUS3L	1,17
Zhang2013 ALT_ACCEPTOR, ALT_DONOR, coding, INTERNAL, intronic best transcript NM_032737	LMNB2	1,17
tetratricopeptide repeat domain 9C	TTC9C	1,17
glycoprotein M6B	GPM6B	1,17
tumor necrosis factor (ligand) superfamily, member 18	TNFSF18	1,17
olfactory receptor, family 13, subfamily J, member 1	OR13J1	1,17
tripartite motif containing 3	TRIM3	1,17
chromosome 7 open reading frame 73	C7orf73	1,17
Transcript Identified by AceView, Entrez Gene ID(s) 7486	WRN	1,17
short chain dehydrogenase/reductase family 39U, member 1	SDR39U1	1,17
chemokine (C-C motif) ligand 4-like 2; chemokine (C-C motif) ligand 4-like 1	CCL4L2; CCL4L1	1,16
FAST kinase domains 2	FASTKD2	1,16
DEAD (Asp-Glu-Ala-Asp) box polypeptide 4	DDX4	1,16
IQ motif containing F1	IQCF1	1,16
polymerase (RNA) III (DNA directed) polypeptide G (32kD)-like	POLR3GL	1,16
Src homology 2 domain containing E	SHE	1,16
SMG8 nonsense mediated mRNA decay factor	SMG8	1,16
membrane-spanning 4-domains, subfamily A, member 8	MS4A8	1,16
calcium activated nucleotidase 1	CANT1	1,16
RAP1 GTPase activating protein	RAP1GAP	1,16
nicotinamide nucleotide transhydrogenase	NNT	1,16
forkhead box I1	FOXI1	1,16
kelch domain containing 9	KLHDC9	1,16
serine peptidase inhibitor, Kazal type 14 (putative)	SPINK14	1,16
coiled-coil domain containing 130	CCDC130	1,16
fission, mitochondrial 1	FIS1	1,16

exportin 4	XPO4	1,16
polymerase (RNA) I polypeptide C	POLR1C	1,16
suppressor of cytokine signaling 5	SOCS5	1,16
neuro-oncological ventral antigen 2	NOVA2	1,16
oxysterol binding protein-like 11	OSBPL11	1,16
chromosome 1 open reading frame 116	C1orf116	1,16
Fc fragment of IgG, high affinity Ib, receptor (CD64)	FCGR1B	1,16
trafficking protein particle complex 9	TRAPPC9	1,16
meiosis specific with coiled-coil domain	MEIOC	1,16
fibrinogen beta chain	FGB	1,16
preferentially expressed antigen in melanoma	PRAME	1,16
SKI family transcriptional corepressor 1	SKOR1	1,16
F-box protein 22	FBXO22	1,16
bridging integrator 2	BIN2	1,16
nuclear pore complex interacting protein family, member B5	NPIPB5	1,16
single-minded family bHLH transcription factor 1	SIM1	1,16
family with sequence similarity 72, member D	FAM72D	1,16
forkhead box J2	FOXJ2	1,16
zinc finger, FYVE domain containing 27	ZFYVE27	1,16
potassium channel, inwardly rectifying subfamily J, member 1	KCNJ1	1,16
integrator complex subunit 3	INTS3	1,16
cancer/testis antigen 1B; cancer/testis antigen 1A	CTAG1B; CTAG1A	1,16
fibrillarin	FBL	1,16
aquaporin 12B	AQP12B	1,16
Homo sapiens uncharacterized LOC388692 (LOC388692), long non-coding RNA.; Homo sapiens family with sequence similarity 231, member D (FAM231D), long non-coding RNA.; novel transcript	LOC388692; FAM231D; RP11-403I13.7; RP11-403I13.8	1,16
late cornified envelope 2A	LCE2A	1,16
methyltransferase like 21C	METTL21C	1,16
ubiquitin specific peptidase 13 (isopeptidase T-3)	USP13	1,16
armadillo repeat containing 7	ARMC7	1,16
phosphoglycerate dehydrogenase	PHGDH	1,16
Zhang2013 ALT_ACCEPTOR, ALT_DONOR, coding, INTERNAL, intronic best transcript NM_014675	CROCC	1,16
ATPase, class V, type 10D	ATP10D	1,16
phospholipase C, delta 4	PLCD4	1,16
kelch-like family member 13	KLHL13	1,16
SRY box 30	SOX30	1,16
DnaJ (Hsp40) homolog, subfamily C, member 7	DNAJC7	1,16
transmembrane protein 52B	TMEM52B	1,16
zinc finger protein 706	ZNF706	1,16
LYN proto-oncogene, Src family tyrosine kinase	LYN	1,16
sorting nexin 31	SNX31	1,16

cytosolic thiouridylase subunit 2 homolog (S. pombe)	CTU2	1,16
Nipped-B homolog (Drosophila)	NIPBL	1,16
spectrin, beta, non-erythrocytic 5	SPTBN5	1,16
sulfatase 2	SULF2	1,16
mitochondrial coiled-coil domain 1	MCCD1	1,16
gamma-glutamyltransferase 1	GGT1	1,16
DEAH (Asp-Glu-Ala-His) box helicase 30	DHX30	1,16
proline rich 22	PRR22	1,16
phosphatidylinositol glycan anchor biosynthesis class Y; PIGY upstream reading frame	PIGY; PYURF	1,16
ST6 (alpha-N-acetyl-neuraminyl-2,3-beta-galactosyl-1,3)-N-acetylgalactosaminide alpha-2,6-sialyltransferase 2	ST6GALNAC2	1,16
glutathione peroxidase 2	GPX2	1,16
histocompatibility (minor) HB-1	HMHB1	1,16
solute carrier family 25 (pyrimidine nucleotide carrier), member 33	SLC25A33	1,16
Transcript Identified by AceView, Entrez Gene ID(s) 7073	TIAL1	1,16
leucine rich repeat containing 4B	LRRC4B	1,16
fatty acid binding protein 9, testis	FABP9	1,16
odd-skipped related transciption factor 2	OSR2	1,16
complement component (3b/4b) receptor 1 (Knops blood group)	CR1	1,16
polo-like kinase 4	PLK4	1,16
potassium channel tetramerization domain containing 13	KCTD13	1,16
zinc finger protein 35	ZNF35	1,16
adhesion G protein-coupled receptor L1	ADGRL1	1,16
mitochondrial ribosomal protein L54	MRPL54	1,16
RUSC1 antisense RNA 1	RUSC1-AS1	1,16
Transcript Identified by AceView, Entrez Gene ID(s) 25788	RAD54B	1,16
coagulation factor XIII, B polypeptide	F13B	1,16
B-cell CLL/lymphoma 9-like	BCL9L	1,16
serum response factor binding protein 1	SRFBP1	1,16
Transcript Identified by AceView, Entrez Gene ID(s) 5126	PCSK2	1,16
Memczak2013 ANTISENSE, coding, INTERNAL, intronic best transcript NM_022157	RRAGC	1,16
keratin associated protein 13-4	KRTAP13-4	1,16
mastermind-like domain containing 1	MAMLD1	1,16
centlein, centrosomal protein	CNTLN	1,16
ADAM metallopeptidase with thrombospondin type 1 motif 16	ADAMTS16	1,16
receptor tyrosine kinase-like orphan receptor 1	ROR1	1,16
UBE2F-SCLY readthrough (NMD candidate)	UBE2F-SCLY	1,16
histidine rich calcium binding protein	HRC	1,16
calmodulin-lysine N-methyltransferase	CAMKMT	1,16
serine incorporator 5	SERINC5	1,16
HECT and RLD domain containing E3 ubiquitin protein ligase 4	HERC4	1,16
tetratricopeptide repeat domain 13	TTC13	1,16

hypoxia up-regulated 1	HYOU1	1,16
nuclear pore complex interacting protein family, member B15	NPIPB15	1,16
TNF receptor-associated factor 5	TRAF5	1,16
zinc finger protein 300	ZNF300	1,16
chromosome 19 open reading frame 45	C19orf45	1,16
twist family bHLH transcription factor 2	TWIST2	1,16
solute carrier organic anion transporter family, member 2B1	SLCO2B1	1,16
low density lipoprotein receptor class A domain containing 4	LDLRAD4	1,16
MAP/microtubule affinity-regulating kinase 4	MARK4	1,16
endothelin 3	EDN3	1,16
G protein-coupled receptor 45	GPR45	1,16
deleted in lung and esophageal cancer 1	DLEC1	1,16
dual oxidase maturation factor 1	DUOXA1	1,16
transcription factor 4	TCF4	1,16
hemicentin 2	HMCN2	1,16
phosphodiesterase 6A, cGMP-specific, rod, alpha	PDE6A	1,16
glutamate receptor, ionotropic, N-methyl D-aspartate 2A	GRIN2A	1,16
taperin	TPRN	1,16
ELMO/CED-12 domain containing 3	ELMOD3	1,16
gap junction protein alpha 4	GJA4	1,16
fibronectin type III domain containing 8	FNDC8	1,16
retinol binding protein 5, cellular	RBP5	1,16
inositol polyphosphate-5-phosphatase B	INPP5B	1,16
kelch-like family member 28	KLHL28	1,16
GRB2-associated binding protein 2	GAB2	1,16
diaphanous-related formin 1	DIAPH1	1,16
peroxisome proliferator-activated receptor gamma, coactivator 1 alpha	PPARGC1A	1,16
Transcript Identified by AceView, Entrez Gene ID(s) 9402; putative novel transcript	RP3-370M22.8; GRAP2	1,16
transient receptor potential cation channel, subfamily C, member 2-like; Salzman2013 ANNOTATED, INTERNAL, ncRNA, OVEXON best transcript NR_029192; Transcript Identified by AceView, Entrez Gene ID(s) 100133315, RefSeq ID(s) NR_029192; Transcript Identified by AceView, Entrez Gene ID(s) 100133315; Putative short transient receptor potential channel 2-like protein [Source:UniProtKB/Swiss-Prot;Acc:Q6ZNB5]	LOC100133315; RP11-849H4.2	1,16
PSMG3 antisense RNA 1 (head to head)	PSMG3-AS1	1,16
chromosome 10 open reading frame 67	C10orf67	1,16
laminin, beta 4	LAMB4	1,16
KIAA0141	KIAA0141	1,16
MSANTD3-TMEFF1 readthrough	MSANTD3-TMEFF1	1,16
sialidase 1 (lysosomal sialidase)	NEU1	1,16

coagulation factor VII (serum prothrombin conversion accelerator)	F7	1,16
testis specific 10 interacting protein	TSGA10IP	1,16
olfactory receptor, family 51, subfamily A, member 7	OR51A7	1,16
coiled-coil-helix-coiled-coil-helix domain containing 4	CHCHD4	1,16
glutamate receptor, ionotropic, kainate 1	GRIK1	1,16
huntingtin interacting protein M	HYPM	1,16
stromal antigen 3-like 3 (pseudogene); stromal antigen 3-like 2 (pseudogene)	STAG3L3; STAG3L2	1,16
myotubularin related protein 10	MTMR10	1,16
zinc finger protein 843	ZNF843	1,16
vesicle associated membrane protein 2	VAMP2	1,16
NOBOX oogenesis homeobox	NOBOX	1,16
Transcript Identified by AceView, Entrez Gene ID(s) 2326	FMO1	1,16
caprin family member 2	CAPRIN2	1,16
protein kinase C, iota	PRKCI	1,16
5-hydroxytryptamine (serotonin) receptor 1B, G protein-coupled	HTR1B	1,16
SAS-6 centriolar assembly protein	SASS6	1,16
chromosome 10 open reading frame 91	C10orf91	1,16
uncharacterized LOC100287225; novel transcript	LOC100287225; RP11-267C16.1	1,16
cylicin, basic protein of sperm head cytoskeleton 1	CYLC1	1,16
HAUS augmin like complex subunit 8	HAUS8	1,16
paired-like homeodomain 2	PITX2	1,16
RAS guanyl releasing protein 2 (calcium and DAG-regulated)	RASGRP2	1,16
cilia and flagella associated protein 61	CFAP61	1,16
tumor necrosis factor receptor superfamily, member 1B; microRNA 4632; microRNA 7846	TNFRSF1B; MIR4632; MIR7846	1,16
serine/arginine repetitive matrix 5	SRRM5	1,16
tweety family member 2	TTYH2	1,16
keratin 36, type I	KRT36	1,16
NFKB inhibitor interacting Ras-like 1	NKIRAS1	1,16
adiponectin receptor 2	ADIPOR2	1,16
golgin A8 family, member A; golgin A8 family, member B	GOLGA8A; GOLGA8B	1,16
BCL2/adenovirus E1B 19kDa interacting protein 2	BNIP2	1,16
hypoxia inducible factor 1, alpha subunit inhibitor	HIF1AN	1,16
F-box protein 2	FBXO2	1,16
5-hydroxytryptamine (serotonin) receptor 1F, G protein-coupled	HTR1F	1,16
follicle stimulating hormone, beta polypeptide	FSHB	1,16
mitochondria localized glutamic acid rich protein	MGARP	1,16
sparc/osteonectin, cwcv and kazal-like domains proteoglycan (testican) 2	SPOCK2	1,16

podoplanin	PDPN	1,16
ATR serine/threonine kinase	ATR	1,16
long intergenic non-protein coding RNA 336	LINC00336	1,16
chymotrypsin C (caldecrin)	CTRC	1,16
chromosome transmission fidelity factor 8	CHTF8	1,16
LIM homeobox 1	LHX1	1,16
glycine dehydrogenase (decarboxylating)	GLDC	1,16
family with sequence similarity 157, member B	FAM157B	1,16
zinc finger protein 862	ZNF862	1,16
solute carrier family 17, member 2	SLC17A2	1,16
hepatocyte growth factor-regulated tyrosine kinase substrate	HGS	1,16
ATPase, H+ transporting, lysosomal 42kDa, V1 subunit C2	ATP6V1C2	1,16
dynein, light chain, roadblock-type 1	DYNLRB1	1,16
inositol polyphosphate-5-phosphatase A	INPP5A	1,16
paraneoplastic Ma antigen family member 6A	PNMA6A	1,16
SPATA31 subfamily A, member 1	SPATA31A1	1,16
ribonuclease H2, subunit C	RNASEH2C	1,16
forkhead box F1	FOXF1	1,16
nuclear receptor subfamily 1, group H, member 3	NR1H3	1,16
lysophospholipase-like 1	LYPLAL1	1,16
UDP-Gal:betaGlcNAc beta 1,4- galactosyltransferase, polypeptide 6	B4GALT6	1,16
galactosamine (N-acetyl)-6-sulfatase	GALNS	1,16
RasGEF domain family member 1C	RASGEF1C	1,16
small integral membrane protein 18	SMIM18	1,16
golgin A4	GOLGA4	1,16
mitogen-activated protein kinase 12	MAPK12	1,16
sorting nexin 24	SNX24	1,16
growth differentiation factor 10	GDF10	1,16
ribosomal RNA processing 15 homolog	RRP15	1,16
Jeck2013 ALT_ACCEPTOR, ALT_DONOR, coding, INTERNAL, intronic best transcript NM_000127; Transcript Identified by AceView	EXT1; spawla	1,16
Transcript Identified by AceView, Entrez Gene ID(s) 8470	SORBS2	1,16
leucine rich repeat and Ig domain containing 1	LINGO1	1,16
lactalbumin, alpha-	LALBA	1,16
long intergenic non-protein coding RNA 1140	LINC01140	1,16
glutathione S-transferase mu 5	GSTM5	1,16
zinc finger protein, FOG family member 1	ZFPM1	1,16
apolipoprotein L, 4	APOL4	1,16
hyaluronan and proteoglycan link protein 1	HAPLN1	1,16
endoglin	ENG	1,16
musashi RNA binding protein 2	MSI2	1,16
zinc finger protein 106	ZNF106	1,16
contactin 3 (plasmacytoma associated)	CNTN3	1,16

Rab interacting lysosomal protein-like 2	RILPL2	1,16
NLR family, pyrin domain containing 8	NLRP8	1,16
ubiquitin specific peptidase 32 pseudogene 2; family with sequence similarity 106, member A; coiled-coil domain containing 144B (pseudogene)	USP32P2; FAM106A; CCDC144B	1,16
cadherin-related family member 3	CDHR3	1,16
isocitrate dehydrogenase 1 (NADP+)	IDH1	1,16
DTW domain containing 2	DTWD2	1,16
ankyrin repeat and BTB (POZ) domain containing 1	ABTB1	1,16
zinc finger and BTB domain containing 32	ZBTB32	1,16
olfactory receptor, family 10, subfamily G, member 8	OR10G8	1,16
olfactory receptor, family 8, subfamily H, member 3	OR8H3	1,16
oxidative stress induced growth inhibitor 1	OSGIN1	1,16
TPTE and PTEN homologous inositol lipid phosphatase pseudogene; Transcript Identified by AceView, Entrez Gene ID(s) 1454; 400927	LOC400927; CSNK1E	1,16
junction mediating and regulatory protein, p53 cofactor	JMY	1,16
testis expressed 14	TEX14	1,16
TEA domain family member 3	TEAD3	1,16
branched chain amino-acid transaminase 2, mitochondrial	BCAT2	1,16
LRRN4 C-terminal like	LRRN4CL	1,16
potassium channel, two pore domain subfamily K, member 13	KCNK13	1,16
potassium channel subfamily M regulatory beta subunit 2	KCNMB2	1,16
negative elongation factor complex member A; microRNA 943	NELFA; MIR943	1,16
coiled-coil domain containing 121	CCDC121	1,16
HEAT repeat containing 5A	HEATR5A	1,16
histone cluster 2, H3d	HIST2H3D	1,16
tumor necrosis factor (ligand) superfamily, member 13b	TNFSF13B	1,16
OTU deubiquitinase 6A	OTUD6A	1,16
interleukin 36, beta	IL36B	1,16
zinc finger protein 625	ZNF625	1,16
ring finger protein 31	RNF31	1,16
RNA binding motif protein 27	RBM27	1,16
SWT1 RNA endoribonuclease homolog	SWT1	1,16
E74-like factor 1 (ets domain transcription factor)	ELF1	1,16
THAP domain containing 5	THAP5	1,16
microtubule associated serine/threonine kinase family member 4	MAST4	1,16
intraflagellar transport 46	IFT46	1,16
anthrax toxin receptor 1	ANTXR1	1,16
solute carrier family 22, member 20	SLC22A20	1,16
transformer 2 alpha homolog (Drosophila)	TRA2A	1,16
alkB homolog 8, tRNA methyltransferase	ALKBH8	1,16
RNA polymerase II associated protein 2	RPAP2	1,16

coiled-coil domain containing 115	CCDC115	1,16
Transcript Identified by AceView, Entrez Gene ID(s) 4643	MYO1E	1,16
Transcript Identified by AceView, Entrez Gene ID(s) 50515	CHST11	1,16
LY6/PLAUR domain containing 4	LYPD4	1,16
biorientation of chromosomes in cell division 1	BOD1	1,16
threonyl-tRNA synthetase	TARS	1,16
mitogen-activated protein kinase-activated protein kinase 3	MAPKAPK3	1,16
Transcript Identified by AceView, Entrez Gene ID(s) 84909; novel transcript	C9orf3; RP11-49O14.3	1,16
TNF receptor-associated factor 3 interacting protein 1	TRAF3IP1	1,16
arginine vasopressin	AVP	1,16
splicing factor 3b subunit 6	SF3B6	1,16
family with sequence similarity 222, member A	FAM222A	1,15
nucleoside-triphosphatase, cancer-related	NTPCR	1,15
taste receptor, type 2, member 13	TAS2R13	1,15
EF-hand calcium binding domain 6	EFCAB6	1,15
glucosidase, alpha; neutral C	GANC	1,15
gastrin-releasing peptide receptor	GRPR	1,15
zinc finger protein 436	ZNF436	1,15
GVQW motif containing 2	GVQW2	1,15
ADAM metallopeptidase domain 29	ADAM29	1,15
uridine-cytidine kinase 1	UCK1	1,15
KH domain containing 3-like, subcortical maternal complex member	KHDC3L	1,15
deoxyhypusine synthase	DHPS	1,15
potassium channel, calcium activated intermediate/small conductance subfamily N alpha, member 1	KCNN1	1,15
myosin, heavy chain 4, skeletal muscle	MYH4	1,15
Jeck2013 ALT_ACCEPTOR, ALT_DONOR, coding, INTERNAL, intronic best transcript NM_001037131; Transcript Identified by AceView	AGAP1; noygloy; torkeybo	1,15
neuroblastoma breakpoint family, member 11	NBPF11	1,15
zinc finger, AN1-type domain 4	ZFAND4	1,15
RNA binding motif protein 33	RBM33	1,15
integrin alpha FG-GAP repeat containing 1	ITFG1	1,15
olfactory receptor, family 6, subfamily Y, member 1	OR6Y1	1,15
calcium channel, voltage-dependent, beta 4 subunit	CACNB4	1,15
suppressor of cancer cell invasion; golgin A1	SCAI; GOLGA1	1,15
glycerophosphodiester phosphodiesterase domain containing 1	GDPD1	1,15
TMEM110-MUSTN1 readthrough; transmembrane protein 110	TMEM110-MUSTN1; TMEM110	1,15
FRY like transcription coactivator	FRYL	1,15
EP300 interacting inhibitor of differentiation 1	EID1	1,15

solute carrier family 5 (sodium/choline cotransporter), member 7	SLC5A7	1,15
integrin alpha 6	ITGA6	1,15
transmembrane protein 208	TMEM208	1,15
H1 histone family, member O, oocyte-specific	H1FOO	1,15
solute carrier family 8 (sodium/calcium exchanger), member 3	SLC8A3	1,15
proline-rich acidic protein 1	PRAP1	1,15
secretoglobin, family 1D, member 4	SCGB1D4	1,15
ribosomal protein S6 kinase, 90kDa, polypeptide 4	RPS6KA4	1,15
SCO-spondin	SSPO	1,15
growth factor, augmenter of liver regeneration	GFER	1,15
RNA binding motif protein 4	RBM4	1,15
anthrax toxin receptor 2	ANTXR2	1,15
signal-regulatory protein gamma	SIRPG	1,15
homeobox D8	HOXD8	1,15
lipase A, lysosomal acid, cholesterol esterase	LIPA	1,15
Jeck2013 ALT_ACCEPTOR, ALT_DONOR, coding, INTERNAL, intronic best transcript NM_005766	FARP1	1,15
RCD1 required for cell differentiation1 homolog (S. pombe)	RQCD1	1,15
tropomodulin 2 (neuronal)	TMOD2	1,15
Kruppel-like factor 1 (erythroid)	KLF1	1,15
defensin, beta 104A; defensin, beta 104B	DEFB104A; DEFB104B	1,15
PIN2/TERF1 interacting, telomerase inhibitor 1; microRNA 1322	PINX1; MIR1322	1,15
sialic acid binding Ig-like lectin 15	SIGLEC15	1,15
IBA57 homolog, iron-sulfur cluster assembly	IBA57	1,15
tigger transposable element derived 6	TIGD6	1,15
uncharacterized LOC100652929; novel transcript; uncharacterized LOC100652929 [Source:EntrezGene;Acc:100652929]	HP09025; CTD-2116F7.1	1,15
centrosomal protein 120kDa	CEP120	1,15
cytochrome c oxidase assembly factor 6	COA6	1,15
cystathionine-beta-synthase	CBS	1,15
KIAA0556	KIAA0556	1,15
hedgehog acyltransferase	HHAT	1,15
LSM2 homolog, U6 small nuclear RNA and mRNA degradation associated	LSM2	1,15
BCL6 corepressor-like 1	BCORL1	1,15
microtubule associated protein 1 light chain 3 gamma	MAP1LC3C	1,15
Memczak2013 ANTISENSE, INTERNAL, intronic, ncRNA best transcript NR_024568	FBXO31	1,15
tripartite motif containing 2	TRIM2	1,15
somatostatin receptor 2	SSTR2	1,15
spectrin repeat containing, nuclear envelope 2	SYNE2	1,15

spermatid perinuclear RNA binding protein; MIR600 host gene; microRNA 600	STRBP; MIR600HG; MIR600	1,15
solute carrier family 38, member 6	SLC38A6	1,15
keratin associated protein 26-1	KRTAP26-1	1,15
ADAM metallopeptidase with thrombospondin type 1 motif 3	ADAMTS3	1,15
zinc finger, DHHC-type containing 3	ZDHHC3	1,15
carboxypeptidase X (M14 family), member 2	CPXM2	1,15
dispatched homolog 2 (Drosophila)	DISP2	1,15
flavin adenine dinucleotide synthetase 1	FLAD1	1,15
SERTA domain containing 4	SERTAD4	1,15
calcium channel, voltage-dependent, L type, alpha 1S subunit	CACNA1S	1,15
olfactory receptor, family 5, subfamily K, member 3	OR5K3	1,15
KIAA0232	KIAA0232	1,15
family with sequence similarity 86, member B1	FAM86B1	1,15
Memczak2013 ALT_DONOR, coding, INTERNAL, intronic best transcript NM_001421	ELF4	1,15
TatD DNase domain containing 3	TATDN3	1,15
bicaudal D homolog 2 (Drosophila)	BICD2	1,15
alpha kinase 3	ALPK3	1,15
FSHD region gene 1	FRG1	1,15
leucine-rich repeats and WD repeat domain containing 1; microRNA 4467; microRNA 5090	LRWD1; MIR4467; MIR5090	1,15
DENN/MADD domain containing 1C	DENND1C	1,15
REC114 meiotic recombination protein	REC114	1,15
alpha thalassemia/mental retardation syndrome X-linked	ATRX	1,15
human immunodeficiency virus type I enhancer binding protein 3	HIVEP3	1,15
ATPase, H+ transporting, lysosomal 38kDa, V0 subunit d2	ATP6V0D2	1,15
WD repeat domain 38	WDR38	1,15
SUB1 homolog, transcriptional regulator	SUB1	1,15
SH3 and SYLF domain containing 1	SH3YL1	1,15
zinc finger protein 280C	ZNF280C	1,15
Fc receptor-like 6	FCRL6	1,15
G antigen 12C; G antigen 12E; G antigen 12H	GAGE12C; GAGE12E; GAGE12H	1,15
MANSC domain containing 1	MANSC1	1,15
Memczak2013 ANTISENSE, coding, INTERNAL, intronic best transcript NM_198390	CMIP	1,15
HECT, C2 and WW domain containing E3 ubiquitin protein ligase 1	HECW1	1,15
guanine nucleotide binding protein (G protein), alpha 14	GNA14	1,15
solute carrier family 35, member F2	SLC35F2	1,15
phosphoserine aminotransferase 1	PSAT1	1,15

Williams Beuren syndrome chromosome region 27	WBSCR27	1,15
interleukin 17 receptor C	IL17RC	1,15
CLK4-associating serine/arginine rich protein	CLASRP	1,15
transcription factor 23	TCF23	1,15
amyloid beta (A4) precursor protein-binding, family B, member 1 interacting protein	APBB1IP	1,15
nipsnap homolog 3A (C. elegans); nipsnap homolog 3B (C. elegans)	NIPSNAP3A; NIPSNAP3B	1,15
cadherin 20, type 2	CDH20	1,15
inositol polyphosphate-5-phosphatase D	INPP5D	1,15
A kinase (PRKA) anchor protein 9	AKAP9	1,15
GLIS family zinc finger 3	GLIS3	1,15
RAB, member of RAS oncogene family-like 2B	RABL2B	1,15
angel homolog 2 (Drosophila)	ANGEL2	1,15
succinate dehydrogenase complex assembly factor 1	SDHAF1	1,15
chromosome 1 open reading frame 131	C1orf131	1,15
cell division cycle 25B	CDC25B	1,15
PAF1 homolog, Paf1/RNA polymerase II complex component	PAF1	1,15
ELAV like RNA binding protein 1	ELAVL1	1,15
ST8 alpha-N-acetyl-neuraminide alpha-2,8-sialyltransferase 1	ST8SIA1	1,15
keratinocyte proline-rich protein	KPRP	1,15
zinc finger, DHHC-type containing 3	ZDHHC3	1,15
Transcript Identified by AceView, Entrez Gene ID(s) 26508	HEYL	1,15
vinculin	VCL	1,15
Transcript Identified by AceView, Entrez Gene ID(s) 149111; novel transcript	CNIH3; RP11-449J1.1	1,15
carbohydrate (N-acetylglucosamine 6-O) sulfotransferase 5	CHST5	1,15
POP1 homolog, ribonuclease P/MRP subunit	POP1	1,15
phospholipase A2, group IVC (cytosolic, calcium-independent)	PLA2G4C	1,15
ZFP2 zinc finger protein	ZFP2	1,15
chromosome 7 open reading frame 43; microRNA 4658	C7orf43; MIR4658	1,15
MAGE family member E2	MAGEE2	1,15
tubulin, gamma complex associated protein 3	TUBGCP3	1,15
arachidonate lipoxygenase 3	ALOXE3	1,15
pterin-4 alpha-carbinolamine dehydratase/dimerization cofactor of hepatocyte nuclear factor 1 alpha	PCBD1	1,15
homeobox B5	HOXB5	1,15
phospholipase A2, group IVD (cytosolic)	PLA2G4D	1,15
tyrosine kinase, non-receptor, 1	TNK1	1,15
NADH dehydrogenase (ubiquinone) Fe-S protein 3, 30kDa (NADH-coenzyme Q reductase)	NDUFS3	1,15
histone deacetylase 9	HDAC9	1,15
ATPase, H+ transporting, lysosomal 13kDa, V1 subunit G2	ATP6V1G2	1,15
TraB domain containing 2A	TRABD2A	1,15

beta-site APP-cleaving enzyme 1	BACE1	1,15
polymerase (RNA) III (DNA directed) polypeptide E (80kD)	POLR3E	1,15
zinc finger, DHHC-type containing 4	ZDHHC4	1,15
transcription factor EB	TFEB	1,15
olfactomedin 4	OLFM4	1,15
cysteine-serine-rich nuclear protein 2	CSRNP2	1,15
MAP7 domain containing 1	MAP7D1	1,15
olfactomedin like 1	OLFML1	1,15
metallothionein 3	MT3	1,15
fibronectin type III and SPRY domain containing 1	FSD1	1,15
G protein-coupled receptor 42 (gene/pseudogene)	GPR42	1,15
collagen, type X, alpha 1	COL10A1	1,15
zinc finger protein 541	ZNF541	1,15
kelch-like family member 21	KLHL21	1,15
betacellulin	BTC	1,15
WD repeat domain 73	WDR73	1,15
cytochrome P450, family 2, subfamily C, polypeptide 19	CYP2C19	1,15
solute carrier family 2 (facilitated glucose transporter), member 13	SLC2A13	1,15
tyrosine kinase with immunoglobulin-like and EGF-like domains 1	TIE1	1,15
inner mitochondrial membrane peptidase subunit 2	IMMP2L	1,15
secretogranin II	SCG2	1,15
MID1 interacting protein 1	MID1IP1	1,15
peripheral myelin protein 22	PMP22	1,15
SEC14-like lipid binding 1; small Cajal body-specific RNA 16; small nucleolar RNA host gene 20; microRNA 6516	SEC14L1; SCARNA16; SNHG20; MIR6516	1,15
serine/arginine repetitive matrix 2	SRRM2	1,15
zinc finger, matrin-type 5	ZMAT5	1,15
cell wall biogenesis 43 C-terminal homolog	CWH43	1,15
Zhang2013 ALT_ACCEPTOR, ALT_DONOR, coding, INTERNAL, intronic best transcript NM_004582	RABGGTB	1,15
general transcription factor IIH subunit 2	GTF2H2	1,15
host cell factor C1	HCFC1	1,15
Transcript Identified by AceView, Entrez Gene ID(s) 9759	HDAC4	1,15
translocase of inner mitochondrial membrane 17 homolog A (yeast)	TIMM17A	1,15
EGF-like, fibronectin type III and laminin G domains	EGFLAM	1,15
salvador family WW domain containing protein 1	SAV1	1,15
formin like 2	FMNL2	1,15
adaptor-related protein complex 3, beta 2 subunit	AP3B2	1,15
family with sequence similarity 217, member B	FAM217B	1,15

5-methyltetrahydrofolate-homocysteine methyltransferase reductase	MTRR	1,15
Eukaryotic translation initiation factor 3 subunit F [Source:UniProtKB/Swiss-Prot;Acc:O00303]	EIF3F	1,15
neudesin neurotrophic factor	NENF	1,15
zinc finger protein 836	ZNF836	1,15
solute carrier family 25, member 51	SLC25A51	1,15
sirtuin 3	SIRT3	1,15
acetyl-CoA carboxylase beta	ACACB	1,15
phosphodiesterase 3A, cGMP-inhibited	PDE3A	1,15
transmembrane protease, serine 13	TMPRSS13	1,15
PDZ and LIM domain 4	PDLIM4	1,15
interferon regulatory factor 7	IRF7	1,15
SAC3 domain containing 1	SAC3D1	1,15
family with sequence similarity 110, member B	FAM110B	1,15
armadillo repeat containing, X-linked 6	ARMCX6	1,15
tyrosine 3-monooxygenase/tryptophan 5-monooxygenase activation protein, eta	YWHAH	1,15
Memczak2013 ANTISENSE, coding, INTERNAL, intronic best transcript NM_001243234	TCF4	1,15
transmembrane and coiled-coil domain family 3; microRNA 7844	TMCC3; MIR7844	1,15
Transcript Identified by AceView, Entrez Gene ID(s) 5287	PIK3C2B	1,15
cytochrome P450, family 3, subfamily A, polypeptide 4	CYP3A4	1,15
SERTA domain containing 4	SERTAD4	1,15
killer cell lectin-like receptor subfamily D, member 1	KLRD1	1,15
polymerase (DNA directed), mu; microRNA 6838	POLM; MIR6838	1,15
serine/threonine kinase 36	STK36	1,15
thymocyte selection-associated high mobility group box	TOX	1,15
leukocyte specific transcript 1	LST1	1,15
NOC2-like nucleolar associated transcriptional repressor	NOC2L	1,15
abhydrolase domain containing 12B	ABHD12B	1,15
olfactory receptor, family 5, subfamily D, member 18	OR5D18	1,15
zinc finger and SCAN domain containing 22; microRNA 6806	ZSCAN22; MIR6806	1,15
CDC42 binding protein kinase beta (DMPK-like)	CDC42BPB	1,15
cytochrome P450, family 11, subfamily B, polypeptide 1	CYP11B1	1,15
late cornified envelope 1D	LCE1D	1,15
FXYD domain containing ion transport regulator 7	FXYD7	1,15
olfactory receptor, family 51, subfamily I, member 2	OR51I2	1,15
fibrous sheath CABYR binding protein	FSCB	1,15
amyloid beta (A4) precursor protein-binding, family B, member 3; microRNA 6831	APBB3; MIR6831	1,15
F-box and leucine-rich repeat protein 13	FBXL13	1,15

SV2 related protein	SVOP	1,15
protocadherin beta 8	PCDHB8	1,15
bestrophin 2	BEST2	1,15
platelet-derived growth factor beta polypeptide	PDGFB	1,15
protocadherin 7	PCDH7	1,15
solute carrier family 10 (sodium/bile acid cotransporter), member 1	SLC10A1	1,15
exportin 5	XPO5	1,15
chromosome 20 open reading frame 195	C20orf195	1,15
olfactory receptor, family 1, subfamily S, member 2	OR1S2	1,15
heat shock transcription factor 1	HSF1	1,15
hyperpolarization activated cyclic nucleotide gated potassium channel 1	HCN1	1,15
solute carrier family 35, member F4	SLC35F4	1,15
iroquois homeobox 6	IRX6	1,15
serpin peptidase inhibitor, clade B (ovalbumin), member 2	SERPINB2	1,15
transforming growth factor beta regulator 1	TBRG1	1,15
Memczak2013 ALT_DONOR, coding, INTERNAL, intronic best transcript NM_198268	HIPK1	1,15
glycerol-3-phosphate acyltransferase, mitochondrial	GPAM	1,15
heterogeneous nuclear ribonucleoprotein C-like 3 [Source:HGNC Symbol;Acc:HGNC:51235]	HNRNPCL3	1,15
transglutaminase 7	TGM7	1,15
syncollin	SYCN	1,15
sosondowah ankyrin repeat domain family member B	SOWAHB	1,15
kelch-like family member 17	KLHL17	1,15
POTE ankyrin domain family, member H	POTEH	1,15
TLC domain containing 2	TLCD2	1,15
complement component 1, q subcomponent, B chain	C1QB	1,15
leukocyte immunoglobulin-like receptor, subfamily B (with TM and ITIM domains), member 4	LILRB4	1,15
chromosome 12 open reading frame 60	C12orf60	1,15
tRNA isopentenyltransferase 1	TRIT1	1,15
REX1, RNA exonuclease 1 homolog-like 11, pseudogene	REXO1L11P	1,15
sterile alpha and TIR motif containing 1	SARM1	1,15
Transcript Identified by AceView, Entrez Gene ID(s) 23112	TNRC6B	1,15
major histocompatibility complex, class II, DR alpha	HLA-DRA	1,15
fructosamine 3 kinase	FN3K	1,15
Jun dimerization protein 2	JDP2	1,15
cyclin I family, member 2	CCNI2	1,15
translocase of inner mitochondrial membrane 50 homolog (S. cerevisiae)	TIMM50	1,15
Memczak2013 ALT_ACCEPTOR, ALT_DONOR, coding, INTERNAL, intronic best transcript NM_052847	GNG7	1,15
potassium channel, two pore domain subfamily K, member 6	KCNK6	1,15

zinc finger protein 35	ZNF35	1,15
mitochondrial ribosomal protein L20	MRPL20	1,15
Memczak2013 ALT_ACCEPTOR, ALT_DONOR, coding, INTERNAL, intronic best transcript NM_012479	YWHAG	1,15
serine/threonine kinase-like domain containing 1	STKLD1	1,15
HscB mitochondrial iron-sulfur cluster co-chaperone	HSCB	1,15
cholinergic receptor, nicotinic alpha 6	CHRNA6	1,15
RAB7B, member RAS oncogene family	RAB7B	1,15
SPARC like 1	SPARCL1	1,15
transcription elongation factor, mitochondrial	TEFM	1,15
long intergenic non-protein coding RNA 1219	LINC01219	1,15
SPEG complex locus	SPEG	1,15
kallikrein related peptidase 3	KLK3	1,15
POU class 3 homeobox 3	POU3F3	1,15
polyhomeotic homolog 3 (Drosophila)	PHC3	1,15
protein interacting with PRKCA 1	PICK1	1,15
delta-like 1 (Drosophila)	DLL1	1,15
MYC binding protein 2, E3 ubiquitin protein ligase	MYCBP2	1,15
snail family zinc finger 2	SNAI2	1,15
protein tyrosine phosphatase, non-receptor type 4 (megakaryocyte)	PTPN4	1,15
chromosome 10 open reading frame 71	C10orf71	1,15
potassium channel, voltage gated subfamily E regulatory beta subunit 5; acyl-CoA synthetase long-chain family member 4	KCNE5; ACSL4	1,15
solute carrier family 24 (sodium/potassium/calcium exchanger), member 3	SLC24A3	1,15
B double prime 1, subunit of RNA polymerase III transcription initiation factor IIIB	BDP1	1,15
autophagy related 3	ATG3	1,15
inositol hexakisphosphate kinase 3	IP6K3	1,15
interleukin 1 receptor accessory protein-like 2	IL1RAPL2	1,15
transcription factor 7 (T-cell specific, HMG-box)	TCF7	1,15
chromodomain helicase DNA binding protein 2; microRNA 3175	CHD2; MIR3175	1,15
U2AF homology motif (UHM) kinase 1	UHMK1	1,15
nuclear receptor subfamily 0, group B, member 1	NR0B1	1,15
complexin 2	CPLX2	1,15
family with sequence similarity 228, member B	FAM228B	1,15
APH1B gamma secretase subunit	APH1B	1,15
nudix hydrolase 22	NUDT22	1,15
fumarylacetoacetate hydrolase domain containing 2A	FAHD2A	1,15
WD repeat domain 91	WDR91	1,15
p53-induced death domain protein 1	PIDD1	1,15
euchromatic histone-lysine N-methyltransferase 1	EHMT1	1,15
fibulin 7	FBLN7	1,15

islet cell autoantigen 1 like	ICA1L	1,15
keratin 222, type II	KRT222	1,15
small integral membrane protein 23	SMIM23	1,15
RAB17, member RAS oncogene family	RAB17	1,15
SLIT-ROBO Rho GTPase activating protein 1	SRGAP1	1,15
nebulin	NEB	1,15
napsin A aspartic peptidase	NAPSA	1,15
cytohesin 2	CYTH2	1,15
WD repeat and FYVE domain containing 3	WDFY3	1,15
O-acyl-ADP-ribose deacylase 1	OARD1	1,15
taste receptor, type 2, member 8	TAS2R8	1,15
structural maintenance of chromosomes 6	SMC6	1,15
neuronal cell adhesion molecule	NRCAM	1,15
G protein-coupled receptor 22	GPR22	1,15
olfactory receptor, family 10, subfamily AG, member 1	OR10AG1	1,15
IMP (inosine 5-monophosphate) dehydrogenase 2	IMPDH2	1,15
superoxide dismutase 3, extracellular	SOD3	1,15
ankyrin repeat domain 33	ANKRD33	1,15
chromosome 10 open reading frame 90	C10orf90	1,15
taste receptor, type 2, member 5	TAS2R5	1,14
BR serine/threonine kinase 1	BRSK1	1,14
WD repeat domain 25	WDR25	1,14
Jeck2013 ANTISENSE, coding, INTERNAL, intronic best transcript NM_000088	COL1A1	1,14
HKR1, GLI-Kruppel zinc finger family member	HKR1	1,14
FAD-dependent oxidoreductase domain containing 1	FOXRED1	1,14
laminin, alpha 1	LAMA1	1,14
TIA1 cytotoxic granule-associated RNA binding protein	TIA1	1,14
N(alpha)-acetyltransferase 11, NatA catalytic subunit	NAA11	1,14
spindlin family, member 2B	SPIN2B	1,14
SEC14-like lipid binding 2	SEC14L2	1,14
layilin	LAYN	1,14
myosin, heavy chain 14, non-muscle	MYH14	1,14
leucine rich repeat containing 72	LRRC72	1,14
golgi transport 1A	GOLT1A	1,14
stomatin (EPB72)-like 3	STOML3	1,14
WAS protein family, member 3	WASF3	1,14
solute carrier family 16 (monocarboxylate transporter), member 1	SLC16A1	1,14
cyclin D binding myb-like transcription factor 1	DMTF1	1,14
rabphilin 3A	RPH3A	1,14
endonuclease, polyU-specific	ENDOU	1,14
Transcript Identified by AceView, Entrez Gene ID(s) 51056	LAP3	1,14
Zhang2013 ALT_ACCEPTOR, ALT_DONOR, coding, INTERNAL, intronic, OVERLAPTX best transcript NM_017550	MIER2	1,14

zinc finger protein 34	ZNF34	1,14
thioesterase superfamily member 4	THEM4	1,14
ribosomal protein S6 kinase, 90kDa, polypeptide 1	RPS6KA1	1,14
ankyrin and armadillo repeat containing	ANKAR	1,14
coiled-coil domain containing 57	CCDC57	1,14
spleen tyrosine kinase	SYK	1,14
trinucleotide repeat containing 18	TNRC18	1,14
zona pellucida glycoprotein 2 (sperm receptor)	ZP2	1,14
SPANX family, member C; SPANX family, member D	SPANXC; SPANXD	1,14
coiled-coil glutamate rich protein 1	CCER1	1,14
alpha tubulin acetyltransferase 1	ATAT1	1,14
dystonin	DST	1,14
laminin, alpha 4	LAMA4	1,14
lipid droplet associated hydrolase	LDAH	1,14
chromosome 16 open reading frame 74	C16orf74	1,14
signal transducer and activator of transcription 1	STAT1	1,14
family with sequence similarity 71, member E1	FAM71E1	1,14
glycine receptor alpha 1	GLRA1	1,14
TSEN54 tRNA splicing endonuclease subunit	TSEN54	1,14
cell death-inducing DFFA-like effector c	CIDEC	1,14
solute carrier family 4, sodium bicarbonate cotransporter, member 9	SLC4A9	1,14
myogenin (myogenic factor 4)	MYOG	1,14
HKR1, GLI-Kruppel zinc finger family member	HKR1	1,14
LIM domain binding 3	LDB3	1,14
synaptotagmin like 1	SYTL1	1,14
adenosine deaminase, RNA-specific, B1	ADARB1	1,14
translocator protein (18kDa)	TSPO	1,14
trio Rho guanine nucleotide exchange factor	TRIO	1,14
zinc finger protein 841	ZNF841	1,14
glutamate receptor, ionotropic, kainate 3	GRIK3	1,14
marginal zone B and B1 cell-specific protein	MZB1	1,14
chondroitin sulfate synthase 3	CHSY3	1,14
olfactory receptor, family 10, subfamily T, member 2	OR10T2	1,14
myotubularin related protein 4	MTMR4	1,14
sialic acid binding Ig-like lectin 9	SIGLEC9	1,14
AHA1, activator of heat shock 90kDa protein ATPase homolog 1 (yeast)	AHSA1	1,14
polyamine modulated factor 1 binding protein 1	PMFBP1	1,14
zinc finger protein 536	ZNF536	1,14
NADPH oxidase 1	NOX1	1,14
dual oxidase 1	DUOX1	1,14
jumonji, AT rich interactive domain 2	JARID2	1,14
coiled-coil domain containing 172	CCDC172	1,14

zinc finger, BED-type containing 2	ZBED2	1,14
chemokine (C-C motif) ligand 8	CCL8	1,14
toll-like receptor 8	TLR8	1,14
ArfGAP with coiled-coil, ankyrin repeat and PH domains 1	ACAP1	1,14
adrenomedullin 5 (putative); carnitine palmitoyltransferase 1C	ADM5; CPT1C	1,14
stabilin 1	STAB1	1,14
ankyrin repeat domain 30B-like	ANKRD30BL	1,14
chromosome 6 open reading frame 52	C6orf52	1,14
aquaporin 7	AQP7	1,14
golgin A6 family-like 10	GOLGA6L10	1,14
dehydrogenase E1 and transketolase domain containing 1	DHTKD1	1,14
ankyrin repeat and SOCS box containing 9	ASB9	1,14
copine IV	CPNE4	1,14
ankyrin repeat domain 31	ANKRD31	1,14
splicing factor 3a, subunit 1, 120kDa	SF3A1	1,14
coiled-coil domain containing 92	CCDC92	1,14
family with sequence similarity 159, member B	FAM159B	1,14
X-linked Kx blood group related 5	XKR5	1,14
serine carboxypeptidase 1	SCPEP1	1,14
wingless-type MMTV integration site family, member 1	WNT1	1,14
zinc finger protein 573	ZNF573	1,14
glycine-N-acyltransferase-like 3	GLYATL3	1,14
zinc finger protein 546	ZNF546	1,14
zinc finger protein 74	ZNF74	1,14
plakophilin 3	PKP3	1,14
chitinase 1 (chitotriosidase)	CHIT1	1,14
nuclear receptor interacting protein 1	NRIP1	1,14
adaptor-related protein complex 4, sigma 1 subunit	AP4S1	1,14
olfactory receptor, family 9, subfamily I, member 1	OR9I1	1,14
TMX2-CTNND1 readthrough (NMD candidate)	TMX2-CTNND1	1,14
CDC42 effector protein (Rho GTPase binding) 5	CDC42EP5	1,14
olfactory receptor, family 10, subfamily H, member 4	OR10H4	1,14
Memczak2013 ANTISENSE, coding, INTERNAL, UTR3 best transcript NM_004475	FLOT2	1,14
Memczak2013 ANTISENSE, coding, INTERNAL, intronic best transcript NM_205548	FAM151B	1,14
hemoglobin, theta 1	HBQ1	1,14
WD repeat domain 83 opposite strand	WDR83OS	1,14
ring finger protein 225	RNF225	1,14
clathrin, light chain A	CLTA	1,14
RNA binding motif protein 11	RBM11	1,14
RAP1A, member of RAS oncogene family	RAP1A	1,14
junction plakoglobin	JUP	1,14
chemokine (C-C motif) ligand 3	CCL3	1,14
diacylglycerol kinase, delta 130kDa	DGKD	1,14

family with sequence similarity 120A	FAM120A	1,14
WD repeat domain 5	WDR5	1,14
uncharacterized LOC388780; putative novel transcript	LOC388780; RP11-128M1.1	1,14
leucine rich colipase-like 1	LRCOL1	1,14
cystatin 8 (cystatin-related epididymal specific)	CST8	1,14
Transcript Identified by AceView, Entrez Gene ID(s) 816	CAMK2B	1,14
tripartite motif containing 44	TRIM44	1,14
chromosome 1 open reading frame 21	C1orf21	1,14
olfactory receptor, family 8, subfamily B, member 8	OR8B8	1,14
Zhang2013 ALT_ACCEPTOR, ALT_DONOR, coding, INTERNAL, intronic best transcript NM_058230	ZNF354B	1,14
fibronectin type III domain containing 3A	FNDC3A	1,14
nocturnin	NOCT	1,14
zinc finger protein 337	ZNF337	1,14
long intergenic non-protein coding RNA 525	LINC00525	1,14
nei-like DNA glycosylase 2	NEIL2	1,14
scavenger receptor cysteine rich family, 5 domains	SSC5D	1,14
G protein-coupled receptor 101	GPR101	1,14
Fraser extracellular matrix complex subunit 1	FRAS1	1,14
PYD (pyrin domain) containing 1	PYDC1	1,14
cysteine sulfinic acid decarboxylase	CSAD	1,14
cytochrome P450, family 3, subfamily A, polypeptide 43	CYP3A43	1,14
inhibitor of kappa light polypeptide gene enhancer in B-cells, kinase beta	IKBKB	1,14
olfactory receptor, family 2, subfamily W, member 1	OR2W1	1,14
spermatogenic leucine zipper 1	SPZ1	1,14
Transcript Identified by AceView, Entrez Gene ID(s) 83699	SH3BGRL2	1,14
centrosomal protein 68kDa	CEP68	1,14
catsper channel auxiliary subunit beta	CATSPERB	1,14
guanine nucleotide binding protein (G protein), alpha transducing activity polypeptide 2	GNAT2	1,14
ceramide synthase 4	CERS4	1,14
megakaryoblastic leukemia (translocation) 1	MKL1	1,14
nuclear pore complex interacting protein family, member A1	NPIPA1	1,14
placenta specific 4	PLAC4	1,14
high mobility group nucleosomal binding domain 2	HMGN2	1,14
acrosin binding protein	ACRBP	1,14
lactate dehydrogenase A-like 6A	LDHAL6A	1,14
pleckstrin homology domain containing, family H (with MyTH4 domain) member 1	PLEKHH1	1,14
bone morphogenetic protein 8b	BMP8B	1,14
ring finger protein 2	RNF2	1,14
transmembrane protein 60	TMEM60	1,14
phosphofurin acidic cluster sorting protein 2	PACS2	1,14

human immunodeficiency virus type I enhancer binding protein 1	HIVEP1	1,14
PDZ and LIM domain 7 (enigma)	PDLIM7	1,14
lysine (K)-specific methyltransferase 2B	KMT2B	1,14
transmembrane protein 234	TMEM234	1,14
family with sequence similarity 72, member B	FAM72B	1,14
zinc finger CCCH-type containing 12B	ZC3H12B	1,14
wingless-type MMTV integration site family, member 9B	WNT9B	1,14
Berardinelli-Seip congenital lipodystrophy 2 (seipin)	BSCL2	1,14
UDP-Gal:betaGlcNAc beta 1,3-galactosyltransferase 4	B3GALT4	1,14
PHD finger protein 24	PHF24	1,14
lysine (K)-specific demethylase 6A	KDM6A	1,14
family with sequence similarity 104, member B	FAM104B	1,14
testin LIM domain protein	TES	1,14
proteasome activator subunit 2; microRNA 7703	PSME2; MIR7703	1,14
solute carrier family 5 (sodium/glucose cotransporter), member 1	SLC5A1	1,14
B-cell CLL/lymphoma 9	BCL9	1,14
family with sequence similarity 180, member A	FAM180A	1,14
chromosome 14 open reading frame 142	C14orf142	1,14
MLX interacting protein	MLXIP	1,14
acyl-CoA binding domain containing 4	ACBD4	1,14
phosphatidylinositol-3,4,5-trisphosphate-dependent Rac exchange factor 2	PREX2	1,14
defensin, beta 108B	DEFB108B	1,14
N-acetyltransferase 8B (GCN5-related, putative, gene/pseudogene)	NAT8B	1,14
radial spoke head 14 homolog (Chlamydomonas)	RSPH14	1,14
G-patch domain and ankyrin repeats 1	GPANK1	1,14
protein tyrosine phosphatase, receptor type, R	PTPRR	1,14
adenosine deaminase, tRNA-specific 2	ADAT2	1,14
activin A receptor type IB	ACVR1B	1,14
testis-specific serine kinase 4	TSSK4	1,14
transmembrane protein 262	TMEM262	1,14
diacylglycerol O-acyltransferase 1; microRNA 6848	DGAT1; MIR6848	1,14
chromosome 9 open reading frame 43	C9orf43	1,14
caspase 4	CASP4	1,14
ankyrin repeat and SOCS box containing 16	ASB16	1,14
ATPase, H+ transporting, lysosomal V0 subunit a4	ATP6V0A4	1,14
IWS1 homolog (S. cerevisiae)	IWS1	1,14
3-hydroxyisobutyryl-CoA hydrolase	HIBCH	1,14
CDC42 small effector 2	CDC42SE2	1,14
PC-esterase domain containing 1B	PCED1B	1,14

PR domain containing 6	PRDM6	1,14
latent transforming growth factor beta binding protein 3	LTBP3	1,14
clathrin, heavy chain (Hc)	CLTC	1,14
teratocarcinoma-derived growth factor 1	TDGF1	1,14
acyl-CoA synthetase medium-chain family member 3	ACSM3	1,14
zinc finger, CCHC domain containing 8	ZCCHC8	1,14
lysine (K)-specific demethylase 2A	KDM2A	1,14
C-type lectin domain family 18, member C	CLEC18C	1,14
TGFB-induced factor homeobox 2-like, X-linked	TGIF2LX	1,14
mediator complex subunit 13-like	MED13L	1,14
SH2 domain containing 4A	SH2D4A	1,14
fibronectin type III domain containing 7	FNDC7	1,14
transcobalamin I (vitamin B12 binding protein, R binder family)	TCN1	1,14
zinc finger protein 765	ZNF765	1,14
POM121 and ZP3 fusion	POMZP3	1,14
N-acyl phosphatidylethanolamine phospholipase D	NAPEPLD	1,14
MLX interacting protein-like	MLXIPL	1,14
transcription factor Dp-2 (E2F dimerization partner 2)	TFDP2	1,14
olfactory receptor, family 13, subfamily F, member 1	OR13F1	1,14
Tax1 (human T-cell leukemia virus type I) binding protein 3	TAX1BP3	1,14
stromal antigen 3-like 4 (pseudogene)	STAG3L4	1,14
Jeck2013 ANTISENSE, CDS, coding, INTERNAL, intronic, OVCODE, OVEXON best transcript NM_017491	WDR1	1,14
CD1b molecule	CD1B	1,14
zinc finger protein 610	ZNF610	1,14
polymerase (DNA directed) iota	POLI	1,14
epsin 3	EPN3	1,14
mitogen-activated protein kinase 11	MAPK11	1,14
protein phosphatase 1, regulatory subunit 21	PPP1R21	1,14
nuclear receptor subfamily 2, group E, member 1	NR2E1	1,14
integrin beta 6	ITGB6	1,14
ATP/GTP binding protein 1	AGTPBP1	1,14
interferon (alpha, beta and omega) receptor 2	IFNAR2	1,14
prefoldin subunit 4	PFDN4	1,14
olfactory receptor, family 10, subfamily G, member 7	OR10G7	1,14
Homo sapiens REX1, RNA exonuclease 1 homolog-like 2, pseudogene (REXO1L2P), non-coding RNA.; Synthetic construct Homo sapiens clone IMAGE:100068172, MGC:195789 REX1, RNA exonuclease 1 homolog (S. cerevisiae)-like 1 (REXO1L1) mRNA, encodes complete protein.; Transcript Identified by AceView, RefSeq ID(s) NR_003594	REXO1L2P; REXO1L1; blarbeyby	1,14
deiodinase, iodothyronine, type II	DIO2	1,14
diacylglycerol O-acyltransferase 2	DGAT2	1,14
poly(ADP-ribose) polymerase family member 15	PARP15	1,14
MET proto-oncogene, receptor tyrosine kinase	MET	1,14

DEAD (Asp-Glu-Ala-Asp) box polypeptide 58	DDX58	1,14
hyaluronan and proteoglycan link protein 4	HAPLN4	1,14
chromosome 3 open reading frame 52	C3orf52	1,14
paired-like homeobox 2b	PHOX2B	1,14
regenerating islet-derived 1 alpha	REG1A	1,14
hematological and neurological expressed 1-like	HN1L	1,14
Transcript Identified by AceView, Entrez Gene ID(s) 4642; novel transcript, sense intronic MYO1D	RP11-466A19.5; MYO1D	1,14
5-hydroxytryptamine (serotonin) receptor 7, adenylate cyclase-coupled	HTR7	1,14
leucine rich repeat neuronal 3	LRRN3	1,14
unconventional SNARE in the ER 1 homolog (S. cerevisiae)	USE1	1,14
regulator of calcineurin 1	RCAN1	1,14
Zhang2013 ALT_DONOR, coding, INTERNAL, intronic best transcript NM_001006	RPS3A	1,14
MORN repeat containing 1	MORN1	1,14
thymopoietin	TMPO	1,14
coiled-coil and C2 domain containing 2A	CC2D2A	1,14
chromosome 12 open reading frame 29	C12orf29	1,14
tubby bipartite transcription factor	TUB	1,14
long intergenic non-protein coding RNA 885	LINC00885	1,14
tumor necrosis factor receptor superfamily, member 1A	TNFRSF1A	1,14
glutamate receptor, ionotropic, kainate 4	GRIK4	1,14
RAS p21 protein activator (GTPase activating protein) 1	RASA1	1,14
JMJD1C antisense RNA 1	JMJD1C-AS1	1,14
chromosome 6 open reading frame 141	C6orf141	1,14
retinoschisin 1	RS1	1,14
long intergenic non-protein coding RNA 1549	LINC01549	1,14
junctophilin 4	JPH4	1,14
transmembrane protein 80	TMEM80	1,14
protein kinase N2	PKN2	1,14
myelodysplastic syndrome 2 translocation associated	MDS2	1,14
Memczak2013 ALT_ACCEPTOR, ALT_DONOR, coding, INTERNAL, intronic best transcript NM_001003800	BICD2	1,14
C2 calcium-dependent domain containing 4C	C2CD4C	1,14
carbohydrate (N-acetylgalactosamine 4-0) sulfotransferase 9	CHST9	1,14
zinc finger protein 239	ZNF239	1,14
MAP6 domain containing 1	MAP6D1	1,14
forkhead box E1	FOXE1	1,14
ATPase, class II, type 9B	ATP9B	1,14
WAP four-disulfide core domain 6	WFDC6	1,14
HNF1 homeobox A	HNF1A	1,14
gasdermin D	GSDMD	1,14
prolyl endopeptidase-like	PREPL	1,14

E1A binding protein p400; small nucleolar RNA, H/ACA box 49	EP400; SNORA49	1,14
homer scaffolding protein 2	HOMER2	1,14
TBC1 domain family, member 26	TBC1D26	1,14
family with sequence similarity 57, member B	FAM57B	1,14
IZUMO1 receptor, JUNO	IZUMO1R	1,14
proteasome 26S subunit, ATPase 4 [Source:HGNC Symbol;Acc:HGNC:9551]	PSMC4	1,14
regulatory factor X-associated protein	RFXAP	1,14
transmembrane protein 225	TMEM225	1,14
matrilin 4	MATN4	1,14
epilepsy, progressive myoclonus type 2A, Lafora disease (laforin)	EPM2A	1,14
GRINL1A complex locus 1; myocardial zonula adherens protein; polymerase (RNA) II (DNA directed) polypeptide M	GCOM1; MYZAP; POLR2M	1,14
cadherin-related family member 5	CDHR5	1,14
chemokine (C-X-C motif) ligand 1 (melanoma growth stimulating activity, alpha)	CXCL1	1,14
FIG4 phosphoinositide 5-phosphatase	FIG4	1,14
olfactory receptor, family 2, subfamily A, member 12	OR2A12	1,14
Ras protein-specific guanine nucleotide-releasing factor 2	RASGRF2	1,14
pancreatic lipase-related protein 2	PNLIPRP2	1,14
SHANK2 antisense RNA 3	SHANK2-AS3	1,14
serine/threonine kinase 38 like	STK38L	1,14
apoptosis-inducing factor, mitochondrion-associated, 2	AIFM2	1,14
tRNA methyltransferase 6	TRMT6	1,14
zinc and ring finger 4	ZNRF4	1,14
antioxidant 1 copper chaperone	ATOX1	1,14
SH3 and cysteine rich domain 2	STAC2	1,14
keratin associated protein 19-1	KRTAP19-1	1,14
zinc finger and BTB domain containing 1	ZBTB1	1,14
tetratricopeptide repeat domain 21B	TTC21B	1,14
proline rich 16	PRR16	1,14
olfactory receptor, family 4, subfamily C, member 46	OR4C46	1,14
cytochrome c oxidase assembly factor 3	COA3	1,14
transcription factor Dp-1	TFDP1	1,14
ring finger protein 182	RNF182	1,14
chimerin 2	CHN2	1,14
sine oculis binding protein homolog	SOBP	1,14
phosphorylase, glycogen; brain	PYGB	1,14
PRAME family member 4	PRAMEF4	1,14
G protein-coupled receptor, class C, group 5, member A; microRNA 614	GPRC5A; MIR614	1,14
COMM domain containing 8	COMMD8	1,14

complement factor B	CFB	1,14
TAF5-like RNA polymerase II, p300/CBP-associated factor (PCAF)-associated factor, 65kDa	TAF5L	1,14
pterin-4 alpha-carbinolamine dehydratase/dimerization cofactor of hepatocyte nuclear factor 1 alpha (TCF1) 2	PCBD2	1,14
numb homolog (Drosophila)-like	NUMBL	1,14
ankyrin repeat and sterile alpha motif domain containing 1A	ANKS1A	1,14
calsyntenin 2	CLSTN2	1,14
neuropilin (NRP) and tolloid (TLL)-like 2	NETO2	1,14
transporter 2, ATP-binding cassette, sub-family B (MDR/TAP)	TAP2	1,14
solute carrier family 8 (sodium/calcium exchanger), member 2	SLC8A2	1,14
component of oligomeric golgi complex 5	COG5	1,14
ribonuclease P/MRP 25kDa subunit	RPP25	1,14
ankyrin repeat domain 44	ANKRD44	1,13
SUMO/sentrin peptidase family member, NEDD8 specific	SENP8	1,13
L1 cell adhesion molecule	L1CAM	1,13
leucine carboxyl methyltransferase 2	LCMT2	1,13
transcription elongation regulator 1	TCERG1	1,13
vacuolar protein sorting 13 homolog B (yeast)	VPS13B	1,13
keratin 81, type II	KRT81	1,13
LDL receptor related protein 8	LRP8	1,13
zinc finger protein 648	ZNF648	1,13
lon peptidase 1, mitochondrial	LONP1	1,13
oxysterol binding protein	OSBP	1,13
transducin (beta)-like 3	TBL3	1,13
Transcript Identified by AceView, Entrez Gene ID(s) 84256	FLYWCH1	1,13
TGF-beta activated kinase 1/MAP3K7 binding protein 1	TAB1	1,13
SPATA31 subfamily D, member 1	SPATA31D1	1,13
Transcript Identified by AceView, Entrez Gene ID(s) 5552	SRGN	1,13
metallo-beta-lactamase domain containing 1	MBLAC1	1,13
programmed cell death 1 ligand 2	PDCD1LG2	1,13
SEC11 homolog C, signal peptidase complex subunit	SEC11C	1,13
regulatory subunit of type II PKA R-subunit (RIIa) domain containing 1	RIIAD1	1,13
zinc finger protein 688	ZNF688	1,13
cyclin O	CCNO	1,13
interferon, gamma-inducible protein 16	IFI16	1,13
translocase of inner mitochondrial membrane 50 homolog (S. cerevisiae)	TIMM50	1,13
FUN14 domain containing 1	FUNDC1	1,13
fumarylacetoacetate hydrolase (fumarylacetoacetase)	FAH	1,13
zinc finger protein 729	ZNF729	1,13
poly(rC) binding protein 4	PCBP4	1,13
flightless I actin binding protein	FLII	1,13
Transcript Identified by AceView, Entrez Gene ID(s) 215	ABCD1	1,13

RAB22A, member RAS oncogene family	RAB22A	1,13
zinc finger protein 713	ZNF713	1,13
olfactory receptor, family 5, subfamily AC, member 2	OR5AC2	1,13
Rap guanine nucleotide exchange factor 3	RAPGEF3	1,13
PRAME family member 27	PRAMEF27	1,13
Transcript Identified by AceView, Entrez Gene ID(s) 5885	RAD21	1,13
defensin, beta 107B; defensin, beta 107A	DEFB107B; DEFB107A	1,13
melatonin receptor 1A	MTNR1A	1,13
synovial sarcoma, X breakpoint 3	SSX3	1,13
THAP domain containing 11	THAP11	1,13
transmembrane protein 179	TMEM179	1,13
Rho GTPase activating protein 22	ARHGAP22	1,13
tet methylcytosine dioxygenase 2	TET2	1,13
sperm acrosome associated 3	SPACA3	1,13
synaptojanin 2	SYNJ2	1,13
leucine rich repeat containing 37A	LRRC37A	1,13
solute carrier family 23 (ascorbic acid transporter), member 1	SLC23A1	1,13
spermatogenesis associated 9	SPATA9	1,13
carboxypeptidase A6	CPA6	1,13
TATA box binding protein associated factor 8	TAF8	1,13
D-tyrosyl-tRNA deacylase 1	DTD1	1,13
chemokine (C motif) ligand 2	XCL2	1,13
Homo sapiens golgin A6 family-like 4 (GOLGA6L4), mRNA.; golgin A6 family-like 4; golgin A6 family-like 4 [Source:HGNC Symbol;Acc:HGNC:27256]	GOLGA6L4	1,13
F-box and leucine-rich repeat protein 14	FBXL14	1,13
chromosome 5 open reading frame 52	C5orf52	1,13
chromosome 9 open reading frame 163	C9orf163	1,13
caudal type homeobox 2	CDX2	1,13
TSR3, 20S rRNA accumulation, homolog (S. cerevisiae)	TSR3	1,13
coiled-coil-helix-coiled-coil-helix domain containing 7	CHCHD7	1,13
anosmin 1	ANOS1	1,13
metastasis associated 1 family member 2	MTA2	1,13
glutamyl-tRNA(Gln) amidotransferase, subunit B	GATB	1,13
cholinergic receptor, nicotinic alpha 3	CHRNA3	1,13
ferritin, heavy polypeptide-like 17	FTHL17	1,13
trafficking protein particle complex 4	TRAPPC4	1,13
actin related protein 2/3 complex subunit 1A	ARPC1A	1,13
Jeck2013 ANTISENSE, coding, INTERNAL, intronic best transcript NM_020973	GBA3	1,13
casein kinase 2, alpha prime polypeptide	CSNK2A2	1,13
phosphomannomutase 2	PMM2	1,13
interleukin 31	IL31	1,13

mutS homolog 5; MSH5-SAPCD1 readthrough (NMD candidate); suppressor APC domain containing 1	MSH5; MSH5-SAPCD1; SAPCD1	1,13
olfactory receptor, family 1, subfamily L, member 8	OR1L8	1,13
ADAM metallopeptidase with thrombospondin type 1 motif 4	ADAMTS4	1,13
bassoon presynaptic cytomatrix protein	BSN	1,13
chromosome 1 open reading frame 35	C1orf35	1,13
LUC7-like	LUC7L	1,13
ubiquitin-conjugating enzyme E2, J2	UBE2J2	1,13
Homo sapiens interleukin 3 receptor, alpha (low affinity) (IL3RA), transcript variant 2, mRNA.; Homo sapiens interleukin 3 receptor, alpha (low affinity) (IL3RA), transcript variant 1, mRNA.; interleukin 3 receptor, alpha (low affinity) [Source:HGNC Symbol;Acc:HGNC:6012]; Homo sapiens interleukin 3 receptor, alpha (low affinity), mRNA (cDNA clone MGC:34174 IMAGE:5167281), complete cds.; Salzman2013 ANNOTATED, CDS, coding, INTERNAL, OVCODE, OVERLAPTX, OVEXON best transcript NM_002183	IL3RA	1,13
retinol binding protein 3, interstitial	RBP3	1,13
killer cell lectin-like receptor subfamily G, member 1	KLRG1	1,13
late cornified envelope 3C	LCE3C	1,13
hydroxysteroid (17-beta) dehydrogenase 6	HSD17B6	1,13
PRAME family member 7	PRAMEF7	1,13
phospholipase A2, group IVF	PLA2G4F	1,13
myosin light chain 7	MYL7	1,13
ILK associated serine/threonine phosphatase	ILKAP	1,13
S-antigen; retina and pineal gland (arrestin)	SAG	1,13
senataxin	SETX	1,13
ATPase, aminophospholipid transporter, class I, type 8B, member 1	ATP8B1	1,13
ADAM metallopeptidase with thrombospondin type 1 motif 13	ADAMTS13	1,13
ankyrin repeat and FYVE domain containing 1	ANKFY1	1,13
olfactory receptor, family 6, subfamily K, member 2	OR6K2	1,13
polycystic kidney and hepatic disease 1 (autosomal recessive)-like 1	PKHD1L1	1,13
lipocalin 10	LCN10	1,13
testis expressed 264	TEX264	1,13
tripartite motif containing 10	TRIM10	1,13
olfactory receptor, family 7, subfamily E, member 24	OR7E24	1,13
Enah/Vasp-like	EVL	1,13
collagen, type II, alpha 1	COL2A1	1,13
GLI pathogenesis-related 1 like 1	GLIPR1L1	1,13
chromosome 1 open reading frame 123	C1orf123	1,13
leiomodin 3 (fetal)	LMOD3	1,13

SWI/SNF related, matrix associated, actin dependent regulator of chromatin, subfamily d, member 3	SMARCD3	1,13
tripartite motif containing 72, E3 ubiquitin protein ligase	TRIM72	1,13
netrin 4	NTN4	1,13
ring finger protein 130	RNF130	1,13
tubulin tyrosine ligase-like family member 3	TTLL3	1,13
gem nuclear organelle associated protein 6	GEMIN6	1,13
stimulator of chondrogenesis 1	SCRG1	1,13
PRELI domain containing 3B; ATP synthase, H+ transporting, mitochondrial F1 complex, epsilon subunit	PRELID3B; ATP5E	1,13
hydroxy-delta-5-steroid dehydrogenase, 3 beta- and steroid delta-isomerase 7	HSD3B7	1,13
DnaJ (Hsp40) homolog, subfamily B, member 3	DNAJB3	1,13
exonuclease NEF-sp; Putative RNA exonuclease NEF-sp [Source:UniProtKB/Swiss-Prot;Acc:Q96IC2]; Salzman2013 ANNOTATED, CDS, coding, OVCODE, OVERLAPTX, OVEXON, UTR3, UTR5 best transcript NM_001199053; Salzman2013 ANNOTATED, CDS, coding, INTERNAL, OVCODE, OVERLAPTX, OVEXON best transcript NM_001199053; Salzman2013 ANNOTATED, CDS, coding, OVCODE, OVERLAPTX, OVEXON, UTR3 best transcript NM_001199053; Transcript Identified by AceView, Entrez Gene ID(s) 81691; U4atac minor spliceosomal RNA [Source:RFAM;Acc:RF00618]	LOC81691; AC004381.6; U4atac	1,13
matrix metallopeptidase 20	MMP20	1,13
aspartate dehydrogenase domain containing	ASPDH	1,13
zinc finger protein 367	ZNF367	1,13
taste receptor, type 1, member 2	TAS1R2	1,13
2-5-oligoadenylate synthetase 2	OAS2	1,13
ADP-ribosylation factor like GTPase 5C	ARL5C	1,13
Memczak2013 ALT_ACCEPTOR, ALT_DONOR, coding, INTERNAL, intronic best transcript NM_001193571; Transcript Identified by AceView	CSRP1; markobu	1,13
signaling lymphocytic activation molecule family member 1	SLAMF1	1,13
natriuretic peptide receptor 2	NPR2	1,13
protein tyrosine kinase 7 (inactive)	PTK7	1,13
LIM homeobox 8	LHX8	1,13
PQ loop repeat containing 3	PQLC3	1,13
HAUS augmin like complex subunit 6	HAUS6	1,13
GLI family zinc finger 4	GLI4	1,13
thioredoxin 2	TXN2	1,13
ankyrin repeat domain 65	ANKRD65	1,13
SIX homeobox 2	SIX2	1,13
zinc finger, DHHC-type containing 13	ZDHHC13	1,13
protein tyrosine phosphatase, receptor type, f polypeptide (PTPRF), interacting protein (liprin), alpha 3	PPFIA3	1,13

elastase, neutrophil expressed	ELANE	1,13
general transcription factor IIIA	GTF3A	1,13
zinc finger protein 19	ZNF19	1,13
SLX1 homolog A, structure-specific endonuclease subunit; SLX1 homolog B, structure-specific endonuclease subunit; SLX1A-SULT1A3 readthrough (NMD candidate); SLX1B-SULT1A4 readthrough (NMD candidate); sulfotransferase family 1A member 3	SLX1A; SLX1B; SLX1A-SULT1A3; SLX1B-SULT1A4; SULT1A3	1,13
exosome component 4; microRNA 6847	EXOSC4; MIR6847	1,13
synaptotagmin I	SYT1	1,13
RAP1, GTP-GDP dissociation stimulator 1	RAP1GDS1	1,13
Transcript Identified by AceView, Entrez Gene ID(s) 55315	SLC29A3	1,13
splicing factor 3b subunit 2	SF3B2	1,13
Tu translation elongation factor, mitochondrial; microRNA 4721	TUFM; MIR4721	1,13
ankyrin repeat and ubiquitin domain containing 1	ANKUB1	1,13
pleckstrin homology domain containing, family G (with RhoGef domain) member 4	PLEKHG4	1,13
ybeY metallopeptidase (putative)	YBEY	1,13
bora, aurora kinase A activator	BORA	1,13
chromosome 9 open reading frame 78	C9orf78	1,13
centromere protein U	CENPU	1,13
NADH dehydrogenase (ubiquinone) 1, alpha/beta subcomplex, 1, 8kDa	NDUFAB1	1,13
immunoglobulin lambda-like polypeptide 5; microRNA 5571; immunoglobulin lambda constant 1 (Mcg marker); immunoglobulin lambda constant 2 (Kern-Oz- marker); immunoglobulin lambda variable 2-5 (pseudogene); immunoglobulin lambda variable 3-1; immunoglobulin lambda joining 1	IGLL5; MIR5571; IGLC1; IGLC2; IGLV2-5; IGLV3-1; IGLJ1	1,13
phosphatidylinositol 4-kinase type 2 beta	PI4K2B	1,13
serpin peptidase inhibitor, clade B (ovalbumin), member 12	SERPINB12	1,13
mannosyl (beta-1,4-)-glycoprotein beta-1,4-N-acetylglucosaminyltransferase	MGAT3	1,13
Transcript Identified by AceView, Entrez Gene ID(s) 149297; novel transcript	RP11-9L18.3; FAM78B	1,13
proteasome 26S subunit, non-ATPase 13	PSMD13	1,13
clusterin associated protein 1	CLUAP1	1,13
E74-like factor 2 (ets domain transcription factor)	ELF2	1,13
SR-related CTD-associated factor 1	SCAF1	1,13
coiled-coil domain containing 63	CCDC63	1,13
zinc finger protein 597	ZNF597	1,13
NLR family, pyrin domain containing 7	NLRP7	1,13

calcium channel, voltage-dependent, gamma subunit 5	CACNG5	1,13
keratin associated protein 10-1	KRTAP10-1	1,13
cryptochrome circadian clock 1	CRY1	1,13
von Willebrand factor A domain containing 3B	VWA3B	1,13
tetratricopeptide repeat domain 21A; microRNA 6822	TTC21A; MIR6822	1,13
PDZ domain containing 8	PDZD8	1,13
BicC family RNA binding protein 1	BICC1	1,13
aldehyde dehydrogenase 8 family, member A1	ALDH8A1	1,13
proline dehydrogenase (oxidase) 1	PRODH	1,13
SWI/SNF related, matrix associated, actin dependent regulator of chromatin, subfamily a, member 5	SMARCA5	1,13
C-x(9)-C motif containing 2	CMC2	1,13
dpy-19-like 3 (C. elegans)	DPY19L3	1,13
polysaccharide biosynthesis domain containing 1	PBDC1	1,13
chromosome 4 open reading frame 19	C4orf19	1,13
transmembrane protein 104	TMEM104	1,13
POM121 transmembrane nucleoporin-like 2	POM121L2	1,13
THUMP domain containing 3	THUMPD3	1,13
latent transforming growth factor beta binding protein 1	LTBP1	1,13
solute carrier family 30, member 10	SLC30A10	1,13
G protein-coupled receptor, class C, group 5, member B	GPRC5B	1,13
transcription factor binding to IGHM enhancer 3	TFE3	1,13
peptidase inhibitor 15	PI15	1,13
tripartite motif containing 7	TRIM7	1,13
mutS homolog 4	MSH4	1,13
receptor-like tyrosine kinase	RYK	1,13
phosphatidylserine synthase 1	PTDSS1	1,13
golgi reassembly stacking protein 1	GORASP1	1,13
kallikrein related peptidase 4	KLK4	1,13
myosin IXA	MYO9A	1,13
zinc finger, BED-type containing 1	ZBED1	1,13
acetylcholinesterase (Yt blood group)	ACHE	1,13
pleckstrin homology domain containing, family B (evectins) member 1	PLEKHB1	1,13
sodium channel, voltage gated, type II alpha subunit	SCN2A	1,13
phosphoprotein enriched in astrocytes 15	PEA15	1,13
nucleoporin 85kDa	NUP85	1,13
RNA binding motif protein 43	RBM43	1,13
FERM and PDZ domain containing 4	FRMPD4	1,13
nuclear RNA export factor 3	NXF3	1,13
chromosome 1 open reading frame 74	C1orf74	1,13
dopey family member 1	DOPEY1	1,13
neuregulin 2	NRG2	1,13
solute carrier family 27 (fatty acid transporter), member 2	SLC27A2	1,13

zinc finger, BED-type containing 8	ZBED8	1,13
BCS1 homolog, ubiquinol-cytochrome c reductase complex chaperone	BCS1L	1,13
adenosine deaminase, RNA-specific, B2 (inactive)	ADARB2	1,13
ribonuclease, RNase K; chromosome 17 open reading frame 49; RNASEK-C17orf49 readthrough	RNASEK; C17orf49; RNASEK-C17orf49	1,13
Memczak2013 ANTISENSE, CDS, coding, INTERNAL best transcript NM_001109	ADAM8	1,13
DDB1 and CUL4 associated factor 17	DCAF17	1,13
Memczak2013 ANTISENSE, CDS, coding, INTERNAL best transcript NM_002957	RXRA	1,13
adrenoceptor alpha 1B	ADRA1B	1,13
centrosomal protein 85kDa	CEP85	1,13
deiodinase, iodothyronine, type III	DIO3	1,13
Memczak2013 ANTISENSE, CDS, coding, INTERNAL best transcript NM_020979	SH2B2	1,13
nucleic acid binding protein 1	NABP1	1,13
Jeck2013 ALT_ACCEPTOR, ALT_DONOR, coding, INTERNAL, intronic best transcript NM_032531	KIRREL3	1,13
chromosome 10 open reading frame 99	C10orf99	1,13
paired-like homeodomain 3	PITX3	1,13
long intergenic non-protein coding RNA 116	LINC00116	1,13
taste receptor, type 2, member 3	TAS2R3	1,13
transcription factor CP2-like 1	TFCP2L1	1,13
par-3 family cell polarity regulator	PARD3	1,13
PDZ and LIM domain 3	PDLIM3	1,13
spermatogenesis associated 2	SPATA2	1,13
coiled-coil domain containing 40	CCDC40	1,13
carboxypeptidase B1 (tissue)	CPB1	1,13
FCH and double SH3 domains 2	FCHSD2	1,13
otopetrin 2	OTOP2	1,13
OCIA domain containing 1	OCIAD1	1,13
aldehyde dehydrogenase 1 family, member A2	ALDH1A2	1,13
histamine receptor H4	HRH4	1,13
spermatogenesis associated 16	SPATA16	1,13
ring finger and WD repeat domain 2, E3 ubiquitin protein ligase	RFWD2	1,13
secretoglobin, family 2A, member 2	SCGB2A2	1,13
KIAA0196	KIAA0196	1,13
spectrin, beta, non-erythrocytic 2	SPTBN2	1,13
potassium channel, inwardly rectifying subfamily J, member 14	KCNJ14	1,13
enolase family member 4	ENO4	1,13
chromosome 9 open reading frame 170	C9orf170	1,13
chromosome 9 open reading frame 142	C9orf142	1,13

Homo sapiens vesicle-associated membrane protein 7 (VAMP7), transcript variant 2, mRNA.; Homo sapiens vesicle-associated membrane protein 7 (VAMP7), transcript variant 3, mRNA.; Homo sapiens vesicle-associated membrane protein 7 (VAMP7), transcript variant 1, mRNA.; Homo sapiens vesicle-associated membrane protein 7 (VAMP7), transcript variant 4, non-coding RNA.; Homo sapiens vesicle-associated membrane protein 7 (VAMP7), transcript variant 5, non-coding RNA.; vesicle-associated membrane protein 7 [Source:HGNC Symbol;Acc:HGNC:11486]; Homo sapiens vesicle-associated membrane protein 7, mRNA (cDNA clone MGC:64832 IMAGE:6503665), complete cds.	VAMP7	1,13
malectin	MLEC	1,13
chromosome 11 open reading frame 58	C11orf58	1,13
RAB36, member RAS oncogene family	RAB36	1,13
major histocompatibility complex, class II, DR beta 1	HLA-DRB1	1,13
zinc finger protein 226	ZNF226	1,13
secretory carrier membrane protein 5	SCAMP5	1,13
Memczak2013 ANTISENSE, coding, INTERNAL, intronic best transcript NM_001170794	BACH2	1,13
proline rich 3	PRR3	1,13
ribosomal protein S6 kinase, 90kDa, polypeptide 1	RPS6KA1	1,13
solute carrier family 7, member 6 opposite strand	SLC7A6OS	1,13
lens intrinsic membrane protein 2	LIM2	1,13
chromosome 11 open reading frame 21	C11orf21	1,13
MAGE family member A4	MAGEA4	1,13
patatin-like phospholipase domain containing 4	PNPLA4	1,13
ring finger protein 144A	RNF144A	1,13
CD200 receptor 1 like	CD200R1L	1,13
phosphoribosyl pyrophosphate synthetase 1	PRPS1	1,13
olfactory receptor, family 51, subfamily S, member 1	OR51S1	1,13
kelch domain containing 4	KLHDC4	1,13
hephaestin	HEPH	1,13
zinc finger, CCHC domain containing 12	ZCCHC12	1,13
Transcript Identified by AceView, Entrez Gene ID(s) 8328; novel transcript	RP11-295G24.4; GFI1B	1,13
dynein, axonemal, heavy chain 11	DNAH11	1,13
keratin 6C, type II	KRT6C	1,13
prefoldin subunit 5	PFDN5	1,13
NIMA-related kinase 2	NEK2	1,13
phospholamban	PLN	1,13
meprin A, beta	MEP1B	1,13
integrin, beta 2 (complement component 3 receptor 3 and 4 subunit)	ITGB2	1,13

nuclear pore complex interacting protein family, member B9	NPIPB9	1,13
chromosome 16 open reading frame 59	C16orf59	1,13
membrane protein, palmitoylated 6	MPP6	1,13
solute carrier family 2 (facilitated glucose transporter), member 6 [Source:HGNC Symbol;Acc:HGNC:11011]	SLC2A6	1,13
polyribonucleotide nucleotidyltransferase 1	PNPT1	1,13
SH3 domain and tetratricopeptide repeats 1	SH3TC1	1,13
limb development membrane protein 1-like	LMBR1L	1,13
lactamase, beta 2	LACTB2	1,13
StAR-related lipid transfer domain containing 5	STARD5	1,13
ring finger protein 121	RNF121	1,13
ring finger protein 145	RNF145	1,13
calcium channel, voltage-dependent, L type, alpha 1F subunit	CACNA1F	1,13
replication timing regulatory factor 1	RIF1	1,13
ret finger protein-like 1	RFPL1	1,13
transforming growth factor beta 3	TGFB3	1,13
surfeit 2	SURF2	1,13
glial fibrillary acidic protein	GFAP	1,13
olfactory receptor, family 2, subfamily M, member 4	OR2M4	1,13
apolipoprotein L, 3	APOL3	1,13
dystrophia myotonica-protein kinase	DMPK	1,13
nuclear transcription factor Y subunit gamma	NFYC	1,13
Memczak2013 ALT_ACCEPTOR, ALT_DONOR, coding, INTERNAL, intronic best transcript NM_138799	MBOAT2	1,13
small nuclear ribonucleoprotein, U11/U12 35kDa subunit	SNRNP35	1,13
vimentin	VIM	1,13
multiple PDZ domain protein	MPDZ	1,13
collagen, type XXIV, alpha 1	COL24A1	1,13
phosphatidylinositol glycan anchor biosynthesis class L	PIGL	1,13
BCL2-like 14 (apoptosis facilitator)	BCL2L14	1,13
transcription termination factor, RNA polymerase II	TTF2	1,13
bone morphogenetic protein 1	BMP1	1,13
early B-cell factor 3	EBF3	1,13
MDM4, p53 regulator	MDM4	1,13
cytoplasmic polyadenylation element binding protein 2	CPEB2	1,13
solute carrier family 17, member 4	SLC17A4	1,13
sushi domain containing 1	SUSD1	1,13
lin-52 DREAM MuvB core complex component	LIN52	1,13
zinc finger protein 570	ZNF570	1,13
ArfGAP with GTPase domain, ankyrin repeat and PH domain 5	AGAP5	1,13
transcription factor AP-2 gamma (activating enhancer binding protein 2 gamma)	TFAP2C	1,13
glucose-fructose oxidoreductase domain containing 2	GFOD2	1,13
mediator complex subunit 9	MED9	1,13
mitochondrial ribosomal protein L52	MRPL52	1,13

olfactory receptor, family 1, subfamily A, member 2	OR1A2	1,13
UBA domain containing 2	UBAC2	1,13
GRB2 associated regulator of MAPK1 1	GAREM1	1,13
polypyrimidine tract binding protein 2	PTBP2	1,13
nuclear receptor subfamily 2, group E, member 3	NR2E3	1,13
decapping mRNA 2	DCP2	1,13
Rho GTPase activating protein 39	ARHGAP39	1,13
SRY box 4	SOX4	1,13
sorting nexin 22	SNX22	1,13
germinal center-associated, signaling and motility-like	GCSAML	1,13
Memczak2013 ANTISENSE, CDS, coding, INTERNAL best transcript NM_001145304	KIAA1683	1,13
spindle apparatus coiled-coil protein 1	SPDL1	1,13
lipocalin-like 1	LCNL1	1,13
sideroflexin 5	SFXN5	1,13
creatine kinase, mitochondrial 2 (sarcomeric)	CKMT2	1,13
regulator of microtubule dynamics 2	RMDN2	1,13
casein kappa	CSN3	1,13
exostosin-like glycosyltransferase 1	EXTL1	1,13
natriuretic peptide receptor 1	NPR1	1,13
ADP-ribosylation factor related protein 1	ARFRP1	1,13
Memczak2013 ANTISENSE, CDS, coding, INTERNAL best transcript NM_153280; Transcript Identified by AceView	UBA1; tydee	1,13
kelch-like family member 12	KLHL12	1,13
polymerase (DNA directed), gamma	POLG	1,13
secretion regulating guanine nucleotide exchange factor	SERGEF	1,13
DnaJ (Hsp40) homolog, subfamily C, member 28	DNAJC28	1,13
zinc finger with KRAB and SCAN domains 7	ZKSCAN7	1,13
methyltransferase like 6	METTL6	1,13
kinase D-interacting substrate 220kDa	KIDINS220	1,13
X-ray repair complementing defective repair in Chinese hamster cells 1	XRCC1	1,13
C1GALT1 specific chaperone 1	C1GALT1C1	1,13
fibrinogen C domain containing 1	FIBCD1	1,13
E2F-associated phosphoprotein	EAPP	1,13
RAD51L3-RFFL readthrough; HCG2039718, isoform CRA_g; Uncharacterized protein [Source:UniProtKB/TrEMBL;Acc:K7EN88]	RAD51L3-RFFL	1,13
DnaJ (Hsp40) homolog, subfamily C, member 18	DNAJC18	1,13
hydroxysteroid (17-beta) dehydrogenase 4	HSD17B4	1,13
solute carrier family 22 (organic cation transporter), member 2	SLC22A2	1,13
coagulation factor XII (Hageman factor)	F12	1,13
selenophosphate synthetase 1	SEPHS1	1,13
olfactory receptor, family 5, subfamily D, member 16	OR5D16	1,13
LIM homeobox 2	LHX2	1,13

BCL2-like 14 (apoptosis facilitator)	BCL2L14	1,13
immunoglobulin superfamily, member 8	IGSF8	1,13
caldesmon 1	CALD1	1,13
secretin	SCT	1,13
solute carrier family 25 (mitochondrial iron transporter), member 28	SLC25A28	1,13
BRF2, RNA polymerase III transcription initiation factor 50 kDa subunit	BRF2	1,13
VENT homeobox	VENTX	1,13
vasoactive intestinal peptide receptor 2	VIPR2	1,13
abhydrolase domain containing 15	ABHD15	1,13
RNA binding motif protein 12B	RBM12B	1,13
rhomboid domain containing 1	RHBDD1	1,13
deiodinase, iodothyronine, type I	DIO1	1,12
Transcript Identified by AceView, Entrez Gene ID(s) 23144	ZC3H3	1,12
chromosome 11 open reading frame 49	C11orf49	1,12
adhesion G protein-coupled receptor L4	ADGRL4	1,12
beta-defensin 131-like	LOC100129216	1,12
DnaJ (Hsp40) homolog, subfamily C, member 3	DNAJC3	1,12
dynein heavy chain -like pseudogene; TEC	LOC730668; CITF22-92A6.2	1,12
syntaxin 18	STX18	1,12
nuclear receptor subfamily 1, group I, member 2	NR1I2	1,12
tetratricopeptide repeat domain 6	TTC6	1,12
phospholipid phosphatase 7 (inactive)	PLPP7	1,12
transketolase	TKT	1,12
alcohol dehydrogenase, iron containing 1; chromosome 8 open reading frame 46	ADHFE1; C8orf46	1,12
Memczak2013 ANTISENSE, CDS, coding, INTERNAL best transcript NM_156039	CSF3R	1,12
actinin, alpha 2	ACTN2	1,12
aldo-keto reductase family 7, member A2	AKR7A2	1,12
family with sequence similarity 89, member A; microRNA 1182	FAM89A; MIR1182	1,12
thioredoxin reductase 2	TXNRD2	1,12
leucine carboxyl methyltransferase 1	LCMT1	1,12
contactin associated protein-like 3	CNTNAP3	1,12
RAB37, member RAS oncogene family	RAB37	1,12
Transcript Identified by AceView, Entrez Gene ID(s) 79875	THSD4	1,12
hedgehog acyltransferase	HHAT	1,12
thyroid peroxidase	TPO	1,12
ATP binding cassette subfamily B member 11	ABCB11	1,12
serine/arginine repetitive matrix 1	SRRM1	1,12
myotilin	MYOT	1,12
tripartite motif containing 58	TRIM58	1,12

SWI/SNF related, matrix associated, actin dependent regulator of chromatin, subfamily b, member 1	SMARCB1	1,12
cancer susceptibility candidate 1	CASC1	1,12
phosphopantothenoylcysteine synthetase; coiled-coil domain containing 30	PPCS; CCDC30	1,12
transmembrane protein 50B	TMEM50B	1,12
enhancer of mRNA decapping 3	EDC3	1,12
tripartite motif containing 52	TRIM52	1,12
cyclin-dependent kinase 19	CDK19	1,12
sex comb on midleg-like 4 (Drosophila)	SCML4	1,12
polymerase (RNA) III (DNA directed) polypeptide A, 155kDa	POLR3A	1,12
ALG1, chitobiosyldiphosphodolichol beta-mannosyltransferase; NAGPA antisense RNA 1	ALG1; NAGPA-AS1	1,12
family with sequence similarity 160, member A2	FAM160A2	1,12
Rho-related BTB domain containing 1	RHOBTB1	1,12
tectonic family member 1	TCTN1	1,12
collagen, type XXVII, alpha 1	COL27A1	1,12
stabilin 2	STAB2	1,12
zinc finger, DHHC-type containing 21	ZDHHC21	1,12
eukaryotic translation initiation factor 4E binding protein 2	EIF4EBP2	1,12
tripartite motif containing 46	TRIM46	1,12
protein tyrosine phosphatase, non-receptor type 6	PTPN6	1,12
anterior gradient 3, protein disulphide isomerase family member	AGR3	1,12
pyruvate dehydrogenase kinase, isozyme 4	PDK4	1,12
glucuronidase, beta pseudogene 2	GUSBP2	1,12
retinitis pigmentosa 9 (autosomal dominant)	RP9	1,12
NIMA-related kinase 3	NEK3	1,12
G protein regulated inducer of neurite outgrowth 2	GPRIN2	1,12
calcitonin-related polypeptide alpha	CALCA	1,12
polycomb group ring finger 1	PCGF1	1,12
ATPase, H+ transporting, lysosomal V0 subunit a2	ATP6V0A2	1,12
upregulator of cell proliferation; URGCP-MRPS24 readthrough	URGCP; URGCP-MRPS24	1,12
sorting nexin 17	SNX17	1,12
cytochrome c oxidase subunit VIIb2	COX7B2	1,12
zinc finger protein 334	ZNF334	1,12
UDP-GlcNAc:betaGal beta-1,3-N-acetylglucosaminyltransferase 8	B3GNT8	1,12
tensin 2	TNS2	1,12
olfactory receptor, family 4, subfamily A, member 47	OR4A47	1,12
PYCARD antisense RNA 1	PYCARD-AS1	1,12
leptin receptor overlapping transcript-like 1	LEPROTL1	1,12
F-box protein 28	FBXO28	1,12

mesoderm posterior bHLH transcription factor 2	MESP2	1,12
SPT20 homolog, SAGA complex component-like 2	SUPT20HL2	1,12
methionine sulfoxide reductase B1	MSRB1	1,12
T-box 15	TBX15	1,12
immunoglobulin superfamily, member 22	IGSF22	1,12
neurofilament, light polypeptide	NEFL	1,12
hepatocyte growth factor (hepapoietin A; scatter factor)	HGF	1,12
nuclear factor I/X (CCAAT-binding transcription factor)	NFIX	1,12
myosin, heavy chain 13, skeletal muscle	MYH13	1,12
RNA binding motif, single stranded interacting protein 3	RBMS3	1,12
vacuolar protein sorting 13 homolog C (S. cerevisiae)	VPS13C	1,12
potassium channel, voltage gated KQT-like subfamily Q, member 2	KCNQ2	1,12
solute carrier family 34 (type II sodium/phosphate cotransporter), member 2	SLC34A2	1,12
Fc receptor-like 1	FCRL1	1,12
zinc finger, DHHC-type containing 23	ZDHHC23	1,12
glutamate receptor, ionotropic, N-methyl-D-aspartate 3B	GRIN3B	1,12
kelch domain containing 2	KLHDC2	1,12
glucosidase, beta, acid	GBA	1,12
GEM interacting protein	GMIP	1,12
sorting nexin 20	SNX20	1,12
calcium responsive transcription factor	CARF	1,12
wingless-type MMTV integration site family, member 6	WNT6	1,12
zinc finger protein 676	ZNF676	1,12
chromosome X open reading frame 36	CXorf36	1,12
SRY box 1	SOX1	1,12
zinc finger CCCH-type containing 4	ZC3H4	1,12
tubulin folding cofactor E	TBCE	1,12
intraflagellar transport 88	IFT88	1,12
nuclear pore complex interacting protein family, member B8	NPIPB8	1,12
neurochondrin	NCDN	1,12
O-6-methylguanine-DNA methyltransferase	MGMT	1,12
CD1c molecule	CD1C	1,12
keratin 26, type I	KRT26	1,12
keratin associated protein 22-2	KRTAP22-2	1,12
tubulin, alpha 3c	TUBA3C	1,12
Nik related kinase	NRK	1,12
VPS33B interacting protein, apical-basolateral polarity regulator, spe-39 homolog	VIPAS39	1,12
cytochrome P450, family 27, subfamily A, polypeptide 1	CYP27A1	1,12
chromosome 16 open reading frame 95	C16orf95	1,12
cyclin L2	CCNL2	1,12
keratin 17, type I	KRT17	1,12
La ribonucleoprotein domain family, member 1	LARP1	1,12

trafficking protein particle complex 2	TRAPPC2	1,12
tripartite motif containing 22	TRIM22	1,12
small integral membrane protein 4	SMIM4	1,12
fragile X mental retardation 1	FMR1	1,12
transglutaminase 4	TGM4	1,12
autophagy related 10	ATG10	1,12
Rab interacting lysosomal protein	RILP	1,12
Transcript Identified by AceView, Entrez Gene ID(s) 65980	BRD9	1,12
transient receptor potential cation channel, subfamily C, member 5	TRPC5	1,12
bestrophin 4	BEST4	1,12
calpastatin	CAST	1,12
ORAI calcium release-activated calcium modulator 1	ORAI1	1,12
myotubularin related protein 1	MTMR1	1,12
synovial sarcoma translocation gene on chromosome 18-like 2	SS18L2	1,12
methyltransferase like 25	METTL25	1,12
CDKN2A interacting protein N-terminal like	CDKN2AIPNL	1,12
K(lysine) acetyltransferase 2A	KAT2A	1,12
olfactory receptor, family 5, subfamily W, member 2	OR5W2	1,12
EF-hand calcium binding domain 2	EFCAB2	1,12
translocase of inner mitochondrial membrane 44 homolog (yeast)	TIMM44	1,12
vacuolar protein sorting 39 homolog (S. cerevisiae)	VPS39	1,12
F-box protein 10	FBXO10	1,12
glycine receptor alpha 3	GLRA3	1,12
Alport syndrome, mental retardation, midface hypoplasia and elliptocytosis chromosomal region gene 1	AMMECR1	1,12
CDKN1A interacting zinc finger protein 1	CIZ1	1,12
pleckstrin homology domain containing, family G (with RhoGef domain) member 4B	PLEKHG4B	1,12
coenzyme Q2 4-hydroxybenzoate polyprenyltransferase	COQ2	1,12
mitogen-activated protein kinase 8 interacting protein 1	MAPK8IP1	1,12
interleukin 3 receptor, alpha (low affinity)	IL3RA	1,12
choline O-acetyltransferase	CHAT	1,12
chromosome 6 open reading frame 120	C6orf120	1,12
nuclear receptor subfamily 3, group C, member 2	NR3C2	1,12
fer (fps/fes related) tyrosine kinase	FER	1,12
exosome component 1	EXOSC1	1,12
chromosome 10 open reading frame 142	C10orf142	1,12
A kinase (PRKA) anchor protein 7	AKAP7	1,12
arrestin domain containing 5	ARRDC5	1,12
WD repeat domain 64	WDR64	1,12
internexin neuronal intermediate filament protein, alpha	INA	1,12
solute carrier family 6 (neutral amino acid transporter), member 17	SLC6A17	1,12

golgin A5	GOLGA5	1,12
dolichyl-phosphate mannosyltransferase polypeptide 2, regulatory subunit	DPM2	1,12
methionine sulfoxide reductase A	MSRA	1,12
SIX homeobox 5	SIX5	1,12
relaxin/insulin-like family peptide receptor 1	RXFP1	1,12
exonuclease 5	EXO5	1,12
gypsy retrotransposon integrase 1	GIN1	1,12
neugrin, neurite outgrowth associated; tubulin tyrosine ligase-like family member 13, pseudogene	NGRN; TTLL13P	1,12
serine/threonine kinase-like domain containing 1 [Source:HGNC Symbol;Acc:HGNC:28669]	STKLD1	1,12
ArfGAP with GTPase domain, ankyrin repeat and PH domain 9; BMS1 ribosome biogenesis factor pseudogene 6	AGAP9; BMS1P6	1,12
TNFAIP3 interacting protein 3	TNIP3	1,12
heat shock 105kDa/110kDa protein 1	HSPH1	1,12
deleted in lymphocytic leukemia, 7	DLEU7	1,12
Memczak2013 ANTISENSE, CDS, coding, INTERNAL best transcript NM_001039590	USP9X	1,12
interferon, alpha 14	IFNA14	1,12
DnaJ (Hsp40) homolog, subfamily C, member 9	DNAJC9	1,12
family with sequence similarity 192, member A	FAM192A	1,12
mitochondrial ribosomal protein L38	MRPL38	1,12
mediator complex subunit 11	MED11	1,12
inositol polyphosphate multikinase	IPMK	1,12
tetratricopeptide repeat domain 9	TTC9	1,12
adipogenesis regulatory factor; ankyrin repeat and GTPase domain Arf GTPase activating protein 11; BMS1 ribosome biogenesis factor pseudogene 3	ADIRF; AGAP11; BMS1P3	1,12
Fc fragment of IgG, low affinity IIc, receptor for (CD32) (gene/pseudogene)	FCGR2C	1,12
olfactory receptor, family 6, subfamily C, member 2	OR6C2	1,12
nascent polypeptide-associated complex alpha subunit	NACA	1,12
MPN domain containing	MPND	1,12
alpha-methylacyl-CoA racemase	AMACR	1,12
protein tyrosine phosphatase, non-receptor type 18 (brain-derived)	PTPN18	1,12
Zhang2013 ALT_ACCEPTOR, ALT_DONOR, coding, INTERNAL, intronic best transcript NM_020338	ZMIZ1	1,12
epoxide hydrolase 2, cytoplasmic	EPHX2	1,12
Memczak2013 ALT_DONOR, coding, INTERNAL, intronic best transcript NM_021078	KAT2A	1,12
matrix metallopeptidase 17 (membrane-inserted)	MMP17	1,12
taxilin alpha	TXLNA	1,12
microtubule-associated protein 9	MAP9	1,12

SRY box 14	SOX14	1,12
MCF.2 cell line derived transforming sequence	MCF2	1,12
UBX domain protein 8	UBXN8	1,12
sodium channel, non voltage gated 1 delta subunit	SCNN1D	1,12
prostaglandin E receptor 2	PTGER2	1,12
sulfotransferase family 6B member 1	SULT6B1	1,12
cancer/testis antigen family 47, member A9; cancer/testis antigen family 47, member A4; cancer/testis antigen family 47, member A11	CT47A9; CT47A4; CT47A11	1,12
cancer/testis antigen family 47, member A3; cancer/testis antigen family 47, member A10	CT47A3; CT47A10	1,12
cancer/testis antigen family 47, member A10; cancer/testis antigen family 47, member A8; cancer/testis antigen family 47, member A9	CT47A10; CT47A8; CT47A9	1,12
cancer/testis antigen family 47, member A6; cancer/testis antigen family 47, member A4	CT47A6; CT47A4	1,12
cancer/testis antigen family 47, member A3	CT47A3	1,12
cancer/testis antigen family 47, member A2	CT47A2	1,12
cancer/testis antigen family 47, member A12; cancer/testis antigen family 47, member A8	CT47A12; CT47A8	1,12
chromosome 9 open reading frame 62	C9orf62	1,12
survival motor neuron domain containing 1	SMNDC1	1,12
coiled-coil domain containing 13	CCDC13	1,12
Kruppel-like factor 7 (ubiquitous)	KLF7	1,12
latent transforming growth factor beta binding protein 2	LTBP2	1,12
c-src tyrosine kinase	CSK	1,12
neurexin 2	NRXN2	1,12
interleukin 15	IL15	1,12
proline, glutamate and leucine rich protein 1	PELP1	1,12
cyclin and CBS domain divalent metal cation transport mediator 4	CNNM4	1,12
S-phase kinase-associated protein 1	SKP1	1,12
nucleotide binding protein-like	NUBPL	1,12
2,4-dienoyl-CoA reductase 2, peroxisomal	DECR2	1,12
nuclear factor of activated T-cells, cytoplasmic, calcineurin-dependent 4	NFATC4	1,12
SNF8, ESCRT-II complex subunit	SNF8	1,12
histone cluster 2, H2ab	HIST2H2AB	1,12
roundabout guidance receptor 3	ROBO3	1,12
Zhang2013 ALT_ACCEPTOR, ALT_DONOR, coding, INTERNAL, intronic best transcript NM_014238	KSR1	1,12
dipeptidyl-peptidase 6	DPP6	1,12
zinc finger protein 585B	ZNF585B	1,12
ATPase, Ca++ transporting, type 2C, member 1	ATP2C1	1,12
olfactory receptor, family 2, subfamily A, member 4	OR2A4	1,12

ancient ubiquitous protein 1	AUP1	1,12
zinc finger protein 569	ZNF569	1,12
Transcript Identified by AceView, Entrez Gene ID(s) 23250	ATP11A	1,12
golgi brefeldin A resistant guanine nucleotide exchange factor 1	GBF1	1,12
transmembrane BAX inhibitor motif containing 1; microRNA 6513	TMBIM1; MIR6513	1,12
adhesion G protein-coupled receptor F2	ADGRF2	1,12
phosphodiesterase 7A	PDE7A	1,12
zinc finger protein 268	ZNF268	1,12
thromboxane A2 receptor	TBXA2R	1,12
DGCR8 microprocessor complex subunit; microRNA 1306; microRNA 3618	DGCR8; MIR1306; MIR3618	1,12
programmed cell death 1	PDCD1	1,12
TAO kinase 1; microRNA 4523	TAOK1; MIR4523	1,12
C-type lectin domain family 17, member A	CLEC17A	1,12
Jeck2013 ALT_DONOR, coding, INTERNAL, intronic best transcript NM_004515	ILF2	1,12
serpin peptidase inhibitor, clade A (alpha-1 antiproteinase, antitrypsin), member 5	SERPINA5	1,12
Usher syndrome 1C	USH1C	1,12
interleukin 17B	IL17B	1,12
v-ets avian erythroblastosis virus E26 oncogene homolog 1	ETS1	1,12
collagen, type VI, alpha 3	COL6A3	1,12
adaptor-related protein complex 1 sigma 1 subunit	AP1S1	1,12
regulatory factor X-associated ankyrin-containing protein	RFXANK	1,12
Rap guanine nucleotide exchange factor 1	RAPGEF1	1,12
GRAM domain containing 2	GRAMD2	1,12
long intergenic non-protein coding RNA 1537	LINC01537	1,12
RAB3 GTPase activating protein subunit 1 (catalytic)	RAB3GAP1	1,12
SH2B adaptor protein 2	SH2B2	1,12
leukemia inhibitory factor	LIF	1,12
regulatory factor X, 5 (influences HLA class II expression)	RFX5	1,12
Homo sapiens proline dehydrogenase (oxidase) 1 (PRODH), transcript variant 2, mRNA.; Homo sapiens proline dehydrogenase (oxidase) 1 (PRODH), transcript variant 1, mRNA.; proline dehydrogenase 1, mitochondrial; Homo sapiens proline dehydrogenase (oxidase) 1, mRNA (cDNA clone MGC:148079 IMAGE:40108133), complete cds.	PRODH; LOC102724788; AC007325.2	1,12
MIF4G domain containing	MIF4GD	1,12
lipase, member I	LIPI	1,12
otoancorin	OTOA	1,12
protogenin	PRTG	1,12
polymerase (RNA) III (DNA directed) polypeptide B	POLR3B	1,12

clathrin heavy chain linker domain containing 1	CLHC1	1,12
coiled-coil domain containing 65	CCDC65	1,12
LIM domain only 1 (rhombotin 1)	LMO1	1,12
Transcript Identified by AceView, Entrez Gene ID(s) 55008; novel transcript	RP11-10L7.1; HERC6	1,12
zymogen granule protein 16	ZG16	1,12
cadherin 13	CDH13	1,12
KRTAP5-1/KRTAP5-2 antisense RNA 1	KRTAP5-AS1	1,12
fibronectin type III domain containing 4	FNDC4	1,12
neuronal guanine nucleotide exchange factor	NGEF	1,12
integrin alpha 5	ITGA5	1,12
protocadherin 20	PCDH20	1,12
SMC5-SMC6 complex localization factor 1	SLF1	1,12
sprouty-related, EVH1 domain containing 3	SPRED3	1,12
cysteine rich with EGF-like domains 2	CRELD2	1,12
transforming growth factor beta 1	TGFB1	1,12
chromosome 2 open reading frame 27B	C2orf27B	1,12
testis expressed 12	TEX12	1,12
ecotropic viral integration site 5-like	EVI5L	1,12
family with sequence similarity 111, member A	FAM111A	1,12
metallothionein 1H	MT1H	1,12
tumor necrosis factor receptor superfamily, member 13C; microRNA 378i	TNFRSF13C; MIR378I	1,12
Transcript Identified by AceView, Entrez Gene ID(s) 3069	HDLBP	1,12
pleckstrin homology domain containing, family M, member 3	PLEKHM3	1,12
cyclin-dependent kinase inhibitor 1B (p27, Kip1)	CDKN1B	1,12
chromosome 7 open reading frame 76; split hand/foot malformation (ectrodactyly) type 1	C7orf76; SHFM1	1,12
potassium channel, two pore domain subfamily K, member 18	KCNK18	1,12
NK2 homeobox 4	NKX2-4	1,12
chromosome X open reading frame 21	CXorf21	1,12
COBW domain containing 5	CBWD5	1,12
glutamate-cysteine ligase, modifier subunit	GCLM	1,12
G protein-coupled receptor 142	GPR142	1,12
KIAA0391	KIAA0391	1,12
leucine rich repeat and fibronectin type III domain containing 5	LRFN5	1,12
chromosome 1 open reading frame 94	C1orf94	1,12
lymphocyte-activation gene 3	LAG3	1,12
crystallin alpha B	CRYAB	1,12
cytochrome c oxidase subunit VIa polypeptide 2	COX6A2	1,12
Kruppel-like factor 14	KLF14	1,12
discs, large homolog 1 (Drosophila)	DLG1	1,12
serine/threonine kinase 11	STK11	1,12
pellino E3 ubiquitin protein ligase family member 2	PELI2	1,12
AT rich interactive domain 1B (SWI1-like)	ARID1B	1,12

NADH dehydrogenase (ubiquinone) complex I, assembly factor 7	NDUFAF7	1,12
polymerase (DNA-directed), delta 3, accessory subunit	POLD3	1,12
immunoglobulin-like domain containing receptor 1	ILDR1	1,12
siah E3 ubiquitin protein ligase family member 3	SIAH3	1,12
myosin IXB	MYO9B	1,12
REST corepressor 1	RCOR1	1,12
calcium channel, voltage-dependent, gamma subunit 2	CACNG2	1,12
mucin 4, cell surface associated	MUC4	1,12
calcineurin-like EF-hand protein 2	CHP2	1,12
calsequestrin 1 (fast-twitch, skeletal muscle)	CASQ1	1,12
slingshot protein phosphatase 1	SSH1	1,12
zinc finger and SCAN domain containing 23	ZSCAN23	1,12
Fc receptor-like 3	FCRL3	1,12
tripartite motif containing 14	TRIM14	1,12
tRNA-yW synthesizing protein 3 homolog (S. cerevisiae)	TYW3	1,12
heart and neural crest derivatives expressed 1	HAND1	1,12
single-strand-selective monofunctional uracil-DNA glycosylase 1	SMUG1	1,12
Jeck2013 ALT_ACCEPTOR, ALT_DONOR, coding, INTERNAL, intronic best transcript NM_032531	KIRREL3	1,12
glutaredoxin 2	GLRX2	1,12
chromosome 1 open reading frame 210	C1orf210	1,12
hypoxia up-regulated 1	HYOU1	1,12
Ras association and DIL domains	RADIL	1,12
interleukin 32	IL32	1,12
myelin transcription factor 1	MYT1	1,12
family with sequence similarity 174, member B	FAM174B	1,12
hormonally up-regulated Neu-associated kinase	HUNK	1,12
chromosome 16 open reading frame 92	C16orf92	1,12
protein phosphatase 4, regulatory subunit 1	PPP4R1	1,12
Zhang2013 ALT_ACCEPTOR, ALT_DONOR, coding, INTERNAL, intronic best transcript NM_022720	DGCR8	1,12
period circadian clock 1; microRNA 6883	PER1; MIR6883	1,12
epithelial cell transforming 2 like	ECT2L	1,12
aldo-keto reductase family 1, member C8, pseudogene	AKR1C8P	1,12
schlafen family member 14	SLFN14	1,12
sperm acrosome associated 5	SPACA5	1,12
olfactory receptor, family 14, subfamily A, member 16	OR14A16	1,12
UDP-GlcNAc:betaGal beta-1,3-N-acetylglucosaminyltransferase 4	B3GNT4	1,12
SIX homeobox 6	SIX6	1,12
interferon regulatory factor 2	IRF2	1,12
teashirt zinc finger homeobox 3	TSHZ3	1,12
TAF7 RNA polymerase II, TATA box binding protein (TBP)-associated factor, 55kDa	TAF7	1,12
serine/threonine kinase 33	STK33	1,12

RNA terminal phosphate cyclase-like 1	RCL1	1,12
family with sequence similarity 206, member A	FAM206A	1,12
fibroblast growth factor 18	FGF18	1,12
anoctamin 10	ANO10	1,12
ceramide synthase 5	CERS5	1,12
TAP binding protein-like	TAPBPL	1,12
solute carrier family 18 (vesicular acetylcholine transporter), member 3	SLC18A3	1,12
mastermind-like transcriptional coactivator 1	MAML1	1,12
lipocalin 6	LCN6	1,12
ribosomal RNA processing 36	RRP36	1,12
early endosome antigen 1	EEA1	1,12
G protein-coupled receptor, class C, group 5, member D	GPRC5D	1,12
musculin	MSC	1,12
renalase, FAD-dependent amine oxidase	RNLS	1,12
acylphosphatase 1, erythrocyte (common) type	ACYP1	1,12
NADH dehydrogenase (ubiquinone) 1 alpha subcomplex, 10, 42kDa	NDUFA10	1,12
POTE ankyrin domain family, member D	POTED	1,12
unc-50 homolog (C. elegans)	UNC50	1,12
ULK4 pseudogene 1; ULK4 pseudogene 2	ULK4P1; ULK4P2	1,12
chromosome 20 open reading frame 202	C20orf202	1,12
chromosome 9 open reading frame 173	C9orf173	1,12
leucine zipper, down-regulated in cancer 1	LDOC1	1,12
thyrotrophic embryonic factor	TEF	1,12
APEX nuclease (apurinic/apyrimidinic endonuclease) 2	APEX2	1,12
nuclear pore complex interacting protein family, member B11	NPIPB11	1,12
family with sequence similarity 78, member B	FAM78B	1,12
melanin-concentrating hormone receptor 1	MCHR1	1,12
glucose-fructose oxidoreductase domain containing 1	GFOD1	1,12
ADAM metallopeptidase domain 2	ADAM2	1,12
family with sequence similarity 227, member A	FAM227A	1,12
HIG1 hypoxia inducible domain family, member 2B	HIGD2B	1,12
histone cluster 1, H3c	HIST1H3C	1,12
Transcript Identified by AceView, Entrez Gene ID(s) 114794	ELFN2	1,12
matrix metallopeptidase 13	MMP13	1,12
Fc fragment of IgG, low affinity IIa, receptor (CD32)	FCGR2A	1,12
WDYHV motif containing 1	WDYHV1	1,12
transmembrane and coiled-coil domains 6	TMCO6	1,12
calcium channel, voltage-dependent, gamma subunit 4	CACNG4	1,12
sphingomyelin phosphodiesterase, acid-like 3A	SMPDL3A	1,12
zinc finger protein 717	ZNF717	1,12
dihydrouridine synthase 4-like	DUS4L	1,12
aldo-keto reductase family 1, member B1 (aldose reductase)	AKR1B1	1,12

annexin A8-like 1; annexin A8	ANXA8L1; ANXA8	1,12
tousled-like kinase 2	TLK2	1,12
spexin hormone	SPX	1,12
zinc finger protein 273	ZNF273	1,12
ubiquilin 4	UBQLN4	1,12
spermatogenesis associated 22	SPATA22	1,12
aminoadipate-semialdehyde synthase	AASS	1,12
PHD finger protein 14	PHF14	1,12
small ArfGAP2	SMAP2	1,12
small integral membrane protein 12	SMIM12	1,12
variable charge, X-linked 3A; variable charge, X-linked	VCX3A; VCX	1,12
cadherin, EGF LAG seven-pass G-type receptor 3; microRNA 4793	CELSR3; MIR4793	1,12
PSMA3 antisense RNA 1	PSMA3-AS1	1,12
CD84 molecule	CD84	1,12
leucine aminopeptidase 3	LAP3	1,12
osteocrin	OSTN	1,12
zinc finger protein 572	ZNF572	1,12
unc-79 homolog (C. elegans)	UNC79	1,12
3-phosphoadenosine 5-phosphosulfate synthase 2	PAPSS2	1,12
ring finger protein 38	RNF38	1,12
chromosome 16 open reading frame 46	C16orf46	1,12
myotubularin related protein 9	MTMR9	1,12
inositol polyphosphate-5-phosphatase D	INPP5D	1,12
lysosomal trafficking regulator	LYST	1,12
chloride channel, nucleotide-sensitive, 1A	CLNS1A	1,12
Rho guanine nucleotide exchange factor 10	ARHGEF10	1,12
long intergenic non-protein coding RNA 1343	LINC01343	1,12
phosphofructokinase, muscle	PFKM	1,12
zinc finger and SCAN domain containing 5B	ZSCAN5B	1,12
kinesin family member 27	KIF27	1,12
STEAP family member 4	STEAP4	1,12
leucine rich repeat neuronal 2	LRRN2	1,12
xin actin binding repeat containing 1	XIRP1	1,12
Memczak2013 ANTISENSE, CDS, coding, INTERNAL best transcript NM_000714	TSPO	1,12
DnaJ (Hsp40) homolog, subfamily B, member 13	DNAJB13	1,12
chemokine (C-C motif) ligand 24	CCL24	1,12
nuclear fragile X mental retardation protein interacting protein 1	NUFIP1	1,12
solute carrier family 22, member 15	SLC22A15	1,12
zinc finger CCCH-type containing 7A	ZC3H7A	1,12
protein associated with topoisomerase II homolog 2 (yeast)	PATL2	1,12
tubulin tyrosine ligase-like family member 5	TTLL5	1,12

crystallin gamma S	CRYGS	1,12
NADH dehydrogenase (ubiquinone) complex I, assembly factor 6	NDUFAF6	1,12
transmembrane protein 59	TMEM59	1,12
protein phosphatase 4, catalytic subunit	PPP4C	1,12
tyrosyl-tRNA synthetase 2, mitochondrial	YARS2	1,11
proline rich 35	PRR35	1,11
ets variant 3	ETV3	1,11
gamma-glutamyltransferase 5	GGT5	1,11
N-terminal EF-hand calcium binding protein 1	NECAB1	1,11
fibroblast growth factor binding protein 3	FGFBP3	1,11
MAGE family member C2	MAGEC2	1,11
centrosomal protein 192kDa	CEP192	1,11
S100 calcium binding protein A12	S100A12	1,11
adipogenin	ADIG	1,11
chromosome 19 open reading frame 84	C19orf84	1,11
fibrillarin	FBL	1,11
olfactory receptor, family 52, subfamily D, member 1	OR52D1	1,11
excision repair cross-complementation group 6-like 2	ERCC6L2	1,11
pepsinogen 4, group I (pepsinogen A)	PGA4	1,11
PDZ domain containing 4	PDZD4	1,11
chromosome 5 open reading frame 64	C5orf64	1,11
GTPase, IMAP family member 4	GIMAP4	1,11
Homo sapiens thymosin beta 15B (TMSB15B), mRNA.; Thymosin beta-15B [Source:UniProtKB/Swiss-Prot;Acc:P0CG35]	TMSB15B; RP11-722G7.1	1,11
asteroid homolog 1 (Drosophila)	ASTE1	1,11
retinoic acid early transcript 1E	RAET1E	1,11
coiled-coil domain containing 175	CCDC175	1,11
sestrin 3	SESN3	1,11
developing brain homeobox 2	DBX2	1,11
COP9 signalosome subunit 6	COPS6	1,11
C1q and tumor necrosis factor related protein 9B	C1QTNF9B	1,11
vacuolar protein sorting 13 homolog A (S. cerevisiae)	VPS13A	1,11
salt-inducible kinase 1	SIK1	1,11
ORMDL sphingolipid biosynthesis regulator 2	ORMDL2	1,11
chromosome 3 open reading frame 49	C3orf49	1,11
sphingosine-1-phosphate lyase 1	SGPL1	1,11
olfactory receptor, family 1, subfamily N, member 2	OR1N2	1,11
cytochrome P450, family 20, subfamily A, polypeptide 1	CYP20A1	1,11
dynein, axonemal, heavy chain 17	DNAH17	1,11
kinesin family member C3	KIFC3	1,11
potassium channel modulatory factor 1	KCMF1	1,11
zinc finger and SCAN domain containing 4	ZSCAN4	1,11
BEN domain containing 7	BEND7	1,11
protein phosphatase, Mg2+/Mn2+ dependent, 1G	PPM1G	1,11
RNA binding motif protein 22	RBM22	1,11

G protein-coupled receptor, class C, group 6, member A	GPRC6A	1,11
olfactory receptor, family 5, subfamily D, member 13 (gene/pseudogene)	OR5D13	1,11
family with sequence similarity 19 (chemokine (C-C motif)-like), member A1	FAM19A1	1,11
S-antigen; retina and pineal gland (arrestin)	SAG	1,11
suppressor of fused homolog (Drosophila)	SUFU	1,11
transcriptional adaptor 3	TADA3	1,11
carbohydrate sulfotransferase 10	CHST10	1,11
fibronectin type III and SPRY domain containing 2	FSD2	1,11
Fanconi anemia complementation group G	FANCG	1,11
zinc finger protein 737	ZNF737	1,11
POU class 6 homeobox 2	POU6F2	1,11
chromosome 16 open reading frame 90	C16orf90	1,11
general transcription factor IIF subunit 1	GTF2F1	1,11
solute carrier family 30 (zinc transporter), member 2	SLC30A2	1,11
chromosome 9 open reading frame 129	C9orf129	1,11
zinc finger protein 821	ZNF821	1,11
Mediterranean fever	MEFV	1,11
caveolin 2	CAV2	1,11
WSC domain containing 1	WSCD1	1,11
melan-A	MLANA	1,11
family with sequence similarity 118, member B	FAM118B	1,11
von Willebrand factor A domain containing 5A	VWA5A	1,11
steroid 5 alpha-reductase 3	SRD5A3	1,11
microtubule interacting and trafficking domain containing 1	MITD1	1,11
transmembrane protein 114	TMEM114	1,11
VPS26 retromer complex component B	VPS26B	1,11
Transcript Identified by AceView, Entrez Gene ID(s) 10450	PPIE	1,11
GRB2-related adaptor protein	GRAP	1,11
adenylate kinase 5	AK5	1,11
zinc finger protein 587B	ZNF587B	1,11
syntaxin 1B	STX1B	1,11
small integral membrane protein 8	SMIM8	1,11
protein phosphatase 2, regulatory subunit B, alpha	PPP2R5A	1,11
family with sequence similarity 71, member F1	FAM71F1	1,11
tenascin XA (pseudogene)	TNXA	1,11
proteasome subunit alpha 6	PSMA6	1,11
C1QTNF3-AMACR readthrough (NMD candidate)	C1QTNF3-AMACR	1,11
transmembrane protein with EGF-like and two follistatin-like domains 1	TMEFF1	1,11
peptidyl-tRNA hydrolase domain containing 1	PTRHD1	1,11
phosphatidylinositol-4,5-bisphosphate 3-kinase, catalytic subunit gamma	PIK3CG	1,11

Memczak2013 ANTISENSE, coding, INTERNAL, intronic best transcript NM_032131	ARMC2	1,11
nucleosome assembly protein 1-like 1	NAP1L1	1,11
family with sequence similarity 201, member A	FAM201A	1,11
regulating synaptic membrane exocytosis 1	RIMS1	1,11
matrix metallopeptidase 24 (membrane-inserted)	MMP24	1,11
PML-RARA regulated adaptor molecule 1	PRAM1	1,11
protein phosphatase 1, regulatory subunit 32	PPP1R32	1,11
leukocyte receptor tyrosine kinase	LTK	1,11
frizzled class receptor 3	FZD3	1,11
tRNA methyltransferase 61A	TRMT61A	1,11
zinc finger and BTB domain containing 2	ZBTB2	1,11
protein kinase C, eta	PRKCH	1,11
ubiquitin specific peptidase 21	USP21	1,11
arylsulfatase family, member I	ARSI	1,11
testis expressed 19	TEX19	1,11
Transcript Identified by AceView, Entrez Gene ID(s) 10777	ARPP21	1,11
CSE1 chromosome segregation 1-like (yeast)	CSE1L	1,11
Fanconi anemia complementation group F	FANCF	1,11
polymerase (RNA) III (DNA directed) polypeptide G (32kD)	POLR3G	1,11
glycoprotein integral membrane 1	GINM1	1,11
ceramide synthase 2	CERS2	1,11
cytochrome P450, family 2, subfamily D, polypeptide 6	CYP2D6	1,11
lectin, galactoside-binding, soluble, 4	LGALS4	1,11
chromosome 6 open reading frame 10	C6orf10	1,11
5-nucleotidase, ecto (CD73)	NT5E	1,11
CMT1A duplicated region transcript 1	CDRT1	1,11
apolipoprotein B mRNA editing enzyme, catalytic polypeptide-like 4 (putative)	APOBEC4	1,11
TATA box binding protein like 2	TBPL2	1,11
transporter 1, ATP-binding cassette, sub-family B (MDR/TAP)	TAP1	1,11
lipase, family member M	LIPM	1,11
Kv channel interacting protein 1	KCNIP1	1,11
DnaJ (Hsp40) homolog, subfamily B, member 4	DNAJB4	1,11
ER membrane protein complex subunit 9	EMC9	1,11
spermatogenesis associated 8	SPATA8	1,11
family with sequence similarity 131, member A	FAM131A	1,11
PHD and ring finger domains 1	PHRF1	1,11
methyl-CpG binding domain protein 3-like 2	MBD3L2	1,11
phospholipase C, zeta 1	PLCZ1	1,11
coiled-coil-helix-coiled-coil-helix domain containing 3	CHCHD3	1,11
autophagy related 16-like 2	ATG16L2	1,11
tripartite motif containing 27	TRIM27	1,11
SRY box 15	SOX15	1,11
phosphodiesterase 1C, calmodulin-dependent 70kDa	PDE1C	1,11

serpin peptidase inhibitor, clade B (ovalbumin), member 1	SERPINB1	1,11
methyltransferase like 24	METTL24	1,11
late cornified envelope-like proline-rich 1	LELP1	1,11
free fatty acid receptor 2	FFAR2	1,11
hyperpolarization activated cyclic nucleotide gated potassium channel 4	HCN4	1,11
upstream binding protein 1 (LBP-1a)	UBP1	1,11
chromosome 19 open reading frame 68	C19orf68	1,11
solute carrier family 25, member 41	SLC25A41	1,11
zinc finger protein 781	ZNF781	1,11
Memczak2013 ANTISENSE, coding, INTERNAL, intronic best transcript NM_001242765	DLGAP1	1,11
regulator of G-protein signaling 6	RGS6	1,11
guanine nucleotide binding protein-like 3 (nucleolar)-like	GNL3L	1,11
secreted phosphoprotein 1	SPP1	1,11
adaptor-related protein complex 4, mu 1 subunit	AP4M1	1,11
serpin peptidase inhibitor, clade B (ovalbumin), member 3	SERPINB3	1,11
family with sequence similarity 49, member B	FAM49B	1,11
sclerostin domain containing 1	SOSTDC1	1,11
neuropeptide Y receptor Y2	NPY2R	1,11
fidgetin-like 2	FIGNL2	1,11
neuralized E3 ubiquitin protein ligase 1	NEURL1	1,11
chromosome 12 open reading frame 43	C12orf43	1,11
ficolin (collagen/fibrinogen domain containing) 3	FCN3	1,11
PARK2 co-regulated like	PACRGL	1,11
proprotein convertase subtilisin/kexin type 2	PCSK2	1,11
Transcript Identified by AceView, Entrez Gene ID(s) 79844	ZDHHC11	1,11
prokineticin 1	PROK1	1,11
Jeck2013 ALT_ACCEPTOR, ALT_DONOR, coding, INTERNAL, intronic best transcript NM_024524	ATP13A3	1,11
POC1 centriolar protein B; POC1B-GALNT4 readthrough; polypeptide N-acetylgalactosaminyltransferase 4	POC1B; POC1B-GALNT4; GALNT4	1,11
WD repeat, sterile alpha motif and U-box domain containing 1	WDSUB1	1,11
DEAD/H (Asp-Glu-Ala-Asp/His) box polypeptide 26B	DDX26B	1,11
growth arrest and DNA-damage-inducible, gamma	GADD45G	1,11
chymotrypsin-like elastase family, member 3B	CELA3B	1,11
zinc finger protein 691	ZNF691	1,11
thrombospondin type 1 domain containing 1	THSD1	1,11
ankyrin repeat domain 18A; family with sequence similarity 95, member C	ANKRD18A; FAM95C	1,11
NADH dehydrogenase (ubiquinone) Fe-S protein 2, 49kDa (NADH-coenzyme Q reductase)	NDUFS2	1,11
gastric inhibitory polypeptide receptor	GIPR	1,11
actin filament associated protein 1-like 2	AFAP1L2	1,11

cullin 4A	CUL4A	1,11
HD domain containing 3	HDDC3	1,11
CMT1A duplicated region transcript 15-like 2	CDRT15L2	1,11
mesenchyme homeobox 2	MEOX2	1,11
ribosomal protein S6 kinase, 90kDa, polypeptide 1	RPS6KA1	1,11
GTPase activating Rap/RanGAP domain-like 3	GARNL3	1,11
serine/arginine-rich splicing factor 6	SRSF6	1,11
frataxin	FXN	1,11
protein tyrosine phosphatase, receptor type, C-associated protein	PTPRCAP	1,11
G protein regulated inducer of neurite outgrowth 1	GPRIN1	1,11
nudix hydrolase 9	NUDT9	1,11
Transcript Identified by AceView, Entrez Gene ID(s) 91445	RNF185	1,11
DEAD (Asp-Glu-Ala-Asp) box polypeptide 19B	DDX19B	1,11
MAGE family member B10	MAGEB10	1,11
pentraxin 4, long	PTX4	1,11
kinase suppressor of ras 2	KSR2	1,11
Parkinson disease 7 domain containing 1	PDDC1	1,11
uncharacterized LOC728485; novel transcript	LOC728485; CTD-2162K18.4	1,11
ATPase, Na+/K+ transporting, beta 4 polypeptide	ATP1B4	1,11
defensin, beta 121	DEFB121	1,11
dual specificity phosphatase 5	DUSP5	1,11
IQ motif containing G	IQCG	1,11
CDV3 homolog (mouse)	CDV3	1,11
zinc finger protein 557	ZNF557	1,11
arrestin domain containing 1	ARRDC1	1,11
dual specificity tyrosine-(Y)-phosphorylation regulated kinase 4	DYRK4	1,11
choline phosphotransferase 1	CHPT1	1,11
pregnancy specific beta-1-glycoprotein 8	PSG8	1,11
chorionic gonadotropin, beta polypeptide 8	CGB8	1,11
NEDD4 binding protein 2-like 2	N4BP2L2	1,11
zinc finger protein 221	ZNF221	1,11
actin-related protein 10 homolog (S. cerevisiae)	ACTR10	1,11
intercellular adhesion molecule 1	ICAM1	1,11
peroxisomal biogenesis factor 7	PEX7	1,11
N-6 adenine-specific DNA methyltransferase 2 (putative)	N6AMT2	1,11
butyrophilin-like 9	BTNL9	1,11
N-acetylated alpha-linked acidic dipeptidase-like 2	NAALADL2	1,11
NADH dehydrogenase (ubiquinone) Fe-S protein 6, 13kDa (NADH-coenzyme Q reductase)	NDUFS6	1,11
EP300 interacting inhibitor of differentiation 2B	EID2B	1,11
T-box 20	TBX20	1,11
lipase, family member J	LIPJ	1,11
mitochondrial ribosomal protein L15	MRPL15	1,11

transmembrane protein 71	TMEM71	1,11
KIAA0922	KIAA0922	1,11
ER membrane protein complex subunit 3	EMC3	1,11
peptidyl arginine deiminase, type I	PADI1	1,11
sperm acrosome associated 6	SPACA6	1,11
family with sequence similarity 209, member A	FAM209A	1,11
suppressor of cytokine signaling 6	SOCS6	1,11
zinc finger protein 816	ZNF816	1,11
neurogenin 1	NEUROG1	1,11
leukemia NUP98 fusion partner 1	LNP1	1,11
ribonuclease, RNase A family, 9 (non-active)	RNASE9	1,11
acyl-CoA synthetase medium-chain family member 6	ACSM6	1,11
ankyrin repeat domain 46	ANKRD46	1,11
transient receptor potential cation channel, subfamily M, member 5	TRPM5	1,11
G protein-coupled receptor 20	GPR20	1,11
rabaptin, RAB GTPase binding effector protein 1	RABEP1	1,11
oxysterol binding protein-like 6	OSBPL6	1,11
Transcript Identified by AceView, Entrez Gene ID(s) 4684	NCAM1	1,11
ADAM metallopeptidase domain 12	ADAM12	1,11
sorting nexin 13	SNX13	1,11
NHP2 ribonucleoprotein	NHP2	1,11
interferon induced transmembrane protein 5	IFITM5	1,11
neuronal PAS domain protein 2	NPAS2	1,11
family with sequence similarity 107, member B	FAM107B	1,11
pleckstrin 2	PLEK2	1,11
bolA family member 1	BOLA1	1,11
solute carrier family 15 (oligopeptide transporter), member 4	SLC15A4	1,11
collagen, type V, alpha 1	COL5A1	1,11
family with sequence similarity 185, member A	FAM185A	1,11
laminin, alpha 2	LAMA2	1,11
ELOVL fatty acid elongase 5	ELOVL5	1,11
cadherin-related family member 2	CDHR2	1,11
DnaJ (Hsp40) homolog, subfamily C, member 4	DNAJC4	1,11
mannosidase, beta A, lysosomal-like	MANBAL	1,11
membrane associated ring finger 6	MARCH6	1,11
zinc finger and BTB domain containing 48	ZBTB48	1,11
transmembrane protein 30B	TMEM30B	1,11
melanocortin 2 receptor accessory protein 2	MRAP2	1,11
TLX1 neighbor	TLX1NB	1,11
CD207 molecule, langerin	CD207	1,11
poly(A) polymerase gamma	PAPOLG	1,11
tetratricopeptide repeat domain 25	TTC25	1,11
Zhang2013 ALT_ACCEPTOR, ALT_DONOR, coding, INTERNAL, intronic best transcript NM_002224	ITPR3	1,11

raftlin family member 2	RFTN2	1,11
Jeck2013 ANTISENSE, coding, INTERNAL, OVEXON, UTR3 best transcript NM_020746	MAVS	1,11
LDL receptor related protein 5 like	LRP5L	1,11
single-stranded DNA binding protein 1, mitochondrial	SSBP1	1,11
family with sequence similarity 83, member H	FAM83H	1,11
transmembrane protein 68	TMEM68	1,11
potassium channel, voltage gated eag related subfamily H, member 7	KCNH7	1,11
CCCTC-binding factor (zinc finger protein)	CTCF	1,11
gap junction protein beta 2	GJB2	1,11
ATP synthase, H+ transporting, mitochondrial Fo complex subunit C2 (subunit 9)	ATP5G2	1,11
olfactory receptor, family 2, subfamily T, member 27	OR2T27	1,11
cytochrome P450, family 2, subfamily D, polypeptide 6	CYP2D6	1,11
vesicular, overexpressed in cancer, prosurvival protein 1	VOPP1	1,11
tetratricopeptide repeat domain 8	TTC8	1,11
V-set and immunoglobulin domain containing 1	VSIG1	1,11
centromere protein F	CENPF	1,11
zinc finger protein 131	ZNF131	1,11
proline rich 18	PRR18	1,11
cyclin-dependent kinase 5, regulatory subunit 1 (p35)	CDK5R1	1,11
von Hippel-Lindau tumor suppressor-like	VHLL	1,11
Bardet-Biedl syndrome 2	BBS2	1,11
protein kinase (cAMP-dependent, catalytic) inhibitor gamma	PKIG	1,11
regulatory factor X, 3 (influences HLA class II expression)	RFX3	1,11
chromosome 7 open reading frame 77	C7orf77	1,11
nucleophosmin/nucleoplasmin 3	NPM3	1,11
nuclear RNA export factor 1	NXF1	1,11
CKLF-like MARVEL transmembrane domain containing 2	CMTM2	1,11
Transcript Identified by AceView, Entrez Gene ID(s) 6579	SLCO1A2	1,11
tumor necrosis factor receptor superfamily, member 9	TNFRSF9	1,11
SCO1 cytochrome c oxidase assembly protein	SCO1	1,11
MLX interacting protein	MLXIP	1,11
sterile alpha motif domain containing 11	SAMD11	1,11
v-myb avian myeloblastosis viral oncogene homolog-like 1	MYBL1	1,11
PC4 and SFRS1 interacting protein 1	PSIP1	1,11
PHD finger protein 2	PHF2	1,11
maestro heat-like repeat family member 8	MROH8	1,11
peptidylprolyl isomerase D	PPID	1,11
NOP2/Sun domain family, member 5	NSUN5	1,11
solute carrier family 6 (neurotransmitter transporter), member 12	SLC6A12	1,11
reticulocalbin 1, EF-hand calcium binding domain	RCN1	1,11
peptidase D	PEPD	1,11

BMX non-receptor tyrosine kinase	BMX	1,11
small nuclear ribonucleoprotein polypeptide N; SNRPN upstream reading frame; small nucleolar RNA, C/D box 107; Prader Willi/Angelman region RNA, SNRPN neighbor	SNRPN; SNURF; SNORD107; PWARSN	1,11
charged multivesicular body protein 2A	CHMP2A	1,11
Rap guanine nucleotide exchange factor 2	RAPGEF2	1,11
defensin, beta 106B; defensin, beta 106A	DEFB106B; DEFB106A	1,11
Transcript Identified by AceView, Entrez Gene ID(s) 375775	PNPLA7	1,11
EMI domain containing 1	EMID1	1,11
interactor of little elongation complex ELL subunit 1	ICE1	1,11
cytoplasmic polyadenylation element binding protein 1	CPEB1	1,11
monooxygenase, DBH-like 1	MOXD1	1,11
family with sequence similarity 153, member B	FAM153B	1,11
HORMA domain containing 2	HORMAD2	1,11
dual specificity phosphatase 19	DUSP19	1,11
PRAME family member 20	PRAMEF20	1,11
odorant binding protein 2B	OBP2B	1,11
pleckstrin homology domain containing, family N member 1	PLEKHN1	1,11
sphingosine-1-phosphate phosphatase 2	SGPP2	1,11
cullin-associated and neddylation-dissociated 2 (putative)	CAND2	1,11
nucleoporin 214kDa	NUP214	1,11
COMM domain containing 6	COMMD6	1,11
C20orf166 antisense RNA 1	C20orf166-AS1	1,11
phosphodiesterase 8A	PDE8A	1,11
spinster homolog 3 (Drosophila)	SPNS3	1,11
splA/ryanodine receptor domain and SOCS box containing 2	SPSB2	1,11
Memczak2013 ALT_ACCEPTOR, ALT_DONOR, coding, INTERNAL, intronic best transcript NM_003664	AP3B1	1,11
C2CD2-like	C2CD2L	1,11
CD33 molecule	CD33	1,11
spermidine/spermine N1-acetyltransferase family member 2	SAT2	1,11
slingshot protein phosphatase 2	SSH2	1,11
dehydrogenase/reductase (SDR family) member 4 like 2	DHRS4L2	1,11
tetratricopeptide repeat domain 27; microRNA 4765	TTC27; MIR4765	1,11
hes family bHLH transcription factor 1	HES1	1,11
JNK1/MAPK8-associated membrane protein	JKAMP	1,11
Memczak2013 ALT_ACCEPTOR, ALT_DONOR, coding, INTERNAL, intronic best transcript NM_018482	ASAP1	1,11
tankyrase 1 binding protein 1	TNKS1BP1	1,11
NPR3-like, GATOR1 complex subunit	NPRL3	1,11
regulator of G protein signaling 9 binding protein	RGS9BP	1,11

Dab, mitogen-responsive phosphoprotein, homolog 2 (Drosophila)	DAB2	1,11
homeobox D13	HOXD13	1,11
ATP binding cassette subfamily A member 1	ABCA1	1,11
synaptotagmin IV	SYT4	1,11
Smith-Magenis syndrome chromosome region, candidate 5 (non-protein coding)	SMCR5	1,11
SVOP-like	SVOPL	1,11
NACHT and WD repeat domain containing 1	NWD1	1,11
ankyrin repeat and SOCS box containing 17	ASB17	1,11
taste receptor, type 2, member 40	TAS2R40	1,11
methyltransferase like 7B	METTL7B	1,11
serine/arginine-rich splicing factor 7	SRSF7	1,11
calcium/calmodulin-dependent protein kinase kinase 1, alpha	CAMKK1	1,11
zinc finger protein 131	ZNF131	1,11
PHD finger protein 20	PHF20	1,11
solute carrier family 16, member 11	SLC16A11	1,11
discs, large (Drosophila) homolog-associated protein 3	DLGAP3	1,11
IMP3, U3 small nucleolar ribonucleoprotein	IMP3	1,11
cramped chromatin regulator homolog 1	CRAMP1	1,11
coiled-coil domain containing 188	CCDC188	1,11
transmembrane protein 207	TMEM207	1,11
cholinergic receptor, nicotinic beta 4	CHRNB4	1,11
solute carrier organic anion transporter family, member 2A1	SLCO2A1	1,11
olfactory receptor, family 52, subfamily B, member 6	OR52B6	1,11
ADAM metallopeptidase domain 19	ADAM19	1,11
CD79b molecule, immunoglobulin-associated beta	CD79B	1,11
long intergenic non-protein coding RNA 1225	LINC01225	1,11
ArfGAP with SH3 domain, ankyrin repeat and PH domain 3	ASAP3	1,11
apolipoprotein B mRNA editing enzyme, catalytic polypeptide-like 3H	APOBEC3H	1,11
Rh blood group, D antigen	RHD	1,11
structural maintenance of chromosomes 1A	SMC1A	1,11
FK506 binding protein 3	FKBP3	1,11
B melanoma antigen; B melanoma antigen family, member 4; B melanoma antigen family, member 3; B melanoma antigen family, member 2; B melanoma antigen family, member 5	BAGE; BAGE4; BAGE3; BAGE2; BAGE5	1,11
Ras association (RalGDS/AF-6) domain family member 6	RASSF6	1,11
GRB2-associated binding protein 3	GAB3	1,11
UPK1A antisense RNA 1	UPK1A-AS1	1,11
osteoclast stimulatory transmembrane protein	OCSTAMP	1,11
nischarin	NISCH	1,11
centrosomal protein 350kDa	CEP350	1,11

family with sequence similarity 25, member G; family with sequence similarity 25, member C; protein FAM25	FAM25G; FAM25C; FAM25BP	1,11
cordon-bleu WH2 repeat protein	COBL	1,11
solute carrier family 7 (cationic amino acid transporter, y+ system), member 3	SLC7A3	1,11
zinc finger protein 764	ZNF764	1,11
adipogenesis associated, Mth938 domain containing	AAMDC	1,11
melanoma cell adhesion molecule; microRNA 6756	MCAM; MIR6756	1,11
lethal giant larvae homolog 2 (Drosophila)	LLGL2	1,11
glycosyltransferase-like 1B	GYLTL1B	1,11
dihydroorotate dehydrogenase (quinone)	DHODH	1,11
beta-1,3-N-acetylgalactosaminyltransferase 2	B3GALNT2	1,11
kinesin family member 24	KIF24	1,11
topoisomerase (DNA) I	TOP1	1,11
fibronectin type III domain containing 3B	FNDC3B	1,11
2-deoxynucleoside 5-phosphate N-hydrolase 1	DNPH1	1,11
lysine-rich nucleolar protein 1	KNOP1	1,11
membrane bound O-acyltransferase domain containing 4	MBOAT4	1,11
bobby sox homolog (Drosophila)	BBX	1,11
Memczak2013 ALT_ACCEPTOR, ALT_DONOR, coding, INTERNAL, intronic best transcript NM_014502	PRPF19	1,11
neuroblastoma amplified sequence	NBAS	1,11
chromatin accessibility complex 1	CHRAC1	1,11
ALG5, dolichyl-phosphate beta-glucosyltransferase	ALG5	1,11
family with sequence similarity 46, member B	FAM46B	1,11
stathmin-like 3	STMN3	1,11
S100 calcium binding protein A9	S100A9	1,11
structural maintenance of chromosomes 6	SMC6	1,11
SECIS binding protein 2	SECISBP2	1,11
chemokine (C-C motif) receptor 5 (gene/pseudogene)	CCR5	1,11
hyaluronan synthase 3	HAS3	1,11
purinergic receptor P2Y, G-protein coupled, 13	P2RY13	1,11
zinc finger protein 317	ZNF317	1,11
LY6/PLAUR domain containing 8	LYPD8	1,11
periphilin 1	PPHLN1	1,11
MLX interacting protein	MLXIP	1,11
solute carrier family 10, member 4	SLC10A4	1,11
phosphatidylinositol-4-phosphate 5-kinase, type I, beta	PIP5K1B	1,11
defensin, beta 104A; defensin, beta 104B	DEFB104A; DEFB104B	1,11
keratin 39, type I	KRT39	1,11
serpin peptidase inhibitor, clade A (alpha-1 antiproteinase, antitrypsin), member 3	SERPINA3	1,11

syntaxin binding protein 1	STXBP1	1,11
golgi membrane protein 1	GOLM1	1,11
lon peptidase 2, peroxisomal	LONP2	1,11
estrogen-related receptor alpha	ESRRA	1,11
Jeck2013 ALT_ACCEPTOR, ALT_DONOR, coding, INTERNAL, intronic best transcript NM_030796	VOPP1	1,11
SWI5 homologous recombination repair protein	SWI5	1,11
calpain 2, (m/II) large subunit	CAPN2	1,11
chromosome 6 open reading frame 58	C6orf58	1,11
zinc finger protein 780A	ZNF780A	1,11
myosin, heavy chain 7B, cardiac muscle, beta	MYH7B	1,11
calcium and integrin binding family member 3	CIB3	1,11
NIMA-related kinase 4	NEK4	1,11
zinc finger, FYVE domain containing 21	ZFYVE21	1,11
docking protein 4	DOK4	1,11
ATP binding cassette subfamily B member 5	ABCB5	1,11
ATPase, Na+/K+ transporting, beta 1 polypeptide	ATP1B1	1,11
Memczak2013 ALT_ACCEPTOR, ALT_DONOR, coding, INTERNAL, intronic best transcript NM_001008895	CUL4A	1,11
iroquois homeobox 5	IRX5	1,11
transcription factor 20 (AR1)	TCF20	1,11
transcription factor 20 (AR1)	TCF20	1,11
protein-L-isoaspartate (D-aspartate) O-methyltransferase domain containing 1	PCMTD1	1,11
chromosome 15 open reading frame 65	C15orf65	1,11
engulfment and cell motility 1	ELMO1	1,11
isocitrate dehydrogenase 3 (NAD+) beta	IDH3B	1,11
olfactory receptor, family 4, subfamily P, member 4	OR4P4	1,11
calcium/calmodulin-dependent protein kinase II delta	CAMK2D	1,11
Dmx-like 1	DMXL1	1,11
protein tyrosine phosphatase, receptor type, O	PTPRO	1,11
chromosome 5 open reading frame 45	C5orf45	1,11
flavin containing monooxygenase 6 pseudogene	FMO6P	1,11
inhibin beta A	INHBA	1,11
thioredoxin	TXN	1,11
mitochondrial pyruvate carrier 2	MPC2	1,11
heat shock transcription factor 2 binding protein	HSF2BP	1,11
family with sequence similarity 103, member A1	FAM103A1	1,11
tubulin, alpha 1c	TUBA1C	1,11
solute carrier family 25, member 38	SLC25A38	1,11
sterile alpha motif domain containing 4B	SAMD4B	1,11
chromosome 11 open reading frame 84	C11orf84	1,11
golgin A8 family, member G	GOLGA8G	1,11
butyrophilin-like 3	BTNL3	1,11
proteasome 26S subunit, non-ATPase 7	PSMD7	1,11

Transcript Identified by AceView, Entrez Gene ID(s) 11059	WWP1	1,11
coiled-coil domain containing 178	CCDC178	1,11
BCL2-associated athanogene 2	BAG2	1,11
DEAD (Asp-Glu-Ala-Asp) box polypeptide 10	DDX10	1,10
forkhead box B1	FOXB1	1,10
Scm-like with four mbt domains 1	SFMBT1	1,10
MCF.2 cell line derived transforming sequence like	MCF2L	1,10
hydroxymethylbilane synthase	HMBS	1,10
ras homolog family member G	RHOG	1,10
K(lysine) acetyltransferase 6B	KAT6B	1,10
myosin binding protein C, fast type	MYBPC2	1,10
Memczak2013 ALT_ACCEPTOR, coding, INTERNAL, intronic best transcript NM_001080443	KIF18B	1,10
BEN domain containing 2	BEND2	1,10
ADAMTS like 2	ADAMTSL2	1,10
eukaryotic translation initiation factor 4E family member 2	EIF4E2	1,10
phosphoinositide-3-kinase adaptor protein 1	PIK3AP1	1,10
coiled-coil domain containing 157	CCDC157	1,10
Kv channel interacting protein 2	KCNIP2	1,10
fascin actin-bundling protein 3, testicular	FSCN3	1,10
regulatory factor X, 2 (influences HLA class II expression)	RFX2	1,10
E2F transcription factor 4, p107/p130-binding	E2F4	1,10
ArfGAP with GTPase domain, ankyrin repeat and PH domain 4	AGAP4	1,10
diacylglycerol kinase, delta 130kDa	DGKD	1,10
pre-mRNA processing factor 3	PRPF3	1,10
SPC24, NDC80 kinetochore complex component	SPC24	1,10
zinc finger, ZZ-type containing 3	ZZZ3	1,10
active BCR-related	ABR	1,10
chromosome 10 open reading frame 54	C10orf54	1,10
potassium channel, voltage gated Shab related subfamily B, member 2	KCNB2	1,10
ring finger protein 149	RNF149	1,10
adrenoceptor beta 3	ADRB3	1,10
family with sequence similarity 50, member A	FAM50A	1,10
phosphodiesterase 1A, calmodulin-dependent	PDE1A	1,10
protein kinase, cAMP-dependent, regulatory, type I, beta	PRKAR1B	1,10
collagen, type IV, alpha 3 (Goodpasture antigen)	COL4A3	1,10
mediator complex subunit 4	MED4	1,10
leukocyte-associated immunoglobulin-like receptor 1	LAIR1	1,10
ataxin 7	ATXN7	1,10
RWD domain containing 2A	RWDD2A	1,10
v-myc avian myelocytomatosis viral oncogene neuroblastoma derived homolog	MYCN	1,10
CCAAT/enhancer binding protein (C/EBP), zeta	CEBPZ	1,10
chemokine (C-C motif) receptor 1	CCR1	1,10

NADH dehydrogenase (ubiquinone) 1 beta subcomplex, 7, 18kDa	NDUFB7	1,10
chromosome 18 open reading frame 63	C18orf63	1,10
STEAP family member 1B	STEAP1B	1,10
exostosin-like glycosyltransferase 2	EXTL2	1,10
target of myb1 like 1 membrane trafficking protein	TOM1L1	1,10
TatD DNase domain containing 1; microRNA 6844	TATDN1; MIR6844	1,10
chromosome 19 open reading frame 57	C19orf57	1,10
ferredoxin reductase	FDXR	1,10
Yip1 domain family member 1	YIPF1	1,10
bromodomain and WD repeat domain containing 1	BRWD1	1,10
collagen alpha-1(II) chain-like; novel transcript	LOC101928841; RP11-102K13.5	1,10
RRN3 homolog, RNA polymerase I transcription factor	RRN3	1,10
zinc finger protein 680	ZNF680	1,10
SPATA31 subfamily A, member 6	SPATA31A6	1,10
myelin oligodendrocyte glycoprotein	MOG	1,10
RELT tumor necrosis factor receptor	RELT	1,10
gap junction protein alpha 8	GJA8	1,10
uncharacterized LOC100128554; Transcript Identified by AceView, Entrez Gene ID(s) 144678; 100128554; novel transcript	LOC100128554; RP5-944M2.3	1,10
keratin associated protein 5-1	KRTAP5-1	1,10
melanoregulin	MREG	1,10
LEM domain containing 2	LEMD2	1,10
tubulin folding cofactor C	TBCC	1,10
peptidyl arginine deiminase, type VI	PADI6	1,10
P antigen family, member 3 (prostate associated)	PAGE3	1,10
GRB2-binding adaptor protein, transmembrane	GAPT	1,10
RAB3A interacting protein	RAB3IP	1,10
coiled-coil domain containing 171	CCDC171	1,10
protein disulfide isomerase family A, member 4	PDIA4	1,10
GINS complex subunit 3 (Psf3 homolog)	GINS3	1,10
ATPase, Ca++ transporting, type 2C, member 2	ATP2C2	1,10
signal peptide, CUB domain, EGF-like 2	SCUBE2	1,10
ankyrin repeat domain 35	ANKRD35	1,10
thyroid hormone receptor interactor 13	TRIP13	1,10
calcium/calmodulin-dependent protein kinase kinase 2, beta	CAMKK2	1,10
eukaryotic translation initiation factor 2B, subunit 5 epsilon, 82kDa	EIF2B5	1,10
ArfGAP with GTPase domain, ankyrin repeat and PH domain 6	AGAP6	1,10
Myb-like, SWIRM and MPN domains 1	MYSM1	1,10
prominin 2	PROM2	1,10
lipoma HMGIC fusion partner-like 1	LHFPL1	1,10

G antigen 12I; G antigen 12F	GAGE12I; GAGE12F	1,10
sorting nexin 29	SNX29	1,10
vacuolar protein sorting 33 homolog A (S. cerevisiae)	VPS33A	1,10
Memczak2013 ALT_ACCEPTOR, ALT_DONOR, coding, INTERNAL, intronic best transcript NM_014713	LAPTM4A	1,10
transmembrane protein 255B	TMEM255B	1,10
coiled-coil domain containing 14	CCDC14	1,10
chromodomain protein, Y-linked, 1B; chromodomain protein, Y-linked, 1	CDY1B; CDY1	1,10
DEAD (Asp-Glu-Ala-Asp) box polypeptide 20	DDX20	1,10
zinc finger protein 18	ZNF18	1,10
phosphoseryl-tRNA kinase	PSTK	1,10
synaptotagmin VI	SYT6	1,10
telomerase-associated protein 1	TEP1	1,10
NADH dehydrogenase (ubiquinone) 1 alpha subcomplex, 11, 14.7kDa	NDUFA11	1,10
syntaxin 6	STX6	1,10
homeobox A6	HOXA6	1,10
cell growth regulator with ring finger domain 1	CGRRF1	1,10
transmembrane protein, adipocyte asscociated 1	TPRA1	1,10
family with sequence similarity 231, member D; long intergenic non-protein coding RNA 869	FAM231D; LINC00869	1,10
translocase of inner mitochondrial membrane 13 homolog (yeast)	TIMM13	1,10
5-hydroxytryptamine (serotonin) receptor 3A, ionotropic	HTR3A	1,10
RNA binding motif protein 26	RBM26	1,10
zinc finger protein 382	ZNF382	1,10
ribosomal protein S6 kinase, 90kDa, polypeptide 1	RPS6KA1	1,10
jun proto-oncogene	JUN	1,10
synaptotagmin-like 3	SYTL3	1,10
glycosyltransferase 1 domain containing 1	GLT1D1	1,10
IQ motif containing F2	IQCF2	1,10
LDL receptor related protein 6	LRP6	1,10
PRAME family member 7	PRAMEF7	1,10
mannosidase, alpha, class 1C, member 1	MAN1C1	1,10
N-ethylmaleimide-sensitive factor	NSF	1,10
chromosome 10 open reading frame 105	C10orf105	1,10
ATP binding cassette subfamily G member 4	ABCG4	1,10
G protein-coupled receptor 152	GPR152	1,10
kinesin family member 21B	KIF21B	1,10
FK506 binding protein 6	FKBP6	1,10
solute carrier organic anion transporter family, member 4A1	SLCO4A1	1,10
Mdm1 nuclear protein	MDM1	1,10
diacylglycerol kinase, delta 130kDa	DGKD	1,10

chromosome 17 open reading frame 97	C17orf97	1,10
claudin 8	CLDN8	1,10
maestro heat-like repeat family member 2A	MROH2A	1,10
BPI fold containing family A, member 3	BPIFA3	1,10
cytohesin 1 interacting protein	CYTIP	1,10
piggyBac transposable element derived 2	PGBD2	1,10
histone cluster 3, H2bb	HIST3H2BB	1,10
glycine amidinotransferase (L-arginine:glycine amidinotransferase)	GATM	1,10
NOP2/Sun domain family, member 3	NSUN3	1,10
polymerase (DNA-directed), delta interacting protein 2	POLDIP2	1,10
multiple C2 domains, transmembrane 1	MCTP1	1,10
syntaxin 3	STX3	1,10
neuropeptide Y receptor Y4	NPY4R	1,10
glucokinase (hexokinase 4) regulator	GCKR	1,10
Transcript Identified by AceView, Entrez Gene ID(s) 65249	ZSWIM4	1,10
receptor (G protein-coupled) activity modifying protein 2	RAMP2	1,10
apolipoprotein C-II	APOC2	1,10
chromosome 9 open reading frame 50	C9orf50	1,10
nucleobindin 2	NUCB2	1,10
myosin VC	MYO5C	1,10
amylo-alpha-1, 6-glucosidase, 4-alpha-glucanotransferase	AGL	1,10
mitochondrial ribosomal protein L9	MRPL9	1,10
G protein-coupled receptor 174	GPR174	1,10
netrin 1	NTN1	1,10
integrator complex subunit 5	INTS5	1,10
ATPase, class VI, type 11A	ATP11A	1,10
paraneoplastic Ma antigen 3	PNMA3	1,10
solute carrier family 6 (proline IMINO transporter), member 20	SLC6A20	1,10
transmembrane protein 179B; microRNA 6748	TMEM179B; MIR6748	1,10
inhibitor of DNA binding 2, dominant negative helix-loop-helix protein	ID2	1,10
transmembrane protein 44	TMEM44	1,10
general transcription factor IIB	GTF2B	1,10
actin, alpha 2, smooth muscle, aorta	ACTA2	1,10
AHA1, activator of heat shock 90kDa protein ATPase homolog 2 (yeast)	AHSA2	1,10
TBC/LysM-associated domain containing 2	TLDC2	1,10
visual system homeobox 1	VSX1	1,10
splicing factor, suppressor of white-apricot family	SFSWAP	1,10
DEAH (Asp-Glu-Ala-His) box polypeptide 38	DHX38	1,10
cell adhesion molecule 1	CADM1	1,10
interferon gamma receptor 1	IFNGR1	1,10
ubiquitin-like 4A	UBL4A	1,10

cryptochrome circadian clock 2	CRY2	1,10
kelch-like family member 4	KLHL4	1,10
golgin A2 pseudogene 5	GOLGA2P5	1,10
RNA binding motif protein 24	RBM24	1,10
SAM domain and HD domain 1	SAMHD1	1,10
zinc finger protein 92	ZNF92	1,10
pregnancy specific beta-1-glycoprotein 4	PSG4	1,10
tumor suppressing subtransferable candidate 4	TSSC4	1,10
spalt-like transcription factor 4	SALL4	1,10
ganglioside induced differentiation associated protein 1	GDAP1	1,10
ubiquitin specific peptidase 17-like family member 2	USP17L2	1,10
transmembrane protein 95	TMEM95	1,10
syntaxin 10	STX10	1,10
guanylate kinase 1	GUK1	1,10
hydroxymethylbilane synthase	HMBS	1,10
uncharacterized LOC79160; novel transcript	LOC79160; RP5-907D15.4	1,10
Ral GTPase activating protein, beta subunit (non-catalytic)	RALGAPB	1,10
transmembrane protein 129, E3 ubiquitin protein ligase	TMEM129	1,10
major histocompatibility complex, class II, DR beta 5	HLA-DRB5	1,10
integrin beta 4	ITGB4	1,10
nth-like DNA glycosylase 1	NTHL1	1,10
ribosomal protein S25	RPS25	1,10
intraflagellar transport 52	IFT52	1,10
TAF11 RNA polymerase II, TATA box binding protein (TBP)-associated factor, 28kDa	TAF11	1,10
family with sequence similarity 49, member A	FAM49A	1,10
class II, major histocompatibility complex, transactivator	CIITA	1,10
zinc finger and BTB domain containing 37	ZBTB37	1,10
putative uncharacterized protein FLJ37770-like; Transcript Identified by AceView, Entrez Gene ID(s) 387790; novel transcript	LOC100506127; RP11-111M22.2; LOC387790	1,10
mucin 19, oligomeric	MUC19	1,10
dual specificity phosphatase 2	DUSP2	1,10
PHD finger protein 23	PHF23	1,10
myosin light chain 12A	MYL12A	1,10
fibrinogen gamma chain	FGG	1,10
NK3 homeobox 2	NKX3-2	1,10
olfactory receptor, family 6, subfamily C, member 3	OR6C3	1,10
testis expressed 33	TEX33	1,10
exocyst complex component 3	EXOC3	1,10
hypoxia up-regulated 1	HYOU1	1,10
AFG3-like AAA ATPase 2	AFG3L2	1,10
zinc finger protein 407	ZNF407	1,10

eukaryotic translation initiation factor 1	EIF1	1,10
small integral membrane protein 10 like 2A	SMIM10L2A	1,10
spinster homolog 2 (Drosophila)	SPNS2	1,10
myosin phosphatase Rho interacting protein	MPRIP	1,10
high mobility group AT-hook 1	HMGA1	1,10
troponin T type 1 (skeletal, slow)	TNNT1	1,10
formin 1	FMN1	1,10
transmembrane phosphoinositide 3-phosphatase and tensin homolog 2	TPTE2	1,10
proline-rich protein BstNI subfamily 2	PRB2	1,10
widely interspaced zinc finger motifs	WIZ	1,10
interleukin 11	IL11	1,10
RWD domain containing 1	RWDD1	1,10
serine peptidase inhibitor, Kazal type 5	SPINK5	1,10
RAB8B, member RAS oncogene family	RAB8B	1,10
complement component 8, alpha polypeptide	C8A	1,10
golgin A6 family-like 22	GOLGA6L22	1,10
RAB1B, member RAS oncogene family	RAB1B	1,10
mitochondrial ribosomal protein S25	MRPS25	1,10
leiomodin 2 (cardiac)	LMOD2	1,10
phosphoinositide-3-kinase, regulatory subunit 6	PIK3R6	1,10
CWC27 spliceosome-associated protein homolog	CWC27	1,10
SPT20 homolog, SAGA complex component-like 1	SUPT20HL1	1,10
family with sequence similarity 214, member A	FAM214A	1,10
solute carrier family 4, sodium bicarbonate cotransporter, member 7	SLC4A7	1,10
HGH1 homolog	HGH1	1,10
chromosome 20 open reading frame 197	C20orf197	1,10
chorionic somatomammotropin hormone-like 1	CSHL1	1,10
retinoic acid induced 2	RAI2	1,10
RNA pseudouridylate synthase domain containing 2	RPUSD2	1,10
HtrA serine peptidase 1	HTRA1	1,10
chromosome 12 open reading frame 65	C12orf65	1,10
long intergenic non-protein coding RNA 1588	LINC01588	1,10
Ras-like without CAAX 2	RIT2	1,10
mevalonate kinase	MVK	1,10
tripartite motif containing 9	TRIM9	1,10
acyl-CoA thioesterase 6	ACOT6	1,10
aspartate beta-hydroxylase domain containing 1	ASPHD1	1,10
ring finger protein 222	RNF222	1,10
thyroid adenoma associated	THADA	1,10
dedicator of cytokinesis 3	DOCK3	1,10
NDUFA4, mitochondrial complex associated	NDUFA4	1,10
claudin 9	CLDN9	1,10
ryanodine receptor 2 (cardiac)	RYR2	1,10

adenosylhomocysteinase	AHCY	1,10
empty spiracles homeobox 2	EMX2	1,10
coiled-coil domain containing 160	CCDC160	1,10
histone deacetylase 2	HDAC2	1,10
autophagy related 16-like 1	ATG16L1	1,10
DIRAS family, GTP-binding RAS-like 3	DIRAS3	1,10
neuronal differentiation 4	NEUROD4	1,10
coiled-coil domain containing 84	CCDC84	1,10
DTW domain containing 1	DTWD1	1,10
interleukin 22 receptor, alpha 2	IL22RA2	1,10
cytochrome P450, family 2, subfamily C, polypeptide 8	CYP2C8	1,10
fatty acid desaturase 3	FADS3	1,10
churchill domain containing 1	CHURC1	1,10
interferon-induced protein with tetratricopeptide repeats 1B	IFIT1B	1,10
nuclear receptor subfamily 1, group H, member 2	NR1H2	1,10
zygote arrest 1	ZAR1	1,10
even-skipped homeobox 2	EVX2	1,10
forkhead box B1	FOXB1	1,10
Transcript Identified by AceView, Entrez Gene ID(s) 91782	CHMP7	1,10
rabphilin 3A-like (without C2 domains)	RPH3AL	1,10
transmembrane protein 156	TMEM156	1,10
potassium channel, calcium activated large conductance subfamily M alpha, member 1	KCNMA1	1,10
T-cell leukemia homeobox 3	TLX3	1,10
dual specificity phosphatase 22	DUSP22	1,10
olfactory receptor, family 10, subfamily A, member 7	OR10A7	1,10
PH domain and leucine rich repeat protein phosphatase 1	PHLPP1	1,10
polymerase (RNA) II (DNA directed) polypeptide F; microRNA 4534; microRNA 6820	POLR2F; MIR4534; MIR6820	1,10
family with sequence similarity 178, member B	FAM178B	1,10
synaptogyrin 1	SYNGR1	1,10
zinc finger protein 671	ZNF671	1,10
TCF3 (E2A) fusion partner (in childhood Leukemia)	TFPT	1,10
biogenesis of lysosomal organelles complex-1, subunit 1	BLOC1S1	1,10
chaperonin containing TCP1, subunit 8 (theta)-like 2	CCT8L2	1,10
hypoxia up-regulated 1	HYOU1	1,10
oxysterol binding protein-like 9	OSBPL9	1,10
ataxin 7-like 2	ATXN7L2	1,10
serum amyloid A-like 1	SAAL1	1,10
spermatogenesis associated, serine-rich 2	SPATS2	1,10
Transcript Identified by AceView, Entrez Gene ID(s) 1791	DNTT	1,10
dihydropyrimidine dehydrogenase	DPYD	1,10
zinc ribbon domain containing 1	ZNRD1	1,10
solute carrier family 35, member E4	SLC35E4	1,10

acid phosphatase 6, lysophosphatidic	ACP6	1,10
transmembrane protein 177	TMEM177	1,10
carboxypeptidase Q	CPQ	1,10
cripto, FRL-1, cryptic family 1	CFC1	1,10
extracellular leucine-rich repeat and fibronectin type III domain containing 2	ELFN2	1,10
cell division cycle 73	CDC73	1,10
zinc finger protein 26	ZNF26	1,10
ATP synthase, H+ transporting, mitochondrial F1 complex, epsilon subunit pseudogene 2	ATP5EP2	1,10
ZFP37 zinc finger protein	ZFP37	1,10
Sin3A associated protein 30kDa	SAP30	1,10
LanC lantibiotic synthetase component C-like 2 (bacterial)	LANCL2	1,10
leucine zipper-EF-hand containing transmembrane protein 2	LETM2	1,10
carcinoembryonic antigen-related cell adhesion molecule 20	CEACAM20	1,10
mitochondrial ribosomal protein L48	MRPL48	1,10
nuclear factor, erythroid 4	NFE4	1,10
unc-5 netrin receptor A	UNC5A	1,10
POTE ankyrin domain family, member G; POTE ankyrin domain family, member M	POTEG; POTEM	1,10
transglutaminase 4	TGM4	1,10
hypoxanthine phosphoribosyltransferase 1	HPRT1	1,10
leucine rich repeat protein 1; ras homolog family member Q pseudogene 1	LRR1; RHOQP1	1,10
peptidylprolyl isomerase (cyclophilin)-like 2	PPIL2	1,10
MAGE family member A8	MAGEA8	1,10
keratin 33B, type I	KRT33B	1,10
tubulin folding cofactor D	TBCD	1,10
leucine rich repeat containing 18	LRRC18	1,10
olfactory receptor, family 2, subfamily AT, member 4	OR2AT4	1,10
decorin	DCN	1,10
UDP glycosyltransferase 8	UGT8	1,10
cadherin 16, KSP-cadherin	CDH16	1,10
RUN domain and cysteine-rich domain containing, Beclin 1-interacting protein; microRNA 922	RUBCN; MIR922	1,10
TAF13 RNA polymerase II, TATA box binding protein (TBP)-associated factor, 18kDa	TAF13	1,10
branched chain keto acid dehydrogenase E1, beta polypeptide	BCKDHB	1,10
transmembrane protein 252	TMEM252	1,10
ubiquitin domain containing 1	UBTD1	1,10
synapsin II	SYN2	1,10
fukutin related protein	FKRP	1,10
translocase of inner mitochondrial membrane 23 homolog B (yeast)	TIMM23B	1,10
ADAM metallopeptidase domain 18	ADAM18	1,10

vav 2 guanine nucleotide exchange factor	VAV2	1,10
myosin VIIB	MYO7B	1,10
germinal center-associated, signaling and motility	GCSAM	1,10
kinesin family member 19	KIF19	1,10
phosphatidylcholine transfer protein	PCTP	1,10
uncharacterized LOC105377348; Salzman2013 ANTISENSE, coding, INTERNAL, intronic, OVERLAPTX best transcript NM_181890; novel transcript, antisense UBE2D3; Transcript Identified by AceView	LOC105377348; RP11-10L12.4; UBE2D3; pugo	1,10
FRY microtubule binding protein	FRY	1,10
surfactant protein B	SFTPB	1,10
tubulin, beta 3 class III; melanocortin 1 receptor (alpha melanocyte stimulating hormone receptor)	TUBB3; MC1R	1,10
heat shock 70kD protein 12B	HSPA12B	1,10
leukocyte-associated immunoglobulin-like receptor 2	LAIR2	1,10
fatty acid amide hydrolase 2	FAAH2	1,10
GPRIN family member 3	GPRIN3	1,10
ankyrin repeat and LEM domain containing 2	ANKLE2	1,10
zinc finger, SWIM-type containing 4	ZSWIM4	1,10
transmembrane protein 170B	TMEM170B	1,10
Transcript Identified by AceView, Entrez Gene ID(s) 11273	ATXN2L	1,10
developmental pluripotency associated 5	DPPA5	1,10
perilipin 2	PLIN2	1,10
ATP binding cassette subfamily B member 1	ABCB1	1,10
chromosome 1 open reading frame 185	C1orf185	1,10
butyrophilin, subfamily 2, member A1	BTN2A1	1,10
dihydrodiol dehydrogenase (dimeric)	DHDH	1,10
zinc finger with KRAB and SCAN domains 7	ZKSCAN7	1,10
v-myc avian myelocytomatosis viral oncogene lung carcinoma derived homolog	MYCL	1,10
ADAM metallopeptidase domain 21	ADAM21	1,10
interferon, alpha 10	IFNA10	1,10
long intergenic non-protein coding RNA 173	LINC00173	1,10
SH3KBP1 binding protein 1	SHKBP1	1,10
growth differentiation factor 5	GDF5	1,10
Memczak2013 ALT_ACCEPTOR, ALT_DONOR, coding, INTERNAL, intronic best transcript NM_002806	PSMC6	1,10
oxidative stress responsive serine-rich 1	OSER1	1,10
bromodomain and PHD finger containing 3	BRPF3	1,10
clarin 3	CLRN3	1,10
deoxyribonuclease I-like 1	DNASE1L1	1,10
chloride channel, voltage-sensitive 5	CLCN5	1,10
CD164 sialomucin-like 2	CD164L2	1,10
tRNA nucleotidyl transferase, CCA-adding, 1	TRNT1	1,10
mediator complex subunit 12 like	MED12L	1,10

mitogen-activated protein kinase 10	MAPK10	1,10
intelectin 1 (galactofuranose binding)	ITLN1	1,10
iroquois homeobox 2	IRX2	1,10
thioredoxin-like 4B	TXNL4B	1,10
zinc finger protein 844	ZNF844	1,10
progastricsin (pepsinogen C)	PGC	1,10
carcinoembryonic antigen-related cell adhesion molecule 6 (non-specific cross reacting antigen)	CEACAM6	1,10
Transcript Identified by AceView, Entrez Gene ID(s) 22849	CPEB3	1,10
D4, zinc and double PHD fingers family 2	DPF2	1,10
twist family bHLH transcription factor 1	TWIST1	1,10
patched domain containing 4	PTCHD4	1,10
mitotic spindle organizing protein 2B	MZT2B	1,10
hepatocyte nuclear factor 4, gamma	HNF4G	1,10
arrestin domain containing 2	ARRDC2	1,10
mitochondrial calcium uniporter regulator 1	MCUR1	1,10
PRAME family member 25	PRAMEF25	1,10
Transcript Identified by AceView, Entrez Gene ID(s) 642812	API5L1	1,10
FRAS1 related extracellular matrix 3	FREM3	1,10
long intergenic non-protein coding RNA 303	LINC00303	1,10
cadherin, EGF LAG seven-pass G-type receptor 1	CELSR1	1,10
kallikrein related peptidase 2	KLK2	1,10
triggering receptor expressed on myeloid cells 2	TREM2	1,10
inositol polyphosphate-5-phosphatase D	INPP5D	1,10
Transcript Identified by AceView, Entrez Gene ID(s) 4134	MAP4	1,10
von Willebrand factor	VWF	1,10
enkurin, TRPC channel interacting protein	ENKUR	1,10
receptor accessory protein 6	REEP6	1,10
zinc finger protein 75a	ZNF75A	1,10
transmembrane protein 147	TMEM147	1,10
nuclear RNA export factor 2B; t-complex 11 family, X-linked 2	NXF2B; TCP11X2	1,10
zinc finger protein 835	ZNF835	1,10
olfactory receptor, family 6, subfamily Q, member 1 (gene/pseudogene)	OR6Q1	1,10
chibby homolog 3 (Drosophila)	CBY3	1,10
zinc finger protein 444	ZNF444	1,10
major histocompatibility complex, class II, DQ beta 1	HLA-DQB1	1,10
long intergenic non-protein coding RNA 311	LINC00311	1,10
vitelline membrane outer layer 1 homolog (chicken)	VMO1	1,10
membrane associated ring finger 3	MARCH3	1,10
chromosome 3 open reading frame 33	C3orf33	1,10
growth arrest-specific 6	GAS6	1,10
tumor protein p53 inducible protein 3	TP53I3	1,10
leucine rich repeat containing 75B	LRRC75B	1,10

late cornified envelope 3A	LCE3A	1,10
high mobility group nucleosomal binding domain 3	HMGN3	1,10
Transcript Identified by AceView, Entrez Gene ID(s) 1997; 100128628	ELF1	1,10
C1QTNF9B antisense RNA 1	C1QTNF9B-AS1	1,10
guanylate cyclase 2C	GUCY2C	1,10
NIPA-like domain containing 1	NIPAL1	1,10
COX17 cytochrome c oxidase copper chaperone	COX17	1,10
chromatin assembly factor 1, subunit A (p150)	CHAF1A	1,10
La ribonucleoprotein domain family, member 7	LARP7	1,10
MAGE family member A3	MAGEA3	1,10
putative UPF0607 protein ENSP00000383783	LOC100131107	1,10
MAX dimerization protein 4; microRNA 4800	MXD4; MIR4800	1,10
WSC domain containing 1	WSCD1	1,10
Tctex1 domain containing 2	TCTEX1D2	1,10
proprotein convertase subtilisin/kexin type 1 inhibitor	PCSK1N	1,10
phosphatidylinositol-specific phospholipase C, X domain containing 3	PLCXD3	1,10
solute carrier family 25, member 45	SLC25A45	1,10
chorionic gonadotropin, beta polypeptide 7	CGB7	1,10
coiled-coil domain containing 71-like	CCDC71L	1,10
myosin phosphatase Rho interacting protein	MPRIP	1,10
RUN and FYVE domain containing 4	RUFY4	1,10
zinc finger protein 419	ZNF419	1,10
A kinase (PRKA) anchor protein 6	AKAP6	1,10
parathyroid hormone 2	PTH2	1,10
guanylate cyclase activator 1A (retina)	GUCA1A	1,10
sel-1 suppressor of lin-12-like 3 (C. elegans)	SEL1L3	1,10
MAP7 domain containing 2	MAP7D2	1,10
ADAM metallopeptidase with thrombospondin type 1 motif 7	ADAMTS7	1,10
potassium channel, two pore domain subfamily K, member 7	KCNK7	1,10
keratin associated protein 20-3	KRTAP20-3	1,10
tetratricopeptide repeat domain 1	TTC1	1,10
TAF12 RNA polymerase II, TATA box binding protein (TBP)-associated factor, 20kDa	TAF12	1,10
LSM3 homolog, U6 small nuclear RNA and mRNA degradation associated	LSM3	1,10
zinc finger and SCAN domain containing 21	ZSCAN21	1,10
cysteine-rich secretory protein 2	CRISP2	1,10
dual specificity phosphatase 12	DUSP12	1,10
unc-119 lipid binding chaperone B; microRNA 4700	UNC119B; MIR4700	1,10
cysteinyl-tRNA synthetase	CARS	1,10

olfactory receptor, family 4, subfamily C, member 13	OR4C13	1,10
myeloid/lymphoid or mixed-lineage leukemia; translocated to, 3	MLLT3	1,10
cytochrome P450, family 4, subfamily B, polypeptide 1	CYP4B1	1,10
abhydrolase domain containing 4	ABHD4	1,10
protein-L-isoaspartate (D-aspartate) O-methyltransferase	PCMT1	1,10
polycystic kidney disease 2 (autosomal dominant)	PKD2	1,10
chromosome 6 open reading frame 118	C6orf118	1,10
death-associated protein	DAP	1,10
defensin, beta 103A; defensin, beta 103B	DEFB103A; DEFB103B	1,10
zinc finger CCCH-type containing 18	ZC3H18	1,10
ADP-ribosylhydrolase like 2	ADPRHL2	1,10
WAP four-disulfide core domain 2	WFDC2	1,10
cholecystokinin A receptor	CCKAR	1,10
stress-induced phosphoprotein 1	STIP1	1,10
chromosome X open reading frame 66	CXorf66	1,10
guanylate binding protein 3	GBP3	1,10
nuclear pore complex interacting protein family, member B6	NPIPB6	1,10
CD70 molecule	CD70	1,10
zinc finger protein 141	ZNF141	1,10
Transcript Identified by AceView, Entrez Gene ID(s) 10558	SPTLC1	1,10
calcium channel, voltage-dependent, gamma subunit 2	CACNG2	1,10
tectonin beta-propeller repeat containing 2	TECPR2	1,10
tetratricopeptide repeat domain 19	TTC19	1,10
transmembrane BAX inhibitor motif containing 4	TMBIM4	1,10
RAS guanyl releasing protein 4	RASGRP4	1,10
solute carrier family 25, member 35	SLC25A35	1,10
CTP synthase 1	CTPS1	1,10
ADAMTS like 5	ADAMTSL5	1,09
topoisomerase (DNA) I, mitochondrial	TOP1MT	1,09
protein phosphatase 2, regulatory subunit B, gamma	PPP2R3C	1,09
collagen and calcium binding EGF domains 1	CCBE1	1,09
tubulin folding cofactor A	TBCA	1,09
leucine rich repeat containing 14B	LRRC14B	1,09
Transcript Identified by AceView, Entrez Gene ID(s) 57798; novel transcript, antisense to ERVW-1 and PEX1	AC007566.10; GATAD1	1,09
COBW domain containing 2	CBWD2	1,09
multiple EGF-like-domains 6	MEGF6	1,09
DDB1 and CUL4 associated factor 13	DCAF13	1,09
mitochondrial ribosomal protein L46	MRPL46	1,09
aldehyde dehydrogenase 1 family, member L1	ALDH1L1	1,09
G protein-coupled receptor associated sorting protein 1	GPRASP1	1,09
Salzman2013 ANTISENSE, coding, INTERNAL, intronic, OVERLAPTX best transcript NM_005603; novel transcript, antisense to ATP8B1	RP11-35G9.5; ATP8B1	1,09

methyltransferase like 11B	METTL11B	1,09
chromosome 18 open reading frame 21	C18orf21	1,09
succinate receptor 1	SUCNR1	1,09
coactosin-like F-actin binding protein 1	COTL1	1,09
carcinoembryonic antigen-related cell adhesion molecule 16	CEACAM16	1,09
titin	TTN	1,09
protein kinase C and casein kinase substrate in neurons 2	PACSIN2	1,09
transmembrane protein 176A	TMEM176A	1,09
schlafen like 1	SLFNL1	1,09
dynamin binding protein	DNMBP	1,09
coiled-coil domain containing 116	CCDC116	1,09
centrin 3	CETN3	1,09
core-binding factor, beta subunit	CBFB	1,09
RNA exonuclease 2	REXO2	1,09
KIAA1551	KIAA1551	1,09
zinc finger family member 788	ZNF788	1,09
RAN binding protein 3	RANBP3	1,09
serum/glucocorticoid regulated kinase 2	SGK2	1,09
LIM domain binding 2	LDB2	1,09
chromosome 6 open reading frame 132	C6orf132	1,09
lymphocyte antigen 6 complex, locus G6C	LY6G6C	1,09
transmembrane protein 120A	TMEM120A	1,09
solute carrier family 22 (organic cation transporter), member 18 antisense	SLC22A18AS	1,09
pellino E3 ubiquitin protein ligase family member 3	PELI3	1,09
collagen, type XIV, alpha 1	COL14A1	1,09
ATP binding cassette subfamily F member 2	ABCF2	1,09
interleukin 1 beta	IL1B	1,09
phosphatase and actin regulator 4	PHACTR4	1,09
monoamine oxidase A	MAOA	1,09
bromodomain containing 9	BRD9	1,09
solute carrier family 5 (sodium/multivitamin and iodide cotransporter), member 6	SLC5A6	1,09
NK2 homeobox 2	NKX2-2	1,09
interleukin 20 receptor, alpha	IL20RA	1,09
plexin B1	PLXNB1	1,09
charged multivesicular body protein 3	CHMP3	1,09
StAR-related lipid transfer domain containing 6	STARD6	1,09
Memczak2013 ANTISENSE, CDS, coding, INTERNAL best transcript NM_003075	SMARCC2	1,09
late cornified envelope 2D	LCE2D	1,09
thymidine kinase 2, mitochondrial	TK2	1,09
C-type lectin domain family 2, member L	CLEC2L	1,09
ret proto-oncogene	RET	1,09
KIT ligand	KITLG	1,09

olfactory receptor, family 10, subfamily A, member 5	OR10A5	1,09
alkB homolog 2, alpha-ketoglutarate-dependent dioxygenase	ALKBH2	1,09
Memczak2013 ANTISENSE, coding, INTERNAL, intronic best transcript NM_173464	L3MBTL4	1,09
C15orf38-AP3S2 readthrough	C15orf38-AP3S2	1,09
peptidylprolyl isomerase E (cyclophilin E)	PPIE	1,09
brain expressed X-linked 5	BEX5	1,09
interleukin 23 receptor	IL23R	1,09
zinc finger protein 829	ZNF829	1,09
olfactory receptor, family 5, subfamily V, member 1	OR5V1	1,09
integrin alpha FG-GAP repeat containing 2	ITFG2	1,09
BTB (POZ) domain containing 16	BTBD16	1,09
phosphogluconate dehydrogenase	PGD	1,09
mitogen-activated protein kinase binding protein 1	MAPKBP1	1,09
transmembrane protein 63C	TMEM63C	1,09
transmembrane protein 235	TMEM235	1,09
family with sequence similarity 134, member A	FAM134A	1,09
SUV3-like helicase	SUPV3L1	1,09
taste receptor, type 2, member 1	TAS2R1	1,09
homeobox A10; homeobox A9; microRNA 196b	HOXA10; HOXA9; MIR196B	1,09
ribonuclease H1	RNASEH1	1,09
endothelial differentiation-related factor 1	EDF1	1,09
SRR1 domain containing	SRRD	1,09
long intergenic non-protein coding RNA 243	LINC00243	1,09
mitochondrial ribosome recycling factor	MRRF	1,09
erythrocyte membrane protein band 4.1 like 5	EPB41L5	1,09
family with sequence similarity 109, member B	FAM109B	1,09
KIAA2022	KIAA2022	1,09
olfactory receptor, family 51, subfamily J, member 1 (gene/pseudogene)	OR51J1	1,09
chloride channel, voltage-sensitive 2	CLCN2	1,09
zinc finger CCCH-type, antiviral 1-like	ZC3HAV1L	1,09
DPY30 domain containing 2	DYDC2	1,09
CCZ1 homolog, vacuolar protein trafficking and biogenesis associated; CCZ1 homolog B, vacuolar protein trafficking and biogenesis associated	CCZ1; CCZ1B	1,09
sodium channel, voltage gated, type III beta subunit	SCN3B	1,09
TM4SF1 antisense RNA 1	TM4SF1-AS1	1,09
relaxin/insulin-like family peptide receptor 3	RXFP3	1,09
somatostatin receptor 3	SSTR3	1,09
Memczak2013 ALT_ACCEPTOR, ALT_DONOR, coding, INTERNAL, intronic best transcript NM_173683; novel transcript	AF131215.3; XKR6	1,09

mitochondrial intermediate peptidase	MIPEP	1,09
transmembrane p24 trafficking protein 1	TMED1	1,09
toll-like receptor 2	TLR2	1,09
serine/arginine-rich splicing factor 12	SRSF12	1,09
cytokine receptor-like factor 2	CRLF2	1,09
ceroid-lipofuscinosis, neuronal 5	CLN5	1,09
solute carrier family 35, member G5	SLC35G5	1,09
thymidylate synthetase	TYMS	1,09
complement component 5a receptor 1	C5AR1	1,09
programmed cell death 4 (neoplastic transformation inhibitor); microRNA 4680	PDCD4; MIR4680	1,09
family with sequence similarity 110, member D	FAM110D	1,09
proteasome activator subunit 3	PSME3	1,09
Transcript Identified by AceView, Entrez Gene ID(s) 51755	CDK12	1,09
calmodulin binding transcription activator 1	CAMTA1	1,09
nuclear receptor coactivator 1	NCOA1	1,09
zinc finger protein 790	ZNF790	1,09
cytochrome c oxidase subunit Vb	COX5B	1,09
TP53 induced glycolysis regulatory phosphatase	TIGAR	1,09
cytokine inducible SH2-containing protein	CISH	1,09
SPRY domain containing 7	SPRYD7	1,09
tripartite motif containing 64C	TRIM64C	1,09
AKT interacting protein	AKTIP	1,09
myogenic differentiation 1	MYOD1	1,09
abhydrolase domain containing 1	ABHD1	1,09
McKusick-Kaufman syndrome	MKKS	1,09
DENN/MADD domain containing 4A	DENND4A	1,09
apolipoprotein L, 6	APOL6	1,09
trimethyllysine hydroxylase, epsilon	TMLHE	1,09
Transcript Identified by AceView, Entrez Gene ID(s) 4211	MEIS1	1,09
phosphatase and actin regulator 3	PHACTR3	1,09
centrosomal protein 78kDa	CEP78	1,09
LKAAEAR motif containing 1	LKAAEAR1	1,09
transmembrane channel like 3	TMC3	1,09
leucine rich transmembrane and O-methyltransferase domain containing	LRTOMT	1,09
CD300c molecule	CD300C	1,09
HTR5A antisense RNA 1	HTR5A-AS1	1,09
RAB5B, member RAS oncogene family	RAB5B	1,09
TIA1 cytotoxic granule-associated RNA binding protein-like 1	TIAL1	1,09
RAB2A, member RAS oncogene family	RAB2A	1,09
fucosidase, alpha-L- 1, tissue	FUCA1	1,09
calmodulin 3 (phosphorylase kinase, delta)	CALM3	1,09
F-box and WD repeat domain containing 4	FBXW4	1,09
solute carrier family 7, member 4	SLC7A4	1,09

zinc finger protein 20	ZNF20	1,09
zymogen granule protein 16B	ZG16B	1,09
solute carrier family 1 (neutral amino acid transporter), member 5	SLC1A5	1,09
MAGE family member A5	MAGEA5	1,09
Zic family member 4	ZIC4	1,09
replication protein A1	RPA1	1,09
SH3 and PX domains 2A	SH3PXD2A	1,09
MON1 secretory trafficking family member B	MON1B	1,09
upregulator of cell proliferation	URGCP	1,09
chemokine (C-C motif) ligand 19	CCL19	1,09
benzodiazepine receptor (peripheral) associated protein 1	BZRAP1	1,09
eukaryotic translation initiation factor 2, subunit 2 beta, 38kDa	EIF2S2	1,09
bone morphogenetic protein/retinoic acid inducible neural-specific 1	BRINP1	1,09
long intergenic non-protein coding RNA 1205	LINC01205	1,09
Transcript Identified by AceView, Entrez Gene ID(s) 26010	SPATS2L	1,09
cytokine receptor-like factor 3	CRLF3	1,09
regulated endocrine-specific protein 18	RESP18	1,09
chromosome 1 open reading frame 137	C1orf137	1,09
immunoglobulin-like domain containing receptor 2	ILDR2	1,09
TBC1 domain family, member 15	TBC1D15	1,09
chromosome 2 open reading frame 44	C2orf44	1,09
SET domain, bifurcated 1	SETDB1	1,09
family with sequence similarity 170, member B	FAM170B	1,09
matrin 3; small nucleolar RNA, H/ACA box 74A; small nucleolar RNA host gene 4	MATR3; SNORA74A; SNHG4	1,09
myosin light chain 2	MYL2	1,09
golgin A6 family-like 3	GOLGA6L3	1,09
zinc finger protein 696	ZNF696	1,09
nescient helix-loop-helix 1	NHLH1	1,09
BLOC1S5-TXNDC5 readthrough (NMD candidate)	BLOC1S5-TXNDC5	1,09
T-box 22	TBX22	1,09
proteasome 26S subunit, non-ATPase 3	PSMD3	1,09
GLI pathogenesis-related 1 like 2	GLIPR1L2	1,09
dynein, axonemal, assembly factor 1	DNAAF1	1,09
nicotinate phosphoribosyltransferase	NAPRT	1,09
nescient helix-loop-helix 2	NHLH2	1,09
SRC kinase signaling inhibitor 1	SRCIN1	1,09
piwi-like RNA-mediated gene silencing 4	PIWIL4	1,09
Williams-Beuren syndrome chromosome region 28	WBSCR28	1,09
ADP-ribosylation factor like GTPase 17B	ARL17B	1,09
heat shock 70kDa protein 9 (mortalin)	HSPA9	1,09

protein phosphatase 1, regulatory subunit 15A	PPP1R15A	1,09
ALG1, chitobiosyldiphosphodolichol beta-mannosyltransferase-like	ALG1L	1,09
orofacial cleft 1 candidate 1	OFCC1	1,09
retinoic acid receptor, gamma	RARG	1,09
SUMO1 activating enzyme subunit 1	SAE1	1,09
UFM1-specific peptidase 2	UFSP2	1,09
Kruppel-like factor 3 (basic)	KLF3	1,09
thioredoxin-like 1	TXNL1	1,09
outer dense fiber of sperm tails 2	ODF2	1,09
DnaJ (Hsp40) homolog, subfamily B, member 7	DNAJB7	1,09
hypoxia up-regulated 1	HYOU1	1,09
ankyrin-repeat and fibronectin type III domain containing 1	ANKFN1	1,09
zinc finger protein 778	ZNF778	1,09
regulator of microtubule dynamics 3	RMDN3	1,09
isocitrate dehydrogenase 3 (NAD+) gamma	IDH3G	1,09
DMRT-like family B with proline-rich C-terminal, 1	DMRTB1	1,09
small nuclear ribonucleoprotein polypeptide A	SNRPA1	1,09
family with sequence similarity 20, member C	FAM20C	1,09
caspase 6	CASP6	1,09
gastrulation brain homeobox 2	GBX2	1,09
DEAH (Asp-Glu-Ala-His) box polypeptide 29	DHX29	1,09
Jeck2013 ANTISENSE, coding, INTERNAL, OVEXON, UTR3 best transcript NM_001039690	CHTF8	1,09
axonemal dynein light chain domain containing 1	AXDND1	1,09
transmembrane 9 superfamily member 2	TM9SF2	1,09
REX1, RNA exonuclease 1 homolog-like 4, pseudogene	REXO1L4P	1,09
LDL receptor related protein 4	LRP4	1,09
protein tyrosine phosphatase, receptor type, T	PTPRT	1,09
LIM homeobox 9	LHX9	1,09
follicle stimulating hormone receptor	FSHR	1,09
transmembrane protein 200B	TMEM200B	1,09
carboxypeptidase A4	CPA4	1,09
chromodomain protein, Y-linked, 2A	CDY2A	1,09
tribbles pseudokinase 2	TRIB2	1,09
nephrosis 1, congenital, Finnish type (nephrin)	NPHS1	1,09
KRAB-A domain containing 2	KRBA2	1,09
chemokine (C motif) receptor 1	XCR1	1,09
excision repair cross-complementation group 2	ERCC2	1,09
tRNA-histidine guanylyltransferase 1-like	THG1L	1,09
REM2 and RAB-like small GTPase 1	RSG1	1,09
Memczak2013 ANTISENSE, CDS, coding, INTERNAL, UTR3 best transcript NM_022727	TRMT2A	1,09
hedgehog acyltransferase	HHAT	1,09
RFPL3 antisense	RFPL3S	1,09

LY6/PLAUR domain containing 5	LYPD5	1,09
transmembrane protein 212	TMEM212	1,09
C1q and tumor necrosis factor related protein 5; membrane frizzled-related protein	C1QTNF5; MFRP	1,09
Transcript Identified by AceView, Entrez Gene ID(s) 9045	RPL14	1,09
chymase 1, mast cell	CMA1	1,09
DEF6 guanine nucleotide exchange factor	DEF6	1,09
Zhang2013 ALT_ACCEPTOR, ALT_DONOR, coding, INTERNAL, intronic best transcript NM_152227	SNX5	1,09
dual specificity tyrosine-(Y)-phosphorylation regulated kinase 1B	DYRK1B	1,09
zinc finger protein 772	ZNF772	1,09
X-linked Kx blood group related 7	XKR7	1,09
CD55 molecule, decay accelerating factor for complement (Cromer blood group)	CD55	1,09
MAGE family member B6	MAGEB6	1,09
glutamate receptor, metabotropic 4	GRM4	1,09
schlafen family member 13	SLFN13	1,09
FOS-like antigen 2	FOSL2	1,09
jade family PHD finger 2	JADE2	1,09
glycyl-tRNA synthetase	GARS	1,09
defensin, beta 133	DEFB133	1,09
GTPase, IMAP family member 6	GIMAP6	1,09
aryl hydrocarbon receptor nuclear translocator	ARNT	1,09
olfactory receptor, family 4, subfamily L, member 1	OR4L1	1,09
xylulokinase homolog (H. influenzae)	XYLB	1,09
Transcript Identified by AceView, Entrez Gene ID(s) 8792	TNFRSF11A	1,09
complement component 5	C5	1,09
ectonucleoside triphosphate diphosphohydrolase 1	ENTPD1	1,09
plakophilin 1	PKP1	1,09
ceroid-lipofuscinosis, neuronal 3; nuclear pore complex interacting protein family, member B7	CLN3; NPIPB7	1,09
interleukin 17D	IL17D	1,09
MCM3AP antisense RNA 1	MCM3AP-AS1	1,09
interleukin 22	IL22	1,09
natriuretic peptide A	NPPA	1,09
elongator acetyltransferase complex subunit 5	ELP5	1,09
reticulocalbin 1, EF-hand calcium binding domain	RCN1	1,09
F-box protein 41	FBXO41	1,09
sorting nexin 3	SNX3	1,09
trafficking protein particle complex 2-like	TRAPPC2L	1,09
cytochrome P450, family 2, subfamily E, polypeptide 1	CYP2E1	1,09
transient receptor potential cation channel, subfamily V, member 2	TRPV2	1,09
stromal antigen 3-like 2 (pseudogene)	STAG3L2	1,09
A kinase (PRKA) anchor protein 5	AKAP5	1,09

family with sequence similarity 3, member B	FAM3B	1,09
	LRIG1	1,09
integrin beta 1 binding protein (melusin) 2	ITGB1BP2	1,09
acrosomal vesicle protein 1	ACRV1	1,09
ring finger protein 10	RNF10	1,09
filamin binding LIM protein 1	FBLIM1	1,09
unc-5 netrin receptor C	UNC5C	1,09
metastasis suppressor 1	MTSS1	1,09
LDL receptor related protein 1B	LRP1B	1,09
biogenesis of lysosomal organelles complex-1, subunit 2	BLOC1S2	1,09
discs, large homolog 3 (Drosophila)	DLG3	1,09
patatin-like phospholipase domain containing 3	PNPLA3	1,09
ataxin 3	ATXN3	1,09
zinc finger protein 763	ZNF763	1,09
galactose-1-phosphate uridylyltransferase	GALT	1,09
REX1, RNA exonuclease 1 homolog-like 2, pseudogene	REXO1L2P	1,09
hydroxysteroid (17-beta) dehydrogenase 8	HSD17B8	1,09
Memczak2013 ALT_ACCEPTOR, ALT_DONOR, coding, INTERNAL, intronic best transcript NM_001170794	BACH2	1,09
family with sequence similarity 163, member B	FAM163B	1,09
GDP dissociation inhibitor 1	GDI1	1,09
Salzman2013 ANTISENSE, CDS, coding, INTERNAL, intronic, OVCODE, OVERLAPTX, OVEXON, UTR5 best transcript NM_005603	ATP8B1	1,09
multimerin 2	MMRN2	1,09
G protein-coupled receptor 27	GPR27	1,09
IGF2 antisense RNA	IGF2-AS	1,09
PRC1 antisense RNA 1	PRC1-AS1	1,09
family with sequence similarity 189, member A1	FAM189A1	1,09
tripartite motif containing 35	TRIM35	1,09
otogelin-like	OTOGL	1,09
family with sequence similarity 25, member C	FAM25C	1,09
enhancer of polycomb homolog 2 (Drosophila)	EPC2	1,09
potassium channel, two pore domain subfamily K, member 12	KCNK12	1,09
transglutaminase 1	TGM1	1,09
nitric oxide synthase trafficking	NOSTRIN	1,09
zinc finger, GRF-type containing 1	ZGRF1	1,09
HIG1 hypoxia inducible domain family, member 2A	HIGD2A	1,09
family with sequence similarity 153, member C, pseudogene	FAM153C	1,09
E74-like factor 5 (ets domain transcription factor)	ELF5	1,09
AI894139 pseudogene; Transcript Identified by AceView, Entrez Gene ID(s) 155060	LOC155060; ZNF783	1,09
echinoderm microtubule associated protein like 1	EML1	1,09
EF-hand calcium binding domain 8	EFCAB8	1,09
scaffold attachment factor B	SAFB	1,09

autophagy related 4D, cysteine peptidase	ATG4D	1,09
glycine receptor alpha 4	GLRA4	1,09
small nuclear ribonucleoprotein, U11/U12 25kDa subunit	SNRNP25	1,09
leucine-rich repeat, immunoglobulin-like and transmembrane domains 1	LRIT1	1,09
aldehyde dehydrogenase 1 family, member A1	ALDH1A1	1,09
hypoxia inducible factor 1, alpha subunit (basic helix-loop-helix transcription factor)	HIF1A	1,09
prostate and testis expressed 2	PATE2	1,09
phenazine biosynthesis-like protein domain containing	PBLD	1,09
Cbl proto-oncogene B, E3 ubiquitin protein ligase	CBLB	1,09
disrupted in renal carcinoma 2	DIRC2	1,09
membrane associated ring finger 4	MARCH4	1,09
RAS-like, family 11, member A	RASL11A	1,09
zinc finger, SWIM-type containing 8	ZSWIM8	1,09
RAB20, member RAS oncogene family	RAB20	1,09
family with sequence similarity 83, member B	FAM83B	1,09
olfactory receptor, family 5, subfamily A, member 1	OR5A1	1,09
KIF25 antisense RNA 1	KIF25-AS1	1,09
envoplakin-like	EVPLL	1,09
EF-hand domain (C-terminal) containing 2	EFHC2	1,09
TAF10 RNA polymerase II, TATA box binding protein (TBP)-associated factor, 30kDa	TAF10	1,09
GA binding protein transcription factor, beta subunit 1	GABPB1	1,09
sclerostin	SOST	1,09
epoxide hydrolase 1, microsomal (xenobiotic)	EPHX1	1,09
polyhomeotic homolog 2 (Drosophila); microRNA 3605	PHC2; MIR3605	1,09
chromosome 14 open reading frame 105	C14orf105	1,09
phosphatase, orphan 1	PHOSPHO1	1,09
tolloid like 2	TLL2	1,09
ribosomal protein L7a	RPL7A	1,09
hypoxia up-regulated 1	HYOU1	1,09
peptidoglycan recognition protein 3	PGLYRP3	1,09
phosphoribosyl pyrophosphate synthetase 2	PRPS2	1,09
cadherin 11, type 2, OB-cadherin (osteoblast)	CDH11	1,09
p53 and DNA-damage regulated 1	PDRG1	1,09
coronin 7; presequence translocase-associated motor 16 homolog (S. cerevisiae); CORO7-PAM16 readthrough	CORO7; PAM16; CORO7-PAM16	1,09
siah E3 ubiquitin protein ligase 2	SIAH2	1,09
malignant T-cell amplified sequence 1	MCTS1	1,09
missing oocyte, meiosis regulator, homolog (Drosophila)	MIOS	1,09
PWP1 homolog, endonuclein	PWP1	1,09
LMO7 downstream neighbor	LMO7DN	1,09
protocadherin beta 1	PCDHB1	1,09

PTGES3L-AARSD1 readthrough; prostaglandin E synthase 3 (cytosolic)-like; alanyl-tRNA synthetase domain containing 1	PTGES3L-AARSD1; PTGES3L; AARSD1	1,09
keratin associated protein 4-4	KRTAP4-4	1,09
insulin-like 6	INSL6	1,09
SPANX family, member N2	SPANXN2	1,09
microtubule associated serine/threonine kinase 2	MAST2	1,09
uncharacterized LOC100287036; novel transcript, antisense to ANKRD11	LOC100287036; AC137932.6	1,09
fibrinogen like 1	FGL1	1,09
major vault protein; PAXIP1 associated glutamate-rich protein 1	MVP; PAGR1	1,09
Memczak2013 ANTISENSE, CDS, coding, INTERNAL best transcript NM_003123	SPN	1,09
C-type lectin domain family 19, member A	CLEC19A	1,09
calbindin 1	CALB1	1,09
tetra-peptide repeat homeobox-like	TPRXL	1,09
defensin, beta 109, pseudogene 1B	DEFB109P1B	1,09
olfactory receptor, family 5, subfamily P, member 3	OR5P3	1,09
armadillo repeat containing 5	ARMC5	1,09
Bardet-Biedl syndrome 4	BBS4	1,09
MLX interacting protein	MLXIP	1,09
sarcoglycan beta	SGCB	1,09
thymidine kinase 2, mitochondrial	TK2	1,09
thrombopoietin	THPO	1,09
suppressor of cytokine signaling 4	SOCS4	1,09
cystatin 9 (testatin)	CST9	1,09
Transcript Identified by AceView, Entrez Gene ID(s) 65977	PLEKHA3	1,09
kelch-like family member 29	KLHL29	1,09
defensin, beta 112	DEFB112	1,09
CD200 molecule	CD200	1,09
de-etiolated homolog 1 (Arabidopsis)	DET1	1,09
high mobility group nucleosome binding domain 1	HMGN1	1,09
CNKSR family member 3	CNKSR3	1,09
PRAME family member 19 [Source:EntrezGene;Acc:645414]	PRAMEF19	1,09
olfactory receptor, family 51, subfamily L, member 1	OR51L1	1,09
PET117 homolog; CSRP2 binding protein	PET117; CSRP2BP	1,09
poly-U binding splicing factor 60KDa	PUF60	1,09
Jeck2013 ALT_ACCEPTOR, ALT_DONOR, coding, INTERNAL, intronic best transcript NM_001014797; Transcript Identified by AceView	KCNMA1; swamu	1,09
cell adhesion molecule 2	CADM2	1,09
zinc finger protein 471	ZNF471	1,09

testis specific protein, Y-linked 3; testis specific protein, Y-linked 4; testis specific protein, Y-linked 8; testis specific protein, Y-linked 1	TSPY3; TSPY4; TSPY8; TSPY1	1,09
tripartite motif containing 47	TRIM47	1,09
NFKB activating protein	NKAP	1,09
fucosyltransferase 3 (galactoside 3(4)-L-fucosyltransferase, Lewis blood group)	FUT3	1,09
erythrocyte membrane protein band 4.1 like 4B	EPB41L4B	1,09
uroporphyrinogen III synthase	UROS	1,09
coiled-coil domain containing 167	CCDC167	1,09
autophagy related 4C, cysteine peptidase	ATG4C	1,09
phosphatidylethanolamine binding protein 1	PEBP1	1,09
centrosomal protein 104kDa	CEP104	1,09
abhydrolase domain containing 18	ABHD18	1,09
dual specificity phosphatase 4	DUSP4	1,09
NUAK family, SNF1-like kinase, 2	NUAK2	1,09
phosphatidylinositol glycan anchor biosynthesis class G	PIGG	1,09
myeloid-derived growth factor	MYDGF	1,09
myosin, heavy chain 3, skeletal muscle, embryonic	MYH3	1,09
family with sequence similarity 21, member A	FAM21A	1,09
dopa decarboxylase	DDC	1,09
transmembrane protein 117	TMEM117	1,09
chromosome 19 open reading frame 24	C19orf24	1,09
Dab, reelin signal transducer, homolog 1 (Drosophila)	DAB1	1,09
gap junction protein beta 7	GJB7	1,09
keratin associated protein 10-11; keratin associated protein 12-3; keratin associated protein 10-12	KRTAP10-11; KRTAP12-3; KRTAP10-12	1,09
chromosome 1 open reading frame 50	C1orf50	1,09
alkylglycerol monooxygenase	AGMO	1,09
chromosome 1 open reading frame 105	C1orf105	1,09
RNA pseudouridylate synthase domain containing 1	RPUSD1	1,09
ring finger protein 112	RNF112	1,09
ATPase, H+ transporting, lysosomal accessory protein 1	ATP6AP1	1,09
tetraspanin 10	TSPAN10	1,09
ATP/GTP binding protein-like 5	AGBL5	1,09
NADH dehydrogenase (ubiquinone) complex I, assembly factor 3	NDUFAF3	1,09
coiled-coil domain containing 106	CCDC106	1,09
ankyrin repeat domain 55	ANKRD55	1,09
integrator complex subunit 10	INTS10	1,09
Src homology 2 domain containing transforming protein D	SHD	1,09
peroxisomal biogenesis factor 16	PEX16	1,09
multiciliate differentiation and DNA synthesis associated cell cycle protein	MCIDAS	1,09
Fanconi anemia complementation group D2	FANCD2	1,09

arginine/serine-rich protein 1	RSRP1	1,09
solute carrier family 9, subfamily A (NHE8, cation proton antiporter 8), member 8	SLC9A8	1,09
stromal antigen 2	STAG2	1,09
TATA box binding protein (TBP)-associated factor, RNA polymerase I, B, 63kDa	TAF1B	1,09
SP100 nuclear antigen	SP100	1,09
zinc finger protein 365	ZNF365	1,09
interferon regulatory factor 6	IRF6	1,09
chemokine (C-X-C motif) ligand 12	CXCL12	1,09
zinc finger protein 28	ZNF28	1,09
zinc finger protein 502	ZNF502	1,09
ZFP14 zinc finger protein	ZFP14	1,09
myelin-associated oligodendrocyte basic protein	MOBP	1,09
dishevelled segment polarity protein 3	DVL3	1,09
parvin, alpha	PARVA	1,09
zinc finger, HIT-type containing 2	ZNHIT2	1,09
purine-rich element binding protein B; microRNA 4657	PURB; MIR4657	1,09
general transcription factor IIIC subunit 1	GTF3C1	1,09
CUB and Sushi multiple domains 1	CSMD1	1,09
cytochrome b reductase 1	CYBRD1	1,09
prune exopolyphosphatase	PRUNE	1,09
POTE ankyrin domain family, member C	POTEC	1,09
WD repeat domain 72	WDR72	1,09
chorionic gonadotropin, beta polypeptide; chorionic gonadotropin, beta polypeptide 5	CGB; CGB5	1,09
vacuolar protein sorting 13 homolog D (S. cerevisiae)	VPS13D	1,09
long intergenic non-protein coding RNA 1578	LINC01578	1,09
C2 calcium-dependent domain containing 4A	C2CD4A	1,09
aldo-keto reductase family 1, member E2	AKR1E2	1,09
kelch domain containing 4	KLHDC4	1,09
rhomboid 5 homolog 2 (Drosophila)	RHBDF2	1,09
microtubule-associated protein 1 light chain 3 alpha	MAP1LC3A	1,09
transcription factor 20 (AR1)	TCF20	1,09
transcription factor 20 (AR1)	TCF20	1,09
chromosome 20 open reading frame 85	C20orf85	1,09
eukaryotic translation initiation factor 6	EIF6	1,09
small nuclear ribonucleoprotein, U5 200kDa subunit	SNRNP200	1,09
sema domain, seven thrombospondin repeats (type 1 and type 1-like), transmembrane domain (TM) and short cytoplasmic domain, (semaphorin) 5A	SEMA5A	1,09
vomeronasal 1 receptor 2	VN1R2	1,09
leucine rich repeat containing 47	LRRC47	1,09
G protein-coupled receptor 82	GPR82	1,09

proline rich Gla (G-carboxyglutamic acid) 3 (transmembrane)	PRRG3	1,09
integrator complex subunit 6	INTS6	1,09
SNF2 histone linker PHD RING helicase, E3 ubiquitin protein ligase	SHPRH	1,09
potassium channel, calcium activated intermediate/small conductance subfamily N alpha, member 3	KCNN3	1,09
cyclin Y	CCNY	1,09
inositol polyphosphate-5-phosphatase D	INPP5D	1,09
ADP-ribosylation factor like GTPase 5A	ARL5A	1,09
store-operated calcium entry-associated regulatory factor	SARAF	1,09
hypoxia up-regulated 1	HYOU1	1,09
ubiquitin specific peptidase 54	USP54	1,09
tumor protein D52-like 1	TPD52L1	1,09
thymocyte selection associated family member 2	THEMIS2	1,09
solute carrier family 9, subfamily A (NHE6, cation proton antiporter 6), member 6	SLC9A6	1,09
TSR1, 20S rRNA accumulation, homolog (S. cerevisiae)	TSR1	1,09
TATA box binding protein associated factor 7 like	TAF7L	1,09
X-linked inhibitor of apoptosis, E3 ubiquitin protein ligase	XIAP	1,09
claudin 20	CLDN20	1,09
retinol dehydrogenase 13 (all-trans/9-cis)	RDH13	1,09
noggin	NOG	1,09
nudix hydrolase 8	NUDT8	1,09
astrotactin 2	ASTN2	1,09
voltage-dependent anion channel 2	VDAC2	1,09
solute carrier family 16 (monocarboxylate transporter), member 1	SLC16A1	1,09
small nuclear ribonucleoprotein D1 polypeptide	SNRPD1	1,09
ADAMTS like 1	ADAMTSL1	1,09
SH3 domain binding glutamate-rich protein like	SH3BGRL	1,09
potassium channel, voltage gated shaker related subfamily A, member 7	KCNA7	1,08
collagen, type XVII, alpha 1; microRNA 936	COL17A1; MIR936	1,08
melanocortin 5 receptor	MC5R	1,08
adducin 3 (gamma)	ADD3	1,08
T-box 18	TBX18	1,08
cytochrome P450, family 26, subfamily B, polypeptide 1	CYP26B1	1,08
sema domain, immunoglobulin domain (Ig), short basic domain, secreted, (semaphorin) 3E	SEMA3E	1,08
ATPase, aminophospholipid transporter, class I, type 8B, member 2	ATP8B2	1,08
family with sequence similarity 27-like	FAM27L	1,08
twinfilin actin binding protein 1	TWF1	1,08
5,3-nucleotidase, mitochondrial	NT5M	1,08

SSU72 homolog, RNA polymerase II CTD phosphatase	SSU72	1,08
isopentenyl-diphosphate delta isomerase 2	IDI2	1,08
paired box 6	PAX6	1,08
lymphoblastic leukemia associated hematopoiesis regulator 1	LYL1	1,08
4-hydroxy-2-oxoglutarate aldolase 1	HOGA1	1,08
solute carrier family 35, member F1	SLC35F1	1,08
LDL receptor related protein 6	LRP6	1,08
Fli-1 proto-oncogene, ETS transcription factor	FLI1	1,08
complement component 1, s subcomponent	C1S	1,08
transducin (beta)-like 2	TBL2	1,08
myeloid leukemia factor 1	MLF1	1,08
Rho guanine nucleotide exchange factor 5	ARHGEF5	1,08
uncharacterized LOC150527; uncharacterized LOC646743; Transcript Identified by AceView, Entrez Gene ID(s) 646743, RefSeq ID(s) NR_033930; Uncharacterized protein [Source:UniProtKB/TrEMBL;Acc:B9A039]	TISP43; LOC646743; AC013269.5	1,08
cleavage and polyadenylation factor I subunit 1	CLP1	1,08
opsin 4	OPN4	1,08
protein phosphatase, EF-hand calcium binding domain 1	PPEF1	1,08
regenerating islet-derived 3 gamma	REG3G	1,08
ribosomal protein L27	RPL27	1,08
coiled-coil domain containing 27	CCDC27	1,08
AT rich interactive domain 4B (RBP1-like)	ARID4B	1,08
signal peptide, CUB domain, EGF-like 1	SCUBE1	1,08
inositol polyphosphate-5-phosphatase D	INPP5D	1,08
Transcript Identified by AceView, Entrez Gene ID(s) 5747	PTK2	1,08
salt-inducible kinase 2	SIK2	1,08
GABA(A) receptor-associated protein like 1	GABARAPL1	1,08
KIAA0513	KIAA0513	1,08
EF-hand calcium binding domain 3	EFCAB3	1,08
calcyclin binding protein	CACYBP	1,08
ninein-like	NINL	1,08
pre-mRNA processing factor 4	PRPF4	1,08
Kruppel-like factor 15	KLF15	1,08
zinc finger and BTB domain containing 46	ZBTB46	1,08
EPH receptor B1	EPHB1	1,08
proline and serine rich 1	PROSER1	1,08
intraflagellar transport 81	IFT81	1,08
DEAD (Asp-Glu-Ala-Asp) box helicase 42	DDX42	1,08
neuronal tyrosine-phosphorylated phosphoinositide-3-kinase adaptor 2	NYAP2	1,08
KiSS-1 metastasis-suppressor	KISS1	1,08
nuclear receptor interacting protein 2	NRIP2	1,08
PR domain containing 9	PRDM9	1,08
PC-esterase domain containing 1A	PCED1A	1,08

tigger transposable element derived 3	TIGD3	1,08
tropomodulin 3 (ubiquitous)	TMOD3	1,08
endogenous retrovirus group K13, member 1	ERVK13-1	1,08
carnitine palmitoyltransferase 1A (liver)	CPT1A	1,08
zinc finger protein 546	ZNF546	1,08
major facilitator superfamily domain containing 6-like	MFSD6L	1,08
BLOC-1 related complex subunit 5	BORCS5	1,08
multiple EGF-like-domains 11	MEGF11	1,08
solute carrier family 5 (sodium/iodide cotransporter), member 5	SLC5A5	1,08
ADAM metallopeptidase domain 11	ADAM11	1,08
regulating synaptic membrane exocytosis 3	RIMS3	1,08
shroom family member 3	SHROOM3	1,08
yippee like 4	YPEL4	1,08
chromosome 7 open reading frame 57	C7orf57	1,08
Jeck2013 ALT_ACCEPTOR, ALT_DONOR, coding, INTERNAL, intronic best transcript NM_001100164	PHACTR2	1,08
calcium/calmodulin-dependent protein kinase II alpha	CAMK2A	1,08
isochorismatase domain containing 2	ISOC2	1,08
fibroblast growth factor 13; long intergenic non-protein coding RNA 889	FGF13; LINC00889	1,08
signal transducer and activator of transcription 4	STAT4	1,08
olfactory receptor, family 10, subfamily S, member 1	OR10S1	1,08
plexin A1	PLXNA1	1,08
olfactory receptor, family 12, subfamily D, member 2 (gene/pseudogene)	OR12D2	1,08
olfactory receptor, family 7, subfamily G, member 3	OR7G3	1,08
cysteinyl-tRNA synthetase 2, mitochondrial (putative)	CARS2	1,08
serpin peptidase inhibitor, clade A (alpha-1 antiproteinase, antitrypsin), member 4	SERPINA4	1,08
solute carrier family 27 (fatty acid transporter), member 1	SLC27A1	1,08
3(2), 5-bisphosphate nucleotidase 1	BPNT1	1,08
zona pellucida glycoprotein 1 (sperm receptor)	ZP1	1,08
protein kinase C, zeta	PRKCZ	1,08
ceroid-lipofuscinosis, neuronal 6, late infantile, variant	CLN6	1,08
pyruvate dehyrogenase phosphatase catalytic subunit 2	PDP2	1,08
neuroblastoma breakpoint family, member 12	NBPF12	1,08
EF-hand and coiled-coil domain containing 1	EFCC1	1,08
neuronal pentraxin II	NPTX2	1,08
APH1A gamma secretase subunit	APH1A	1,08
excision repair cross-complementation group 3	ERCC3	1,08
dual specificity phosphatase 8	DUSP8	1,08
ribonucleoprotein, PTB-binding 2	RAVER2	1,08
kelch-like family member 42	KLHL42	1,08
ubiquitin specific peptidase 25	USP25	1,08

sphingosine-1-phosphate receptor 4	S1PR4	1,08
SEBOX homeobox	SEBOX	1,08
MYC-associated zinc finger protein (purine-binding transcription factor)	MAZ	1,08
SH3 and multiple ankyrin repeat domains 2	SHANK2	1,08
signal recognition particle receptor, B subunit	SRPRB	1,08
adaptor-related protein complex 3, delta 1 subunit	AP3D1	1,08
	LRIG1	1,08
cathepsin F	CTSF	1,08
cleavage and polyadenylation specific factor 3	CPSF3	1,08
prickle homolog 1	PRICKLE1	1,08
spermatogenesis associated 19	SPATA19	1,08
zinc finger protein 506	ZNF506	1,08
NADPH dependent diflavin oxidoreductase 1	NDOR1	1,08
Zhang2013 ALT_ACCEPTOR, ALT_DONOR, coding, INTERNAL, intronic best transcript NM_020338	ZMIZ1	1,08
SMAD family member 2	SMAD2	1,08
Memczak2013 ALT_ACCEPTOR, ALT_DONOR, coding, INTERNAL, intronic best transcript NM_014938	MLXIP	1,08
prostaglandin-endoperoxide synthase 1 (prostaglandin G/H synthase and cyclooxygenase)	PTGS1	1,08
MAX dimerization protein 3	MXD3	1,08
tripartite motif containing 67	TRIM67	1,08
serine/threonine kinase 17b	STK17B	1,08
chordin-like 1	CHRDL1	1,08
transport and golgi organization 6 homolog	TANGO6	1,08
deoxyribose-phosphate aldolase (putative)	DERA	1,08
Memczak2013 ANTISENSE, CDS, coding, upstream_start, UTR3, UTR5 best transcript NM_003523	HIST1H2BE	1,08
ST3 beta-galactoside alpha-2,3-sialyltransferase 4	ST3GAL4	1,08
membrane protein, palmitoylated 4	MPP4	1,08
poly(A) binding protein, cytoplasmic 5	PABPC5	1,08
ribosomal protein L15	RPL15	1,08
armadillo repeat containing, X-linked 2	ARMCX2	1,08
protein phosphatase 2, catalytic subunit, alpha isozyme	PPP2CA	1,08
CEBPZ opposite strand	CEBPZOS	1,08
sideroflexin 4	SFXN4	1,08
transmembrane 4 L six family member 4	TM4SF4	1,08
ribokinase; BRE antisense RNA 1	RBKS; BRE-AS1	1,08
lysyl-tRNA synthetase	KARS	1,08
zinc finger, DHHC-type containing 3	ZDHHC3	1,08
tuberous sclerosis 2	TSC2	1,08
RAB10, member RAS oncogene family	RAB10	1,08
mannosidase, alpha, class 2C, member 1	MAN2C1	1,08
ectonucleotide pyrophosphatase/phosphodiesterase 7	ENPP7	1,08

zinc finger protein 100	ZNF100	1,08
chloride channel, voltage-sensitive 1	CLCN1	1,08
ras-related C3 botulinum toxin substrate 3 (rho family, small GTP binding protein Rac3)	RAC3	1,08
splicing factor proline/glutamine-rich	SFPQ	1,08
serine/arginine-rich splicing factor 4	SRSF4	1,08
ST6 (alpha-N-acetyl-neuraminyl-2,3-beta-galactosyl-1,3)-N-acetylgalactosaminide alpha-2,6-sialyltransferase 5	ST6GALNAC5	1,08
ArfGAP with SH3 domain, ankyrin repeat and PH domain 1; ASAP1 intronic transcript 2	ASAP1; ASAP1-IT2	1,08
sterile alpha motif domain containing 12	SAMD12	1,08
transient receptor potential cation channel, subfamily M, member 7	TRPM7	1,08
ankyrin repeat and SOCS box containing 12	ASB12	1,08
mediator of DNA-damage checkpoint 1	MDC1	1,08
mitochondrial carrier 1	MTCH1	1,08
lipoma HMGIC fusion partner-like 5	LHFPL5	1,08
keratin associated protein 6-1	KRTAP6-1	1,08
double homeobox 4 like 24; double homeobox 4 like 23	DUX4L24; DUX4L23	1,08
double homeobox 4 like 22; double homeobox 4 like 21	DUX4L22; DUX4L21	1,08
double homeobox 4 like 10; double homeobox 4 like 11	DUX4L10; DUX4L11	1,08
keratin associated protein 21-1	KRTAP21-1	1,08
armadillo repeat gene deleted in velocardiofacial syndrome	ARVCF	1,08
RNA binding motif protein 12; copine I	RBM12; CPNE1	1,08
procollagen C-endopeptidase enhancer	PCOLCE	1,08
keratin associated protein 10-4	KRTAP10-4	1,08
zinc finger protein 511	ZNF511	1,08
ATP synthase, H+ transporting, mitochondrial F1 complex, O subunit	ATP5O	1,08
solute carrier family 32 (GABA vesicular transporter), member 1	SLC32A1	1,08
solute carrier family 9, subfamily A (NHE1, cation proton antiporter 1), member 1	SLC9A1	1,08
cyclin-dependent kinase 10	CDK10	1,08
methyltransferase like 7A	METTL7A	1,08
chromosome 9 open reading frame 172	C9orf172	1,08
coiled-coil domain containing 185	CCDC185	1,08
ubiquitin specific peptidase 4 (proto-oncogene)	USP4	1,08
NADH dehydrogenase (ubiquinone) 1 alpha subcomplex, 6, 14kDa	NDUFA6	1,08
solute carrier family 12 (potassium/chloride transporter), member 6	SLC12A6	1,08
growth hormone releasing hormone receptor	GHRHR	1,08

pregnancy specific beta-1-glycoprotein 7 (gene/pseudogene)	PSG7	1,08
espin-like	ESPNL	1,08
long intergenic non-protein coding RNA 935	LINC00935	1,08
solute carrier family 16 (monocarboxylate transporter), member 5	SLC16A5	1,08
early B-cell factor 1	EBF1	1,08
WD repeat domain 77	WDR77	1,08
homeobox C11	HOXC11	1,08
galactosidase, alpha	GLA	1,08
collagen, type IV, alpha 3 (Goodpasture antigen) binding protein	COL4A3BP	1,08
small nuclear ribonucleoprotein polypeptide A	SNRPA	1,08
adaptor-related protein complex 1, beta 1 subunit	AP1B1	1,08
selectin P ligand	SELPLG	1,08
Jeck2013 ALT_ACCEPTOR, ALT_DONOR, coding, INTERNAL, intronic best transcript NM_000127; Transcript Identified by AceView	EXT1; hunera	1,08
POU class 1 homeobox 1	POU1F1	1,08
polymerase (RNA) I polypeptide E	POLR1E	1,08
topoisomerase (DNA) III beta	TOP3B	1,08
nuclear pore complex interacting protein family, member B3	NPIPB3	1,08
hydroxysteroid dehydrogenase like 1	HSDL1	1,08
Nanog homeobox	NANOG	1,08
serine hydroxymethyltransferase 2 (mitochondrial)	SHMT2	1,08
basic helix-loop-helix family, member a9	BHLHA9	1,08
coagulation factor II (thrombin) receptor-like 1	F2RL1	1,08
intraflagellar transport 172	IFT172	1,08
retrotransposon gag domain containing 4	RGAG4	1,08
fatty acid amide hydrolase	FAAH	1,08
BCL2-like 14 (apoptosis facilitator)	BCL2L14	1,08
zinc finger CCCH-type containing 8	ZC3H8	1,08
interferon-induced protein with tetratricopeptide repeats 5	IFIT5	1,08
potassium channel, voltage gated subfamily E regulatory beta subunit 1	KCNE1	1,08
long intergenic non-protein coding RNA 452	LINC00452	1,08
zinc finger protein 32	ZNF32	1,08
nitric oxide synthase 1 (neuronal)	NOS1	1,08
GTF2H2 family member C, copy 2; GTF2H2 family member C; general transcription factor IIH subunit 2B (pseudogene)	GTF2H2C_2; GTF2H2C; GTF2H2B	1,08
zinc finger protein 576	ZNF576	1,08
microRNA 5195; immunoglobulin heavy variable 5-78 (pseudogene)	MIR5195; IGHV5-78	1,08
clavesin 2	CLVS2	1,08
calcium homeostasis modulator 3	CALHM3	1,08
coiled-coil domain containing 88C	CCDC88C	1,08

dystrobrevin, alpha	DTNA	1,08
SAM pointed domain containing ETS transcription factor	SPDEF	1,08
basic leucine zipper transcription factor, ATF-like 2	BATF2	1,08
tumor necrosis factor receptor superfamily, member 4	TNFRSF4	1,08
adherens junctions associated protein 1	AJAP1	1,08
chitinase domain containing 1	CHID1	1,08
hydroxyacid oxidase 2 (long chain)	HAO2	1,08
endothelin converting enzyme 2	ECE2	1,08
NK2 homeobox 3	NKX2-3	1,08
proteasome (prosome, macropain) assembly chaperone 3	PSMG3	1,08
anoctamin 10	ANO10	1,08
long intergenic non-protein coding RNA 222	LINC00222	1,08
zinc finger, C2HC-type containing 1A	ZC2HC1A	1,08
Transcript Identified by AceView, Entrez Gene ID(s) 116154	PHACTR3	1,08
cytochrome P450, family 4, subfamily A, polypeptide 22	CYP4A22	1,08
histone deacetylase 8	HDAC8	1,08
macrophage scavenger receptor 1	MSR1	1,08
RAS p21 protein activator 4	RASA4	1,08
Ras suppressor protein 1	RSU1	1,08
transmembrane protein 14A	TMEM14A	1,08
angiopoietin 4	ANGPT4	1,08
heat shock protein 70kDa family, member 13	HSPA13	1,08
LY6/PLAUR domain containing 5	LYPD5	1,08
family with sequence similarity 166, member B	FAM166B	1,08
ubiquitin D	UBD	1,08
lumican	LUM	1,08
metastasis associated 1 family member 3	MTA3	1,08
tetratricopeptide repeat domain 23-like	TTC23L	1,08
AE binding protein 2	AEBP2	1,08
proteasome subunit alpha 5	PSMA5	1,08
T-box 21	TBX21	1,08
Memczak2013 ALT_ACCEPTOR, ALT_DONOR, coding, INTERNAL, intronic best transcript NM_016836	RBMS1	1,08
olfactory receptor, family 10, subfamily H, member 3	OR10H3	1,08
phosphodiesterase 3B, cGMP-inhibited	PDE3B	1,08
LanC lantibiotic synthetase component C-like 3 (bacterial)	LANCL3	1,08
EYA transcriptional coactivator and phosphatase 3	EYA3	1,08
Homo sapiens LSM12 homolog (S. cerevisiae), mRNA (cDNA clone MGC:57206 IMAGE:4794614), complete cds.	LSM12	1,08
	AC208162.1	1,08
Zic family member 5	ZIC5	1,08
glycogen synthase kinase 3 alpha	GSK3A	1,08
shisa family member 5	SHISA5	1,08
adenylate cyclase 9	ADCY9	1,08
C2 calcium-dependent domain containing 5	C2CD5	1,08

angiopoietin like 7	ANGPTL7	1,08
S100 calcium binding protein A6	S100A6	1,08
programmed cell death 2	PDCD2	1,08
gap junction protein beta 6	GJB6	1,08
ATPase, H+ transporting, lysosomal 70kDa, V1 subunit A	ATP6V1A	1,08
F-box protein 15	FBXO15	1,08
alpha- and gamma-adaptin binding protein	AAGAB	1,08
guanylate cyclase 1, soluble, beta 3	GUCY1B3	1,08
TNF receptor-associated factor 1	TRAF1	1,08
ring finger protein 181	RNF181	1,08
histone cluster 4, H4	HIST4H4	1,08
family with sequence similarity 200, member A	FAM200A	1,08
ankyrin repeat domain 61	ANKRD61	1,08
microtubule associated protein 1B	MAP1B	1,08
TATA box binding protein associated factor 15	TAF15	1,08
tRNA methyltransferase O	TRMO	1,08
leucine rich repeat containing 2	LRRC2	1,08
netrin G2	NTNG2	1,08
serine peptidase inhibitor, Kazal type 6	SPINK6	1,08
testis expressed 101	TEX101	1,08
grainyhead-like transcription factor 2	GRHL2	1,08
RNA binding motif protein 48	RBM48	1,08
histone deacetylase 10	HDAC10	1,08
UDP-glucose ceramide glucosyltransferase	UGCG	1,08
methylenetetrahydrofolate reductase (NAD(P)H)	MTHFR	1,08
claudin 11	CLDN11	1,08
glycogen synthase 2 (liver)	GYS2	1,08
leucine rich repeat and fibronectin type III domain containing 2	LRFN2	1,08
coiled-coil domain containing 163, pseudogene	CCDC163P	1,08
COBW domain containing 6; COBW domain containing 5; COBW domain containing 7	CBWD6; CBWD5; CBWD7	1,08
synovial sarcoma, X breakpoint 1	SSX1	1,08
olfactory receptor, family 5, subfamily G, member 5 pseudogene; olfactory receptor, family 5, subfamily G, member 3 (gene/pseudogene)	OR5G5P; OR5G3	1,08
PPARGC1 and ESRR induced regulator, muscle 1	PERM1	1,08
zinc finger protein 501 [Source:HGNC Symbol;Acc:HGNC:23717]	ZNF501	1,08
ubiquitin specific peptidase 17-like family member 27; ubiquitin specific peptidase 17-like family member 28	USP17L27; USP17L28	1,08
ubiquitin specific peptidase 17-like family member 28; ubiquitin specific peptidase 17-like family member 30	USP17L28; USP17L30	1,08
ubiquitin specific peptidase 17-like family member 29; ubiquitin specific peptidase 17-like family member 5	USP17L29; USP17L5	1,08

ubiquitin specific peptidase 17-like family member 25; ubiquitin specific peptidase 17-like family member 30	USP17L25; USP17L30	1,08
zinc finger protein 197	ZNF197	1,08
proprotein convertase subtilisin/kexin type 5	PCSK5	1,08
Salzman2013 ALT_ACCEPTOR, ALT_DONOR, coding, INTERNAL, intronic best transcript NM_006989	RASA4	1,08
LGALS8 antisense RNA 1	LGALS8-AS1	1,08
zinc finger protein 69	ZNF69	1,08
E2F transcription factor 2	E2F2	1,08
chromosome 5 open reading frame 30	C5orf30	1,08
ZFP42 zinc finger protein	ZFP42	1,08
kinesin family member 20A	KIF20A	1,08
alkB homolog 3, alpha-ketoglutarate-dependent dioxygenase; SEC14-like 1 pseudogene 1	ALKBH3; SEC14L1P1	1,08
elongation factor Tu GTP binding domain containing 1	EFTUD1	1,08
diacylglycerol kinase, delta 130kDa	DGKD	1,08
K(lysine) acetyltransferase 6B	KAT6B	1,08
coiled-coil domain containing 13	CCDC13	1,08
metastasis associated 1	MTA1	1,08
cell division cycle 20B	CDC20B	1,08
PRAME family member 8	PRAMEF8	1,08
RAB40B, member RAS oncogene family	RAB40B	1,08
family with sequence similarity 47, member C	FAM47C	1,08
protein phosphatase 1, regulatory subunit 12B	PPP1R12B	1,08
checkpoint with forkhead and ring finger domains, E3 ubiquitin protein ligase	CHFR	1,08
abl-interactor 2	ABI2	1,08
cyclin-dependent kinase 9	CDK9	1,08
STARD3 N-terminal like	STARD3NL	1,08
Fc fragment of IgE, high affinity I, receptor for; gamma polypeptide	FCER1G	1,08
zinc finger protein 611	ZNF611	1,08
Memczak2013 ANTISENSE, coding, INTERNAL, intronic best transcript NM_001001660	LYRM5	1,08
Transcript Identified by AceView, Entrez Gene ID(s) 389362	PSMG4	1,08
zinc finger protein 440	ZNF440	1,08
lipase, member H	LIPH	1,08
1-aminocyclopropane-1-carboxylate synthase (inactive)-like	ACCSL	1,08
Transcript Identified by AceView, Entrez Gene ID(s) 130888	FBXO36	1,08
adaptor-related protein complex 5, zeta 1 subunit	AP5Z1	1,08
hyaluronoglucosaminidase 4	HYAL4	1,08
NLR family, pyrin domain containing 11	NLRP11	1,08
protein phosphatase 5, catalytic subunit	PPP5C	1,08
tRNA methyltransferase 13 homolog (S. cerevisiae)	TRMT13	1,08
tryptophan 2,3-dioxygenase	TDO2	1,08

CD99 molecule-like 2	CD99L2	1,08
glutamate-rich WD repeat containing 1	GRWD1	1,08
regenerating islet-derived 3 alpha	REG3A	1,08
chemokine (C-C motif) ligand 3-like 3; chemokine (C-C motif) ligand 3-like 1	CCL3L3; CCL3L1	1,08
gamma-glutamyl hydrolase (conjugase, folylpolygammaglutamyl hydrolase)	GGH	1,08
forkhead-associated (FHA) phosphopeptide binding domain 1	FHAD1	1,08
SET domain containing 1A	SETD1A	1,08
transmembrane and coiled-coil domains 3	TMCO3	1,08
chromosome 7 open reading frame 66	C7orf66	1,08
transmembrane protein 75	TMEM75	1,08
G protein-coupled receptor 171	GPR171	1,08
odontogenic, ameloblast asssociated	ODAM	1,08
macrophage stimulating 1 receptor	MST1R	1,08
folliculin	FLCN	1,08
hematopoietic SH2 domain containing	HSH2D	1,08
HEN1 methyltransferase homolog 1 (Arabidopsis)	HENMT1	1,08
teneurin transmembrane protein 3	TENM3	1,08
mir-99a-let-7c cluster host gene	MIR99AHG	1,08
keratin associated protein 3-2	KRTAP3-2	1,08
zinc finger protein 354C	ZNF354C	1,08
Abelson helper integration site 1	AHI1	1,08
autophagy related 16-like 1	ATG16L1	1,08
calcium/calmodulin-dependent serine protein kinase (MAGUK family)	CASK	1,08
intraflagellar transport 22	IFT22	1,08
spermatogenesis associated, serine-rich 2-like	SPATS2L	1,08
glutamyl-tRNA synthetase 2, mitochondrial	EARS2	1,08
chondroitin sulfate proteoglycan 4	CSPG4	1,08
BVES antisense RNA 1	BVES-AS1	1,08
acid sensing ion channel family member 5	ASIC5	1,08
keratin 5, type II	KRT5	1,08
sulfotransferase family 1B member 1	SULT1B1	1,08
kinesin light chain 4	KLC4	1,08
gamma-aminobutyric acid (GABA) A receptor, alpha 4	GABRA4	1,08
PDS5 cohesin associated factor A	PDS5A	1,08
dynein, axonemal, heavy chain 7	DNAH7	1,08
SWIM-type zinc finger 7 associated protein 1	SWSAP1	1,08
zinc finger and AT hook domain containing	ZFAT	1,08
tubulin, beta 8 class VIII	TUBB8	1,08
thyroid hormone receptor interactor 10	TRIP10	1,08
aminopeptidase puromycin sensitive	NPEPPS	1,08
zinc finger protein 10	ZNF10	1,08
aspartate beta-hydroxylase	ASPH	1,08

TBC1 domain family, member 22B	TBC1D22B	1,08
RuvB-like AAA ATPase 1	RUVBL1	1,08
neuroblastoma breakpoint family, member 19	NBPF19	1,08
transmembrane protein 160	TMEM160	1,08
PR domain containing 12	PRDM12	1,08
uncharacterized LOC730183; Transcript Identified by AceView; novel transcript, antisense to SRCAP	LOC730183; RP11-146F11.1; hitema	1,08
aspartylglucosaminidase	AGA	1,08
Transcript Identified by AceView, Entrez Gene ID(s) 3561; putative novel transcript	RP5-1091N2.9; IL2RG	1,08
PHD finger protein 13	PHF13	1,08
zinc finger protein 574	ZNF574	1,08
vitronectin	VTN	1,08
selenophosphate synthetase 2	SEPHS2	1,08
ninjurin 1	NINJ1	1,08
bestrophin 1	BEST1	1,08
tripartite motif containing 5	TRIM5	1,08
uncharacterized LOC100130705; putative novel transcript	LOC100130705; RP11-309L24.4	1,08
eva-1 homolog A (C. elegans)	EVA1A	1,08
interleukin 9	IL9	1,08
Memczak2013 ALT_ACCEPTOR, ALT_DONOR, coding, INTERNAL, intronic best transcript NM_017931	TTC38	1,08
TGFB-induced factor homeobox 2-like, Y-linked	TGIF2LY	1,08
protein tyrosine phosphatase type IVA, member 1	PTP4A1	1,08
toll-like receptor 3	TLR3	1,08
cholinergic receptor, nicotinic beta 3	CHRNB3	1,08
myosin light chain, phosphorylatable, fast skeletal muscle	MYLPF	1,08
trypsin domain containing 1	TYSND1	1,08
chromosome 4 open reading frame 45	C4orf45	1,08
thymic stromal lymphopoietin	TSLP	1,08
JRK-like	JRKL	1,08
RAN binding protein 10	RANBP10	1,08
stimulated by retinoic acid 6	STRA6	1,08
thioredoxin-related transmembrane protein 2; chromosome 11 open reading frame 31	TMX2; C11orf31	1,08
transmembrane protein 9	TMEM9	1,08
THO complex 7	THOC7	1,08
microsomal glutathione S-transferase 3	MGST3	1,08
NADH dehydrogenase (ubiquinone) 1 alpha subcomplex, 4-like 2	NDUFA4L2	1,08
transmembrane channel like 7	TMC7	1,08
vacuolar protein sorting 8 homolog (S. cerevisiae)	VPS8	1,08

polymerase (DNA-directed), epsilon 4, accessory subunit	POLE4	1,08
RNA binding protein S1, serine-rich domain	RNPS1	1,08
Transcript Identified by AceView, Entrez Gene ID(s) 55619	DOCK10	1,08
KN motif and ankyrin repeat domains 4	KANK4	1,08
polymerase (RNA) I polypeptide A	POLR1A	1,08
Transcript Identified by AceView, Entrez Gene ID(s) 150590; 51601; 51263; chromosome 2 open reading frame 15 [Source:HGNC Symbol;Acc:HGNC:28436]	C2orf15andLIPT1andMRPL30; C2orf15	1,08
MLX interacting protein	MLXIP	1,08
nuclear pore complex interacting protein family, member A7	NPIPA7	1,08
transforming growth factor beta receptor associated protein 1	TGFBRAP1	1,08
interferon, alpha 8	IFNA8	1,08
dual specificity phosphatase 16	DUSP16	1,08
endogenous retrovirus group 3, member 1; zinc finger protein 117	ERV3-1; ZNF117	1,08
HECT and RLD domain containing E3 ubiquitin protein ligase family member 1	HERC1	1,08
SH3-domain GRB2-like endophilin B1	SH3GLB1	1,08
zinc finger protein 155	ZNF155	1,08
ectodysplasin A2 receptor	EDA2R	1,08
tight junction associated protein 1 (peripheral)	TJAP1	1,08
URI1, prefoldin-like chaperone	URI1	1,08
neuronal PAS domain protein 3	NPAS3	1,08
caspase 8, apoptosis-related cysteine peptidase	CASP8	1,08
zinc finger, DHHC-type containing 3	ZDHHC3	1,08
peroxiredoxin 6	PRDX6	1,08
ubiquitin associated protein 2; small nucleolar RNA, C/D box 121A; small nucleolar RNA, C/D box 121B	UBAP2; SNORD121A; SNORD121B	1,08
poly (ADP-ribose) glycohydrolase	PARG	1,08
guanylate cyclase 1, soluble, alpha 3	GUCY1A3	1,08
bolA family member 3	BOLA3	1,08
Transcript Identified by AceView, Entrez Gene ID(s) 27086; novel transcript, sense intronic to FOXP1	FOXP1; RP11-298C2.1	1,08
Rho GTPase activating protein 17	ARHGAP17	1,08
small nuclear ribonucleoprotein D2 polypeptide	SNRPD2	1,08
phosphatidylserine decarboxylase; microRNA 7109	PISD; MIR7109	1,08
zinc finger, CCHC domain containing 6	ZCCHC6	1,08
myeloid-associated differentiation marker-like 2	MYADML2	1,08
nucleoporin 50kDa	NUP50	1,08
BMP and activin membrane-bound inhibitor	BAMBI	1,08
Memczak2013 ALT_ACCEPTOR, ALT_DONOR, coding, INTERNAL, intronic best transcript NM_003036; Transcript Identified by AceView	SKI; kerlabu	1,08
ribosomal protein S6 kinase, 70kDa, polypeptide 2	RPS6KB2	1,08
complement factor I	CFI	1,08

complement component 1, q subcomponent binding protein	C1QBP	1,08
myoglobin	MB	1,08
NDRG family member 3	NDRG3	1,08
lysine (K)-specific demethylase 4E	KDM4E	1,08
serrate, RNA effector molecule	SRRT	1,08
Memczak2013 ALT_ACCEPTOR, ALT_DONOR, coding, INTERNAL, intronic best transcript NM_000969	RPL5	1,08
phosphatidylserine synthase 2	PTDSS2	1,08
splA/ryanodine receptor domain and SOCS box containing 2	SPSB2	1,08
fizzy/cell division cycle 20 related 1	FZR1	1,08
ubiquitin associated and SH3 domain containing A	UBASH3A	1,08
pumilio RNA binding family member 3	PUM3	1,08
monocyte to macrophage differentiation-associated 2	MMD2	1,08
ankyrin repeat domain 6	ANKRD6	1,08
solute carrier family 2 (facilitated glucose transporter), member 8	SLC2A8	1,08
choroideremia-like (Rab escort protein 2)	CHML	1,08
NK2 homeobox 8	NKX2-8	1,08
proteasome subunit beta 1	PSMB1	1,08
atonal bHLH transcription factor 8	ATOH8	1,08
ubiquitin B	UBB	1,08
ZFP69 zinc finger protein	ZFP69	1,08
limb and CNS expressed 1	LIX1	1,08
inner mitochondrial membrane peptidase subunit 1	IMMP1L	1,08
pirin	PIR	1,08
acetyl-CoA acetyltransferase 1	ACAT1	1,08
nuclear receptor subfamily 1, group H, member 4	NR1H4	1,08
makorin ring finger protein 1	MKRN1	1,08
3-hydroxyisobutyrate dehydrogenase	HIBADH	1,08
ribosomal protein L22	RPL22	1,08
transcription elongation factor A (SII)-like 2	TCEAL2	1,08
carbonic anhydrase XIV	CA14	1,08
BIVM-ERCC5 readthrough	BIVM-ERCC5	1,08
solute carrier family 7 (cationic amino acid transporter, y+ system), member 1	SLC7A1	1,08
small leucine-rich protein 1	SMLR1	1,08
cell division cycle 20	CDC20	1,08
Transcript Identified by AceView, Entrez Gene ID(s) 1803	DPP4	1,08
chromosome 22 open reading frame 15	C22orf15	1,08
C-type lectin domain family 2, member D	CLEC2D	1,08
SIK family kinase 3	SIK3	1,08
origin recognition complex subunit 4	ORC4	1,08
gasdermin C	GSDMC	1,08
cyclin J-like	CCNJL	1,08
family with sequence similarity 196, member B	FAM196B	1,08

SET binding protein 1	SETBP1	1,08
osteoclast associated, immunoglobulin-like receptor	OSCAR	1,08
C-reactive protein, pentraxin-related	CRP	1,08
olfactory receptor, family 8, subfamily S, member 1	OR8S1	1,08
translocase of outer mitochondrial membrane 20 homolog (yeast)-like	TOMM20L	1,08
nitrilase 1	NIT1	1,08
polymerase (RNA) III (DNA directed) polypeptide F, 39 kDa	POLR3F	1,08
2-oxoglutarate and iron-dependent oxygenase domain containing 3	OGFOD3	1,08
ribosomal RNA processing 1B	RRP1B	1,08
phosphohistidine phosphatase 1	PHPT1	1,08
ribosome production factor 2 homolog	RPF2	1,08
neuropeptides B/W receptor 2	NPBWR2	1,08
chromosome 3 open reading frame 62	C3orf62	1,08
ribosomal protein S6 kinase, 90kDa, polypeptide 1	RPS6KA1	1,08
interferon, alpha 2	IFNA2	1,08
male-specific lethal 2 homolog (Drosophila)	MSL2	1,08
kelch-like family member 15	KLHL15	1,08
proline rich 29	PRR29	1,08
collagen, type IX, alpha 2	COL9A2	1,08
coiled-coil domain containing 69	CCDC69	1,08
spastic paraplegia 7 (pure and complicated autosomal recessive)	SPG7	1,08
mesogenin 1	MSGN1	1,08
olfactory receptor, family 1, subfamily I, member 1	OR1I1	1,08
RNA binding motif protein 17	RBM17	1,08
ATPase, Ca++ transporting, plasma membrane 1	ATP2B1	1,08
surfactant associated 3	SFTA3	1,08
RAD54-like 2 (S. cerevisiae)	RAD54L2	1,08
Memczak2013 ANTISENSE, coding, INTERNAL, intronic best transcript NM_002072; Transcript Identified by AceView	GNAQ; skeyplorbu	1,08
Homo sapiens family with sequence similarity 103, member A1, mRNA (cDNA clone MGC:2560 IMAGE:2989772), complete cds.; Homo sapiens family with sequence similarity 103, member A1, mRNA (cDNA clone MGC:18029 IMAGE:3924570), complete cds.; Homo sapiens family with sequence similarity 103, member A1, mRNA (cDNA clone MGC:102778 IMAGE:5578103), complete cds.	FAM103A1	1,08
family with sequence similarity 171, member A2	FAM171A2	1,08
transmembrane protease, serine 3	TMPRSS3	1,08
GDNF family receptor alpha 3	GFRA3	1,08
taste receptor, type 2, member 16	TAS2R16	1,08
Memczak2013 ANTISENSE, CDS, coding, INTERNAL best transcript NM_017607	PPP1R12C	1,08

integral membrane protein 2B	ITM2B	1,08
Jeck2013 ANTISENSE, CDS, coding, INTERNAL, OVCODE, OVEXON, UTR3 best transcript NM_001013	RPS9	1,08
ceroid-lipofuscinosis, neuronal 8	CLN8	1,08
Memczak2013 ANTISENSE, CDS, coding, INTERNAL best transcript NM_001184717	TIPARP	1,08
post-GPI attachment to proteins 3	PGAP3	1,08
claudin 17	CLDN17	1,08
NTPase, KAP family P-loop domain containing 1	NKPD1	1,08
MLX interacting protein	MLXIP	1,08
AT hook, DNA binding motif, containing 1	AHDC1	1,08
gap junction protein alpha 10	GJA10	1,07
tetratricopeptide repeat domain 5	TTC5	1,07
chromosome 21 open reading frame 33	C21orf33	1,07
mesoderm development candidate 1	MESDC1	1,07
leucine-rich pentatricopeptide repeat containing	LRPPRC	1,07
Memczak2013 ANTISENSE, CDS, coding, INTERNAL, intronic best transcript NM_178860	SEZ6	1,07
SHC SH2-domain binding protein 1	SHCBP1	1,07
paraoxonase 2	PON2	1,07
zinc finger protein 200	ZNF200	1,07
forkhead box P2	FOXP2	1,07
Transcript Identified by AceView, Entrez Gene ID(s) 57504	MTA3	1,07
glutamate dehydrogenase 2	GLUD2	1,07
peroxisomal biogenesis factor 5	PEX5	1,07
Era-like 12S mitochondrial rRNA chaperone 1	ERAL1	1,07
neurexophilin and PC-esterase domain family, member 1	NXPE1	1,07
desumoylating isopeptidase 2	DESI2	1,07
uncharacterized LOC283710; novel transcript; Transcript Identified by AceView	LOC283710; RP11-16E12.2; rukama	1,07
BMP2 inducible kinase	BMP2K	1,07
ataxin 10	ATXN10	1,07
HECT and RLD domain containing E3 ubiquitin protein ligase 3	HERC3	1,07
peptidase M20 domain containing 1	PM20D1	1,07
Salzman2013 ANNOTATED, coding, OVEXON, UTR5 best transcript NM_001005862	ERBB2	1,07
angiomotin like 1	AMOTL1	1,07
jade family PHD finger 3	JADE3	1,07
zinc finger protein 142	ZNF142	1,07
transmembrane protein 210	TMEM210	1,07
zinc finger protein 280A	ZNF280A	1,07
par-6 family cell polarity regulator gamma	PARD6G	1,07
fibrillarin	FBL	1,07
chromosome 2 open reading frame 54	C2orf54	1,07

olfactory receptor, family 2, subfamily T, member 5	OR2T5	1,07
chromosome 11 open reading frame 44	C11orf44	1,07
family with sequence similarity 181, member A	FAM181A	1,07
anoctamin 5	ANO5	1,07
prolyl 3-hydroxylase 1	P3H1	1,07
cadherin-related family member 3	CDHR3	1,07
beta-1,3-glucuronyltransferase 3	B3GAT3	1,07
tyrosine 3-monooxygenase/tryptophan 5-monooxygenase activation protein, theta	YWHAQ	1,07
chromosome 5 open reading frame 63	C5orf63	1,07
frizzled class receptor 8; microRNA 4683	FZD8; MIR4683	1,07
tubulin polymerization-promoting protein family member 3	TPPP3	1,07
zinc finger protein 283	ZNF283	1,07
MLX interacting protein	MLXIP	1,07
zinc finger protein 469	ZNF469	1,07
family with sequence similarity 151, member B	FAM151B	1,07
guanine nucleotide binding protein (G protein), alpha transducing activity polypeptide 1	GNAT1	1,07
DEAD (Asp-Glu-Ala-Asp) box polypeptide 54	DDX54	1,07
butyrophilin, subfamily 2, member A3, pseudogene	BTN2A3P	1,07
serine/threonine kinase 3	STK3	1,07
NOP2/Sun domain family, member 5 pseudogene 1	NSUN5P1	1,07
SET domain containing 2	SETD2	1,07
fibronectin leucine rich transmembrane protein 1	FLRT1	1,07
leucine-rich repeats and calponin homology (CH) domain containing 4	LRCH4	1,07
BIN3 intronic transcript 1	BIN3-IT1	1,07
mucin 16, cell surface associated	MUC16	1,07
ariadne RBR E3 ubiquitin protein ligase 2	ARIH2	1,07
phosphatidylinositol transfer protein, alpha	PITPNA	1,07
spermatogenesis associated 17	SPATA17	1,07
uncharacterized LOC401052; Salzman2013 ANNOTATED, coding, INTERNAL, OVERLAPTX, OVEXON, UTR5 best transcript NM_001008737; novel transcript; Transcript Identified by AceView, Entrez Gene ID(s) 401052; 55831	LOC401052; AC022007.5; TMEM111	1,07
kinesin family member 4A	KIF4A	1,07
xeroderma pigmentosum, complementation group A	XPA	1,07
mucin 5AC, oligomeric mucus/gel-forming	MUC5AC	1,07
Pim-3 proto-oncogene, serine/threonine kinase	PIM3	1,07
Fas apoptotic inhibitory molecule	FAIM	1,07
C-type lectin domain family 3, member B	CLEC3B	1,07
CTTNBP2 N-terminal like	CTTNBP2NL	1,07
AF4/FMR2 family, member 4	AFF4	1,07
zinc finger, AN1-type domain 3	ZFAND3	1,07
piwi-like RNA-mediated gene silencing 3	PIWIL3	1,07

pecanex-like 3 (Drosophila)	PCNXL3	1,07
N-6 adenine-specific DNA methyltransferase 1 (putative)	N6AMT1	1,07
protein inhibitor of activated STAT 3	PIAS3	1,07
chromosome X open reading frame 67	CXorf67	1,07
coenzyme Q5, methyltransferase	COQ5	1,07
Transcript Identified by AceView, Entrez Gene ID(s) 64405	CDH22	1,07
repulsive guidance molecule family member b	RGMB	1,07
SHC (Src homology 2 domain containing) transforming protein 1	SHC1	1,07
mitochondrial fission regulator 2	MTFR2	1,07
double homeobox 3	DUX3	1,07
Nance-Horan syndrome (congenital cataracts and dental anomalies)	NHS	1,07
transmembrane protein 209	TMEM209	1,07
leucine-rich repeat LGI family, member 3	LGI3	1,07
adaptor-related protein complex 2, beta 1 subunit	AP2B1	1,07
Memczak2013 ALT_ACCEPTOR, ALT_DONOR, coding, INTERNAL, intronic best transcript NM_006224	PITPNA	1,07
PRAME family member 14	PRAMEF14	1,07
STARD7 antisense RNA 1	STARD7-AS1	1,07
DEAD (Asp-Glu-Ala-Asp) box polypeptide 49	DDX49	1,07
keratin associated protein 5-5	KRTAP5-5	1,07
leucine-rich repeats and calponin homology (CH) domain containing 3	LRCH3	1,07
Transcript Identified by AceView, Entrez Gene ID(s) 5825	ABCD3	1,07
complement factor H-related 5	CFHR5	1,07
katanin p80 subunit B-like 1	KATNBL1	1,07
cAMP responsive element binding protein 3-like 1	CREB3L1	1,07
glutaredoxin 3	GLRX3	1,07
tectorin beta	TECTB	1,07
Transcript Identified by AceView, Entrez Gene ID(s) 288	ANK3	1,07
family with sequence similarity 134, member C	FAM134C	1,07
zinc finger protein 845	ZNF845	1,07
inositol 1,4,5-trisphosphate receptor interacting protein-like 1	ITPRIPL1	1,07
zinc finger, SWIM-type containing 7	ZSWIM7	1,07
Memczak2013 ALT_ACCEPTOR, ALT_DONOR, coding, INTERNAL, intronic best transcript NM_001012478	U2AF2	1,07
glutamate rich 1	ERICH1	1,07
Transcript Identified by AceView, Entrez Gene ID(s) 63898	SH2D4A	1,07
ROS proto-oncogene 1 , receptor tyrosine kinase	ROS1	1,07
zinc finger protein 780A	ZNF780A	1,07
zinc finger protein 460	ZNF460	1,07
NFU1 iron-sulfur cluster scaffold	NFU1	1,07
interferon-induced protein 44	IFI44	1,07
SPT7-like STAGA complex gamma subunit	SUPT7L	1,07

chromosome X open reading frame 51A; chromosome X open reading frame 51B	CXorf51A; CXorf51B	1,07
tumor necrosis factor, alpha-induced protein 8-like 3	TNFAIP8L3	1,07
GLI family zinc finger 2	GLI2	1,07
RAS-like, family 10, member A	RASL10A	1,07
spermatid associated	SPERT	1,07
lactate dehydrogenase C	LDHC	1,07
FBJ murine osteosarcoma viral oncogene homolog B	FOSB	1,07
chromosome 1 open reading frame 52	C1orf52	1,07
pleckstrin homology domain containing, family H (with MyTH4 domain) member 2	PLEKHH2	1,07
autophagy related 16-like 1	ATG16L1	1,07
SPANX family, member D	SPANXD	1,07
solute carrier family 52 (riboflavin transporter), member 3	SLC52A3	1,07
basic transcription factor 3-like 4	BTF3L4	1,07
mitochondrial assembly of ribosomal large subunit 1	MALSU1	1,07
lipoyl(octanoyl) transferase 2 (putative)	LIPT2	1,07
ubiquilin 1	UBQLN1	1,07
CD160 molecule	CD160	1,07
mitochondrial ribosomal protein S11	MRPS11	1,07
minichromosome maintenance complex component 3 associated protein	MCM3AP	1,07
prion protein	PRNP	1,07
nuclear receptor 2C2-associated protein	NR2C2AP	1,07
serpin peptidase inhibitor, clade A (alpha-1 antiproteinase, antitrypsin), member 2 (gene/pseudogene)	SERPINA2	1,07
ATPase, Cu++ transporting, beta polypeptide	ATP7B	1,07
zinc finger and BTB domain containing 7C	ZBTB7C	1,07
estrogen-related receptor gamma	ESRRG	1,07
proteasome 26S subunit, non-ATPase 6	PSMD6	1,07
ADAMTSL4 antisense RNA 1	ADAMTSL4-AS1	1,07
rhophilin associated tail protein 1B	ROPN1B	1,07
Kv channel interacting protein 4	KCNIP4	1,07
adhesion G protein-coupled receptor B1	ADGRB1	1,07
YKT6 v-SNARE homolog (S. cerevisiae)	YKT6	1,07
ZFP82 zinc finger protein	ZFP82	1,07
diacylglycerol lipase, alpha	DAGLA	1,07
testis specific protein, Y-linked 3	TSPY3	1,07
zinc finger protein 833, pseudogene	ZNF833P	1,07
amyloid beta (A4) precursor protein-binding, family B, member 3	APBB3	1,07
rhophilin associated tail protein 1	ROPN1	1,07
chorionic gonadotropin, beta polypeptide 1	CGB1	1,07
ATP binding cassette subfamily F member 1	ABCF1	1,07

sulfotransferase family 1A member 4; sulfotransferase family 1A member 3	SULT1A4; SULT1A3	1,07
potassium channel, voltage gated subfamily E regulatory beta subunit 1	KCNE1	1,07
collapsin response mediator protein 1	CRMP1	1,07
opioid receptor, kappa 1	OPRK1	1,07
cytochrome c oxidase subunit VIIb	COX7B	1,07
UDP-glucose pyrophosphorylase 2	UGP2	1,07
A kinase (PRKA) anchor protein 12	AKAP12	1,07
transmembrane protein 169	TMEM169	1,07
zinc finger protein 592	ZNF592	1,07
dentin matrix acidic phosphoprotein 1	DMP1	1,07
SRY box 10	SOX10	1,07
Memczak2013 ANTISENSE, coding, INTERNAL, intronic best transcript NM_001165899	PDE4D	1,07
mucin 15, cell surface associated	MUC15	1,07
paired-like homeobox 2a	PHOX2A	1,07
BTB (POZ) domain containing 3	BTBD3	1,07
spermatid maturation 1	SPEM1	1,07
zinc finger protein 780A	ZNF780A	1,07
potassium channel, voltage gated modifier subfamily G, member 4	KCNG4	1,07
interleukin 1 receptor associated kinase 4	IRAK4	1,07
enoyl-CoA hydratase domain containing 2	ECHDC2	1,07
chromosome 4 open reading frame 26	C4orf26	1,07
chromosome 8 open reading frame 44	C8orf44	1,07
zinc finger and SCAN domain containing 20	ZSCAN20	1,07
ribonuclease, RNase A family, 3	RNASE3	1,07
phosphomevalonate kinase	PMVK	1,07
interferon regulatory factor 3	IRF3	1,07
family with sequence similarity 135, member B	FAM135B	1,07
myeloid/lymphoid or mixed-lineage leukemia; translocated to, 10	MLLT10	1,07
beaded filament structural protein 2, phakinin	BFSP2	1,07
zinc finger protein 814	ZNF814	1,07
presenilin enhancer gamma secretase subunit	PSENEN	1,07
diacylglycerol kinase, eta	DGKH	1,07
calcium binding and coiled-coil domain 2	CALCOCO2	1,07
heterogeneous nuclear ribonucleoprotein M	HNRNPM	1,07
potassium channel, voltage gated shaker related subfamily A, member 1	KCNA1	1,07
zinc finger protein 750	ZNF750	1,07
olfactory receptor, family 5, subfamily K, member 1	OR5K1	1,07
cat eye syndrome chromosome region, candidate 6	CECR6	1,07
keratin 84, type II	KRT84	1,07

tRNA methyltransferase 10C, mitochondrial RNase P subunit	TRMT10C	1,07
uncharacterized LOC100287728; Transcript Identified by AceView; novel transcript	LOC100287728; RP11-85L21.4; jerva	1,07
Transcript Identified by AceView, Entrez Gene ID(s) 4114	MAGEB3	1,07
transcription elongation factor B polypeptide 3C-like	TCEB3CL	1,07
olfactory receptor, family 5, subfamily P, member 3	OR5P3	1,07
adaptor-related protein complex 3, sigma 2 subunit; microRNA 5009	AP3S2; MIR5009	1,07
purinergic receptor P2X, ligand gated ion channel, 4	P2RX4	1,07
ras responsive element binding protein 1	RREB1	1,07
phosphoinositide-3-kinase, regulatory subunit 2 (beta); interferon, gamma-inducible protein 30	PIK3R2; IFI30	1,07
nitric oxide synthase interacting protein	NOSIP	1,07
interleukin 12 receptor, beta 2	IL12RB2	1,07
phenylalanine hydroxylase	PAH	1,07
trichohyalin like 1	TCHHL1	1,07
neurocalcin delta	NCALD	1,07
lipocalin 9	LCN9	1,07
complement component 1, q subcomponent-like 2	C1QL2	1,07
leucine-rich repeats and IQ motif containing 1	LRRIQ1	1,07
helt bHLH transcription factor	HELT	1,07
olfactory receptor, family 2, subfamily L, member 13	OR2L13	1,07
uncharacterized LOC401081; uncharacterized LOC401081 [Source:EntrezGene;Acc:401081]; novel transcript	FLJ22763; RP11-59E19.1	1,07
zinc finger protein 280D	ZNF280D	1,07
structure specific recognition protein 1	SSRP1	1,07
actin-related protein 2/3 complex inhibitor	ARPIN	1,07
visual system homeobox 2	VSX2	1,07
prefoldin subunit 2	PFDN2	1,07
heme oxygenase 2	HMOX2	1,07
LMBR1 domain containing 1	LMBRD1	1,07
late cornified envelope 1E	LCE1E	1,07
RUN domain containing 1	RUNDC1	1,07
calpastatin	CAST	1,07
contactin associated protein-like 2	CNTNAP2	1,07
testis specific protein, Y-linked 10	TSPY10	1,07
glycoprotein (transmembrane) nmb	GPNMB	1,07
MICAL C-terminal like	MICALCL	1,07
mitogen-activated protein kinase kinase 2	MAP2K2	1,07
mannosidase, endo-alpha	MANEA	1,07
developmentally regulated GTP binding protein 1	DRG1	1,07
ATP binding cassette subfamily A member 6	ABCA6	1,07
nitric oxide associated 1	NOA1	1,07

Salzman2013 ANNOTATED, CDS, coding, INTERNAL, OVCODE, OVEXON best transcript NM_001190810; centaurin, gamma-like family, member 11 pseudogene [Source:HGNC Symbol;Acc:HGNC:23660]	AGAP9; CTGLF11P	1,07
myeloma overexpressed	MYEOV	1,07
tumor necrosis factor (ligand) superfamily, member 12	TNFSF12	1,07
gamma-aminobutyric acid (GABA) A receptor, rho 1	GABRR1	1,07
ZNF529 antisense RNA 1	ZNF529-AS1	1,07
chromosome 19 open reading frame 70	C19orf70	1,07
ADIRF antisense RNA 1 [Source:HGNC Symbol;Acc:HGNC:45127]; Transcript Identified by AceView, Entrez Gene ID(s) 79812; ADIRF antisense RNA 1; novel transcript, antisense to ADIRF	ADIRF-AS1; RP11-96C23.15; MMRN2	1,07
leucine rich repeat transmembrane neuronal 4	LRRTM4	1,07
myopalladin	MYPN	1,07
zinc finger protein 484	ZNF484	1,07
CD2-associated protein	CD2AP	1,07
N(alpha)-acetyltransferase 35, NatC auxiliary subunit	NAA35	1,07
acid phosphatase, prostate	ACPP	1,07
RAB40A, member RAS oncogene family-like	RAB40AL	1,07
vomeronasal 1 receptor 5 (gene/pseudogene)	VN1R5	1,07
WD repeat domain 27	WDR27	1,07
interleukin 17A	IL17A	1,07
rhophilin, Rho GTPase binding protein 2	RHPN2	1,07
transient receptor potential cation channel, subfamily M, member 8	TRPM8	1,07
troponin C type 2 (fast)	TNNC2	1,07
staphylococcal nuclease and tudor domain containing 1; SND1 intronic transcript 1	SND1; SND1-IT1	1,07
solute carrier organic anion transporter family, member 3A1	SLCO3A1	1,07
microtubule associated protein 2	MAP2	1,07
cAMP responsive element modulator	CREM	1,07
ubiquitin associated protein 1	UBAP1	1,07
hydroxyacylglutathione hydrolase-like	HAGHL	1,07
glioma tumor suppressor candidate region gene 2; small nucleolar RNA, C/D box 23	GLTSCR2; SNORD23	1,07
paraneoplastic Ma antigen family-like 1	PNMAL1	1,07
v-maf avian musculoaponeurotic fibrosarcoma oncogene homolog A	MAFA	1,07
uncharacterized LOC55338; novel transcript	LOC55338; CTC-242N15.1	1,07
SGT1 homolog, MIS12 kinetochore complex assembly cochaperone	SUGT1	1,07
RGP1 homolog, RAB6A GEF complex partner 1	RGP1	1,07
proteasome 26S subunit, ATPase 5	PSMC5	1,07

zinc finger protein 44	ZNF44	1,07
diphosphoinositol pentakisphosphate kinase 1	PPIP5K1	1,07
inositol polyphosphate-5-phosphatase D	INPP5D	1,07
cut-like homeobox 1	CUX1	1,07
olfactory receptor, family 6, subfamily N, member 1	OR6N1	1,07
von Willebrand factor A domain containing 2	VWA2	1,07
proteasome 26S subunit, ATPase 2	PSMC2	1,07
chromosome 14 open reading frame 2	C14orf2	1,07
retinal degeneration 3-like	RD3L	1,07
uncharacterized LOC101929319; Salzman2013 ANTISENSE, CDS, coding, INTERNAL, intronic, OVCODE, OVEXON best transcript NM_007115	LOC101929319; TNFAIP6	1,07
potassium channel, voltage gated KQT-like subfamily Q, member 4	KCNQ4	1,07
ubiquitin conjugating enzyme E2G 2	UBE2G2	1,07
mitochondrial ribosomal protein S22	MRPS22	1,07
glutamine-fructose-6-phosphate transaminase 2	GFPT2	1,07
lysine (K)-specific methyltransferase 2D	KMT2D	1,07
cytochrome b5 reductase 1	CYB5R1	1,07
mago homolog B, exon junction complex core component	MAGOHB	1,07
major facilitator superfamily domain containing 1	MFSD1	1,07
polymerase (DNA directed), theta	POLQ	1,07
ALX homeobox 1	ALX1	1,07
olfactomedin 1	OLFM1	1,07
family with sequence similarity 175, member B	FAM175B	1,07
dynein, cytoplasmic 2, light intermediate chain 1	DYNC2LI1	1,07
olfactory receptor, family 6, subfamily A, member 2	OR6A2	1,07
coiled-coil-helix-coiled-coil-helix domain containing 2	CHCHD2	1,07
nuclear receptor subfamily 4, group A, member 2	NR4A2	1,07
musculoskeletal, embryonic nuclear protein 1	MUSTN1	1,07
interferon induced, with helicase C domain 1	IFIH1	1,07
autophagy related 16-like 1	ATG16L1	1,07
component of oligomeric golgi complex 3	COG3	1,07
dual specificity tyrosine-(Y)-phosphorylation regulated kinase 3	DYRK3	1,07
BLOC-1 related complex subunit 7	BORCS7	1,07
Rho guanine nucleotide exchange factor 26	ARHGEF26	1,07
retinoic acid receptor responder (tazarotene induced) 2	RARRES2	1,07
potassium channel, two pore domain subfamily K, member 5	KCNK5	1,07
oncoprotein induced transcript 3	OIT3	1,07
parathyroid hormone 2 receptor	PTH2R	1,07
aquaporin 5	AQP5	1,07
Memczak2013 ANTISENSE, coding, INTERNAL, intronic best transcript NM_014699	ZNF646	1,07
gem nuclear organelle associated protein 8	GEMIN8	1,07

Memczak2013 ANTISENSE, coding, INTERNAL, UTR3 best transcript NM_002567	PEBP1	1,07
transmembrane protein 199; microRNA 4723	TMEM199; MIR4723	1,07
sperm flagellar 2	SPEF2	1,07
ATP synthase, H+ transporting, mitochondrial F1 complex, beta polypeptide	ATP5B	1,07
olfactory receptor, family 1, subfamily P, member 1 (gene/pseudogene)	OR1P1	1,07
TERF1 (TRF1)-interacting nuclear factor 2	TINF2	1,07
zinc finger protein 84	ZNF84	1,07
mohawk homeobox	MKX	1,07
cochlin	COCH	1,07
cysteine-rich secretory protein LCCL domain containing 2	CRISPLD2	1,07
NADH dehydrogenase (ubiquinone) 1 alpha subcomplex, 10, 42kDa	NDUFA10	1,07
ubiquitin C	UBC	1,07
keratin 6B, type II	KRT6B	1,07
immunoglobulin superfamily, member 6	IGSF6	1,07
NLR family, pyrin domain containing 2	NLRP2	1,07
myotubularin related protein 8	MTMR8	1,07
S100 calcium binding protein A7 like 2	S100A7L2	1,07
FYVE, RhoGEF and PH domain containing 3	FGD3	1,07
G protein-coupled receptor 37 like 1	GPR37L1	1,07
succinate-CoA ligase, ADP-forming, beta subunit	SUCLA2	1,07
zinc finger protein 33A	ZNF33A	1,07
SRSF protein kinase 3	SRPK3	1,07
solute carrier family 26, member 10	SLC26A10	1,07
popeye domain containing 2	POPDC2	1,07
transmembrane and tetratricopeptide repeat containing 1	TMTC1	1,07
PIGB opposite strand 1; RAB27A, member RAS oncogene family	PIGBOS1; RAB27A	1,07
CSAG family, member 3	CSAG3	1,07
phospholipase C, delta 3	PLCD3	1,07
guanine nucleotide binding protein (G protein), alpha 15 (Gq class)	GNA15	1,07
STT3B, subunit of the oligosaccharyltransferase complex (catalytic)	STT3B	1,07
erb-b2 receptor tyrosine kinase 2	ERBB2	1,07
complement factor H-related 2	CFHR2	1,07
defensin, beta 132	DEFB132	1,07
casein kinase 1, alpha 1-like	CSNK1A1L	1,07
barttin CLCNK-type chloride channel accessory beta subunit	BSND	1,07
interleukin 25	IL25	1,07
cholinergic receptor, muscarinic 3	CHRM3	1,07

Rho GTPase activating protein 15	ARHGAP15	1,07
endosulfine alpha	ENSA	1,07
charged multivesicular body protein 4C	CHMP4C	1,07
solute carrier family 11 (proton-coupled divalent metal ion transporter), member 1	SLC11A1	1,07
chromosome 15 open reading frame 61	C15orf61	1,07
ceramide synthase 6	CERS6	1,07
DnaJ (Hsp40) homolog, subfamily C, member 5 beta	DNAJC5B	1,07
ubiquitin specific peptidase 5 (isopeptidase T)	USP5	1,07
interleukin 6 receptor	IL6R	1,07
regulator of microtubule dynamics 1	RMDN1	1,07
butyrophilin-like 8	BTNL8	1,07
zinc finger protein 512	ZNF512	1,07
serpin peptidase inhibitor, clade B (ovalbumin), member 10	SERPINB10	1,07
ALG14, UDP-N-acetylglucosaminyltransferase subunit	ALG14	1,07
NADH dehydrogenase (ubiquinone) 1 alpha subcomplex, 10, 42kDa	NDUFA10	1,07
PCI domain containing 2	PCID2	1,07
zinc finger protein 823	ZNF823	1,07
family with sequence similarity 171, member B	FAM171B	1,07
CASP8 and FADD like apoptosis regulator	CFLAR	1,07
transmembrane protein 239; chromosome 20 open reading frame 141	TMEM239; C20orf141	1,07
heterogeneous nuclear ribonucleoprotein H2 (H)	HNRNPH2	1,07
NCK-associated protein 1	NCKAP1	1,07
sperm adhesion molecule 1 (PH-20 hyaluronidase, zona pellucida binding)	SPAM1	1,07
TBC1 domain family, member 3L; TBC1 domain family, member 3E	TBC1D3L; TBC1D3E	1,07
ribosomal protein L7a; small nucleolar RNA, C/D box 36C; small nucleolar RNA, C/D box 36B; small nucleolar RNA, C/D box 24; small nucleolar RNA, C/D box 36A	RPL7A; SNORD36C; SNORD36B; SNORD24; SNORD36A	1,07
keratinocyte differentiation factor 1	KDF1	1,07
phospholipase A2, group XV	PLA2G15	1,07
Jeck2013 ALT_ACCEPTOR, ALT_DONOR, coding, INTERNAL, intronic, OVERLAPTX best transcript NM_024911	WLS	1,07
protein phosphatase 4, regulatory subunit 4	PPP4R4	1,07
chromosome 19 open reading frame 35	C19orf35	1,07
sarcoglycan epsilon	SGCE	1,07
chromosome 9 open reading frame 152	C9orf152	1,07
origin recognition complex subunit 2	ORC2	1,07
family with sequence similarity 214, member B	FAM214B	1,07
CPX chromosome region, candidate 1	CPXCR1	1,07

endoplasmic reticulum to nucleus signaling 1	ERN1	1,07
transmembrane protein 8A	TMEM8A	1,07
fuzzy planar cell polarity protein	FUZ	1,07
Jeck2013 ANTISENSE, coding, INTERNAL, intronic best transcript NM_001207014	SERPINH1	1,07
olfactory receptor, family 5, subfamily R, member 1 (gene/pseudogene)	OR5R1	1,07
zinc finger protein 396	ZNF396	1,07
U2 small nuclear RNA auxiliary factor 2	U2AF2	1,07
YTH domain containing 1	YTHDC1	1,07
uncharacterized LOC100129924; Transcript Identified by AceView, Entrez Gene ID(s) 79078; novel transcript	LOC100129924; C1orf50; RP5-994D16.11	1,07
brain expressed X-linked 4	BEX4	1,07
kinesin family member 7	KIF7	1,07
opioid receptor, mu 1	OPRM1	1,07
StAR-related lipid transfer domain containing 3	STARD3	1,07
transcription factor 7-like 1 (T-cell specific, HMG-box)	TCF7L1	1,07
proline rich 4 (lacrimal)	PRR4	1,07
solute carrier family 26 (anion exchanger), member 1	SLC26A1	1,07
acetoacetyl-CoA synthetase pseudogene 1	AACSP1	1,07
TSC22 domain family, member 2	TSC22D2	1,07
autophagy related 2A	ATG2A	1,07
heat shock 22kDa protein 8	HSPB8	1,07
ankyrin repeat, family A (RFXANK-like), 2	ANKRA2	1,07
Ets2 repressor factor	ERF	1,07
metallothionein 1E	MT1E	1,07
Sjogren syndrome antigen B (autoantigen La)	SSB	1,07
retinol dehydrogenase 8 (all-trans)	RDH8	1,07
fibroblast activation protein alpha	FAP	1,07
hematological and neurological expressed 1	HN1	1,07
axin interactor, dorsalization associated	AIDA	1,07
transcription elongation factor A (SII), 3	TCEA3	1,07
TAR (HIV-1) RNA binding protein 2	TARBP2	1,07
ATPase, H+ transporting, lysosomal 38kDa, V0 subunit d1	ATP6V0D1	1,07
E4F transcription factor 1	E4F1	1,07
N-acetylglucosamine-1-phosphate transferase, alpha and beta subunits	GNPTAB	1,07
glutamate receptor, ionotropic, N-methyl-D-aspartate 3A	GRIN3A	1,07
RAB GTPase activating protein 1-like	RABGAP1L	1,07
mitochondrial ribosomal protein L47	MRPL47	1,07
olfactory receptor, family 10, subfamily X, member 1 (gene/pseudogene)	OR10X1	1,07
thrombospondin 4	THBS4	1,07

amyloid beta (A4) precursor protein-binding, family B, member 2	APBB2	1,07
adhesion G protein-coupled receptor G2	ADGRG2	1,07
mitochondrial ribosomal protein L39	MRPL39	1,07
cyclin-dependent kinase 12	CDK12	1,07
secretoglobin, family 2A, member 1	SCGB2A1	1,07
Spi-C transcription factor (Spi-1/PU.1 related)	SPIC	1,07
regulatory factor X, 6	RFX6	1,07
cytidine deaminase	CDA	1,07
cholinergic receptor, nicotinic alpha 5	CHRNA5	1,07
G protein-coupled receptor 139	GPR139	1,07
signal recognition particle 19kDa; zinc finger (CCCH type), RNA binding motif and serine/arginine rich 1	SRP19; ZRSR1	1,07
dynactin 3 (p22)	DCTN3	1,07
growth factor receptor bound protein 14	GRB14	1,07
UBX domain protein 11	UBXN11	1,07
olfactomedin like 2B	OLFML2B	1,07
olfactory receptor, family 6, subfamily C, member 70	OR6C70	1,07
HERPUD family member 2	HERPUD2	1,07
RALY RNA binding protein-like	RALYL	1,07
enabled homolog (Drosophila)	ENAH	1,07
eyes shut homolog (Drosophila)	EYS	1,07
mitochondrial ribosomal protein S18C	MRPS18C	1,07
cytochrome c oxidase assembly factor 1 homolog	COA1	1,07
HOP homeobox	HOPX	1,07
proline rich 23B	PRR23B	1,07
lymphoid enhancer-binding factor 1	LEF1	1,07
orthodenticle homeobox 1	OTX1	1,07
	LRIG1	1,07
olfactory receptor, family 5, subfamily B, member 21	OR5B21	1,07
retrotransposon-like 1	RTL1	1,07
CD247 molecule	CD247	1,07
Jeck2013 ANTISENSE, CDS, coding, INTERNAL, intronic, OVCODE, OVEXON best transcript NM_002183	IL3RA	1,07
ribosomal protein S9	RPS9	1,07
lymphocyte antigen 6 complex, locus G6E (pseudogene)	LY6G6E	1,07
transforming, acidic coiled-coil containing protein 3	TACC3	1,07
death-associated protein kinase 3; microRNA 637	DAPK3; MIR637	1,07
ubiquinol-cytochrome c reductase complex assembly factor 2	UQCC2	1,07
coiled-coil domain containing 109B	CCDC109B	1,07
calcium channel, voltage-dependent, R type, alpha 1E subunit	CACNA1E	1,07
carbohydrate kinase domain containing	CARKD	1,07
G protein pathway suppressor 1	GPS1	1,07
DNA meiotic recombinase 1	DMC1	1,07

eukaryotic translation initiation factor 4 gamma, 3	EIF4G3	1,07
KISS1 receptor	KISS1R	1,07
zinc finger protein 558	ZNF558	1,07
solute carrier family 35 (UDP-xylose/UDP-N-acetylglucosamine transporter), member B4	SLC35B4	1,07
coiled-coil domain containing 184	CCDC184	1,07
bladder cancer associated protein	BLCAP	1,07
anti-silencing function 1A histone chaperone	ASF1A	1,07
stanniocalcin 2	STC2	1,07
ras homolog family member U	RHOU	1,07
cytohesin 3	CYTH3	1,07
guanylate binding protein 5	GBP5	1,07
guanylate binding protein 2, interferon-inducible	GBP2	1,07
ubiquitin protein ligase E3 component n-recognin 4	UBR4	1,07
zinc finger, AN1-type domain 2A	ZFAND2A	1,07
URB2 ribosome biogenesis 2 homolog (S. cerevisiae)	URB2	1,07
Finkel-Biskis-Reilly murine sarcoma virus (FBR-MuSV) ubiquitously expressed	FAU	1,07
Memczak2013 ANTISENSE, CDS, coding, INTERNAL best transcript NM_001204426	LIMK1	1,07
ABO blood group (transferase A, alpha 1-3-N-acetylgalactosaminyltransferase; transferase B, alpha 1-3-galactosyltransferase) [Source:HGNC Symbol;Acc:HGNC:79]	ABO	1,07
ankyrin repeat domain 2 (stretch responsive muscle)	ANKRD2	1,07
transmembrane protein 220	TMEM220	1,07
MANSC domain containing 1	MANSC1	1,07
RAB3C, member RAS oncogene family	RAB3C	1,07
fibroblast growth factor 22	FGF22	1,07
fatty acid desaturase 2	FADS2	1,07
placenta specific 9	PLAC9	1,07
phospholipid scramblase 2	PLSCR2	1,07
ATP binding cassette subfamily G member 2 (Junior blood group)	ABCG2	1,07
adenosine deaminase	ADA	1,07
zinc finger protein 140	ZNF140	1,07
spastic paraplegia 21 (autosomal recessive, Mast syndrome)	SPG21	1,07
zinc finger protein 184	ZNF184	1,07
TBC1 domain family, member 2	TBC1D2	1,07
immunoglobulin superfamily, member 11	IGSF11	1,07
interferon-related developmental regulator 1	IFRD1	1,07
proline-rich transmembrane protein 3	PRRT3	1,07
DEAD (Asp-Glu-Ala-Asp) box helicase 21	DDX21	1,07
leucine zipper, down-regulated in cancer 1-like	LDOC1L	1,07
2-5-oligoadenylate synthetase 1	OAS1	1,07
tectonic family member 3	TCTN3	1,07

myosin light chain kinase	MYLK	1,07
ficolin (collagen/fibrinogen domain containing) 1	FCN1	1,07
testis expressed 29	TEX29	1,07
surfactant protein A1	SFTPA1	1,07
family with sequence similarity 136, member A	FAM136A	1,07
neuropeptide B	NPB	1,07
caspase recruitment domain family, member 6	CARD6	1,07
coiled-coil domain containing 166	CCDC166	1,07
anoctamin 6	ANO6	1,07
olfactory receptor, family 2, subfamily V, member 2	OR2V2	1,07
carbohydrate (N-acetylglucosamine 6-O) sulfotransferase 6	CHST6	1,07
nucleolar protein 4	NOL4	1,07
POC5 centriolar protein	POC5	1,07
SET domain and mariner transposase fusion gene	SETMAR	1,07
seryl-tRNA synthetase	SARS	1,07
general transcription factor IIE subunit 1	GTF2E1	1,07
Transcript Identified by AceView, Entrez Gene ID(s) 51663	ZFR	1,07
kinesin family member 1B	KIF1B	1,07
synovial sarcoma translocation, chromosome 18	SS18	1,07
RCSD domain containing 1	RCSD1	1,07
synovial sarcoma, X breakpoint 2B	SSX2B	1,07
TAF9 RNA polymerase II, TATA box binding protein (TBP)-associated factor, 32kDa; adenylate kinase 6	TAF9; AK6	1,07
nuclear pore complex interacting protein family, member B4	NPIPB4	1,07
MANSC domain containing 1	MANSC1	1,07
Fanconi anemia core complex associated protein 20	FAAP20	1,07
KAT8 regulatory NSL complex subunit 2; small nucleolar RNA, H/ACA box 2B	KANSL2; SNORA2B	1,07
Sjogren syndrome/scleroderma autoantigen 1	SSSCA1	1,07
BTB (POZ) domain containing 1	BTBD1	1,07
arrestin 3, retinal (X-arrestin)	ARR3	1,07
MIR1-1 host gene; microRNA 133a-2	MIR1-1HG; MIR133A2	1,07
B-box and SPRY domain containing	BSPRY	1,07
EPH receptor B2	EPHB2	1,07
nuclear transport factor 2	NUTF2	1,07
LIM domain kinase 2	LIMK2	1,07
palmdelphin	PALMD	1,07
stomatin (EPB72)-like 2	STOML2	1,07
transmembrane protease, serine 11B	TMPRSS11B	1,07
tyrosine 3-monooxygenase/tryptophan 5-monooxygenase activation protein, gamma	YWHAG	1,07
EPS8-like 3	EPS8L3	1,07
spectrin repeat containing, nuclear envelope 2	SYNE2	1,07
parathyroid hormone 1 receptor	PTH1R	1,07

kelch-like family member 40	KLHL40	1,07
cortactin	CTTN	1,07
cytochrome P450, family 4, subfamily B, polypeptide 1	CYP4B1	1,07
RNA binding motif protein 15	RBM15	1,07
basonuclin 2	BNC2	1,07
ATPase, Ca++ transporting, plasma membrane 2	ATP2B2	1,07
olfactory receptor, family 2, subfamily B, member 3	OR2B3	1,07
ankyrin repeat and zinc finger domain containing 1	ANKZF1	1,07
interleukin 36 receptor antagonist	IL36RN	1,07
zinc finger protein 780A	ZNF780A	1,07
cysteine and glycine-rich protein 3 (cardiac LIM protein)	CSRP3	1,07
aldehyde dehydrogenase 3 family, member A2	ALDH3A2	1,07
olfactory receptor, family 3, subfamily A, member 3	OR3A3	1,07
macrophage migration inhibitory factor (glycosylation-inhibiting factor)	MIF	1,07
ankyrin repeat domain 24	ANKRD24	1,06
olfactory receptor, family 4, subfamily X, member 2 (gene/pseudogene)	OR4X2	1,06
VPS26 retromer complex component A	VPS26A	1,06
phosphoinositide kinase, FYVE finger containing	PIKFYVE	1,06
NHL repeat containing E3 ubiquitin protein ligase 1	NHLRC1	1,06
sirtuin 7	SIRT7	1,06
dynactin associated protein	DYNAP	1,06
uncharacterized LOC79999; Homo sapiens uncharacterized protein ENSP00000382042 (LOC388436), mRNA.; novel transcript	LOC79999; LOC388436; RP11-744A16.4	1,06
SH3-binding domain protein 5-like	SH3BP5L	1,06
BCL2-like 11 (apoptosis facilitator)	BCL2L11	1,06
lysosomal-associated membrane protein 3	LAMP3	1,06
translin-associated factor X interacting protein 1	TSNAXIP1	1,06
transmembrane protein 183B	TMEM183B	1,06
lipocalin 2	LCN2	1,06
BCL2-associated athanogene 4	BAG4	1,06
exocyst complex component 3	EXOC3	1,06
carbohydrate (chondroitin 6) sulfotransferase 3	CHST3	1,06
brevican	BCAN	1,06
chromosome 2 open reading frame 50	C2orf50	1,06
zinc finger CCCH-type containing 3	ZC3H3	1,06
chromosome 9 open reading frame 91	C9orf91	1,06
coiled-coil domain containing 90B	CCDC90B	1,06
zinc finger protein 85	ZNF85	1,06
N-glycanase 1	NGLY1	1,06
E2F transcription factor 5, p130-binding	E2F5	1,06
glycine-N-acyltransferase-like 2	GLYATL2	1,06

Memczak2013 ANTISENSE, CDS, coding, INTERNAL best transcript NM_017519	ARID1B	1,06
hydroxymethylbilane synthase	HMBS	1,06
CREB3 regulatory factor	CREBRF	1,06
coiled-coil domain containing 191	CCDC191	1,06
solute carrier family 40 (iron-regulated transporter), member 1	SLC40A1	1,06
NIMA-related kinase 5	NEK5	1,06
3-hydroxybutyrate dehydrogenase, type 2	BDH2	1,06
PILR alpha associated neural protein	PIANP	1,06
5-nucleotidase, cytosolic IIIB	NT5C3B	1,06
translocase of outer mitochondrial membrane 5 homolog (yeast)	TOMM5	1,06
charged multivesicular body protein 4B	CHMP4B	1,06
lipoyltransferase 1	LIPT1	1,06
MRT4 homolog, ribosome maturation factor	MRTO4	1,06
golgi-associated, gamma adaptin ear containing, ARF binding protein 2	GGA2	1,06
single-minded family bHLH transcription factor 2	SIM2	1,06
IK cytokine, down-regulator of HLA II; microRNA 3655	IK; MIR3655	1,06
RE1-silencing transcription factor	REST	1,06
taste receptor, type 2, member 50	TAS2R50	1,06
minichromosome maintenance domain containing 2	MCMDC2	1,06
gap junction protein delta 4	GJD4	1,06
tubulin, alpha 4a	TUBA4A	1,06
C-type lectin domain family 4, member G	CLEC4G	1,06
lipoprotein, Lp(a)-like 2, pseudogene	LPAL2	1,06
heat shock 70kDa protein 14	HSPA14	1,06
G protein-coupled receptor 143	GPR143	1,06
actin related protein 2/3 complex subunit 1B	ARPC1B	1,06
uridine-cytidine kinase 1-like 1; microRNA 1914	UCKL1; MIR1914	1,06
phospholipid phosphatase 2	PLPP2	1,06
smoothened, frizzled class receptor	SMO	1,06
EPB41L4A antisense RNA 2 (head to head)	EPB41L4A-AS2	1,06
forkhead box H1	FOXH1	1,06
RUN and FYVE domain containing 2	RUFY2	1,06
proteasome 26S subunit, ATPase 4 [Source:HGNC Symbol;Acc:HGNC:9551]	PSMC4	1,06
branched chain amino-acid transaminase 1, cytosolic	BCAT1	1,06
ubiquitin-conjugating enzyme E2U (putative)	UBE2U	1,06
ER membrane protein complex subunit 2	EMC2	1,06
serine/threonine kinase 11 interacting protein	STK11IP	1,06
poliovirus receptor-related 1 (herpesvirus entry mediator C)	PVRL1	1,06
zinc finger protein 596	ZNF596	1,06
Fc fragment of IgE, low affinity II, receptor for (CD23)	FCER2	1,06

TNFSF12-TNFSF13 readthrough	TNFSF12-TNFSF13	1,06
B-cell translocation gene 4	BTG4	1,06
protease, serine 36	PRSS36	1,06
WAP four-disulfide core domain 13	WFDC13	1,06
methyl-CpG binding domain protein 3-like 1	MBD3L1	1,06
microspherule protein 1	MCRS1	1,06
Jeck2013 ALT_ACCEPTOR, ALT_DONOR, coding, INTERNAL, intronic best transcript NM_133509	RAD51B	1,06
transcription factor 19	TCF19	1,06
synaptonemal complex protein 2-like	SYCP2L	1,06
ZNF816-ZNF321P readthrough	ZNF816-ZNF321P	1,06
SLIT-ROBO Rho GTPase activating protein 2	SRGAP2	1,06
protein phosphatase 1, regulatory (inhibitor) subunit 14B	PPP1R14B	1,06
chromosome 6 open reading frame 99	C6orf99	1,06
Transcript Identified by AceView, Entrez Gene ID(s) 80232	WDR26	1,06
protein tyrosine phosphatase, non-receptor type 5 (striatum-enriched)	PTPN5	1,06
cyclin-dependent kinase 17	CDK17	1,06
lysozyme-like 2	LYZL2	1,06
toll-interleukin 1 receptor (TIR) domain containing adaptor protein	TIRAP	1,06
aldo-keto reductase family 1, member C4	AKR1C4	1,06
cell division cycle 34	CDC34	1,06
transducin (beta)-like 1, Y-linked	TBL1Y	1,06
phosphatidylinositol 4-kinase, catalytic, alpha pseudogene 2	PI4KAP2	1,06
Rho guanine nucleotide exchange factor 19	ARHGEF19	1,06
exosome component 2	EXOSC2	1,06
bleomycin hydrolase	BLMH	1,06
phospholipase A2, group V	PLA2G5	1,06
receptor accessory protein 5	REEP5	1,06
bromodomain PHD finger transcription factor	BPTF	1,06
V-set and immunoglobulin domain containing 10	VSIG10	1,06
cannabinoid receptor 1 (brain)	CNR1	1,06
CD3e molecule, epsilon associated protein	CD3EAP	1,06
tumor protein D52-like 2	TPD52L2	1,06
chemokine (C-X3-C motif) receptor 1	CX3CR1	1,06
ribosomal protein S6 kinase, 90kDa, polypeptide 1	RPS6KA1	1,06
protein O-linked mannose N-acetylglucosaminyltransferase 1 (beta 1,2-)	POMGNT1	1,06
F-box protein 9	FBXO9	1,06
SRA stem-loop interacting RNA binding protein	SLIRP	1,06
phospholipid phosphatase 6	PLPP6	1,06
Transcript Identified by AceView, Entrez Gene ID(s) 54453	RIN2	1,06

defensin, beta 110	DEFB110	1,06
peptidylprolyl isomerase (cyclophilin)-like 1	PPIL1	1,06
establishment of sister chromatid cohesion N-acetyltransferase 1	ESCO1	1,06
solute carrier family 41 (magnesium transporter), member 2	SLC41A2	1,06
NHL repeat containing 2	NHLRC2	1,06
PWP2 periodic tryptophan protein homolog (yeast)	PWP2	1,06
hexosaminidase B (beta polypeptide)	HEXB	1,06
DDB1 and CUL4 associated factor 4-like 1	DCAF4L1	1,06
ring finger protein 125, E3 ubiquitin protein ligase	RNF125	1,06
lysine (K)-specific demethylase 2B	KDM2B	1,06
solute carrier family 7 (anionic amino acid transporter), member 13	SLC7A13	1,06
matrix metallopeptidase 8	MMP8	1,06
olfactory receptor, family 1, subfamily K, member 1	OR1K1	1,06
ubiquitin protein ligase E3 component n-recognin 7 (putative)	UBR7	1,06
keratin 83, type II	KRT83	1,06
ribosomal protein S19	RPS19	1,06
metal response element binding transcription factor 2	MTF2	1,06
family with sequence similarity 83, member E	FAM83E	1,06
TRAF interacting protein	TRAIP	1,06
chromosome 7 open reading frame 62	C7orf62	1,06
serine peptidase inhibitor, Kunitz type, 3	SPINT3	1,06
tripartite motif containing 64	TRIM64	1,06
splicing factor proline/glutamine-rich	SFPQ	1,06
NADH dehydrogenase (ubiquinone) 1 alpha subcomplex, 10, 42kDa	NDUFA10	1,06
keratin 28, type I	KRT28	1,06
H6 family homeobox 3	HMX3	1,06
transcriptional adaptor 2A	TADA2A	1,06
major histocompatibility complex, class I-related	MR1	1,06
golgin A1	GOLGA1	1,06
metastasis suppressor 1-like	MTSS1L	1,06
family with sequence similarity 101, member A	FAM101A	1,06
zinc finger, BED-type containing 1; dehydrogenase/reductase (SDR family) X-linked	ZBED1; DHRSX	1,06
Memczak2013 ANTISENSE, coding, INTERNAL, UTR3 best transcript NM_016815	GYPC	1,06
protein tyrosine phosphatase, receptor type, S	PTPRS	1,06
EBNA1 binding protein 2; microRNA 6733	EBNA1BP2; MIR6733	1,06
long intergenic non-protein coding RNA 1270	LINC01270	1,06
BRF1, RNA polymerase III transcription initiation factor 90 kDa subunit	BRF1	1,06
ribosomal protein S6 kinase, 90kDa, polypeptide 1	RPS6KA1	1,06

zinc finger protein 267	ZNF267	1,06
ring finger protein 219	RNF219	1,06
tektin 2 (testicular)	TEKT2	1,06
tetratricopeptide repeat domain 34	TTC34	1,06
coiled-coil domain containing 86	CCDC86	1,06
isovaleryl-CoA dehydrogenase	IVD	1,06
leiomodin 1 (smooth muscle)	LMOD1	1,06
ankyrin repeat domain 13C	ANKRD13C	1,06
LTV1 ribosome biogenesis factor	LTV1	1,06
protease, serine 42	PRSS42	1,06
Salzman2013 ALT_ACCEPTOR, ALT_DONOR, coding, INTERNAL, intronic best transcript NM_012215	MGEA5	1,06
MYC binding protein; gap junction protein alpha 9	MYCBP; GJA9	1,06
solute carrier family 4 (anion exchanger), member 1 (Diego blood group)	SLC4A1	1,06
TBC1D22A antisense RNA 1	TBC1D22A-AS1	1,06
follistatin-like 3 (secreted glycoprotein)	FSTL3	1,06
angiopoietin like 4	ANGPTL4	1,06
PMS1 homolog 2, mismatch repair system component pseudogene 3	PMS2P3	1,06
Usher syndrome 1G (autosomal recessive)	USH1G	1,06
ADAM metallopeptidase domain 15	ADAM15	1,06
Homo sapiens XK, Kell blood group complex subunit-related, Y-linked 2 (XKRY2), mRNA.; Homo sapiens XK, Kell blood group complex subunit-related, Y-linked (XKRY), mRNA.; Homo sapiens XK, Kell blood group complex subunit-related, Y-linked, mRNA (cDNA clone MGC:164514 IMAGE:40146905), complete cds.; Homo sapiens XK, Kell blood group complex subunit-related, Y-linked, mRNA (cDNA clone MGC:164516 IMAGE:40146907), complete cds.; Homo sapiens XK, Kell blood group complex subunit-related, Y-linked 2, mRNA (cDNA clone MGC:164532 IMAGE:40146923), complete cds.; Homo sapiens XK, Kell blood group complex subunit-related, Y-linked 2, mRNA (cDNA clone MGC:164534 IMAGE:40146925), complete cds.; Synthetic construct Homo sapiens clone IMAGE:100015836, MGC:183189 XK, Kell blood group complex subunit-related, Y-linked 2 (XKRY2) mRNA, encodes complete protein.; Synthetic construct Homo sapiens clone IMAGE:100016528, MGC:184271 XK, Kell blood group complex subunit-related, Y-linked (XKRY) mRNA, encodes complete protein.; Transcript Identified by AceView, Entrez Gene ID(s) 353515, RefSeq ID(s) NM_004677, NM_001002906	XKRY2; XKRY	1,06
V-set and transmembrane domain containing 5	VSTM5	1,06
gamma-secretase activating protein	GSAP	1,06

Memczak2013 ANTISENSE, coding, INTERNAL, UTR3 best transcript NM_006378	SEMA4D	1,06
diablo, IAP-binding mitochondrial protein	DIABLO	1,06
TBC1 domain family, member 17; microRNA 4750	TBC1D17; MIR4750	1,06
mutS homolog 6	MSH6	1,06
homeobox A4	HOXA4	1,06
G protein-coupled receptor kinase 1	GRK1	1,06
CAP-GLY domain containing linker protein 1	CLIP1	1,06
lysine (K)-specific methyltransferase 2C	KMT2C	1,06
lactase-like	LCTL	1,06
transmembrane protein 191C	TMEM191C	1,06
family with sequence similarity 47, member B	FAM47B	1,06
RuvB-like AAA ATPase 2	RUVBL2	1,06
family with sequence similarity 132, member A	FAM132A	1,06
SH2 domain containing 1A	SH2D1A	1,06
aminoadipate-semialdehyde dehydrogenase-phosphopantetheinyl transferase	AASDHPPT	1,06
family with sequence similarity 69, member A	FAM69A	1,06
cystatin SN	CST1	1,06
excision repair cross-complementation group 6; ERCC6-PGBD3 readthrough; piggyBac transposable element derived 3	ERCC6; ERCC6-PGBD3; PGBD3	1,06
transmembrane channel like 4	TMC4	1,06
serine/arginine-rich splicing factor 5	SRSF5	1,06
tetraspanin 13	TSPAN13	1,06
aldo-keto reductase family 1, member D1	AKR1D1	1,06
myosin binding protein H	MYBPH	1,06
matrix metallopeptidase 19	MMP19	1,06
fibronectin leucine rich transmembrane protein 3	FLRT3	1,06
V-set and immunoglobulin domain containing 8	VSIG8	1,06
spermatogenesis and oogenesis specific basic helix-loop-helix 2	SOHLH2	1,06
testis-specific kinase 2	TESK2	1,06
spindle and kinetochore associated complex subunit 2	SKA2	1,06
zinc finger and SCAN domain containing 31	ZSCAN31	1,06
transmembrane protein 88B	TMEM88B	1,06
ets homologous factor	EHF	1,06
mitochondrial ribosomal protein S36	MRPS36	1,06
single-pass membrane protein with coiled-coil domains 2	SMCO2	1,06
general transcription factor IIIC subunit 6	GTF3C6	1,06
microRNA 650; immunoglobulin lambda variable 3-9 (gene/pseudogene); immunoglobulin lambda variable 2-8	MIR650; IGLV3-9; IGLV2-8	1,06
estrogen receptor 1	ESR1	1,06
H2B histone family, member W, testis-specific	H2BFWT	1,06
potassium channel, inwardly rectifying subfamily J, member 6	KCNJ6	1,06
sequestosome 1	SQSTM1	1,06

cytokine like 1	CYTL1	1,06
poly(ADP-ribose) polymerase 1	PARP1	1,06
acid sensing ion channel 2	ASIC2	1,06
symplekin	SYMPK	1,06
solute carrier family 44, member 5	SLC44A5	1,06
N-acetyltransferase 6 (GCN5-related)	NAT6	1,06
cerebellar degeneration-related protein 2-like	CDR2L	1,06
protease, serine 27	PRSS27	1,06
roundabout guidance receptor 4	ROBO4	1,06
proline-serine-threonine phosphatase interacting protein 2	PSTPIP2	1,06
family with sequence similarity 184, member B	FAM184B	1,06
zinc finger protein 860	ZNF860	1,06
adipocyte plasma membrane associated protein	APMAP	1,06
chromosome 9 open reading frame 84	C9orf84	1,06
Memczak2013 ANTISENSE, CDS, coding, INTERNAL best transcript NM_002473	MYH9	1,06
heat shock transcription factor family, X-linked 1	HSFX1	1,06
fatty acid 2-hydroxylase	FA2H	1,06
ATP binding cassette subfamily B member 4	ABCB4	1,06
VAMP associated protein A	VAPA	1,06
activin A receptor type IIA	ACVR2A	1,06
synaptonemal complex protein 1	SYCP1	1,06
caveolin 3	CAV3	1,06
TBC1 (tre-2/USP6, BUB2, cdc16) domain family, member 1	TBC1D1	1,06
ADAM-like, decysin 1	ADAMDEC1	1,06
inhibin beta B	INHBB	1,06
scavenger receptor class F, member 1	SCARF1	1,06
Jeck2013 ALT_DONOR, coding, INTERNAL, intronic best transcript NM_032134	QRICH2	1,06
Rho GTPase activating protein 10	ARHGAP10	1,06
Memczak2013 ANTISENSE, CDS, coding, INTERNAL best transcript NM_014423	AFF4	1,06
WAS protein family, member 1	WASF1	1,06
zinc finger, FYVE domain containing 9	ZFYVE9	1,06
synaptogyrin 2	SYNGR2	1,06
acyl-CoA thioesterase 4	ACOT4	1,06
Transcript Identified by AceView, Entrez Gene ID(s) 9736	USP34	1,06
peroxisome proliferator-activated receptor gamma, coactivator 1 beta	PPARGC1B	1,06
ring finger protein 216	RNF216	1,06
TSEN15 tRNA splicing endonuclease subunit	TSEN15	1,06
WD repeat domain 43; small nucleolar RNA, C/D box 53	WDR43; SNORD53	1,06
mitochondrial ribosomal protein L22	MRPL22	1,06
chromosome transmission fidelity factor 18	CHTF18	1,06

chromosome 10 open reading frame 76	C10orf76	1,06
catechol-O-methyltransferase; microRNA 4761	COMT; MIR4761	1,06
chromosome 20 open reading frame 24	C20orf24	1,06
tribbles pseudokinase 3	TRIB3	1,06
amine oxidase, copper containing 1	AOC1	1,06
zinc finger protein 546	ZNF546	1,06
glycosylphosphatidylinositol anchored high density lipoprotein binding protein 1	GPIHBP1	1,06
protein phosphatase 1, regulatory subunit 3F	PPP1R3F	1,06
cation channel, sperm associated 4	CATSPER4	1,06
canopy FGF signaling regulator 4	CNPY4	1,06
DnaJ (Hsp40) homolog, subfamily C, member 1	DNAJC1	1,06
solute carrier family 46, member 2	SLC46A2	1,06
potassium channel tetramerization domain containing 4	KCTD4	1,06
ZNF503 antisense RNA 2	ZNF503-AS2	1,06
FAT atypical cadherin 4	FAT4	1,06
serpin peptidase inhibitor, clade A (alpha-1 antiproteinase, antitrypsin), member 6	SERPINA6	1,06
beta 3-glucosyltransferase	B3GLCT	1,06
keratin 33A, type I	KRT33A	1,06
popeye domain containing 3	POPDC3	1,06
CoA synthase	COASY	1,06
killer cell lectin-like receptor subfamily F, member 2	KLRF2	1,06
zinc finger RNA binding protein	ZFR	1,06
G protein-coupled receptor 180	GPR180	1,06
ADAM metallopeptidase domain 28	ADAM28	1,06
zinc finger protein 43	ZNF43	1,06
coiled-coil domain containing 9	CCDC9	1,06
MDM2 binding protein	MTBP	1,06
collagen, type V, alpha 2	COL5A2	1,06
transforming, acidic coiled-coil containing protein 1	TACC1	1,06
anoctamin 4	ANO4	1,06
3-ketodihydrosphingosine reductase	KDSR	1,06
luteinizing hormone/choriogonadotropin receptor	LHCGR	1,06
dual specificity phosphatase 3	DUSP3	1,06
LY6/PLAUR domain containing 3	LYPD3	1,06
RAD50 homolog, double strand break repair protein	RAD50	1,06
mucolipin 1	MCOLN1	1,06
zinc finger, FYVE domain containing 19	ZFYVE19	1,06
S100 calcium binding protein A14	S100A14	1,06
aldehyde dehydrogenase 7 family, member A1	ALDH7A1	1,06
BTB (POZ) domain containing 8	BTBD8	1,06
thymosin beta 15a	TMSB15A	1,06
pseudouridylate synthase 3	PUS3	1,06

adhesion G protein-coupled receptor E5	ADGRE5	1,06
PDZ domain containing ring finger 3	PDZRN3	1,06
ubiquitin A-52 residue ribosomal protein fusion product 1	UBA52	1,06
origin recognition complex subunit 6	ORC6	1,06
zinc finger protein 888	ZNF888	1,06
islet cell autoantigen 1	ICA1	1,06
methyltransferase like 16	METTL16	1,06
homeobox C6; homeobox C4; homeobox C5	HOXC6; HOXC4; HOXC5	1,06
WD and tetratricopeptide repeats 1	WDTC1	1,06
methionyl aminopeptidase type 1D (mitochondrial)	METAP1D	1,06
BAI1-associated protein 2-like 1	BAIAP2L1	1,06
chromosome 2 open reading frame 57	C2orf57	1,06
ubiquitin conjugating enzyme E2N	UBE2N	1,06
transmembrane protein 125	TMEM125	1,06
dopamine receptor D2	DRD2	1,06
microRNA 1247	MIR1247	1,06
NK6 homeobox 1	NKX6-1	1,06
ZFP30 zinc finger protein	ZFP30	1,06
chromosome 4 open reading frame 47	C4orf47	1,06
HtrA serine peptidase 3	HTRA3	1,06
adaptor-related protein complex 3, mu 2 subunit	AP3M2	1,06
sperm antigen with calponin homology and coiled-coil domains 1-like	SPECC1L	1,06
lipocalin 1	LCN1	1,06
FK506 binding protein 4	FKBP4	1,06
dystroglycan 1 (dystrophin-associated glycoprotein 1)	DAG1	1,06
ATPase, aminophospholipid transporter (APLT), class I, type 8A, member 1	ATP8A1	1,06
FtsJ RNA methyltransferase homolog 2 (E. coli)	FTSJ2	1,06
glucokinase (hexokinase 4)	GCK	1,06
zinc finger protein 850	ZNF850	1,06
metallothionein 1G	MT1G	1,06
DnaJ (Hsp40) homolog, subfamily C, member 2	DNAJC2	1,06
cathepsin W	CTSW	1,06
WAS protein family, member 2	WASF2	1,06
proteasome 26S subunit, ATPase 4	PSMC4	1,06
calcyphosine	CAPS	1,06
endomucin	EMCN	1,06
GEN1 Holliday junction 5 flap endonuclease	GEN1	1,06
tubulin, gamma complex associated protein 4	TUBGCP4	1,06
protein tyrosine phosphatase, receptor type, G	PTPRG	1,06
hypoxia up-regulated 1	HYOU1	1,06
solute carrier family 38, member 3	SLC38A3	1,06
cyclin-dependent kinase inhibitor 2D (p19, inhibits CDK4)	CDKN2D	1,06

androgen-induced 1	AIG1	1,06
nuclear prelamin A recognition factor	NARF	1,06
zinc and ring finger 3	ZNRF3	1,06
achalasia, adrenocortical insufficiency, alacrimia	AAAS	1,06
asparagine-linked glycosylation 1-like 9, pseudogene	ALG1L9P	1,06
citrate lyase beta like	CLYBL	1,06
annexin A11	ANXA11	1,06
hydroxyacyl-CoA dehydrogenase/3-ketoacyl-CoA thiolase/enoyl-CoA hydratase (trifunctional protein), alpha subunit	HADHA	1,06
kinesin family member 15	KIF15	1,06
chromosome 9 open reading frame 3; microRNA 24-1; microRNA 23b; microRNA 27b	C9orf3; MIR24-1; MIR23B; MIR27B	1,06
N-terminal EF-hand calcium binding protein 3	NECAB3	1,06
coiled-coil domain containing 187	CCDC187	1,06
valosin containing protein	VCP	1,06
P antigen family, member 2 (prostate associated)	PAGE2	1,06
mitogen-activated protein kinase-activated protein kinase 2	MAPKAPK2	1,06
v-myc avian myelocytomatosis viral oncogene homolog	MYC	1,06
growth arrest and DNA-damage-inducible, alpha	GADD45A	1,06
protease, serine, 54	PRSS54	1,06
Transcript Identified by AceView, Entrez Gene ID(s) 93624	TADA2B	1,06
zinc finger, DHHC-type containing 7	ZDHHC7	1,06
major facilitator superfamily domain containing 5	MFSD5	1,06
huntingtin-associated protein 1	HAP1	1,06
homeodomain interacting protein kinase 3	HIPK3	1,06
docking protein 6	DOK6	1,06
pyruvate carboxylase	PC	1,06
spermatogenesis and centriole associated 1	SPATC1	1,06
Memczak2013 ALT_ACCEPTOR, ALT_DONOR, coding, INTERNAL, intronic best transcript NM_002015	FOXO1	1,06
poly(ADP-ribose) polymerase family member 14	PARP14	1,06
HEAT repeat containing 9	HEATR9	1,06
SWI/SNF related, matrix associated, actin dependent regulator of chromatin, subfamily d, member 2	SMARCD2	1,06
ventricular zone expressed PH domain containing 1	VEPH1	1,06
HHIP-like 1	HHIPL1	1,06
delta(4)-desaturase, sphingolipid 2	DEGS2	1,06
solute carrier family 4 (anion exchanger), member 1, adaptor protein	SLC4A1AP	1,06
surfeit 2 [Source:HGNC Symbol;Acc:HGNC:11475]	SURF2	1,06
kelch-like family member 7	KLHL7	1,06
ring finger protein 165	RNF165	1,06
integrator complex subunit 9	INTS9	1,06

PPAN-P2RY11 readthrough; purinergic receptor P2Y, G-protein coupled, 11; peter pan homolog (Drosophila)	PPAN-P2RY11; P2RY11; PPAN	1,06
cellular repressor of E1A-stimulated genes 1	CREG1	1,06
long intergenic non-protein coding RNA 1590; small integral membrane protein 8	LINC01590; SMIM8	1,06
FLJ33360 protein; FLJ33360 protein [Source:EntrezGene;Acc:401172]; putative novel transcript	FLJ33360; CTD-2324F15.2	1,06
WEE1 G2 checkpoint kinase	WEE1	1,06
zinc finger with KRAB and SCAN domains 8	ZKSCAN8	1,06
adhesion G protein-coupled receptor G7	ADGRG7	1,06
ALS2, alsin Rho guanine nucleotide exchange factor	ALS2	1,06
ankyrin repeat and LEM domain containing 1	ANKLE1	1,06
DnaJ (Hsp40) homolog, subfamily B, member 12	DNAJB12	1,06
inositol polyphosphate phosphatase-like 1	INPPL1	1,06
histone cluster 1, H2bk	HIST1H2BK	1,06
mitochondrial ribosomal protein L37	MRPL37	1,06
peptide deformylase (mitochondrial); component of oligomeric golgi complex 8; transmembrane p24 trafficking protein 6	PDF; COG8; TMED6	1,06
olfactory receptor, family 2, subfamily H, member 1	OR2H1	1,06
cytochrome P450, family 4, subfamily X, polypeptide 1	CYP4X1	1,06
general transcription factor IIIC subunit 5	GTF3C5	1,06
zinc finger and BTB domain containing 39	ZBTB39	1,06
CREB regulated transcription coactivator 2	CRTC2	1,06
methionine sulfoxide reductase B2	MSRB2	1,06
Transcript Identified by AceView, Entrez Gene ID(s) 9695	EDEM1	1,06
septin 8	sept-08	1,06
family with sequence similarity 47, member A	FAM47A	1,06
zinc finger protein 587	ZNF587	1,06
neuronal differentiation 6	NEUROD6	1,06
myosin light chain 1	MYL1	1,06
Rho family GTPase 2	RND2	1,06
DENN/MADD domain containing 2C	DENND2C	1,06
spondin 1, extracellular matrix protein	SPON1	1,06
spermatogenesis associated 12	SPATA12	1,06
heterogeneous nuclear ribonucleoprotein A/B	HNRNPAB	1,06
family with sequence similarity 81, member A	FAM81A	1,06
Sp1 transcription factor	SP1	1,06
chromosome 17 open reading frame 112	C17orf112	1,06
Mab-21 domain containing 1	MB21D1	1,06
BRICHOS domain containing 5	BRICD5	1,06
growth differentiation factor 3	GDF3	1,06
NSFL1 (p97) cofactor (p47)	NSFL1C	1,06
3-hydroxymethyl-3-methylglutaryl-CoA lyase	HMGCL	1,06
inositol polyphosphate-5-phosphatase F	INPP5F	1,06
GDP-mannose 4,6-dehydratase	GMDS	1,06

corepressor interacting with RBPJ, 1	CIR1	1,06
Transcript Identified by AceView, Entrez Gene ID(s) 2788	GNG7	1,06
nascent polypeptide-associated complex alpha subunit 2	NACA2	1,06
neuropeptide VF precursor	NPVF	1,06
coiled-coil domain containing 91	CCDC91	1,06
purinergic receptor P2X, ligand gated ion channel, 6	P2RX6	1,06
SET domain containing 3	SETD3	1,06
nuclear receptor binding SET domain protein 1	NSD1	1,06
inosine triphosphatase (nucleoside triphosphate pyrophosphatase)	ITPA	1,06
IQ motif and Sec7 domain 2	IQSEC2	1,06
zinc finger, B-box domain containing	ZBBX	1,06
protein phosphatase 2, regulatory subunit B, delta	PPP2R5D	1,06
activating transcription factor 2	ATF2	1,06
profilin family, member 4	PFN4	1,06
coiled-coil domain containing 85C	CCDC85C	1,06
proline rich 25	PRR25	1,06
chromosome 16 open reading frame 72-like	LOC389895	1,06
patatin-like phospholipase domain containing 6	PNPLA6	1,06
Transcript Identified by AceView, Entrez Gene ID(s) 91057	CCDC34	1,06
replication protein A4	RPA4	1,06
JAKMIP2 antisense RNA 1	JAKMIP2-AS1	1,06
double homeobox 4 like 8	DUX4L8	1,06
microtubule-associated protein 1 light chain 3 beta 2	MAP1LC3B2	1,06
bombesin-like receptor 3	BRS3	1,06
DiGeorge syndrome critical region gene 2; DiGeorge syndrome critical region gene 11 (non-protein coding)	DGCR2; DGCR11	1,06
polo-like kinase 2	PLK2	1,06
listerin E3 ubiquitin protein ligase 1	LTN1	1,06
Transcript Identified by AceView, Entrez Gene ID(s) 54108	CHRAC1	1,06
YOD1 deubiquitinase	YOD1	1,06
adaptor-related protein complex 1 sigma 3 subunit	AP1S3	1,06
Memczak2013 ANTISENSE, CDS, coding, INTERNAL best transcript NM_012235	SCAP	1,06
pancreatic polypeptide	PPY	1,06
protein phosphatase 1, regulatory subunit 9B	PPP1R9B	1,06
opioid receptor, delta 1	OPRD1	1,06
secretoglobin, family 1D, member 2	SCGB1D2	1,06
long intergenic non-protein coding RNA 1559	LINC01559	1,06
ankyrin repeat and SOCS box containing 4	ASB4	1,06
phosphoglucomutase 5	PGM5	1,06
Memczak2013 ALT_ACCEPTOR, ALT_DONOR, coding, INTERNAL, intronic best transcript NM_001142393	NEDD9	1,06
DDB1 and CUL4 associated factor 4-like 2	DCAF4L2	1,06

regulator of chromosome condensation (RCC1) and BTB (POZ) domain containing protein 2	RCBTB2	1,06
WD repeat domain 92	WDR92	1,06
keratin associated protein 29-1	KRTAP29-1	1,06
arsA arsenite transporter, ATP-binding, homolog 1 (bacterial)	ASNA1	1,06
NCK-associated protein 1-like	NCKAP1L	1,06
inhibitor of Bruton agammaglobulinemia tyrosine kinase	IBTK	1,06
lysine (K)-specific demethylase 7A	KDM7A	1,06
ureidopropionase, beta	UPB1	1,06
Transcript Identified by AceView, Entrez Gene ID(s) 79598	CEP97	1,06
family with sequence similarity 46, member D	FAM46D	1,06
ADP-ribosylation factor like GTPase 4A	ARL4A	1,06
cofilin 1 (non-muscle)	CFL1	1,06
enamelin	ENAM	1,06
NIF3 NGG1 interacting factor 3-like 1	NIF3L1	1,06
lymphocyte-specific protein 1 pseudogene 3	LSP1P3	1,06
ER membrane-associated RNA degradation	ERMARD	1,06
dynamin 2	DNM2	1,06
olfactory receptor, family 10, subfamily J, member 4 (gene/pseudogene) [Source:HGNC Symbol;Acc:HGNC:15408]	OR10J4	1,06
tripartite motif containing 45	TRIM45	1,06
myotubularin related protein 11	MTMR11	1,06
chromosome 1 open reading frame 87	C1orf87	1,06
synovial sarcoma, X breakpoint 5	SSX5	1,06
serine/arginine-rich splicing factor 8	SRSF8	1,06
mitogen-activated protein kinase 14	MAPK14	1,06
indoleamine 2,3-dioxygenase 2	IDO2	1,06
malonyl-CoA-acyl carrier protein transacylase	MCAT	1,06
zinc finger protein 480	ZNF480	1,06
protocadherin 8	PCDH8	1,06
GA binding protein transcription factor, beta subunit 2	GABPB2	1,06
InaD-like (Drosophila)	INADL	1,06
double homeobox 4 like 14; double homeobox 4 like 15	DUX4L14; DUX4L15	1,06
dual specificity tyrosine-(Y)-phosphorylation regulated kinase 1B	DYRK1B	1,06
MAF transcriptional regulator RNA	MAFTRR	1,06
eukaryotic translation initiation factor 3, subunit C; eukaryotic translation initiation factor 3, subunit C-like	EIF3C; EIF3CL	1,06
HtrA serine peptidase 4	HTRA4	1,06
modulator of apoptosis 1	MOAP1	1,06
GSG1-like	GSG1L	1,06
coiled-coil domain containing 159	CCDC159	1,06
protein phosphatase 1, catalytic subunit, alpha isozyme	PPP1CA	1,06
cleavage and polyadenylation specific factor 6	CPSF6	1,06
pannexin 2	PANX2	1,06

glutamate receptor, ionotropic, N-methyl D-aspartate-associated protein 1 (glutamate binding)	GRINA	1,06
ubiquitin associated protein 2 like	UBAP2L	1,06
mitogen-activated protein kinase kinase kinase kinase 4	MAP4K4	1,06
hedgehog acyltransferase	HHAT	1,06
ankyrin repeat and SOCS box containing 10	ASB10	1,06
ankyrin repeat and SOCS box containing 14	ASB14	1,06
protein kinase, cAMP-dependent, regulatory, type II, alpha	PRKAR2A	1,06
topoisomerase I binding, arginine/serine-rich, E3 ubiquitin protein ligase	TOPORS	1,06
ciliary neurotrophic factor receptor	CNTFR	1,06
nudix hydrolase 4 pseudogene 1; nudix hydrolase 4 pseudogene 2; nudix hydrolase 4	NUDT4P1; NUDT4P2; NUDT4	1,06
nudix hydrolase 4; nudix hydrolase 4 pseudogene 1	NUDT4; NUDT4P1	1,06
DEAD (Asp-Glu-Ala-Asp) box polypeptide 60-like	DDX60L	1,06
PCF11 cleavage and polyadenylation factor subunit	PCF11	1,06
T-box 5	TBX5	1,06
cystatin 11	CST11	1,06
calmodulin regulated spectrin-associated protein family, member 2	CAMSAP2	1,06
Yip1 domain family member 3	YIPF3	1,06
wingless-type MMTV integration site family, member 5A	WNT5A	1,06
zinc finger protein 710	ZNF710	1,06
laminin, gamma 1 (formerly LAMB2)	LAMC1	1,06
sel-1 suppressor of lin-12-like (C. elegans)	SEL1L	1,06
matrix metallopeptidase 21	MMP21	1,06
KRI1 homolog	KRI1	1,06
CCR4-NOT transcription complex subunit 6-like	CNOT6L	1,06
STAU2 antisense RNA 1	STAU2-AS1	1,06
F-box and leucine-rich repeat protein 17	FBXL17	1,06
pyrin domain containing 2	PYDC2	1,06
leucine rich repeat and fibronectin type III domain containing 3	LRFN3	1,06
MARVEL domain containing 2	MARVELD2	1,06
G protein-coupled receptor 31	GPR31	1,06
CKLF-like MARVEL transmembrane domain containing 3	CMTM3	1,06
family with sequence similarity 162, member B	FAM162B	1,06
nicotinamide riboside kinase 2	NMRK2	1,06
S-antigen; retina and pineal gland (arrestin)	SAG	1,06
vomeronasal 1 receptor 3 (gene/pseudogene)	VN1R3	1,06
aldehyde dehydrogenase 3 family, member A1	ALDH3A1	1,06
membrane-spanning 4-domains, subfamily A, member 14	MS4A14	1,06
signal transducer and activator of transcription 5A	STAT5A	1,06
ribosomal protein S6 kinase, 90kDa, polypeptide 1	RPS6KA1	1,06

PR domain containing 16	PRDM16	1,06
NSA2 ribosome biogenesis homolog	NSA2	1,06
anoctamin 2, calcium activated chloride channel	ANO2	1,06
centrosome and spindle pole associated protein 1	CSPP1	1,06
solute carrier family 38, member 5	SLC38A5	1,06
olfactory receptor, family 4, subfamily D, member 2	OR4D2	1,06
zinc finger protein 774	ZNF774	1,06
trafficking protein particle complex 4	TRAPPC4	1,06
transcription factor 25 (basic helix-loop-helix)	TCF25	1,06
tubulin, alpha 8	TUBA8	1,06
abhydrolase domain containing 2	ABHD2	1,06
discoidin, CUB and LCCL domain containing 2	DCBLD2	1,06
nuclear transcription factor Y subunit beta	NFYB	1,06
aldo-keto reductase family 1, member C2	AKR1C2	1,06
PRAME family member 13	PRAMEF13	1,06
DnaJ (Hsp40) homolog, subfamily B, member 5	DNAJB5	1,06
roundabout guidance receptor 1	ROBO1	1,06
NADH dehydrogenase (ubiquinone) 1 alpha subcomplex, 2, 8kDa	NDUFA2	1,06
G-protein signaling modulator 3	GPSM3	1,06
SMAD specific E3 ubiquitin protein ligase 1	SMURF1	1,06
protein kinase C substrate 80K-H	PRKCSH	1,06
phosphodiesterase 8B	PDE8B	1,06
coatomer protein complex subunit gamma 1	COPG1	1,06
ribosomal protein L32; small nucleolar RNA, H/ACA box 7A	RPL32; SNORA7A	1,06
nuclear receptor subfamily 0, group B, member 2	NR0B2	1,06
olfactory receptor, family 5, subfamily AR, member 1 (gene/pseudogene)	OR5AR1	1,06
chromosome 11 open reading frame 63	C11orf63	1,06
MAFF interacting protein (pseudogene); tektin 4 pseudogene 2	MAFIP; TEKT4P2	1,06
olfactory receptor, family 6, subfamily T, member 1	OR6T1	1,06
Transcript Identified by AceView, Entrez Gene ID(s) 27019	DNAI1	1,06
sushi domain containing 5	SUSD5	1,06
syntaxin binding protein 5-like	STXBP5L	1,06
insulin-like growth factor 2 mRNA binding protein 1	IGF2BP1	1,06
mago homolog, exon junction complex core component	MAGOH	1,06
tyrosine 3-monooxygenase/tryptophan 5-monooxygenase activation protein, beta	YWHAB	1,06
methionyl-tRNA synthetase; microRNA 6758	MARS; MIR6758	1,06
eukaryotic translation initiation factor 4E binding protein 1	EIF4EBP1	1,06
FERM domain containing 4A	FRMD4A	1,06
succinate-CoA ligase, alpha subunit	SUCLG1	1,06
baculoviral IAP repeat containing 5	BIRC5	1,06

actin related protein 2/3 complex subunit 3	ARPC3	1,06
ALG11, alpha-1,2-mannosyltransferase; UTP14, U3 small nucleolar ribonucleoprotein, homolog C (yeast)	ALG11; UTP14C	1,06
oxytocin receptor	OXTR	1,06
coiled-coil domain containing 78	CCDC78	1,06
receptor tyrosine kinase-like orphan receptor 2	ROR2	1,06
heterogeneous nuclear ribonucleoprotein C-like 1	HNRNPCL1	1,06
heat shock transcription factor, Y-linked 2; heat shock transcription factor, Y-linked 1	HSFY2; HSFY1	1,06
PRELI domain containing 1	PRELID1	1,06
DEAD (Asp-Glu-Ala-Asp) box polypeptide 19A	DDX19A	1,06
deoxynucleotidyltransferase, terminal, interacting protein 1	DNTTIP1	1,06
NADH dehydrogenase (ubiquinone) Fe-S protein 5, 15kDa (NADH-coenzyme Q reductase)	NDUFS5	1,06
activity-dependent neuroprotector homeobox	ADNP	1,06
solute carrier family 25 (mitochondrial carrier; phosphate carrier), member 25	SLC25A25	1,06
Memczak2013 ANTISENSE, coding, INTERNAL, intronic best transcript NM_182734	PLCB1	1,06
cytochrome c oxidase subunit VIIa polypeptide 2 like	COX7A2L	1,06
zinc finger protein 148	ZNF148	1,06
FERM domain containing 4B	FRMD4B	1,06
RAB12, member RAS oncogene family	RAB12	1,06
proline rich 15	PRR15	1,06
ATP binding cassette subfamily F member 3	ABCF3	1,06
RAS p21 protein activator 3	RASA3	1,06
arylsulfatase family, member H	ARSH	1,06
NFAT activating protein with ITAM motif 1	NFAM1	1,06
cytochrome P450, family 2, subfamily S, polypeptide 1	CYP2S1	1,06
PX domain containing serine/threonine kinase	PXK	1,06
neural retina leucine zipper	NRL	1,05
lysine (K)-specific methyltransferase 5C	KMT5C	1,05
zinc finger with KRAB and SCAN domains 7	ZKSCAN7	1,05
phosducin like 3	PDCL3	1,05
v-myb avian myeloblastosis viral oncogene homolog	MYB	1,05
HEPACAM family member 2	HEPACAM2	1,05
isochorismatase domain containing 1	ISOC1	1,05
chromosome 17 open reading frame 53	C17orf53	1,05
nucleolar protein 4-like	NOL4L	1,05
colipase, pancreatic	CLPS	1,05
glucosamine-6-phosphate deaminase 1	GNPDA1	1,05
autophagy related 4A, cysteine peptidase	ATG4A	1,05
nei-like DNA glycosylase 3	NEIL3	1,05
ATPase, Na+/K+ transporting, alpha 4 polypeptide	ATP1A4	1,05
breast cancer metastasis suppressor 1	BRMS1	1,05

cytochrome P450, family 2, subfamily D, polypeptide 6	CYP2D6	1,05
kazrin, periplakin interacting protein	KAZN	1,05
matrix metallopeptidase 7	MMP7	1,05
FSHD region gene 1 family member B, pseudogene	FRG1BP	1,05
Memczak2013 ANTISENSE, CDS, coding, INTERNAL best transcript NM_005120	MED12	1,05
cysteine and histidine rich domain containing 1	CHORDC1	1,05
Memczak2013 ALT_ACCEPTOR, ALT_DONOR, coding, INTERNAL, intronic best transcript NM_001025108	AFF3	1,05
tescalcin	TESC	1,05
STE20-related kinase adaptor alpha	STRADA	1,05
p21 protein (Cdc42/Rac)-activated kinase 2	PAK2	1,05
capping protein (actin filament) muscle Z-line, alpha 1	CAPZA1	1,05
prostaglandin-endoperoxide synthase 2 (prostaglandin G/H synthase and cyclooxygenase)	PTGS2	1,05
hydroxymethylbilane synthase	HMBS	1,05
patatin-like phospholipase domain containing 1	PNPLA1	1,05
family with sequence similarity 163, member A	FAM163A	1,05
Obg-like ATPase 1	OLA1	1,05
vacuolar protein sorting 36 homolog (S. cerevisiae)	VPS36	1,05
leucine rich repeat containing 55	LRRC55	1,05
activating transcription factor 4	ATF4	1,05
transmembrane 9 superfamily protein member 4	TM9SF4	1,05
CDK5 regulatory subunit associated protein 2	CDK5RAP2	1,05
chromosome 11 open reading frame 70	C11orf70	1,05
CTD nuclear envelope phosphatase 1 regulatory subunit 1	CNEP1R1	1,05
transmembrane protein 39B	TMEM39B	1,05
stromal antigen 1	STAG1	1,05
dipeptidyl-peptidase 9	DPP9	1,05
arginyl-tRNA synthetase 2, mitochondrial	RARS2	1,05
NFKB repressing factor	NKRF	1,05
synergin, gamma	SYNRG	1,05
zinc finger protein 174	ZNF174	1,05
Ewing tumor-associated antigen 1	ETAA1	1,05
solute carrier family 18 (vesicular monoamine transporter), member 2	SLC18A2	1,05
zinc finger protein 879	ZNF879	1,05
ADP-ribosylation factor 6	ARF6	1,05
RAB11 family interacting protein 5 (class I)	RAB11FIP5	1,05
zinc finger protein 771	ZNF771	1,05
zinc finger protein 789	ZNF789	1,05
speedy/RINGO cell cycle regulator family member E3	SPDYE3	1,05
N(alpha)-acetyltransferase 20, NatB catalytic subunit	NAA20	1,05

novel transcript, antisense to C16orf53 and MVP; PAXIP1 associated glutamate-rich protein 1 [Source:HGNC Symbol;Acc:HGNC:28707]	AC009133.12; PAGR1	1,05
NOP2/Sun domain family, member 5 pseudogene 2; NOP2/Sun domain family, member 5 pseudogene 1	NSUN5P2; NSUN5P1	1,05
epithelial cell adhesion molecule	EPCAM	1,05
succinyl-CoA:glutarate-CoA transferase	SUGCT	1,05
fibroblast growth factor receptor substrate 2	FRS2	1,05
family with sequence similarity 234, member A; Rho GDP dissociation inhibitor (GDI) gamma	FAM234A; ARHGDIG	1,05
matrix metallopeptidase 1	MMP1	1,05
solute carrier family 35 (adenosine 3-phospho 5-phosphosulfate transporter), member B2; microRNA 4647	SLC35B2; MIR4647	1,05
ubiquitin conjugating enzyme E2N-like (gene/pseudogene)	UBE2NL	1,05
F-box and leucine-rich repeat protein 7	FBXL7	1,05
SH2 domain containing 2A	SH2D2A	1,05
chromosome 1 open reading frame 112	C1orf112	1,05
dCTP pyrophosphatase 1	DCTPP1	1,05
ERI1 exoribonuclease family member 2	ERI2	1,05
olfactory receptor, family 2, subfamily T, member 12	OR2T12	1,05
Jeck2013 ALT_ACCEPTOR, ALT_DONOR, coding, INTERNAL, intronic, OVERLAPTX, OVEXON best transcript NM_000942	PPIB	1,05
acidic nuclear phosphoprotein 32 family member D	ANP32D	1,05
ALX homeobox 4	ALX4	1,05
CDGSH iron sulfur domain 1	CISD1	1,05
tryptase beta 2 (gene/pseudogene); tryptase alpha/beta 1	TPSB2; TPSAB1	1,05
coiled-coil domain containing 6	CCDC6	1,05
Transcript Identified by AceView, Entrez Gene ID(s) 4952	OCRL	1,05
heterogeneous nuclear ribonucleoprotein A1	HNRNPA1	1,05
chloride intracellular channel 4	CLIC4	1,05
PRAME family member 8	PRAMEF8	1,05
mitochondrial ribosomal protein S31	MRPS31	1,05
serine palmitoyltransferase, long chain base subunit 2	SPTLC2	1,05
core 1 synthase, glycoprotein-N-acetylgalactosamine 3-beta-galactosyltransferase 1	C1GALT1	1,05
serpin peptidase inhibitor, clade F (alpha-2 antiplasmin, pigment epithelium derived factor), member 1	SERPINF1	1,05
fasciculation and elongation protein zeta 1	FEZ1	1,05
G protein-coupled receptor kinase 6	GRK6	1,05
calcium channel, voltage-dependent, alpha 2/delta subunit 3	CACNA2D3	1,05
ubiquinol-cytochrome c reductase, Rieske iron-sulfur polypeptide 1	UQCRFS1	1,05
KIAA0355	KIAA0355	1,05
trinucleotide repeat containing 6A	TNRC6A	1,05

transforming growth factor beta 2; TGFB2 overlapping transcript 1	TGFB2; TGFB2-OT1	1,05
GRB2-related adaptor protein 2	GRAP2	1,05
actin filament associated protein 1-like 1	AFAP1L1	1,05
hydroxymethylbilane synthase	HMBS	1,05
tetratricopeptide repeat domain 30B	TTC30B	1,05
growth hormone 2	GH2	1,05
phytanoyl-CoA 2-hydroxylase	PHYH	1,05
membrane protein, palmitoylated 3	MPP3	1,05
deleted in esophageal cancer 1	DEC1	1,05
ring finger protein 141	RNF141	1,05
DnaJ (Hsp40) homolog, subfamily B, member 1	DNAJB1	1,05
basic leucine zipper transcription factor, ATF-like 3	BATF3	1,05
chromosome 9 open reading frame 85	C9orf85	1,05
zinc finger protein 134	ZNF134	1,05
even-skipped homeobox 1	EVX1	1,05
RNA binding motif protein 18	RBM18	1,05
fibrillarin	FBL	1,05
cytochrome P450, family 7, subfamily B, polypeptide 1	CYP7B1	1,05
NAD(P)H dehydrogenase, quinone 2	NQO2	1,05
DNA (cytosine-5-)-methyltransferase 3 alpha	DNMT3A	1,05
pecanex-like 4 (Drosophila)	PCNXL4	1,05
vesicle (multivesicular body) trafficking 1	VTA1	1,05
Wolf-Hirschhorn syndrome candidate 1	WHSC1	1,05
syncoilin, intermediate filament protein	SYNC	1,05
olfactory receptor, family 13, subfamily C, member 2	OR13C2	1,05
NOP2 nucleolar protein	NOP2	1,05
olfactory receptor, family 8, subfamily K, member 3 (gene/pseudogene)	OR8K3	1,05
chromosome 5 open reading frame 67	C5orf67	1,05
transcription factor 20 (AR1)	TCF20	1,05
uncharacterized DKFZp779M0652; Transcript Identified by AceView, Entrez Gene ID(s) 374387, RefSeq ID(s) NR_027134; novel transcript; uncharacterized DKFZp779M0652 [Source:EntrezGene;Acc:374387]	DKFZp779M0652; CTD-2210P24.4	1,05
LEM domain containing 1	LEMD1	1,05
platelet-derived growth factor alpha polypeptide	PDGFA	1,05
receptor (TNFRSF)-interacting serine-threonine kinase 1	RIPK1	1,05
N-terminal EF-hand calcium binding protein 2	NECAB2	1,05
Src homology 2 domain containing F	SHF	1,05
phosphatidylinositol 3-kinase, catalytic subunit type 3	PIK3C3	1,05
fucosyltransferase 8 (alpha (1,6) fucosyltransferase)	FUT8	1,05
zinc finger protein 621	ZNF621	1,05
membrane protein, palmitoylated 1	MPP1	1,05
calpain 8	CAPN8	1,05

REV3 like, DNA directed polymerase zeta catalytic subunit	REV3L	1,05
MYC induced nuclear antigen	MINA	1,05
family with sequence similarity 200, member B	FAM200B	1,05
family with sequence similarity 76, member A	FAM76A	1,05
Homo sapiens neuropeptide Y receptor Y4 (NPY4R), transcript variant 2, mRNA.; neuropeptide Y receptor type 4-like; Homo sapiens pancreatic polypeptide receptor 1, mRNA (cDNA clone MGC:116895 IMAGE:40005502), complete cds.; Homo sapiens pancreatic polypeptide receptor 1, mRNA (cDNA clone MGC:116897 IMAGE:40005506), complete cds.; novel transcript	NPY4R; CH17-360D5.1; PPYR1	1,05
Transcript Identified by AceView, Entrez Gene ID(s) 5108	PCM1	1,05
optineurin	OPTN	1,05
Transcript Identified by AceView, Entrez Gene ID(s) 64327	LMBR1	1,05
septin 12	sept-12	1,05
early growth response 3	EGR3	1,05
chromosome 11 open reading frame 68	C11orf68	1,05
carboxymethylenebutenolidase homolog (Pseudomonas)	CMBL	1,05
tumor susceptibility 101	TSG101	1,05
formin 1	FMN1	1,05
WD repeat domain 5B	WDR5B	1,05
serum/glucocorticoid regulated kinase family, member 3	SGK3	1,05
Ras association (RalGDS/AF-6) domain family member 1	RASSF1	1,05
cyclin-dependent kinase 16	CDK16	1,05
profilin 1	PFN1	1,05
fumarate hydratase	FH	1,05
calpastatin	CAST	1,05
epididymal peptidase inhibitor	EPPIN	1,05
5-nucleotidase domain containing 3	NT5DC3	1,05
Memczak2013 ANTISENSE, CDS, coding, INTERNAL best transcript NM_033054	MYO1G	1,05
mitochondrial ribosomal protein L55	MRPL55	1,05
zinc finger, DHHC-type containing 12	ZDHHC12	1,05
fidgetin	FIGN	1,05
long intergenic non-protein coding RNA 1587	LINC01587	1,05
lysine (K)-specific demethylase 5C	KDM5C	1,05
density-regulated protein	DENR	1,05
carcinoembryonic antigen-related cell adhesion molecule 8	CEACAM8	1,05
chaperonin containing TCP1, subunit 3 (gamma)	CCT3	1,05
DEAD (Asp-Glu-Ala-Asp) box polypeptide 55	DDX55	1,05
akirin 2	AKIRIN2	1,05
zinc finger, CCHC domain containing 24	ZCCHC24	1,05
olfactory receptor, family 1, subfamily L, member 3	OR1L3	1,05
olfactory receptor, family 5, subfamily M, member 8	OR5M8	1,05
coiled-coil domain containing 43	CCDC43	1,05
dual specificity phosphatase 16	DUSP16	1,05

N-deacetylase/N-sulfotransferase (heparan glucosaminyl) 3	NDST3	1,05
zinc finger, DHHC-type containing 3	ZDHHC3	1,05
clarin 1	CLRN1	1,05
F-box protein 42	FBXO42	1,05
protein prenyltransferase alpha subunit repeat containing 1	PTAR1	1,05
FEV (ETS oncogene family)	FEV	1,05
ubiquitin protein ligase E3 component n-recognin 5	UBR5	1,05
insulin-like growth factor binding protein, acid labile subunit	IGFALS	1,05
eukaryotic translation initiation factor 3, subunit L	EIF3L	1,05
developing brain homeobox 1	DBX1	1,05
5-hydroxytryptamine (serotonin) receptor 3D, ionotropic	HTR3D	1,05
SLX1 homolog A, structure-specific endonuclease subunit; SLX1 homolog B, structure-specific endonuclease subunit	SLX1A; SLX1B	1,05
IQ motif containing H	IQCH	1,05
PAP associated domain containing 5	PAPD5	1,05
vesicle associated membrane protein 8	VAMP8	1,05
cytochrome c oxidase assembly factor 7 (putative)	COA7	1,05
cold inducible RNA binding protein	CIRBP	1,05
solute carrier family 36, member 3	SLC36A3	1,05
sphingosine-1-phosphate receptor 5	S1PR5	1,05
enoyl-CoA delta isomerase 2	ECI2	1,05
ubiquitin conjugating enzyme E2I	UBE2I	1,05
TBC1 domain family, member 3B; TBC1 domain family, member 3H; TBC1 domain family, member 3I; TBC1 domain family, member 3F; TBC1 domain family, member 3G	TBC1D3B; TBC1D3H; TBC1D3I; TBC1D3F; TBC1D3G	1,05
potassium channel, inwardly rectifying subfamily J, member 4	KCNJ4	1,05
retinal degeneration 3	RD3	1,05
TGIF2-C20orf24 readthrough	TGIF2-C20orf24	1,05
phosphofructokinase, platelet	PFKP	1,05
heparan sulfate 6-O-sulfotransferase 1	HS6ST1	1,05
cilia and flagella associated protein 46	CFAP46	1,05
ER lipid raft associated 1	ERLIN1	1,05
folliculin interacting protein 1	FNIP1	1,05
endothelin converting enzyme 1	ECE1	1,05
heterogeneous nuclear ribonucleoprotein C (C1/C2)	HNRNPC	1,05
peptidylprolyl isomerase (cyclophilin)-like 6	PPIL6	1,05
olfactory receptor, family 10, subfamily G, member 4	OR10G4	1,05
AVL9 homolog (S. cerevisiase)	AVL9	1,05
mucin 2, oligomeric mucus/gel-forming	MUC2	1,05
F-box protein, helicase, 18	FBXO18	1,05
glucosidase, beta, acid 3 (gene/pseudogene)	GBA3	1,05
tubulin tyrosine ligase-like family member 2	TTLL2	1,05

PRKR interacting protein 1 (IL11 inducible)	PRKRIP1	1,05
gap junction protein gamma 3	GJC3	1,05
coiled-coil serine rich protein 1	CCSER1	1,05
integrin beta 3 binding protein (beta3-endonexin)	ITGB3BP	1,05
5-3 exoribonuclease 2	XRN2	1,05
flavin containing monooxygenase 3	FMO3	1,05
zinc finger CCCH-type containing 12A; microRNA 6732	ZC3H12A; MIR6732	1,05
lymphocyte antigen 6 complex, locus H	LY6H	1,05
N(alpha)-acetyltransferase 50, NatE catalytic subunit	NAA50	1,05
chromosome 7 open reading frame 65	C7orf65	1,05
Memczak2013 ANTISENSE, CDS, coding, INTERNAL best transcript NM_182962	BIRC3	1,05
signal peptide, CUB domain, EGF-like 1	SCUBE1	1,05
natriuretic peptide C	NPPC	1,05
protein tyrosine phosphatase, receptor type, A; vacuolar protein sorting 16 homolog (S. cerevisiae)	PTPRA; VPS16	1,05
NADH dehydrogenase (ubiquinone) complex I, assembly factor 4	NDUFAF4	1,05
IQ motif containing C	IQCC	1,05
dipeptidase 2	DPEP2	1,05
tubulin, gamma complex associated protein 5	TUBGCP5	1,05
synaptotagmin III	SYT3	1,05
novel transcript, sense overlapping TAF10; Transcript Identified by AceView, Entrez Gene ID(s) 6881	RP11-732A19.2; TAF10	1,05
family with sequence similarity 129, member A	FAM129A	1,05
CCR4-NOT transcription complex subunit 3	CNOT3	1,05
sestrin 1	SESN1	1,05
ribosomal protein, large, P0	RPLP0	1,05
neuroligin 2	NLGN2	1,05
myosin IH	MYO1H	1,05
glyceronephosphate O-acyltransferase	GNPAT	1,05
myotubularin related protein 14	MTMR14	1,05
ubiquitin specific peptidase 17-like family member 11; ubiquitin specific peptidase 17-like family member 18; ubiquitin specific peptidase 17-like family member 20	USP17L11; USP17L18; USP17L20	1,05
ADNP homeobox 2	ADNP2	1,05
zinc finger protein 274	ZNF274	1,05
sarcospan	SSPN	1,05
NADPH oxidase 4	NOX4	1,05
chromosome 22 open reading frame 24	C22orf24	1,05
CASK interacting protein 2	CASKIN2	1,05
chromosome 11 open reading frame 1	C11orf1	1,05
WSC domain containing 2	WSCD2	1,05
olfactory receptor, family 4, subfamily K, member 13	OR4K13	1,05

tetraspanin 4	TSPAN4	1,05
ASB16 antisense RNA 1	ASB16-AS1	1,05
ribosomal protein L24	RPL24	1,05
ADAM metallopeptidase domain 22	ADAM22	1,05
zinc finger protein 474	ZNF474	1,05
Nedd4 family interacting protein 1	NDFIP1	1,05
inositol 1,4,5-trisphosphate receptor, type 3	ITPR3	1,05
platelet-activating factor acetylhydrolase 1b, catalytic subunit 2 (30kDa)	PAFAH1B2	1,05
chromosome 7 open reading frame 71	C7orf71	1,05
mitochondrial translational release factor 1	MTRF1	1,05
olfactory receptor, family 4, subfamily E, member 2	OR4E2	1,05
spectrin repeat containing, nuclear envelope family member 3	SYNE3	1,05
amyloid beta (A4) precursor protein-binding, family A, member 2	APBA2	1,05
von Willebrand factor A domain containing 5B1	VWA5B1	1,05
apoptotic chromatin condensation inducer 1	ACIN1	1,05
chromosome 11 open reading frame 57	C11orf57	1,05
olfactomedin like 2A	OLFML2A	1,05
testis development related protein	TDRP	1,05
CREB/ATF bZIP transcription factor	CREBZF	1,05
Yip1 domain family member 7	YIPF7	1,05
mannose-binding lectin (protein A) 1, pseudogene; BMS1 ribosome biogenesis factor pseudogene 21	MBL1P; BMS1P21	1,05
radial spoke head 10 homolog B2 (Chlamydomonas); radial spoke head 10 homolog B (Chlamydomonas)	RSPH10B2; RSPH10B	1,05
Jeck2013 ALT_ACCEPTOR, ALT_DONOR, coding, INTERNAL, intronic best transcript NM_001014797	KCNMA1	1,05
succinate dehydrogenase complex assembly factor 2	SDHAF2	1,05
small integral membrane protein 21	SMIM21	1,05
serpin peptidase inhibitor, clade B (ovalbumin), member 4	SERPINB4	1,05
zinc finger protein 497; alpha-1-B glycoprotein	ZNF497; A1BG	1,05
tigger transposable element derived 7	TIGD7	1,05
DNAJC25-GNG10 readthrough	DNAJC25-GNG10	1,05
collagen, type IV, alpha 6	COL4A6	1,05
RAB32, member RAS oncogene family	RAB32	1,05
succinate dehydrogenase complex assembly factor 3	SDHAF3	1,05
nucleus accumbens associated 1, BEN and BTB (POZ) domain containing	NACC1	1,05
numb homolog (Drosophila)	NUMB	1,05
meiosis 1 associated protein	M1AP	1,05
chromosome 10 open reading frame 55	C10orf55	1,05
wingless-type MMTV integration site family, member 5A	WNT5A	1,05
activating transcription factor 6 beta	ATF6B	1,05

annexin A8	ANXA8	1,05
copine VI (neuronal)	CPNE6	1,05
prostate and breast cancer overexpressed 1	PBOV1	1,05
surfeit 4	SURF4	1,05
phosphatidylinositol glycan anchor biosynthesis class H	PIGH	1,05
zinc finger protein 180	ZNF180	1,05
calpain, small subunit 1	CAPNS1	1,05
proliferation-associated 2G4	PA2G4	1,05
methylcrotonoyl-CoA carboxylase 1	MCCC1	1,05
transcription factor AP-2 epsilon (activating enhancer binding protein 2 epsilon)	TFAP2E	1,05
carnosine N-methyltransferase 1	CARNMT1	1,05
proline rich 19	PRR19	1,05
translocase of inner mitochondrial membrane 8 homolog B (yeast)	TIMM8B	1,05
frequently rearranged in advanced T-cell lymphomas 2	FRAT2	1,05
zinc finger protein 493	ZNF493	1,05
cytochrome P450, family 4, subfamily F, polypeptide 22	CYP4F22	1,05
dihydrouridine synthase 2	DUS2	1,05
synaptophysin-like 2	SYPL2	1,05
regulator of G-protein signaling 13	RGS13	1,05
olfactory receptor, family 2, subfamily L, member 3	OR2L3	1,05
intraflagellar transport 20	IFT20	1,05
mediator complex subunit 17	MED17	1,05
forkhead box A1	FOXA1	1,05
ligase III, DNA, ATP-dependent	LIG3	1,05
FK506 binding protein 11; ADP-ribosylation factor 3	FKBP11; ARF3	1,05
BMS1 ribosome biogenesis factor pseudogene 20; immunoglobulin lambda variable 1-51; immunoglobulin lambda variable 5-52	BMS1P20; IGLV1-51; IGLV5-52	1,05
receptor (chemosensory) transporter protein 5 (putative)	RTP5	1,05
KxDL motif containing 1	KXD1	1,05
ring finger protein, transmembrane 2	RNFT2	1,05
pellino E3 ubiquitin protein ligase 1	PELI1	1,05
N-acetylneuraminic acid phosphatase	NANP	1,05
DNA fragmentation factor, 45kDa, alpha polypeptide	DFFA	1,05
claudin 16	CLDN16	1,05
zinc finger protein 420	ZNF420	1,05
aldo-keto reductase family 1, member A1 (aldehyde reductase)	AKR1A1	1,05
LIM domain only 4	LMO4	1,05
lectin, mannose-binding, 1 like	LMAN1L	1,05
mitochondrial ribosomal protein S12	MRPS12	1,05
peroxisomal biogenesis factor 11 beta	PEX11B	1,05
arylsulfatase D	ARSD	1,05
inositol(myo)-1(or 4)-monophosphatase 2	IMPA2	1,05

chromodomain protein, Y-like	CDYL	1,05
general transcription factor IIIC subunit 2	GTF3C2	1,05
POP4 homolog, ribonuclease P/MRP subunit	POP4	1,05
malonyl-CoA decarboxylase	MLYCD	1,05
chaperonin containing TCP1, subunit 5 (epsilon)	CCT5	1,05
endogenous Bornavirus-like nucleoprotein 2	EBLN2	1,05
lysophospholipase I	LYPLA1	1,05
COL18A1 antisense RNA 1	COL18A1-AS1	1,05
cullin 1	CUL1	1,05
zinc finger protein 692	ZNF692	1,05
transmembrane protein 5	TMEM5	1,05
molybdenum cofactor sulfurase	MOCOS	1,05
golgin A8 family, member S	GOLGA8S	1,05
ABI family, member 3 (NESH) binding protein	ABI3BP	1,05
cleavage and polyadenylation specific factor 1; microRNA 939; microRNA 1234; microRNA 6849	CPSF1; MIR939; MIR1234; MIR6849	1,05
UDP-glucose 6-dehydrogenase	UGDH	1,05
LDL receptor related protein 1	LRP1	1,05
maestro heat-like repeat family member 9	MROH9	1,05
nuclear receptor subfamily 4, group A, member 1	NR4A1	1,05
CDC42 binding protein kinase gamma (DMPK-like)	CDC42BPG	1,05
biotinidase	BTD	1,05
WD repeat domain 62	WDR62	1,05
phosphatidylinositol glycan anchor biosynthesis class B	PIGB	1,05
ribosomal protein S20; small nucleolar RNA, C/D box 54	RPS20; SNORD54	1,05
DnaJ (Hsp40) homolog, subfamily C, member 27	DNAJC27	1,05
coiled-coil domain containing 125	CCDC125	1,05
olfactory receptor, family 4, subfamily A, member 16	OR4A16	1,05
solute carrier family 1 (high affinity aspartate/glutamate transporter), member 6	SLC1A6	1,05
acid phosphatase, testicular	ACPT	1,05
RAN binding protein 3-like	RANBP3L	1,05
leucine-zipper-like transcription regulator 1	LZTR1	1,05
ribosomal protein S6 kinase, 90kDa, polypeptide 1	RPS6KA1	1,05
carbonic anhydrase XII	CA12	1,05
tektin 5	TEKT5	1,05
slit guidance ligand 2	SLIT2	1,05
meningioma (disrupted in balanced translocation) 1	MN1	1,05
kelch domain containing 8A	KLHDC8A	1,05
exportin 6	XPO6	1,05
nuclear cap binding protein subunit 1	NCBP1	1,05
lymphotoxin alpha	LTA	1,05

acyl-CoA thioesterase 12	ACOT12	1,05
interleukin 1 receptor associated kinase 1 binding protein 1	IRAK1BP1	1,05
C-type lectin domain family 1, member A	CLEC1A	1,05
acetyl-CoA acyltransferase 1	ACAA1	1,05
ABL proto-oncogene 1, non-receptor tyrosine kinase	ABL1	1,05
mex-3 RNA binding family member A	MEX3A	1,05
pleckstrin homology-like domain, family B, member 3	PHLDB3	1,05
decapping mRNA 1B	DCP1B	1,05
zinc finger protein 136	ZNF136	1,05
vestigial-like family member 2	VGLL2	1,05
galactose-3-O-sulfotransferase 4	GAL3ST4	1,05
C2 calcium-dependent domain containing 2	C2CD2	1,05
family with sequence similarity 101, member A	FAM101A	1,05
protocadherin 17	PCDH17	1,05
bystin-like	BYSL	1,05
dual specificity phosphatase 16	DUSP16	1,05
A-Raf proto-oncogene, serine/threonine kinase	ARAF	1,05
histone cluster 1, H2bl	HIST1H2BL	1,05
succinate dehydrogenase complex subunit B, iron sulfur (Ip)	SDHB	1,05
homeobox C9	HOXC9	1,05
GID complex subunit 8	GID8	1,05
zinc finger protein 70	ZNF70	1,05
peptidylprolyl isomerase (cyclophilin)-like 3	PPIL3	1,05
forkhead box D4-like 5	FOXD4L5	1,05
anaphase promoting complex subunit 7	ANAPC7	1,05
nucleoredoxin	NXN	1,05
zinc finger CCCH-type containing 10	ZC3H10	1,05
olfactory receptor, family 4, subfamily K, member 17	OR4K17	1,05
TYMS opposite strand	TYMSOS	1,05
FAST kinase domains 3	FASTKD3	1,05
zinc finger (CCCH type), RNA binding motif and serine/arginine rich 2	ZRSR2	1,05
tetraspanin 5	TSPAN5	1,05
clock circadian regulator	CLOCK	1,05
ubiquitin specific peptidase 22	USP22	1,05
cyclin B1 interacting protein 1, E3 ubiquitin protein ligase; small nucleolar RNA, C/D box 126	CCNB1IP1; SNORD126	1,05
mitochondrial ribosomal protein S7	MRPS7	1,05
chromosome 17 open reading frame 82	C17orf82	1,05
caspase recruitment domain family, member 8	CARD8	1,05
SAP30 binding protein	SAP30BP	1,05
SPATA31 subfamily C, member 2	SPATA31C2	1,05
chloride channel CLIC-like 1	CLCC1	1,05
forkhead box P3	FOXP3	1,05
bromodomain and WD repeat domain containing 3	BRWD3	1,05

dihydropyrimidinase-like 5	DPYSL5	1,05
ataxin 2-like	ATXN2L	1,05
lymphocyte antigen 6 complex, locus K	LY6K	1,05
mitogen-activated protein kinase-activated protein kinase 5; ADAM metallopeptidase domain 1A (pseudogene)	MAPKAPK5; ADAM1A	1,05
ATM interactor	ATMIN	1,05
mannosidase, beta A, lysosomal	MANBA	1,05
metallothionein 2A	MT2A	1,05
ribosomal protein L13; small nucleolar RNA, C/D box 68	RPL13; SNORD68	1,05
iron-sulfur cluster assembly enzyme	ISCU	1,05
zinc finger protein 780A	ZNF780A	1,05
deafness, autosomal dominant 5	DFNA5	1,05
long intergenic non-protein coding RNA 92	LINC00092	1,05
E2F transcription factor 3	E2F3	1,05
guanine nucleotide binding protein (G protein), alpha activating activity polypeptide O	GNAO1	1,05
acrosin	ACR	1,05
LDL receptor related protein 6	LRP6	1,05
pleckstrin homology-like domain, family A, member 1	PHLDA1	1,05
abhydrolase domain containing 8	ABHD8	1,05
solute carrier family 6 (neurotransmitter transporter), member 4	SLC6A4	1,05
SWI/SNF related, matrix associated, actin dependent regulator of chromatin, subfamily d, member 1	SMARCD1	1,05
microfibrillar associated protein 2	MFAP2	1,05
MARVEL domain containing 1	MARVELD1	1,05
erb-b2 receptor tyrosine kinase 3	ERBB3	1,05
forkhead box F2	FOXF2	1,05
WW domain binding protein 1	WBP1	1,05
sepiapterin reductase (7,8-dihydrobiopterin:NADP+ oxidoreductase)	SPR	1,05
protein phosphatase 1, regulatory subunit 15B	PPP1R15B	1,05
EXOC3 antisense RNA 1	EXOC3-AS1	1,05
DDHD domain containing 1	DDHD1	1,05
prostate and testis expressed 1	PATE1	1,05
kinesin family member 20B	KIF20B	1,05
leucine rich repeat and fibronectin type III domain containing 4	LRFN4	1,05
LIM homeobox transcription factor 1, alpha	LMX1A	1,05
plakophilin 2	PKP2	1,05
Werner helicase interacting protein 1	WRNIP1	1,05
forkhead box O1	FOXO1	1,05
eukaryotic translation initiation factor 2D	EIF2D	1,05
Wilms tumor 1 associated protein	WTAP	1,05
phospholipase C, beta 3 (phosphatidylinositol-specific)	PLCB3	1,05

adhesion G protein-coupled receptor E3	ADGRE3	1,05
NADH dehydrogenase (ubiquinone) 1 alpha subcomplex, 3, 9kDa	NDUFA3	1,05
butyrobetaine (gamma), 2-oxoglutarate dioxygenase (gamma-butyrobetaine hydroxylase) 1	BBOX1	1,05
zinc finger protein 679	ZNF679	1,05
transient receptor potential cation channel, subfamily C, member 6	TRPC6	1,05
poly(A) binding protein, nuclear 1	PABPN1	1,05
mediator complex subunit 19	MED19	1,05
SEC24 homolog C, COPII coat complex component	SEC24C	1,05
protein tyrosine kinase 2	PTK2	1,05
olfactory receptor, family 6, subfamily B, member 3	OR6B3	1,05
spermidine synthase	SRM	1,05
NADH dehydrogenase (ubiquinone) 1 beta subcomplex, 9, 22kDa	NDUFB9	1,05
dynein, axonemal, heavy chain 9	DNAH9	1,05
inositol 1,4,5-trisphosphate receptor, type 1	ITPR1	1,05
glutaminyl-tRNA synthase (glutamine-hydrolyzing)-like 1	QRSL1	1,05
WW and C2 domain containing 1	WWC1	1,05
protein tyrosine phosphatase, receptor type, D	PTPRD	1,05
family with sequence similarity 180, member B	FAM180B	1,05
adrenomedullin 2	ADM2	1,05
transmembrane protein 41A	TMEM41A	1,05
solute carrier family 30 (zinc transporter), member 8	SLC30A8	1,05
sphingosine-1-phosphate phosphatase 1	SGPP1	1,05
nuclear factor of kappa light polypeptide gene enhancer in B-cells 2 (p49/p100)	NFKB2	1,05
G protein-coupled receptor 150	GPR150	1,05
proline rich 32	PRR32	1,05
MHC class I polypeptide-related sequence A	MICA	1,05
novel transcript, sense overlapping TTC17; Transcript Identified by AceView, Entrez Gene ID(s) 55761	RP11-484D2.4; TTC17	1,05
olfactory receptor, family 1, subfamily D, member 2	OR1D2	1,05
coiled-coil domain containing 28A	CCDC28A	1,05
aspartyl aminopeptidase	DNPEP	1,05
olfactory receptor, family 51, subfamily B, member 5	OR51B5	1,05
spectrin, beta, non-erythrocytic 1	SPTBN1	1,05
mitochondrial ribosomal protein S23	MRPS23	1,05
POTE ankyrin domain family member D-like; Synthetic construct Homo sapiens clone IMAGE:100066427, MGC:195564 POTE ankyrin domain family, member D (POTED) mRNA, encodes complete protein.; uncharacterized protein LOC100288966 [Source:RefSeq peptide;Acc:NP_001244291]	LOC100288966; POTED; BX072566.1	1,05
zinc finger homeobox 4	ZFHX4	1,05
PRAME family member 6; PRAME family member 5	PRAMEF6; PRAMEF5	1,05

chemokine (C-C motif) ligand 21	CCL21	1,05
endoplasmic reticulum oxidoreductase beta	ERO1B	1,05
Scm-like with four mbt domains 2	SFMBT2	1,05
ubiquitin-conjugating enzyme E2Q family member 1	UBE2Q1	1,05
interferon regulatory factor 4	IRF4	1,05
NFKB activating protein-like	NKAPL	1,05
keratin associated protein 9-3	KRTAP9-3	1,05
bestrophin 3	BEST3	1,05
guanine nucleotide binding protein (G protein), alpha inhibiting activity polypeptide 2	GNAI2	1,05
cell division cycle 26	CDC26	1,05
kinesin family member 21A	KIF21A	1,05
zinc finger and SCAN domain containing 16	ZSCAN16	1,05
pescadillo ribosomal biogenesis factor 1	PES1	1,05
developmental pluripotency associated 2	DPPA2	1,05
neuroblastoma breakpoint family, member 4	NBPF4	1,05
TBC1 domain family, member 2B	TBC1D2B	1,05
coagulation factor X	F10	1,05
sema domain, transmembrane domain (TM), and cytoplasmic domain, (semaphorin) 6B	SEMA6B	1,05
solute carrier family 2 (facilitated glucose transporter), member 6 [Source:HGNC Symbol;Acc:HGNC:11011]	SLC2A6	1,05
zinc finger, MYND-type containing 19	ZMYND19	1,05
uncharacterized LOC101928161; Salzman2013 ANTISENSE, coding, INTERNAL, intronic, OVERLAPTX best transcript NM_207363; novel transcript, antisense to NCKAP5; Transcript Identified by AceView	LOC101928161; AC010890.1; NCKAP5; spawfa	1,05
ALS2 C-terminal like	ALS2CL	1,05
cartilage oligomeric matrix protein	COMP	1,05
Y box binding protein 1	YBX1	1,05
energy homeostasis associated	ENHO	1,05
RELT-like 1	RELL1	1,05
myosin IF	MYO1F	1,05
capping protein (actin filament) muscle Z-line, alpha 2	CAPZA2	1,05
coiled-coil domain containing 81	CCDC81	1,05
phosphatase domain containing, paladin 1	PALD1	1,05
ubiquitin-conjugating enzyme E2O	UBE2O	1,05
kringle containing transmembrane protein 2	KREMEN2	1,05
ribosomal protein S6 kinase-like 1	RPS6KL1	1,05
TM2 domain containing 2	TM2D2	1,05
REX1, RNA exonuclease 1 homolog-like 1, pseudogene; REX1, RNA exonuclease 1 homolog-like 3, pseudogene	REXO1L1P; REXO1L3P	1,05
cleavage and polyadenylation specific factor 3-like; microRNA 6727	CPSF3L; MIR6727	1,05
sphingomyelin synthase 2	SGMS2	1,05

transcription factor-like 5 (basic helix-loop-helix)	TCFL5	1,05
ribosomal protein S25	RPS25	1,05
cystatin SA	CST2	1,05
biglycan	BGN	1,05
PDZ and LIM domain 5	PDLIM5	1,05
src kinase associated phosphoprotein 2	SKAP2	1,05
paired box 8	PAX8	1,05
histone cluster 1, H1c	HIST1H1C	1,05
CLPTM1-like	CLPTM1L	1,05
Transcript Identified by AceView, Entrez Gene ID(s) 8089	YEATS4	1,05
inhibitor of growth family member 5	ING5	1,05
phosphatidylinositol-4-phosphate 5-kinase, type I, gamma	PIP5K1C	1,05
double homeobox 5	DUX5	1,05
signal transducing adaptor family member 1	STAP1	1,05
contactin 4	CNTN4	1,05
GTP binding protein overexpressed in skeletal muscle	GEM	1,05
olfactory receptor, family 10, subfamily AD, member 1	OR10AD1	1,05
NIPA-like domain containing 2	NIPAL2	1,05
phosphorylase, glycogen, liver	PYGL	1,05
DiGeorge syndrome critical region gene 14	DGCR14	1,05
heterogeneous nuclear ribonucleoprotein H3 (2H9)	HNRNPH3	1,05
THO complex 3 pseudogene; Homo sapiens THO complex 3, mRNA (cDNA clone MGC:5469 IMAGE:3451612), complete cds.; Homo sapiens THO complex 3, mRNA (cDNA clone MGC:87233 IMAGE:5262880), complete cds.; Salzman2013 ANNOTATED, INTERNAL, ncRNA, OVERLAPTX, OVEXON best transcript NR_003615; Transcript Identified by AceView, Entrez Gene ID(s) 728554	LOC728554; THOC3	1,05
uncharacterized LOC100133091; Salzman2013 ANNOTATED, INTERNAL, ncRNA, OVEXON best transcript NR_029411; Transcript Identified by AceView, Entrez Gene ID(s) 554248; 100133091, RefSeq ID(s) NR_029411	LOC100133091	1,05
major facilitator superfamily domain containing 8	MFSD8	1,05
transcription elongation factor A (SII)-like 7	TCEAL7	1,05
zinc finger with KRAB and SCAN domains 7	ZKSCAN7	1,05
solute carrier family 25 (mitochondrial iron transporter), member 37	SLC25A37	1,05
insulin like growth factor binding protein 7	IGFBP7	1,05
podocan-like 1	PODNL1	1,05
BCL2-like 14 (apoptosis facilitator)	BCL2L14	1,05
forkhead box P4; microRNA 4641	FOXP4; MIR4641	1,05
chromosome 8 open reading frame 89	C8orf89	1,05
cell division cycle 37; microRNA 1181	CDC37; MIR1181	1,05

general transcription factor IIH subunit 2; general transcription factor IIH subunit 2B (pseudogene)	GTF2H2; GTF2H2B	1,05
zinc finger and BTB domain containing 6	ZBTB6	1,05
defensin, beta 127	DEFB127	1,05
solute carrier family 36 (proton/amino acid symporter), member 1	SLC36A1	1,05
RAB, member of RAS oncogene family-like 2A	RABL2A	1,05
transmembrane protein 109	TMEM109	1,05
TBC1 domain family, member 31	TBC1D31	1,05
ATPase, Na+/K+ transporting, beta 3 polypeptide	ATP1B3	1,05
zinc finger protein 784	ZNF784	1,05
StAR-related lipid transfer domain containing 13	STARD13	1,05
metallothionein 1F	MT1F	1,05
mannosidase, alpha, class 1A, member 2	MAN1A2	1,05
T-box 10	TBX10	1,05
mitochondrial ribosomal protein L34	MRPL34	1,05
tetratricopeptide repeat and ankyrin repeat containing 1	TRANK1	1,05
mirror-image polydactyly 1	MIPOL1	1,05
glycerophosphodiester phosphodiesterase domain containing 3	GDPD3	1,05
RAB19, member RAS oncogene family	RAB19	1,05
collagen, type VI, alpha 6	COL6A6	1,05
maturin, neural progenitor differentiation regulator homolog (Xenopus)	MTURN	1,05
microtubule-associated protein 7	MAP7	1,05
olfactory receptor, family 14, subfamily J, member 1	OR14J1	1,05
cathepsin A	CTSA	1,05
doublecortin domain containing 2C	DCDC2C	1,05
Transcript Identified by AceView, Entrez Gene ID(s) 54874	FNBP1L	1,05
dynactin 1	DCTN1	1,05
protein arginine methyltransferase 9	PRMT9	1,05
uncharacterized LOC100131303; novel transcript, antisense to DUS2L	LOC100131303; CTC-479C5.6	1,05
X-ray repair complementing defective repair in Chinese hamster cells 2	XRCC2	1,05
dynein, axonemal, intermediate chain 2	DNAI2	1,05
chromosome 9 open reading frame 16	C9orf16	1,05
ring finger protein 4	RNF4	1,05
RANBP2-like and GRIP domain containing 5; RANBP2-like and GRIP domain containing 8	RGPD5; RGPD8	1,05
ATPase, Ca++ transporting, plasma membrane 4	ATP2B4	1,05
myeloid cell nuclear differentiation antigen	MNDA	1,05
Transcript Identified by AceView, Entrez Gene ID(s) 23122	CLASP2	1,05
mitochondrial ribosomal protein S9	MRPS9	1,04
speedy/RINGO cell cycle regulator family member C	SPDYC	1,04
cysteine rich with EGF-like domains 1	CRELD1	1,04

tetratricopeptide repeat domain 24	TTC24	1,04
mitogen-activated protein kinase kinase 4; microRNA 744	MAP2K4; MIR744	1,04
stress-associated endoplasmic reticulum protein 1	SERP1	1,04
dynactin 6	DCTN6	1,04
Transcript Identified by AceView, Entrez Gene ID(s) 100133204; Salzman2013 ANTISENSE, CDS, coding, downstream_end, intronic, OVCODE, OVERLAPTX, OVEXON, UTR3 best transcript NM_032303; chromosome 9 open reading frame 147; chromosome 9 open reading frame 147 [Source:HGNC Symbol;Acc:HGNC:31438]	C9orf147; HSDL2	1,04
peroxisome proliferator-activated receptor delta	PPARD	1,04
olfactory receptor, family 52, subfamily J, member 3	OR52J3	1,04
olfactory receptor, family 51, subfamily H, member 1	OR51H1	1,04
solute carrier family 35 (CMP-sialic acid transporter), member A1	SLC35A1	1,04
zona pellucida binding protein 2	ZPBP2	1,04
Memczak2013 ANTISENSE, CDS, coding, INTERNAL best transcript NM_006184	NUCB1	1,04
acyl-CoA thioesterase 11	ACOT11	1,04
NOP10 ribonucleoprotein	NOP10	1,04
tripartite motif containing 36	TRIM36	1,04
solute carrier family 2 (facilitated glucose transporter), member 12	SLC2A12	1,04
zinc finger CCCH-type containing 7B	ZC3H7B	1,04
translocase of inner mitochondrial membrane 9 homolog (yeast)	TIMM9	1,04
fibroblast growth factor 10	FGF10	1,04
family with sequence similarity 149, member B1	FAM149B1	1,04
Memczak2013 ALT_ACCEPTOR, ALT_DONOR, INTERNAL, intronic, ncRNA best transcript NR_037774	STAM	1,04
vacuolar protein sorting 4 homolog B (S. cerevisiae)	VPS4B	1,04
inter-alpha-trypsin inhibitor heavy chain family, member 5	ITIH5	1,04
PRAME family member 8	PRAMEF8	1,04
endo/exonuclease (5-3), endonuclease G-like	EXOG	1,04
chromosome 1 open reading frame 226	C1orf226	1,04
serine/arginine-rich splicing factor 9	SRSF9	1,04
cyclin T1	CCNT1	1,04
stromal antigen 3	STAG3	1,04
insulin-like 3 (Leydig cell)	INSL3	1,04
DnaJ (Hsp40) homolog, subfamily B, member 14	DNAJB14	1,04
CDC like kinase 3	CLK3	1,04
trafficking protein particle complex 8	TRAPPC8	1,04
kinesin family member 18B	KIF18B	1,04

neuron specific gene family member 1; Neuron-specific protein family member 1 [Source:UniProtKB/Swiss-Prot;Acc:P42857]	NSG1; D4S234E	1,04
NADH dehydrogenase, subunit 1 (complex I)	ND1	1,04
connector enhancer of kinase suppressor of Ras 1	CNKSR1	1,04
S-antigen; retina and pineal gland (arrestin)	SAG	1,04
speckle-type POZ protein-like	SPOPL	1,04
tetraspanin 6	TSPAN6	1,04
magnesium-dependent phosphatase 1; NEDD8-MDP1 readthrough; neural precursor cell expressed, developmentally down-regulated 8; charged multivesicular body protein 4A	MDP1; NEDD8-MDP1; NEDD8; CHMP4A	1,04
PHD finger protein 5A	PHF5A	1,04
Jeck2013 ANTISENSE, coding, INTERNAL, intronic best transcript NM_016263	FZR1	1,04
septin 4	sept-04	1,04
transcription factor 3	TCF3	1,04
family with sequence similarity 159, member A	FAM159A	1,04
intraflagellar transport 80	IFT80	1,04
folate receptor 3 (gamma)	FOLR3	1,04
disrupted in renal carcinoma 1	DIRC1	1,04
hypoxia up-regulated 1	HYOU1	1,04
zinc finger protein 684	ZNF684	1,04
bridging integrator 1	BIN1	1,04
hypermethylated in cancer 1	HIC1	1,04
ankyrin 2, neuronal	ANK2	1,04
cofilin 1 (non-muscle)	CFL1	1,04
G protein-coupled receptor 151	GPR151	1,04
HIV-1 Tat interactive protein 2	HTATIP2	1,04
DnaJ (Hsp40) homolog, subfamily C, member 15	DNAJC15	1,04
Sys1 golgi trafficking protein	SYS1	1,04
NLR family, pyrin domain containing 1	NLRP1	1,04
family with sequence similarity 60, member A	FAM60A	1,04
coagulation factor II (thrombin)	F2	1,04
phospholipase D family, member 6	PLD6	1,04
eukaryotic translation initiation factor 3, subunit E	EIF3E	1,04
dachsous cadherin-related 2	DCHS2	1,04
COBW domain containing 3	CBWD3	1,04
ral guanine nucleotide dissociation stimulator-like 1	RGL1	1,04
asparagine synthetase domain containing 1	ASNSD1	1,04
polymerase (RNA) III (DNA directed) polypeptide D, 44kDa	POLR3D	1,04
G protein-coupled receptor kinase 7	GRK7	1,04
MORC family CW-type zinc finger 3	MORC3	1,04
G protein-coupled receptor 26	GPR26	1,04
chromosome 3 open reading frame 38	C3orf38	1,04
synapsin III	SYN3	1,04
ubiquinol-cytochrome c reductase complex III subunit VII	UQCRQ	1,04

cation channel, sperm associated 1	CATSPER1	1,04
junctophilin 1	JPH1	1,04
RFT1 homolog	RFT1	1,04
nucleic acid binding protein 2	NABP2	1,04
ring finger protein 14	RNF14	1,04
protein phosphatase 2, regulatory subunit A, beta	PPP2R1B	1,04
GTPase activating protein (SH3 domain) binding protein 1	G3BP1	1,04
myosin, heavy chain 15	MYH15	1,04
casein kinase 1, delta	CSNK1D	1,04
transcription factor B1, mitochondrial	TFB1M	1,04
inner membrane protein, mitochondrial	IMMT	1,04
glutamate receptor, ionotropic, delta 2	GRID2	1,04
interferon regulatory factor 8	IRF8	1,04
mitochondrial ribosomal protein L41	MRPL41	1,04
mitogen-activated protein kinase kinase 3	MAP2K3	1,04
NEDD4 binding protein 3	N4BP3	1,04
FBJ murine osteosarcoma viral oncogene homolog	FOS	1,04
ubiquitin specific peptidase 7 (herpes virus-associated)	USP7	1,04
zinc finger, MYM-type 2	ZMYM2	1,04
WD repeat domain 26; microRNA 4742	WDR26; MIR4742	1,04
collagen, type XVIII, alpha 1	COL18A1	1,04
apolipoprotein A-I binding protein	APOA1BP	1,04
janus kinase and microtubule interacting protein 1	JAKMIP1	1,04
adenylate cyclase 4	ADCY4	1,04
UPF3 regulator of nonsense transcripts homolog A (yeast)	UPF3A	1,04
uncharacterized LOC400661; novel transcript	LOC400661; AC034110.1	1,04
Homo sapiens TBC1 domain family, member 3H (TBC1D3H), mRNA.; Homo sapiens TBC1 domain family, member 3G (TBC1D3G), mRNA.; Homo sapiens TBC1 domain family, member 3I (TBC1D3I), mRNA.; Homo sapiens TBC1 domain family member-like (LOC101060389), mRNA.; Homo sapiens TBC1 domain family, member 3C, mRNA (cDNA clone MGC:87891 IMAGE:5165385), complete cds.; Homo sapiens TBC1 domain family, member 3, mRNA (cDNA clone MGC:87893 IMAGE:5742850), complete cds.	TBC1D3H; TBC1D3G; TBC1D3I; LOC101060389; TBC1D3C; TBC1D3	1,04
atypical chemokine receptor 4	ACKR4	1,04
mitochondrial ribosomal protein S15	MRPS15	1,04
3-hydroxyacyl-CoA dehydratase 2	HACD2	1,04
ring finger protein 13	RNF13	1,04
sosondowah ankyrin repeat domain family member D	SOWAHD	1,04
C1q and tumor necrosis factor related protein 4	C1QTNF4	1,04
TRAF3 interacting protein 2	TRAF3IP2	1,04

methylenetetrahydrofolate dehydrogenase (NADP+ dependent) 1, methenyltetrahydrofolate cyclohydrolase, formyltetrahydrofolate synthetase	MTHFD1	1,04
microtubule crosslinking factor 1	MTCL1	1,04
chromosome 11 open reading frame 42	C11orf42	1,04
maestro	MRO	1,04
transmembrane epididymal protein 1	TEDDM1	1,04
odorant binding protein 2B	OBP2B	1,04
PRAME family member 7; PRAME family member 8	PRAMEF7; PRAMEF8	1,04
maestro heat-like repeat family member 6	MROH6	1,04
mitogen-activated protein kinase kinase kinase 5	MAP3K5	1,04
homeobox and leucine zipper encoding	HOMEZ	1,04
ankyrin repeat domain 19, pseudogene	ANKRD19P	1,04
polypeptide N-acetylgalactosaminyltransferase 16	GALNT16	1,04
carboxylesterase 1 pseudogene 1; carboxylesterase 1	CES1P1; CES1	1,04
mitochondrial ribosomal protein L45	MRPL45	1,04
methyltransferase like 12; small nucleolar RNA, H/ACA box 57	METTL12; SNORA57	1,04
proteasome 26S subunit, ATPase 6	PSMC6	1,04
GATA binding protein 5	GATA5	1,04
mutY DNA glycosylase	MUTYH	1,04
zinc finger, MYND-type containing 12	ZMYND12	1,04
ankyrin repeat and death domain containing 1A	ANKDD1A	1,04
tudor domain containing 15	TDRD15	1,04
protocadherin beta 3	PCDHB3	1,04
small lysine-rich protein 1	SMKR1	1,04
sperm associated antigen 6	SPAG6	1,04
S100 calcium binding protein G	S100G	1,04
methionine adenosyltransferase II, alpha	MAT2A	1,04
UTP3, small subunit (SSU) processome component, homolog (S. cerevisiae)	UTP3	1,04
SREBF chaperone	SCAP	1,04
muscle RAS oncogene homolog	MRAS	1,04
transmembrane protein 229A	TMEM229A	1,04
nudix hydrolase 14	NUDT14	1,04
activating transcription factor 7; neuropeptide FF-amide peptide precursor	ATF7; NPFF	1,04
ubiquitin conjugating enzyme E2E 1	UBE2E1	1,04
F-box and WD repeat domain containing 9	FBXW9	1,04
phosphatidylinositol glycan anchor biosynthesis class C	PIGC	1,04
keratin 8, type II	KRT8	1,04
lipase A, lysosomal acid, cholesterol esterase	LIPA	1,04
wingless-type MMTV integration site family, member 10B	WNT10B	1,04
olfactory receptor, family 6, subfamily P, member 1	OR6P1	1,04

SET domain, bifurcated 2	SETDB2	1,04
sperm acrosome associated 7	SPACA7	1,04
ubiquitination factor E4B	UBE4B	1,04
solute carrier family 29 (equilibrative nucleoside transporter), member 4	SLC29A4	1,04
phytanoyl-CoA dioxygenase domain containing 1	PHYHD1	1,04
LIM domains containing 1	LIMD1	1,04
calbindin 2	CALB2	1,04
ATPase, H+ transporting, lysosomal V0 subunit a1; microRNA 5010	ATP6V0A1; MIR5010	1,04
olfactory receptor, family 6, subfamily B, member 2	OR6B2	1,04
long intergenic non-protein coding RNA 1600	LINC01600	1,04
Transcript Identified by AceView, Entrez Gene ID(s) 56895	AGPAT4	1,04
PRAME family member 11	PRAMEF11	1,04
hypoxia up-regulated 1	HYOU1	1,04
checkpoint kinase 1	CHEK1	1,04
heparanase	HPSE	1,04
3-oxoacyl-ACP synthase, mitochondrial	OXSM	1,04
carcinoembryonic antigen-related cell adhesion molecule 5	CEACAM5	1,04
growth factor independent 1 transcription repressor	GFI1	1,04
gephyrin	GPHN	1,04
cancer susceptibility candidate 5	CASC5	1,04
WAS/WASL interacting protein family, member 2	WIPF2	1,04
calcium regulated heat stable protein 1	CARHSP1	1,04
ras homolog family member F (in filopodia)	RHOF	1,04
APOC4-APOC2 readthrough (NMD candidate); apolipoprotein C-II	APOC4-APOC2; APOC2	1,04
coatomer protein complex subunit gamma 2; testis specific 13	COPG2; TSGA13	1,04
early growth response 1	EGR1	1,04
transmembrane protein 55B	TMEM55B	1,04
NECAP endocytosis associated 1	NECAP1	1,04
SPHK1 interactor, AKAP domain containing	SPHKAP	1,04
IgLON family member 5	IGLON5	1,04
signal transducing adaptor molecule (SH3 domain and ITAM motif) 2	STAM2	1,04
aryl hydrocarbon receptor nuclear translocator-like 2	ARNTL2	1,04
kinesin family member 6	KIF6	1,04
ankyrin repeat and sterile alpha motif domain containing 4B	ANKS4B	1,04
teneurin transmembrane protein 2	TENM2	1,04
WNT1 inducible signaling pathway protein 1	WISP1	1,04
adhesion regulating molecule 1	ADRM1	1,04
spindle and kinetochore associated complex subunit 3	SKA3	1,04
mutS homolog 2	MSH2	1,04
FERM domain containing 6	FRMD6	1,04

translocation associated membrane protein 1	TRAM1	1,04
cytochrome P450, family 2, subfamily D, polypeptide 6	CYP2D6	1,04
piwi-like RNA-mediated gene silencing 1	PIWIL1	1,04
cytochrome P450, family 2, subfamily C, polypeptide 18	CYP2C18	1,04
heterogeneous nuclear ribonucleoprotein C-like 1	HNRNPCL1	1,04
olfactory receptor, family 4, subfamily N, member 2	OR4N2	1,04
ubiquitin family domain containing 1	UBFD1	1,04
synaptotagmin XI	SYT11	1,04
phosphorylase kinase, gamma 1 (muscle)	PHKG1	1,04
pogo transposable element with ZNF domain	POGZ	1,04
component of oligomeric golgi complex 7	COG7	1,04
NIN1/RPN12 binding protein 1 homolog	NOB1	1,04
ribosomal protein S18	RPS18	1,04
SH3 domain containing ring finger 2	SH3RF2	1,04
SSSCA1 antisense RNA 1 (head to head)	SSSCA1-AS1	1,04
transient receptor potential cation channel, subfamily M, member 3	TRPM3	1,04
ATPase inhibitory factor 1	ATPIF1	1,04
proline-rich transmembrane protein 1	PRRT1	1,04
zinc finger protein 552	ZNF552	1,04
HEAT repeat containing 5B	HEATR5B	1,04
receptor (chemosensory) transporter protein 3	RTP3	1,04
integrator complex subunit 4	INTS4	1,04
CD83 molecule	CD83	1,04
ribosomal protein S25	RPS25	1,04
Fc fragment of IgG, high affinity Ia, receptor (CD64)	FCGR1A	1,04
phosphoribosylformylglycinamidine synthase	PFAS	1,04
perilipin 4	PLIN4	1,04
cleavage stimulation factor, 3 pre-RNA, subunit 2	CSTF2	1,04
ring finger protein 186	RNF186	1,04
secretion associated, Ras related GTPase 1B	SAR1B	1,04
NAC alpha domain containing	NACAD	1,04
reelin	RELN	1,04
vacuolar protein sorting 4 homolog A (S. cerevisiae)	VPS4A	1,04
Transcript Identified by AceView, Entrez Gene ID(s) 10485; novel transcript	C1orf61; RP11-98G7.1	1,04
LETM1 domain containing 1	LETMD1	1,04
zinc finger protein 449	ZNF449	1,04
olfactory receptor, family 5, subfamily T, member 1	OR5T1	1,04
ribosome binding factor A (putative); RBFA downstream neighbor (non-protein coding)	RBFA; RBFADN	1,04
transmembrane protein 151A	TMEM151A	1,04
transmembrane protein 127	TMEM127	1,04
methyltransferase like 21A	METTL21A	1,04
clusterin; microRNA 6843	CLU; MIR6843	1,04

S-antigen; retina and pineal gland (arrestin)	SAG	1,04
coiled-coil domain containing 84	CCDC84	1,04
Fc fragment of IgG, low affinity IIb, receptor (CD32)	FCGR2B	1,04
forkhead box I3	FOXI3	1,04
chromosome 10 open reading frame 120	C10orf120	1,04
phosducin	PDC	1,04
chemokine (C-C motif) receptor 7	CCR7	1,04
ribosomal protein L4; small nucleolar RNA, C/D box 16; small nucleolar RNA, C/D box 18A; small nucleolar RNA, C/D box 18B; small nucleolar RNA, C/D box 18C	RPL4; SNORD16; SNORD18A; SNORD18B; SNORD18C	1,04
ribosome production factor 1 homolog	RPF1	1,04
karyopherin alpha 5 (importin alpha 6)	KPNA5	1,04
methyltransferase like 5	METTL5	1,04
mitochondrial ribosomal protein L18	MRPL18	1,04
zinc finger protein 525	ZNF525	1,04
MAGE family member A1	MAGEA1	1,04
leucine-rich single-pass membrane protein 1	LSMEM1	1,04
RANBP2-like and GRIP domain containing 1; RANBP2-like and GRIP domain containing 2	RGPD1; RGPD2	1,04
cysteine-rich secretory protein 3	CRISP3	1,04
fatty acid binding protein 3, muscle and heart	FABP3	1,04
purinergic receptor P2Y, G-protein coupled, 8	P2RY8	1,04
Homo sapiens purinergic receptor P2Y, G-protein coupled, 8 (P2RY8), mRNA.; purinergic receptor P2Y, G-protein coupled, 8 [Source:HGNC Symbol;Acc:HGNC:15524]; Homo sapiens purinergic receptor P2Y, G-protein coupled, 8, mRNA (cDNA clone MGC:50878 IMAGE:5787268), complete cds.	P2RY8	1,04
Memczak2013 ALT_ACCEPTOR, ALT_DONOR, coding, INTERNAL, intronic best transcript NM_001170794	BACH2	1,04
T-box, brain, 1	TBR1	1,04
folate hydrolase 1B	FOLH1B	1,04
formin binding protein 1	FNBP1	1,04
fms-related tyrosine kinase 4	FLT4	1,04
keratin 16, type I	KRT16	1,04
chromosome 6 open reading frame 48; small nucleolar RNA, C/D box 52; small nucleolar RNA, C/D box 48	C6orf48; SNORD52; SNORD48	1,04
keratin associated protein 4-11	KRTAP4-11	1,04
catsper channel auxiliary subunit gamma	CATSPERG	1,04
endogenous retrovirus group V, member 1	ERVV-1	1,04
GLIS family zinc finger 2	GLIS2	1,04
dopamine receptor D4	DRD4	1,04
nuclear factor, erythroid 2-like 2	NFE2L2	1,04
nuclear receptor subfamily 4, group A, member 3	NR4A3	1,04

MIS12 kinetochore complex component	MIS12	1,04
coiled-coil domain containing 189	CCDC189	1,04
trafficking protein particle complex 11	TRAPPC11	1,04
RAB3 GTPase activating protein subunit 2 (non-catalytic); aurora kinase A pseudogene 1	RAB3GAP2; AURKAPS1	1,04
toll interacting protein	TOLLIP	1,04
WD repeat domain 59	WDR59	1,04
junctional sarcoplasmic reticulum protein 1	JSRP1	1,04
TSR2, 20S rRNA accumulation, homolog (S. cerevisiae)	TSR2	1,04
interleukin 21 receptor	IL21R	1,04
eukaryotic translation elongation factor 1 gamma; microRNA 3654	EEF1G; MIR3654	1,04
Niemann-Pick disease, type C1	NPC1	1,04
component of oligomeric golgi complex 6	COG6	1,04
ornithine decarboxylase 1; small nucleolar RNA, H/ACA box 80B	ODC1; SNORA80B	1,04
protein phosphatase 3, regulatory subunit B, alpha	PPP3R1	1,04
KIAA0319-like	KIAA0319L	1,04
lysine (K)-specific demethylase 8	KDM8	1,04
chromosome 1 open reading frame 95	C1orf95	1,04
MON1 secretory trafficking family member A	MON1A	1,04
proprotein convertase subtilisin/kexin type 7	PCSK7	1,04
transmembrane protein 134	TMEM134	1,04
ribosomal protein S6 kinase, 90kDa, polypeptide 6	RPS6KA6	1,04
putative novel transcript; Transcript Identified by AceView, Entrez Gene ID(s) 6048	XXbac-BPG154L12.4; RNF5	1,04
C-type lectin domain family 16, member A	CLEC16A	1,04
translocase of inner mitochondrial membrane 17 homolog B (yeast)	TIMM17B	1,04
heat shock 70kDa protein 4	HSPA4	1,04
mitochondrial ribosomal protein L16	MRPL16	1,04
olfactory receptor, family 7, subfamily A, member 17	OR7A17	1,04
centromere protein H	CENPH	1,04
transmembrane protein 130	TMEM130	1,04
lipoxygenase homology domains 1	LOXHD1	1,04
ribosomal protein L22-like 1	RPL22L1	1,04
synovial sarcoma, X breakpoint 4B; synovial sarcoma, X breakpoint 4	SSX4B; SSX4	1,04
signal peptide peptidase like 2A	SPPL2A	1,04
Rho GTPase activating protein 29	ARHGAP29	1,04
prenyl (decaprenyl) diphosphate synthase, subunit 1	PDSS1	1,04
Memczak2013 ANTISENSE, CDS, coding, INTERNAL, UTR3 best transcript NM_001080533	UNC119B	1,04
solute carrier family 25, member 48	SLC25A48	1,04

DnaJ (Hsp40) homolog, subfamily C, member 19	DNAJC19	1,04
GABA(A) receptor-associated protein	GABARAP	1,04
sterile alpha motif domain containing 10	SAMD10	1,04
uncharacterized LOC400558; Transcript Identified by AceView, Entrez Gene ID(s) 400558; novel transcript	LOC400558; RP11-46C24.3	1,04
tubulin tyrosine ligase-like family member 4	TTLL4	1,04
guanine nucleotide binding protein (G protein), gamma 2	GNG2	1,04
adenosine A3 receptor; transmembrane and immunoglobulin domain containing 3	ADORA3; TMIGD3	1,04
uncoupling protein 2 (mitochondrial, proton carrier)	UCP2	1,04
DEAH (Asp-Glu-Ala-His) box polypeptide 35	DHX35	1,04
splicing factor 3b subunit 2	SF3B2	1,04
CD19 molecule	CD19	1,04
transmembrane protein 41B	TMEM41B	1,04
suppression of tumorigenicity 7; ST7 overlapping transcript 4; ST7 overlapping transcript 3; microRNA 6132	ST7; ST7-OT4; ST7-OT3; MIR6132	1,04
zinc finger protein 564	ZNF564	1,04
tetraspanin 15	TSPAN15	1,04
protein kinase (cAMP-dependent, catalytic) inhibitor beta	PKIB	1,04
pre-B-cell leukemia homeobox 1	PBX1	1,04
D-2-hydroxyglutarate dehydrogenase	D2HGDH	1,04
extended synaptotagmin-like protein 1	ESYT1	1,04
kelch-like family member 32	KLHL32	1,04
Kazal-type serine peptidase inhibitor domain 1	KAZALD1	1,04
calcineurin-like EF-hand protein 1	CHP1	1,04
proline and serine rich 2	PROSER2	1,04
cadherin-related 23	CDH23	1,04
glycophorin E (MNS blood group)	GYPE	1,04
cell cycle associated protein 1	CAPRIN1	1,04
zona pellucida binding protein	ZPBP	1,04
sorting nexin 5	SNX5	1,04
phosphate cytidylyltransferase 2, ethanolamine	PCYT2	1,04
chromosome X open reading frame 57	CXorf57	1,04
family with sequence similarity 117, member B	FAM117B	1,04
lengsin, lens protein with glutamine synthetase domain	LGSN	1,04
ATP6V1G2-DDX39B readthrough (NMD candidate)	ATP6V1G2-DDX39B	1,04
actin binding LIM protein family, member 3	ABLIM3	1,04
BRISC and BRCA1 A complex member 1	BABAM1	1,04
HHIP-like 2	HHIPL2	1,04
motilin	MLN	1,04
SWI/SNF related, matrix associated, actin dependent regulator of chromatin, subfamily a, member 4	SMARCA4	1,04
sprouty RTK signaling antagonist 3	SPRY3	1,04

Homo sapiens sprouty RTK signaling antagonist 3 (SPRY3), transcript variant 1, mRNA.; sprouty homolog 3 (Drosophila) [Source:HGNC Symbol;Acc:HGNC:11271]; Homo sapiens sprouty homolog 3 (Drosophila), mRNA (cDNA clone MGC:97072 IMAGE:7262284), complete cds.; Homo sapiens sprouty homolog 3 (Drosophila), mRNA (cDNA clone MGC:103904 IMAGE:30915293), complete cds.; Homo sapiens sprouty homolog 3 (Drosophila), mRNA (cDNA clone MGC:104063 IMAGE:30915511), complete cds.	SPRY3	1,04
vanin 2	VNN2	1,04
proline rich 23C	PRR23C	1,04
pepsinogen 5, group I (pepsinogen A)	PGA5	1,04
nuclear receptor binding protein 1	NRBP1	1,04
PHD finger protein 8	PHF8	1,04
siah E3 ubiquitin protein ligase 1	SIAH1	1,04
transmembrane protein 189	TMEM189	1,04
YLP motif containing 1	YLPM1	1,04
F-box protein 11	FBXO11	1,04
SRY box 6; microRNA 6073	SOX6; MIR6073	1,04
mitochondrial ribosomal protein S18B	MRPS18B	1,04
serologically defined colon cancer antigen 8	SDCCAG8	1,04
nucleosome assembly protein 1-like 3	NAP1L3	1,04
RPL17-C18orf32 readthrough	RPL17-C18orf32	1,04
solute carrier family 9, subfamily B (NHA2, cation proton antiporter 2), member 2	SLC9B2	1,04
surfeit 1	SURF1	1,04
gastric intrinsic factor (vitamin B synthesis)	GIF	1,04
potassium channel, voltage gated Shab related subfamily B, member 1	KCNB1	1,04
opsin 3	OPN3	1,04
neuralized E3 ubiquitin protein ligase 1B	NEURL1B	1,04
ankyrin repeat domain 62	ANKRD62	1,04
CNDP dipeptidase 2 (metallopeptidase M20 family)	CNDP2	1,04
neurotrophin 3	NTF3	1,04
diacylglycerol lipase, beta	DAGLB	1,04
BBSome interacting protein 1	BBIP1	1,04
long intergenic non-protein coding RNA 1235	LINC01235	1,04
glycogen synthase kinase 3 beta	GSK3B	1,04
charged multivesicular body protein 2B	CHMP2B	1,04
metallothionein 1X	MT1X	1,04
solute carrier family 24 (sodium/potassium/calcium exchanger), member 1	SLC24A1	1,04
opiate receptor-like 1	OPRL1	1,04

RAS protein activator like 3	RASAL3	1,04
diacylglycerol kinase, delta 130kDa	DGKD	1,04
kelch-like family member 22	KLHL22	1,04
tec protein tyrosine kinase	TEC	1,04
insulin-like growth factor 2 receptor	IGF2R	1,04
ubiquitin protein ligase E3 component n-recognin 1	UBR1	1,04
dipeptidase 3	DPEP3	1,04
TBC1 domain family, member 8 (with GRAM domain)	TBC1D8	1,04
G protein-coupled receptor 137	GPR137	1,04
aquaporin 8	AQP8	1,04
chromosome 10 open reading frame 35	C10orf35	1,04
DAZ associated protein 1	DAZAP1	1,04
dymeclin	DYM	1,04
neogenin 1	NEO1	1,04
WD repeat domain 11	WDR11	1,04
minichromosome maintenance complex binding protein	MCMBP	1,04
translocase of outer mitochondrial membrane 34	TOMM34	1,04
keratin 32, type I	KRT32	1,04
olfactory receptor, family 14, subfamily I, member 1	OR14I1	1,04
zinc finger protein 652	ZNF652	1,04
dystrophin	DMD	1,04
nuclear RNA export factor 5	NXF5	1,04
phosphatase and actin regulator 2	PHACTR2	1,04
calmodulin regulated spectrin-associated protein family, member 3	CAMSAP3	1,04
Synthetic construct Homo sapiens clone IMAGE:100069019, MGC:199030 methyl-CpG binding domain protein 3-like 2 (MBD3L2) mRNA, encodes complete protein.; novel transcript	MBD3L2; CTB-25J19.1	1,04
double homeobox 4	DUX4	1,04
RAB35, member RAS oncogene family	RAB35	1,04
tetratricopeptide repeat domain 29	TTC29	1,04
hypoxia up-regulated 1	HYOU1	1,04
phosphatidylinositol 4-kinase type 2 alpha	PI4K2A	1,04
coronin, actin binding protein, 1A	CORO1A	1,04
ribosomal protein S27-like	RPS27L	1,04
NUT family member 2G	NUTM2G	1,04
paraneoplastic Ma antigen 1	PNMA1	1,04
nucleobindin 1	NUCB1	1,04
dedicator of cytokinesis 7	DOCK7	1,04
A kinase (PRKA) anchor protein 1	AKAP1	1,04
solute carrier family 25 (S-adenosylmethionine carrier), member 26	SLC25A26	1,04
glutamate receptor, ionotropic, N-methyl D-aspartate 2C	GRIN2C	1,04
guanine nucleotide binding protein (G protein), gamma 10	GNG10	1,04
G protein-coupled receptor kinase interacting ArfGAP 1	GIT1	1,04

hypoxia up-regulated 1	HYOU1	1,04
zinc finger protein 91	ZNF91	1,04
synaptotagmin X	SYT10	1,04
transcription elongation factor A (SII), 1	TCEA1	1,04
vacuolar protein sorting 45 homolog (S. cerevisiae)	VPS45	1,04
pyruvate dehydrogenase (lipoamide) alpha 1	PDHA1	1,04
secreted phosphoprotein 2	SPP2	1,04
hook microtubule-tethering protein 2	HOOK2	1,04
deltex 4, E3 ubiquitin ligase	DTX4	1,04
opioid binding protein/cell adhesion molecule-like	OPCML	1,04
olfactory receptor, family 8, subfamily H, member 1	OR8H1	1,04
adhesion G protein-coupled receptor B3	ADGRB3	1,04
chromosome X open reading frame 40B	CXorf40B	1,04
transmembrane protein 219	TMEM219	1,04
WD repeat and FYVE domain containing 1	WDFY1	1,04
NDC80 kinetochore complex component	NDC80	1,04
luteinizing hormone beta polypeptide	LHB	1,04
PRAME family member 12	PRAMEF12	1,04
coordinator of PRMT5, differentiation stimulator	COPRS	1,04
clathrin, heavy chain-like 1	CLTCL1	1,04
centrosomal protein 72kDa	CEP72	1,04
solute carrier family 31 (copper transporter), member 1	SLC31A1	1,04
SH2 domain containing 4B	SH2D4B	1,04
chromosome 14 open reading frame 93	C14orf93	1,04
coiled-coil domain containing 58	CCDC58	1,04
heat shock 70kDa protein 5 (glucose-regulated protein, 78kDa)	HSPA5	1,04
sphingosine-1-phosphate receptor 2	S1PR2	1,04
olfactory receptor, family 2, subfamily H, member 2	OR2H2	1,04
SH3-domain binding protein 5 (BTK-associated)	SH3BP5	1,04
leucine rich repeat containing 46	LRRC46	1,04
transmembrane BAX inhibitor motif containing 6	TMBIM6	1,04
harbinger transposase derived 1	HARBI1	1,04
zinc finger with KRAB and SCAN domains 1	ZKSCAN1	1,04
BCL2L2-PABPN1 readthrough	BCL2L2-PABPN1	1,04
regulator of chromosome condensation 2	RCC2	1,04
golgin A6 family-like 2	GOLGA6L2	1,04
eukaryotic translation initiation factor 2B, subunit 3 gamma, 58kDa	EIF2B3	1,04
anaphase promoting complex subunit 11	ANAPC11	1,04
opioid growth factor receptor-like 1	OGFRL1	1,04
Janus kinase 2	JAK2	1,04
chromodomain helicase DNA binding protein 4	CHD4	1,04
protein tyrosine phosphatase, non-receptor type 7	PTPN7	1,04
ribosomal protein S5	RPS5	1,04

zinc finger and BTB domain containing 18	ZBTB18	1,04
CCAAT/enhancer binding protein (C/EBP), delta	CEBPD	1,04
phosphatidylglycerophosphate synthase 1	PGS1	1,04
nucleoporin 35kDa	NUP35	1,04
zinc finger, AN1-type domain 5	ZFAND5	1,04
TATA box binding protein	TBP	1,04
transient receptor potential cation channel, subfamily C, member 3	TRPC3	1,04
cysteine-serine-rich nuclear protein 3	CSRNP3	1,04
polycystic kidney disease 2-like 1	PKD2L1	1,04
neuron navigator 2	NAV2	1,04
cyclin-dependent kinase 2 associated protein 2	CDK2AP2	1,04
mitochondrial ribosomal protein L1	MRPL1	1,04
sorting nexin 15	SNX15	1,04
complement component 1, q subcomponent-like 3	C1QL3	1,04
GDP-D-glucose phosphorylase 1	GDPGP1	1,04
striatin interacting protein 1	STRIP1	1,04
protein kinase C, gamma	PRKCG	1,04
olfactory receptor, family 5, subfamily F, member 1	OR5F1	1,04
epiplakin 1	EPPK1	1,04
Memczak2013 ANTISENSE, CDS, coding, INTERNAL best transcript NM_002649	PIK3CG	1,04
major facilitator superfamily domain containing 6	MFSD6	1,04
gamma-aminobutyric acid (GABA) A receptor, alpha 1	GABRA1	1,04
methyltransferase like 10	METTL10	1,04
dual specificity tyrosine-(Y)-phosphorylation regulated kinase 1B	DYRK1B	1,04
SRY box 17	SOX17	1,04
uncharacterized LOC101928034; Salzman2013 ANTISENSE, CDS, coding, INTERNAL, intronic, OVCODE, OVEXON, UTR5 best transcript NM_002960	LOC101928034; S100A3	1,04
ubiquitin specific peptidase 27, X-linked	USP27X	1,04
olfactory receptor, family 5, subfamily H, member 2	OR5H2	1,04
Jeck2013 ALT_ACCEPTOR, ALT_DONOR, coding, INTERNAL, intronic best transcript NM_017660	GATAD2A	1,04
mitochondrial ribosome-associated GTPase 1	MTG1	1,04
NLR family, pyrin domain containing 13	NLRP13	1,04
coiled-coil domain containing 169	CCDC169	1,04
F-box protein 44	FBXO44	1,04
calcium homeostasis modulator 1	CALHM1	1,04
Hermansky-Pudlak syndrome 1; microRNA 4685	HPS1; MIR4685	1,04
N(alpha)-acetyltransferase 25, NatB auxiliary subunit	NAA25	1,04
histidine triad nucleotide binding protein 2	HINT2	1,04
casein kinase 1, epsilon	CSNK1E	1,04
actin-like 9	ACTL9	1,04
stathmin-like 4	STMN4	1,04

cleavage stimulation factor, 3 pre-RNA, subunit 2, tau variant	CSTF2T	1,04
apolipoprotein B mRNA editing enzyme, catalytic polypeptide 1	APOBEC1	1,04
pseudouridylate synthase 7 (putative)	PUS7	1,04
novel transcript, antisense to C16orf53 and MVP; PAXIP1 associated glutamate-rich protein 1 [Source:HGNC Symbol;Acc:HGNC:28707]	AC009133.12; PAGR1	1,04
WD repeat containing planar cell polarity effector	WDPCP	1,04
solute carrier family 6 (neurotransmitter transporter), member 3	SLC6A3	1,04
isoprenylcysteine carboxyl methyltransferase	ICMT	1,04
regulatory factor X, 2 (influences HLA class II expression)	RFX2	1,04
intraflagellar transport 57	IFT57	1,04
WD repeat domain 83	WDR83	1,04
zinc finger protein 852	ZNF852	1,04
zinc finger protein 35	ZNF35	1,04
HRAS-like suppressor family, member 5	HRASLS5	1,04
transcription factor 20 (AR1)	TCF20	1,04
transcription factor 20 (AR1)	TCF20	1,04
cytoplasmic linker associated protein 2	CLASP2	1,04
fatty acid binding protein 4, adipocyte	FABP4	1,04
myosin, heavy chain 1, skeletal muscle, adult	MYH1	1,04
G-patch domain containing 1	GPATCH1	1,04
protein tyrosine phosphatase, non-receptor type 22 (lymphoid)	PTPN22	1,04
DAZ associated protein 2	DAZAP2	1,04
ubiquitously expressed prefoldin like chaperone	UXT	1,04
Zic family member 1	ZIC1	1,04
microsomal glutathione S-transferase 1	MGST1	1,04
kringle containing transmembrane protein 1	KREMEN1	1,04
hypoxia up-regulated 1	HYOU1	1,04
MDS1 and EVI1 complex locus	MECOM	1,04
COMM domain containing 7	COMMD7	1,04
LIM domain containing preferred translocation partner in lipoma	LPP	1,04
PDZ domain containing ring finger 4	PDZRN4	1,04
zinc finger protein 775	ZNF775	1,04
crystallin beta A1	CRYBA1	1,04
vesicle associated membrane protein 7	VAMP7	1,04
protein disulfide isomerase family A, member 6	PDIA6	1,04
family with sequence similarity 122C	FAM122C	1,04
programmed cell death 2-like	PDCD2L	1,04
leucine-rich repeat, immunoglobulin-like and transmembrane domains 3	LRIT3	1,04
oxidoreductase NAD-binding domain containing 1	OXNAD1	1,04
secretory carrier membrane protein 3	SCAMP3	1,04

zinc finger protein 761; tropomyosin 3 pseudogene 9	ZNF761; TPM3P9	1,04
insulin-like growth factor 1 (somatomedin C)	IGF1	1,04
polypeptide N-acetylgalactosaminyltransferase 12	GALNT12	1,04
nucleoporin like 2	NUPL2	1,04
MICAL-like 2	MICALL2	1,04
olfactory receptor, family 3, subfamily A, member 1	OR3A1	1,04
Salzman2013 ALT_ACCEPTOR, ALT_DONOR, coding, INTERNAL, intronic best transcript NM_133474	ZNF721	1,04
zinc finger protein 549	ZNF549	1,04
phosphodiesterase 4D interacting protein	PDE4DIP	1,04
CUGBP, Elav-like family member 6	CELF6	1,04
cell division cycle associated 7-like	CDCA7L	1,04
piwi-like RNA-mediated gene silencing 2	PIWIL2	1,04
nuclear mitotic apparatus protein 1	NUMA1	1,04
aryl hydrocarbon receptor nuclear translocator-like	ARNTL	1,04
2,3-bisphosphoglycerate mutase	BPGM	1,04
ribosomal protein L18a; small nucleolar RNA, H/ACA box 68	RPL18A; SNORA68	1,04
membrane bound transcription factor peptidase, site 1	MBTPS1	1,04
chromosome 3 open reading frame 30	C3orf30	1,04
glutamate receptor, ionotropic, N-methyl D-aspartate 2D	GRIN2D	1,04
Transcript Identified by AceView, Entrez Gene ID(s) 2066	ERBB4	1,04
castor zinc finger 1	CASZ1	1,04
granulin	GRN	1,04
proteasome subunit beta 10	PSMB10	1,04
acyl-CoA synthetase short-chain family member 2	ACSS2	1,04
Ellis van Creveld syndrome 2	EVC2	1,04
zinc finger protein 536	ZNF536	1,04
arylacetamide deacetylase-like 3	AADACL3	1,04
kinesin family member 15	KIF15	1,04
chromosome 22 open reading frame 31	C22orf31	1,04
ankyrin repeat and SOCS box containing 18	ASB18	1,04
aminoacyl tRNA synthetase complex-interacting multifunctional protein 2	AIMP2	1,04
prostaglandin D2 synthase 21kDa (brain)	PTGDS	1,04
CDC like kinase 2	CLK2	1,04
Transcript Identified by AceView, Entrez Gene ID(s) 80271	ITPKC	1,04
lysine (K)-specific demethylase 1A; microRNA 3115	KDM1A; MIR3115	1,04
dual specificity phosphatase 15	DUSP15	1,04
Memczak2013 ALT_ACCEPTOR, ALT_DONOR, coding, INTERNAL, intronic best transcript NM_006537	USP3	1,04
PRAME family member 33, pseudogene	PRAMEF33P	1,04
trafficking protein particle complex 1	TRAPPC1	1,04

smoothelin-like 1	SMTNL1	1,04
NIMA-related kinase 8	NEK8	1,04
SHC (Src homology 2 domain containing) transforming protein 3	SHC3	1,04
transcription elongation factor A (SII)-like 3	TCEAL3	1,04
coiled-coil domain containing 84	CCDC84	1,04
zinc finger protein 646	ZNF646	1,04
pinin, desmosome associated protein	PNN	1,04
Rap guanine nucleotide exchange factor 6	RAPGEF6	1,04
small proline-rich protein 3	SPRR3	1,04
cell division cycle 16	CDC16	1,04
WW domain containing adaptor with coiled-coil	WAC	1,04
golgi phosphoprotein 3-like	GOLPH3L	1,04
protein phosphatase 2, regulatory subunit B, alpha	PPP2R3A	1,04
B-cell CLL/lymphoma 3; microRNA 8085	BCL3; MIR8085	1,04
chromosome 11 open reading frame 53	C11orf53	1,04
MORN repeat containing 2	MORN2	1,04
olfactory receptor, family 8, subfamily G, member 5	OR8G5	1,04
kelch-like family member 14	KLHL14	1,04
tigger transposable element derived 2	TIGD2	1,04
TRIM39-RPP21 readthrough	TRIM39-RPP21	1,04
phospholipase D family, member 5	PLD5	1,04
leucine rich repeat containing 45	LRRC45	1,04
stathmin 2	STMN2	1,04
ribosomal protein S6 kinase, 90kDa, polypeptide 1	RPS6KA1	1,04
Transcript Identified by AceView, Entrez Gene ID(s) 2903	GRIN2A	1,04
inter-alpha-trypsin inhibitor heavy chain family, member 6	ITIH6	1,04
legumain	LGMN	1,04
chromosome 10 open reading frame 12	C10orf12	1,04
NADH dehydrogenase (ubiquinone) flavoprotein 3, 10kDa	NDUFV3	1,04
interleukin 2 receptor, alpha	IL2RA	1,04
glutathione S-transferase alpha 4	GSTA4	1,04
hyaluronoglucosaminidase 2	HYAL2	1,04
transmembrane protein 126A	TMEM126A	1,04
creatine kinase, mitochondrial 1B; creatine kinase, mitochondrial 1A	CKMT1B; CKMT1A	1,04
SH3 domain binding kinase family, member 2	SBK2	1,04
plasminogen receptor, C-terminal lysine transmembrane protein	PLGRKT	1,04
angiotensin II receptor, type 2	AGTR2	1,04
platelet-activating factor acetylhydrolase 2	PAFAH2	1,04
transmembrane protein 89	TMEM89	1,04
methyltransferase like 3	METTL3	1,04
septin 9	sept-09	1,04
endoplasmic reticulum-golgi intermediate compartment 1	ERGIC1	1,04
apolipoprotein H (beta-2-glycoprotein I)	APOH	1,04

dual specificity tyrosine-(Y)-phosphorylation regulated kinase 1B	DYRK1B	1,04
UXT antisense RNA 1	UXT-AS1	1,04
centromere protein W	CENPW	1,04
macrophage receptor with collagenous structure	MARCO	1,04
MGAT4 family, member D	MGAT4D	1,04
CCDC144NL antisense RNA 1	CCDC144NL-AS1	1,04
transmembrane protein 178B	TMEM178B	1,04
growth arrest-specific 2	GAS2	1,04
chimerin 2	CHN2	1,04
activation-induced cytidine deaminase	AICDA	1,04
BTB (POZ) domain containing 7	BTBD7	1,04
ubiquitin specific peptidase 10	USP10	1,04
paired related homeobox 1	PRRX1	1,04
oxoeicosanoid (OXE) receptor 1	OXER1	1,04
ferric-chelate reductase 1	FRRS1	1,04
cartilage acidic protein 1	CRTAC1	1,04
survival of motor neuron 1, telomeric; survival of motor neuron 2, centromeric	SMN1; SMN2	1,04
complement component 3	C3	1,04
mitochondrial transcription termination factor 2	MTERF2	1,04
killer cell lectin-like receptor subfamily C, member 2	KLRC2	1,04
coenzyme Q6 monooxygenase	COQ6	1,04
heterogeneous nuclear ribonucleoprotein A2/B1	HNRNPA2B1	1,03
crystallin beta A4	CRYBA4	1,03
taste receptor, type 2, member 60	TAS2R60	1,03
endoplasmic reticulum aminopeptidase 1	ERAP1	1,03
zinc finger protein, X-linked	ZFX	1,03
transglutaminase 4	TGM4	1,03
fragile X mental retardation 1 neighbor	FMR1NB	1,03
Eukaryotic translation initiation factor 3 subunit F [Source:UniProtKB/Swiss-Prot;Acc:O00303]	EIF3F	1,03
integrin, alpha M (complement component 3 receptor 3 subunit)	ITGAM	1,03
testis-specific serine kinase 6	TSSK6	1,03
IZUMO family member 3	IZUMO3	1,03
Homo sapiens polyhomeotic homolog 1 (Drosophila), mRNA (cDNA clone MGC:87926 IMAGE:5788132), complete cds.; Homo sapiens polyhomeotic homolog 1 (Drosophila), mRNA (cDNA clone MGC:189745 IMAGE:9057069), complete cds.	PHC1	1,03
dopamine receptor D1	DRD1	1,03
G protein-coupled receptor 61	GPR61	1,03
apoptosis antagonizing transcription factor	AATF	1,03
heterogeneous nuclear ribonucleoprotein C-like 4 [Source:HGNC Symbol;Acc:HGNC:51333]	HNRNPCL4	1,03

G protein-coupled receptor kinase interacting ArfGAP 2	GIT2	1,03
exocyst complex component 5	EXOC5	1,03
zona pellucida glycoprotein 4	ZP4	1,03
retinaldehyde binding protein 1	RLBP1	1,03
HERV-H LTR-associating 3	HHLA3	1,03
SREK1-interacting protein 1	SREK1IP1	1,03
Synthetic construct Homo sapiens clone IMAGE:100062553, MGC:190557 B melanoma antigen family, member 5 (BAGE5) mRNA, encodes complete protein.; B melanoma antigen family, member 5 [Source:EntrezGene;Acc:85316]	BAGE5	1,03
NAD(P)H dehydrogenase, quinone 1	NQO1	1,03
cilia and flagella associated protein 58	CFAP58	1,03
cutC copper transporter	CUTC	1,03
farnesyltransferase, CAAX box, beta	FNTB	1,03
dynein, cytoplasmic 1, light intermediate chain 1	DYNC1LI1	1,03
phospholipase A2, group IIF	PLA2G2F	1,03
PRP40 homolog, pre-mRNA processing factor B	PRPF40B	1,03
synaptotagmin V	SYT5	1,03
CD82 molecule	CD82	1,03
CTR9 homolog, Paf1/RNA polymerase II complex component	CTR9	1,03
ATPase, H+ transporting, lysosomal accessory protein 2	ATP6AP2	1,03
protein phosphatase, Mg2+/Mn2+ dependent, 1F	PPM1F	1,03
ribosomal protein L21	RPL21	1,03
ankyrin repeat and SOCS box containing 13	ASB13	1,03
angiotensin II receptor-associated protein	AGTRAP	1,03
UDP-GlcNAc:betaGal beta-1,3-N-acetylglucosaminyltransferase 7	B3GNT7	1,03
zinc finger protein 44	ZNF44	1,03
solute carrier family 46, member 3	SLC46A3	1,03
HLA complex group 27 (non-protein coding)	HCG27	1,03
protease, serine 41	PRSS41	1,03
bromodomain and PHD finger containing 1	BRPF1	1,03
centriole, cilia and spindle-associated protein	CCSAP	1,03
argininosuccinate lyase	ASL	1,03
cholinergic receptor, muscarinic 1	CHRM1	1,03
mitochondrial ribosomal protein L32	MRPL32	1,03
megakaryocyte-associated tyrosine kinase	MATK	1,03
testis expressed 26	TEX26	1,03
AT rich interactive domain 3A (BRIGHT-like)	ARID3A	1,03
basic transcription factor 3	BTF3	1,03
aryl-hydrocarbon receptor nuclear translocator 2; microRNA 5572	ARNT2; MIR5572	1,03
SHC (Src homology 2 domain containing) transforming protein 2	SHC2	1,03
Jeck2013 ANTISENSE, CDS, coding, INTERNAL, intronic, OVCODE, OVEXON best transcript NM_012242	DKK1	1,03

formin binding protein 4	FNBP4	1,03
ATPase, H+ transporting, lysosomal 34kDa, V1 subunit D	ATP6V1D	1,03
keratin associated protein 15-1	KRTAP15-1	1,03
uncharacterized LOC729220; novel transcript antisense to TAC4	FLJ45513; RP11-304F15.3	1,03
regulatory associated protein of MTOR, complex 1	RPTOR	1,03
coiled-coil domain containing 94	CCDC94	1,03
focadhesin	FOCAD	1,03
centrosomal protein 135kDa	CEP135	1,03
poly(ADP-ribose) polymerase family member 6	PARP6	1,03
SLIT and NTRK-like family, member 2	SLITRK2	1,03
nonhomologous end-joining factor 1	NHEJ1	1,03
GAS6 antisense RNA 1	GAS6-AS1	1,03
paired box 4	PAX4	1,03
sorbitol dehydrogenase	SORD	1,03
FANCD2/FANCI-associated nuclease 1	FAN1	1,03
alanyl (membrane) aminopeptidase	ANPEP	1,03
olfactory receptor, family 2, subfamily D, member 2	OR2D2	1,03
enoyl-CoA hydratase, short chain, 1, mitochondrial	ECHS1	1,03
DNA nucleotidylexotransferase	DNTT	1,03
collagen, type XXII, alpha 1	COL22A1	1,03
Transcript Identified by AceView, Entrez Gene ID(s) 287	ANK2	1,03
olfactory receptor, family 4, subfamily D, member 10	OR4D10	1,03
nucleoporin 155kDa	NUP155	1,03
catenin (cadherin-associated protein), delta 1	CTNND1	1,03
solute carrier family 9, subfamily B (NHA1, cation proton antiporter 1), member 1	SLC9B1	1,03
Homo sapiens ribosomal protein S26, mRNA (cDNA clone MGC:27148 IMAGE:4807004), complete cds.; Homo sapiens ribosomal protein S26, mRNA (cDNA clone MGC:88201 IMAGE:6388128), complete cds.; Homo sapiens ribosomal protein S26, mRNA (cDNA clone MGC:104291 IMAGE:4287636), complete cds.; Homo sapiens ribosomal protein S26, mRNA (cDNA clone MGC:104292 IMAGE:6726218), complete cds.	RPS26	1,03
sex comb on midleg homolog 1 (Drosophila)	SCMH1	1,03
CXXC finger protein 5	CXXC5	1,03
taste receptor, type 2, member 20	TAS2R20	1,03
motile sperm domain containing 3	MOSPD3	1,03
NudC domain containing 3	NUDCD3	1,03
synaptophysin-like 1	SYPL1	1,03
armadillo repeat containing 9	ARMC9	1,03
thyroid stimulating hormone receptor	TSHR	1,03
zinc finger protein 182	ZNF182	1,03
Salzman2013 ALT_ACCEPTOR, ALT_DONOR, coding, INTERNAL, intronic best transcript NM_007112	THBS3	1,03

melanoma inhibitory activity family, member 3	MIA3	1,03
B-cell linker	BLNK	1,03
pleckstrin homology domain containing, family G (with RhoGef domain) member 5	PLEKHG5	1,03
ribosomal protein L23a pseudogene 5	RPL23AP5	1,03
pentatricopeptide repeat domain 3	PTCD3	1,03
glutathione S-transferase kappa 1	GSTK1	1,03
nucleolar protein 11	NOL11	1,03
transmembrane protein 33	TMEM33	1,03
activating signal cointegrator 1 complex subunit 1	ASCC1	1,03
CD177 molecule	CD177	1,03
queuine tRNA-ribosyltransferase 1	QTRT1	1,03
regulating synaptic membrane exocytosis 2	RIMS2	1,03
FEZ family zinc finger 1	FEZF1	1,03
Cdk5 and Abl enzyme substrate 2	CABLES2	1,03
toll-like receptor 1	TLR1	1,03
olfactory receptor, family 5, subfamily P, member 2	OR5P2	1,03
NOP2/Sun RNA methyltransferase family, member 2	NSUN2	1,03
POU class 5 homeobox 1	POU5F1	1,03
transmembrane protein 99	TMEM99	1,03
tripartite motif family like 1	TRIML1	1,03
TRAF-type zinc finger domain containing 1	TRAFD1	1,03
ATPase, H+/K+ transporting, nongastric, alpha polypeptide	ATP12A	1,03
polymerase (RNA) II (DNA directed) polypeptide K, 7.0kDa	POLR2K	1,03
chemokine (C-C motif) ligand 23	CCL23	1,03
microRNA 4728; erb-b2 receptor tyrosine kinase 2	MIR4728; ERBB2	1,03
epsin 1	EPN1	1,03
uncharacterized LOC285692; novel transcript; Transcript Identified by AceView, Entrez Gene ID(s) 285692; 50834	LOC285692; CTD-2143L24.1; TAS2R1	1,03
solute carrier family 37, member 3	SLC37A3	1,03
proteasome subunit alpha 2	PSMA2	1,03
RNA binding motif protein 39	RBM39	1,03
heparan sulfate 6-O-sulfotransferase 3	HS6ST3	1,03
glycosyltransferase 8 domain containing 2	GLT8D2	1,03
apolipoprotein C-IV	APOC4	1,03
olfactory receptor, family 9, subfamily G, member 4	OR9G4	1,03
solute carrier family 2 (facilitated glucose transporter), member 6 [Source:HGNC Symbol;Acc:HGNC:11011]	SLC2A6	1,03
isoamyl acetate-hydrolyzing esterase 1 homolog	IAH1	1,03
chromosome 22 open reading frame 42	C22orf42	1,03
inositol-trisphosphate 3-kinase A	ITPKA	1,03
cytochrome P450, family 2, subfamily D, polypeptide 6	CYP2D6	1,03

zinc finger, CCHC domain containing 9	ZCCHC9	1,03
lysophosphatidic acid receptor 3	LPAR3	1,03
GINS complex subunit 3 (Psf3 homolog)	GINS3	1,03
zinc finger protein 749	ZNF749	1,03
aldehyde dehydrogenase 2 family (mitochondrial)	ALDH2	1,03
microtubule-associated protein, RP/EB family, member 2	MAPRE2	1,03
adenomatosis polyposis coli down-regulated 1	APCDD1	1,03
DDB1 and CUL4 associated factor 4	DCAF4	1,03
chromosome 16 open reading frame 52	C16orf52	1,03
DnaJ (Hsp40) homolog, subfamily A, member 1	DNAJA1	1,03
DDB1 and CUL4 associated factor 15	DCAF15	1,03
ATPase type 13A5	ATP13A5	1,03
claudin 3	CLDN3	1,03
prolactin	PRL	1,03
chaperonin containing TCP1, subunit 6A (zeta 1)	CCT6A	1,03
peptidylprolyl cis/trans isomerase, NIMA-interacting 4	PIN4	1,03
nuclear receptor binding factor 2	NRBF2	1,03
sphingomyelin phosphodiesterase 3, neutral membrane (neutral sphingomyelinase II)	SMPD3	1,03
ATPase, class II, type 9A	ATP9A	1,03
Transcript Identified by AceView, Entrez Gene ID(s) 153572	IRX2	1,03
porcupine homolog (Drosophila)	PORCN	1,03
ribosomal protein L7	RPL7	1,03
myosin light chain 9	MYL9	1,03
dynein, axonemal, heavy chain 10 opposite strand	DNAH10OS	1,03
ring finger protein 20, E3 ubiquitin protein ligase	RNF20	1,03
uncharacterized LOC285150; uncharacterized LOC285150 [Source:EntrezGene;Acc:285150]; novel transcript	FLJ33534; AC062028.1	1,03
solute carrier family 2 (facilitated glucose transporter), member 11	SLC2A11	1,03
zinc finger protein 768	ZNF768	1,03
calponin 3, acidic	CNN3	1,03
zinc finger protein 350	ZNF350	1,03
cathepsin C	CTSC	1,03
transmembrane protein 242	TMEM242	1,03
NADH dehydrogenase, subunit 6 (complex I)	ND6	1,03
ribosomal protein L27a; small nucleolar RNA, H/ACA box 3A; small nucleolar RNA, H/ACA box 3B	RPL27A; SNORA3A; SNORA3B	1,03
small nuclear ribonucleoprotein polypeptide B	SNRPB2	1,03
vestigial-like family member 3	VGLL3	1,03
alcohol dehydrogenase 1B (class I), beta polypeptide	ADH1B	1,03
engrailed homeobox 1	EN1	1,03
tripartite motif containing 34	TRIM34	1,03
ATPase family, AAA domain containing 2B	ATAD2B	1,03

ring finger protein 7	RNF7	1,03
CD244 molecule, natural killer cell receptor 2B4	CD244	1,03
carbohydrate (N-acetylgalactosamine 4-0) sulfotransferase 8	CHST8	1,03
regulatory factor X, 1 (influences HLA class II expression)	RFX1	1,03
fibroblast growth factor binding protein 2	FGFBP2	1,03
SWAP switching B-cell complex 70kDa subunit	SWAP70	1,03
O-linked N-acetylglucosamine (GlcNAc) transferase	OGT	1,03
phosphatidylinositol 4-kinase, catalytic, beta	PI4KB	1,03
toll-like receptor 10	TLR10	1,03
Bardet-Biedl syndrome 10	BBS10	1,03
ALG8, alpha-1,3-glucosyltransferase	ALG8	1,03
amphiregulin	AREG	1,03
homeobox A7	HOXA7	1,03
Zhang2013 ALT_ACCEPTOR, ALT_DONOR, coding, INTERNAL, intronic best transcript NM_001006641	SLC25A25	1,03
ankyrin repeat domain 28	ANKRD28	1,03
SRY box 21	SOX21	1,03
Rho GTPase activating protein 26	ARHGAP26	1,03
HAUS augmin like complex subunit 3; polymerase (DNA directed) nu	HAUS3; POLN	1,03
A kinase (PRKA) anchor protein 8	AKAP8	1,03
SON DNA binding protein; microRNA 6501	SON; MIR6501	1,03
Leber congenital amaurosis 5	LCA5	1,03
general transcription factor IIF subunit 2	GTF2F2	1,03
vacuolar protein sorting 28 homolog (S. cerevisiae)	VPS28	1,03
2,4-dienoyl-CoA reductase 1, mitochondrial	DECR1	1,03
glutamate receptor, metabotropic 7	GRM7	1,03
transmembrane protease, serine 11F	TMPRSS11F	1,03
acidic residue methyltransferase 1	ARMT1	1,03
C1q and tumor necrosis factor related protein 1	C1QTNF1	1,03
prospero homeobox 2	PROX2	1,03
chromosome 1 open reading frame 100	C1orf100	1,03
solute carrier family 2 (facilitated glucose transporter), member 6 [Source:HGNC Symbol;Acc:HGNC:11011]	SLC2A6	1,03
PRAME family member 9	PRAMEF9	1,03
chromosome 16 open reading frame 96	C16orf96	1,03
exostosin-like glycosyltransferase 3	EXTL3	1,03
apoptosis enhancing nuclease	AEN	1,03
leucine-rich repeats and calponin homology (CH) domain containing 1	LRCH1	1,03
solute carrier family 30 (zinc transporter), member 6	SLC30A6	1,03
interferon, lambda 4 (gene/pseudogene)	IFNL4	1,03
elongation factor, RNA polymerase II, 2	ELL2	1,03
glycogenin 2	GYG2	1,03
Werner syndrome, RecQ helicase-like	WRN	1,03

Transcript Identified by AceView, Entrez Gene ID(s) 10019	SH2B3	1,03
ubiquitin specific peptidase 17-like family member 10	USP17L10	1,03
cellular retinoic acid binding protein 2	CRABP2	1,03
zinc finger protein 394	ZNF394	1,03
vesicle transport through interaction with t-SNAREs 1B	VTI1B	1,03
ajuba LIM protein	AJUBA	1,03
claudin 22	CLDN22	1,03
solute carrier family 9, subfamily A (NHE3, cation proton antiporter 3), member 3	SLC9A3	1,03
lipin 1; microRNA 548s	LPIN1; MIR548S	1,03
prohibitin 2; small Cajal body-specific RNA 12	PHB2; SCARNA12	1,03
mitochondrial ribosomal protein S27	MRPS27	1,03
BRI3 binding protein	BRI3BP	1,03
HERV-H LTR-associating 1	HHLA1	1,03
forkhead box C2	FOXC2	1,03
tetraspanin 31	TSPAN31	1,03
ras homolog family member U	RHOU	1,03
double C2-like domains, beta	DOC2B	1,03
serine/threonine kinase 17a	STK17A	1,03
pro-platelet basic protein	PPBP	1,03
heparan sulfate (glucosamine) 3-O-sulfotransferase 6	HS3ST6	1,03
dolichol kinase	DOLK	1,03
chromosome 6 open reading frame 89	C6orf89	1,03
testis expressed 28	TEX28	1,03
Transcript Identified by AceView, Entrez Gene ID(s) 57448	BIRC6	1,03
BCL2-like 14 (apoptosis facilitator)	BCL2L14	1,03
ribosomal protein S19 binding protein 1	RPS19BP1	1,03
proline-rich protein HaeIII subfamily 1	PRH1	1,03
nuclear receptor subfamily 6, group A, member 1	NR6A1	1,03
heterogeneous nuclear ribonucleoprotein K; microRNA 7-1	HNRNPK; MIR7-1	1,03
coiled-coil domain containing 112	CCDC112	1,03
ferredoxin 1	FDX1	1,03
solute carrier family 44, member 4	SLC44A4	1,03
Memczak2013 ANTISENSE, CDS, coding, INTERNAL best transcript NM_015070	ZC3H13	1,03
coiled-coil domain containing 24	CCDC24	1,03
catalase	CAT	1,03
ST3 beta-galactoside alpha-2,3-sialyltransferase 1	ST3GAL1	1,03
Kruppel-like factor 8	KLF8	1,03
zinc finger protein 296	ZNF296	1,03
potassium channel, voltage gated subfamily E regulatory beta subunit 4	KCNE4	1,03

phosphodiesterase 6B, cGMP-specific, rod, beta	PDE6B	1,03
chromosome 5 open reading frame 66	C5orf66	1,03
calponin 2	CNN2	1,03
ectonucleoside triphosphate diphosphohydrolase 3	ENTPD3	1,03
CDC42 small effector 2	CDC42SE2	1,03
hypoxia inducible lipid droplet-associated	HILPDA	1,03
nucleoporin 88kDa	NUP88	1,03
cytochrome c oxidase subunit II	COX2	1,03
gliomedin	GLDN	1,03
related RAS viral (r-ras) oncogene homolog	RRAS	1,03
syntaxin 11	STX11	1,03
TTK protein kinase	TTK	1,03
paralemmin 2	PALM2	1,03
5-hydroxytryptamine (serotonin) receptor 1D, G protein-coupled	HTR1D	1,03
olfactory receptor, family 51, subfamily F, member 2	OR51F2	1,03
IQ motif containing with AAA domain 1 like	IQCA1L	1,03
calpain, small subunit 2	CAPNS2	1,03
coiled-coil domain containing 92	CCDC92	1,03
oxidative stress induced growth inhibitor family member 2	OSGIN2	1,03
neurogenin 2	NEUROG2	1,03
strawberry notch homolog 1 (Drosophila); microRNA 8072	SBNO1; MIR8072	1,03
cystinosin, lysosomal cystine transporter	CTNS	1,03
anti-Mullerian hormone; microRNA 4321	AMH; MIR4321	1,03
peptidyl arginine deiminase, type VI	PADI6	1,03
delta-like 3 (Drosophila)	DLL3	1,03
suppressor of cytokine signaling 7	SOCS7	1,03
protein phosphatase, Mg2+/Mn2+ dependent, 1M	PPM1M	1,03
zinc finger protein 330	ZNF330	1,03
F-box and leucine-rich repeat protein 4	FBXL4	1,03
GPN-loop GTPase 1	GPN1	1,03
zinc finger protein 224	ZNF224	1,03
nuclear receptor coactivator 5	NCOA5	1,03
mitochondrial ribosomal protein S14	MRPS14	1,03
presenilin associated, rhomboid-like	PARL	1,03
thymocyte nuclear protein 1	THYN1	1,03
NADH dehydrogenase (ubiquinone) 1 alpha subcomplex, 10, 42kDa	NDUFA10	1,03
abhydrolase domain containing 3	ABHD3	1,03
Williams-Beuren syndrome chromosome region 16	WBSCR16	1,03
dolichyl-phosphate mannosyltransferase polypeptide 1, catalytic subunit	DPM1	1,03
histone H4 transcription factor	HINFP	1,03
RNA binding motif protein 14	RBM14	1,03
BPI fold containing family A, member 1	BPIFA1	1,03

transmembrane protein 138	TMEM138	1,03
LON peptidase N-terminal domain and ring finger 3	LONRF3	1,03
C-type lectin domain family 4, member C	CLEC4C	1,03
zinc finger protein 319	ZNF319	1,03
coiled-coil domain containing 22	CCDC22	1,03
crystallin gamma N	CRYGN	1,03
opsin 1 (cone pigments), short-wave-sensitive	OPN1SW	1,03
keratin associated protein 19-3	KRTAP19-3	1,03
coiled-coil domain containing 93	CCDC93	1,03
TBC1 domain family, member 3	TBC1D3	1,03
RAB33A, member RAS oncogene family	RAB33A	1,03
coiled-coil domain containing 77	CCDC77	1,03
pyrophosphatase (inorganic) 2	PPA2	1,03
family with sequence similarity 193, member B	FAM193B	1,03
Transcript Identified by AceView, Entrez Gene ID(s) 80305	TRABD	1,03
dynein, axonemal, heavy chain 5	DNAH5	1,03
cytochrome c oxidase subunit VIb polypeptide 1 (ubiquitous)	COX6B1	1,03
ATPase, Na+/K+ transporting, alpha 3 polypeptide	ATP1A3	1,03
nuclear receptor subfamily 5, group A, member 2	NR5A2	1,03
zinc finger, BED-type containing 9	ZBED9	1,03
mitochondrial poly(A) polymerase	MTPAP	1,03
lysine (K)-specific demethylase 6B	KDM6B	1,03
ATPase, H+ transporting, lysosomal 34kDa, V1 subunit D	ATP6V1D	1,03
ubiquinol-cytochrome c reductase core protein II	UQCRC2	1,03
proteasome 26S subunit, non-ATPase 2	PSMD2	1,03
zinc finger protein 724, pseudogene	ZNF724P	1,03
motilin receptor	MLNR	1,03
catenin (cadherin-associated protein), alpha 2	CTNNA2	1,03
claudin 1	CLDN1	1,03
BCL2/adenovirus E1B 19kD interacting protein like	BNIPL	1,03
kinesin light chain 2	KLC2	1,03
SPC25, NDC80 kinetochore complex component	SPC25	1,03
Fanconi anemia core complex associated protein 100	FAAP100	1,03
histone deacetylase 4	HDAC4	1,03
ubiquitin conjugating enzyme E2D 3	UBE2D3	1,03
RAB40C, member RAS oncogene family	RAB40C	1,03
nuclear receptor coactivator 2	NCOA2	1,03
guanine nucleotide binding protein (G protein), beta polypeptide 1	GNB1	1,03
ghrelin/obestatin prepropeptide	GHRL	1,03
transmembrane protein 87A	TMEM87A	1,03
cytochrome b5 reductase 4	CYB5R4	1,03
nicotinamide riboside kinase 1	NMRK1	1,03
protein arginine methyltransferase 3	PRMT3	1,03
golgin A8 family, member M	GOLGA8M	1,03

guanine nucleotide binding protein (G protein), q polypeptide	GNAQ	1,03
hydroxymethylbilane synthase	HMBS	1,03
keratin associated protein 24-1	KRTAP24-1	1,03
chromosome 7 open reading frame 72	C7orf72	1,03
zinc finger and BTB domain containing 11	ZBTB11	1,03
Rho guanine nucleotide exchange factor 7	ARHGEF7	1,03
zinc finger, X-linked, duplicated B	ZXDB	1,03
cornichon family AMPA receptor auxiliary protein 3	CNIH3	1,03
ubiquitin conjugating enzyme E2G 1	UBE2G1	1,03
lamin A/C	LMNA	1,03
fibulin 1	FBLN1	1,03
calpain 7	CAPN7	1,03
mitochondrial ribosomal protein L40	MRPL40	1,03
adhesion molecule with Ig-like domain 1	AMIGO1	1,03
zinc finger and BTB domain containing 12	ZBTB12	1,03
nuclear RNA export factor 2B; nuclear RNA export factor 2	NXF2B; NXF2	1,03
complement component 9	C9	1,03
Transcript Identified by AceView, Entrez Gene ID(s) 114757; novel transcript, antisense to PRCD	CYGB; RP11-666A8.8	1,03
myosin binding protein C, slow type	MYBPC1	1,03
ADAM metallopeptidase domain 23	ADAM23	1,03
eukaryotic translation elongation factor 2; small nucleolar RNA, C/D box 37	EEF2; SNORD37	1,03
annexin A5	ANXA5	1,03
family with sequence similarity 229, member B	FAM229B	1,03
zinc finger protein 76	ZNF76	1,03
cornichon family AMPA receptor auxiliary protein 4	CNIH4	1,03
beaded filament structural protein 1, filensin	BFSP1	1,03
FIC domain containing	FICD	1,03
radical S-adenosyl methionine domain containing 2	RSAD2	1,03
protein kinase, cGMP-dependent, type I	PRKG1	1,03
ribosomal protein L9	RPL9	1,03
SPATA31 subfamily A, member 7; SPATA31 subfamily A, member 5	SPATA31A7; SPATA31A5	1,03
phosphodiesterase 5A, cGMP-specific	PDE5A	1,03
tetratricopeptide repeat domain 7A	TTC7A	1,03
lin-28 homolog B (C. elegans)	LIN28B	1,03
mitochondrial methionyl-tRNA formyltransferase	MTFMT	1,03
single stranded DNA binding protein 3	SSBP3	1,03
zinc finger protein 461	ZNF461	1,03
limbic system-associated membrane protein	LSAMP	1,03
dehydrodolichyl diphosphate synthase subunit	DHDDS	1,03
ST3 beta-galactoside alpha-2,3-sialyltransferase 5	ST3GAL5	1,03
USO1 vesicle transport factor	USO1	1,03

NADH dehydrogenase (ubiquinone) 1 alpha subcomplex, 10, 42kDa	NDUFA10	1,03
Kin17 DNA and RNA binding protein	KIN	1,03
LSM6 homolog, U6 small nuclear RNA and mRNA degradation associated	LSM6	1,03
surfeit 4	SURF4	1,03
surfeit 4	SURF4	1,03
BCL2-like 15	BCL2L15	1,03
Rho/Rac guanine nucleotide exchange factor 18	ARHGEF18	1,03
WD repeat domain 90	WDR90	1,03
interleukin 17 receptor A	IL17RA	1,03
sema domain, transmembrane domain (TM), and cytoplasmic domain, (semaphorin) 6C	SEMA6C	1,03
2-5-oligoadenylate synthetase 3	OAS3	1,03
FK506 binding protein 7	FKBP7	1,03
protein kinase C, theta	PRKCQ	1,03
zinc finger protein 254	ZNF254	1,03
growth hormone inducible transmembrane protein	GHITM	1,03
oxysterol binding protein-like 5	OSBPL5	1,03
zinc finger protein 169	ZNF169	1,03
K(lysine) acetyltransferase 6B	KAT6B	1,03
mitochondrial fission factor	MFF	1,03
phosphodiesterase 6H, cGMP-specific, cone, gamma	PDE6H	1,03
zinc finger protein 324B	ZNF324B	1,03
F-box and WD repeat domain containing 11	FBXW11	1,03
deoxyribonuclease II, lysosomal	DNASE2	1,03
tachykinin receptor 2	TACR2	1,03
cyclin D3	CCND3	1,03
long intergenic non-protein coding RNA 282	LINC00282	1,03
aldehyde dehydrogenase 5 family, member A1	ALDH5A1	1,03
Zhang2013 ALT_ACCEPTOR, ALT_DONOR, coding, INTERNAL, intronic best transcript NM_002882	RANBP1	1,03
CDK5 regulatory subunit associated protein 1-like 1	CDKAL1	1,03
pyruvate dehyrogenase phosphatase catalytic subunit 1	PDP1	1,03
mitochondrial ribosomal protein L21	MRPL21	1,03
potassium channel, two pore domain subfamily K, member 10	KCNK10	1,03
transmembrane protein 257	TMEM257	1,03
Thy-1 cell surface antigen	THY1	1,03
basigin (Ok blood group)	BSG	1,03
galanin/GMAP prepropeptide	GAL	1,03
VPS11, CORVET/HOPS core subunit [Source:HGNC Symbol;Acc:HGNC:14583]	VPS11	1,03
pleckstrin homology domain containing, family A (phosphoinositide binding specific) member 3	PLEKHA3	1,03
ring finger protein 207	RNF207	1,03

erythrocyte membrane protein band 4.1	EPB41	1,03
cyclin and CBS domain divalent metal cation transport mediator 2	CNNM2	1,03
suppressor of glucose, autophagy associated 1	SOGA1	1,03
regulator of G-protein signaling 7	RGS7	1,03
hydroxymethylbilane synthase	HMBS	1,03
leucine rich repeat containing 8 family, member E	LRRC8E	1,03
cilia and flagella associated protein 161	CFAP161	1,03
GPN-loop GTPase 2	GPN2	1,03
bromodomain containing 3	BRD3	1,03
acetyl-CoA acyltransferase 2	ACAA2	1,03
transmembrane protein 92	TMEM92	1,03
LDL receptor related protein 6	LRP6	1,03
chemokine (C-C motif) ligand 2	CCL2	1,03
lysine (K)-specific demethylase 4A	KDM4A	1,03
kinase suppressor of ras 1	KSR1	1,03
dynein, cytoplasmic 1, intermediate chain 2	DYNC1I2	1,03
tektin 1	TEKT1	1,03
sema domain, immunoglobulin domain (Ig), short basic domain, secreted, (semaphorin) 3A	SEMA3A	1,03
Vac14 homolog (S. cerevisiae)	VAC14	1,03
POU class 2 associating factor 1	POU2AF1	1,03
transmembrane p24 trafficking protein family member 8	TMED8	1,03
glutamate receptor, ionotropic, delta 1	GRID1	1,03
ribosomal protein L18a	RPL18A	1,03
solute carrier family 12, member 8	SLC12A8	1,03
fibroblast growth factor receptor 4	FGFR4	1,03
runt-related transcription factor 1	RUNX1	1,03
Fanconi anemia core complex associated protein 24	FAAP24	1,03
guanylate cyclase activator 2B (uroguanylin)	GUCA2B	1,03
leucine rich repeat containing 37, member A11, pseudogene	LRRC37A11P	1,03
stabilizer of axonemal microtubules 1	SAXO1	1,03
chromosome 3 open reading frame 17	C3orf17	1,03
SPRY domain containing 3	SPRYD3	1,03
transmembrane protein 216	TMEM216	1,03
odorant binding protein 2B	OBP2B	1,03
enoyl-CoA hydratase 1, peroxisomal	ECH1	1,03
protein phosphatase 3, regulatory subunit B, beta	PPP3R2	1,03
zinc finger protein 8	ZNF8	1,03
family with sequence similarity 105, member A	FAM105A	1,03
neuromedin U receptor 1	NMUR1	1,03
cell cycle exit and neuronal differentiation 1	CEND1	1,03
transmembrane protein 246	TMEM246	1,03
family with sequence similarity 24, member A	FAM24A	1,03
homeodomain interacting protein kinase 2	HIPK2	1,03

XPA binding protein 2	XAB2	1,03
ADP-ribosylation factor 5	ARF5	1,03
methionine adenosyltransferase I, alpha	MAT1A	1,03
MAS-related GPR, member X3	MRGPRX3	1,03
Transcript Identified by AceView, Entrez Gene ID(s) 10926	DBF4	1,03
5-hydroxytryptamine (serotonin) receptor 2A, G protein-coupled	HTR2A	1,03
zinc finger protein 546	ZNF546	1,03
calpain 1, (mu/I) large subunit	CAPN1	1,03
major facilitator superfamily domain containing 12	MFSD12	1,03
peroxiredoxin 5	PRDX5	1,03
ubinuclein 1	UBN1	1,03
carnitine palmitoyltransferase 1B (muscle)	CPT1B	1,03
ubiquitin conjugating enzyme E2L 3	UBE2L3	1,03
golgin A6 family-like 22; golgin A6 family-like 1	GOLGA6L22; GOLGA6L1	1,03
inositol polyphosphate-4-phosphatase type II B	INPP4B	1,03
purinergic receptor P2X, ligand gated ion channel, 7	P2RX7	1,03
ubiquitin-conjugating enzyme E2C	UBE2C	1,03
glycerophosphodiester phosphodiesterase 1	GDE1	1,03
Hermansky-Pudlak syndrome 5	HPS5	1,03
high mobility group AT-hook 2	HMGA2	1,03
ribosomal protein S4, X-linked	RPS4X	1,03
hes family bHLH transcription factor 3	HES3	1,03
solute carrier family 7 (amino acid transporter light chain, L system), member 8	SLC7A8	1,03
carboxypeptidase D	CPD	1,03
retinol saturase (all-trans-retinol 13,14-reductase)	RETSAT	1,03
DEAH (Asp-Glu-Ala-His) box polypeptide 16	DHX16	1,03
block of proliferation 1; microRNA 7112	BOP1; MIR7112	1,03
Rab9 effector protein with kelch motifs	RABEPK	1,03
chromosome 10 open reading frame 2	C10orf2	1,03
macrophage stimulating 1-like	MST1L	1,03
protease, serine, 57	PRSS57	1,03
LIM domain kinase 1	LIMK1	1,03
cholinergic receptor, nicotinic alpha 10; nucleoporin 98kDa	CHRNA10; NUP98	1,03
heat shock 60kDa protein 1 (chaperonin)	HSPD1	1,03
ralA binding protein 1	RALBP1	1,03
pogo transposable element with KRAB domain	POGK	1,03
chromosome 2 open reading frame 47	C2orf47	1,03
phosphatase, orphan 2	PHOSPHO2	1,03
NAD(P) dependent steroid dehydrogenase-like	NSDHL	1,03
ArfGAP with RhoGAP domain, ankyrin repeat and PH domain 2	ARAP2	1,03
cache domain containing 1	CACHD1	1,03

cleavage stimulation factor, 3 pre-RNA, subunit 1	CSTF1	1,03
synaptosome associated protein 23kDa	SNAP23	1,03
suppressor of cytokine signaling 1	SOCS1	1,03
pyridine nucleotide-disulphide oxidoreductase domain 1	PYROXD1	1,03
UBX domain protein 2A	UBXN2A	1,03
ankyrin repeat domain 12	ANKRD12	1,03
transmembrane protein 191B	TMEM191B	1,03
proteasome 26S subunit, ATPase 1	PSMC1	1,03
sterile alpha motif domain containing 13	SAMD13	1,03
G protein-coupled receptor 84	GPR84	1,03
sterol O-acyltransferase 2	SOAT2	1,03
late cornified envelope 1B	LCE1B	1,03
proteoglycan 2, bone marrow (natural killer cell activator, eosinophil granule major basic protein)	PRG2	1,03
solute carrier family 22, member 24	SLC22A24	1,03
sodium channel, non voltage gated 1 beta subunit	SCNN1B	1,03
zinc finger protein 281	ZNF281	1,03
solute carrier family 30 (zinc transporter), member 4	SLC30A4	1,03
Memczak2013 ANTISENSE, coding, INTERNAL, intronic best transcript NM_153234	LIX1	1,03
SMAD family member 6	SMAD6	1,03
ezrin	EZR	1,03
heat shock transcription factor 4; F-box and leucine-rich repeat protein 8	HSF4; FBXL8	1,03
muskelin 1, intracellular mediator containing kelch motifs	MKLN1	1,03
NCK adaptor protein 1	NCK1	1,03
growth factor receptor bound protein 2	GRB2	1,03
argonaute RISC catalytic component 1	ago-01	1,03
NYN domain and retroviral integrase containing	NYNRIN	1,03
olfactory receptor, family 10, subfamily A, member 4	OR10A4	1,03
RAB21, member RAS oncogene family	RAB21	1,03
dihydropyrimidinase	DPYS	1,03
transmembrane protein 221	TMEM221	1,03
pleckstrin homology domain containing, family A (phosphoinositide binding specific) member 4	PLEKHA4	1,03
golgin A8 family, member J	GOLGA8J	1,03
fermitin family member 1	FERMT1	1,03
indian hedgehog	IHH	1,03
FGGY carbohydrate kinase domain containing	FGGY	1,03
coiled-coil domain containing 61	CCDC61	1,03
splicing regulatory glutamine/lysine-rich protein 1	SREK1	1,03
ribosomal protein L41	RPL41	1,03
dishevelled segment polarity protein 1; microRNA 6808	DVL1; MIR6808	1,03
chromosome 12 open reading frame 57; RNA, U7 small nuclear 1	C12orf57; RNU7-1	1,03

potassium channel, voltage gated Shal related subfamily D, member 2	KCND2	1,03
periaxin	PRX	1,03
diacylglycerol kinase, delta 130kDa	DGKD	1,03
ring finger protein 144B	RNF144B	1,03
Fas (TNFRSF6) binding factor 1	FBF1	1,03
Rhox homeobox family, member 2B; Rhox homeobox family, member 2	RHOXF2B; RHOXF2	1,03
tubulin polyglutamylase complex subunit 2	TPGS2	1,03
chromosome 18 open reading frame 65	C18orf65	1,03
zinc finger protein 622	ZNF622	1,03
coiled-coil domain containing 96	CCDC96	1,03
NODAL modulator 3 [Source:HGNC Symbol;Acc:HGNC:25242]; novel transcript, antisense to NOMO3; RP11-517A5.4 (from geneSymbol)	RP11-517A5.5; NOMO3; RP11-517A5.4	1,03
uncharacterized LOC101927668; Transcript Identified by AceView, Entrez Gene ID(s) 346389; novel transcript, sense overlapping to MACC1	LOC101927668; AC005062.2; MACC1	1,03
SET nuclear proto-oncogene	SET	1,03
coagulation factor VIII-associated 2	F8A2	1,03
centriolin	CNTRL	1,03
cytochrome c oxidase subunit VIa polypeptide 1	COX6A1	1,03
polymerase (RNA) III (DNA directed) polypeptide C (62kD)	POLR3C	1,03
leucine rich repeat containing 75A	LRRC75A	1,03
Transcript Identified by AceView, Entrez Gene ID(s) 4928	NUP98	1,03
methionine sulfoxide reductase A	MSRA	1,03
gasdermin A	GSDMA	1,03
interleukin 1 alpha	IL1A	1,03
mitochondrial ribosomal protein L14	MRPL14	1,03
CCR4-NOT transcription complex subunit 11	CNOT11	1,03
MyoD family inhibitor domain containing	MDFIC	1,03
carboxypeptidase N, polypeptide 2	CPN2	1,03
indolethylamine N-methyltransferase	INMT	1,03
sorting nexin family member 30	SNX30	1,03
olfactory receptor, family 52, subfamily A, member 5	OR52A5	1,03
neurogranin (protein kinase C substrate, RC3)	NRGN	1,03
NLR family, CARD domain containing 4	NLRC4	1,03
ADP-ribosylation factor GTPase activating protein 2	ARFGAP2	1,03
tubulin, alpha-like 3	TUBAL3	1,03
multiple EGF-like-domains 9	MEGF9	1,03
phosphoglucomutase 2-like 1	PGM2L1	1,03
mutated in colorectal cancers	MCC	1,03
kinase D-interacting substrate 220kDa	KIDINS220	1,03
zinc finger protein 208	ZNF208	1,03
ankyrin repeat domain 33B	ANKRD33B	1,03

telomeric repeat binding factor 2	TERF2	1,03
centrosomal protein 57kDa-like 1	CEP57L1	1,03
ring finger protein 212B	RNF212B	1,03
mediator of cell motility 1	MEMO1	1,03
protein kinase, interferon-inducible double stranded RNA dependent activator	PRKRA	1,03
glutamic pyruvate transaminase (alanine aminotransferase) 2	GPT2	1,03
3-hydroxyacyl-CoA dehydratase 4	HACD4	1,03
myosin light chain 12B	MYL12B	1,03
PDZK1 interacting protein 1	PDZK1IP1	1,03
zinc finger and BTB domain containing 14	ZBTB14	1,03
Homo sapiens TP53 target 3D (TP53TG3D), transcript variant 2, non-coding RNA.; TP53-target gene 3 protein; Homo sapiens TP53 target 3, mRNA (cDNA clone MGC:119889 IMAGE:40015196), complete cds.; Homo sapiens TP53 target 3, mRNA (cDNA clone MGC:119888 IMAGE:40015195), complete cds.	TP53TG3D; LOC102723655; TP53TG3; AC136612.1	1,03
zinc finger protein 79	ZNF79	1,03
peptidylprolyl isomerase C (cyclophilin C)	PPIC	1,03
colony stimulating factor 3	CSF3	1,03
eukaryotic translation initiation factor 3, subunit D	EIF3D	1,03
outer dense fiber of sperm tails 3B	ODF3B	1,03
chorionic gonadotropin, beta polypeptide 2	CGB2	1,03
heterogeneous nuclear ribonucleoprotein R	HNRNPR	1,03
myosin light chain 5	MYL5	1,03
trophoblast glycoprotein-like	TPBGL	1,03
RIO kinase 1	RIOK1	1,03
acylphosphatase 2, muscle type	ACYP2	1,03
PTC7 protein phosphatase homolog	PPTC7	1,03
long intergenic non-protein coding RNA 1296; double homeobox A pseudogene 10	LINC01296; DUXAP10	1,03
proprotein convertase subtilisin/kexin type 1	PCSK1	1,03
chromosome 14 open reading frame 1	C14orf1	1,03
syntaxin 7	STX7	1,03
poly(A) binding protein, nuclear 1-like (cytoplasmic)	PABPN1L	1,03
protein phosphatase, Mg2+/Mn2+ dependent, 1L	PPM1L	1,03
ADAM metallopeptidase with thrombospondin type 1 motif 13	ADAMTS13	1,03
MTND2	ND2	1,03
nudix hydrolase 3	NUDT3	1,03
insulin like growth factor binding protein-like 1	IGFBPL1	1,03
prothymosin, alpha	PTMA	1,03
solute carrier family 43 (amino acid system L transporter), member 1	SLC43A1	1,03
pre-B-cell leukemia homeobox 2	PBX2	1,03
death associated protein 3	DAP3	1,03

family with sequence similarity 131, member C	FAM131C	1,03
ubiquitin-like modifier activating enzyme 2	UBA2	1,03
leucine rich repeat containing 10B	LRRC10B	1,03
translocator protein 2	TSPO2	1,03
dehydrogenase/reductase (SDR family) member 4	DHRS4	1,03
F-box protein 7	FBXO7	1,03
cystatin E/M	CST6	1,03
BLOC-1 related complex subunit 6	BORCS6	1,03
LIM homeobox 4	LHX4	1,03
pentatricopeptide repeat domain 1	PTCD1	1,03
reticulon 1	RTN1	1,03
chromosome 1 open reading frame 61	C1orf61	1,03
prolyl 3-hydroxylase 2	P3H2	1,03
l(3)mbt-like 3 (Drosophila)	L3MBTL3	1,03
HtrA serine peptidase 2	HTRA2	1,03
hydroxyacyl-CoA dehydrogenase	HADH	1,03
kinesin family member 3B	KIF3B	1,03
X-ray repair complementing defective repair in Chinese hamster cells 6	XRCC6	1,03
mutS homolog 2	MSH2	1,03
complement factor properdin	CFP	1,03
family with sequence similarity 83, member D	FAM83D	1,03
ADAM metallopeptidase with thrombospondin type 1 motif 9	ADAMTS9	1,03
keratin associated protein 5-9	KRTAP5-9	1,03
RNA binding protein, fox-1 homolog (C. elegans) 3	RBFOX3	1,03
sushi, nidogen and EGF-like domains 1	SNED1	1,03
ribonuclease P/MRP 38kDa subunit	RPP38	1,03
ELMO/CED-12 domain containing 1	ELMOD1	1,03
protein phosphatase 6, regulatory subunit 3	PPP6R3	1,03
triggering receptor expressed on myeloid cells-like 4	TREML4	1,03
retinol binding protein 4, plasma	RBP4	1,03
signal peptidase complex subunit 3	SPCS3	1,03
ring finger protein 187	RNF187	1,03
melanoma associated antigen (mutated) 1	MUM1	1,03
tyrosine 3-monooxygenase/tryptophan 5-monooxygenase activation protein, epsilon	YWHAE	1,03
serine peptidase inhibitor, Kunitz type, 2	SPINT2	1,03
androglobin	ADGB	1,03
desmoglein 2	DSG2	1,03
lysozyme G-like 2	LYG2	1,03
diphthamide biosynthesis 7	DPH7	1,03
serine peptidase inhibitor, Kazal type 4	SPINK4	1,03
filaggrin family member 2	FLG2	1,03
FERM domain containing 8	FRMD8	1,03
iron-sulfur cluster assembly 1	ISCA1	1,03

low density lipoprotein receptor adaptor protein 1	LDLRAP1	1,03
solute carrier family 25 (aspartate/glutamate carrier), member 12	SLC25A12	1,03
F-box and leucine-rich repeat protein 20	FBXL20	1,03
cholinergic receptor, nicotinic alpha 1	CHRNA1	1,03
methylmalonic aciduria (cobalamin deficiency) cblB type	MMAB	1,03
cerebral cavernous malformation 2	CCM2	1,03
peroxidasin	PXDN	1,03
paired box 2	PAX2	1,03
neuroplastin	NPTN	1,03
importin 9	IPO9	1,03
ash2 (absent, small, or homeotic)-like (Drosophila)	ASH2L	1,03
NADH dehydrogenase (ubiquinone) 1 alpha subcomplex, 6, 14kDa	NDUFA6	1,03
WD repeat domain 87	WDR87	1,03
solute carrier family 9, subfamily A (NHE7, cation proton antiporter 7), member 7	SLC9A7	1,03
signal peptidase complex subunit 2	SPCS2	1,03
dihydrofolate reductase like 1	DHFRL1	1,03
mitogen-activated protein kinase kinase kinase 11	MAP3K11	1,03
microsomal glutathione S-transferase 2	MGST2	1,03
SET binding factor 2	SBF2	1,03
zinc finger and BTB domain containing 40	ZBTB40	1,03
ELMO/CED-12 domain containing 2	ELMOD2	1,03
coagulation factor IX	F9	1,03
potassium channel, voltage gated eag related subfamily H, member 4	KCNH4	1,03
kinesin family member 15	KIF15	1,03
chromosome 21 open reading frame 91; C21orf91 overlapping transcript 1	C21orf91; C21orf91-OT1	1,03
tetratricopeptide repeat domain 9C	TTC9C	1,03
chromosome 11 open reading frame 86	C11orf86	1,03
ornithine decarboxylase antizyme 1	OAZ1	1,03
uncharacterized LOC101927550; Salzman2013 ANTISENSE, CDS, coding, INTERNAL, intronic, OVCODE, OVERLAPTX, OVEXON best transcript NM_020429; novel transcript	LOC101927550; AC004893.11; SMURF1	1,03
placental growth factor	PGF	1,03
mesoderm development candidate 2	MESDC2	1,03
shadow of prion protein homolog (zebrafish)	SPRN	1,03
chromosome 4 open reading frame 32	C4orf32	1,03
chromosome 1 open reading frame 228	C1orf228	1,03
regulation of nuclear pre-mRNA domain containing 1B	RPRD1B	1,03
L-2-hydroxyglutarate dehydrogenase	L2HGDH	1,03
proline synthetase co-transcribed homolog (bacterial)	PROSC	1,03
ADP-ribosylhydrolase like 1	ADPRHL1	1,03

ADP-ribosylation factor like GTPase 14 effector protein like	ARL14EPL	1,03
zeta chain of T cell receptor associated protein kinase 70kDa	ZAP70	1,03
DNA cross-link repair 1B	DCLRE1B	1,03
DCN1, defective in cullin neddylation 1, domain containing 4	DCUN1D4	1,03
heparan sulfate proteoglycan 2	HSPG2	1,03
dishevelled-binding antagonist of beta-catenin 3	DACT3	1,03
glycerate kinase	GLYCTK	1,03
RAD9 checkpoint clamp component A	RAD9A	1,03
mitochondrial translational release factor 1-like	MTRF1L	1,03
galactose mutarotase (aldose 1-epimerase)	GALM	1,03
EPM2A (laforin) interacting protein 1	EPM2AIP1	1,03
ribosomal protein S25	RPS25	1,03
lipocalin 8	LCN8	1,03
Zhang2013 ALT_ACCEPTOR, ALT_DONOR, coding, INTERNAL, intronic best transcript NM_003908	EIF2S2	1,03
sialic acid binding Ig-like lectin 10	SIGLEC10	1,03
heat shock protein 90kDa alpha (cytosolic), class A member 1	HSP90AA1	1,03
pyrroline-5-carboxylate reductase family, member 2; microRNA 6741	PYCR2; MIR6741	1,03
6-phosphofructo-2-kinase/fructose-2,6-biphosphatase 2	PFKFB2	1,03
thymocyte expressed, positive selection associated 1	TESPA1	1,03
zinc finger protein 638	ZNF638	1,03
adducin 1 (alpha)	ADD1	1,03
ring finger protein 215	RNF215	1,03
PR domain containing 14	PRDM14	1,03
adducin 2 (beta)	ADD2	1,03
toll-like receptor adaptor molecule 1	TICAM1	1,03
polymerase (DNA directed), gamma 2, accessory subunit	POLG2	1,03
KAT8 regulatory NSL complex subunit 3	KANSL3	1,03
DDB1 and CUL4 associated factor 6	DCAF6	1,03
KIAA1324	KIAA1324	1,03
thyroid hormone receptor interactor 12	TRIP12	1,03
G protein-coupled receptor 108; microRNA 6791	GPR108; MIR6791	1,03
Rho-related BTB domain containing 3	RHOBTB3	1,03
surfeit 4	SURF4	1,03
olfactory receptor, family 11, subfamily H, member 2	OR11H2	1,03
N-acetylglucosamine-1-phosphate transferase, gamma subunit	GNPTG	1,03

Homo sapiens proteasome (prosome, macropain) 26S subunit, ATPase, 1, mRNA (cDNA clone MGC:8541 IMAGE:2822718), complete cds.; Homo sapiens proteasome (prosome, macropain) 26S subunit, ATPase, 1, mRNA (cDNA clone MGC:24583 IMAGE:4133348), complete cds.; Homo sapiens proteasome (prosome, macropain) 26S subunit, ATPase, 1, mRNA (cDNA clone MGC:86994 IMAGE:5264945), complete cds.; Homo sapiens proteasome (prosome, macropain) 26S subunit, ATPase, 1, mRNA (cDNA clone MGC:88853 IMAGE:5456334), complete cds.	PSMC1	1,03
mitochondrial fission regulator 1	MTFR1	1,03
opsin 1 (cone pigments), medium-wave-sensitive; opsin 1 (cone pigments), medium-wave-sensitive 2; opsin 1 (cone pigments), medium-wave-sensitive 3	OPN1MW; OPN1MW2; OPN1MW3	1,03
F11 receptor	F11R	1,03
transmembrane protein 87B	TMEM87B	1,03
tyrosinase	TYR	1,03
fermitin family member 3	FERMT3	1,03
sialophorin	SPN	1,03
DEAD (Asp-Glu-Ala-Asp) box polypeptide 27	DDX27	1,03
dynein, light chain, Tctex-type 1	DYNLT1	1,03
phosphatidylinositol glycan anchor biosynthesis class P	PIGP	1,03
protein kinase C, delta	PRKCD	1,03
galactose-3-O-sulfotransferase 2	GAL3ST2	1,03
dual specificity phosphatase 16	DUSP16	1,03
ADAM metallopeptidase with thrombospondin type 1 motif 10	ADAMTS10	1,03
LDL receptor related protein 6	LRP6	1,03
MyoD family inhibitor	MDFI	1,03
poly(A) binding protein, cytoplasmic 4-like	PABPC4L	1,03
olfactory receptor, family 8, subfamily I, member 2	OR8I2	1,03
keratin associated protein 3-3	KRTAP3-3	1,03
Memczak2013 ANTISENSE, coding, INTERNAL, intronic best transcript NM_024830	LPCAT1	1,03
proteasome subunit beta 5	PSMB5	1,03
solute carrier family 25, member 34	SLC25A34	1,03
coiled-coil glutamate rich protein 2	CCER2	1,03
chromosome 9 open reading frame 9	C9orf9	1,03
cytochrome P450, family 2, subfamily F, polypeptide 1	CYP2F1	1,03
retinoblastoma binding protein 7	RBBP7	1,02
5-nucleotidase domain containing 1	NT5DC1	1,02
WAS protein homolog associated with actin, golgi membranes and microtubules	WHAMM	1,02
paired box 1	PAX1	1,02
TM2 domain containing 1	TM2D1	1,02
cyclin-dependent kinase inhibitor 3	CDKN3	1,02

hydroxy-delta-5-steroid dehydrogenase, 3 beta- and steroid delta-isomerase 1	HSD3B1	1,02
leucine zipper protein 1	LUZP1	1,02
mannosyl (alpha-1,6-)-glycoprotein beta-1,6-N-acetyl-glucosaminyltransferase, isozyme B	MGAT5B	1,02
interferon (alpha, beta and omega) receptor 1	IFNAR1	1,02
forkhead box K1	FOXK1	1,02
rhomboid domain containing 2	RHBDD2	1,02
protocadherin beta 13	PCDHB13	1,02
polymerase (DNA directed), delta 1, catalytic subunit	POLD1	1,02
metallophosphoesterase domain containing 1	MPPED1	1,02
Transcript Identified by AceView, Entrez Gene ID(s) 146167	SLC38A8	1,02
RWD domain containing 4	RWDD4	1,02
calnexin	CANX	1,02
Jeck2013 ANTISENSE, coding, INTERNAL, intronic best transcript NM_014191	SCN8A	1,02
karyopherin alpha 2 (RAG cohort 1, importin alpha 1)	KPNA2	1,02
zinc finger protein 362	ZNF362	1,02
ADAM metallopeptidase domain 8	ADAM8	1,02
arachidonate 12-lipoxygenase, 12R type	ALOX12B	1,02
nicotinamide nucleotide adenylyltransferase 1	NMNAT1	1,02
NPC1-like 1	NPC1L1	1,02
proline rich 11	PRR11	1,02
protein phosphatase 2, catalytic subunit, beta isozyme	PPP2CB	1,02
nitric oxide synthase 2, inducible	NOS2	1,02
catechol-O-methyltransferase domain containing 1	COMTD1	1,02
poly(ADP-ribose) polymerase 2	PARP2	1,02
zinc finger, C3H1-type containing	ZFC3H1	1,02
poly(A) binding protein interacting protein 2	PAIP2	1,02
fibroblast growth factor receptor 2	FGFR2	1,02
voltage-dependent anion channel 1	VDAC1	1,02
keratin associated protein 25-1	KRTAP25-1	1,02
annexin A10	ANXA10	1,02
pecanex-like 2 (Drosophila)	PCNXL2	1,02
microtubule-associated protein, RP/EB family, member 1	MAPRE1	1,02
synuclein beta	SNCB	1,02
solute carrier family 4 (sodium bicarbonate cotransporter), member 5	SLC4A5	1,02

Homo sapiens A kinase (PRKA) anchor protein 17A (AKAP17A), transcript variant 1, mRNA.; Homo sapiens A kinase (PRKA) anchor protein 17A (AKAP17A), transcript variant 2, non-coding RNA.; A kinase (PRKA) anchor protein 17A [Source:HGNC Symbol;Acc:HGNC:18783]; Homo sapiens splicing factor, arginine/serine-rich 17A, mRNA (cDNA clone MGC:39904 IMAGE:5217247), complete cds.; Homo sapiens splicing factor, arginine/serine-rich 17A, mRNA (cDNA clone MGC:125365 IMAGE:40007662), complete cds.; Homo sapiens splicing factor, arginine/serine-rich 17A, mRNA (cDNA clone MGC:125366 IMAGE:40007663), complete cds.; Jeck2013 ANNOTATED, INTERNAL, ncRNA, OVCODE, OVEXON best transcript NR_027383	AKAP17A; SFRS17A	1,02
isocitrate dehydrogenase 3 (NAD+) alpha	IDH3A	1,02
antizyme inhibitor 1	AZIN1	1,02
regulation of nuclear pre-mRNA domain containing 2	RPRD2	1,02
inositol hexakisphosphate kinase 1	IP6K1	1,02
F-box and leucine-rich repeat protein 18	FBXL18	1,02
solute carrier family 13 (sodium-dependent dicarboxylate transporter), member 2	SLC13A2	1,02
family with sequence similarity 170, member A	FAM170A	1,02
stromal interaction molecule 2	STIM2	1,02
brain-specific homeobox	BSX	1,02
ring finger protein 40, E3 ubiquitin protein ligase	RNF40	1,02
tudor domain containing 3	TDRD3	1,02
ribonucleotide reductase M2 B (TP53 inducible)	RRM2B	1,02
muscle, skeletal, receptor tyrosine kinase	MUSK	1,02
chaperonin containing TCP1, subunit 7 (eta)	CCT7	1,02
C-terminal binding protein 2	CTBP2	1,02
negative elongation factor complex member C/D	NELFCD	1,02
family with sequence similarity 155, member B	FAM155B	1,02
cyclin-dependent kinase 2 associated protein 1	CDK2AP1	1,02
membrane associated ring finger 7	MARCH7	1,02
transmembrane protein 53	TMEM53	1,02
glutamate rich 4	ERICH4	1,02
ring finger protein 157	RNF157	1,02
maltase-glucoamylase	MGAM	1,02
PAX interacting (with transcription-activation domain) protein 1	PAXIP1	1,02
required for meiotic nuclear division 5 homolog A	RMND5A	1,02
Jeck2013 ALT_DONOR, coding, INTERNAL, intronic best transcript NM_006845	KIF2C	1,02
AT rich interactive domain 2 (ARID, RFX-like)	ARID2	1,02
Salzman2013 ALT_ACCEPTOR, ALT_DONOR, coding, INTERNAL, intronic best transcript NM_152513	MEI1	1,02
sialidase 2 (cytosolic sialidase)	NEU2	1,02

coagulation factor X	F10	1,02
nyctalopin	NYX	1,02
phospholipase A2, group IVE	PLA2G4E	1,02
immunoglobulin superfamily, DCC subclass, member 4	IGDCC4	1,02
leucine rich repeat containing 52	LRRC52	1,02
annexin A1	ANXA1	1,02
gamma-aminobutyric acid (GABA) A receptor, pi	GABRP	1,02
golgi integral membrane protein 4	GOLIM4	1,02
prostate and testis expressed 3	PATE3	1,02
N-sulfoglucosamine sulfohydrolase	SGSH	1,02
phosphatidylinositol transfer protein, cytoplasmic 1	PITPNC1	1,02
N-ethylmaleimide-sensitive factor attachment protein, alpha	NAPA	1,02
NADH dehydrogenase (ubiquinone) 1 beta subcomplex, 3, 12kDa	NDUFB3	1,02
H2A histone family, member V	H2AFV	1,02
WAP four-disulfide core domain 8	WFDC8	1,02
crystallin mu	CRYM	1,02
insulin-like growth factor 1 receptor	IGF1R	1,02
autophagy related 16-like 1	ATG16L1	1,02
ribosomal protein L28; microRNA 6805	RPL28; MIR6805	1,02
solute carrier family 6 (neutral amino acid transporter), member 19	SLC6A19	1,02
glutamate-ammonia ligase	GLUL	1,02
ELOVL fatty acid elongase 3	ELOVL3	1,02
SH3 domain containing ring finger 3	SH3RF3	1,02
MAGE family member A6	MAGEA6	1,02
tubulin tyrosine ligase-like family member 6	TTLL6	1,02
glyceraldehyde-3-phosphate dehydrogenase, spermatogenic	GAPDHS	1,02
chromosome 16 open reading frame 82	C16orf82	1,02
NODAL modulator 2	NOMO2	1,02
hepatocyte nuclear factor 4, alpha	HNF4A	1,02
formimidoyltransferase cyclodeaminase	FTCD	1,02
death-associated protein kinase 1	DAPK1	1,02
armadillo repeat containing 8	ARMC8	1,02
LMBR1 domain containing 2	LMBRD2	1,02
hemoglobin, epsilon 1	HBE1	1,02
TM2 domain containing 3	TM2D3	1,02
Salzman2013 ANTISENSE, coding, INTERNAL, intronic best transcript NM_001153	ANXA4	1,02
ubiquitin-like modifier activating enzyme 7; microRNA 5193	UBA7; MIR5193	1,02
fucosyltransferase 10 (alpha (1,3) fucosyltransferase)	FUT10	1,02
Ts translation elongation factor, mitochondrial	TSFM	1,02
synaptopodin 2	SYNPO2	1,02
WD repeat domain 41	WDR41	1,02

small integral membrane protein 17	SMIM17	1,02
forkhead box P1	FOXP1	1,02
QKI, KH domain containing, RNA binding	QKI	1,02
dual serine/threonine and tyrosine protein kinase	DSTYK	1,02
olfactory receptor, family 1, subfamily M, member 1	OR1M1	1,02
dual specificity tyrosine-(Y)-phosphorylation regulated kinase 1A	DYRK1A	1,02
dipeptidyl-peptidase 7	DPP7	1,02
zinc finger protein 483	ZNF483	1,02
excision repair cross-complementation group 8	ERCC8	1,02
zinc finger protein 157	ZNF157	1,02
DEK proto-oncogene	DEK	1,02
zinc fingers and homeoboxes 3	ZHX3	1,02
sperm associated antigen 9	SPAG9	1,02
Memczak2013 ANTISENSE, coding, INTERNAL, intronic best transcript NM_003940	USP13	1,02
squamous cell carcinoma antigen recognized by T-cells 1	SART1	1,02
BCL2-related protein A1	BCL2A1	1,02
chromosome 5 open reading frame 49	C5orf49	1,02
host cell factor C1 regulator 1 (XPO1 dependent)	HCFC1R1	1,02
long intergenic non-protein coding RNA 612	LINC00612	1,02
v-kit Hardy-Zuckerman 4 feline sarcoma viral oncogene homolog	KIT	1,02
ribosomal RNA adenine dimethylase domain containing 1	RRNAD1	1,02
STIP1 homology and U-box containing protein 1, E3 ubiquitin protein ligase	STUB1	1,02
fascin actin-bundling protein 1	FSCN1	1,02
chromosome 1 open reading frame 43	C1orf43	1,02
quinolinate phosphoribosyltransferase	QPRT	1,02
nemo-like kinase	NLK	1,02
envoplakin	EVPL	1,02
polypeptide N-acetylgalactosaminyltransferase-like 6	GALNTL6	1,02
solute carrier family 39, member 11	SLC39A11	1,02
solute carrier family 6 (neurotransmitter transporter), member 1	SLC6A1	1,02
splicing factor 3a subunit 3	SF3A3	1,02
Meis homeobox 1	MEIS1	1,02
t-complex 11, testis-specific-like 1	TCP11L1	1,02
estrogen receptor 2 (ER beta)	ESR2	1,02
protein tyrosine phosphatase, non-receptor type 1	PTPN1	1,02
FRAS1 related extracellular matrix 1	FREM1	1,02
Rhox homeobox family, member 1	RHOXF1	1,02
tRNA 5-methylaminomethyl-2-thiouridylate methyltransferase	TRMU	1,02
formyl peptide receptor 2	FPR2	1,02
solute carrier family 7 (amino acid transporter light chain, L system), member 5	SLC7A5	1,02
coiled-coil domain containing 180	CCDC180	1,02

SLX1A-SULT1A3 readthrough (NMD candidate)	SLX1A-SULT1A3	1,02
cancer/testis antigen family 47, member B1	CT47B1	1,02
ethylmalonic encephalopathy 1	ETHE1	1,02
peroxisomal biogenesis factor 5-like	PEX5L	1,02
Sp3 transcription factor	SP3	1,02
zinc finger, MYND-type containing 15	ZMYND15	1,02
UTP4 small subunit (SSU) processome component	UTP4	1,02
ATPase, H+ transporting V0 subunit e2	ATP6V0E2	1,02
potassium channel, calcium activated intermediate/small conductance subfamily N alpha, member 4	KCNN4	1,02
P antigen family, member 5 (prostate associated)	PAGE5	1,02
PNN-interacting serine/arginine-rich protein	PNISR	1,02
tRNA methyltransferase 10A	TRMT10A	1,02
diacylglycerol kinase, iota	DGKI	1,02
nuclear pore complex interacting protein family, member A7; nuclear pore complex interacting protein family, member A8; polycystic kidney disease 1 (autosomal dominant) pseudogene 1	NPIPA7; NPIPA8; PKD1P1	1,02
SMAD family member 5	SMAD5	1,02
PHD finger protein 21B	PHF21B	1,02
dehydrogenase/reductase (SDR family) member 2	DHRS2	1,02
suppression of tumorigenicity 5	ST5	1,02
ribosomal protein L26-like 1	RPL26L1	1,02
ubiquitin specific peptidase 45	USP45	1,02
bromodomain containing 4	BRD4	1,02
general transcription factor IIi	GTF2I	1,02
ribosomal protein S4, Y-linked 1	RPS4Y1	1,02
general transcription factor IIH subunit 2	GTF2H2	1,02
nudC nuclear distribution protein	NUDC	1,02
farnesyl diphosphate synthase	FDPS	1,02
chromosome 2 open reading frame 61	C2orf61	1,02
apelin	APLN	1,02
interleukin 27	IL27	1,02
chemokine-like factor; CKLF-CMTM1 readthrough	CKLF; CKLF-CMTM1	1,02
zinc finger protein 699	ZNF699	1,02
lysine (K)-specific demethylase 4D	KDM4D	1,02
GrpE-like 1, mitochondrial (E. coli)	GRPEL1	1,02
transmembrane protein 230	TMEM230	1,02
arginyl aminopeptidase (aminopeptidase B)	RNPEP	1,02
natural cytotoxicity triggering receptor 3	NCR3	1,02
clavesin 1	CLVS1	1,02
transglutaminase 3	TGM3	1,02
ribosomal protein, large, P2; small nucleolar RNA, H/ACA box 52	RPLP2; SNORA52	1,02

potassium channel, two pore domain subfamily K, member 9	KCNK9	1,02
SH3-domain binding protein 4	SH3BP4	1,02
zinc finger protein 414	ZNF414	1,02
protein tyrosine phosphatase, receptor type, K	PTPRK	1,02
cilia and flagella associated protein 69	CFAP69	1,02
polymerase (RNA) II (DNA directed) polypeptide J, 13.3kDa	POLR2J	1,02
protection of telomeres 1	POT1	1,02
fumarylacetoacetate hydrolase domain containing 2B	FAHD2B	1,02
lysophosphatidylcholine acyltransferase 2	LPCAT2	1,02
calmodulin regulated spectrin-associated protein 1	CAMSAP1	1,02
adaptor-related protein complex 2, alpha 1 subunit	AP2A1	1,02
HMG box domain containing 3	HMGXB3	1,02
autophagy related 14	ATG14	1,02
golgin A8 family, member J; golgin A8 family, member I, pseudogene	GOLGA8J; GOLGA8IP	1,02
cytochrome P450, family 2, subfamily W, polypeptide 1	CYP2W1	1,02
interleukin 1 receptor-like 2	IL1RL2	1,02
methyltransferase like 22	METTL22	1,02
leucine zipper protein 4	LUZP4	1,02
C2 calcium-dependent domain containing 3	C2CD3	1,02
SET and MYND domain containing 3	SMYD3	1,02
acidic nuclear phosphoprotein 32 family member A	ANP32A	1,02
TP53 target 3B; TP53 target 3; TP53 target 3C	TP53TG3B; TP53TG3; TP53TG3C	1,02
GDNF family receptor alpha 4	GFRA4	1,02
actinin, alpha 3 (gene/pseudogene)	ACTN3	1,02
progressive rod-cone degeneration	PRCD	1,02
colony stimulating factor 1 (macrophage)	CSF1	1,02
phosphatidylinositol-3,4,5-trisphosphate-dependent Rac exchange factor 1	PREX1	1,02
pleckstrin homology domain containing, family M (with RUN domain) member 2	PLEKHM2	1,02
caspase 3	CASP3	1,02
tRNA-yW synthesizing protein 1 homolog (S. cerevisiae)	TYW1	1,02
MAGE family member B1; MAGE family member B4	MAGEB1; MAGEB4	1,02
protein tyrosine phosphatase domain containing 1	PTPDC1	1,02
zinc finger protein 7	ZNF7	1,02
serine/threonine/tyrosine interacting protein	STYX	1,02
IQ motif containing K	IQCK	1,02
adaptor-related protein complex 5, sigma 1 subunit	AP5S1	1,02
TSPY-like 5	TSPYL5	1,02
ankyrin repeat domain 11	ANKRD11	1,02

endonuclease/exonuclease/phosphatase family domain containing 1	EEPD1	1,02
dihydrouridine synthase 1-like	DUS1L	1,02
protein phosphatase, Mg2+/Mn2+ dependent, 1A	PPM1A	1,02
TNF receptor-associated factor 3	TRAF3	1,02
Memczak2013 ANTISENSE, CDS, coding, INTERNAL best transcript NM_015356	SCRIB	1,02
interleukin 10	IL10	1,02
ornithine decarboxylase antizyme 3	OAZ3	1,02
family with sequence similarity 50, member B	FAM50B	1,02
small proline-rich protein 2F	SPRR2F	1,02
nuclear receptor subfamily 1, group D, member 1	NR1D1	1,02
RAB11B, member RAS oncogene family	RAB11B	1,02
Transcript Identified by AceView, Entrez Gene ID(s) 84193	SETD3	1,02
cardiolipin synthase 1	CRLS1	1,02
ubiquilin-like	UBQLNL	1,02
SIL1 nucleotide exchange factor	SIL1	1,02
transmembrane p24 trafficking protein 9	TMED9	1,02
chondroitin polymerizing factor 2; microRNA 671	CHPF2; MIR671	1,02
glycine C-acetyltransferase	GCAT	1,02
potassium channel, voltage gated Shaw related subfamily C, member 3	KCNC3	1,02
RAP2A, member of RAS oncogene family	RAP2A	1,02
olfactory receptor, family 1, subfamily Q, member 1	OR1Q1	1,02
apoptosis-inducing factor, mitochondrion-associated, 1	AIFM1	1,02
potassium channel, sodium activated subfamily T, member 1	KCNT1	1,02
actin related protein 2/3 complex subunit 4	ARPC4	1,02
phosphatidylinositol glycan anchor biosynthesis class T	PIGT	1,02
zinc finger protein 17	ZNF17	1,02
killer cell lectin-like receptor subfamily C, member 3	KLRC3	1,02
tripartite motif containing 43B	TRIM43B	1,02
STT3A, subunit of the oligosaccharyltransferase complex (catalytic)	STT3A	1,02
protein geranylgeranyltransferase type I, beta subunit	PGGT1B	1,02
golgin A8 family, member N	GOLGA8N	1,02
family with sequence similarity 83, member E	FAM83E	1,02
nudix hydrolase 6	NUDT6	1,02
prosaposin	PSAP	1,02
olfactory receptor, family 4, subfamily A, member 5	OR4A5	1,02
long intergenic non-protein coding RNA 1496	LINC01496	1,02
cerebellin 2 precursor	CBLN2	1,02
cAMP responsive element binding protein 3-like 4	CREB3L4	1,02
fragile X mental retardation, autosomal homolog 2	FXR2	1,02
retinoblastoma binding protein 6	RBBP6	1,02

Memczak2013 ALT_ACCEPTOR, ALT_DONOR, coding, INTERNAL, intronic best transcript NM_001193522	FAM65A	1,02
zinc finger, AN1-type domain 1	ZFAND1	1,02
NK2 homeobox 6	NKX2-6	1,02
NADH dehydrogenase (ubiquinone) 1 beta subcomplex, 10, 22kDa	NDUFB10	1,02
mitogen-activated protein kinase 1 interacting protein 1-like	MAPK1IP1L	1,02
A kinase (PRKA) anchor protein 11	AKAP11	1,02
vacuolar protein sorting 37 homolog C (S. cerevisiae)	VPS37C	1,02
mitochondrial fission regulator 1	MTFR1	1,02
olfactory receptor, family 9, subfamily A, member 2	OR9A2	1,02
ryanodine receptor 3	RYR3	1,02
transmembrane protein 106C	TMEM106C	1,02
cytoskeleton associated protein 2	CKAP2	1,02
SMAD family member 7	SMAD7	1,02
ATPase, H+ transporting, lysosomal 31kDa, V1 subunit E1	ATP6V1E1	1,02
lanosterol synthase (2,3-oxidosqualene-lanosterol cyclase)	LSS	1,02
synaptogyrin 4	SYNGR4	1,02
wingless-type MMTV integration site family, member 8B	WNT8B	1,02
ubiquitin conjugating enzyme E2H	UBE2H	1,02
lysophosphatidic acid receptor 5	LPAR5	1,02
secretogranin III	SCG3	1,02
alcohol dehydrogenase 6 (class V)	ADH6	1,02
solute carrier family 35, member A5	SLC35A5	1,02
calicin	CCIN	1,02
coenzyme Q10B	COQ10B	1,02
phytanoyl-CoA 2-hydroxylase interacting protein	PHYHIP	1,02
ATPase, H+ transporting, lysosomal 21kDa, V0 subunit b	ATP6V0B	1,02
zinc finger protein 250	ZNF250	1,02
desmoglein 4	DSG4	1,02
eukaryotic translation initiation factor 3, subunit B	EIF3B	1,02
Transcript Identified by AceView, Entrez Gene ID(s) 6904	TBCD	1,02
marker of proliferation Ki-67	MKI67	1,02
arginine/serine-rich coiled-coil 1	RSRC1	1,02
glycosyltransferase 8 domain containing 1	GLT8D1	1,02
serpin peptidase inhibitor, clade F (alpha-2 antiplasmin, pigment epithelium derived factor), member 2	SERPINF2	1,02
gap junction protein beta 3	GJB3	1,02
procollagen-lysine, 2-oxoglutarate 5-dioxygenase 3	PLOD3	1,02
autophagy related 16-like 1	ATG16L1	1,02
proteasome 26S subunit, non-ATPase 4	PSMD4	1,02
Homo sapiens uncharacterized LOC79999 (LOC79999), mRNA.; uncharacterized protein ENSP00000382042; novel transcript	LOC79999; LOC388436; AC007952.5	1,02
plexin A4	PLXNA4	1,02

zinc finger, HIT-type containing 1	ZNHIT1	1,02
adaptor-related protein complex 1, gamma 1 subunit	AP1G1	1,02
family with sequence similarity 171, member A1	FAM171A1	1,02
inhibitor of growth family member 1	ING1	1,02
endogenous retrovirus group FRD, member 1	ERVFRD-1	1,02
coiled-coil domain containing 183; RAB, member RAS oncogene family-like 6	CCDC183; RABL6	1,02
unc-13 homolog A (C. elegans)	UNC13A	1,02
Usher syndrome 2A (autosomal recessive, mild)	USH2A	1,02
chemokine (C-C motif) ligand 18	CCL18	1,02
parkinson protein 7	PARK7	1,02
chromosome 20 open reading frame 173	C20orf173	1,02
solute carrier family 16, member 13	SLC16A13	1,02
RANBP2-like and GRIP domain containing 2; RANBP2-like and GRIP domain containing 1	RGPD2; RGPD1	1,02
BTG3 associated nuclear protein	BANP	1,02
OTU deubiquitinase 7A	OTUD7A	1,02
signal sequence receptor, beta (translocon-associated protein beta)	SSR2	1,02
zinc finger protein 197	ZNF197	1,02
ADP-ribosylation factor 1; microRNA 3620	ARF1; MIR3620	1,02
zinc finger, matrin-type 4	ZMAT4	1,02
zona pellucida binding protein	ZPBP	1,02
zw10 kinetochore protein	ZW10	1,02
proapoptotic nucleolar protein 1	PANO1	1,02
sorting nexin 16	SNX16	1,02
tryptase delta 1	TPSD1	1,02
SPANX family, member A2	SPANXA2	1,02
membrane associated ring finger 5	MARCH5	1,02
telomerase reverse transcriptase	TERT	1,02
phospholipase C, eta 2	PLCH2	1,02
late cornified envelope 1A	LCE1A	1,02
BOC cell adhesion associated, oncogene regulated	BOC	1,02
wingless-type MMTV integration site family, member 9B	WNT9B	1,02
chromosome 6 open reading frame 223	C6orf223	1,02
tubulin folding cofactor B	TBCB	1,02
nucleoporin 160kDa	NUP160	1,02
ELKS/RAB6-interacting/CAST family member 2	ERC2	1,02
MSS51 mitochondrial translational activator	MSS51	1,02
adrenoceptor alpha 2C	ADRA2C	1,02
F-box protein 33	FBXO33	1,02
dishevelled-binding antagonist of beta-catenin 1	DACT1	1,02
kinesin family member 13A	KIF13A	1,02
klotho beta	KLB	1,02

poly(ADP-ribose) polymerase family member 16	PARP16	1,02
insulin-like growth factor 2 mRNA binding protein 2	IGF2BP2	1,02
SDE2 telomere maintenance homolog (S. pombe)	SDE2	1,02
potassium channel tetramerization domain containing 3	KCTD3	1,02
protein-O-mannosyltransferase 2	POMT2	1,02
SPT4 homolog, DSIF elongation factor subunit	SUPT4H1	1,02
DC-STAMP domain containing 1	DCST1	1,02
chemokine (C-X-C motif) ligand 2	CXCL2	1,02
pleckstrin and Sec7 domain containing 3	PSD3	1,02
nucleolar protein 9	NOL9	1,02
solute carrier family 46 (folate transporter), member 1	SLC46A1	1,02
scavenger receptor cysteine rich family, 4 domains	SSC4D	1,02
ribosomal protein L3; small nucleolar RNA, C/D box 83B; small nucleolar RNA, C/D box 43	RPL3; SNORD83B; SNORD43	1,02
nucleoporin 54kDa	NUP54	1,02
olfactory receptor, family 52, subfamily E, member 4	OR52E4	1,02
CD46 molecule, complement regulatory protein	CD46	1,02
solute carrier family 17 (vesicular glutamate transporter), member 7	SLC17A7	1,02
glycerol kinase 5 (putative)	GK5	1,02
5-hydroxytryptamine (serotonin) receptor 2C, G protein-coupled	HTR2C	1,02
La ribonucleoprotein domain family, member 1B	LARP1B	1,02
ring finger and SPRY domain containing 1	RSPRY1	1,02
gastrulation brain homeobox 1	GBX1	1,02
LysM, putative peptidoglycan-binding, domain containing 1	LYSMD1	1,02
TOX high mobility group box family member 2	TOX2	1,02
guanine nucleotide binding protein (G protein), gamma 5	GNG5	1,02
chemokine (C-X-C motif) ligand 16	CXCL16	1,02
TRPC5 opposite strand	TRPC5OS	1,02
zinc finger protein 214	ZNF214	1,02
cleavage stimulation factor, 3 pre-RNA, subunit 3	CSTF3	1,02
solute carrier family 19 (folate transporter), member 1	SLC19A1	1,02
ribosomal protein S6	RPS6	1,02
UDP-glucuronate decarboxylase 1	UXS1	1,02
RAB23, member RAS oncogene family	RAB23	1,02
aspartyl-tRNA synthetase 2, mitochondrial	DARS2	1,02
membrane-spanning 4-domains, subfamily A, member 4E	MS4A4E	1,02
POP5 homolog, ribonuclease P/MRP subunit	POP5	1,02
solute carrier family 2 (facilitated glucose transporter), member 7	SLC2A7	1,02
adaptor-related protein complex 1 associated regulatory protein	AP1AR	1,02
Jeck2013 ALT_ACCEPTOR, ALT_DONOR, coding, INTERNAL, intronic best transcript NM_058175	COL6A2	1,02

Zhang2013 ALT_DONOR, coding, INTERNAL, intronic best transcript NM_002482	NASP	1,02
olfactory receptor, family 6, subfamily S, member 1	OR6S1	1,02
valosin containing protein (p97)/p47 complex interacting protein 1	VCPIP1	1,02
cytochrome b-245, alpha polypeptide	CYBA	1,02
mitogen-activated protein kinase kinase kinase 8	MAP3K8	1,02
tumor necrosis factor (ligand) superfamily, member 15	TNFSF15	1,02
ADP-ribosylation factor guanine nucleotide-exchange factor 2 (brefeldin A-inhibited)	ARFGEF2	1,02
membrane-spanning 4-domains, subfamily A, member 13	MS4A13	1,02
dihydropyrimidinase-like 3	DPYSL3	1,02
zinc finger protein 777	ZNF777	1,02
matrix Gla protein	MGP	1,02
chromosome alignment maintaining phosphoprotein 1	CHAMP1	1,02
protocadherin alpha 9; protocadherin alpha subfamily C, 1; protocadherin alpha subfamily C, 2; protocadherin alpha 1; protocadherin alpha 10; protocadherin alpha 11; protocadherin alpha 12; protocadherin alpha 13; protocadherin alpha 2; protocadherin alpha 3; protocadherin alpha 4; protocadherin alpha 5; protocadherin alpha 6; protocadherin alpha 7; protocadherin alpha 8; protocadherin alpha 14 pseudogene	PCDHA9; PCDHAC1; PCDHAC2; PCDHA1; PCDHA10; PCDHA11; PCDHA12; PCDHA13; PCDHA2; PCDHA3; PCDHA4; PCDHA5; PCDHA6; PCDHA7; PCDHA8; PCDHA14	1,02
suppressor of cytokine signaling 3	SOCS3	1,02
1-acylglycerol-3-phosphate O-acyltransferase 4	AGPAT4	1,02
ORMDL sphingolipid biosynthesis regulator 1	ORMDL1	1,02
phosphoglucomutase 1	PGM1	1,02
radial spoke head 10 homolog B2 (Chlamydomonas)	RSPH10B2	1,02
Memczak2013 ALT_ACCEPTOR, ALT_DONOR, coding, INTERNAL, intronic best transcript NM_020820	PREX1	1,02
Transcript Identified by AceView, Entrez Gene ID(s) 57448	BIRC6	1,02
ciliogenesis associated TTC17 interacting protein	CATIP	1,02
dishevelled associated activator of morphogenesis 1	DAAM1	1,02
serine threonine kinase 39	STK39	1,02
TGFB-induced factor homeobox 1	TGIF1	1,02
IKAROS family zinc finger 5	IKZF5	1,02
trefoil factor 2	TFF2	1,02

ankyrin repeat domain 20 family, member A1; ankyrin repeat domain 20 family, member A3	ANKRD20A1; ANKRD20A3	1,02
solute carrier family 12 (sodium/potassium/chloride transporter), member 2	SLC12A2	1,02
oligodendrocytic myelin paranodal and inner loop protein	OPALIN	1,02
Jeck2013 ALT_ACCEPTOR, ALT_DONOR, coding, INTERNAL, intronic best transcript NM_012474	UCK2	1,02
nudix hydrolase 10	NUDT10	1,02
glyoxylate reductase 1 homolog (Arabidopsis)	GLYR1	1,02
G protein-coupled receptor 1	GPR1	1,02
myeloid/lymphoid or mixed-lineage leukemia; translocated to, 6	MLLT6	1,02
serpin peptidase inhibitor, clade A (alpha-1 antiproteinase, antitrypsin), member 1	SERPINA1	1,02
hydroxyacylglutathione hydrolase-like	HAGHL	1,02
ras-related C3 botulinum toxin substrate 1 (rho family, small GTP binding protein Rac1)	RAC1	1,02
Transcript Identified by AceView, Entrez Gene ID(s) 9697	TRAM2	1,02
zinc finger family member 783	ZNF783	1,02
dynein, axonemal, heavy chain 12	DNAH12	1,02
gametocyte specific factor 1	GTSF1	1,02
RAB11 family interacting protein 4 (class II); microRNA 4724	RAB11FIP4; MIR4724	1,02
utrophin	UTRN	1,02
B-cell translocation gene 1, anti-proliferative; long intergenic non-protein coding RNA 1619	BTG1; LINC01619	1,02
coiled-coil and C2 domain containing 1B	CC2D1B	1,02
serine/threonine kinase 25	STK25	1,02
olfactory receptor, family 5, subfamily J, member 2	OR5J2	1,02
glutamate receptor, ionotropic, kainate 5	GRIK5	1,02
sialic acid binding Ig-like lectin 11	SIGLEC11	1,02
chemokine (C-C motif) receptor 9	CCR9	1,02
wingless-type MMTV integration site family, member 9A	WNT9A	1,02
NADH dehydrogenase (ubiquinone) 1 alpha subcomplex, 10, 42kDa	NDUFA10	1,02
Transcript Identified by AceView, Entrez Gene ID(s) 108	ADCY2	1,02
keratin 13, type I	KRT13	1,02
sulfotransferase family 1A member 2	SULT1A2	1,02
TEA domain family member 2	TEAD2	1,02
calcium channel, voltage-dependent, gamma subunit 7	CACNG7	1,02
zinc finger protein 705B	ZNF705B	1,02
hexamethylene bis-acetamide inducible 2	HEXIM2	1,02
caseinolytic mitochondrial matrix peptidase chaperone subunit	CLPX	1,02
protease, serine, 23	PRSS23	1,02
ribosomal protein L41 pseudogene 2	RPL41P2	1,02
peroxiredoxin 3	PRDX3	1,02

eva-1 homolog B (C. elegans)	EVA1B	1,02
chromosome 1 open reading frame 64	C1orf64	1,02
chromosome 7 open reading frame 33	C7orf33	1,02
acyl-CoA synthetase medium-chain family member 4	ACSM4	1,02
carboxypeptidase B2 (plasma)	CPB2	1,02
deleted in malignant brain tumors 1	DMBT1	1,02
Meis homeobox 3	MEIS3	1,02
Rho GTPase activating protein 32	ARHGAP32	1,02
TBC1 domain family, member 32	TBC1D32	1,02
proline rich 13	PRR13	1,02
chromosome 16 open reading frame 13	C16orf13	1,02
huntingtin interacting protein K	HYPK	1,02
poly(ADP-ribose) polymerase family member 9	PARP9	1,02
TBK1 binding protein 1	TBKBP1	1,02
CGRP receptor component	CRCP	1,02
UDP-Gal:betaGlcNAc beta 1,3-galactosyltransferase 5	B3GALT5	1,02
zinc finger protein 207; microRNA 632	ZNF207; MIR632	1,02
cytochrome b561 family, member A3	CYB561A3	1,02
5-aminolevulinate synthase 1	ALAS1	1,02
sphingosine-1-phosphate receptor 1	S1PR1	1,02
meningioma expressed antigen 5 (hyaluronidase)	MGEA5	1,02
family with sequence similarity 126, member A	FAM126A	1,02
cystatin C	CST3	1,02
beta-2-microglobulin	B2M	1,02
adhesion G protein-coupled receptor G5	ADGRG5	1,02
Rho GTPase activating protein 33	ARHGAP33	1,02
host cell factor C2	HCFC2	1,02
tocopherol (alpha) transfer protein-like	TTPAL	1,02
transmembrane protein 150A	TMEM150A	1,02
transmembrane 7 superfamily member 3	TM7SF3	1,02
GLI family zinc finger 3	GLI3	1,02
DLC1 Rho GTPase activating protein	DLC1	1,02
leucine rich repeat containing 8 family, member D	LRRC8D	1,02
coiled-coil domain containing 162, pseudogene	CCDC162P	1,02
glial cell derived neurotrophic factor	GDNF	1,02
ribophorin II	RPN2	1,02
keratinocyte associated protein 2	KRTCAP2	1,02
Zhang2013 ALT_ACCEPTOR, ALT_DONOR, coding, INTERNAL, intronic, OVERLAPTX, OVEXON best transcript NM_003315	DNAJC7	1,02
NADH dehydrogenase (ubiquinone) Fe-S protein 8, 23kDa (NADH-coenzyme Q reductase); microRNA 4691; microRNA 7113	NDUFS8; MIR4691; MIR7113	1,02
polyglutamine binding protein 1	PQBP1	1,02
apoptosis-inducing factor, mitochondrion-associated, 3	AIFM3	1,02

glutaminase	GLS	1,02
EFR3 homolog B	EFR3B	1,02
ATPase, H+/K+ exchanging, alpha polypeptide	ATP4A	1,02
ribosomal protein S2; small nucleolar RNA, H/ACA box 64; small nucleolar RNA, H/ACA box 10	RPS2; SNORA64; SNORA10	1,02
APITD1-CORT readthrough; cortistatin; apoptosis-inducing, TAF9-like domain 1	APITD1-CORT; CORT; APITD1	1,02
FOXL2 neighbor	FOXL2NB	1,02
acidic nuclear phosphoprotein 32 family member B	ANP32B	1,02
ribosomal protein S3A; small nucleolar RNA, C/D box 73A	RPS3A; SNORD73A	1,02
proteasome 26S subunit, non-ATPase 14	PSMD14	1,02
negative regulator of ubiquitin-like proteins 1	NUB1	1,02
autophagy related 5	ATG5	1,02
MAD2L1 binding protein	MAD2L1BP	1,02
THAP9 antisense RNA 1	THAP9-AS1	1,02
mitogen-activated protein kinase 15	MAPK15	1,02
carboxypeptidase X (M14 family), member 1	CPXM1	1,02
collagen, type XXIII, alpha 1	COL23A1	1,02
solute carrier organic anion transporter family, member 6A1	SLCO6A1	1,02
zinc finger protein 644	ZNF644	1,02
solute carrier family 22, member 18	SLC22A18	1,02
COP9 signalosome subunit 5	COPS5	1,02
family with sequence similarity 8, member A1	FAM8A1	1,02
SPANX family, member N5	SPANXN5	1,02
small integral membrane protein 7; mediator complex subunit 26	SMIM7; MED26	1,02
zinc finger protein 675	ZNF675	1,02
olfactory receptor, family 52, subfamily M, member 1	OR52M1	1,02
phosphatidylinositol transfer protein, beta	PITPNB	1,02
DMRT-like family C1; family with sequence similarity 226, member B (non-protein coding); family with sequence similarity 226, member A (non-protein coding); DMRT-like family C1B	DMRTC1; FAM226B; FAM226A; DMRTC1B	1,02
chromosome 14 open reading frame 39	C14orf39	1,02
ribosomal protein L30	RPL30	1,02
cytochrome P450, family 46, subfamily A, polypeptide 1	CYP46A1	1,02
ATP synthase, H+ transporting, mitochondrial Fo complex subunit D	ATP5H	1,02
ERGIC and golgi 3	ERGIC3	1,02
transcription factor AP-2 delta (activating enhancer binding protein 2 delta)	TFAP2D	1,02
ZFP36 ring finger protein-like 2	ZFP36L2	1,02
TOR signaling pathway regulator	TIPRL	1,02

IKAROS family zinc finger 4	IKZF4	1,02
uncharacterized LOC100288748; novel transcript, antisense to ESRP1	LOC100288748; RP11-22C11.2	1,02
aconitase 2, mitochondrial	ACO2	1,02
ankyrin repeat domain 13 family, member D	ANKRD13D	1,02
dual specificity phosphatase 15	DUSP15	1,02
S100 calcium binding protein P	S100P	1,02
zinc finger, SWIM-type containing 6	ZSWIM6	1,02
glutamic-oxaloacetic transaminase 2, mitochondrial	GOT2	1,02
hemoglobin, mu	HBM	1,02
olfactomedin 2	OLFM2	1,02
midnolin	MIDN	1,02
hydroxysteroid (17-beta) dehydrogenase 10	HSD17B10	1,02
transmembrane protein 206	TMEM206	1,02
protein phosphatase 2, regulatory subunit B, beta	PPP2R5B	1,02
spermatogenesis associated 25	SPATA25	1,02
ST8 alpha-N-acetyl-neuraminide alpha-2,8-sialyltransferase 1	ST8SIA1	1,02
engrailed homeobox 2	EN2	1,02
ZFP64 zinc finger protein	ZFP64	1,02
family with sequence similarity 160, member B1	FAM160B1	1,02
casein kinase 2, alpha 3 polypeptide	CSNK2A3	1,02
Ran GTPase activating protein 1	RANGAP1	1,02
zinc finger protein 705G	ZNF705G	1,02
origin recognition complex subunit 1	ORC1	1,02
keratin associated protein 9-6	KRTAP9-6	1,02
Transcript Identified by AceView, Entrez Gene ID(s) 7248	TSC1	1,02
Transcript Identified by AceView, Entrez Gene ID(s) 5604	MAP2K1	1,02
eukaryotic translation initiation factor 2B, subunit 2 beta, 39kDa	EIF2B2	1,02
inhibitor of DNA binding 4, dominant negative helix-loop-helix protein	ID4	1,02
Hermansky-Pudlak syndrome 6	HPS6	1,02
general transcription factor IIA 1-like; STON1-GTF2A1L readthrough; stonin 1	GTF2A1L; STON1-GTF2A1L; STON1	1,02
variable charge, X-linked 2	VCX2	1,02
phosphoinositide-3-kinase, regulatory subunit 1 (alpha)	PIK3R1	1,02
centrin 2	CETN2	1,02
cell adhesion molecule 3	CADM3	1,02
family with sequence similarity 109, member A	FAM109A	1,02
general transcription factor IIA 2	GTF2A2	1,02
keratin associated protein 19-7	KRTAP19-7	1,02
oxidative stress responsive 1	OXSR1	1,02
keratin associated protein 19-5	KRTAP19-5	1,02
ring finger protein 19B	RNF19B	1,02

protein serine kinase H2	PSKH2	1,02
brain-enriched guanylate kinase-associated	BEGAIN	1,02
aminoacylase 3	ACY3	1,02
ganglioside induced differentiation associated protein 1-like 1	GDAP1L1	1,02
transmembrane protein 8C	TMEM8C	1,02
exocyst complex component 2	EXOC2	1,02
ciliary rootlet coiled-coil, rootletin	CROCC	1,02
zinc finger protein 705E	ZNF705E	1,02
ribosomal protein S25	RPS25	1,02
ATP binding cassette subfamily A member 9	ABCA9	1,02
Transcript Identified by AceView, Entrez Gene ID(s) 51074	APIP	1,02
RB-associated KRAB zinc finger; RBAK-RBAKDN readthrough; RBAK downstream neighbor (non-protein coding)	RBAK; RBAK-RBAKDN; RBAKDN	1,02
furin (paired basic amino acid cleaving enzyme)	FURIN	1,02
solute carrier family 22 (organic anion/urate transporter), member 12	SLC22A12	1,02
Memczak2013 ANTISENSE, CDS, coding, INTERNAL best transcript NM_145295	ZNF627	1,02
solute carrier family 15 (oligopeptide transporter), member 3	SLC15A3	1,02
zinc finger, MYND-type containing 8	ZMYND8	1,02
Transcript Identified by AceView, Entrez Gene ID(s) 79922; novel transcript	CTB-75G16.3; MRM1	1,02
Zhang2013 ALT_ACCEPTOR, ALT_DONOR, coding, INTERNAL, intronic best transcript NM_025176	NINL	1,02
ADP-ribosylation factor like GTPase 15	ARL15	1,02
chromodomain helicase DNA binding protein 7	CHD7	1,02
HECT and RLD domain containing E3 ubiquitin protein ligase 3	HERC3	1,02
hepatoma-derived growth factor, related protein 3; Hepatoma-derived growth factor-related protein 3 [Source:UniProtKB/Swiss-Prot;Acc:Q9Y3E1]; Transcript Identified by AceView, Entrez Gene ID(s) 50810; Jeck2013, Memczak2013, Salzman2013 ANNOTATED, CDS, coding, INTERNAL, OVCODE, OVEXON best transcript NM_016073; Jeck2013 ALT_ACCEPTOR, ALT_DONOR, coding, INTERNAL, intronic best transcript NM_016073; Salzman2013 ANNOTATED, CDS, coding, INTERNAL, OVCODE, OVEXON best transcript NM_016073	HDGFRP3	1,02
fibroblast growth factor 17	FGF17	1,02
NADPH oxidase organizer 1	NOXO1	1,02
apolipoprotein A-IV	APOA4	1,02
chondroadherin	CHAD	1,02
BPI fold containing family B, member 6	BPIFB6	1,02
solute carrier family 9, subfamily A (NHE5, cation proton antiporter 5), member 5	SLC9A5	1,02

cholecystokinin B receptor	CCKBR	1,02
mediator complex subunit 28	MED28	1,02
ankyrin repeat and SOCS box containing 11, E3 ubiquitin protein ligase	ASB11	1,02
intercellular adhesion molecule 5	ICAM5	1,02
cytochrome b5 type A (microsomal)	CYB5A	1,02
uncharacterized LOC497048; uncharacterized LOC497048 [Source:EntrezGene;Acc:497048]; putative novel transcript	KU-MEL-3; RP4-529N6.1	1,02
3-phosphoadenosine 5-phosphosulfate synthase 1	PAPSS1	1,02
fucosyltransferase 9 (alpha (1,3) fucosyltransferase)	FUT9	1,02
matrix metallopeptidase 2	MMP2	1,02
hypoxia up-regulated 1	HYOU1	1,02
olfactory receptor, family 6, subfamily M, member 1	OR6M1	1,02
NSE4 homolog A, SMC5-SMC6 complex component	NSMCE4A	1,02
epidermal growth factor receptor pathway substrate 8	EPS8	1,02
olfactory receptor, family 5, subfamily AK, member 2	OR5AK2	1,02
Transcript Identified by AceView, Entrez Gene ID(s) 11157	LSM6	1,02
REX1, RNA exonuclease 1 homolog-like 10, pseudogene	REXO1L10P	1,02
zinc finger, AN1-type domain 6	ZFAND6	1,02
mitochondrial ribosomal protein L10	MRPL10	1,02
adenine phosphoribosyltransferase	APRT	1,02
prolyl 4-hydroxylase, alpha polypeptide II	P4HA2	1,02
ST8 alpha-N-acetyl-neuraminide alpha-2,8-sialyltransferase 4	ST8SIA4	1,02
SEC11 homolog A, signal peptidase complex subunit	SEC11A	1,02
zinc finger with KRAB and SCAN domains 3	ZKSCAN3	1,02
family with sequence similarity 73, member B	FAM73B	1,02
uncharacterized LOC101927322; novel transcript, antisense to MALT1	LOC101927322; RP11-126O1.5	1,02
Jrk helix-turn-helix protein	JRK	1,02
transmembrane protein 154	TMEM154	1,02
KIAA1683	KIAA1683	1,02
stathmin domain containing 1	STMND1	1,02
anaphase promoting complex subunit 5	ANAPC5	1,02
gap junction protein beta 1	GJB1	1,02
protein phosphatase 3, catalytic subunit, alpha isozyme	PPP3CA	1,02
Memczak2013 ALT_ACCEPTOR, ALT_DONOR, coding, INTERNAL, intronic best transcript NM_002865	RAB2A	1,02
cytochrome P450, family 3, subfamily A, polypeptide 5	CYP3A5	1,02
hemoglobin, gamma G; hemoglobin, gamma A	HBG2; HBG1	1,02
intraflagellar transport 74	IFT74	1,02
peptidase (mitochondrial processing) alpha	PMPCA	1,02
ATPase type 13A3	ATP13A3	1,02
zinc finger protein 738	ZNF738	1,02
emopamil binding protein (sterol isomerase)	EBP	1,02

keratin 14, type I	KRT14	1,02
transaldolase 1	TALDO1	1,02
poly(ADP-ribose) polymerase family member 10	PARP10	1,02
sphingomyelin synthase 1	SGMS1	1,02
v-akt murine thymoma viral oncogene homolog 1	AKT1	1,02
surfactant protein C	SFTPC	1,02
homeobox containing 1	HMBOX1	1,02
beta-carotene oxygenase 1	BCO1	1,02
derlin 1	DERL1	1,02
protamine 3	PRM3	1,02
protein phosphatase 1, regulatory subunit 27	PPP1R27	1,02
F-box and WD repeat domain containing 12	FBXW12	1,01
USP6 N-terminal like	USP6NL	1,01
phosphatidylinositol glycan anchor biosynthesis class C	PIGC	1,01
Fc fragment of IgG binding protein	FCGBP	1,01
coagulation factor X	F10	1,01
RBM14-RBM4 readthrough	RBM14-RBM4	1,01
XIAP associated factor 1	XAF1	1,01
spastic paraplegia 20 (Troyer syndrome)	SPG20	1,01
ras-related C3 botulinum toxin substrate 2 (rho family, small GTP binding protein Rac2)	RAC2	1,01
LIM domain binding 1	LDB1	1,01
CD36 molecule (thrombospondin receptor)	CD36	1,01
transmembrane 4 L six family member 18	TM4SF18	1,01
ribosomal protein L31	RPL31	1,01
chromosome 21 open reading frame 58	C21orf58	1,01
ATP binding cassette subfamily A member 5	ABCA5	1,01
hephaestin-like 1	HEPHL1	1,01
ATP5J2-PTCD1 readthrough; pentatricopeptide repeat domain 1	ATP5J2-PTCD1; PTCD1	1,01
golgin A6 family-like 6	GOLGA6L6	1,01
protein disulfide isomerase family A, member 2	PDIA2	1,01
activating transcription factor 7 interacting protein 2	ATF7IP2	1,01
LYR motif containing 2	LYRM2	1,01
armadillo repeat containing 6	ARMC6	1,01
drebrin-like; microRNA 6837	DBNL; MIR6837	1,01
formin 1	FMN1	1,01
ADP-ribosylation factor like GTPase 6 interacting protein 4	ARL6IP4	1,01
parvin, beta	PARVB	1,01
claudin 4	CLDN4	1,01
magnesium transporter 1	MAGT1	1,01
colipase-like 2	CLPSL2	1,01
PHD finger protein 10	PHF10	1,01

solute carrier family 25 (mitochondrial carrier; phosphate carrier), member 3; small nucleolar RNA, H/ACA box 53	SLC25A3; SNORA53	1,01
chromosome 2 open reading frame 66	C2orf66	1,01
helicase with zinc finger 2, transcriptional coactivator	HELZ2	1,01
tyrosylprotein sulfotransferase 2	TPST2	1,01
vaccinia related kinase 2	VRK2	1,01
macrophage erythroblast attacher	MAEA	1,01
cytokine receptor-like factor 2	CRLF2	1,01
olfactory receptor, family 5, subfamily A, member 2	OR5A2	1,01
replication timing regulatory factor 1	RIF1	1,01
patched 2	PTCH2	1,01
Transcript Identified by AceView, Entrez Gene ID(s) 54940	OCIAD1	1,01
surfactant associated 2	SFTA2	1,01
exostosin glycosyltransferase 2	EXT2	1,01
coiled-coil domain containing 141	CCDC141	1,01
Sad1 and UNC84 domain containing 5	SUN5	1,01
chromobox homolog 1	CBX1	1,01
cullin 2	CUL2	1,01
long intergenic non-protein coding RNA 1561	LINC01561	1,01
sorting nexin 7	SNX7	1,01
golgi transport 1B	GOLT1B	1,01
dehydrogenase/reductase (SDR family) member 4 like 1	DHRS4L1	1,01
nuclear receptor subfamily 2, group C, member 2	NR2C2	1,01
protein kinase, AMP-activated, gamma 3 non-catalytic subunit	PRKAG3	1,01
catenin (cadherin-associated protein), beta 1	CTNNB1	1,01
chromosome 3 open reading frame 18	C3orf18	1,01
guanylate cyclase activator 2A (guanylin)	GUCA2A	1,01
keratin associated protein 20-4	KRTAP20-4	1,01
Memczak2013 ALT_ACCEPTOR, ALT_DONOR, coding, INTERNAL, intronic best transcript NM_001190457	CORO2B	1,01
SIX homeobox 4	SIX4	1,01
inhibitor of kappa light polypeptide gene enhancer in B-cells, kinase complex-associated protein	IKBKAP	1,01
5-3 exoribonuclease 1	XRN1	1,01
zinc finger protein 613	ZNF613	1,01
DALR anticodon binding domain containing 3	DALRD3	1,01
angiomotin like 2; microRNA 6827; ribosomal protein L39 pseudogene 5	AMOTL2; MIR6827; RPL39P5	1,01
neurexin 3	NRXN3	1,01
olfactory receptor, family 7, subfamily D, member 2	OR7D2	1,01
Transcript Identified by AceView, Entrez Gene ID(s) 6777	STAT5B	1,01
family with sequence similarity 133, member B	FAM133B	1,01
mediator complex subunit 15	MED15	1,01

Memczak2013 ALT_ACCEPTOR, ALT_DONOR, coding, INTERNAL, intronic best transcript NM_000655	SELL	1,01
heterogeneous nuclear ribonucleoprotein H1 (H)	HNRNPH1	1,01
pyroglutamyl-peptidase I-like	PGPEP1L	1,01
leishmanolysin-like (metallopeptidase M8 family)	LMLN	1,01
chloride channel, voltage-sensitive 3	CLCN3	1,01
transmembrane protein 176B	TMEM176B	1,01
zinc finger protein 565	ZNF565	1,01
flavin containing monooxygenase 4	FMO4	1,01
mitogen-activated protein kinase kinase kinase 9	MAP3K9	1,01
BMP binding endothelial regulator	BMPER	1,01
mitochondrial pyruvate carrier 1-like	MPC1L	1,01
proteasome 26S subunit, non-ATPase 1	PSMD1	1,01
ADAM metallopeptidase with thrombospondin type 1 motif 2	ADAMTS2	1,01
solute carrier family 25 (mitochondrial carrier; peroxisomal membrane protein, 34kDa), member 17	SLC25A17	1,01
ribosomal protein S17	RPS17	1,01
C1D nuclear receptor corepressor	C1D	1,01
RBBP8 N-terminal like	RBBP8NL	1,01
PAP associated domain containing 4	PAPD4	1,01
zinc finger protein 720	ZNF720	1,01
peptidylprolyl isomerase B (cyclophilin B)	PPIB	1,01
integrin alpha 3	ITGA3	1,01
myeloid/lymphoid or mixed-lineage leukemia; translocated to, 4	MLLT4	1,01
NADH dehydrogenase (ubiquinone) 1 alpha subcomplex, 6, 14kDa	NDUFA6	1,01
iron-sulfur cluster assembly 2	ISCA2	1,01
Transcript Identified by AceView, Entrez Gene ID(s) 171586	ABHD3	1,01
cytochrome c oxidase subunit VIb polypeptide 2 (testis)	COX6B2	1,01
proline-rich coiled-coil 2C	PRRC2C	1,01
LY6/PLAUR domain containing 2	LYPD2	1,01
microtubule associated monooxygenase, calponin and LIM domain containing 3	MICAL3	1,01
DnaJ (Hsp40) homolog, subfamily C, member 10	DNAJC10	1,01
mixed lineage kinase 4; Mitogen-activated protein kinase kinase kinase MLK4 [Source:UniProtKB/Swiss-Prot;Acc:Q5TCX8]; Salzman2013 ANNOTATED, CDS, coding, INTERNAL, OVCODE, OVEXON best transcript NM_032435	KIAA1804; MLK4	1,01
Memczak2013 ANTISENSE, CDS, coding, INTERNAL best transcript NM_001005417	B4GALT2	1,01
reticulon 4	RTN4	1,01
signal-induced proliferation-associated 1	SIPA1	1,01
NEDD4 binding protein 1	N4BP1	1,01
inositol-trisphosphate 3-kinase C	ITPKC	1,01
DIS3 like 3-5 exoribonuclease 2	DIS3L2	1,01

K(lysine) acetyltransferase 6A	KAT6A	1,01
tripartite motif containing 41	TRIM41	1,01
cysteine-rich PDZ-binding protein	CRIPT	1,01
transducer of ERBB2, 1	TOB1	1,01
KIAA1958	KIAA1958	1,01
tumor necrosis factor, alpha-induced protein 1 (endothelial)	TNFAIP1	1,01
Memczak2013 ALT_ACCEPTOR, ALT_DONOR, coding, INTERNAL, intronic best transcript NM_022479	WBSCR17	1,01
protein arginine methyltransferase 8	PRMT8	1,01
suppressor of variegation 3-9 homolog 1 (Drosophila)	SUV39H1	1,01
high mobility group box 3	HMGB3	1,01
zinc finger protein 185 (LIM domain)	ZNF185	1,01
CGG triplet repeat binding protein 1	CGGBP1	1,01
prostate tumor overexpressed 1; microRNA 4749	PTOV1; MIR4749	1,01
solute carrier family 1 (glutamate transporter), member 7	SLC1A7	1,01
Sec23 homolog B, COPII coat complex component	SEC23B	1,01
TSC22 domain family, member 4	TSC22D4	1,01
protein phosphatase 1, regulatory subunit 8	PPP1R8	1,01
matrilin 1, cartilage matrix protein	MATN1	1,01
splicing factor 3a subunit 2	SF3A2	1,01
ARP3 actin-related protein 3 homolog B (yeast)	ACTR3B	1,01
olfactory receptor, family 2, subfamily AG, member 2	OR2AG2	1,01
helicase-like transcription factor	HLTF	1,01
folliculin interacting protein 2	FNIP2	1,01
Sin3A associated protein 130kDa	SAP130	1,01
insulin receptor substrate 4	IRS4	1,01
dynein, cytoplasmic 1, light intermediate chain 2	DYNC1LI2	1,01
solute carrier family 25 (mitochondrial thiamine pyrophosphate carrier), member 19	SLC25A19	1,01
zinc finger protein 629	ZNF629	1,01
tRNA methyltransferase 1	TRMT1	1,01
distal-less homeobox 3	DLX3	1,01
chromosome 1 open reading frame 216	C1orf216	1,01
ADP-ribosylation factor like GTPase 13B	ARL13B	1,01
ATP binding cassette subfamily B member 6 (Langereis blood group)	ABCB6	1,01
transmembrane protein 78	TMEM78	1,01
anaphase promoting complex subunit 4	ANAPC4	1,01
ankyrin repeat domain 22	ANKRD22	1,01
unkempt family zinc finger-like	UNKL	1,01
histone cluster 1, H4e	HIST1H4E	1,01
aldo-keto reductase family 7-like (gene/pseudogene)	AKR7L	1,01
leucine rich repeat containing 41	LRRC41	1,01
oxidation resistance 1	OXR1	1,01

DPY30 domain containing 1	DYDC1	1,01
DnaJ (Hsp40) homolog, subfamily C, member 5; microRNA 941-1; microRNA 941-2; microRNA 941-3; microRNA 941-4; microRNA 941-5	DNAJC5; MIR941-1; MIR941-2; MIR941-3; MIR941-4; MIR941-5	1,01
general transcription factor IIH subunit 5	GTF2H5	1,01
leucine rich repeat containing 57	LRRC57	1,01
tyrosine aminotransferase	TAT	1,01
WW domain binding protein 2	WBP2	1,01
Mov10 RISC complex RNA helicase	MOV10	1,01
olfactory receptor, family 10, subfamily J, member 5	OR10J5	1,01
protein tyrosine kinase 6	PTK6	1,01
transmembrane protein 184B	TMEM184B	1,01
chromosome 1 open reading frame 233	C1orf233	1,01
ubiquitin specific peptidase 17-like family member 28; ubiquitin specific peptidase 17-like family member 5; ubiquitin specific peptidase 17-like family member 24	USP17L28; USP17L5; USP17L24	1,01
von Willebrand factor D and EGF domains	VWDE	1,01
eukaryotic translation initiation factor 1A, Y-linked	EIF1AY	1,01
ARV1 homolog, fatty acid homeostasis modulator	ARV1	1,01
neuron navigator 1	NAV1	1,01
synaptosome associated protein 91kDa	SNAP91	1,01
matrix metallopeptidase 23B; matrix metallopeptidase 23A (pseudogene)	MMP23B; MMP23A	1,01
metal-regulatory transcription factor 1	MTF1	1,01
cytochrome P450, family 21, subfamily A, polypeptide 1 pseudogene; cytochrome P450, family 21, subfamily A, polypeptide 2	CYP21A1P; CYP21A2	1,01
chromosome 7 open reading frame 13	C7orf13	1,01
POTE ankyrin domain family, member A	POTEA	1,01
transcription elongation factor A (SII)-like 6	TCEAL6	1,01
SUZ12 polycomb repressive complex 2 subunit	SUZ12	1,01
ribosomal protein S11; small nucleolar RNA, C/D box 35B	RPS11; SNORD35B	1,01
tripartite motif containing 61	TRIM61	1,01
glutaredoxin 5	GLRX5	1,01
keratin 78, type II	KRT78	1,01
NME/NM23 nucleoside diphosphate kinase 6	NME6	1,01
DAZ interacting zinc finger protein 3	DZIP3	1,01
C1q and tumor necrosis factor related protein 2	C1QTNF2	1,01
serine/threonine kinase-like domain containing 1 [Source:HGNC Symbol;Acc:HGNC:28669]	STKLD1	1,01
HRAS-like suppressor	HRASLS	1,01
heterochromatin protein 1, binding protein 3	HP1BP3	1,01

YY1 associated protein 1	YY1AP1	1,01
baculoviral IAP repeat containing 6	BIRC6	1,01
GRB10 interacting GYF protein 1	GIGYF1	1,01
zinc finger protein 331	ZNF331	1,01
protein phosphatase 1, regulatory subunit 3B	PPP1R3B	1,01
cytotoxic T-lymphocyte-associated protein 4	CTLA4	1,01
mitochondrial ribosome-associated GTPase 2	MTG2	1,01
R-spondin 3	RSPO3	1,01
distal-less homeobox 5	DLX5	1,01
prospero homeobox 1	PROX1	1,01
protein phosphatase 1, regulatory subunit 3G	PPP1R3G	1,01
Bcl2 modifying factor	BMF	1,01
exocyst complex component 6	EXOC6	1,01
Rho GTPase activating protein 5	ARHGAP5	1,01
ECSIT signalling integrator	ECSIT	1,01
growth hormone releasing hormone receptor	GHRHR	1,01
plasmolipin	PLLP	1,01
p21 protein (Cdc42/Rac)-activated kinase 6; BUB1 mitotic checkpoint serine/threonine kinase B	PAK6; BUB1B	1,01
calcineurin binding protein 1	CABIN1	1,01
solute carrier organic anion transporter family, member 4C1	SLCO4C1	1,01
potassium channel, voltage gated subfamily E regulatory beta subunit 2	KCNE2	1,01
zinc finger protein 780B	ZNF780B	1,01
BTB and CNC homology 1, basic leucine zipper transcription factor 1	BACH1	1,01
protein tyrosine phosphatase, receptor type, J	PTPRJ	1,01
calmodulin binding transcription activator 2	CAMTA2	1,01
protease, serine 46	PRSS46	1,01
chromosome 1 open reading frame 167	C1orf167	1,01
Memczak2013 ANTISENSE, CDS, coding, INTERNAL, intronic best transcript NM_003049	SLC10A1	1,01
long intergenic non-protein coding RNA 1547	LINC01547	1,01
GTPase activating protein (SH3 domain) binding protein 2	G3BP2	1,01
Zhang2013 ALT_ACCEPTOR, ALT_DONOR, coding, INTERNAL, intronic best transcript NM_012398	PIP5K1C	1,01
CD63 molecule	CD63	1,01
cholinergic receptor, muscarinic 2	CHRM2	1,01
signal peptidase complex subunit 2	SPCS2	1,01
MT-RNR2-like 2; MT-RNR2-like 12	MTRNR2L2; MTRNR2L12	1,01
kizuna centrosomal protein	KIZ	1,01
CKLF-like MARVEL transmembrane domain containing 1	CMTM1	1,01
endothelin 1	EDN1	1,01
BUD31 homolog	BUD31	1,01

PRELI domain containing 2	PRELID2	1,01
ribosomal protein L19	RPL19	1,01
DNA replication helicase/nuclease 2	DNA2	1,01
uncharacterized LOC101927572; Memczak2013 ANTISENSE, CDS, coding, INTERNAL, intronic best transcript NM_015526; novel transcript, antisense to CLIP3 and THAP8	LOC101927572; AC002116.7; CLIP3	1,01
zinc finger protein 439	ZNF439	1,01
general transcription factor IIA 1	GTF2A1	1,01
ribosome binding protein 1	RRBP1	1,01
eukaryotic translation initiation factor 2B, subunit 1 alpha, 26kDa	EIF2B1	1,01
zinc finger protein 582	ZNF582	1,01
KCTD21 antisense RNA 1	KCTD21-AS1	1,01
butyrophilin, subfamily 3, member A1	BTN3A1	1,01
methyl-CpG binding domain protein 2	MBD2	1,01
C-type lectin-like 1	CLECL1	1,01
stromal cell derived factor 4	SDF4	1,01
proline-rich coiled-coil 2B	PRRC2B	1,01
NADH dehydrogenase (ubiquinone) 1 alpha subcomplex, 6, 14kDa	NDUFA6	1,01
origin recognition complex subunit 3	ORC3	1,01
dehydrogenase/reductase (SDR family) member 3; microRNA 6730	DHRS3; MIR6730	1,01
KIAA1522	KIAA1522	1,01
NDC1 transmembrane nucleoporin	NDC1	1,01
zinc finger protein 496	ZNF496	1,01
BTG family, member 2	BTG2	1,01
Gse1 coiled-coil protein	GSE1	1,01
inositol(myo)-1(or 4)-monophosphatase 1	IMPA1	1,01
neural cell adhesion molecule 2	NCAM2	1,01
INO80 complex subunit	INO80	1,01
erythrocyte membrane protein band 4.1-like 3	EPB41L3	1,01
PEST proteolytic signal containing nuclear protein	PCNP	1,01
mindbomb E3 ubiquitin protein ligase 1	MIB1	1,01
chloride intracellular channel 3	CLIC3	1,01
chromosome 16 open reading frame 72	C16orf72	1,01
Rho guanine nucleotide exchange factor 28	ARHGEF28	1,01
SRY box 9	SOX9	1,01
homeobox B13	HOXB13	1,01
BCL2-associated X protein	BAX	1,01
interleukin 16	IL16	1,01
CXXC finger protein 4	CXXC4	1,01
TNFRSF1A-associated via death domain	TRADD	1,01
ribonuclease P/MRP 40kDa subunit	RPP40	1,01
copine family member IX	CPNE9	1,01

receptor-interacting serine-threonine kinase 2	RIPK2	1,01
TBC1 domain family, member 29	TBC1D29	1,01
cingulin	CGN	1,01
ABO blood group (transferase A, alpha 1-3-N-acetylgalactosaminyltransferase; transferase B, alpha 1-3-galactosyltransferase)	ABO	1,01
zinc finger, C2HC-type containing 1C	ZC2HC1C	1,01
tubulin, alpha 1b	TUBA1B	1,01
coiled-coil domain containing 130	CCDC130	1,01
inositol-tetrakisphosphate 1-kinase	ITPK1	1,01
solute carrier family 16 (aromatic amino acid transporter), member 10	SLC16A10	1,01
chromosome 8 open reading frame 76	C8orf76	1,01
charged multivesicular body protein 1B	CHMP1B	1,01
phosphatidylinositol glycan anchor biosynthesis class S	PIGS	1,01
thyrotropin-releasing hormone receptor	TRHR	1,01
RAD9-HUS1-RAD1 interacting nuclear orphan 1	RHNO1	1,01
syntaxin 12	STX12	1,01
F-box and WD repeat domain containing 5	FBXW5	1,01
keratin associated protein 4-8	KRTAP4-8	1,01
ZFP91-CNTF readthrough (NMD candidate)	ZFP91-CNTF	1,01
defensin, alpha 1B; defensin, alpha 1; defensin, alpha 3, neutrophil-specific	DEFA1B; DEFA1; DEFA3	1,01
defensin, alpha 1; defensin, alpha 1B	DEFA1; DEFA1B	1,01
leukocyte cell derived chemotaxin 1	LECT1	1,01
myosin light chain kinase family member 4	MYLK4	1,01
keratin 1, type II	KRT1	1,01
phospholipase A2, group XIIB	PLA2G12B	1,01
CDGSH iron sulfur domain 2	CISD2	1,01
regulation of nuclear pre-mRNA domain containing 1A	RPRD1A	1,01
SMG5 nonsense mediated mRNA decay factor	SMG5	1,01
family with sequence similarity 177, member B	FAM177B	1,01
coenzyme Q7 homolog, ubiquinone (yeast)	COQ7	1,01
BCL2-like 2	BCL2L2	1,01
chromosome 12 open reading frame 49	C12orf49	1,01
acyl-CoA synthetase family member 2	ACSF2	1,01
lectin, mannose-binding 2-like	LMAN2L	1,01
G protein-coupled receptor 183	GPR183	1,01
shootin 1	SHTN1	1,01
zinc finger protein 808; ribosomal protein L39 pseudogene 34	ZNF808; RPL39P34	1,01
olfactory receptor, family 52, subfamily K, member 1	OR52K1	1,01
family with sequence similarity 213, member A	FAM213A	1,01
pentatricopeptide repeat domain 2	PTCD2	1,01

CCAAT/enhancer binding protein (C/EBP), gamma	CEBPG	1,01
protein disulfide isomerase family A member 3	PDIA3	1,01
cortexin 2	CTXN2	1,01
zinc finger protein 727	ZNF727	1,01
protein phosphatase 1, regulatory subunit 7	PPP1R7	1,01
zinc finger protein 442	ZNF442	1,01
ribosomal RNA processing 9, small subunit (SSU) processome component, homolog (yeast)	RRP9	1,01
WD repeat domain 45; PRA1 domain family, member 2	WDR45; PRAF2	1,01
homeodomain interacting protein kinase 4	HIPK4	1,01
fatty acid binding protein 5 (psoriasis-associated)	FABP5	1,01
TMEM9 domain family, member B	TMEM9B	1,01
Epstein-Barr virus induced 3	EBI3	1,01
kinesin family member 1A	KIF1A	1,01
solute carrier family 25 (pyrimidine nucleotide carrier), member 36	SLC25A36	1,01
FERM and PDZ domain containing 3	FRMPD3	1,01
5-hydroxytryptamine (serotonin) receptor 1E, G protein-coupled	HTR1E	1,01
amyloid beta (A4) precursor protein	APP	1,01
cerebral dopamine neurotrophic factor	CDNF	1,01
CD86 molecule	CD86	1,01
POU class 4 homeobox 3	POU4F3	1,01
leucine rich repeat containing 8 family, member C	LRRC8C	1,01
euchromatic histone-lysine N-methyltransferase 2	EHMT2	1,01
coiled-coil domain containing 7	CCDC7	1,01
ELAV like neuron-specific RNA binding protein 2	ELAVL2	1,01
casein kinase 2, alpha 1 polypeptide	CSNK2A1	1,01
olfactory receptor, family 2, subfamily G, member 6	OR2G6	1,01
chitobiase, di-N-acetyl-	CTBS	1,01
zinc finger and BTB domain containing 8 opposite strand	ZBTB8OS	1,01
long intergenic non-protein coding RNA 272	LINC00272	1,01
methyl-CpG binding domain protein 3-like 5	MBD3L5	1,01
RAB4A, member RAS oncogene family; S-phase response (cyclin related)	RAB4A; SPHAR	1,01
fucosyltransferase 5 (alpha (1,3) fucosyltransferase)	FUT5	1,01
SNW domain containing 1	SNW1	1,01
T-cell lymphoma invasion and metastasis 1	TIAM1	1,01
choline kinase beta	CHKB	1,01
solute carrier family 8 (sodium/calcium exchanger), member 1	SLC8A1	1,01
protein kinase (cAMP-dependent, catalytic) inhibitor alpha	PKIA	1,01
Memczak2013 ANTISENSE, CDS, coding, INTERNAL best transcript NM_007277	EXOC3	1,01
UTP14A small subunit (SSU) processome component	UTP14A	1,01
diphthamide biosynthesis 1; ovarian tumor suppressor candidate 2	DPH1; OVCA2	1,01

catenin, beta interacting protein 1	CTNNBIP1	1,01
ChaC glutathione-specific gamma-glutamylcyclotransferase 1	CHAC1	1,01
SMG7 nonsense mediated mRNA decay factor	SMG7	1,01
EWS RNA binding protein 1	EWSR1	1,01
5-methyltetrahydrofolate-homocysteine methyltransferase	MTR	1,01
chromosome 1 open reading frame 195	C1orf195	1,01
Y box binding protein 2	YBX2	1,01
chromosome 6 open reading frame 203	C6orf203	1,01
ribosomal protein L7	RPL7	1,01
lin-9 DREAM MuvB core complex component	LIN9	1,01
NADH dehydrogenase (ubiquinone) complex I, assembly factor 5	NDUFAF5	1,01
CKLF-CMTM1 readthrough	CKLF-CMTM1	1,01
coronin, actin binding protein, 2A	CORO2A	1,01
uromodulin-like 1	UMODL1	1,01
chromosome 16 open reading frame 71	C16orf71	1,01
zinc finger protein 562	ZNF562	1,01
interferon induced transmembrane protein 3	IFITM3	1,01
ADP-ribosylation factor like GTPase 6 interacting protein 1	ARL6IP1	1,01
ripply transcriptional repressor 1	RIPPLY1	1,01
RAS and EF-hand domain containing	RASEF	1,01
COP9 signalosome subunit 7B	COPS7B	1,01
mesoderm induction early response 1, transcriptional regulator	MIER1	1,01
immunoglobulin superfamily containing leucine-rich repeat	ISLR	1,01
FK506 binding protein 9	FKBP9	1,01
tubulin, beta 2A class IIa	TUBB2A	1,01
mitochondrial translational initiation factor 2	MTIF2	1,01
olfactory receptor, family 51, subfamily B, member 2 (gene/pseudogene)	OR51B2	1,01
KIAA2026	KIAA2026	1,01
TBC1 domain family, member 22A	TBC1D22A	1,01
ribosomal protein S29	RPS29	1,01
t-complex 10	TCP10	1,01
GABA(A) receptor-associated protein like 2	GABARAPL2	1,01
transmembrane protein 190	TMEM190	1,01
Kruppel-like factor 4 (gut)	KLF4	1,01
GID complex subunit 4 homolog	GID4	1,01
prolyl 4-hydroxylase, alpha polypeptide III	P4HA3	1,01
cyclic nucleotide gated channel alpha 1	CNGA1	1,01
discoidin, CUB and LCCL domain containing 1	DCBLD1	1,01
histone cluster 2, H2be	HIST2H2BE	1,01
centromere protein B	CENPB	1,01
hook microtubule-tethering protein 3	HOOK3	1,01
HAUS augmin like complex subunit 4; microRNA 4707	HAUS4; MIR4707	1,01
deltex 1, E3 ubiquitin ligase	DTX1	1,01

Myb/SANT-like DNA-binding domain containing 1	MSANTD1	1,01
ADP-ribosylation factor GTPase activating protein 1; microRNA 4326	ARFGAP1; MIR4326	1,01
neural EGFL like 2	NELL2	1,01
eukaryotic translation initiation factor 3, subunit F	EIF3F	1,01
hemicentin 1	HMCN1	1,01
talin 2	TLN2	1,01
ELK4, ETS-domain protein (SRF accessory protein 1)	ELK4	1,01
ring finger protein, transmembrane 1	RNFT1	1,01
adenylate cyclase 2 (brain)	ADCY2	1,01
adenosine deaminase domain containing 1	ADAD1	1,01
SWI/SNF related, matrix associated, actin dependent regulator of chromatin, subfamily c, member 2	SMARCC2	1,01
chromosome 15 open reading frame 59	C15orf59	1,01
tensin 3	TNS3	1,01
sodium channel, voltage gated, type IV beta subunit	SCN4B	1,01
upstream transcription factor 1	USF1	1,01
Memczak2013 ANTISENSE, CDS, coding, INTERNAL, UTR3 best transcript NM_001752	CAT	1,01
signal recognition particle 54kDa	SRP54	1,01
F-box protein 43	FBXO43	1,01
forkhead box D4-like 1	FOXD4L1	1,01
Transcript Identified by AceView, Entrez Gene ID(s) 79781	IQCA1	1,01
breast carcinoma amplified sequence 2	BCAS2	1,01
CWC15 spliceosome-associated protein	CWC15	1,01
dolichyl-phosphate (UDP-N-acetylglucosamine) N-acetylglucosaminephosphotransferase 1 (GlcNAc-1-P transferase)	DPAGT1	1,01
zinc finger, imprinted 3	ZIM3	1,01
early growth response 2	EGR2	1,01
amyloid beta (A4) precursor protein-binding, family A, member 1	APBA1	1,01
vacuolar protein sorting 16 homolog (S. cerevisiae)	VPS16	1,01
enhancer of yellow 2 homolog (Drosophila)	ENY2	1,01
v-mos Moloney murine sarcoma viral oncogene homolog	MOS	1,01
excision repair cross-complementation group 5	ERCC5	1,01
SWI/SNF related, matrix associated, actin dependent regulator of chromatin, subfamily a-like 1	SMARCAL1	1,01
zinc finger protein 320	ZNF320	1,01
heme binding protein 2	HEBP2	1,01
carbohydrate (keratan sulfate Gal-6) sulfotransferase 1	CHST1	1,01
BCL2-interacting killer (apoptosis-inducing)	BIK	1,01
zinc finger protein 517	ZNF517	1,01
protein phosphatase 1, regulatory (inhibitor) subunit 14A	PPP1R14A	1,01
mitochondrial calcium uptake family, member 3	MICU3	1,01

MAGE family member B17	MAGEB17	1,01
Jeck2013 ANTISENSE, CDS, coding, INTERNAL, OVCODE, OVEXON best transcript NM_001042476	CARHSP1	1,01
Rho GTPase activating protein 8	ARHGAP8	1,01
AF4/FMR2 family, member 1	AFF1	1,01
UDP glucuronosyltransferase 2 family, polypeptide B4	UGT2B4	1,01
podocalyxin-like 2	PODXL2	1,01
KIAA0586	KIAA0586	1,01
U2 snRNP-associated SURP domain containing	U2SURP	1,01
polyamine-modulated factor 1; PMF1-BGLAP readthrough; bone gamma-carboxyglutamate (gla) protein	PMF1; PMF1-BGLAP; BGLAP	1,01
Wiskott-Aldrich syndrome-like	WASL	1,01
defensin, alpha 3, neutrophil-specific	DEFA3	1,01
calpain 13	CAPN13	1,01
melanoma inhibitory activity 2	MIA2	1,01
archaelysin family metallopeptidase 2	AMZ2	1,01
Rho guanine nucleotide exchange factor 16	ARHGEF16	1,01
myogenic factor 5	MYF5	1,01
septin 7	sept-07	1,01
mediator complex subunit 14	MED14	1,01
eukaryotic translation initiation factor 5A	EIF5A	1,01
pseudouridylate synthase 1	PUS1	1,01
basic leucine zipper and W2 domains 2	BZW2	1,01
adrenoceptor alpha 2B	ADRA2B	1,01
transmembrane protein 119	TMEM119	1,01
IQ motif containing F5	IQCF5	1,01
chloride channel accessory 2	CLCA2	1,01
B-cell CLL/lymphoma 6, member B	BCL6B	1,01
ribosomal protein, large, P1	RPLP1	1,01
ArfGAP with RhoGAP domain, ankyrin repeat and PH domain 2	ARAP2	1,01
autocrine motility factor receptor, E3 ubiquitin protein ligase	AMFR	1,01
mannosidase, endo-alpha-like	MANEAL	1,01
ankyrin repeat and ubiquitin domain containing 1	ANKUB1	1,01
TBC1 domain family, member 8 (with GRAM domain)	TBC1D8	1,01
amylase, alpha 2A (pancreatic)	AMY2A	1,01
Zhang2013 ALT_ACCEPTOR, ALT_DONOR, coding, INTERNAL, intronic best transcript NM_001670	ARVCF	1,01
neuroligin 3	NLGN3	1,01
asialoglycoprotein receptor 2	ASGR2	1,01
RAS-like, family 11, member B	RASL11B	1,01
KIAA1107	KIAA1107	1,01
peroxisomal trans-2-enoyl-CoA reductase	PECR	1,01
guanylyl cyclase domain containing 1	GUCD1	1,01
acylphosphatase 2, muscle type	ACYP2	1,01
sushi domain containing 3	SUSD3	1,01

somatomedin B and thrombospondin type 1 domain containing	SBSPON	1,01
long intergenic non-protein coding RNA 346	LINC00346	1,01
erythrocyte membrane protein band 4.1-like 2	EPB41L2	1,01
olfactory receptor, family 13, subfamily H, member 1	OR13H1	1,01
flotillin 1	FLOT1	1,01
mannosidase, alpha, class 2A, member 2	MAN2A2	1,01
carbonic anhydrase VA, mitochondrial	CA5A	1,01
sialic acid binding Ig-like lectin 14; sialic acid binding Ig-like lectin 5	SIGLEC14; SIGLEC5	1,01
fibronectin type III domain containing 9	FNDC9	1,01
two pore segment channel 1	TPCN1	1,01
Fas associated factor family member 2	FAF2	1,01
G protein-coupled receptor 25	GPR25	1,01
arylsulfatase G	ARSG	1,01
olfactory receptor, family 9, subfamily A, member 4	OR9A4	1,01
phospholipase A2, group IIC	PLA2G2C	1,01
NCBP2 antisense RNA 2 (head to head)	NCBP2-AS2	1,01
extracellular leucine-rich repeat and fibronectin type III domain containing 1	ELFN1	1,01
nuclear receptor subfamily 2, group C, member 1	NR2C1	1,01
partner of NOB1 homolog	PNO1	1,01
TBC1 domain family, member 20	TBC1D20	1,01
zinc finger protein 594	ZNF594	1,01
adhesion molecule with Ig-like domain 3	AMIGO3	1,01
tRNA-yW synthesizing protein 1 homolog B (S. cerevisiae)	TYW1B	1,01
Homo sapiens ribosomal protein S26, mRNA (cDNA clone MGC:104291 IMAGE:4287636), complete cds.	RPS26	1,01
chemokine (C-X-C motif) ligand 10	CXCL10	1,01
transcription elongation factor B polypeptide 3C-like; transcription elongation factor B polypeptide 3C (elongin A3)	TCEB3CL; TCEB3C	1,01
nudix hydrolase 7	NUDT7	1,01
prefoldin subunit 6; microRNA 6834	PFDN6; MIR6834	1,01
golgin B1	GOLGB1	1,01
zinc finger protein 441	ZNF441	1,01
membrane associated ring finger 8	MARCH8	1,01
acyl-CoA dehydrogenase family, member 10	ACAD10	1,01
serine dehydratase-like	SDSL	1,01
alanine and arginine rich domain containing protein	AARD	1,01
MLX interacting protein	MLXIP	1,01
tryptase alpha/beta 1; tryptase beta 2 (gene/pseudogene)	TPSAB1; TPSB2	1,01
doublecortin domain containing 5; doublecortin domain containing 1	DCDC5; DCDC1	1,01
chromosome 12 open reading frame 10	C12orf10	1,01

solute carrier family 5 (sodium/glucose cotransporter), member 2	SLC5A2	1,01
major histocompatibility complex, class II, DM beta	HLA-DMB	1,01
ubiquitin specific peptidase 26	USP26	1,01
proteasome subunit beta 2	PSMB2	1,01
leucine rich repeat containing 9	LRRC9	1,01
eukaryotic translation initiation factor 3, subunit C; eukaryotic translation initiation factor 3, subunit C-like	EIF3C; EIF3CL	1,01
zinc finger protein 417	ZNF417	1,01
nucleoporin 93kDa	NUP93	1,01
HLA complex group 9 (non-protein coding)	HCG9	1,01
helicase, POLQ-like	HELQ	1,01
carcinoembryonic antigen-related cell adhesion molecule 21	CEACAM21	1,01
tripartite motif containing 60	TRIM60	1,01
caspase 2	CASP2	1,01
lysine (K)-specific methyltransferase 5B	KMT5B	1,01
chromosome X open reading frame 49B; chromosome X open reading frame 49	CXorf49B; CXorf49	1,01
olfactory receptor, family 6, subfamily K, member 3	OR6K3	1,01
eukaryotic translation initiation factor 5B	EIF5B	1,01
HIG1 hypoxia inducible domain family, member 1C	HIGD1C	1,01
protocadherin beta 16	PCDHB16	1,01
chromosome 21 open reading frame 33	C21orf33	1,01
BTB (POZ) domain containing 2	BTBD2	1,01
olfactory receptor, family 2, subfamily V, member 1	OR2V1	1,01
acid sensing ion channel family member 4	ASIC4	1,01
Hermansky-Pudlak syndrome 3	HPS3	1,01
nucleoporin 210kDa like; microRNA 5698	NUP210L; MIR5698	1,01
transcription factor EC	TFEC	1,01
F-box and leucine-rich repeat protein 22	FBXL22	1,01
riboflavin kinase	RFK	1,01
surfeit 2 [Source:HGNC Symbol;Acc:HGNC:11475]	SURF2	1,01
glypican 3	GPC3	1,01
leucine rich repeat containing 30	LRRC30	1,01
acyl-CoA thioesterase 7	ACOT7	1,01
Rab geranylgeranyltransferase, alpha subunit	RABGGTA	1,01
farnesyltransferase, CAAX box, alpha	FNTA	1,01
family with sequence similarity 208, member A	FAM208A	1,01
CD2 molecule	CD2	1,01
major histocompatibility complex, class II, DP alpha 1	HLA-DPA1	1,01
trafficking protein, kinesin binding 2	TRAK2	1,01
bolA family member 2; bolA family member 2B	BOLA2; BOLA2B	1,01
RNA guanylyltransferase and 5-phosphatase	RNGTT	1,01

aldehyde dehydrogenase 1 family, member B1	ALDH1B1	1,01
leukocyte immunoglobulin-like receptor, subfamily A (with TM domain), member 1	LILRA1	1,01
endoplasmic reticulum metallopeptidase 1	ERMP1	1,01
eukaryotic translation initiation factor 5	EIF5	1,01
RAD21 cohesin complex component; microRNA 3610	RAD21; MIR3610	1,01
chromosome 2 open reading frame 82	C2orf82	1,01
ubiquitin conjugating enzyme E2Q family member 2-like	UBE2Q2L	1,01
WD repeat domain 17	WDR17	1,01
VPS11, CORVET/HOPS core subunit [Source:HGNC Symbol;Acc:HGNC:14583]	VPS11	1,01
HEAT repeat containing 1	HEATR1	1,01
complement component 1, q subcomponent, A chain	C1QA	1,01
mitochondrial tRNA translation optimization 1	MTO1	1,01
inter-alpha-trypsin inhibitor heavy chain 1	ITIH1	1,01
neuropeptides B/W receptor 1	NPBWR1	1,01
syndecan 1	SDC1	1,01
ribosomal protein L35	RPL35	1,01
transport and golgi organization 2 homolog	TANGO2	1,01
transmembrane protein 63B	TMEM63B	1,01
engulfment and cell motility 2	ELMO2	1,01
4-hydroxyphenylpyruvate dioxygenase-like	HPDL	1,01
chromobox homolog 5; microRNA 3198-2	CBX5; MIR3198-2	1,01
apolipoprotein E	APOE	1,01
ubiquitin C-terminal hydrolase L1	UCHL1	1,01
chromosome 16 open reading frame 87	C16orf87	1,01
chymotrypsin-like elastase family, member 2A	CELA2A	1,01
catenin (cadherin-associated protein), delta 2	CTNND2	1,01
RAB5C, member RAS oncogene family	RAB5C	1,01
transmembrane protein 165	TMEM165	1,01
integrin alpha L	ITGAL	1,01
ribonuclease, RNase A family, 8	RNASE8	1,01
solute carrier family 2 (facilitated glucose transporter), member 4	SLC2A4	1,01
family with sequence similarity 133, member A pseudogene	LOC100421561	1,01
zinc finger protein 257	ZNF257	1,01
transducin-like enhancer of split 2	TLE2	1,01
colipase-like 1	CLPSL1	1,01
adenylate cyclase 5	ADCY5	1,01
Jeck2013 ALT_ACCEPTOR, ALT_DONOR, coding, INTERNAL, intronic best transcript NM_178423	HDAC9	1,01
nucleolar complex associated 4 homolog	NOC4L	1,01
SERTA domain containing 2	SERTAD2	1,01

Rho GTPase activating protein 11B; Rho GTPase activating protein 11A	ARHGAP11B; ARHGAP11A	1,01
somatostatin receptor 4	SSTR4	1,01
ribosomal protein L41 pseudogene 5	RPL41P5	1,01
NME/NM23 nucleoside diphosphate kinase 1; NME1-NME2 readthrough; NME/NM23 nucleoside diphosphate kinase 2	NME1; NME1-NME2; NME2	1,01
centriolar coiled coil protein 110kDa	CCP110	1,01
protein kinase, membrane associated tyrosine/threonine 1	PKMYT1	1,01
tRNA methyltransferase 11-2 homolog (S. cerevisiae)	TRMT112	1,01
microfibrillar associated protein 3	MFAP3	1,01
mitogen-activated protein kinase kinase kinase kinase 1	MAP4K1	1,01
CTD phosphatase subunit 1	CTDP1	1,01
fem-1 homolog a (C. elegans)	FEM1A	1,01
Transcript Identified by AceView, Entrez Gene ID(s) 55701	RP11-998D10.7; FLJ10357	1,01
MARCKS-like 1	MARCKSL1	1,01
alkB homolog 4, lysine demthylase	ALKBH4	1,01
protein tyrosine phosphatase, non-receptor type 13 (APO-1/CD95 (Fas)-associated phosphatase)	PTPN13	1,01
phospholipid phosphatase 5	PLPP5	1,01
Jeck2013 ALT_DONOR, coding, INTERNAL, intronic best transcript NM_016732	RALY	1,01
zinc finger protein 880	ZNF880	1,01
dephospho-CoA kinase domain containing	DCAKD	1,01
hemoglobin, delta	HBD	1,01
PRAME family member 1	PRAMEF1	1,01
septin 3	sept-03	1,01
hippocalcin like 4	HPCAL4	1,01
biliverdin reductase A	BLVRA	1,01
Transcript Identified by AceView, Entrez Gene ID(s) 90338	ZNF160	1,01
keratin 86, type II	KRT86	1,01
sperm acrosome associated 4	SPACA4	1,01
cyclin-dependent kinase 12	CDK12	1,01
FERM domain containing 1	FRMD1	1,01
multivesicular body subunit 12B	MVB12B	1,01
ubiquitin-conjugating enzyme E2M	UBE2M	1,01
importin 11; leucine rich repeat containing 70	IPO11; LRRC70	1,01
kelch domain containing 7B	KLHDC7B	1,01
V-set and transmembrane domain containing 2 like	VSTM2L	1,01
mutL homolog 3	MLH3	1,01
sodium channel, voltage gated, type X alpha subunit	SCN10A	1,01
zinc finger, BED-type containing 6; zinc finger CCCH-type containing 11A	ZBED6; ZC3H11A	1,01
cilia and flagella associated protein 99	CFAP99	1,01

BCL2-antagonist/killer 1	BAK1	1,01
YTH N(6)-methyladenosine RNA binding protein 2	YTHDF2	1,01
ribosomal protein L38	RPL38	1,01
src kinase associated phosphoprotein 1	SKAP1	1,01
kinesin family member 11	KIF11	1,01
HAUS augmin like complex subunit 2	HAUS2	1,01
ephrin-B1	EFNB1	1,01
small EDRK-rich factor 1B (centromeric); small EDRK-rich factor 1A (telomeric)	SERF1B; SERF1A	1,01
small EDRK-rich factor 1A (telomeric)	SERF1A	1,01
renin	REN	1,01
KIAA1919	KIAA1919	1,01
acidic nuclear phosphoprotein 32 family member E	ANP32E	1,01
ribosomal protein L17; small nucleolar RNA, C/D box 58A; small nucleolar RNA, C/D box 58B; small nucleolar RNA, C/D box 58C	RPL17; SNORD58A; SNORD58B; SNORD58C	1,01
heat shock protein 90kDa beta (Grp94), member 1; microRNA 3652	HSP90B1; MIR3652	1,01
zinc finger protein 431; vomeronasal 1 receptor 82 pseudogene	ZNF431; VN1R82P	1,01
GRAM domain containing 4	GRAMD4	1,01
zinc finger protein 425	ZNF425	1,01
TruB pseudouridine (psi) synthase family member 2	TRUB2	1,01
protein phosphatase 6, catalytic subunit	PPP6C	1,01
small nucleolar RNA, C/D box 49A; small nucleolar RNA, C/D box 65; LRRC75A antisense RNA 1	SNORD49A; SNORD65; LRRC75A-AS1	1,01
potassium channel, voltage gated KQT-like subfamily Q, member 5	KCNQ5	1,01
yippee like 3	YPEL3	1,01
keratin 31, type I	KRT31	1,01
centromere protein L	CENPL	1,01
transmembrane protein 14B	TMEM14B	1,01
Ras-related GTP binding C	RRAGC	1,01
mindbomb E3 ubiquitin protein ligase 2	MIB2	1,01
ribosomal protein L8; microRNA 6850	RPL8; MIR6850	1,01
tyrosyl-DNA phosphodiesterase 1	TDP1	1,01
potassium channel tetramerization domain containing 5	KCTD5	1,01
retention in endoplasmic reticulum sorting receptor 1	RER1	1,01
nuclear receptor corepressor 1	NCOR1	1,01
epithelial splicing regulatory protein 2; microRNA 6773	ESRP2; MIR6773	1,01
zinc finger, C4H2 domain containing	ZC4H2	1,01

keratin 15, type I; microRNA 6510	KRT15; MIR6510	1,01
membrane associated ring finger 9	MARCH9	1,01
F-box and leucine-rich repeat protein 15	FBXL15	1,01
cilia and flagella associated protein 77	CFAP77	1,01
protein phosphatase 2, regulatory subunit B, beta	PPP2R3B	1,01
protein phosphatase 2, regulatory subunit B, beta	PPP2R3B	1,01
intestinal cell (MAK-like) kinase	ICK	1,01
eukaryotic translation initiation factor 4B	EIF4B	1,01
lipase maturation factor 2	LMF2	1,01
KIAA1143	KIAA1143	1,01
transportin 1	TNPO1	1,01
actin-like 6A	ACTL6A	1,01
ubiquinol-cytochrome c reductase complex assembly factor 1	UQCC1	1,01
estrogen receptor binding site associated, antigen, 9	EBAG9	1,01
cytochrome P450, family 17, subfamily A, polypeptide 1	CYP17A1	1,01
methyltransferase like 2B	METTL2B	1,01
Memczak2013 ANTISENSE, CDS, coding, INTERNAL, UTR3 best transcript NM_080668	CDCA5	1,01
histidine rich carboxyl terminus 1	HRCT1	1,01
all-trans retinoic acid-induced differentiation factor	ATRAID	1,01
short chain dehydrogenase/reductase family 42E, member 2	SDR42E2	1,01
endonuclease G	ENDOG	1,01
histone acetyltransferase 1	HAT1	1,01
long intergenic non-protein coding RNA 550	LINC00550	1,01
cell death-inducing p53 target 1	CDIP1	1,01
chromosome 17 open reading frame 107	C17orf107	1,01
cytidine monophosphate N-acetylneuraminic acid synthetase	CMAS	1,01
SEC62 homolog, preprotein translocation factor	SEC62	1,01
desumoylating isopeptidase 1	DESI1	1,01
plasmolipin	PLLP	1,01
chitinase, acidic	CHIA	1,01
FtsJ homolog 3 (E. coli)	FTSJ3	1,01
SKI/DACH domain containing 1	SKIDA1	1,01
ST7 antisense RNA 1	ST7-AS1	1,01
Eukaryotic translation initiation factor 3 subunit F [Source:UniProtKB/Swiss-Prot;Acc:O00303]	EIF3F	1,01
ribonuclease/angiogenin inhibitor 1	RNH1	1,01
acyl-CoA synthetase short-chain family member 3	ACSS3	1,01
adenosylhomocysteinase like 1	AHCYL1	1,01
ST6 beta-galactosamide alpha-2,6-sialyltranferase 2	ST6GAL2	1,00
ArfGAP with RhoGAP domain, ankyrin repeat and PH domain 3	ARAP3	1,00
RAB5A, member RAS oncogene family	RAB5A	1,00
thioredoxin domain containing 17	TXNDC17	1,00
CD300 molecule-like family member f	CD300LF	1,00

matrix metallopeptidase 27	MMP27	1,00
Williams Beuren syndrome chromosome region 22	WBSCR22	1,00
UBAP1-MVB12-associated (UMA) domain containing 1	UMAD1	1,00
mitochondrial ribosomal protein S2	MRPS2	1,00
ATPase, Ca++ transporting, cardiac muscle, slow twitch 2	ATP2A2	1,00
serine-rich and transmembrane domain containing 1	SERTM1	1,00
WW domain binding protein 5	WBP5	1,00
phospholipid phosphatase related 4	PLPPR4	1,00
acidic repeat containing	ACRC	1,00
receptor accessory protein 3	REEP3	1,00
chloride channel, voltage-sensitive Ka	CLCNKA	1,00
ATP binding cassette subfamily C member 2	ABCC2	1,00
HLA complex P5 (non-protein coding)	HCP5	1,00
major histocompatibility complex, class II, DQ beta 2	HLA-DQB2	1,00
SIX homeobox 3	SIX3	1,00
RasGEF domain family member 1A	RASGEF1A	1,00
nuclear receptor coactivator 3	NCOA3	1,00
surfeit 4	SURF4	1,00
vaccinia related kinase 3	VRK3	1,00
alkaline ceramidase 1	ACER1	1,00
heterogeneous nuclear ribonucleoprotein F	HNRNPF	1,00
otospiralin	OTOS	1,00
zinc finger and BTB domain containing 49	ZBTB49	1,00
calmodulin 1 (phosphorylase kinase, delta)	CALM1	1,00
protein phosphatase 2, regulatory subunit B, gamma	PPP2R5C	1,00
autophagy related 16-like 1	ATG16L1	1,00
zinc finger protein 197	ZNF197	1,00
propionyl-CoA carboxylase beta subunit	PCCB	1,00
laminin, beta 3; microRNA 4260	LAMB3; MIR4260	1,00
cyclin-dependent kinase 7	CDK7	1,00
LSM12 homolog	LSM12	1,00
dynein assembly factor with WDR repeat domains 1	DAW1	1,00
kinesin family member C1	KIFC1	1,00
contactin 6	CNTN6	1,00
calcium/calmodulin-dependent protein kinase II inhibitor 1	CAMK2N1	1,00
dynamin 1	DNM1	1,00
family with sequence similarity 24, member B	FAM24B	1,00
consortin, connexin sorting protein	CNST	1,00
homocysteine-inducible, endoplasmic reticulum stress-inducible, ubiquitin-like domain member 1	HERPUD1	1,00
zinc finger protein interacting with K protein 1	ZIK1	1,00
family with sequence similarity 72, member C	FAM72C	1,00
synaptosome associated protein 29kDa	SNAP29	1,00
GUF1 homolog, GTPase	GUF1	1,00

ERI1 exoribonuclease family member 3; ERI3 intronic transcript 1	ERI3; ERI3-IT1	1,00
CD3g molecule, gamma (CD3-TCR complex)	CD3G	1,00
hydroxymethylbilane synthase	HMBS	1,00
growth hormone regulated TBC protein 1	GRTP1	1,00
GATS, stromal antigen 3 opposite strand	GATS	1,00
elongator acetyltransferase complex subunit 3	ELP3	1,00
guanosine monophosphate reductase 2	GMPR2	1,00
ectonucleoside triphosphate diphosphohydrolase 7	ENTPD7	1,00
erb-b2 receptor tyrosine kinase 4	ERBB4	1,00
CDC42 effector protein (Rho GTPase binding) 1	CDC42EP1	1,00
chromodomain helicase DNA binding protein 6	CHD6	1,00
MAP/microtubule affinity-regulating kinase 2	MARK2	1,00
methylmalonic aciduria (cobalamin deficiency) cblA type	MMAA	1,00
keratin associated protein 6-2	KRTAP6-2	1,00
proteasome subunit alpha 4	PSMA4	1,00
ELM2 and Myb/SANT-like domain containing 1	ELMSAN1	1,00
late endosomal/lysosomal adaptor, MAPK and MTOR activator 2	LAMTOR2	1,00
homeobox B2	HOXB2	1,00
thrombospondin type 1 domain containing 7A	THSD7A	1,00
actin binding LIM protein 1	ABLIM1	1,00
orosomucoid 2	ORM2	1,00
pleckstrin homology domain containing, family A member 7	PLEKHA7	1,00
dehydrogenase/reductase (SDR family) member 11	DHRS11	1,00
VGF nerve growth factor inducible	VGF	1,00
kelch domain containing 3	KLHDC3	1,00
N-ethylmaleimide-sensitive factor attachment protein, gamma	NAPG	1,00
pleckstrin homology domain containing, family G (with RhoGef domain) member 7	PLEKHG7	1,00
TMED7-TICAM2 readthrough; toll-like receptor adaptor molecule 2; transmembrane p24 trafficking protein 7	TMED7-TICAM2; TICAM2; TMED7	1,00
chronic lymphocytic leukemia up-regulated 1	CLLU1	1,00
UDP-glucose glycoprotein glucosyltransferase 1	UGGT1	1,00
major histocompatibility complex, class I, F	HLA-F	1,00
fatty acid desaturase 6	FADS6	1,00
tight junction protein 3	TJP3	1,00
isoleucyl-tRNA synthetase 2, mitochondrial	IARS2	1,00
coiled-coil serine rich protein 2	CCSER2	1,00
F-box protein 4	FBXO4	1,00
cholesterol 25-hydroxylase	CH25H	1,00
taste receptor, type 2, member 42	TAS2R42	1,00
polo-like kinase 3	PLK3	1,00

contactin associated protein-like 4	CNTNAP4	1,00
FAST kinase domains 1	FASTKD1	1,00
golgi SNAP receptor complex member 1	GOSR1	1,00
VPS11, CORVET/HOPS core subunit [Source:HGNC Symbol;Acc:HGNC:14583]	VPS11	1,00
LIM domain and actin binding 1	LIMA1	1,00
RPS10-NUDT3 readthrough	RPS10-NUDT3	1,00
Sec61 translocon alpha 2 subunit	SEC61A2	1,00
solute carrier family 2 (facilitated glucose transporter), member 6	SLC2A6	1,00
hydroxyacyl-CoA dehydrogenase/3-ketoacyl-CoA thiolase/enoyl-CoA hydratase (trifunctional protein), beta subunit	HADHB	1,00
hypoxia up-regulated 1	HYOU1	1,00
aristaless related homeobox	ARX	1,00
lysophosphatidic acid receptor 1	LPAR1	1,00
solute carrier family 37 (glucose-6-phosphate transporter), member 4 [Source:HGNC Symbol;Acc:HGNC:4061]	SLC37A4	1,00
ARP3 actin-related protein 3 homolog C (yeast)	ACTR3C	1,00
zinc finger protein 599	ZNF599	1,00
FGR proto-oncogene, Src family tyrosine kinase	FGR	1,00
uridine-cytidine kinase 2; microRNA 3658	UCK2; MIR3658	1,00
Sp9 transcription factor	SP9	1,00
long intergenic non-protein coding RNA 273	LINC00273	1,00
TNFAIP3 interacting protein 1	TNIP1	1,00
cyclin-dependent kinase inhibitor 1A (p21, Cip1)	CDKN1A	1,00
microsomal glutathione S-transferase 3	MGST3	1,00
spectrin, beta, non-erythrocytic 4	SPTBN4	1,00
FGFR1 oncogene partner 2	FGFR1OP2	1,00
growth regulation by estrogen in breast cancer-like	GREB1L	1,00
mannosidase, alpha, class 2B, member 1	MAN2B1	1,00
SH3 and cysteine rich domain 3	STAC3	1,00
RAB13, member RAS oncogene family	RAB13	1,00
NDUFC2-KCTD14 readthrough; NADH dehydrogenase (ubiquinone) 1, subcomplex unknown, 2, 14.5kDa; potassium channel tetramerization domain containing 14	NDUFC2-KCTD14; NDUFC2; KCTD14	1,00
Rho-associated, coiled-coil containing protein kinase 1	ROCK1	1,00
suppression of tumorigenicity 13 (colon carcinoma) (Hsp70 interacting protein)	ST13	1,00
exonuclease 1	EXO1	1,00
serine incorporator 3	SERINC3	1,00
zinc finger protein 197	ZNF197	1,00
trophinin associated protein	TROAP	1,00
tumor necrosis factor, alpha-induced protein 2	TNFAIP2	1,00

tryptase gamma 1	TPSG1	1,00
basic leucine zipper and W2 domains 1	BZW1	1,00
ST3 beta-galactoside alpha-2,3-sialyltransferase 6	ST3GAL6	1,00
transmembrane protein 247	TMEM247	1,00
protoporphyrinogen oxidase	PPOX	1,00
transducer of ERBB2, 2	TOB2	1,00
ring finger protein 103	RNF103	1,00
vacuolar protein sorting 53 homolog (S. cerevisiae); ribosomal protein S4X pseudogene 17	VPS53; RPS4XP17	1,00
G protein-coupled receptor associated sorting protein 2	GPRASP2	1,00
Memczak2013 ANTISENSE, coding, INTERNAL, UTR5 best transcript NM_005583	LYL1	1,00
translocation associated membrane protein 1-like 1	TRAM1L1	1,00
synovial sarcoma, X breakpoint 2 interacting protein	SSX2IP	1,00
transcription factor A, mitochondrial	TFAM	1,00
STAM binding protein	STAMBP	1,00
glutamyl-tRNA(Gln) amidotransferase, subunit C	GATC	1,00
Rho GTPase activating protein 12	ARHGAP12	1,00
potassium channel, voltage gated subfamily E regulatory beta subunit 3	KCNE3	1,00
rhomboid domain containing 3	RHBDD3	1,00
neuropeptide S	NPS	1,00
Bloom syndrome, RecQ helicase-like	BLM	1,00
Rho-related BTB domain containing 2	RHOBTB2	1,00
phosphatidylinositol glycan anchor biosynthesis class M	PIGM	1,00
transcription factor 12	TCF12	1,00
ectonucleotide pyrophosphatase/phosphodiesterase 4 (putative)	ENPP4	1,00
eukaryotic translation elongation factor 1 delta (guanine nucleotide exchange protein)	EEF1D	1,00
zinc finger protein 578	ZNF578	1,00
ankyrin repeat domain 34B	ANKRD34B	1,00
dynein, axonemal, assembly factor 5	DNAAF5	1,00
argonaute RISC catalytic component 4	ago-04	1,00
hypoxia up-regulated 1	HYOU1	1,00
IQ motif containing B1	IQCB1	1,00
diazepam binding inhibitor (GABA receptor modulator, acyl-CoA binding protein)	DBI	1,00
protein tyrosine phosphatase, non-receptor type 11	PTPN11	1,00
kielin/chordin-like protein	KCP	1,00
chromosome 11 open reading frame 91	C11orf91	1,00
olfactory receptor, family 2, subfamily T, member 10	OR2T10	1,00
solute carrier family 15 (oligopeptide transporter), member 1	SLC15A1	1,00
small EDRK-rich factor 2	SERF2	1,00
G1 to S phase transition 2	GSPT2	1,00
progesterone receptor membrane component 2	PGRMC2	1,00

malignant fibrous histiocytoma amplified sequence 1	MFHAS1	1,00
family with sequence similarity 84, member A	FAM84A	1,00
CDP-diacylglycerol--inositol 3-phosphatidyltransferase	CDIPT	1,00
neuroblastoma breakpoint family, member 26	NBPF26	1,00
cysteine-rich PAK1 inhibitor	CRIPAK	1,00
necdin, MAGE family member	NDN	1,00
PWP2 periodic tryptophan protein homolog (yeast)	PWP2	1,00
surfactant protein C	SFTPC	1,00
Transcript Identified by AceView, Entrez Gene ID(s) 79572	ATP13A3	1,00
trafficking protein particle complex 4; microRNA 3656	TRAPPC4; MIR3656	1,00
pentraxin 3, long	PTX3	1,00
GDP dissociation inhibitor 2	GDI2	1,00
golgin A6 family, member D	GOLGA6D	1,00
surfeit 4	SURF4	1,00
solute carrier family 7 (amino acid transporter light chain, y+L system), member 6	SLC7A6	1,00
KIAA1217	KIAA1217	1,00
coiled-coil domain containing 3	CCDC3	1,00
zinc finger protein 304	ZNF304	1,00
galactose-3-O-sulfotransferase 3	GAL3ST3	1,00
Synthetic construct Homo sapiens clone IMAGE:100063377, MGC:190767 ribosomal protein L41 (RPL41) mRNA, encodes complete protein.	RPL41	1,00
sperm associated antigen 11B	SPAG11B	1,00
nitrilase family, member 2	NIT2	1,00
ZFP90 zinc finger protein	ZFP90	1,00
pyridoxal (pyridoxine, vitamin B6) kinase	PDXK	1,00
transmembrane protease, serine 5	TMPRSS5	1,00
coenzyme Q9	COQ9	1,00
SAP30-like	SAP30L	1,00
transmembrane protein 61	TMEM61	1,00
THAP domain containing 8	THAP8	1,00
family with sequence similarity 72, member A	FAM72A	1,00
cerebellin 1 precursor	CBLN1	1,00
Sad1 and UNC84 domain containing 1	SUN1	1,00
dynein regulatory complex subunit 1	DRC1	1,00
purinergic receptor P2Y, G-protein coupled, 14	P2RY14	1,00
carbohydrate (chondroitin 4) sulfotransferase 11	CHST11	1,00
TGF-beta activated kinase 1/MAP3K7 binding protein 3	TAB3	1,00
family with sequence similarity 101, member B	FAM101B	1,00
LYR motif containing 1	LYRM1	1,00
increased sodium tolerance 1 homolog (yeast)	IST1	1,00
zinc finger and BTB domain containing 47	ZBTB47	1,00
kin of IRRE like 2 (Drosophila)	KIRREL2	1,00

Yes-associated protein 1	YAP1	1,00
ArfGAP with FG repeats 1; microRNA 5703	AGFG1; MIR5703	1,00
interleukin 1 receptor associated kinase 2	IRAK2	1,00
family with sequence similarity 71, member A	FAM71A	1,00
xeroderma pigmentosum, complementation group C	XPC	1,00
zinc finger protein 320	ZNF320	1,00
solute carrier family 25, member 46	SLC25A46	1,00
neuronatin	NNAT	1,00
CDC42 small effector 2	CDC42SE2	1,00
major facilitator superfamily domain containing 14A	MFSD14A	1,00
timeless circadian clock	TIMELESS	1,00
eukaryotic translation initiation factor 2 alpha kinase 4	EIF2AK4	1,00
RAP2C, member of RAS oncogene family	RAP2C	1,00
transmembrane protein 52	TMEM52	1,00
acyl-CoA wax alcohol acyltransferase 1	AWAT1	1,00
SprT-like N-terminal domain	SPRTN	1,00
motile sperm domain containing 2	MOSPD2	1,00
SDA1 domain containing 1	SDAD1	1,00
ets variant 5	ETV5	1,00
small integral membrane protein 20	SMIM20	1,00
ADAM metallopeptidase with thrombospondin type 1 motif 5	ADAMTS5	1,00
coagulation factor II (thrombin) receptor	F2R	1,00
homeobox C13	HOXC13	1,00
tubulin, gamma 2	TUBG2	1,00
RNA 2,3-cyclic phosphate and 5-OH ligase	RTCB	1,00
histocompatibility (minor) 13; malignant T-cell amplified sequence 2, pseudogene	HM13; MCTS2P	1,00
proenkephalin	PENK	1,00
BTB (POZ) domain containing 19	BTBD19	1,00
tubulin, beta 4B class IVb	TUBB4B	1,00
nephronophthisis 4	NPHP4	1,00
fatty acyl-CoA reductase 2	FAR2	1,00
PRR5-ARHGAP8 readthrough	PRR5-ARHGAP8	1,00
ret finger protein-like 2	RFPL2	1,00
crumbs family member 1, photoreceptor morphogenesis associated	CRB1	1,00
kinesin heavy chain member 2A	KIF2A	1,00
solute carrier family 35, member G6	SLC35G6	1,00
5,10-methenyltetrahydrofolate synthetase (5-formyltetrahydrofolate cyclo-ligase)	MTHFS	1,00
multivesicular body subunit 12A; BST2 interferon stimulated positive regulator (non-protein coding)	MVB12A; BISPR	1,00
WD repeat domain 1	WDR1	1,00

zinc finger and BTB domain containing 34	ZBTB34	1,00
SOGA family member 3; KIAA0408	SOGA3; KIAA0408	1,00
F-box protein 40	FBXO40	1,00
CTAGE family, member 9	CTAGE9	1,00
MT-RNR2-like 4	MTRNR2L4	1,00
IL2-inducible T-cell kinase	ITK	1,00
zinc finger protein 48	ZNF48	1,00
jagunal homolog 1	JAGN1	1,00
transcription elongation factor B (SIII), polypeptide 1 (15kDa, elongin C)	TCEB1	1,00
SLIT-ROBO Rho GTPase activating protein 2B	SRGAP2B	1,00
xin actin binding repeat containing 2	XIRP2	1,00
ribosomal protein L6	RPL6	1,00
neurotrophin 4	NTF4	1,00
rhodopsin	RHO	1,00
histone cluster 1, H2bd	HIST1H2BD	1,00
polypeptide N-acetylgalactosaminyltransferase 15	GALNT15	1,00
IQ motif containing E	IQCE	1,00
patatin-like phospholipase domain containing 7	PNPLA7	1,00
L-3-hydroxyproline dehydratase (trans-)	L3HYPDH	1,00
trafficking protein particle complex 4	TRAPPC4	1,00
von Willebrand factor C domain containing protein 2-like	VWC2L	1,00
S100 calcium binding protein A3	S100A3	1,00
ZNF32 antisense RNA 3	ZNF32-AS3	1,00
GATA binding protein 4	GATA4	1,00
Jeck2013 ALT_ACCEPTOR, ALT_DONOR, coding, INTERNAL, intronic best transcript NM_002737	PRKCA	1,00
solute carrier family 25 (mitochondrial carrier; adenine nucleotide translocator), member 6	SLC25A6	1,00
myozenin 1	MYOZ1	1,00
DDB1 and CUL4 associated factor 12-like 2	DCAF12L2	1,00
speedy/RINGO cell cycle regulator family member A	SPDYA	1,00
TSEN34 tRNA splicing endonuclease subunit	TSEN34	1,00
small G protein signaling modulator 3	SGSM3	1,00
histone deacetylase 1	HDAC1	1,00
SEC14 and spectrin domains 1	SESTD1	1,00
SH3 domain containing ring finger 1	SH3RF1	1,00
interferon, alpha-inducible protein 27-like 2	IFI27L2	1,00
zinc finger protein 273	ZNF273	1,00
5, 3-nucleotidase, cytosolic	NT5C	1,00
cyclin B2	CCNB2	1,00
nudE neurodevelopment protein 1-like 1	NDEL1	1,00
glutamate receptor, metabotropic 6	GRM6	1,00
RAS-like, family 12	RASL12	1,00

ubiquitin protein ligase E3A	UBE3A	1,00
polymerase (DNA directed), delta 2, accessory subunit	POLD2	1,00
dynein, light chain, LC8-type 2	DYNLL2	1,00
polypeptide N-acetylgalactosaminyltransferase 14	GALNT14	1,00
divergent-paired related homeobox	DPRX	1,00
lens epithelial protein	LENEP	1,00
gamma-aminobutyric acid (GABA) A receptor, rho 2	GABRR2	1,00
RAD54-like (S. cerevisiae)	RAD54L	1,00
solute carrier family 1 (neuronal/epithelial high affinity glutamate transporter, system Xag), member 1	SLC1A1	1,00
ATPase, Ca++ transporting, plasma membrane 3	ATP2B3	1,00
pantothenate kinase 3	PANK3	1,00
MAGE family member A2; MAGE family member A2B	MAGEA2; MAGEA2B	1,00
low density lipoprotein receptor class A domain containing 1	LDLRAD1	1,00
aquaporin 4	AQP4	1,00
notochord homeobox	NOTO	1,00
calcium channel, voltage-dependent, alpha 2/delta subunit 1	CACNA2D1	1,00
vacuolar protein sorting 72 homolog (S. cerevisiae)	VPS72	1,00
gap junction protein alpha 5	GJA5	1,00
5-hydroxytryptamine (serotonin) receptor 2B, G protein-coupled	HTR2B	1,00
TOX high mobility group box family member 3	TOX3	1,00
NAD kinase	NADK	1,00
fucose mutarotase	FUOM	1,00
glutathione S-transferase pi 1	GSTP1	1,00
AT-hook transcription factor	AKNA	1,00
defensin, beta 129	DEFB129	1,00
transmembrane protein 132B	TMEM132B	1,00
mitogen-activated protein kinase 3	MAPK3	1,00
variable charge, X-linked 3B; variable charge, X-linked	VCX3B; VCX	1,00
inositol polyphosphate-5-phosphatase D	INPP5D	1,00
radical S-adenosyl methionine domain containing 2	RSAD2	1,00
methionine adenosyltransferase II, beta	MAT2B	1,00
metastasis associated in colon cancer 1	MACC1	1,00
MOB kinase activator 3B	MOB3B	1,00
serine/threonine kinase receptor associated protein	STRAP	1,00
killer cell lectin-like receptor subfamily A pseudogene 1	KLRAP1	1,00
adrenoceptor alpha 1D	ADRA1D	1,00
mixed lineage kinase domain-like	MLKL	1,00
melanocortin 3 receptor	MC3R	1,00
olfactory receptor, family 56, subfamily A, member 1	OR56A1	1,00
	LRIG1	1,00
proteasome inhibitor subunit 1	PSMF1	1,00
olfactory receptor, family 52, subfamily E, member 6	OR52E6	1,00
RIMS binding protein 3	RIMBP3	1,00

ubiquitin-fold modifier conjugating enzyme 1	UFC1	1,00
RNA pseudouridylate synthase domain containing 3	RPUSD3	1,00
peptidyl-prolyl cis-trans isomerase A-like 4G; Peptidyl-prolyl cis-trans isomerase [Source:UniProtKB/TrEMBL;Acc:A0A075B767]	LOC105371242; AC243756.1	1,00
cilia and flagella associated protein 157	CFAP157	1,00
sema domain, transmembrane domain (TM), and cytoplasmic domain, (semaphorin) 6A	SEMA6A	1,00
golgi-associated, gamma adaptin ear containing, ARF binding protein 3	GGA3	1,00
S-antigen; retina and pineal gland (arrestin)	SAG	1,00
Ras interacting protein 1	RASIP1	1,00
RNA binding motif protein, Y-linked, family 1, member F; RNA binding motif protein, Y-linked, family 2, member F pseudogene; RNA binding motif protein, Y-linked, family 1, member J	RBMY1F; RBMY2FP; RBMY1J	1,00
HECT domain containing E3 ubiquitin protein ligase 1	HECTD1	1,00
troponin T type 2 (cardiac)	TNNT2	1,00
solute carrier family 12 (sodium/chloride transporter), member 3	SLC12A3	1,00
necdin-like 2, SMC5-SMC6 complex component	NDNL2	1,00
keratin associated protein 10-2	KRTAP10-2	1,00
BCL2-like 14 (apoptosis facilitator)	BCL2L14	1,00
novel transcript; Transcript Identified by AceView, Entrez Gene ID(s) 7099	RP11-281A20.2; TLR4	1,00
zinc finger, MYM-type 4	ZMYM4	1,00
proteasome 26S subunit, non-ATPase 5	PSMD5	1,00
calcium and integrin binding 1 (calmyrin)	CIB1	1,00
coiled-coil domain containing 137	CCDC137	1,00
translocated promoter region, nuclear basket protein	TPR	1,00
Sin3A associated protein 18kDa	SAP18	1,00
Homo sapiens MGC57346-CRHR1 readthrough (MGC57346-CRHR1), transcript variant 5, mRNA.; Homo sapiens MGC57346-CRHR1 readthrough (MGC57346-CRHR1), transcript variant 6, mRNA.; novel transcript	MGC57346-CRHR1; RP11-105N13.4	1,00
BRCA1/BRCA2-containing complex subunit 3	BRCC3	1,00
HAND2 antisense RNA 1 (head to head)	HAND2-AS1	1,00
coronin, actin binding protein, 2B	CORO2B	1,00
toll-like receptor 6	TLR6	1,00
DEAD (Asp-Glu-Ala-Asp) box polypeptide 41	DDX41	1,00
NADH dehydrogenase (ubiquinone) 1 alpha subcomplex, 10, 42kDa	NDUFA10	1,00
histone cluster 1, H2bf	HIST1H2BF	1,00
ubiquinol-cytochrome c reductase core protein I	UQCRC1	1,00
tumor protein, translationally-controlled 1; small nucleolar RNA, H/ACA box 31	TPT1; SNORA31	1,00

leucine rich repeat containing 25	LRRC25	1,00
pleckstrin homology domain containing, family A member 5	PLEKHA5	1,00
argonaute RISC catalytic component 3	ago-03	1,00
olfactory receptor, family 10, subfamily A, member 3	OR10A3	1,00
serine incorporator 1	SERINC1	1,00
ribosomal protein S15a	RPS15A	1,00
apoptosis resistant E3 ubiquitin protein ligase 1	AREL1	1,00
pygopus family PHD finger 1	PYGO1	1,00
POM121 transmembrane nucleoporin-like 12	POM121L12	1,00
regulator of G-protein signaling 14	RGS14	1,00
transmembrane 7 superfamily member 2	TM7SF2	1,00
olfactory receptor, family 51, subfamily V, member 1	OR51V1	1,00
death inducer-obliterator 1	DIDO1	1,00
cyclin-dependent kinase inhibitor 2A	CDKN2A	1,00
GDNF family receptor alpha 2	GFRA2	1,00
RAS (RAD and GEM)-like GTP-binding 1	REM1	1,00
ubiquitin specific peptidase 12	USP12	1,00
zinc finger protein 837	ZNF837	1,00
calcium channel, voltage-dependent, T type, alpha 1H subunit	CACNA1H	1,00
chromodomain helicase DNA binding protein 5	CHD5	1,00
jagged 1	JAG1	1,00
DnaJ (Hsp40) homolog, subfamily B, member 8	DNAJB8	1,00
neuritin 1-like	NRN1L	1,00
forkhead box N4	FOXN4	1,00
zyg-11 family member A, cell cycle regulator	ZYG11A	1,00
reticulon 3	RTN3	1,00
ribosomal protein L23a; small nucleolar RNA, C/D box 4B; small nucleolar RNA, C/D box 42B; small nucleolar RNA, C/D box 42A	RPL23A; SNORD4B; SNORD42B; SNORD42A	1,00
uncharacterized LOC149950; novel transcript	LOC149950; RP11-410N8.4	1,00
nephrosis 2, idiopathic, steroid-resistant (podocin)	NPHS2	1,00
leucine rich repeat containing 56	LRRC56	1,00
signal sequence receptor, delta	SSR4	1,00
serine peptidase inhibitor, Kazal type 9	SPINK9	1,00
homeodomain interacting protein kinase 1	HIPK1	1,00
thioredoxin reductase 3	TXNRD3	1,00
family with sequence similarity 207, member A	FAM207A	1,00
BR serine/threonine kinase 2	BRSK2	1,00
5-hydroxytryptamine (serotonin) receptor 3E, ionotropic	HTR3E	1,00
bone morphogenetic protein 2	BMP2	1,00
complement component 4B (Chido blood group)	C4B	1,00
complement component 4A (Rodgers blood group)	C4A	1,00
vestigial-like family member 1	VGLL1	1,00

uncharacterized protein LOC158434 [Source:RefSeq peptide;Acc:NP_001243337]; uncharacterized LOC158434; Protein LOC158434 [Source:UniProtKB/TrEMBL;Acc:A0A087X204]; novel transcript; Transcript Identified by AceView	RP11-569G13.3; LOC158434; setora	1,00
KIAA0226-like	KIAA0226L	1,00
Ras association (RalGDS/AF-6) domain family member 4	RASSF4	1,00
inter-alpha-trypsin inhibitor heavy chain 2	ITIH2	1,00
zinc finger and BTB domain containing 22	ZBTB22	1,00
septin 10	sept-10	1,00
ankyrin repeat and MYND domain containing 2	ANKMY2	1,00
LIM homeobox 5	LHX5	1,00
zinc finger protein 555	ZNF555	1,00
myristoylated alanine-rich protein kinase C substrate	MARCKS	1,00
carbohydrate (chondroitin 4) sulfotransferase 12	CHST12	1,00
basonuclin 1	BNC1	1,00
defensin, beta 119	DEFB119	1,00
nucleolar protein 3 (apoptosis repressor with CARD domain)	NOL3	1,00
cholinergic receptor, nicotinic epsilon	CHRNE	1,00
Kruppel-like factor 12	KLF12	1,00
SHOC2 leucine-rich repeat scaffold protein	SHOC2	1,00
prostate stem cell antigen	PSCA	1,00
synovial apoptosis inhibitor 1, synoviolin	SYVN1	1,00
MAGE family member A11	MAGEA11	1,00
chromosome 11 open reading frame 74	C11orf74	1,00
glutaminyl-peptide cyclotransferase-like	QPCTL	1,00
lactamase, beta	LACTB	1,00
C-type lectin domain family 14, member A	CLEC14A	1,00
zinc finger protein 37A	ZNF37A	1,00
ovo-like zinc finger 1	OVOL1	1,00
S-antigen; retina and pineal gland (arrestin)	SAG	1,00
cytochrome c oxidase assembly factor 5	COA5	1,00
far upstream element (FUSE) binding protein 1	FUBP1	1,00
myeloid/lymphoid or mixed-lineage leukemia; translocated to, 11	MLLT11	1,00
Jeck2013 ALT_ACCEPTOR, ALT_DONOR, coding, INTERNAL, intronic best transcript NM_004307	APBB2	1,00
DnaJ (Hsp40) homolog, subfamily C , member 25	DNAJC25	1,00
transmembrane protein 174	TMEM174	1,00
transferrin	TF	1,00
aldehyde dehydrogenase 6 family, member A1	ALDH6A1	1,00
protein tyrosine phosphatase, receptor type, Q	PTPRQ	1,00
TSPY-like 1	TSPYL1	1,00

t-complex 1; small nucleolar RNA, H/ACA box 20; small nucleolar RNA, H/ACA box 29	TCP1; SNORA20; SNORA29	1,00
POM121 transmembrane nucleoporin C	POM121C	1,00
ret finger protein-like 4B	RFPL4B	1,00
phospholipase D family, member 3	PLD3	1,00
glutamic-oxaloacetic transaminase 1, soluble	GOT1	1,00
family with sequence similarity 46, member A	FAM46A	1,00
transmembrane protein 144	TMEM144	1,00
chromosome 3 open reading frame 84	C3orf84	1,00
Niemann-Pick disease, type C2; microRNA 4709	NPC2; MIR4709	1,00
transient receptor potential cation channel, subfamily C, member 7	TRPC7	1,00
LSM5 homolog, U6 small nuclear RNA and mRNA degradation associated	LSM5	1,00
chemokine (C-X-C motif) ligand 17	CXCL17	1,00
putative UPF0607 protein ENSP00000383144	LOC100129307	1,00
uncharacterized protein LOC100652758 [Source:RefSeq peptide;Acc:NP_001265011]; uncharacterized LOC100652758; novel transcript	RP11-54C4.3; LOC100652758	1,00
killer cell lectin-like receptor subfamily C, member 1	KLRC1	1,00
kinesin family member 22	KIF22	1,00
forkhead box D2	FOXD2	1,00
actin-like 8	ACTL8	1,00
UDP glucuronosyltransferase 2 family, polypeptide B10	UGT2B10	1,00
hematopoietic cell signal transducer	HCST	1,00
CCZ1 homolog B, vacuolar protein trafficking and biogenesis associated	CCZ1B	1,00
homogentisate 1,2-dioxygenase	HGD	1,00
coiled-coil domain containing 103	CCDC103	1,00
zinc finger, X-linked, duplicated A	ZXDA	1,00
target of myb1 like 2 membrane trafficking protein	TOM1L2	1,00
mast cell immunoglobulin-like receptor 1	MILR1	1,00
inverted formin, FH2 and WH2 domain containing	INF2	1,00
cytochrome P450, family 1, subfamily A, polypeptide 1	CYP1A1	1,00
SEC24 homolog B, COPII coat complex component	SEC24B	1,00
asialoglycoprotein receptor 1	ASGR1	1,00
immunoglobulin superfamily, member 5	IGSF5	1,00
histamine N-methyltransferase	HNMT	1,00
family with sequence similarity 199, X-linked	FAM199X	1,00
CCR4-NOT transcription complex subunit 4	CNOT4	1,00
oxysterol binding protein-like 7	OSBPL7	1,00
glycoprotein M6A	GPM6A	1,00
replication termination factor 2 domain containing 1	RTFDC1	1,00

mitotic spindle organizing protein 2A; microRNA 4784	MZT2A; MIR4784	1,00
polyamine oxidase (exo-N4-amino)	PAOX	1,00
receptor accessory protein 4	REEP4	1,00
chromosome 15 open reading frame 32	C15orf32	1,00
ribosomal protein L35a	RPL35A	1,00
B-cell CLL/lymphoma 6	BCL6	1,00
signal-induced proliferation-associated 1 like 1	SIPA1L1	1,00
T-box 1	TBX1	1,00
solute carrier family 35 (GDP-fucose transporter), member C2	SLC35C2	1,00
IGF like family receptor 1	IGFLR1	1,00
male germ cell-associated kinase	MAK	1,00
NADH dehydrogenase (ubiquinone) 1 alpha subcomplex, 10, 42kDa	NDUFA10	1,00
steroid receptor RNA activator 1	SRA1	1,00
hypoxia up-regulated 1	HYOU1	1,00
inducible T-cell co-stimulator	ICOS	1,00
GPR75-ASB3 readthrough	GPR75-ASB3	1,00
topoisomerase (DNA) II binding protein 1	TOPBP1	1,00
GTP binding protein 3 (mitochondrial)	GTPBP3	1,00
podocalyxin-like	PODXL	1,00
guanylate cyclase 2D, membrane (retina-specific)	GUCY2D	1,00
dynactin 5 (p25)	DCTN5	1,00
neuralized E3 ubiquitin protein ligase 3	NEURL3	1,00
ATM serine/threonine kinase	ATM	1,00
growth hormone secretagogue receptor	GHSR	1,00
sulfatase modifying factor 1	SUMF1	1,00
ATP synthase, H+ transporting, mitochondrial Fo complex subunit C3 (subunit 9)	ATP5G3	1,00
carbonic anhydrase VII	CA7	1,00
pyrophosphatase (inorganic) 1	PPA1	1,00
additional sex combs like transcriptional regulator 1	ASXL1	1,00
PRP40 pre-mRNA processing factor 40 homolog A	PRPF40A	1,00
secernin 1	SCRN1	1,00
nuclear factor of activated T-cells, cytoplasmic, calcineurin-dependent 1	NFATC1	1,00
proteasome (prosome, macropain) assembly chaperone 1	PSMG1	1,00
methyltransferase like 9	METTL9	1,00
regulator of G-protein signaling 3	RGS3	1,00
dual specificity phosphatase 14	DUSP14	1,00
olfactory receptor, family 6, subfamily C, member 75	OR6C75	1,00
putative homeodomain transcription factor 2	PHTF2	1,00
piezo-type mechanosensitive ion channel component 1	PIEZO1	1,00
major facilitator superfamily domain containing 9	MFSD9	1,00
ATP binding cassette subfamily A member 4	ABCA4	1,00

eukaryotic translation initiation factor 3, subunit G	EIF3G	1,00
sperm mitochondria-associated cysteine-rich protein	SMCP	1,00
trophoblast glycoprotein	TPBG	1,00
importin 8	IPO8	1,00
thyroid hormone receptor, alpha	THRA	1,00
prenylcysteine oxidase 1	PCYOX1	1,00
repulsive guidance molecule family member a	RGMA	1,00
adenylosuccinate synthase	ADSS	1,00
centromere protein V	CENPV	1,00
ankyrin 1, erythrocytic	ANK1	1,00
adenosine deaminase domain containing 2	ADAD2	1,00
transmembrane protein 150C	TMEM150C	1,00
potassium channel, voltage gated modifier subfamily G, member 1	KCNG1	1,00
GDP-mannose pyrophosphorylase A	GMPPA	1,00
family with sequence similarity 63, member B	FAM63B	1,00
olfactory receptor, family 11, subfamily H, member 1	OR11H1	1,00
ring finger protein 212	RNF212	1,00
valosin containing protein lysine (K) methyltransferase	VCPKMT	1,00
ATPase, H+ transporting, lysosomal 9kDa, V0 subunit e1	ATP6V0E1	1,00
dual specificity phosphatase 13	DUSP13	1,00
gamma-glutamyltransferase 6	GGT6	1,00
prohibitin	PHB	1,00
solute carrier family 43 (amino acid system L transporter), member 2	SLC43A2	1,00
prolyl endopeptidase	PREP	1,00
dynamin 3	DNM3	1,00
olfactory receptor, family 8, subfamily D, member 2 (gene/pseudogene)	OR8D2	1,00
FK506 binding protein 15	FKBP15	1,00
histone cluster 1, H4d	HIST1H4D	1,00
RFX family member 8, lacking RFX DNA binding domain	RFX8	1,00
occludin	OCLN	1,00
myosin, heavy chain 9, non-muscle	MYH9	1,00
cell division cycle 6	CDC6	1,00
golgi to ER traffic protein 4	GET4	1,00
dicer 1, ribonuclease type III	DICER1	1,00
metallothionein 1L (gene/pseudogene)	MT1L	1,00
phosphodiesterase 6D, cGMP-specific, rod, delta	PDE6D	1,00
CUGBP, Elav-like family member 4	CELF4	1,00
hypoxia up-regulated 1	HYOU1	1,00
zinc finger protein 273	ZNF273	1,00
cancer/testis antigen 2	CTAG2	1,00
F-box protein 5	FBXO5	1,00
RFNG O-fucosylpeptide 3-beta-N-acetylglucosaminyltransferase	RFNG	1,00

zinc finger protein 534	ZNF534	1,00
BARX homeobox 1	BARX1	1,00
glutaredoxin	GLRX	1,00
trans-golgi network vesicle protein 23 homolog B (S. cerevisiae)	TVP23B	1,00
ciliary neurotrophic factor	CNTF	1,00
ribosomal protein S6 kinase, 90kDa, polypeptide 1	RPS6KA1	1,00
tubulin, alpha 1a	TUBA1A	1,00
chromosome 19 open reading frame 66	C19orf66	1,00
Zic family member 2	ZIC2	1,00
parathymosin	PTMS	1,00
SNU13 homolog, small nuclear ribonucleoprotein (U4/U6.U5)	SNU13	1,00
oxysterol binding protein-like 2	OSBPL2	1,00
polypeptide N-acetylgalactosaminyltransferase 5	GALNT5	1,00
acetoacetyl-CoA synthetase	AACS	1,00
advanced glycosylation end product-specific receptor	AGER	1,00
Rho GTPase activating protein 40	ARHGAP40	1,00
protein kinase, cAMP-dependent, catalytic, beta	PRKACB	1,00
chromosome 2 open reading frame 27A	C2orf27A	1,00
HECT and RLD domain containing E3 ubiquitin protein ligase family member 6	HERC6	1,00
WD repeat domain 44	WDR44	1,00
kallikrein related peptidase 8	KLK8	1,00
lysophospholipase II	LYPLA2	1,00
solute carrier family 35, member E2	SLC35E2	1,00
DEAD (Asp-Glu-Ala-Asp) box helicase 56	DDX56	1,00
Yip1 domain family member 5	YIPF5	1,00
mitochondrial pyruvate carrier 1	MPC1	1,00
coiled-coil domain containing 66	CCDC66	1,00
gamma-aminobutyric acid (GABA) A receptor, alpha 2	GABRA2	1,00
thioredoxin domain containing 8 (spermatozoa)	TXNDC8	1,00
transducin (beta)-like 1X-linked	TBL1X	1,00
RNA binding motif protein, Y-linked, family 1, member E; RNA binding motif protein, Y-linked, family 1, member D; RNA binding motif protein, Y-linked, family 1, member B	RBMY1E; RBMY1D; RBMY1B	1,00
Transcript Identified by AceView, Entrez Gene ID(s) 4345; novel transcript	CD200; RP11-90K6.1	1,00
nucleolar protein interacting with the FHA domain of MKI67	NIFK	1,00
mucin 17, cell surface associated	MUC17	1,00
calpain 15; microRNA 5587	CAPN15; MIR5587	1,00
citrate synthase	CS	1,00
signal transducing adaptor family member 2	STAP2	1,00
crystallin gamma D	CRYGD	1,00
RAB31, member RAS oncogene family	RAB31	1,00
LON peptidase N-terminal domain and ring finger 2	LONRF2	1,00

LINE-1 type transposase domain containing 1	L1TD1	1,00
secernin 3	SCRN3	1,00
galactosidase beta 1; transmembrane protein with metallophosphoesterase domain	GLB1; TMPPE	1,00
recoverin	RCVRN	1,00
somatostatin receptor 5	SSTR5	1,00
capping protein (actin filament) muscle Z-line, beta	CAPZB	1,00
ribosomal protein S25	RPS25	1,00
Zhang2013 ALT_ACCEPTOR, ALT_DONOR, coding, INTERNAL, intronic best transcript NM_032447; Transcript Identified by AceView	FBN3; mumari	1,00
calpain 11	CAPN11	1,00
chromosome 11 open reading frame 24	C11orf24	1,00
ovo-like zinc finger 3	OVOL3	1,00
nitric oxide synthase 1 (neuronal) adaptor protein	NOS1AP	1,00
chitinase 1 (chitotriosidase)	CHIT1	1,00
chromosome 16 open reading frame 45	C16orf45	1,00
solute carrier family 25, member 47	SLC25A47	1,00
PBX/knotted 1 homeobox 1	PKNOX1	1,00
Transcript Identified by AceView, Entrez Gene ID(s) 284440; 440515	ZNF506	1,00
ribosomal protein S28	RPS28	1,00
protein disulfide isomerase family A, member 5; microRNA 7110	PDIA5; MIR7110	1,00
fibrosin	FBRS	1,00
translocase of inner mitochondrial membrane domain containing 1	TIMMDC1	1,00
ADAM metallopeptidase domain 20	ADAM20	1,00
SUZ RNA binding domain containing 1	SZRD1	1,00
diacylglycerol kinase, epsilon 64kDa	DGKE	1,00
ST20 antisense RNA 1	ST20-AS1	1,00
DEAD/H (Asp-Glu-Ala-Asp/His) box helicase 11	DDX11	1,00
forkhead box K2	FOXK2	1,00
Memczak2013 ANTISENSE, CDS, coding, INTERNAL best transcript NM_004946	DOCK2	1,00
Transcript Identified by AceView, Entrez Gene ID(s) 54715	A2BP1	1,00
surfactant protein A2; surfactant protein A1	SFTPA2; SFTPA1	1,00
neuroglobin	NGB	1,00
Morf4 family associated protein 1	MRFAP1	1,00
UDP-Gal:betaGlcNAc beta 1,3-galactosyltransferase 5	B3GALT5	1,00
solute carrier family 35 (UDP-GlcNAc/UDP-glucose transporter), member D2	SLC35D2	1,00
olfactory receptor, family 2, subfamily Z, member 1	OR2Z1	1,00
chromosome 19 open reading frame 44	C19orf44	1,00

sushi-repeat containing protein, X-linked 2	SRPX2	1,00
leucine rich repeat containing 37B	LRRC37B	1,00
KIAA1143 [Source:HGNC Symbol;Acc:HGNC:29198]	KIAA1143	1,00
OTU deubiquitinase with linear linkage specificity	OTULIN	1,00
myosin IC	MYO1C	1,00
Olfactory receptor 4N4 [Source:UniProtKB/Swiss-Prot;Acc:Q8N0Y3]	AC171558.1	1,00
BRCA1-associated ATM activator 1	BRAT1	1,00
family with sequence similarity 96, member B	FAM96B	1,00
kallikrein related peptidase 11	KLK11	1,00
dynactin 2 (p50)	DCTN2	1,00
arginyl-tRNA synthetase	RARS	1,00
ubiquitin protein ligase E3 component n-recognin 3 (putative)	UBR3	1,00
glycine-N-acyltransferase-like 1	GLYATL1	1,00
DDHD domain containing 2	DDHD2	1,00
myelin expression factor 2	MYEF2	1,00
deoxyguanosine kinase	DGUOK	1,00
adaptor protein, phosphotyrosine interaction, PH domain and leucine zipper containing 2	APPL2	1,00
frizzled class receptor 10	FZD10	1,00
mediator complex subunit 13	MED13	1,00
eukaryotic translation initiation factor 1B	EIF1B	1,00
CAP-GLY domain containing linker protein 3	CLIP3	1,00
WD repeat domain 82	WDR82	1,00
MAGI family member, X-linked	MAGIX	1,00
PQ loop repeat containing 2	PQLC2	1,00
trace amine associated receptor 1	TAAR1	1,00
bromo adjacent homology domain containing 1	BAHD1	1,00
solute carrier family 15 (oligopeptide transporter), member 2	SLC15A2	1,00
MX dynamin-like GTPase 2	MX2	1,00
taste receptor, type 2, member 41	TAS2R41	1,00
MAGE family member D2	MAGED2	1,00
related RAS viral (r-ras) oncogene homolog 2	RRAS2	1,00
interleukin 1 receptor associated kinase 1; microRNA 718	IRAK1; MIR718	1,00
proteasome subunit beta 6	PSMB6	1,00
dynein, light chain, LC8-type 1	DYNLL1	1,00
microtubule associated serine/threonine kinase-like	MASTL	1,00
osteomodulin	OMD	1,00
leucine rich repeat containing 10	LRRC10	1,00
uroplakin 3B	UPK3B	1,00
eukaryotic translation elongation factor 1 beta 2; small nucleolar RNA, H/ACA box 41	EEF1B2; SNORA41	1,00
biphenyl hydrolase-like (serine hydrolase)	BPHL	1,00
zinc finger protein 205	ZNF205	1,00
ORAI calcium release-activated calcium modulator 2	ORAI2	1,00

integral membrane protein 2C	ITM2C	1,00
tetraspanin 2	TSPAN2	1,00
ubiquitin specific peptidase 2	USP2	1,00
phospholipase D family, member 4	PLD4	1,00
protease, serine, 21 (testisin)	PRSS21	1,00
RASSF1 antisense RNA 1	RASSF1-AS1	1,00
ribonuclease P/MRP 30kDa subunit	RPP30	1,00
dedicator of cytokinesis 10	DOCK10	1,00
ubiquinol-cytochrome c reductase binding protein	UQCRB	1,00
glycophorin C (Gerbich blood group)	GYPC	1,00
fibrillarin	FBL	0,99
adaptor-related protein complex 3, beta 1 subunit	AP3B1	0,99
chromosome 19 open reading frame 18	C19orf18	0,99
microRNA 7162; golgin A2 pseudogene 6; mitochondrial poly(A) polymerase	MIR7162; GOLGA2P6; MTPAP	0,99
olfactory receptor, family 9, subfamily Q, member 1	OR9Q1	0,99
S-antigen; retina and pineal gland (arrestin)	SAG	0,99
chromosome 2 open reading frame 88	C2orf88	0,99
Wiskott-Aldrich syndrome	WAS	0,99
copine III	CPNE3	0,99
ubiquitin-conjugating enzyme E2S; ubiquitin conjugating enzyme E2S pseudogene 1	UBE2S; UBE2SP1	0,99
caspase 16, pseudogene	CASP16P	0,99
zinc finger protein 780A	ZNF780A	0,99
RAB29, member RAS oncogene family	RAB29	0,99
olfactory receptor, family 5, subfamily B, member 2	OR5B2	0,99
sorting nexin 29 pseudogene 2; sorting nexin 29 pseudogene 1	SNX29P2; SNX29P1	0,99
chromosome 17 open reading frame 80	C17orf80	0,99
heterogeneous nuclear ribonucleoprotein U-like 1	HNRNPUL1	0,99
major histocompatibility complex, class II, DQ alpha 2	HLA-DQA2	0,99
protein phosphatase 1, regulatory (inhibitor) subunit 1C	PPP1R1C	0,99
family with sequence similarity 58, member A	FAM58A	0,99
annexin A2 receptor	ANXA2R	0,99
PRAME family member 19 [Source:EntrezGene;Acc:645414]	PRAMEF19	0,99
ribosomal protein S21	RPS21	0,99
nuclear receptor corepressor 2	NCOR2	0,99
propionyl-CoA carboxylase alpha subunit	PCCA	0,99
zinc finger protein 446	ZNF446	0,99
major facilitator superfamily domain containing 14C	MFSD14C	0,99
downstream neighbor of SON; crystallin zeta like 1	DONSON; CRYZL1	0,99
kinesin family member 17	KIF17	0,99

exocyst complex component 7; microRNA 6868	EXOC7; MIR6868	0,99
nuclear pore complex interacting protein family, member A3	NPIPA3	0,99
autoimmune regulator	AIRE	0,99
zinc finger protein 22	ZNF22	0,99
cytochrome P450, family 4, subfamily F, polypeptide 2	CYP4F2	0,99
forkhead box L1	FOXL1	0,99
zinc finger, DHHC-type containing 3	ZDHHC3	0,99
golgi reassembly stacking protein 2	GORASP2	0,99
chromosome 1 open reading frame 53	C1orf53	0,99
AT rich interactive domain 3B (BRIGHT-like)	ARID3B	0,99
chromodomain helicase DNA binding protein 1-like	CHD1L	0,99
corticotropin releasing hormone receptor 1	CRHR1	0,99
glucosamine (N-acetyl)-6-sulfatase	GNS	0,99
NCK adaptor protein 2	NCK2	0,99
damage-specific DNA binding protein 2	DDB2	0,99
syntaphilin	SNPH	0,99
mitogen-activated protein kinase associated protein 1	MAPKAP1	0,99
MAP3K7 C-terminal like	MAP3K7CL	0,99
TAF9B RNA polymerase II, TATA box binding protein (TBP)-associated factor, 31kDa	TAF9B	0,99
CD59 molecule, complement regulatory protein	CD59	0,99
tankyrase, TRF1-interacting ankyrin-related ADP-ribose polymerase 2	TNKS2	0,99
leucine rich repeat containing 40	LRRC40	0,99
adiponectin receptor 1	ADIPOR1	0,99
aryl hydrocarbon receptor interacting protein	AIP	0,99
unc-5 netrin receptor D	UNC5D	0,99
acylglycerol kinase	AGK	0,99
ankyrin repeat, SAM and basic leucine zipper domain containing 1	ASZ1	0,99
COX20 cytochrome c oxidase assembly factor	COX20	0,99
striatin, calmodulin binding protein 3	STRN3	0,99
immature colon carcinoma transcript 1	ICT1	0,99
hypoxia up-regulated 1	HYOU1	0,99
uridine phosphorylase 2	UPP2	0,99
solute carrier family 22, member 14	SLC22A14	0,99
family with sequence similarity 217, member A	FAM217A	0,99
mitochondrial ribosomal protein S16	MRPS16	0,99
ARPC4-TTLL3 readthrough	ARPC4-TTLL3	0,99
diacylglycerol kinase, delta 130kDa	DGKD	0,99
nuclear factor of activated T-cells 5, tonicity-responsive	NFAT5	0,99
serine peptidase inhibitor, Kazal type 2 (acrosin-trypsin inhibitor)	SPINK2	0,99
catenin (cadherin-associated protein), alpha 3	CTNNA3	0,99

zinc finger protein 766; microRNA 643	ZNF766; MIR643	0,99
cyclin G1	CCNG1	0,99
chemokine (C-C motif) receptor 8	CCR8	0,99
polymerase (RNA) II (DNA directed) polypeptide A, 220kDa	POLR2A	0,99
UDP-glucose glycoprotein glucosyltransferase 2	UGGT2	0,99
forkhead-associated (FHA) phosphopeptide binding domain 1	FHAD1	0,99
ADAMTS like 3	ADAMTSL3	0,99
proliferating cell nuclear antigen	PCNA	0,99
chromosome 1 open reading frame 27	C1orf27	0,99
Purkinje cell protein 4 like 1	PCP4L1	0,99
NADH dehydrogenase (ubiquinone) Fe-S protein 4, 18kDa (NADH-coenzyme Q reductase)	NDUFS4	0,99
carnosine dipeptidase 1 (metallopeptidase M20 family)	CNDP1	0,99
interleukin 37	IL37	0,99
integrin alpha 8	ITGA8	0,99
NLR family member X1	NLRX1	0,99
MAD2 mitotic arrest deficient-like 1 (yeast)	MAD2L1	0,99
polybromo 1	PBRM1	0,99
UPF2 regulator of nonsense transcripts homolog (yeast)	UPF2	0,99
zinc finger protein 787	ZNF787	0,99
nucleolar protein 6 (RNA-associated)	NOL6	0,99
MAK16 homolog	MAK16	0,99
collagen, type XI, alpha 1	COL11A1	0,99
solute carrier family 35, member E2B	SLC35E2B	0,99
zygote arrest 1-like	ZAR1L	0,99
eukaryotic translation initiation factor 2-alpha kinase 2	EIF2AK2	0,99
collagen beta(1-O)galactosyltransferase 2	COLGALT2	0,99
superoxide dismutase 3, extracellular	SOD3	0,99
sema domain, immunoglobulin domain (Ig), short basic domain, secreted, (semaphorin) 3F	SEMA3F	0,99
olfactory receptor, family 13, subfamily C, member 8	OR13C8	0,99
coiled-coil domain containing 144A	CCDC144A	0,99
zinc finger protein 654	ZNF654	0,99
protein phosphatase, Mg2+/Mn2+ dependent, 1D	PPM1D	0,99
endothelin receptor type B	EDNRB	0,99
solute carrier family 38, member 1	SLC38A1	0,99
deoxythymidylate kinase	DTYMK	0,99
goosecoid homeobox 2	GSC2	0,99
ZHX1-C8orf76 readthrough	ZHX1-C8orf76	0,99
lymphocyte antigen 6 complex, locus G5B; casein kinase 2, beta polypeptide	LY6G5B; CSNK2B	0,99
COBW domain containing 1	CBWD1	0,99
stearoyl-CoA desaturase (delta-9-desaturase)	SCD	0,99
CMT1A duplicated region transcript 15	CDRT15	0,99

cytochrome b561 family, member D2	CYB561D2	0,99
proline-rich protein BstNI subfamily 3	PRB3	0,99
chromosome 22 open reading frame 39; histone cell cycle regulator	C22orf39; HIRA	0,99
regulator of chromosome condensation (RCC1) and BTB (POZ) domain containing protein 1	RCBTB1	0,99
insulin like growth factor binding protein 6	IGFBP6	0,99
DIS3 like exosome 3-5 exoribonuclease	DIS3L	0,99
perilipin 5	PLIN5	0,99
ring finger protein 208	RNF208	0,99
phospholipase C, delta 1	PLCD1	0,99
ADAM metallopeptidase with thrombospondin type 1 motif 13	ADAMTS13	0,99
phosphatidylinositol-4,5-bisphosphate 3-kinase, catalytic subunit beta	PIK3CB	0,99
extended synaptotagmin-like protein 3	ESYT3	0,99
ubiquitin specific peptidase 28	USP28	0,99
tripartite motif containing 38	TRIM38	0,99
myosin VB	MYO5B	0,99
RNA binding motif protein 19	RBM19	0,99
diacylglycerol kinase, delta 130kDa	DGKD	0,99
zinc finger protein 256	ZNF256	0,99
steroid-5-alpha-reductase, alpha polypeptide 1 (3-oxo-5 alpha-steroid delta 4-dehydrogenase alpha 1)	SRD5A1	0,99
lymphocyte antigen 6 complex, locus G6D	LY6G6D	0,99
Memczak2013 ALT_ACCEPTOR, ALT_DONOR, coding, INTERNAL, intronic best transcript NM_020896	OSBPL5	0,99
PRAME family member 6 [Source:HGNC Symbol;Acc:HGNC:30583]	PRAMEF6	0,99
death effector domain containing	DEDD	0,99
importin 7; small nucleolar RNA, H/ACA box 23	IPO7; SNORA23	0,99
proline-rich protein BstNI subfamily 4	PRB4	0,99
syntrophin, beta 1 (dystrophin-associated protein A1, 59kDa, basic component 1)	SNTB1	0,99
protein phosphatase 1, regulatory (inhibitor) subunit 14C	PPP1R14C	0,99
testis expressed 13A	TEX13A	0,99
chemerin chemokine-like receptor 1	CMKLR1	0,99
macrophage expressed 1	MPEG1	0,99
ferritin, heavy polypeptide 1	FTH1	0,99
zinc finger, MYND-type containing 11	ZMYND11	0,99
1-acylglycerol-3-phosphate O-acyltransferase 5	AGPAT5	0,99
FK506 binding protein 5	FKBP5	0,99
proteasome activator subunit 4	PSME4	0,99
paternally expressed 3; zinc finger, imprinted 2	PEG3; ZIM2	0,99
calcium binding tyrosine-(Y)-phosphorylation regulated	CABYR	0,99

corneodesmosin	CDSN	0,99
leucine zipper protein 6; myotrophin	LUZP6; MTPN	0,99
thiopurine S-methyltransferase	TPMT	0,99
spermatogenesis associated 2-like	SPATA2L	0,99
ash1 (absent, small, or homeotic)-like (Drosophila); microRNA 555	ASH1L; MIR555	0,99
membrane associated guanylate kinase, WW and PDZ domain containing 1	MAGI1	0,99
metallothionein 1A	MT1A	0,99
guanylate cyclase 2F, retinal	GUCY2F	0,99
phosphatidylinositol transfer protein, membrane-associated 1	PITPNM1	0,99
tripartite motif containing 50	TRIM50	0,99
calcineurin-like phosphoesterase domain containing 1	CPPED1	0,99
aarF domain containing kinase 4	ADCK4	0,99
tripartite motif containing 17	TRIM17	0,99
mitochondrial ribosomal protein S26	MRPS26	0,99
NADH dehydrogenase (ubiquinone) 1 beta subcomplex, 8, 19kDa	NDUFB8	0,99
tetratricopeptide repeat domain 3	TTC3	0,99
denticleless E3 ubiquitin protein ligase homolog (Drosophila)	DTL	0,99
KIAA1210	KIAA1210	0,99
cell division cycle 7	CDC7	0,99
3-hydroxy-3-methylglutaryl-CoA reductase	HMGCR	0,99
PDZ binding kinase	PBK	0,99
ADP-ribosylation factor like GTPase 9	ARL9	0,99
chromosome 5 open reading frame 47	C5orf47	0,99
transmembrane protein 14C	TMEM14C	0,99
trace amine associated receptor 5	TAAR5	0,99
trans-2,3-enoyl-CoA reductase; microRNA 639	TECR; MIR639	0,99
zinc finger protein 24	ZNF24	0,99
TDP-glucose 4,6-dehydratase	TGDS	0,99
NADH dehydrogenase (ubiquinone) Fe-S protein 1, 75kDa (NADH-coenzyme Q reductase); glycine cleavage system protein H (aminomethyl carrier) pseudogene 3	NDUFS1; GCSHP3	0,99
leucine rich repeat containing 38	LRRC38	0,99
serine/threonine kinase 31	STK31	0,99
erythropoietin receptor	EPOR	0,99
methyltransferase like 14	METTL14	0,99
glucosaminyl (N-acetyl) transferase 4, core 2	GCNT4	0,99
protein phosphatase 1, regulatory subunit 9A	PPP1R9A	0,99
phosphate regulating endopeptidase homolog, X-linked	PHEX	0,99
alkB homolog 6	ALKBH6	0,99
mex-3 RNA binding family member B	MEX3B	0,99
ubiquitin C-terminal hydrolase L5	UCHL5	0,99
solute carrier family 30 (zinc transporter), member 1	SLC30A1	0,99
SATB homeobox 1	SATB1	0,99

replication factor C subunit 4	RFC4	0,99
speedy/RINGO cell cycle regulator family member E4	SPDYE4	0,99
interleukin 16	IL16	0,99
IQ motif and Sec7 domain 1	IQSEC1	0,99
guanine nucleotide binding protein (G protein), alpha 13	GNA13	0,99
acyl-CoA dehydrogenase family, member 8	ACAD8	0,99
mitochondrial E3 ubiquitin protein ligase 1	MUL1	0,99
DDB1 and CUL4 associated factor 7	DCAF7	0,99
glioblastoma amplified sequence	GBAS	0,99
FAM3C pseudogene	FAM3C2	0,99
dickkopf-like 1	DKKL1	0,99
fyn-related Src family tyrosine kinase	FRK	0,99
WD repeat domain 61	WDR61	0,99
chromosome 4 open reading frame 33	C4orf33	0,99
mortality factor 4 like 1	MORF4L1	0,99
ribosomal protein SA pseudogene 58; ribosomal protein SA	RPSAP58; RPSA	0,99
CDC28 protein kinase regulatory subunit 1B	CKS1B	0,99
NLR family, pyrin domain containing 12	NLRP12	0,99
Transcript Identified by AceView, Entrez Gene ID(s) 378465; 65125	WNK1	0,99
chemokine (C-C motif) ligand 27	CCL27	0,99
bactericidal/permeability-increasing protein	BPI	0,99
family with sequence similarity 212, member B	FAM212B	0,99
thymosin beta 15B	TMSB15B	0,99
glucose-6-phosphate dehydrogenase	G6PD	0,99
EH domain binding protein 1 like 1	EHBP1L1	0,99
tRNA 5-methylaminomethyl-2-thiouridylate methyltransferase	TRMU	0,99
GS homeobox 1	GSX1	0,99
pancreas specific transcription factor, 1a	PTF1A	0,99
calcium binding protein 4	CABP4	0,99
C9orf135 antisense RNA 1 (head to head)	C9orf135-AS1	0,99
BTG family, member 3	BTG3	0,99
calcium/calmodulin-dependent protein kinase IV	CAMK4	0,99
SAMM50 sorting and assembly machinery component	SAMM50	0,99
sulfotransferase family 1E member 1	SULT1E1	0,99
zinc finger protein 415	ZNF415	0,99
inhibin alpha	INHA	0,99
adaptor-related protein complex 1, gamma 2 subunit	AP1G2	0,99
t-complex-associated-testis-expressed 1	TCTE1	0,99
ELL associated factor 1	EAF1	0,99
neuroblastoma 1, DAN family BMP antagonist	NBL1	0,99
mediator complex subunit 6	MED6	0,99
zinc finger protein 589	ZNF589	0,99
	LRIG1	0,99

torsin A interacting protein 1	TOR1AIP1	0,99
RAB43, member RAS oncogene family	RAB43	0,99
StAR-related lipid transfer domain containing 8	STARD8	0,99
mitogen-activated protein kinase 4	MAPK4	0,99
APAF1 interacting protein	APIP	0,99
Zhang2013 ALT_ACCEPTOR, ALT_DONOR, coding, INTERNAL, intronic best transcript NM_015179	RRP12	0,99
chromosome 1 open reading frame 122	C1orf122	0,99
karyopherin alpha 6 (importin alpha 7)	KPNA6	0,99
zinc finger protein 234	ZNF234	0,99
TIMELESS interacting protein	TIPIN	0,99
solute carrier family 29 (equilibrative nucleoside transporter), member 2	SLC29A2	0,99
potassium channel, voltage gated modifier subfamily V, member 1	KCNV1	0,99
chromosome 15 open reading frame 52	C15orf52	0,99
nipsnap homolog 1 (C. elegans)	NIPSNAP1	0,99
acyl-CoA synthetase medium-chain family member 2A	ACSM2A	0,99
zinc finger protein 721; ATP binding cassette subfamily A member 11, pseudogene	ZNF721; ABCA11P	0,99
chromosome 17 open reading frame 99	C17orf99	0,99
Y box binding protein 3	YBX3	0,99
zinc finger protein 776	ZNF776	0,99
platelet-derived growth factor receptor-like	PDGFRL	0,99
hydrolethalus syndrome 1	HYLS1	0,99
interleukin 1 receptor associated kinase 3; microRNA 6502	IRAK3; MIR6502	0,99
HOXA cluster antisense RNA 3	HOXA-AS3	0,99
ubiquitin specific peptidase 9, Y-linked; testis-specific transcript, Y-linked 15 (non-protein coding)	USP9Y; TTTY15	0,99
neurexophilin and PC-esterase domain family, member 4	NXPE4	0,99
G protein-coupled receptor 37 (endothelin receptor type B-like)	GPR37	0,99
bone morphogenetic protein 3	BMP3	0,99
activating signal cointegrator 1 complex subunit 2	ASCC2	0,99
succinate dehydrogenase complex subunit A, flavoprotein (Fp)	SDHA	0,99
transmembrane protease, serine 15	TMPRSS15	0,99
zinc finger protein 566	ZNF566	0,99
Ras protein-specific guanine nucleotide-releasing factor 1	RASGRF1	0,99
cysteine/histidine-rich 1	CYHR1	0,99
WD repeat domain 19	WDR19	0,99
interleukin 17 receptor E	IL17RE	0,99
acetyl-CoA acetyltransferase 2	ACAT2	0,99
UBX domain protein 1	UBXN1	0,99
ArfGAP with coiled-coil, ankyrin repeat and PH domains 2	ACAP2	0,99
l(3)mbt-like 4 (Drosophila)	L3MBTL4	0,99

MpV17 mitochondrial inner membrane protein	MPV17	0,99
zinc finger protein 665	ZNF665	0,99
family with sequence similarity 107, member A	FAM107A	0,99
MAGE family member C1	MAGEC1	0,99
chromosome 1 open reading frame 234	C1orf234	0,99
WNT inhibitory factor 1	WIF1	0,99
ALG3, alpha-1,3- mannosyltransferase	ALG3	0,99
EF-hand domain family member D1	EFHD1	0,99
testis specific protein, Y-linked 8	TSPY8	0,99
keratin 34, type I	KRT34	0,99
NADH dehydrogenase, subunit 5 (complex I)	ND5	0,99
F-box protein 16	FBXO16	0,99
bolA family member 2B; bolA family member 2	BOLA2B; BOLA2	0,99
Ras and Rab interactor 3	RIN3	0,99
C-type lectin domain family 18, member A	CLEC18A	0,99
olfactory receptor, family 2, subfamily A, member 42; olfactory receptor, family 2, subfamily A, member 1	OR2A42; OR2A1	0,99
interferon-related developmental regulator 2	IFRD2	0,99
glycolipid transfer protein domain containing 2	GLTPD2	0,99
mitofusin 1	MFN1	0,99
hedgehog acyltransferase-like	HHATL	0,99
dual specificity phosphatase 21	DUSP21	0,99
transcriptional adaptor 2B	TADA2B	0,99
olfactory receptor, family 51, subfamily G, member 2	OR51G2	0,99
methenyltetrahydrofolate synthetase domain containing	MTHFSD	0,99
protein arginine methyltransferase 5	PRMT5	0,99
Eukaryotic translation initiation factor 3 subunit F [Source:UniProtKB/Swiss-Prot;Acc:O00303]	EIF3F	0,99
MT-RNR2-like 2	MTRNR2L2	0,99
nucleosome assembly protein 1-like 2	NAP1L2	0,99
T-cell acute lymphocytic leukemia 1	TAL1	0,99
NRSN2 antisense RNA 1	NRSN2-AS1	0,99
zinc finger and BTB domain containing 7B	ZBTB7B	0,99
sema domain, immunoglobulin domain (Ig), short basic domain, secreted, (semaphorin) 3C	SEMA3C	0,99
SH3-domain GRB2-like endophilin B2	SH3GLB2	0,99
OMA1 zinc metallopeptidase	OMA1	0,99
NHL repeat containing 4; phosphatidylinositol glycan anchor biosynthesis class Q	NHLRC4; PIGQ	0,99
heparan sulfate 2-O-sulfotransferase 1	HS2ST1	0,99
diacylglycerol kinase, beta 90kDa	DGKB	0,99
ubiquitin-conjugating enzyme E2Z	UBE2Z	0,99
tubulin folding cofactor E-like	TBCEL	0,99
hypocretin (orexin) neuropeptide precursor	HCRT	0,99

testis expressed 22	TEX22	0,99
protocadherin beta 11	PCDHB11	0,99
E1A binding protein p300; microRNA 1281	EP300; MIR1281	0,99
vascular endothelial zinc finger 1	VEZF1	0,99
Alstrom syndrome protein 1	ALMS1	0,99
Memczak2013 ANTISENSE, CDS, coding, INTERNAL best transcript NM_006044	HDAC6	0,99
LDL receptor related protein 11	LRP11	0,99
uncharacterized LOC442028; Transcript Identified by AceView, Entrez Gene ID(s) 729171	LOC442028; ANKRD20B	0,99
interactor of little elongation complex ELL subunit 2	ICE2	0,99
Memczak2013 ALT_ACCEPTOR, ALT_DONOR, coding, INTERNAL, intronic best transcript NM_001076787	TP53I11	0,99
interleukin 36, alpha	IL36A	0,99
desert hedgehog	DHH	0,99
karyopherin alpha 7 (importin alpha 8)	KPNA7	0,99
pleckstrin homology-like domain, family B, member 2; phosphatidylinositol-specific phospholipase C, X domain containing 2	PHLDB2; PLCXD2	0,99
Ral GEF with PH domain and SH3 binding motif 1	RALGPS1	0,99
ribosomal protein L36a	RPL36A	0,99
zinc finger protein 839	ZNF839	0,99
Memczak2013 ALT_ACCEPTOR, ALT_DONOR, coding, INTERNAL, intronic best transcript NM_022480	KLHL25	0,99
Yip1 domain family member 4	YIPF4	0,99
protein phosphatase 2, regulatory subunit B, delta	PPP2R2D	0,99
long intergenic non-protein coding RNA 493	LINC00493	0,99
Homo sapiens mitochondrial ribosomal protein L23 (MRPL23), mRNA.; 39S ribosomal protein L23, mitochondrial-like; Homo sapiens mitochondrial ribosomal protein L23, mRNA (cDNA clone MGC:12795 IMAGE:4298000), complete cds.	MRPL23; LOC102724828; AC004556.1	0,99
basal cell adhesion molecule (Lutheran blood group)	BCAM	0,99
GLTSCR1-like	GLTSCR1L	0,99
3-oxoacid CoA-transferase 2	OXCT2	0,99
coiled-coil domain containing 67	CCDC67	0,99
MPV17 mitochondrial membrane protein-like 2	MPV17L2	0,99
leucine rich repeat transmembrane neuronal 1	LRRTM1	0,99
yippee like 2	YPEL2	0,99
zinc finger CCCH-type containing 15	ZC3H15	0,99
collagen, type VIII, alpha 2	COL8A2	0,99
solute carrier family 29 (equilibrative nucleoside transporter), member 1	SLC29A1	0,99
N-acetyltransferase 8-like (GCN5-related, putative)	NAT8L	0,99
CCAAT/enhancer binding protein (C/EBP), epsilon	CEBPE	0,99

tripartite motif containing 24	TRIM24	0,99
WD repeat domain 37	WDR37	0,99
APEX nuclease (multifunctional DNA repair enzyme) 1	APEX1	0,99
selectin L	SELL	0,99
patched domain containing 2	PTCHD2	0,99
olfactory receptor, family 7, subfamily C, member 2	OR7C2	0,99
interleukin 10 receptor, beta	IL10RB	0,99
notch 2	NOTCH2	0,99
solute carrier family 35 (UDP-GlcA/UDP-GalNAc transporter), member D1	SLC35D1	0,99
growth differentiation factor 9	GDF9	0,99
TAF6-like RNA polymerase II, p300/CBP-associated factor (PCAF)-associated factor, 65kDa	TAF6L	0,99
maestro heat-like repeat family member 7; tetratricopeptide repeat domain 4; MROH7-TTC4 readthrough (NMD candidate)	MROH7; TTC4; MROH7-TTC4	0,99
Memczak2013 ANTISENSE, coding, INTERNAL, UTR3 best transcript NM_005188	CBL	0,99
nucleophosmin (nucleolar phosphoprotein B23, numatrin)	NPM1	0,99
gelsolin	GSN	0,99
olfactory receptor, family 9, subfamily Q, member 2	OR9Q2	0,99
splicing factor 3b, subunit 4, 49kDa	SF3B4	0,99
calmodulin 2 (phosphorylase kinase, delta)	CALM2	0,99
zinc finger protein 229	ZNF229	0,99
guanine nucleotide binding protein (G protein), beta polypeptide 2-like 1; small nucleolar RNA, C/D box 95; small nucleolar RNA, C/D box 96A	GNB2L1; SNORD95; SNORD96A	0,99
malate dehydrogenase 1	MDH1	0,99
protein tyrosine phosphatase, receptor type, Q	PTPRQ	0,99
protease, serine, 2 (trypsin 2)	PRSS2	0,99
ring finger protein 138, E3 ubiquitin protein ligase	RNF138	0,99
dehydrogenase/reductase (SDR family) member 12	DHRS12	0,99
POM121 transmembrane nucleoporin	POM121	0,99
olfactory receptor, family 13, subfamily G, member 1	OR13G1	0,99
NmrA-like family domain containing 1	NMRAL1	0,99
NUT family member 2F	NUTM2F	0,99
RPA interacting protein	RPAIN	0,99
ureidoimidazoline (2-oxo-4-hydroxy-4-carboxy-5-) decarboxylase	URAD	0,99
protein phosphatase 1, regulatory (inhibitor) subunit 2	PPP1R2	0,99
EGF domain-specific O-linked N-acetylglucosamine (GlcNAc) transferase	EOGT	0,99
olfactory receptor, family 2, subfamily T, member 2	OR2T2	0,99
interferon-induced protein with tetratricopeptide repeats 3	IFIT3	0,99
Ras association (RalGDS/AF-6) domain family member 2	RASSF2	0,99
doublecortin domain containing 1	DCDC1	0,99
Transcript Identified by AceView, Entrez Gene ID(s) 205564	SENP5	0,99

fucosidase, alpha-L- 2, plasma	FUCA2	0,99
C-type lectin domain family 18, member B	CLEC18B	0,99
WD repeat domain 3	WDR3	0,99
cyclin T2	CCNT2	0,99
PACRG antisense RNA 3	PACRG-AS3	0,99
chromosome 8 open reading frame 48	C8orf48	0,99
myozenin 2	MYOZ2	0,99
N-acetyltransferase domain containing 1	NATD1	0,99
fibronectin leucine rich transmembrane protein 2	FLRT2	0,99
myosin XVIIIB	MYO18B	0,99
frizzled class receptor 4	FZD4	0,99
forkhead box R2	FOXR2	0,99
zinc finger protein 846	ZNF846	0,99
24-dehydrocholesterol reductase	DHCR24	0,99
strawberry notch homolog 2 (Drosophila)	SBNO2	0,99
cyclin-dependent kinase 14	CDK14	0,99
RB1-inducible coiled-coil 1	RB1CC1	0,99
nucleoporin 133kDa	NUP133	0,99
coiled-coil domain containing 124	CCDC124	0,99
homeobox B6	HOXB6	0,99
CUGBP, Elav-like family member 5	CELF5	0,99
pre-mRNA processing factor 18	PRPF18	0,99
heparan sulfate (glucosamine) 3-O-sulfotransferase 4	HS3ST4	0,99
ATP synthase, H+ transporting, mitochondrial Fo complex subunit G	ATP5L	0,99
guanylate binding protein 4	GBP4	0,99
tubulin polyglutamylase complex subunit 1	TPGS1	0,99
TBC1 domain containing kinase	TBCK	0,99
interferon, lambda 3	IFNL3	0,99
acyl-CoA oxidase-like	ACOXL	0,99
Memczak2013 ANTISENSE, CDS, coding, INTERNAL best transcript NM_030821	PLA2G12A	0,99
Transcript Identified by AceView, Entrez Gene ID(s) 114800	CCDC85A	0,99
syndecan 2	SDC2	0,99
alpha-1-microglobulin/bikunin precursor	AMBP	0,99
NADH dehydrogenase (ubiquinone) complex I, assembly factor 2	NDUFAF2	0,99
thioredoxin-related transmembrane protein 1	TMX1	0,99
heterogeneous nuclear ribonucleoprotein L	HNRNPL	0,99
attractin	ATRN	0,99
lipolysis stimulated lipoprotein receptor	LSR	0,99
formin 1	FMN1	0,99
integrin alpha 4	ITGA4	0,99
polymerase (RNA) II (DNA directed) polypeptide D	POLR2D	0,99

Synthetic construct Homo sapiens clone IMAGE:100063377, MGC:190767 ribosomal protein L41 (RPL41) mRNA, encodes complete protein.	RPL41	0,99
zinc finger protein 490	ZNF490	0,99
GIMAP family P-loop NTPase domain containing 1	GIMD1	0,99
actin related protein 2/3 complex subunit 5-like	ARPC5L	0,99
WW domain containing E3 ubiquitin protein ligase 1	WWP1	0,99
THAP domain containing, apoptosis associated protein 1	THAP1	0,99
ataxin 7-like 3	ATXN7L3	0,99
ectodysplasin A	EDA	0,99
Jeck2013 ANTISENSE, CDS, coding, INTERNAL, intronic, OVCODE, OVEXON best transcript NM_005529	HSPG2	0,99
cysteine and glycine-rich protein 2	CSRP2	0,99
F-box protein 30	FBXO30	0,99
potassium channel, sodium activated subfamily T, member 2	KCNT2	0,99
myosin light chain 6B	MYL6B	0,99
mitogen-activated protein kinase kinase kinase 7	MAP3K7	0,99
solute carrier family 25 (mitochondrial carrier; adenine nucleotide translocator), member 5	SLC25A5	0,99
influenza virus NS1A binding protein	IVNS1ABP	0,99
ribosomal protein L7a	RPL7A	0,99
NIMA-related kinase 9	NEK9	0,99
ceramide synthase 1; growth differentiation factor 1	CERS1; GDF1	0,99
TMEM189-UBE2V1 readthrough	TMEM189-UBE2V1	0,99
itchy E3 ubiquitin protein ligase	ITCH	0,99
complexin 1	CPLX1	0,99
androgen-dependent TFPI-regulating protein	ADTRP	0,99
formin 1	FMN1	0,99
zinc finger, SWIM-type containing 5	ZSWIM5	0,99
ribosomal protein S26	RPS26	0,99
calpain 6	CAPN6	0,99
zinc finger protein 410	ZNF410	0,99
aldehyde dehydrogenase 18 family, member A1	ALDH18A1	0,99
WD repeat domain 20	WDR20	0,99
shisa family member 3	SHISA3	0,99
bone morphogenetic protein receptor type IB	BMPR1B	0,99
Transcript Identified by AceView, Entrez Gene ID(s) 11031	RAB31	0,99
CAP, adenylate cyclase-associated protein, 2 (yeast)	CAP2	0,99
zinc finger protein 398	ZNF398	0,99
adaptor-related protein complex 3, sigma 1 subunit	AP3S1	0,99
ER degradation enhancer, mannosidase alpha-like 2	EDEM2	0,99
shisa family member 4	SHISA4	0,99
STE20-related kinase adaptor beta	STRADB	0,99
SR-related CTD-associated factor 4	SCAF4	0,99

fer-1-like family member 5	FER1L5	0,99
negative elongation factor complex member E; microRNA 1236	NELFE; MIR1236	0,99
kinesin family member 3C	KIF3C	0,99
phosphatase and tensin homolog	PTEN	0,99
presenilin 1	PSEN1	0,99
RNA binding motif protein 41	RBM41	0,99
zinc finger protein 143	ZNF143	0,99
Transcript Identified by AceView, Entrez Gene ID(s) 4702	NDUFA8	0,99
interleukin 9 receptor	IL9R	0,99
Homo sapiens interleukin 9 receptor (IL9R), transcript variant 1, mRNA.; Homo sapiens interleukin 9 receptor (IL9R), transcript variant 2, mRNA.; interleukin 9 receptor [Source:HGNC Symbol;Acc:HGNC:6030]; Homo sapiens interleukin 9 receptor, mRNA (cDNA clone MGC:59852 IMAGE:6304829), complete cds.; Transcript Identified by AceView, Entrez Gene ID(s) 3581, RefSeq ID(s) NM_002186; Transcript Identified by AceView, Entrez Gene ID(s) 3581	IL9R	0,99
ubiquitin specific peptidase 35	USP35	0,99
testis expressed 36	TEX36	0,99
NADH dehydrogenase (ubiquinone) 1 beta subcomplex, 2, 8kDa	NDUFB2	0,99
dynein heavy chain domain 1	DNHD1	0,99
ATP synthase, H+ transporting, mitochondrial F1 complex, delta subunit	ATP5D	0,99
leucine rich repeat containing 63	LRRC63	0,99
kelch-like family member 23	KLHL23	0,99
ribosomal protein S15	RPS15	0,99
islet amyloid polypeptide	IAPP	0,99
B-cell scaffold protein with ankyrin repeats 1	BANK1	0,99
Rho guanine nucleotide exchange factor 38	ARHGEF38	0,99
thioredoxin domain containing 15	TXNDC15	0,99
plectin	PLEC	0,99
BCL2/adenovirus E1B 19kDa interacting protein 1	BNIP1	0,99
IQ motif containing F6	IQCF6	0,99
proline/serine-rich coiled-coil 1	PSRC1	0,99
ring finger protein 43	RNF43	0,99
mitochondrial ribosomal protein S35	MRPS35	0,99
family with sequence similarity 173, member A	FAM173A	0,99
filaggrin	FLG	0,99
pre-mRNA processing factor 4B	PRPF4B	0,99
zinc finger and BTB domain containing 24	ZBTB24	0,99
protein phosphatase 1, regulatory subunit 35	PPP1R35	0,99
Smith-Magenis syndrome chromosome region, candidate 8	SMCR8	0,99
Transcript Identified by AceView, Entrez Gene ID(s) 23518	R3HDM1	0,99
carbonic anhydrase II	CA2	0,99

theg spermatid protein	THEG	0,99
transmembrane protein 59-like	TMEM59L	0,99
transforming growth factor beta receptor III like	TGFBR3L	0,99
coiled-coil domain containing 154	CCDC154	0,99
small nuclear ribonucleoprotein, U11/U12 48KDa subunit	SNRNP48	0,99
chromosome 10 open reading frame 82	C10orf82	0,99
regulator of G-protein signaling 17	RGS17	0,99
transmembrane protein 27	TMEM27	0,99
novel transcript; Transcript Identified by AceView, Entrez Gene ID(s) 199964	RP11-67L3.5; RP11-67L3.4; TMEM61	0,99
polymerase (RNA) I polypeptide D	POLR1D	0,99
IMP4 homolog, U3 small nucleolar ribonucleoprotein	IMP4	0,99
zinc finger protein 195	ZNF195	0,99
endoplasmic reticulum to nucleus signaling 2	ERN2	0,99
ADP-ribosylation factor 4	ARF4	0,99
keratin 3, type II	KRT3	0,99
UPF1 regulator of nonsense transcripts homolog (yeast)	UPF1	0,99
neuronal PAS domain protein 4	NPAS4	0,99
polymerase (DNA directed), alpha 2, accessory subunit	POLA2	0,99
solute carrier family 30 (zinc transporter), member 3	SLC30A3	0,99
small nuclear ribonucleoprotein polypeptide E	SNRPE	0,99
DnaJ (Hsp40) homolog, subfamily C, member 7	DNAJC7	0,99
collagen, type XIII, alpha 1	COL13A1	0,99
N(alpha)-acetyltransferase 16, NatA auxiliary subunit	NAA16	0,99
PET100 homolog	PET100	0,99
CTD nuclear envelope phosphatase 1	CTDNEP1	0,99
microtubule associated monooxygenase, calponin and LIM domain containing 1	MICAL1	0,99
jumping translocation breakpoint	JTB	0,99
nicotinamide phosphoribosyltransferase	NAMPT	0,99
leukocyte immunoglobulin-like receptor, subfamily B (with TM and ITIM domains), member 1	LILRB1	0,99
tRNA methyltransferase 2 homolog A	TRMT2A	0,99
src-related kinase lacking C-terminal regulatory tyrosine and N-terminal myristylation sites	SRMS	0,99
ATP binding cassette subfamily G member 5	ABCG5	0,99
neurofascin	NFASC	0,99
ubiquitin conjugating enzyme E2D 2	UBE2D2	0,99
4-hydroxyphenylpyruvate dioxygenase	HPD	0,99
glutathione S-transferase alpha 3	GSTA3	0,99
ELK3, ETS-domain protein (SRF accessory protein 2)	ELK3	0,99
septin 14	sept-14	0,99
transmembrane protein 132A	TMEM132A	0,99
small nuclear ribonucleoprotein polypeptide G	SNRPG	0,99

histone deacetylase 6	HDAC6	0,99
Transcript Identified by AceView, Entrez Gene ID(s) 8896	BUD31	0,99
transmembrane protein 42; microRNA 564	TMEM42; MIR564	0,99
trafficking protein particle complex 4	TRAPPC4	0,99
olfactomedin 3	OLFM3	0,99
WW domain binding protein 11	WBP11	0,99
peptidyl arginine deiminase, type III	PADI3	0,99
exosome component 7	EXOSC7	0,99
leucine-rich repeats and IQ motif containing 4	LRRIQ4	0,99
RWD domain containing 2B	RWDD2B	0,99
zinc finger and SCAN domain containing 25	ZSCAN25	0,99
caseinolytic mitochondrial matrix peptidase proteolytic subunit	CLPP	0,99
coiled-coil domain containing 39	CCDC39	0,99
cytochrome b5 reductase 2	CYB5R2	0,99
ubiquitin conjugating enzyme E2K	UBE2K	0,99
family with sequence similarity 90, member A1	FAM90A1	0,99
aurora kinase A	AURKA	0,99
SRY box 18	SOX18	0,99
solute carrier family 12 (sodium/potassium/chloride transporter), member 1	SLC12A1	0,99
peroxisomal biogenesis factor 3	PEX3	0,99
frizzled class receptor 7	FZD7	0,99
contactin associated protein-like 3 pseudogene 2; contactin associated protein-like 3	CNTNAP3P2; CNTNAP3	0,99
ubiquitin-conjugating enzyme E2L 6	UBE2L6	0,99
novel transcript; Transcript Identified by AceView, Entrez Gene ID(s) 8559; RP11-295P9.3 (from geneSymbol)	RP11-295P9.3; PRPF18	0,99
LysM, putative peptidoglycan-binding, domain containing 4	LYSMD4	0,99
trafficking protein particle complex 6A	TRAPPC6A	0,99
twinfilin actin binding protein 2	TWF2	0,99
neurofilament, medium polypeptide	NEFM	0,99
cadherin 7, type 2	CDH7	0,99
stromal cell-derived factor 2	SDF2	0,99
diacylglycerol kinase, delta 130kDa	DGKD	0,99
proline rich 14	PRR14	0,99
OTU deubiquitinase 3	OTUD3	0,99
lysine (K)-specific methyltransferase 2A	KMT2A	0,99
GLIS family zinc finger 1	GLIS1	0,99
RAR-related orphan receptor C	RORC	0,99
calpain 9	CAPN9	0,99
long intergenic non-protein coding RNA 518	LINC00518	0,99
T-box 19	TBX19	0,99
glutamate receptor, metabotropic 8	GRM8	0,99
hydroxymethylbilane synthase	HMBS	0,99

tRNA methyltransferase 12 homolog (S. cerevisiae)	TRMT12	0,99
sorbin and SH3 domain containing 1	SORBS1	0,99
lysyl oxidase-like 1	LOXL1	0,99
Aly/REF export factor	ALYREF	0,99
mitogen-activated protein kinase 13	MAPK13	0,99
BTB (POZ) domain containing 6	BTBD6	0,99
olfactory receptor, family 6, subfamily V, member 1	OR6V1	0,99
spindlin family, member 2A	SPIN2A	0,99
multiple EGF-like-domains 10	MEGF10	0,99
CUB and Sushi multiple domains 2	CSMD2	0,99
unc-119 lipid binding chaperone	UNC119	0,99
tropomyosin 2 (beta)	TPM2	0,99
tripartite motif containing 42	TRIM42	0,99
synapse differentiation inducing 1	SYNDIG1	0,99
zinc finger protein 546	ZNF546	0,99
transcription elongation factor B polypeptide 3C-like 2; transcription elongation factor B polypeptide 3C-like	TCEB3CL2; TCEB3CL	0,99
nuclear transcription factor, X-box binding-like 1	NFXL1	0,99
Transcript Identified by AceView, Entrez Gene ID(s) 23126	POGZ	0,99
solute carrier family 25 (aspartate/glutamate carrier), member 13	SLC25A13	0,99
zinc metallopeptidase STE24	ZMPSTE24	0,99
CaM kinase-like vesicle-associated	CAMKV	0,99
netrin G1	NTNG1	0,99
otopetrin 1	OTOP1	0,99
ATP binding cassette subfamily B member 7	ABCB7	0,99
forkhead box N1	FOXN1	0,99
proteasome subunit beta 8	PSMB8	0,99
alpha-2-HS-glycoprotein	AHSG	0,99
nuclear receptor coactivator 6	NCOA6	0,99
tryptophan hydroxylase 2	TPH2	0,99
SH3 domain binding glutamate-rich protein	SH3BGR	0,99
guanylate cyclase 1, soluble, alpha 2	GUCY1A2	0,99
CD27 molecule	CD27	0,99
ubiquilin 3	UBQLN3	0,99
zinc finger protein 438	ZNF438	0,99
protein phosphatase 2, regulatory subunit B, alpha	PPP2R2A	0,99
RAD18 E3 ubiquitin protein ligase	RAD18	0,99
beta-1,4-glucuronyltransferase 1	B4GAT1	0,99
calsequestrin 2 (cardiac muscle)	CASQ2	0,99
lectin, galactoside-binding-like	LGALSL	0,99
chromodomain helicase DNA binding protein 3	CHD3	0,99
ATPase type 13A2	ATP13A2	0,99
keratin associated protein 13-3	KRTAP13-3	0,99
adhesion G protein-coupled receptor A1	ADGRA1	0,99

Memczak2013 ANTISENSE, coding, INTERNAL, intronic best transcript NM_001024808	BCL7A	0,99
EML2 antisense RNA 1	EML2-AS1	0,99
coiled-coil domain containing 85A	CCDC85A	0,99
SERTA domain containing 2	SERTAD2	0,99
midline 2	MID2	0,99
hydrogen voltage gated channel 1	HVCN1	0,99
pyruvate dehydrogenase complex, component X	PDHX	0,99
neuroligin 4, Y-linked	NLGN4Y	0,99
family with sequence similarity 69, member C	FAM69C	0,99
tripartite motif containing 48	TRIM48	0,99
heat shock 70kDa protein 8; small nucleolar RNA, C/D box 14C; small nucleolar RNA, C/D box 14D	HSPA8; SNORD14C; SNORD14D	0,99
lysophosphatidic acid receptor 2	LPAR2	0,99
interphotoreceptor matrix proteoglycan 1	IMPG1	0,99
suppressor of tumorigenicity 20	ST20	0,99
ATP binding cassette subfamily D member 3	ABCD3	0,99
ubiquitin specific peptidase 43	USP43	0,99
hydroxysteroid (17-beta) dehydrogenase 12	HSD17B12	0,99
F-box protein 6	FBXO6	0,99
adhesion G protein-coupled receptor A3	ADGRA3	0,99
WAP four-disulfide core domain 9	WFDC9	0,99
basic charge, Y-linked, 2C	BPY2C	0,99
BCDIN3 domain containing	BCDIN3D	0,99
transmembrane anterior posterior transformation 1	TAPT1	0,99
steroidogenic acute regulatory protein	STAR	0,99
ATPase, H+ transporting, lysosomal 16kDa, V0 subunit c	ATP6V0C	0,99
G protein pathway suppressor 2	GPS2	0,99
EGF-like-domain, multiple 7	EGFL7	0,99
protein phosphatase 3, catalytic subunit, beta isozyme	PPP3CB	0,99
general transcription factor IIIC subunit 3	GTF3C3	0,99
zinc finger, C3HC-type containing 1	ZC3HC1	0,99
heat shock transcription factor family member 5	HSF5	0,99
gap junction protein delta 2	GJD2	0,99
SPANX family, member N1	SPANXN1	0,99
nudE neurodevelopment protein 1-like 1	NDEL1	0,99
RAN guanine nucleotide release factor	RANGRF	0,99
mitochondrial ribosomal protein L33	MRPL33	0,99
zinc finger protein 782	ZNF782	0,99
AT rich interactive domain 3C (BRIGHT-like)	ARID3C	0,99
5-nucleotidase, cytosolic IA	NT5C1A	0,99
dihydrolipoamide S-acetyltransferase	DLAT	0,99
DnaJ (Hsp40) homolog, subfamily C, member 21	DNAJC21	0,99
cyclin-dependent kinase 15	CDK15	0,99

keratin associated protein 17-1	KRTAP17-1	0,99
neurotensin receptor 1 (high affinity)	NTSR1	0,99
zinc finger protein 735	ZNF735	0,99
cyclin-dependent kinase 20	CDK20	0,99
aryl hydrocarbon receptor	AHR	0,99
CD1a molecule	CD1A	0,99
cytidine monophosphate (UMP-CMP) kinase 2, mitochondrial	CMPK2	0,99
Memczak2013 ANTISENSE, INTERNAL, ncRNA best transcript NR_003587	MYO15B	0,99
Fas cell surface death receptor	FAS	0,99
serine/threonine kinase 32C	STK32C	0,99
signal recognition particle 14kDa	SRP14	0,99
atonal bHLH transcription factor 1	ATOH1	0,99
zinc finger protein 732	ZNF732	0,99
guanine nucleotide binding protein (G protein), gamma 11	GNG11	0,99
proteasome maturation protein	POMP	0,99
THO complex 5	THOC5	0,99
dpy-30 histone methyltransferase complex regulatory subunit	DPY30	0,99
chloride channel, voltage-sensitive Kb	CLCNKB	0,99
transmembrane protein 248	TMEM248	0,99
zinc finger protein 91	ZNF91	0,99
carboxylesterase 5A	CES5A	0,98
nuclear factor of kappa light polypeptide gene enhancer in B-cells 1	NFKB1	0,98
reticulon 4 receptor-like 1	RTN4RL1	0,98
cilia and flagella associated protein 57	CFAP57	0,98
glutamine--fructose-6-phosphate transaminase 1	GFPT1	0,98
microtubule associated protein 4	MAP4	0,98
spalt-like transcription factor 2	SALL2	0,98
tRNA methyltransferase 44 homolog (S. cerevisiae)	TRMT44	0,98
gametogenetin binding protein 2	GGNBP2	0,98
Memczak2013 ANTISENSE, coding, INTERNAL, intronic best transcript NM_014754	PTDSS1	0,98
electron-transfer-flavoprotein, alpha polypeptide	ETFA	0,98
protein kinase, AMP-activated, alpha 2 catalytic subunit	PRKAA2	0,98
multiple coagulation factor deficiency 2	MCFD2	0,98
olfactory receptor, family 5, subfamily C, member 1	OR5C1	0,98
ring finger protein 220	RNF220	0,98
diencephalon/mesencephalon homeobox 1	DMBX1	0,98
chymotrypsin-like elastase family, member 1	CELA1	0,98
hexokinase domain containing 1	HKDC1	0,98
sulfatase modifying factor 2	SUMF2	0,98
solute carrier family 9, subfamily C (Na+-transporting carboxylic acid decarboxylase), member 1	SLC9C1	0,98

potassium channel, voltage gated subfamily A regulatory beta subunit 2	KCNAB2	0,98
NADH dehydrogenase, subunit 4L (complex I); NADH dehydrogenase, subunit 4 (complex I)	ND4L; ND4	0,98
glycine receptor alpha 3	GLRA3	0,98
chromosome 1 open reading frame 111	C1orf111	0,98
prokineticin 2	PROK2	0,98
cholinergic receptor, muscarinic 3	CHRM3	0,98
ribosomal protein S13; small nucleolar RNA, C/D box 14B	RPS13; SNORD14B	0,98
threonyl-tRNA synthetase 2, mitochondrial (putative); microRNA 6878	TARS2; MIR6878	0,98
phosphoribosylglycinamide formyltransferase, phosphoribosylglycinamide synthetase, phosphoribosylaminoimidazole synthetase	GART	0,98
actinin, alpha 1; high mobility group nucleosome binding domain 1 pseudogene 3	ACTN1; HMGN1P3	0,98
ubiquitin-like 5	UBL5	0,98
cancer/testis antigen family 45, member A2	CT45A2	0,98
ISG15 ubiquitin-like modifier	ISG15	0,98
transmembrane protein 65	TMEM65	0,98
heat shock 70kDa protein 1B; heat shock 70kDa protein 1A	HSPA1B; HSPA1A	0,98
V-set domain containing T cell activation inhibitor 1	VTCN1	0,98
zinc finger protein 507	ZNF507	0,98
ribosomal protein L29	RPL29	0,98
filamin C, gamma	FLNC	0,98
guanine nucleotide binding protein (G protein), beta polypeptide 2	GNB2	0,98
radixin	RDX	0,98
transmembrane protein 97	TMEM97	0,98
potassium voltage-gated channel, modifier subfamily S, member 1	KCNS1	0,98
ST6 (alpha-N-acetyl-neuraminyl-2,3-beta-galactosyl-1,3)-N-acetylgalactosaminide alpha-2,6-sialyltransferase 6	ST6GALNAC6	0,98
TOX high mobility group box family member 4	TOX4	0,98
RANBP2-like and GRIP domain containing 8; RANBP2-like and GRIP domain containing 6	RGPD8; RGPD6	0,98
high density lipoprotein binding protein	HDLBP	0,98
chromosome 10 open reading frame 113	C10orf113	0,98
interleukin 33	IL33	0,98
Transcript Identified by AceView, Entrez Gene ID(s) 84173	ELMOD3	0,98
enoyl-CoA delta isomerase 1	ECI1	0,98
serpin peptidase inhibitor, clade A (alpha-1 antiproteinase, antitrypsin), member 7	SERPINA7	0,98

mitochondrial ribosomal protein S24	MRPS24	0,98
serpin peptidase inhibitor, clade B (ovalbumin), member 11 (gene/pseudogene)	SERPINB11	0,98
von Willebrand factor C domain containing 2	VWC2	0,98
Homo sapiens H3 histone, family 3A, pseudogene 4 (H3F3AP4), non-coding RNA.; Homo sapiens H3 histone, family 3A, mRNA (cDNA clone MGC:47761 IMAGE:6014954), complete cds.; Homo sapiens H3 histone, family 3B (H3.3B), mRNA (cDNA clone MGC:87782 IMAGE:3884607), complete cds.; Homo sapiens H3 histone, family 3A, mRNA (cDNA clone MGC:87783 IMAGE:5162817), complete cds.	H3F3AP4; H3F3A; H3F3B	0,98
cyclin-dependent kinase 4	CDK4	0,98
HGF activator	HGFAC	0,98
G protein-coupled bile acid receptor 1	GPBAR1	0,98
complement factor H-related 1	CFHR1	0,98
NOP16 nucleolar protein	NOP16	0,98
polymerase (RNA) II (DNA directed) polypeptide M	POLR2M	0,98
nephronophthisis 3 (adolescent)	NPHP3	0,98
neuronal growth regulator 1	NEGR1	0,98
ADAM metallopeptidase with thrombospondin type 1 motif 8	ADAMTS8	0,98
lysine (K)-specific demethylase 5D	KDM5D	0,98
RNA polymerase II associated protein 1	RPAP1	0,98
NOP2/Sun domain family, member 6	NSUN6	0,98
chromosome 1 open reading frame 174	C1orf174	0,98
adrenoceptor alpha 2A	ADRA2A	0,98
proteasome subunit alpha 7	PSMA7	0,98
asunder spermatogenesis regulator	ASUN	0,98
SLIT and NTRK-like family, member 5	SLITRK5	0,98
family with sequence similarity 53, member B	FAM53B	0,98
uncharacterized LOC102467081; novel transcript, antisense to GZMK; Transcript Identified by AceView	LOC102467081; CTD-2313F11.1; slygee	0,98
transmembrane protein 168	TMEM168	0,98
zinc finger and BTB domain containing 26	ZBTB26	0,98
interleukin 18	IL18	0,98
kinesin-associated protein 3	KIFAP3	0,98
G protein-coupled receptor 21; RAB GTPase activating protein 1	GPR21; RABGAP1	0,98
loricrin	LOR	0,98
tripartite motif containing 4	TRIM4	0,98
arylacetamide deacetylase	AADAC	0,98
zinc finger and BTB domain containing 10	ZBTB10	0,98
platelet derived growth factor D	PDGFD	0,98
polycomb group ring finger 2	PCGF2	0,98

mitochondrial carrier 2	MTCH2	0,98
kelch repeat and BTB (POZ) domain containing 6	KBTBD6	0,98
zinc finger CCCH-type containing 12D	ZC3H12D	0,98
formin 1	FMN1	0,98
mal, T-cell differentiation protein-like	MALL	0,98
DPP9 antisense RNA 1	DPP9-AS1	0,98
telomeric repeat binding factor (NIMA-interacting) 1	TERF1	0,98
UTP11-like, U3 small nucleolar ribonucleoprotein (yeast)	UTP11L	0,98
chromosome 4 open reading frame 17	C4orf17	0,98
retinoic acid induced 14	RAI14	0,98
apolipoprotein B mRNA editing enzyme, catalytic polypeptide-like 3A	APOBEC3A	0,98
CKLF-like MARVEL transmembrane domain containing 6	CMTM6	0,98
myomesin 1	MYOM1	0,98
PLAC8-like 1	PLAC8L1	0,98
olfactory receptor, family 4, subfamily F, member 5	OR4F5	0,98
ARP1 actin-related protein 1 homolog A, centractin alpha (yeast)	ACTR1A	0,98
hexosaminidase (glycosyl hydrolase family 20, catalytic domain) containing	HEXDC	0,98
NOP56 ribonucleoprotein; small nucleolar RNA, C/D box 57; small nucleolar RNA, C/D box 56; small nucleolar RNA, H/ACA box 51; small nucleolar RNA, C/D box 110; small nucleolar RNA, C/D box 86; microRNA 1292	NOP56; SNORD57; SNORD56; SNORA51; SNORD110; SNORD86; MIR1292	0,98
IQ motif containing GTPase activating protein 2	IQGAP2	0,98
transmembrane protein 203	TMEM203	0,98
ST6 (alpha-N-acetyl-neuraminyl-2,3-beta-galactosyl-1,3)-N-acetylgalactosaminide alpha-2,6-sialyltransferase 4	ST6GALNAC4	0,98
WD repeat domain 33	WDR33	0,98
cathepsin G	CTSG	0,98
insulin like growth factor binding protein 4	IGFBP4	0,98
R3H domain containing 1	R3HDM1	0,98
guanylate binding protein 7	GBP7	0,98
ribosomal protein L13a; small nucleolar RNA, C/D box 35A; small nucleolar RNA, C/D box 34; small nucleolar RNA, C/D box 33; small nucleolar RNA, C/D box 32A	RPL13A; SNORD35A; SNORD34; SNORD33; SNORD32A	0,98
zinc finger CCCH-type containing 13	ZC3H13	0,98
K(lysine) acetyltransferase 6B	KAT6B	0,98
ST8 alpha-N-acetyl-neuraminide alpha-2,8-sialyltransferase 1	ST8SIA1	0,98
WAP four-disulfide core domain 3	WFDC3	0,98

nuclear transcription factor, X-box binding 1	NFX1	0,98
GRB10 interacting GYF protein 2	GIGYF2	0,98
retinol dehydrogenase 16 (all-trans)	RDH16	0,98
Memczak2013 ANTISENSE, coding, INTERNAL, intronic best transcript NM_001165136	HERC6	0,98
UDP-Gal:betaGlcNAc beta 1,4- galactosyltransferase, polypeptide 1	B4GALT1	0,98
cyclic nucleotide gated channel alpha 2	CNGA2	0,98
progestin and adipoQ receptor family member VII	PAQR7	0,98
arylsulfatase family, member J	ARSJ	0,98
hydroxymethylbilane synthase	HMBS	0,98
actin-like 7A	ACTL7A	0,98
farnesyl-diphosphate farnesyltransferase 1	FDFT1	0,98
ClpB homolog, mitochondrial AAA ATPase chaperonin	CLPB	0,98
pregnancy-zone protein	PZP	0,98
translocase of inner mitochondrial membrane 10 homolog (yeast)	TIMM10	0,98
zinc finger protein 146	ZNF146	0,98
protein phosphatase, Mg2+/Mn2+ dependent, 1B	PPM1B	0,98
TGFB1-induced anti-apoptotic factor 1; myosin XVIIIA	TIAF1; MYO18A	0,98
tudor domain containing 5	TDRD5	0,98
Memczak2013 ALT_ACCEPTOR, ALT_DONOR, coding, INTERNAL, intronic best transcript NM_001098797	TOX2	0,98
unkempt family zinc finger	UNK	0,98
Homo sapiens ribosomal protein L21, mRNA (cDNA clone MGC:2150 IMAGE:3543702), complete cds.; Homo sapiens ribosomal protein L21, mRNA (cDNA clone MGC:4136 IMAGE:2964441), complete cds.; Homo sapiens ribosomal protein L21, mRNA (cDNA clone MGC:71252 IMAGE:6084268), complete cds.; Homo sapiens ribosomal protein L21, mRNA (cDNA clone MGC:88165 IMAGE:4281405), complete cds.; Homo sapiens ribosomal protein L21, mRNA (cDNA clone MGC:88326 IMAGE:4248655), complete cds.; Homo sapiens ribosomal protein L21, mRNA (cDNA clone MGC:88334 IMAGE:3931616), complete cds.; Homo sapiens ribosomal protein L21, mRNA (cDNA clone MGC:88581 IMAGE:4901492), complete cds.; Homo sapiens ribosomal protein L21, mRNA (cDNA clone MGC:104275 IMAGE:6724053), complete cds.	RPL21	0,98
keratin associated protein 20-2	KRTAP20-2	0,98
YES proto-oncogene 1, Src family tyrosine kinase	YES1	0,98
POU class 2 homeobox 3	POU2F3	0,98
chromosome 6 open reading frame 222	C6orf222	0,98
N(alpha)-acetyltransferase 15, NatA auxiliary subunit	NAA15	0,98
EPH receptor B3	EPHB3	0,98

ENTH domain containing 1	ENTHD1	0,98
BLOC-1 related complex subunit 8; myocyte enhancer factor 2B; MEF2BNB-MEF2B readthrough	BORCS8; MEF2B; MEF2BNB-MEF2B	0,98
cancer/testis antigen family 45, member A8	CT45A8	0,98
cancer/testis antigen family 45, member A9	CT45A9	0,98
transforming growth factor, beta-induced, 68kDa	TGFBI	0,98
UMODL1 antisense RNA 1	UMODL1-AS1	0,98
ribosomal protein L39-like	RPL39L	0,98
BCL2-like 14 (apoptosis facilitator)	BCL2L14	0,98
guanine nucleotide binding protein (G protein), gamma transducing activity polypeptide 1	GNGT1	0,98
RNA binding motif protein 7	RBM7	0,98
small integral membrane protein 10 like 2B	SMIM10L2B	0,98
small ubiquitin-like modifier 1	SUMO1	0,98
solute carrier family 35, member F6	SLC35F6	0,98
BCL2-associated agonist of cell death	BAD	0,98
olfactory receptor, family 52, subfamily R, member 1 (gene/pseudogene)	OR52R1	0,98
rhomboid, veinlet-like 2 (Drosophila)	RHBDL2	0,98
ATPase, class VI, type 11C	ATP11C	0,98
cancer/testis antigen family 45, member A1	CT45A1	0,98
phosphatidylinositol glycan anchor biosynthesis class F	PIGF	0,98
isoleucyl-tRNA synthetase	IARS	0,98
coagulation factor VIII-associated 3	F8A3	0,98
ubiquitin-conjugating enzyme E2S	UBE2S	0,98
wingless-type MMTV integration site family, member 10A	WNT10A	0,98
adhesion G protein-coupled receptor E2	ADGRE2	0,98
peptidyl-tRNA hydrolase 2	PTRH2	0,98
transmembrane protein 265; Snf2-related CREBBP activator protein	TMEM265; SRCAP	0,98
X antigen family, member 1B; X antigen family, member 1E	XAGE1B; XAGE1E	0,98
major facilitator superfamily domain containing 2B	MFSD2B	0,98
angiotensin I converting enzyme	ACE	0,98
cAMP responsive element binding protein 1	CREB1	0,98
protein kinase, cAMP-dependent, catalytic, gamma	PRKACG	0,98
transmembrane protein 98	TMEM98	0,98
IKAROS family zinc finger 2	IKZF2	0,98
mucin 6, oligomeric mucus/gel-forming	MUC6	0,98
karyopherin alpha 4 (importin alpha 3)	KPNA4	0,98
solute carrier family 35, member B1	SLC35B1	0,98
RNA binding protein, fox-1 homolog (C. elegans) 2	RBFOX2	0,98
COP9 signalosome subunit 3	COPS3	0,98

solute carrier family 25 (mitochondrial carrier; adenine nucleotide translocator), member 6	SLC25A6	0,98
ELAV like neuron-specific RNA binding protein 3	ELAVL3	0,98
MEF2 activating motif and SAP domain containing transcriptional regulator	MAMSTR	0,98
calsyntenin 3	CLSTN3	0,98
major facilitator superfamily domain containing 11	MFSD11	0,98
zinc finger protein 16	ZNF16	0,98
small EDRK-rich factor 2	SERF2	0,98
p53-responsive gene 1	PRG1	0,98
mitochondrial ribosomal protein S5	MRPS5	0,98
enhancer of polycomb homolog 1 (Drosophila)	EPC1	0,98
MGA, MAX dimerization protein	MGA	0,98
ATP binding cassette subfamily G member 8	ABCG8	0,98
phosphatase and actin regulator 1	PHACTR1	0,98
deleted in azoospermia 3	DAZ3	0,98
CCHC-type zinc finger, nucleic acid binding protein	CNBP	0,98
ubiquitin-like modifier activating enzyme 3	UBA3	0,98
GDNF-inducible zinc finger protein 1	GZF1	0,98
SMAD specific E3 ubiquitin protein ligase 2	SMURF2	0,98
ArfGAP with GTPase domain, ankyrin repeat and PH domain 1	AGAP1	0,98
transmembrane protein 106B	TMEM106B	0,98
olfactory receptor, family 4, subfamily D, member 11	OR4D11	0,98
B-cell receptor-associated protein 31	BCAP31	0,98
polymerase (DNA directed), beta	POLB	0,98
MAM domain containing glycosylphosphatidylinositol anchor 2	MDGA2	0,98
2-hydroxyacyl-CoA lyase 1	HACL1	0,98
ATP binding cassette subfamily D member 2	ABCD2	0,98
ARP1 actin-related protein 1 homolog B, centractin beta (yeast)	ACTR1B	0,98
forkhead box L2	FOXL2	0,98
ADP-ribosylation factor interacting protein 2	ARFIP2	0,98
nudix hydrolase 19	NUDT19	0,98
MAM and LDL receptor class A domain containing 1	MALRD1	0,98
cyclin J-like	CCNJL	0,98
single-pass membrane protein with coiled-coil domains 4	SMCO4	0,98
myosin IB	MYO1B	0,98
solute carrier family 25 (mitochondrial carrier; dicarboxylate transporter), member 10	SLC25A10	0,98
triggering receptor expressed on myeloid cells-like 2	TREML2	0,98
neuronal differentiation 2	NEUROD2	0,98
twisted gastrulation BMP signaling modulator 1	TWSG1	0,98
tubulin tyrosine ligase	TTL	0,98
EF-hand domain (C-terminal) containing 1	EFHC1	0,98
ribosomal protein S10	RPS10	0,98
solute carrier family 20 (phosphate transporter), member 1	SLC20A1	0,98

interferon, alpha 6	IFNA6	0,98
phosphatidylethanolamine N-methyltransferase	PEMT	0,98
coiled-coil domain containing 127	CCDC127	0,98
acyl-CoA thioesterase 8	ACOT8	0,98
SKI family transcriptional corepressor 2	SKOR2	0,98
solute carrier family 6 (neutral amino acid transporter), member 15	SLC6A15	0,98
histone cluster 1, H3a	HIST1H3A	0,98
mitochondrial ribosomal protein S34	MRPS34	0,98
molybdenum cofactor synthesis 1	MOCS1	0,98
contactin 5	CNTN5	0,98
RNA pseudouridylate synthase domain containing 4	RPUSD4	0,98
acylaminoacyl-peptide hydrolase	APEH	0,98
zinc finger protein 831	ZNF831	0,98
tectonic family member 2	TCTN2	0,98
retinol dehydrogenase 13 (all-trans/9-cis)	RDH13	0,98
collagen, type IV, alpha 1	COL4A1	0,98
neurexophilin 3	NXPH3	0,98
retina and anterior neural fold homeobox 2	RAX2	0,98
ADP-ribosylation factor like GTPase 5A	ARL5A	0,98
REX4 homolog, 3'-5' exonuclease [Source:HGNC Symbol;Acc:HGNC:12820]	REXO4	0,98
zinc finger protein 571	ZNF571	0,98
calcium/calmodulin-dependent protein kinase ID	CAMK1D	0,98
glucosamine-phosphate N-acetyltransferase 1	GNPNAT1	0,98
B-cell receptor-associated protein 29	BCAP29	0,98
proline rich 20D; proline rich 20B	PRR20D; PRR20B	0,98
proline rich 20D; proline rich 20B	PRR20D; PRR20B	0,98
proline rich 20C; proline rich 20E	PRR20C; PRR20E	0,98
proline rich 20B; proline rich 20E; proline rich 20A; proline rich 20D	PRR20B; PRR20E; PRR20A; PRR20D	0,98
proline rich 20C; proline rich 20D	PRR20C; PRR20D	0,98
fat mass and obesity associated	FTO	0,98
prolylcarboxypeptidase	PRCP	0,98
chemokine (C-X-C motif) receptor 2	CXCR2	0,98
NADH dehydrogenase (ubiquinone) 1 beta subcomplex, 1, 7kDa	NDUFB1	0,98
syndecan 4	SDC4	0,98
potassium channel tetramerization domain containing 20	KCTD20	0,98
DEAD (Asp-Glu-Ala-Asp) box polypeptide 46	DDX46	0,98

signal-induced proliferation-associated 1 like 2	SIPA1L2	0,98
hyaluronan synthase 2	HAS2	0,98
tuftelin 1	TUFT1	0,98
talin 1; microRNA 6852	TLN1; MIR6852	0,98
centromere protein E	CENPE	0,98
potassium channel, inwardly rectifying subfamily J, member 12	KCNJ12	0,98
ATP binding cassette subfamily B member 8	ABCB8	0,98
TEA domain family member 1 (SV40 transcriptional enhancer factor)	TEAD1	0,98
polymerase (RNA) II (DNA directed) polypeptide L, 7.6kDa	POLR2L	0,98
homeobox B7	HOXB7	0,98
interferon, lambda 1	IFNL1	0,98
DIS3 homolog, exosome endoribonuclease and 3-5 exoribonuclease	DIS3	0,98
suppressor of IKBKE 1	SIKE1	0,98
SH3 domain binding kinase family, member 3	SBK3	0,98
butyrylcholinesterase	BCHE	0,98
NLR family, pyrin domain containing 10	NLRP10	0,98
relaxin/insulin-like family peptide receptor 2	RXFP2	0,98
testis specific protein, Y-linked 2	TSPY2	0,98
leucine rich repeat containing 8 family, member A	LRRC8A	0,98
solute carrier family 39 (zinc transporter), member 14	SLC39A14	0,98
kinase non-catalytic C-lobe domain (KIND) containing 1	KNDC1	0,98
RAD23 homolog B, nucleotide excision repair protein	RAD23B	0,98
SLIT and NTRK-like family, member 6	SLITRK6	0,98
deleted in azoospermia-like	DAZL	0,98
olfactory receptor, family 2, subfamily D, member 3	OR2D3	0,98
membrane-spanning 4-domains, subfamily A, member 6A	MS4A6A	0,98
mitochondrial ribosomal protein L24	MRPL24	0,98
F-box and leucine-rich repeat protein 5	FBXL5	0,98
insulin receptor substrate 2	IRS2	0,98
stromal interaction molecule 1; microRNA 4687	STIM1; MIR4687	0,98
VPS11, CORVET/HOPS core subunit [Source:HGNC Symbol;Acc:HGNC:14583]	VPS11	0,98
coiled-coil domain containing 71	CCDC71	0,98
glycoprotein Ib (platelet), alpha polypeptide	GP1BA	0,98
butyrophilin, subfamily 3, member A2	BTN3A2	0,98
ATPase, H+ transporting, lysosomal 14kDa, V1 subunit F	ATP6V1F	0,98
cell division cycle 123	CDC123	0,98
replication protein A2	RPA2	0,98
chemokine (C-X-C motif) ligand 13	CXCL13	0,98
tubby like protein 1	TULP1	0,98
eukaryotic translation initiation factor 4A3	EIF4A3	0,98
mannosyl-oligosaccharide glucosidase	MOGS	0,98

SMAD family member 1	SMAD1	0,98
sorting nexin 1	SNX1	0,98
POU class 2 homeobox 1	POU2F1	0,98
lysosomal protein transmembrane 4 alpha	LAPTM4A	0,98
histone cluster 1, H4i	HIST1H4I	0,98
cytochrome c oxidase subunit VIIc; microRNA 3607	COX7C; MIR3607	0,98
dyskeratosis congenita 1, dyskerin; small nucleolar RNA, H/ACA box 36A; small nucleolar RNA, H/ACA box 56; microRNA 664b	DKC1; SNORA36A; SNORA56; MIR664B	0,98
cholinergic receptor, muscarinic 5	CHRM5	0,98
death domain containing 1	DTHD1	0,98
POU6F2 antisense RNA 2 [Source:HGNC Symbol;Acc:HGNC:21887]; Salzman2013 ANTISENSE, coding, INTERNAL, intronic, OVERLAPTX best transcript NM_007252; novel transcript, antisense to POU6F2	POU6F2-AS2; POU6F2; AC011292.2	0,98
keratin associated protein 1-4	KRTAP1-4	0,98
G protein-coupled receptor 68	GPR68	0,98
complement component 8, beta polypeptide	C8B	0,98
sel-1 suppressor of lin-12-like 2 (C. elegans)	SEL1L2	0,98
Jeck2013 ALT_ACCEPTOR, ALT_DONOR, coding, INTERNAL, intronic best transcript NM_001160133	KCNQ5	0,98
transmembrane 4 L six family member 1	TM4SF1	0,98
SUMO1/sentrin specific peptidase 7	SENP7	0,98
CD3d molecule, delta (CD3-TCR complex)	CD3D	0,98
casein kinase 1, epsilon	CSNK1E	0,98
RAB8A, member RAS oncogene family	RAB8A	0,98
eukaryotic elongation factor 2 kinase	EEF2K	0,98
ectonucleoside triphosphate diphosphohydrolase 5	ENTPD5	0,98
ubiquitin specific peptidase 39	USP39	0,98
gamma-aminobutyric acid (GABA) A receptor, theta	GABRQ	0,98
olfactory receptor, family 56, subfamily A, member 3	OR56A3	0,98
COX18 cytochrome c oxidase assembly factor	COX18	0,98
cadherin 19, type 2	CDH19	0,98
kallikrein related peptidase 9	KLK9	0,98
solute carrier family 35 (UDP-galactose transporter), member A2	SLC35A2	0,98
methylmalonyl-CoA mutase	MUT	0,98
double C2-like domains, alpha	DOC2A	0,98
lysine (K)-specific demethylase 4B	KDM4B	0,98
signal transducer and activator of transcription 3 (acute-phase response factor)	STAT3	0,98
WAP four-disulfide core domain 12	WFDC12	0,98
granzyme K	GZMK	0,98

factor interacting with PAPOLA and CPSF1	FIP1L1	0,98
TBC/LysM-associated domain containing 1	TLDC1	0,98
eukaryotic translation elongation factor 1 epsilon 1	EEF1E1	0,98
hedgehog acyltransferase	HHAT	0,98
hydroxymethylbilane synthase	HMBS	0,98
spermidine/spermine N1-acetyltransferase 1	SAT1	0,98
superoxide dismutase 1, soluble	SOD1	0,98
abhydrolase domain containing 14A	ABHD14A	0,98
myotubularin related protein 2	MTMR2	0,98
DnaJ (Hsp40) homolog, subfamily C, member 8	DNAJC8	0,98
nucleolar protein 8	NOL8	0,98
aquaporin 1 (Colton blood group)	AQP1	0,98
zinc finger and BTB domain containing 5	ZBTB5	0,98
ankyrin repeat and KH domain containing 1; eukaryotic translation initiation factor 4E binding protein 3; ANKHD1-EIF4EBP3 readthrough	ANKHD1; EIF4EBP3; ANKHD1-EIF4EBP3	0,98
B-cell CLL/lymphoma 10	BCL10	0,98
anaphase promoting complex subunit 1	ANAPC1	0,98
zinc finger, BED-type containing 4	ZBED4	0,98
ribosomal protein S27a	RPS27A	0,98
chromosome 22 open reading frame 34	C22orf34	0,98
ribosomal protein S28	RPS28	0,98
chromosome 17 open reading frame 64	C17orf64	0,98
LysM, putative peptidoglycan-binding, domain containing 4	LYSMD4	0,98
NLR family, pyrin domain containing 14	NLRP14	0,98
fucosyltransferase 4 (alpha (1,3) fucosyltransferase, myeloid-specific)	FUT4	0,98
leucyl-tRNA synthetase 2, mitochondrial	LARS2	0,98
mitochondrial ribosomal protein S21	MRPS21	0,98
phosphatidylinositol 4-kinase, catalytic, alpha	PI4KA	0,98
leucine-rich repeat containing G protein-coupled receptor 6	LGR6	0,98
ATPase, class VI, type 11A	ATP11A	0,98
ATPase, Na+/K+ transporting, alpha 1 polypeptide	ATP1A1	0,98
microsomal glutathione S-transferase 1	MGST1	0,98
debranching RNA lariats 1	DBR1	0,98
ankyrin repeat domain 60	ANKRD60	0,98
methylmalonic aciduria (cobalamin deficiency) cblD type, with homocystinuria	MMADHC	0,98
zinc finger protein 286B; forkhead box O3B pseudogene	ZNF286B; FOXO3B	0,98
protein O-glucosyltransferase 1	POGLUT1	0,98
potassium channel, voltage gated shaker related subfamily A, member 4	KCNA4	0,98
N-acylsphingosine amidohydrolase (acid ceramidase) 1	ASAH1	0,98

solute carrier family 44 (choline transporter), member 2	SLC44A2	0,98
tRNA methyltransferase 5	TRMT5	0,98
ALG9, alpha-1,2-mannosyltransferase	ALG9	0,98
KIAA0319	KIAA0319	0,98
cytokine induced apoptosis inhibitor 1	CIAPIN1	0,98
nucleolar protein with MIF4G domain 1	NOM1	0,98
chromosome 6 open reading frame 25	C6orf25	0,98
salvador family WW domain containing protein 1	SAV1	0,98
coronin 6	CORO6	0,98
translocation associated membrane protein 2	TRAM2	0,98
nucleoporin 62kDa C-terminal like	NUP62CL	0,98
olfactory receptor, family 7, subfamily C, member 1	OR7C1	0,98
H2A histone family, member Y	H2AFY	0,98
pappalysin 2	PAPPA2	0,98
nephroblastoma overexpressed	NOV	0,98
forkhead box D4-like 3	FOXD4L3	0,98
cyclic nucleotide gated channel alpha 3	CNGA3	0,98
EF-hand calcium binding domain 8	EFCAB8	0,98
family with sequence similarity 217, member A	FAM217A	0,98
mitochondrial ribosomal protein L50	MRPL50	0,98
axin 2	AXIN2	0,98
osteosarcoma amplified 9, endoplasmic reticulum lectin	OS9	0,98
serine hydrolase-like (pseudogene)	SERHL	0,98
late cornified envelope 4A	LCE4A	0,98
TBC1 domain family, member 9B (with GRAM domain)	TBC1D9B	0,98
keratin 2, type II	KRT2	0,98
proteasome 26S subunit, non-ATPase 9	PSMD9	0,98
tumor protein D52	TPD52	0,98
tRNA aspartic acid methyltransferase 1	TRDMT1	0,98
ATP/GTP binding protein-like 4	AGBL4	0,98
NEDD8 activating enzyme E1 subunit 1	NAE1	0,98
cell cycle progression 1; microRNA 628	CCPG1; MIR628	0,98
adenosine kinase; mitochondrial ribosomal protein L35 pseudogene 3	ADK; MRPL35P3	0,98
tryptophan rich basic protein	WRB	0,98
canopy FGF signaling regulator 3	CNPY3	0,98
potassium channel, inwardly rectifying subfamily J, member 13	KCNJ13	0,98
cutA divalent cation tolerance homolog (E. coli)	CUTA	0,98
keratin 75, type II	KRT75	0,98
structural maintenance of chromosomes 2	SMC2	0,98
cholinergic receptor, nicotinic alpha 4	CHRNA4	0,98
non imprinted in Prader-Willi/Angelman syndrome 1	NIPA1	0,98
Ras and Rab interactor 2	RIN2	0,98
progestin and adipoQ receptor family member IX	PAQR9	0,98

solute carrier family 33 (acetyl-CoA transporter), member 1	SLC33A1	0,98
cytochrome b5 reductase-like	CYB5RL	0,98
5-hydroxytryptamine (serotonin) receptor 5A, G protein-coupled	HTR5A	0,98
glucagon-like peptide 2 receptor	GLP2R	0,98
transmembrane protein 178A	TMEM178A	0,98
sodium leak channel, non selective	NALCN	0,98
B-cell acute lymphoblastic leukemia expressed	BLACE	0,98
regulator of G-protein signaling 16	RGS16	0,98
major facilitator superfamily domain containing 3	MFSD3	0,98
quinoid dihydropteridine reductase	QDPR	0,98
transmembrane protein 123	TMEM123	0,98
opsin 1 (cone pigments), long-wave-sensitive	OPN1LW	0,98
tubulin, gamma complex associated protein 2	TUBGCP2	0,98
chemokine (C-X-C motif) ligand 9	CXCL9	0,98
inhibitor of growth family member 4	ING4	0,98
oligodendrocyte transcription factor 3	OLIG3	0,98
galactosidase beta 1 like 2	GLB1L2	0,98
wingless-type MMTV integration site family, member 7A	WNT7A	0,98
erythrocyte membrane protein band 4.1-like 1	EPB41L1	0,98
distal-less homeobox 1	DLX1	0,98
phosphoglycerate kinase 2	PGK2	0,98
pleckstrin homology domain containing, family F (with FYVE domain) member 2	PLEKHF2	0,98
cytosolic iron-sulfur assembly component 1	CIAO1	0,98
G protein-coupled receptor 149	GPR149	0,98
phenylalanine hydroxylase	PAH	0,98
serpin peptidase inhibitor, clade B (ovalbumin), member 6	SERPINB6	0,98
leukotriene A4 hydrolase	LTA4H	0,98
ALG12, alpha-1,6-mannosyltransferase	ALG12	0,98
nuclear factor of kappa light polypeptide gene enhancer in B-cells inhibitor, alpha	NFKBIA	0,98
serine/threonine kinase 38	STK38	0,98
uncharacterized LOC100129083; Transcript Identified by AceView, Entrez Gene ID(s) 100129083; novel transcript, antisense to SIGLEC10	LOC100129083; CTD-2616J11.2	0,98
tyrosinase-related protein 1	TYRP1	0,98
exocyst complex component 3-like 4	EXOC3L4	0,98
Memczak2013 ANTISENSE, coding, INTERNAL, intronic best transcript NM_080760	DACH1	0,98
glycosyltransferase like domain containing 1	GTDC1	0,98
ribosomal protein S6 kinase, 90kDa, polypeptide 1	RPS6KA1	0,98
cyclin-dependent kinase-like 1 (CDC2-related kinase)	CDKL1	0,98
centrosomal protein 170kDa	CEP170	0,98
hydroxyacylglutathione hydrolase	HAGH	0,98
drosha, ribonuclease type III	DROSHA	0,98

Memczak2013 ALT_ACCEPTOR, ALT_DONOR, coding, INTERNAL, intronic best transcript NM_001172639	CELF1	0,98
absent in melanoma 1-like	AIM1L	0,98
ARP5 actin-related protein 5 homolog (yeast)	ACTR5	0,98
homeobox C10	HOXC10	0,98
NIP7, nucleolar pre-rRNA processing protein	NIP7	0,98
basic, immunoglobulin-like variable motif containing	BIVM	0,98
putative homeodomain transcription factor 1	PHTF1	0,98
carbohydrate (N-acetylglucosamine 6-O) sulfotransferase 4	CHST4	0,98
testis expressed 261	TEX261	0,98
WNK lysine deficient protein kinase 3	WNK3	0,98
surfeit 1	SURF1	0,98
mucin 5B, oligomeric mucus/gel-forming	MUC5B	0,98
glucose 6 phosphatase, catalytic, 3	G6PC3	0,98
olfactory receptor, family 5, subfamily K, member 2	OR5K2	0,98
chromosome 5 open reading frame 28	C5orf28	0,98
vascular cell adhesion molecule 1	VCAM1	0,98
metallothionein 1B; metallothionein 1C, pseudogene	MT1B; MT1CP	0,98
WNK lysine deficient protein kinase 2	WNK2	0,98
zinc finger protein 266	ZNF266	0,98
mediator complex subunit 16	MED16	0,98
sarcosine dehydrogenase	SARDH	0,98
chloride intracellular channel 1	CLIC1	0,98
epithelial membrane protein 1	EMP1	0,98
SH3 and PX domains 2B	SH3PXD2B	0,98
acyloxyacyl hydrolase (neutrophil)	AOAH	0,98
family with sequence similarity 117, member A	FAM117A	0,98
abl-interactor 1	ABI1	0,98
armadillo repeat containing 1	ARMC1	0,98
StAR-related lipid transfer domain containing 9	STARD9	0,98
Jeck2013 ALT_ACCEPTOR, ALT_DONOR, coding, INTERNAL, intronic best transcript NM_004996	ABCC1	0,98
C-x(9)-C motif containing 4; mature T-cell proliferation 1	CMC4; MTCP1	0,98
Transcript Identified by AceView, Entrez Gene ID(s) 57545	CC2D2A	0,98
family with sequence similarity 86, member C1	FAM86C1	0,98
multimerin 1	MMRN1	0,98
ribosomal protein L26	RPL26	0,98
glutamate rich 6	ERICH6	0,98
PGAM family member 5, serine/threonine protein phosphatase, mitochondrial	PGAM5	0,98
ER membrane protein complex subunit 7	EMC7	0,98
choline dehydrogenase	CHDH	0,98
growth arrest-specific 2 like 3	GAS2L3	0,98
chromosome 19 open reading frame 12	C19orf12	0,98

ribosomal protein S8; small nucleolar RNA, C/D box 55; small nucleolar RNA, C/D box 46; small nucleolar RNA, C/D box 38A; small nucleolar RNA, C/D box 38B	RPS8; SNORD55; SNORD46; SNORD38A; SNORD38B	0,98
nuclear prelamin A recognition factor-like	NARFL	0,98
solute carrier family 18, subfamily B, member 1	SLC18B1	0,98
chemokine (C-X-C motif) ligand 6	CXCL6	0,98
ankyrin repeat and SOCS box containing 8	ASB8	0,98
family with sequence similarity 83, member F	FAM83F	0,98
aldehyde dehydrogenase 1 family, member A3	ALDH1A3	0,98
vascular endothelial growth factor B	VEGFB	0,98
Memczak2013 ALT_ACCEPTOR, ALT_DONOR, coding, INTERNAL, intronic best transcript NM_015130	TBC1D9	0,98
hemochromatosis	HFE	0,98
tectonin beta-propeller repeat containing 1	TECPR1	0,98
ribosomal protein L5; small nucleolar RNA, C/D box 21; small nucleolar RNA, H/ACA box 66	RPL5; SNORD21; SNORA66	0,98
ArfGAP with RhoGAP domain, ankyrin repeat and PH domain 1	ARAP1	0,98
kinesin family member 3A	KIF3A	0,98
fibrillarin	FBL	0,98
mortality factor 4 like 2	MORF4L2	0,98
laminin, beta 1	LAMB1	0,98
solute carrier family 16 (monocarboxylate transporter), member 1	SLC16A1	0,98
zinc finger and BTB domain containing 33	ZBTB33	0,98
CCR4-NOT transcription complex subunit 7	CNOT7	0,98
amyloid beta (A4) precursor protein-binding, family A, member 3	APBA3	0,98
zinc finger protein 793	ZNF793	0,98
N-terminal asparagine amidase	NTAN1	0,98
protein kinase N1	PKN1	0,98
TRAF2 and NCK interacting kinase	TNIK	0,98
ring finger protein 122	RNF122	0,98
interferon, epsilon; MIR31 host gene	IFNE; MIR31HG	0,98
zinc finger protein 302	ZNF302	0,98
v-rel avian reticuloendotheliosis viral oncogene homolog A	RELA	0,98
olfactory receptor, family 6, subfamily F, member 1; olfactory receptor, family 14, subfamily A, member 2	OR6F1; OR14A2	0,98
heterogeneous nuclear ribonucleoprotein A1 pseudogene 33; long intergenic non-protein coding RNA 842; annexin A8-like 1	HNRNPA1P33; LINC00842; ANXA8L1	0,98
Raf-1 proto-oncogene, serine/threonine kinase	RAF1	0,98

uncharacterized LOC339059; novel transcript, antisense to FAM38A	LOC339059; RP5-1142A6.8	0,98
aminoadipate-semialdehyde dehydrogenase	AASDH	0,98
runt-related transcription factor 3	RUNX3	0,98
leucine-rich repeats and immunoglobulin-like domains 1	LRIG1	0,98
DDB1 and CUL4 associated factor 5	DCAF5	0,98
solute carrier family 25, member 43	SLC25A43	0,98
5-aminoimidazole-4-carboxamide ribonucleotide formyltransferase/IMP cyclohydrolase	ATIC	0,98
pleiomorphic adenoma gene-like 1; hydatidiform mole associated and imprinted (non-protein coding)	PLAGL1; HYMAI	0,98
OTU deubiquitinase 4	OTUD4	0,98
Transcript Identified by AceView, Entrez Gene ID(s) 10363	HMG20A	0,98
amyloid beta (A4) precursor-like protein 1	APLP1	0,98
transient receptor potential cation channel, subfamily C, member 1	TRPC1	0,98
interphotoreceptor matrix proteoglycan 2	IMPG2	0,98
mitochondrial ribosomal protein L23	MRPL23	0,98
GPALPP motifs containing 1	GPALPP1	0,98
solute carrier family 48 (heme transporter), member 1	SLC48A1	0,98
mitochondrial elongation factor 2	MIEF2	0,98
histone cluster 1, H4g	HIST1H4G	0,98
discs, large (Drosophila) homolog-associated protein 4	DLGAP4	0,98
neurturin	NRTN	0,98
SAFB-like, transcription modulator	SLTM	0,98
cystatin B (stefin B)	CSTB	0,98
phosphorylase, glycogen, muscle	PYGM	0,98
RAB11A, member RAS oncogene family	RAB11A	0,98
serine palmitoyltransferase, small subunit A	SPTSSA	0,98
dynein, axonemal, light chain 4	DNAL4	0,98
LIM homeobox 3	LHX3	0,98
apolipoprotein B mRNA editing enzyme, catalytic polypeptide-like 3G	APOBEC3G	0,98
phosphotriesterase related	PTER	0,98
copine VII	CPNE7	0,98
limb and CNS expressed 1 like	LIX1L	0,98
nudix hydrolase 16	NUDT16	0,98
ribosomal protein L41 pseudogene 1	RPL41P1	0,98
troponin I type 2 (skeletal, fast)	TNNI2	0,98
glycoprotein hormone beta 5	GPHB5	0,98
v-maf avian musculoaponeurotic fibrosarcoma oncogene homolog F	MAFF	0,98
nephronophthisis 1 (juvenile)	NPHP1	0,98
bone morphogenetic protein/retinoic acid inducible neural-specific 3	BRINP3	0,98

golgin A8 family, member N; golgin A8 family, member H	GOLGA8N; GOLGA8H	0,98
zinc finger protein 479	ZNF479	0,98
fem-1 homolog b (C. elegans)	FEM1B	0,98
transmembrane protein 161B	TMEM161B	0,98
tripartite motif containing 49D2; tripartite motif containing 49D1	TRIM49D2; TRIM49D1	0,98
eukaryotic translation initiation factor 5A-like 1	EIF5AL1	0,98
ribosomal protein S24	RPS24	0,98
tumor protein p53 regulated apoptosis inducing protein 1	TP53AIP1	0,98
ribosomal protein S16	RPS16	0,98
Holliday junction recognition protein	HJURP	0,98
Jeck2013 ALT_ACCEPTOR, ALT_DONOR, coding, INTERNAL, intronic best transcript NM_133459	CCBE1	0,98
ring finger protein 214	RNF214	0,98
defender against cell death 1	DAD1	0,98
neuronal pentraxin receptor	NPTXR	0,98
tudor domain containing 6	TDRD6	0,98
RAS guanyl releasing protein 1 (calcium and DAG-regulated)	RASGRP1	0,98
leucine rich repeat containing 26	LRRC26	0,98
thioredoxin domain containing 9	TXNDC9	0,98
ubiquitin specific peptidase 15; microRNA 6125	USP15; MIR6125	0,98
potassium channel, voltage gated eag related subfamily H, member 1	KCNH1	0,98
intraflagellar transport 43	IFT43	0,98
Sec61 translocon beta subunit	SEC61B	0,98
chromosome 19 open reading frame 43	C19orf43	0,98
ring finger protein 168, E3 ubiquitin protein ligase	RNF168	0,98
potassium channel, inwardly rectifying subfamily J, member 18	KCNJ18	0,98
tumor suppressing subtransferable candidate 1	TSSC1	0,98
Memczak2013 ANTISENSE, CDS, coding, INTERNAL best transcript NM_005207	CRKL	0,98
DEAD (Asp-Glu-Ala-Asp) box polypeptide 53	DDX53	0,98
SMG1 phosphatidylinositol 3-kinase-related kinase	SMG1	0,98
transmembrane and immunoglobulin domain containing 2	TMIGD2	0,98
transmembrane protein 131	TMEM131	0,98
myeloid cell leukemia 1	MCL1	0,98
UHRF1 binding protein 1-like	UHRF1BP1L	0,98
methyl-CpG binding domain protein 1	MBD1	0,98
kynureninase	KYNU	0,98
phospholipid scramblase 1	PLSCR1	0,98
malic enzyme 3, NADP(+)-dependent, mitochondrial	ME3	0,98
charged multivesicular body protein 7	CHMP7	0,98
germ cell-less, spermatogenesis associated 1	GMCL1	0,98

SATB homeobox 2	SATB2	0,98
D-tyrosyl-tRNA deacylase 2 (putative)	DTD2	0,98
MRG/MORF4L binding protein	MRGBP	0,98
leucine rich repeat and sterile alpha motif containing 1	LRSAM1	0,98
dynein, axonemal, heavy chain 10	DNAH10	0,98
MAF1 homolog, negative regulator of RNA polymerase III	MAF1	0,98
epididymal protein 3B	EDDM3B	0,98
orosomucoid 1	ORM1	0,98
6-pyruvoyltetrahydropterin synthase	PTS	0,98
PDX1 C-terminal inhibiting factor 1	PCIF1	0,98
outer dense fiber of sperm tails 3-like 1	ODF3L1	0,98
Memczak2013 ALT_ACCEPTOR, ALT_DONOR, coding, INTERNAL, intronic best transcript NM_001202557	CD44	0,98
tumor necrosis factor receptor superfamily, member 12A	TNFRSF12A	0,98
ependymin related 1	EPDR1	0,98
cathepsin D	CTSD	0,98
PRAME family member 19	PRAMEF19	0,98
metallothionein-like 5, testis-specific (tesmin)	MTL5	0,98
BCL2-like 14 (apoptosis facilitator)	BCL2L14	0,98
keratin 6A, type II	KRT6A	0,98
Rho GTPase activating protein 11B	ARHGAP11B	0,98
SFT2 domain containing 1	SFT2D1	0,98
chromosome 15 open reading frame 57; mitochondrial ribosomal protein L42 pseudogene 5	C15orf57; MRPL42P5	0,98
outer dense fiber of sperm tails 4	ODF4	0,98
Memczak2013 ANTISENSE, coding, INTERNAL, intronic best transcript NM_014265	ADAM28	0,98
v-ral simian leukemia viral oncogene homolog B	RALB	0,98
small glutamine-rich tetratricopeptide repeat (TPR)-containing, beta	SGTB	0,98
dynein regulatory complex subunit 7	DRC7	0,98
shisa family member 6	SHISA6	0,98
zinc finger protein 730	ZNF730	0,98
inositol hexakisphosphate kinase 2	IP6K2	0,98
nuclear speckle splicing regulatory protein 1; microRNA 423	NSRP1; MIR423	0,98
v-myb avian myeloblastosis viral oncogene homolog-like 2	MYBL2	0,98
histone cluster 1, H4h	HIST1H4H	0,98
solute carrier family 39 (zinc transporter), member 4	SLC39A4	0,98
ADP-ribosylation factor like GTPase 3	ARL3	0,98
N-acetylated alpha-linked acidic dipeptidase 2	NAALAD2	0,98
ABL proto-oncogene 2, non-receptor tyrosine kinase	ABL2	0,98
insulin; INS-IGF2 readthrough	INS; INS-IGF2	0,98
perilipin 1	PLIN1	0,98

solute carrier family 17 (vesicular nucleotide transporter), member 9	SLC17A9	0,98
solute carrier family 39 (zinc transporter), member 12	SLC39A12	0,98
integrin alpha E	ITGAE	0,98
ATPase, Ca++ transporting, ubiquitous	ATP2A3	0,98
polycystic kidney disease 1 (autosomal dominant); microRNA 6511b-2; microRNA 6511b-1	PKD1; MIR6511B2; MIR6511B1	0,98
SPT20 homolog, SAGA complex component	SUPT20H	0,98
eukaryotic translation initiation factor 1A, X-linked	EIF1AX	0,98
hedgehog acyltransferase	HHAT	0,98
BUB1 mitotic checkpoint serine/threonine kinase B	BUB1B	0,98
fat storage-inducing transmembrane protein 2	FITM2	0,98
ribosomal RNA processing 7 homolog A	RRP7A	0,98
microfibrillar associated protein 3 like	MFAP3L	0,98
sphingomyelin phosphodiesterase 4, neutral membrane (neutral sphingomyelinase-3)	SMPD4	0,98
MPV17 mitochondrial membrane protein-like	MPV17L	0,98
family with sequence similarity 124 member A	FAM124A	0,98
glycoprotein VI (platelet)	GP6	0,98
poliovirus receptor-related 2 (herpesvirus entry mediator B)	PVRL2	0,98
chromosome 19 open reading frame 25	C19orf25	0,98
zinc finger protein 678	ZNF678	0,98
ribosomal RNA processing 12 homolog	RRP12	0,98
Cbp/p300-interacting transactivator, with Glu/Asp rich carboxy-terminal domain, 4	CITED4	0,98
family with sequence similarity 231, member B	FAM231B	0,98
zinc finger protein 223	ZNF223	0,98
WD repeat domain 66	WDR66	0,98
importin 13	IPO13	0,98
secretory carrier membrane protein 2	SCAMP2	0,98
protein tyrosine phosphatase, receptor type, f polypeptide (PTPRF), interacting protein (liprin), alpha 2	PPFIA2	0,98
protease, serine, 55	PRSS55	0,98
family with sequence similarity 32, member A	FAM32A	0,98
phospholipid phosphatase 3	PLPP3	0,98
chymotrypsinogen B1	CTRB1	0,98
tetratricopeptide repeat domain 31	TTC31	0,98
	LRIG1	0,98
notch 2 N-terminal like	NOTCH2NL	0,98
solute carrier family 47 (multidrug and toxin extrusion), member 1	SLC47A1	0,98
S-antigen; retina and pineal gland (arrestin)	SAG	0,98
SFT2 domain containing 2	SFT2D2	0,98
surfeit 4	SURF4	0,98

lysine (K)-specific demethylase 5A	KDM5A	0,98
mitochondrial ribosomal protein L35	MRPL35	0,98
protein arginine methyltransferase 1	PRMT1	0,98
S100 calcium binding protein A5	S100A5	0,98
ubiquitin related modifier 1	URM1	0,98
syndecan binding protein (syntenin) 2	SDCBP2	0,98
discoidin domain receptor tyrosine kinase 1; microRNA 4640	DDR1; MIR4640	0,98
radial spoke head 6 homolog A (Chlamydomonas)	RSPH6A	0,98
Jrk helix-turn-helix protein	JRK	0,98
tubby like protein 2	TULP2	0,98
ubiquitin specific peptidase 1	USP1	0,98
serine/threonine kinase 16	STK16	0,98
DCN1, defective in cullin neddylation 1, domain containing 5	DCUN1D5	0,98
meiotic nuclear divisions 1	MND1	0,98
RAS p21 protein activator 3	RASA3	0,98
chromosome X open reading frame 38	CXorf38	0,98
anaphase promoting complex subunit 16	ANAPC16	0,98
karyopherin alpha 1	KPNA1	0,98
F-box protein 45	FBXO45	0,98
WD repeat domain 46; microRNA 6873	WDR46; MIR6873	0,98
coxsackie virus and adenovirus receptor	CXADR	0,98
MAGE family member B18	MAGEB18	0,98
transient receptor potential cation channel, subfamily V, member 5	TRPV5	0,98
NADP-dependent oxidoreductase domain containing 1	NOXRED1	0,98
metallothionein 1I, pseudogene	MT1IP	0,98
glycine cleavage system protein H (aminomethyl carrier)	GCSH	0,98
prickle homolog 3	PRICKLE3	0,98
peroxiredoxin 2	PRDX2	0,98
Transcript Identified by AceView, Entrez Gene ID(s) 51633	OTUD6B	0,98
histone cluster 1, H1b	HIST1H1B	0,98
mbt domain containing 1	MBTD1	0,98
Transcript Identified by AceView, Entrez Gene ID(s) 116442	RAB39B	0,98
heat shock transcription factor 2	HSF2	0,98
N-terminal Xaa-Pro-Lys N-methyltransferase 1	NTMT1	0,98
secretory leukocyte peptidase inhibitor	SLPI	0,98
zinc finger protein 554	ZNF554	0,98
decapping enzyme, scavenger	DCPS	0,98
pyridoxamine 5-phosphate oxidase	PNPO	0,98
small ubiquitin-like modifier 2	SUMO2	0,98
leucine rich repeat and Ig domain containing 2	LINGO2	0,98
glutathione S-transferase mu 2 (muscle)	GSTM2	0,98
mitochondrial ribosomal protein L44	MRPL44	0,98

DNA-damage regulated autophagy modulator 2	DRAM2	0,98
SLIT and NTRK-like family, member 3	SLITRK3	0,98
adaptor-related protein complex 1, mu 1 subunit	AP1M1	0,98
PHD finger protein 3	PHF3	0,98
cyclin A1	CCNA1	0,98
absent in melanoma 1	AIM1	0,98
phosphatidylinositol-4-phosphate 3-kinase, catalytic subunit type 2 gamma	PIK3C2G	0,98
solute carrier family 35, member G3	SLC35G3	0,98
acyl-CoA synthetase long-chain family member 5	ACSL5	0,98
solute carrier family 45, member 1	SLC45A1	0,98
DNL-type zinc finger	DNLZ	0,98
serine/arginine-rich splicing factor 10	SRSF10	0,98
chromosome 17 open reading frame 62	C17orf62	0,98
angiopoietin like 6	ANGPTL6	0,98
MRE11 homolog A, double strand break repair nuclease	MRE11A	0,98
resistin	RETN	0,98
aldolase A, fructose-bisphosphate	ALDOA	0,97
integrator complex subunit 12	INTS12	0,97
lectin, mannose-binding 2	LMAN2	0,97
PIK3CD antisense RNA 2	PIK3CD-AS2	0,97
UL16 binding protein 3	ULBP3	0,97
serine palmitoyltransferase, small subunit B	SPTSSB	0,97
oxidase (cytochrome c) assembly 1-like	OXA1L	0,97
polymerase (RNA) II (DNA directed) polypeptide I, 14.5kDa	POLR2I	0,97
PR domain containing 5	PRDM5	0,97
HNRNPUL2-BSCL2 readthrough (NMD candidate)	HNRNPUL2-BSCL2	0,97
mitochondrial calcium uniporter	MCU	0,97
absent in melanoma 2	AIM2	0,97
septin 2	sept-02	0,97
RAB, member of RAS oncogene family-like 3	RABL3	0,97
nucleolin	NCL	0,97
proteasome (prosome, macropain) assembly chaperone 2	PSMG2	0,97
zinc finger, CCHC domain containing 7	ZCCHC7	0,97
ankyrin repeat domain 26	ANKRD26	0,97
pyroglutamylated RFamide peptide	QRFP	0,97
lysine (K)-specific methyltransferase 5A	KMT5A	0,97
UTP15, U3 small nucleolar ribonucleoprotein, homolog (S. cerevisiae)	UTP15	0,97
adenylate cyclase 7	ADCY7	0,97
heat shock factor binding protein 1-like 1	HSBP1L1	0,97
heat shock protein family B (small), member 11	HSPB11	0,97
CD48 molecule	CD48	0,97
extended synaptotagmin-like protein 2	ESYT2	0,97

acyl-CoA dehydrogenase, very long chain	ACADVL	0,97
dpy-19-like 1 (C. elegans)	DPY19L1	0,97
resistin like beta	RETNLB	0,97
ubiquitin specific peptidase 17-like family member 24; ubiquitin specific peptidase 17-like family member 26; ubiquitin specific peptidase 17-like family member 5; ubiquitin specific peptidase 17-like family member 27; ubiquitin specific peptidase 17-like family member 29; ubiquitin specific peptidase 17-like family member 30	USP17L24; USP17L26; USP17L5; USP17L27; USP17L29; USP17L30	0,97
ubiquitin specific peptidase 17-like family member 25; ubiquitin specific peptidase 17-like family member 27	USP17L25; USP17L27	0,97
protein kinase, cAMP-dependent, regulatory, type II, beta	PRKAR2B	0,97
neural proliferation, differentiation and control, 1	NPDC1	0,97
notch 1	NOTCH1	0,97
olfactory receptor family 4 subfamily K member 3 (gene/pseudogene) [Source:HGNC Symbol;Acc:HGNC:14731]	OR4K3	0,97
tetratricopeptide repeat domain 17	TTC17	0,97
chromosome 8 open reading frame 82; leucine rich repeat containing 24	C8orf82; LRRC24	0,97
methyltransferase like 20	METTL20	0,97
ribonucleotide reductase M1	RRM1	0,97
trans-2,3-enoyl-CoA reductase-like	TECRL	0,97
chromosome 19 open reading frame 67	C19orf67	0,97
VAMP (vesicle-associated membrane protein)-associated protein B and C	VAPB	0,97
transmembrane and immunoglobulin domain containing 1	TMIGD1	0,97
zinc finger protein 799	ZNF799	0,97
transmembrane protein 115	TMEM115	0,97
ADAM metallopeptidase with thrombospondin type 1 motif 13	ADAMTS13	0,97
family with sequence similarity 120A opposite strand	FAM120AOS	0,97
zinc finger and SCAN domain containing 30	ZSCAN30	0,97
NLR family, pyrin domain containing 5	NLRP5	0,97
deleted in lymphocytic leukemia 1 (non-protein coding)	DLEU1	0,97
eukaryotic translation initiation factor 4A2; small nucleolar RNA, H/ACA box 63; small nucleolar RNA, C/D box 2; small nucleolar RNA, H/ACA box 4; small nucleolar RNA, H/ACA box 81; microRNA 1248	EIF4A2; SNORA63; SNORD2; SNORA4; SNORA81; MIR1248	0,97
carboxypeptidase A1 (pancreatic)	CPA1	0,97
cadherin 12, type 2 (N-cadherin 2)	CDH12	0,97
transmembrane protein 8B	TMEM8B	0,97
angiotensin I converting enzyme 2	ACE2	0,97
huntingtin interacting protein 1 related	HIP1R	0,97
four and a half LIM domains 1	FHL1	0,97

pre-B lymphocyte 3	VPREB3	0,97
Transcript Identified by AceView, Entrez Gene ID(s) 7182	NR2C2	0,97
transmembrane protein 30A	TMEM30A	0,97
protein phosphatase 1, catalytic subunit, gamma isozyme	PPP1CC	0,97
KIAA1468	KIAA1468	0,97
potassium channel, voltage gated eag related subfamily H, member 5	KCNH5	0,97
synaptotagmin binding, cytoplasmic RNA interacting protein	SYNCRIP	0,97
zinc finger protein 593	ZNF593	0,97
UTP6, small subunit (SSU) processome component, homolog (yeast)	UTP6	0,97
protease, serine, 37	PRSS37	0,97
RNA binding motif protein 28	RBM28	0,97
ral guanine nucleotide dissociation stimulator-like 4	RGL4	0,97
RAB38, member RAS oncogene family	RAB38	0,97
karyopherin (importin) beta 1	KPNB1	0,97
WD repeat domain 4	WDR4	0,97
cell adhesion molecule 4	CADM4	0,97
olfactory receptor, family 52, subfamily K, member 2	OR52K2	0,97
WD repeat domain 35	WDR35	0,97
HEAT repeat containing 6	HEATR6	0,97
cholecystokinin	CCK	0,97
NADH dehydrogenase (ubiquinone) 1 alpha subcomplex, 10, 42kDa	NDUFA10	0,97
UTP18 small subunit (SSU) processome component	UTP18	0,97
high mobility group box 1	HMGB1	0,97
sirtuin 5	SIRT5	0,97
phosphate cytidylyltransferase 1, choline, alpha	PCYT1A	0,97
POU class 5 homeobox 1B	POU5F1B	0,97
long intergenic non-protein coding RNA 851	LINC00851	0,97
Fas (TNFRSF6)-associated via death domain	FADD	0,97
zinc finger protein 217	ZNF217	0,97
wingless-type MMTV integration site family, member 16	WNT16	0,97
polo-like kinase 5	PLK5	0,97
Rho family GTPase 3	RND3	0,97
novel transcript; Transcript Identified by AceView, Entrez Gene ID(s) 5690	RP4-728D4.2; PSMB2	0,97
villin 1	VIL1	0,97
ubiquitin specific peptidase 49	USP49	0,97
maestro heat-like repeat family member 1	MROH1	0,97
guanine monophosphate synthase	GMPS	0,97
regulator of G-protein signaling like 1	RGSL1	0,97
polymerase (DNA-directed), delta interacting protein 3	POLDIP3	0,97
unc-51 like autophagy activating kinase 1	ULK1	0,97
TBC1 domain family, member 3E	TBC1D3E	0,97

ubiquitin-like 4B	UBL4B	0,97
ribosomal protein L10a	RPL10A	0,97
excision repair cross-complementation group 4	ERCC4	0,97
claudin 25	CLDN25	0,97
complement component 4 binding protein, beta	C4BPB	0,97
collagen, type XXV, alpha 1	COL25A1	0,97
adenosine A2b receptor	ADORA2B	0,97
small integral membrane protein 6	SMIM6	0,97
PQ loop repeat containing 1	PQLC1	0,97
adenosylhomocysteinase-like 2	AHCYL2	0,97
methylphosphate capping enzyme	MEPCE	0,97
ATP synthase F0 subunit 8; ATP synthase F0 subunit 6; cytochrome c oxidase III	ATP8; ATP6; COX3	0,97
cilia and flagella associated protein 20	CFAP20	0,97
Fc receptor-like 5	FCRL5	0,97
heat shock factor binding protein 1	HSBP1	0,97
DNA (cytosine-5-)-methyltransferase 1	DNMT1	0,97
centrosomal protein 63kDa	CEP63	0,97
S100 calcium binding protein A1	S100A1	0,97
eomesodermin	EOMES	0,97
protein S (alpha)	PROS1	0,97
hepcidin antimicrobial peptide	HAMP	0,97
olfactory receptor, family 2, subfamily F, member 2	OR2F2	0,97
acyl-CoA oxidase 1, palmitoyl	ACOX1	0,97
ribulose-5-phosphate-3-epimerase-like 1	RPEL1	0,97
claudin 12	CLDN12	0,97
uncharacterized LOC654841; Salzman2013 ANTISENSE, CDS, coding, INTERNAL, intronic, OVCODE, OVERLAPTX, OVEXON best transcript NM_000091; novel transcript, antisense to COL4A3	LOC654841; AC097662.2; COL4A3	0,97
small proline-rich protein 1B	SPRR1B	0,97
lysozyme-like 1	LYZL1	0,97
zinc finger protein 462	ZNF462	0,97
coiled-coil domain containing 84	CCDC84	0,97
cyclin-dependent kinase 13	CDK13	0,97
unc-93 homolog B1 (C. elegans)	UNC93B1	0,97
mast cell-expressed membrane protein 1	MCEMP1	0,97
dopey family member 2	DOPEY2	0,97
ATP synthase, H+ transporting, mitochondrial Fo complex subunit F6	ATP5J	0,97
protein tyrosine phosphatase, non-receptor type 9	PTPN9	0,97
advillin	AVIL	0,97
DEAH (Asp-Glu-Ala-Asp/His) box polypeptide 57	DHX57	0,97
peroxiredoxin 1	PRDX1	0,97
cyclic nucleotide gated channel beta 1	CNGB1	0,97

peptidylprolyl isomerase F	PPIF	0,97
cleavage and polyadenylation specific factor 2	CPSF2	0,97
homeobox C12	HOXC12	0,97
solute carrier family 45, member 3	SLC45A3	0,97
methylcrotonoyl-CoA carboxylase 2	MCCC2	0,97
phospholipase C, gamma 1; ribosomal protein L23a pseudogene 81	PLCG1; RPL23AP81	0,97
protocadherin 18	PCDH18	0,97
PR domain containing 4	PRDM4	0,97
Fanconi anemia complementation group E	FANCE	0,97
telomere maintenance 2	TELO2	0,97
CDC42 effector protein (Rho GTPase binding) 3	CDC42EP3	0,97
protein phosphatase 1, regulatory subunit 3D	PPP1R3D	0,97
Memczak2013 ANTISENSE, CDS, coding, INTERNAL best transcript NM_001076552	ACSS2	0,97
dystrotelin	DYTN	0,97
lysophosphatidylcholine acyltransferase 4	LPCAT4	0,97
coiled-coil domain containing 34	CCDC34	0,97
collectin subfamily member 11	COLEC11	0,97
exostosin glycosyltransferase 1	EXT1	0,97
major histocompatibility complex, class I, A	HLA-A	0,97
BUB1 mitotic checkpoint serine/threonine kinase	BUB1	0,97
SIL1 nucleotide exchange factor	SIL1	0,97
zinc finger, CCHC domain containing 16	ZCCHC16	0,97
TGFB-induced factor homeobox 2	TGIF2	0,97
scavenger receptor class F, member 2	SCARF2	0,97
minichromosome maintenance complex component 7	MCM7	0,97
solute carrier family 5 (sodium/sugar cotransporter), member 10	SLC5A10	0,97
chromosome 10 open reading frame 95	C10orf95	0,97
junctional adhesion molecule 3	JAM3	0,97
peroxisome proliferator-activated receptor gamma, coactivator-related 1	PPRC1	0,97
nudix hydrolase 15	NUDT15	0,97
uncharacterized LOC149373; Transcript Identified by AceView; novel transcript	LOC149373; RP5-1097F14.3; kosimo	0,97
protocadherin 11 X-linked	PCDH11X	0,97
zinc finger protein 321, pseudogene	ZNF321P	0,97
adrenomedullin	ADM	0,97
solute carrier family 5 (sodium/myo-inositol cotransporter), member 3; mitochondrial ribosomal protein S6	SLC5A3; MRPS6	0,97
solute carrier family 51, beta subunit	SLC51B	0,97
lipoma HMGIC fusion partner-like 2	LHFPL2	0,97

premelanosome protein	PMEL	0,97
UDP glucuronosyltransferase 2 family, polypeptide B7	UGT2B7	0,97
interleukin 1 receptor accessory protein	IL1RAP	0,97
ADP-ribosylation factor like GTPase 6 interacting protein 5	ARL6IP5	0,97
chromosome 2 open reading frame 61	C2orf61	0,97
adhesion G protein-coupled receptor G4	ADGRG4	0,97
thrombospondin 1	THBS1	0,97
mitochondrial antiviral signaling protein	MAVS	0,97
FANCD2 opposite strand	FANCD2OS	0,97
H3 histone, family 3B (H3.3B)	H3F3B	0,97
calpain 14	CAPN14	0,97
phosphorylase kinase, gamma 2 (testis)	PHKG2	0,97
tigger transposable element derived 1; microRNA 5001	TIGD1; MIR5001	0,97
nibrin	NBN	0,97
zinc finger protein 780B	ZNF780B	0,97
chemokine (C-C motif) ligand 14; chemokine (C-C motif) ligand 15; CCL15-CCL14 readthrough (NMD candidate)	CCL14; CCL15; CCL15-CCL14	0,97
cilia and flagella associated 54	CFAP54	0,97
differentially expressed in FDCP 8 homolog (mouse)	DEF8	0,97
dynein, axonemal, heavy chain 1	DNAH1	0,97
N-acetyltransferase 10 (GCN5-related)	NAT10	0,97
regulator of G-protein signaling 1	RGS1	0,97
dispatched homolog 2 (Drosophila)	DISP2	0,97
K(lysine) acetyltransferase 6B	KAT6B	0,97
spectrin, alpha, non-erythrocytic 1	SPTAN1	0,97
NADH dehydrogenase (ubiquinone) 1 alpha subcomplex, 6, 14kDa	NDUFA6	0,97
LysM, putative peptidoglycan-binding, domain containing 2	LYSMD2	0,97
adaptor-related protein complex 3, mu 1 subunit	AP3M1	0,97
chromosome 6 open reading frame 226	C6orf226	0,97
mucin 1, cell surface associated	MUC1	0,97
chromosome 2 open reading frame 76	C2orf76	0,97
B-cell scaffold protein with ankyrin repeats 1	BANK1	0,97
mal, T-cell differentiation protein 2 (gene/pseudogene)	MAL2	0,97
transmembrane protein 37	TMEM37	0,97
scleraxis bHLH transcription factor	SCX	0,97
olfactory marker protein	OMP	0,97
insulin-like 5	INSL5	0,97
arrestin, beta 2	ARRB2	0,97
DIX domain containing 1	DIXDC1	0,97
mediator complex subunit 22	MED22	0,97
aftiphilin	AFTPH	0,97
erythroid differentiation regulatory factor 1	EDRF1	0,97
centrosomal protein 44kDa	CEP44	0,97

NADH dehydrogenase (ubiquinone) 1 beta subcomplex, 5, 16kDa	NDUFB5	0,97
ALG13, UDP-N-acetylglucosaminyltransferase subunit	ALG13	0,97
transmembrane protein 187	TMEM187	0,97
uncharacterized LOC101927844; Transcript Identified by AceView	LOC101927844; serfawbu	0,97
zinc finger protein 518A	ZNF518A	0,97
mannosidase, alpha, class 2C, member 1	MAN2C1	0,97
family with sequence similarity 69, member B	FAM69B	0,97
leucine zipper transcription factor like 1	LZTFL1	0,97
leucine twenty homeobox	LEUTX	0,97
RAB6A, member RAS oncogene family	RAB6A	0,97
zinc finger, DHHC-type containing 3	ZDHHC3	0,97
serine/threonine kinase 40	STK40	0,97
mutS homolog 6	MSH6	0,97
iroquois homeobox 1	IRX1	0,97
tetratricopeptide repeat domain 16	TTC16	0,97
CTD small phosphatase like	CTDSPL	0,97
forkhead box D4-like 4	FOXD4L4	0,97
coiled-coil domain containing 30	CCDC30	0,97
transmembrane protein 164	TMEM164	0,97
poly(ADP-ribose) polymerase family member 12	PARP12	0,97
histone cluster 1, H2ag	HIST1H2AG	0,97
UTP20 small subunit (SSU) processome component	UTP20	0,97
cerebral cavernous malformation 2-like	CCM2L	0,97
tripartite motif containing 49B	TRIM49B	0,97
solute carrier family 6 (neurotransmitter transporter), member 5	SLC6A5	0,97
limb bud and heart development	LBH	0,97
chordin	CHRD	0,97
nuclear factor of kappa light polypeptide gene enhancer in B-cells inhibitor-like 1	NFKBIL1	0,97
mesothelin	MSLN	0,97
ligand dependent nuclear receptor corepressor	LCOR	0,97
cytochrome b	CYTB	0,97
ribosomal protein L37	RPL37	0,97
CAP-GLY domain containing linker protein 2	CLIP2	0,97
ribosomal protein S7	RPS7	0,97
nuclear receptor subfamily 2, group F, member 2	NR2F2	0,97
zinc finger, DHHC-type containing 8	ZDHHC8	0,97
phosphoribosyl pyrophosphate synthetase-associated protein 1	PRPSAP1	0,97
FYN proto-oncogene, Src family tyrosine kinase	FYN	0,97
solute carrier family 5 (sodium/monocarboxylate cotransporter), member 12	SLC5A12	0,97
zinc finger protein 728	ZNF728	0,97
transmembrane protein 2	TMEM2	0,97

CCZ1 homolog B, vacuolar protein trafficking and biogenesis associated	CCZ1B	0,97
FYVE, RhoGEF and PH domain containing 2	FGD2	0,97
zona pellucida-like domain containing 1	ZPLD1	0,97
protein phosphatase 2, regulatory subunit B, beta	PPP2R5B	0,97
homeobox D11	HOXD11	0,97
sema domain, immunoglobulin domain (Ig), short basic domain, secreted, (semaphorin) 3D	SEMA3D	0,97
tumor protein p53 inducible nuclear protein 1	TP53INP1	0,97
membrane associated guanylate kinase, WW and PDZ domain containing 2	MAGI2	0,97
tudor domain containing 12	TDRD12	0,97
pyruvate dehydrogenase kinase, isozyme 2	PDK2	0,97
autism susceptibility candidate 2	AUTS2	0,97
zinc finger protein 510	ZNF510	0,97
C-type lectin domain family 2, member A	CLEC2A	0,97
bromodomain containing 7	BRD7	0,97
transducin-like enhancer of split 1 (E(sp1) homolog, Drosophila)	TLE1	0,97
zinc finger RNA binding protein 2	ZFR2	0,97
microcephalin 1	MCPH1	0,97
toll-like receptor 9	TLR9	0,97
zinc finger protein 219	ZNF219	0,97
FGFR1 oncogene partner	FGFR1OP	0,97
SYS1-DBNDD2 readthrough (NMD candidate)	SYS1-DBNDD2	0,97
solute carrier family 35 (UDP-N-acetylglucosamine (UDP-GlcNAc) transporter), member A3	SLC35A3	0,97
gelsolin	GSN	0,97
X-ray repair complementing defective repair in Chinese hamster cells 4	XRCC4	0,97
zinc finger protein 474-like	LOC100505841	0,97
plexin A2	PLXNA2	0,97
kelch-like family member 5	KLHL5	0,97
olfactory receptor, family 5, subfamily AL, member 1 (gene/pseudogene)	OR5AL1	0,97
NCK-associated protein 5	NCKAP5	0,97
RAB25, member RAS oncogene family	RAB25	0,97
coagulation factor VIII-associated 2; coagulation factor VIII-associated 3; coagulation factor VIII-associated 1	F8A2; F8A3; F8A1	0,97
eukaryotic translation initiation factor 3, subunit A	EIF3A	0,97
coronin, actin binding protein, 1B	CORO1B	0,97
tropomyosin 3	TPM3	0,97
zinc finger protein 813	ZNF813	0,97
DEP domain containing 1B	DEPDC1B	0,97
gamma-aminobutyric acid (GABA) A receptor, delta	GABRD	0,97
ubiquitin conjugating enzyme E2T	UBE2T	0,97

transducin-like enhancer of split 6	TLE6	0,97
polymerase (DNA-directed), delta 4, accessory subunit	POLD4	0,97
MIR205 host gene; microRNA 205	MIR205HG; MIR205	0,97
zinc finger protein 668	ZNF668	0,97
sarcolipin	SLN	0,97
M-phase phosphoprotein 10 (U3 small nucleolar ribonucleoprotein)	MPHOSPH10	0,97
heterogeneous nuclear ribonucleoprotein D	HNRNPD	0,97
nuclear pore complex interacting protein family, member A5	NPIPA5	0,97
ADP-ribosylation factor interacting protein 1	ARFIP1	0,97
antigen p97 (melanoma associated) identified by monoclonal antibodies 133.2 and 96.5	MFI2	0,97
2-oxoglutarate and iron-dependent oxygenase domain containing 1	OGFOD1	0,97
ALG2, alpha-1,3/1,6-mannosyltransferase	ALG2	0,97
Down syndrome critical region 4	DSCR4	0,97
esterase D	ESD	0,97
chromosome 21 open reading frame 59	C21orf59	0,97
ARP3 actin-related protein 3 homolog (yeast)	ACTR3	0,97
diacylglycerol kinase, kappa	DGKK	0,97
olfactory receptor, family 5, subfamily I, member 1	OR5I1	0,97
DEAD (Asp-Glu-Ala-Asp) box polypeptide 51	DDX51	0,97
wingless-type MMTV integration site family, member 11	WNT11	0,97
histone cluster 1, H1e	HIST1H1E	0,97
Transcript Identified by AceView, Entrez Gene ID(s) 5987	TRIM27	0,97
family with sequence similarity 177, member A1	FAM177A1	0,97
transmembrane p24 trafficking protein 5	TMED5	0,97
SID1 transmembrane family, member 2	SIDT2	0,97
synaptotagmin-like 5	SYTL5	0,97
hemogen	HEMGN	0,97
hypoxia up-regulated 1	HYOU1	0,97
gametocyte specific factor 1-like	GTSF1L	0,97
iodotyrosine deiodinase	IYD	0,97
transcription elongation factor B (SIII), polypeptide 3 (110kDa, elongin A)	TCEB3	0,97
peroxisomal biogenesis factor 13	PEX13	0,97
cilia and flagella associated protein 53	CFAP53	0,97
mitochondrial ribosomal protein L30	MRPL30	0,97
H3 histone, family 3C	H3F3C	0,97
fatty acid desaturase 1; microRNA 1908	FADS1; MIR1908	0,97
methyl-CpG binding domain protein 6	MBD6	0,97
early B-cell factor 2	EBF2	0,97
KIAA0125	KIAA0125	0,97

interleukin 12A	IL12A	0,97
COX11 cytochrome c oxidase copper chaperone	COX11	0,97
Tax1 (human T-cell leukemia virus type I) binding protein 1	TAX1BP1	0,97
dicarbonyl/L-xylulose reductase	DCXR	0,97
purinergic receptor P2X, ligand gated ion channel, 3	P2RX3	0,97
kidney associated antigen 1	KAAG1	0,97
mitogen-activated protein kinase kinase kinase kinase 3	MAP4K3	0,97
uncharacterized LOC339862; novel transcript; Transcript Identified by AceView	LOC339862; RP11-320G10.1; skerdorbo	0,97
transcription termination factor, RNA polymerase I	TTF1	0,97
DnaJ (Hsp40) homolog, subfamily C, member 16	DNAJC16	0,97
mitogen-activated protein kinase kinase 1	MAP2K1	0,97
Memczak2013 ANTISENSE, CDS, coding, INTERNAL best transcript NM_001020819	MYADM	0,97
EPS8-like 2	EPS8L2	0,97
fibrinogen alpha chain	FGA	0,97
immediate early response 3 interacting protein 1	IER3IP1	0,97
proteasome (prosome, macropain) subunit, beta type, 11	PSMB11	0,97
pyroglutamylated RFamide peptide receptor	QRFPR	0,97
transmembrane and ubiquitin-like domain containing 1	TMUB1	0,97
zinc finger, DHHC-type containing 3	ZDHHC3	0,97
triggering receptor expressed on myeloid cells-like 1	TREML1	0,97
Transcript Identified by AceView, Entrez Gene ID(s) 50515	CHST11	0,97
Memczak2013 ALT_ACCEPTOR, ALT_DONOR, coding, INTERNAL, intronic best transcript NM_032493	AP1M1	0,97
rhotekin 2	RTKN2	0,97
chromosome 11 open reading frame 88	C11orf88	0,97
conserved helix-loop-helix ubiquitous kinase	CHUK	0,97
fibroblast growth factor receptor 1	FGFR1	0,97
family with sequence similarity 205, member A	FAM205A	0,97
platelet-activating factor acetylhydrolase 1b, catalytic subunit 3 (29kDa)	PAFAH1B3	0,97
tumor necrosis factor, alpha-induced protein 3	TNFAIP3	0,97
major facilitator superfamily domain containing 7	MFSD7	0,97
synapse defective 1, Rho GTPase, homolog 2 (C. elegans)	SYDE2	0,97
Homo sapiens family with sequence similarity 231, member A (FAM231A), mRNA.; Homo sapiens family with sequence similarity 231, member C (FAM231C), mRNA.; Protein FAM231A/C [Source:UniProtKB/Swiss-Prot;Acc:A6NEA5]	FAM231A; FAM231C; RP5-1182A14.6	0,97
zinc finger protein 502	ZNF502	0,97
zinc finger protein 567	ZNF567	0,97
olfactory receptor, family 2, subfamily B, member 2	OR2B2	0,97
UDP glucuronosyltransferase 2 family, polypeptide B11	UGT2B11	0,97

partner and localizer of BRCA2	PALB2	0,97
synaptotagmin XIII	SYT13	0,97
nucleoporin 88kDa	NUP88	0,97
ATP synthase, H+ transporting, mitochondrial Fo complex subunit C1 (subunit 9)	ATP5G1	0,97
leucine rich repeat containing 3B	LRRC3B	0,97
family with sequence similarity 198, member A	FAM198A	0,97
H1 histone family, member N, testis-specific	H1FNT	0,97
DMRT-like family A2	DMRTA2	0,97
zinc finger E-box binding homeobox 2	ZEB2	0,97
NK6 homeobox 2	NKX6-2	0,97
decapping mRNA 1A	DCP1A	0,97
SRY box 13	SOX13	0,97
collagen, type XIX, alpha 1	COL19A1	0,97
intraflagellar transport 140	IFT140	0,97
UBX domain protein 4	UBXN4	0,97
Zhang2013 ALT_ACCEPTOR, ALT_DONOR, coding, INTERNAL, intronic best transcript NM_003086	SNAPC4	0,97
lactamase, beta-like 1	LACTBL1	0,97
zinc finger, HIT-type containing 3	ZNHIT3	0,97
protein arginine methyltransferase 2	PRMT2	0,97
solute carrier family 15, member 5	SLC15A5	0,97
structural maintenance of chromosomes 5	SMC5	0,97
RDH10 antisense RNA 1	RDH10-AS1	0,97
tubulin, alpha 3d	TUBA3D	0,97
CDP-diacylglycerol synthase 2	CDS2	0,97
Transcript Identified by AceView; Memczak2013 ANTISENSE, CDS, coding, INTERNAL, intronic, UTR5 best transcript NM_018284	flawmawbo; GBP3	0,97
tau tubulin kinase 2	TTBK2	0,97
EYA transcriptional coactivator and phosphatase 4	EYA4	0,97
potassium channel tetramerization domain containing 8	KCTD8	0,97
OTU deubiquitinase 1	OTUD1	0,97
dipeptidyl-peptidase 8	DPP8	0,97
solute carrier family 22, member 17	SLC22A17	0,97
INO80 complex subunit C	INO80C	0,97
transmembrane protein 106A	TMEM106A	0,97
phosphoprotein membrane anchor with glycosphingolipid microdomains 1	PAG1	0,97
CD300 molecule-like family member b	CD300LB	0,97
ubiquitin associated protein 1 like	UBAP1L	0,97
RNA binding motif protein 8A	RBM8A	0,97
chromosome 20 open reading frame 78	C20orf78	0,97
phospholipase A2, group IIE	PLA2G2E	0,97

nuclear factor of kappa light polypeptide gene enhancer in B-cells inhibitor, epsilon	NFKBIE	0,97
MCF.2 cell line derived transforming sequence like	MCF2L	0,97
cyclin K	CCNK	0,97
creatine kinase, muscle	CKM	0,97
Rho GTPase activating protein 35	ARHGAP35	0,97
coiled-coil domain containing 50	CCDC50	0,97
phenylalanyl-tRNA synthetase beta subunit	FARSB	0,97
spectrin, beta, erythrocytic; microRNA 7855	SPTB; MIR7855	0,97
chromosome 12 open reading frame 66	C12orf66	0,97
zinc finger protein 232	ZNF232	0,97
ATP11A upstream neighbor	ATP11AUN	0,97
myocyte enhancer factor 2C	MEF2C	0,97
amelotin	AMTN	0,97
chemokine (C-C motif) ligand 26	CCL26	0,97
NADH dehydrogenase (ubiquinone) 1 alpha subcomplex, 9, 39kDa	NDUFA9	0,97
long intergenic non-protein coding RNA 504	LINC00504	0,97
ATPase family, AAA domain containing 3A	ATAD3A	0,97
heart and neural crest derivatives expressed 2	HAND2	0,97
serine palmitoyltransferase, long chain base subunit 3	SPTLC3	0,97
RING1 and YY1 binding protein	RYBP	0,97
ubiquitin protein ligase E3D	UBE3D	0,97
mitochondrial ribosomal protein S18A	MRPS18A	0,97
CCR4-NOT transcription complex subunit 6	CNOT6	0,97
synovial sarcoma, X breakpoint 7	SSX7	0,97
cytochrome P450, family 4, subfamily Z, polypeptide 1	CYP4Z1	0,97
stomatin	STOM	0,97
NADH dehydrogenase (ubiquinone) 1 alpha subcomplex, 8, 19kDa	NDUFA8	0,97
programmed cell death 6 interacting protein	PDCD6IP	0,97
zinc finger protein 383	ZNF383	0,97
glycosylphosphatidylinositol specific phospholipase D1	GPLD1	0,97
CWC22 homolog, spliceosome-associated protein	CWC22	0,97
translocase of outer mitochondrial membrane 7 homolog (yeast)	TOMM7	0,97
TOPBP1-interacting checkpoint and replication regulator	TICRR	0,97
pre-mRNA processing factor 6	PRPF6	0,97
ring finger protein 5, E3 ubiquitin protein ligase	RNF5	0,97
KH-type splicing regulatory protein; microRNA 3940	KHSRP; MIR3940	0,97
SWI/SNF related, matrix associated, actin dependent regulator of chromatin, subfamily c, member 1	SMARCC1	0,97
myeloid/lymphoid or mixed-lineage leukemia; translocated to, 1	MLLT1	0,97
pyrroline-5-carboxylate reductase 1	PYCR1	0,97

solute carrier family 16 (monocarboxylate transporter), member 1	SLC16A1	0,97
apolipoprotein O	APOO	0,97
methionyl-tRNA synthetase 2, mitochondrial	MARS2	0,97
phosphoglucomutase 2	PGM2	0,97
structural maintenance of chromosomes 3	SMC3	0,97
NADPH oxidase activator 1	NOXA1	0,97
kinesin family member 15	KIF15	0,97
replication initiator 1	REPIN1	0,97
olfactory receptor, family 10, subfamily K, member 1	OR10K1	0,97
Memczak2013 ANTISENSE, CDS, coding, INTERNAL, UTR3 best transcript NM_001127457	CRY2	0,97
leucyl/cystinyl aminopeptidase	LNPEP	0,97
RAD51 paralog C	RAD51C	0,97
atrophin 1	ATN1	0,97
ribosomal protein L9	RPL9	0,97
anaphase promoting complex subunit 13	ANAPC13	0,97
fibrillarin	FBL	0,97
calcitonin-related polypeptide beta	CALCB	0,97
ribosomal protein L37a	RPL37A	0,97
DCN1, defective in cullin neddylation 1, domain containing 3	DCUN1D3	0,97
mitochondrial ribosomal protein S30	MRPS30	0,97
progestin and adipoQ receptor family member VIII	PAQR8	0,97
exportin 1	XPO1	0,97
WDFY family member 4	WDFY4	0,97
Transcript Identified by AceView, Entrez Gene ID(s) 3775	KCNK1	0,97
voltage-dependent anion channel 3	VDAC3	0,97
sorting nexin 6	SNX6	0,97
collagen, type VII, alpha 1; microRNA 711	COL7A1; MIR711	0,97
cilia and flagella associated protein 65	CFAP65	0,97
phospholipase A2, group XIIA	PLA2G12A	0,97
glycerol-3-phosphate acyltransferase 2, mitochondrial	GPAT2	0,97
myeloid-associated differentiation marker	MYADM	0,97
lymphocyte-specific protein 1; microRNA 7847	LSP1; MIR7847	0,97
secretory carrier membrane protein 1	SCAMP1	0,97
zinc finger protein 253	ZNF253	0,97
transmembrane protein 94; microRNA 6785	TMEM94; MIR6785	0,97
glutamate dehydrogenase 1	GLUD1	0,97
chaperonin containing TCP1, subunit 6B (zeta 2)	CCT6B	0,97
transmembrane protein 241	TMEM241	0,97
hyaluronoglucosaminidase 3	HYAL3	0,97
olfactory receptor, family 51, subfamily D, member 1	OR51D1	0,97
destrin (actin depolymerizing factor)	DSTN	0,97

cell division cycle 42	CDC42	0,97
neurofibromin 2 (merlin)	NF2	0,97
Kruppel-like factor 17	KLF17	0,97
transmembrane protein 11	TMEM11	0,97
vacuolar protein sorting 54 homolog (S. cerevisiae)	VPS54	0,97
cytohesin 1	CYTH1	0,97
doublecortin domain containing 2B	DCDC2B	0,97
malate dehydrogenase 2	MDH2	0,97
keratin associated protein 4-6	KRTAP4-6	0,97
zinc finger protein 701; zinc finger protein 137, pseudogene	ZNF701; ZNF137P	0,97
meiotic double-stranded break formation protein 4	MEI4	0,97
family with sequence similarity 104, member A	FAM104A	0,97
zinc finger protein 112	ZNF112	0,97
olfactory receptor, family 2, subfamily W, member 3	OR2W3	0,97
nudix hydrolase 4	NUDT4	0,97
coiled-coil domain containing 138	CCDC138	0,97
chromosome 1 open reading frame 109	C1orf109	0,97
DnaJ (Hsp40) homolog, subfamily A, member 4	DNAJA4	0,97
mechanistic target of rapamycin (serine/threonine kinase)	MTOR	0,97
activin A receptor type IIB	ACVR2B	0,97
tRNA methyltransferase 61B	TRMT61B	0,97
tubulin, gamma 1	TUBG1	0,97
chromosome 4 open reading frame 27	C4orf27	0,97
tyrosine 3-monooxygenase/tryptophan 5-monooxygenase activation protein, zeta	YWHAZ	0,97
stearoyl-CoA desaturase 5	SCD5	0,97
eukaryotic translation initiation factor 4 gamma, 2; small nucleolar RNA, C/D box 97	EIF4G2; SNORD97	0,97
proteasome 26S subunit, non-ATPase 8	PSMD8	0,97
KH domain containing, RNA binding, signal transduction associated 3	KHDRBS3	0,97
sorting nexin 33	SNX33	0,97
protein inhibitor of activated STAT 2	PIAS2	0,97
proteasome 26S subunit, ATPase 4 [Source:HGNC Symbol;Acc:HGNC:9551]	PSMC4	0,97
alkaline phosphatase, liver/bone/kidney	ALPL	0,97
tetraspanin 8	TSPAN8	0,97
plakophilin 4	PKP4	0,97
SHC SH2-domain binding protein 1-like	SHCBP1L	0,97
apolipoprotein C-I	APOC1	0,97
growth arrest-specific 1	GAS1	0,97
NADH dehydrogenase (ubiquinone) 1 alpha subcomplex, 1, 7.5kDa	NDUFA1	0,97
lipase, hepatic	LIPC	0,97

Synthetic construct Homo sapiens clone IMAGE:100063377, MGC:190767 ribosomal protein L41 (RPL41) mRNA, encodes complete protein.; 60S ribosomal protein L41 [Source:UniProtKB/Swiss-Prot;Acc:P62945]	RPL41	0,97
cystin 1	CYS1	0,97
hemoglobin, beta	HBB	0,97
testis specific protein, Y-linked 10	TSPY10	0,97
tripartite motif containing 33	TRIM33	0,97
leukotriene C4 synthase	LTC4S	0,97
apolipoprotein A-I	APOA1	0,97
major histocompatibility complex, class I, G	HLA-G	0,97
Transcript Identified by AceView, Entrez Gene ID(s) 118424	UBE2J2	0,97
protocadherin beta 9	PCDHB9	0,97
ADAM metallopeptidase domain 32	ADAM32	0,97
transmembrane protein 184C	TMEM184C	0,97
Fer3-like bHLH transcription factor	FERD3L	0,97
RNA binding motif protein 42	RBM42	0,97
SEC63 homolog, protein translocation regulator	SEC63	0,97
mitochondrial ribosomal protein L27	MRPL27	0,97
corticotropin releasing hormone	CRH	0,97
deltex 2, E3 ubiquitin ligase	DTX2	0,97
dynein, axonemal, assembly factor 2	DNAAF2	0,97
amidohydrolase domain containing 1	AMDHD1	0,97
KH and NYN domain containing	KHNYN	0,97
Rho guanine nucleotide exchange factor 37	ARHGEF37	0,97
urotensin 2	UTS2	0,97
corticotropin releasing hormone binding protein	CRHBP	0,97
EGF containing fibulin-like extracellular matrix protein 1	EFEMP1	0,97
myelin protein zero-like 1	MPZL1	0,97
SMAD family member 9	SMAD9	0,97
VPS29 retromer complex component	VPS29	0,97
3-hydroxy-3-methylglutaryl-CoA synthase 1 (soluble)	HMGCS1	0,97
purine nucleoside phosphorylase	PNP	0,97
cancer/testis antigen 62	CT62	0,97
NudC domain containing 1	NUDCD1	0,97
claudin domain containing 1	CLDND1	0,97
KRAB box domain containing 1	KRBOX1	0,97
jun D proto-oncogene	JUND	0,97
family with sequence similarity 26, member F	FAM26F	0,97
KH homology domain containing 1	KHDC1	0,97
acyl-CoA dehydrogenase, C-2 to C-3 short chain	ACADS	0,97
transmembrane p24 trafficking protein 10	TMED10	0,97
poliovirus receptor related immunoglobulin domain containing	PVRIG	0,97
trans-golgi network protein 2	TGOLN2	0,97
chromosome 2 open reading frame 49	C2orf49	0,97

ELOVL fatty acid elongase 1; microRNA 6734	ELOVL1; MIR6734	0,97
kelch repeat and BTB (POZ) domain containing 4	KBTBD4	0,97
autophagy related 16-like 1	ATG16L1	0,97
RNA binding motif protein 4B	RBM4B	0,97
gamma-aminobutyric acid (GABA) A receptor, gamma 3	GABRG3	0,97
mitogen-activated protein kinase kinase kinase 19	MAP3K19	0,97
retinal outer segment membrane protein 1	ROM1	0,97
olfactory receptor, family 4, subfamily K, member 1	OR4K1	0,97
glycosylated lysosomal membrane protein	GLMP	0,97
mitochondrial ribosomal protein L36	MRPL36	0,97
transmembrane protein 249	TMEM249	0,97
retbindin	RTBDN	0,97
proline-rich protein HaeIII subfamily 2	PRH2	0,97
zinc finger protein 428	ZNF428	0,97
S-antigen; retina and pineal gland (arrestin)	SAG	0,97
gasdermin B	GSDMB	0,97
chromosome 15 open reading frame 53	C15orf53	0,97
cytochrome P450, family 2, subfamily B, polypeptide 6	CYP2B6	0,97
leucine rich repeat containing 73	LRRC73	0,97
DENN/MADD domain containing 5B	DENND5B	0,97
Transcript Identified by AceView, Entrez Gene ID(s) 23126	POGZ	0,97
insulin induced gene 1	INSIG1	0,97
adrenergic, beta, receptor kinase 2	ADRBK2	0,97
signal recognition particle 68kDa	SRP68	0,97
NSE1 homolog, SMC5-SMC6 complex component	NSMCE1	0,97
matrix metallopeptidase 9	MMP9	0,97
RASD family, member 2	RASD2	0,97
peroxidasin like	PXDNL	0,97
RAB33B, member RAS oncogene family	RAB33B	0,97
transmembrane protein 200C	TMEM200C	0,97
UBA-like domain containing 2	UBALD2	0,97
cytoplasmic polyadenylation element binding protein 4	CPEB4	0,97
ribosomal protein S6 kinase, 90kDa, polypeptide 1	RPS6KA1	0,97
Memczak2013 ALT_ACCEPTOR, ALT_DONOR, coding, INTERNAL, intronic best transcript NM_201998	SF1	0,97
N(alpha)-acetyltransferase 60, NatF catalytic subunit	NAA60	0,97
myelin transcription factor 1-like	MYT1L	0,97
interleukin 17 receptor B	IL17RB	0,97
gem nuclear organelle associated protein 7	GEMIN7	0,97
urocanate hydratase 1	UROC1	0,97
solute carrier family 23, member 3	SLC23A3	0,97
serine palmitoyltransferase, long chain base subunit 1	SPTLC1	0,97
protein phosphatase 6, regulatory subunit 1	PPP6R1	0,97
tubulin tyrosine ligase-like family member 9	TTLL9	0,97

spermatogenesis associated 24	SPATA24	0,97
GLE1 RNA export mediator	GLE1	0,97
hydroxymethylbilane synthase	HMBS	0,97
EPH receptor B6	EPHB6	0,97
tripartite motif containing 49D1; tripartite motif containing 49D2	TRIM49D1; TRIM49D2	0,97
membrane associated guanylate kinase, WW and PDZ domain containing 3	MAGI3	0,97
zinc finger protein 780A	ZNF780A	0,97
TBC1 domain family, member 16	TBC1D16	0,97
gap junction protein alpha 3	GJA3	0,97
motor neuron and pancreas homeobox 1	MNX1	0,97
OCIA domain containing 2	OCIAD2	0,97
Transcript Identified by AceView, Entrez Gene ID(s) 126231	ZNF573	0,97
hydroxysteroid (11-beta) dehydrogenase 1	HSD11B1	0,97
potassium channel subfamily M regulatory beta subunit 1	KCNMB1	0,97
adaptor-related protein complex 5, mu 1 subunit	AP5M1	0,97
discs, large (Drosophila) homolog-associated protein 5	DLGAP5	0,97
BRCA2 and CDKN1A interacting protein	BCCIP	0,97
lymphocyte cytosolic protein 1 (L-plastin)	LCP1	0,97
RAB interacting factor	RABIF	0,97
trafficking protein particle complex 4	TRAPPC4	0,97
tumor necrosis factor receptor superfamily, member 21	TNFRSF21	0,97
phospholipase D1, phosphatidylcholine-specific	PLD1	0,97
mitogen-activated protein kinase kinase kinase 1, E3 ubiquitin protein ligase	MAP3K1	0,97
glutamate receptor, metabotropic 5	GRM5	0,97
metaxin 1	MTX1	0,97
armadillo repeat containing, X-linked 5	ARMCX5	0,97
rabenosyn, RAB effector	RBSN	0,97
taste receptor, type 1, member 3	TAS1R3	0,97
ST8 alpha-N-acetyl-neuraminide alpha-2,8-sialyltransferase 5	ST8SIA5	0,97
transmembrane p24 trafficking protein 4	TMED4	0,97
prostaglandin D2 receptor (DP)	PTGDR	0,97
N-acylsphingosine amidohydrolase (non-lysosomal ceramidase) 2B	ASAH2B	0,97
CTAGE family, member 5	CTAGE5	0,97
Transcript Identified by AceView, Entrez Gene ID(s) 166378	SPATA5	0,97
uncharacterized LOC100652768; Salzman2013 ANNOTATED, ncRNA, OVCODE, OVERLAPTX, OVEXON best transcript NR_045215; Transcript Identified by AceView, Entrez Gene ID(s) 9159	LOC100652768; PCSK7	0,97
interleukin 26	IL26	0,97
cingulin-like 1	CGNL1	0,97
coenzyme Q10A	COQ10A	0,97

zinc finger homeobox 3	ZFHX3	0,97
hornerin	HRNR	0,97
nuclear factor of kappa light polypeptide gene enhancer in B-cells inhibitor, beta	NFKBIB	0,97
EF-hand calcium binding domain 8	EFCAB8	0,97
protein kinase, AMP-activated, beta 1 non-catalytic subunit	PRKAB1	0,97
small cell adhesion glycoprotein	SMAGP	0,97
NADH dehydrogenase (ubiquinone) 1 beta subcomplex, 11, 17.3kDa	NDUFB11	0,97
family with sequence similarity 92, member A1	FAM92A1	0,97
zinc finger protein 726; zinc finger protein 92 pseudogene 3	ZNF726; ZNF92P3	0,97
prolyl 4-hydroxylase, beta polypeptide	P4HB	0,97
DnaJ (Hsp40) homolog, subfamily C, member 11	DNAJC11	0,97
myocyte enhancer factor 2A	MEF2A	0,97
fibrillin 1	FBN1	0,97
derlin 3	DERL3	0,97
	AC208162.1	0,97
chromosome 12 open reading frame 73	C12orf73	0,97
ubiquitin specific peptidase 17-like family member 26; ubiquitin specific peptidase 17-like family member 24	USP17L26; USP17L24	0,97
ribosomal protein S3; small nucleolar RNA, C/D box 15A	RPS3; SNORD15A	0,97
ubiquitin specific peptidase 17-like family member 7	USP17L7	0,97
GDP-mannose pyrophosphorylase B	GMPPB	0,97
DEAH (Asp-Glu-Ala-His) box polypeptide 32	DHX32	0,97
dedicator of cytokinesis 6	DOCK6	0,97
microtubule-associated protein 1 light chain 3 beta	MAP1LC3B	0,97
ArfGAP with GTPase domain, ankyrin repeat and PH domain 3	AGAP3	0,97
family with sequence similarity 53, member C	FAM53C	0,97
phosphorylase kinase, beta	PHKB	0,97
TAO kinase 2	TAOK2	0,97
WAPL cohesin release factor	WAPL	0,97
tumor necrosis factor (ligand) superfamily, member 13	TNFSF13	0,97
coenzyme Q4	COQ4	0,97
transmembrane protein 170A	TMEM170A	0,97
NADH dehydrogenase (ubiquinone) 1 alpha subcomplex, 13	NDUFA13	0,96
UDP-GlcNAc:betaGal beta-1,3-N-acetylglucosaminyltransferase 9	B3GNT9	0,96
negative elongation factor complex member B	NELFB	0,96
LIM and SH3 protein 1	LASP1	0,96
mediator complex subunit 27	MED27	0,96
protease, serine 38	PRSS38	0,96
stathmin 1; microRNA 3917	STMN1; MIR3917	0,96

transmembrane protease, serine 9	TMPRSS9	0,96
cytidine and dCMP deaminase domain containing 1	CDADC1	0,96
glycerophosphodiester phosphodiesterase domain containing 2	GDPD2	0,96
long intergenic non-protein coding RNA 1276	LINC01276	0,96
cap methyltransferase 2	CMTR2	0,96
SET-like protein	SETSIP	0,96
zinc finger protein 502	ZNF502	0,96
uncharacterized LOC286238; Transcript Identified by AceView, Entrez Gene ID(s) 286238, RefSeq ID(s) NM_001100111; novel transcript	LOC286238; RP13-60M5.2	0,96
solute carrier family 13 (sodium/sulfate symporter), member 4	SLC13A4	0,96
zinc fingers and homeoboxes 1	ZHX1	0,96
lysozyme	LYZ	0,96
fibrous sheath interacting protein 1	FSIP1	0,96
NME/NM23 nucleoside diphosphate kinase 4	NME4	0,96
vitrin	VIT	0,96
N-acetyltransferase 16 (GCN5-related, putative)	NAT16	0,96
phosphatidylinositol binding clathrin assembly protein	PICALM	0,96
ubiquitin-conjugating enzyme E2E 3	UBE2E3	0,96
transmembrane protein 222	TMEM222	0,96
WD repeat and FYVE domain containing 2	WDFY2	0,96
CCDC169-SOHLH2 readthrough	CCDC169-SOHLH2	0,96
tripartite motif containing 32	TRIM32	0,96
serine/threonine kinase 32C	STK32C	0,96
ret finger protein-like 4A	RFPL4A	0,96
uromodulin	UMOD	0,96
interferon, alpha 1	IFNA1	0,96
ral guanine nucleotide dissociation stimulator-like 2	RGL2	0,96
phosphodiesterase 4C, cAMP-specific	PDE4C	0,96
olfactory receptor, family 5, subfamily AC, member 1 (gene/pseudogene)	OR5AC1	0,96
chromosome 9 open reading frame 40	C9orf40	0,96
exosome component 5	EXOSC5	0,96
far upstream element (FUSE) binding protein 3	FUBP3	0,96
interferon, gamma	IFNG	0,96
ABI family, member 3	ABI3	0,96
WD repeat domain 70	WDR70	0,96
mercaptopyruvate sulfurtransferase	MPST	0,96
membrane-spanning 4-domains, subfamily A, member 2	MS4A2	0,96
family with sequence similarity 102, member A	FAM102A	0,96
zinc finger protein 197	ZNF197	0,96
N-acetylglucosamine-1-phosphodiester alpha-N-acetylglucosaminidase	NAGPA	0,96
arachidonate 12-lipoxygenase	ALOX12	0,96

glutathione S-transferase mu 4	GSTM4	0,96
taste receptor, type 2, member 4	TAS2R4	0,96
transmembrane protein 198	TMEM198	0,96
mitochondrial transcription termination factor 1	MTERF1	0,96
protein tyrosine phosphatase, receptor type, E	PTPRE	0,96
notchless homolog 1 (Drosophila)	NLE1	0,96
HECT domain containing E3 ubiquitin protein ligase 4; microRNA 6861	HECTD4; MIR6861	0,96
helicase with zinc finger	HELZ	0,96
CTD small phosphatase 2	CTDSP2	0,96
target of EGR1, member 1 (nuclear)	TOE1	0,96
UBX domain protein 8	UBXN8	0,96
annexin A8	ANXA8	0,96
delta(4)-desaturase, sphingolipid 1	DEGS1	0,96
pleckstrin homology-like domain, family B, member 1; microRNA 6716	PHLDB1; MIR6716	0,96
forkhead box B2	FOXB2	0,96
solute carrier family 20 (phosphate transporter), member 2	SLC20A2	0,96
transmembrane protein 261	TMEM261	0,96
signal sequence receptor, gamma (translocon-associated protein gamma)	SSR3	0,96
alkB homolog 1, histone H2A dioxygenase	ALKBH1	0,96
autophagy related 101	ATG101	0,96
thymidine kinase 1, soluble	TK1	0,96
emopamil binding protein-like	EBPL	0,96
peripherin	PRPH	0,96
primase and DNA directed polymerase	PRIMPOL	0,96
baculoviral IAP repeat containing 7	BIRC7	0,96
potassium channel tetramerization domain containing 21	KCTD21	0,96
protein arginine methyltransferase 6	PRMT6	0,96
calcium channel, voltage-dependent, alpha 2/delta subunit 4	CACNA2D4	0,96
ubiquitin specific peptidase 8	USP8	0,96
arachidonate 5-lipoxygenase	ALOX5	0,96
keratin associated protein 13-2	KRTAP13-2	0,96
zinc finger protein 852	ZNF852	0,96
Bardet-Biedl syndrome 5	BBS5	0,96
WD repeat domain 76	WDR76	0,96
zinc finger protein 616	ZNF616	0,96
zinc finger protein 135	ZNF135	0,96
SURP and G-patch domain containing 1	SUGP1	0,96
zinc finger and BTB domain containing 21	ZBTB21	0,96
calcium channel flower domain containing 1	CACFD1	0,96
leucine rich repeat containing 1	LRRC1	0,96
NIMA-related kinase 10	NEK10	0,96
pancreatic and duodenal homeobox 1	PDX1	0,96

Ellis van Creveld protein	EVC	0,96
calmodulin-like 6	CALML6	0,96
G1 to S phase transition 1	GSPT1	0,96
poliovirus receptor-related 3	PVRL3	0,96
ubiquitin-like domain containing CTD phosphatase 1	UBLCP1	0,96
ribonuclease, RNase A family, 13 (non-active)	RNASE13	0,96
ring finger protein 212	RNF212	0,96
radial spoke 3 homolog (Chlamydomonas)	RSPH3	0,96
fragile X mental retardation, autosomal homolog 1	FXR1	0,96
actin related protein 2/3 complex subunit 3	ARPC3	0,96
tripartite motif containing 31	TRIM31	0,96
Transcript Identified by AceView, Entrez Gene ID(s) 128209; novel transcript	RP5-1198O20.4; KLF17	0,96
uncharacterized LOC102724957; novel transcript	LOC102724957; RP11-396O20.1	0,96
allantoicase	ALLC	0,96
LY6/PLAUR domain containing 6B	LYPD6B	0,96
caspase recruitment domain family, member 9	CARD9	0,96
hydroxymethylbilane synthase	HMBS	0,96
FtsJ RNA methyltransferase homolog 1 (E. coli)	FTSJ1	0,96
ring finger protein 151	RNF151	0,96
GNAS complex locus	GNAS	0,96
coagulation factor XIII, A1 polypeptide	F13A1	0,96
cell division cycle associated 4	CDCA4	0,96
endogenous retrovirus group V, member 2	ERVV-2	0,96
family with sequence similarity 84, member B	FAM84B	0,96
autophagy/beclin-1 regulator 1	AMBRA1	0,96
Jeck2013 ALT_ACCEPTOR, ALT_DONOR, coding, INTERNAL, intronic best transcript NM_001080396	FAM155A	0,96
mitochondrial fission regulator 1-like	MTFR1L	0,96
goosecoid homeobox	GSC	0,96
glycogen synthase 1 (muscle)	GYS1	0,96
cell division cycle 5-like	CDC5L	0,96
G-patch domain containing 11	GPATCH11	0,96
small nuclear RNA activating complex polypeptide 2	SNAPC2	0,96
chorionic gonadotropin, beta polypeptide 5; chorionic gonadotropin, beta polypeptide 8	CGB5; CGB8	0,96
retinol dehydrogenase 11 (all-trans/9-cis/11-cis)	RDH11	0,96
biogenesis of lysosomal organelles complex-1, subunit 3	BLOC1S3	0,96
disco-interacting protein 2 homolog B	DIP2B	0,96
transmembrane protein 55A	TMEM55A	0,96
CDC42 effector protein (Rho GTPase binding) 2	CDC42EP2	0,96
chromosome 1 open reading frame 162	C1orf162	0,96

zinc finger, CCHC domain containing 17	ZCCHC17	0,96
Memczak2013 ANTISENSE, CDS, coding, INTERNAL, UTR5 best transcript NM_001199573	TRIM22	0,96
catenin, beta like 1	CTNNBL1	0,96
TSNAX-DISC1 readthrough (NMD candidate)	TSNAX-DISC1	0,96
PMS1 homolog 1, mismatch repair system component	PMS1	0,96
retinitis pigmentosa GTPase regulator interacting protein 1	RPGRIP1	0,96
Rho GTPase activating protein 29	ARHGAP29	0,96
ankyrin repeat domain 36C	ANKRD36C	0,96
SERTA domain containing 3	SERTAD3	0,96
T cell receptor gamma joining 1; T cell receptor gamma constant 2; T cell receptor gamma joining 2; T cell receptor gamma variable 9; T cell receptor gamma constant 1; T cell receptor gamma joining P; T cell receptor gamma joining P2	TRGJ1; TRGC2; TRGJ2; TRGV9; TRGC1; TRGJP; TRGJP2	0,96
sperm acrosome associated 1	SPACA1	0,96
major histocompatibility complex, class I, E	HLA-E	0,96
caspase 10	CASP10	0,96
LIM and senescent cell antigen-like domains 1	LIMS1	0,96
Sp7 transcription factor	SP7	0,96
spindlin 1	SPIN1	0,96
sodium channel, non voltage gated 1 gamma subunit	SCNN1G	0,96
cytochrome c oxidase assembly factor 4 homolog	COA4	0,96
RNF103-CHMP3 readthrough; charged multivesicular body protein 3	RNF103-CHMP3; CHMP3	0,96
family with sequence similarity 221, member B	FAM221B	0,96
SH3 domain binding glutamate-rich protein like 2	SH3BGRL2	0,96
solute carrier family 25, member 39	SLC25A39	0,96
C2 calcium-dependent domain containing 4D	C2CD4D	0,96
ST6 beta-galactosamide alpha-2,6-sialyltranferase 1	ST6GAL1	0,96
SEC24 homolog D, COPII coat complex component	SEC24D	0,96
ZFP91 zinc finger protein	ZFP91	0,96
zinc finger protein 341	ZNF341	0,96
phospholipid scramblase family, member 5	PLSCR5	0,96
GINS complex subunit 1 (Psf1 homolog)	GINS1	0,96
solute carrier organic anion transporter family, member 1B7 (non-functional); solute carrier organic anion transporter family, member 1B1	SLCO1B7; SLCO1B1	0,96
erythrocyte membrane protein band 4.1 like 4A	EPB41L4A	0,96
killer cell immunoglobulin-like receptor, two domains, long cytoplasmic tail, 4	KIR2DL4	0,96
zinc finger, DHHC-type containing 17	ZDHHC17	0,96
microtubule associated protein 1A	MAP1A	0,96
leukocyte immunoglobulin-like receptor, subfamily B (with TM and ITIM domains), member 2	LILRB2	0,96
endothelial cell-specific molecule 1	ESM1	0,96

centrosomal protein 131kDa	CEP131	0,96
kelch-like family member 23	KLHL23	0,96
calcium channel flower domain containing 1	CACFD1	0,96
RAS-like, family 10, member B	RASL10B	0,96
cyclin B3	CCNB3	0,96
neuromedin B receptor	NMBR	0,96
transmembrane protein 202	TMEM202	0,96
uncharacterized protein FLJ30679; uncharacterized protein FLJ30679 [Source:EntrezGene;Acc:146512]; novel transcript	FLJ30679; RP11-463O9.8	0,96
glutamate receptor, ionotropic, N-methyl D-aspartate 2B	GRIN2B	0,96
T-cell, immune regulator 1, ATPase, H+ transporting, lysosomal V0 subunit A3	TCIRG1	0,96
carbonyl reductase 4	CBR4	0,96
cytoplasmic linker associated protein 1	CLASP1	0,96
zinc finger protein 800	ZNF800	0,96
WAP, follistatin/kazal, immunoglobulin, kunitz and netrin domain containing 2	WFIKKN2	0,96
tau tubulin kinase 1	TTBK1	0,96
zinc finger, matrin-type 3	ZMAT3	0,96
enolase-phosphatase 1	ENOPH1	0,96
tissue specific transplantation antigen P35B	TSTA3	0,96
progesterone receptor membrane component 1	PGRMC1	0,96
ring finger protein 213	RNF213	0,96
beta-site APP-cleaving enzyme 2	BACE2	0,96
desmocollin 2	DSC2	0,96
complement component 6	C6	0,96
otopetrin 3	OTOP3	0,96
olfactory receptor, family 6, subfamily K, member 6	OR6K6	0,96
phosphatidylinositol-4,5-bisphosphate 3-kinase, catalytic subunit alpha	PIK3CA	0,96
adenosine monophosphate deaminase 3	AMPD3	0,96
RNA binding motif protein 10	RBM10	0,96
nuclear pore complex interacting protein family, member A2	NPIPA2	0,96
neuronal pentraxin I	NPTX1	0,96
adenomatosis polyposis coli down-regulated 1-like	APCDD1L	0,96
EGF-like-domain, multiple 6	EGFL6	0,96
melanocortin 1 receptor (alpha melanocyte stimulating hormone receptor)	MC1R	0,96
Transcript Identified by AceView, Entrez Gene ID(s) 91445	RNF185	0,96
TBC1 domain family, member 14	TBC1D14	0,96
TAO kinase 3	TAOK3	0,96
eukaryotic translation initiation factor 3, subunit M	EIF3M	0,96
pleckstrin and Sec7 domain containing	PSD	0,96
solute carrier family 13 (sodium-dependent dicarboxylate transporter), member 3; TP53 regulating kinase	SLC13A3; TP53RK	0,96

integral membrane protein 2A	ITM2A	0,96
histone cluster 1, H4b	HIST1H4B	0,96
keratin associated protein 4-7	KRTAP4-7	0,96
solute carrier family 22 (organic cation/carnitine transporter), member 16	SLC22A16	0,96
amyloid beta (A4) precursor protein-binding, family B, member 1 (Fe65)	APBB1	0,96
myomesin 2	MYOM2	0,96
kelch repeat and BTB (POZ) domain containing 11	KBTBD11	0,96
keratin associated protein 4-1	KRTAP4-1	0,96
TAP binding protein (tapasin)	TAPBP	0,96
zinc finger, MIZ-type containing 1	ZMIZ1	0,96
tRNA-yW synthesizing protein 5	TYW5	0,96
up-regulated during skeletal muscle growth 5 homolog (mouse); microRNA 1307	USMG5; MIR1307	0,96
speedy/RINGO cell cycle regulator family member E16 [Source:HGNC Symbol;Acc:HGNC:51512]	SPDYE16	0,96
pre T-cell antigen receptor alpha	PTCRA	0,96
pyridine nucleotide-disulphide oxidoreductase domain 2; microRNA 1287	PYROXD2; MIR1287	0,96
actin related protein 2/3 complex subunit 2	ARPC2	0,96
tachykinin 4 (hemokinin)	TAC4	0,96
PDZ and LIM domain 1	PDLIM1	0,96
breast cancer metastasis-suppressor 1-like	BRMS1L	0,96
RTF1 homolog, Paf1/RNA polymerase II complex component	RTF1	0,96
Transcript Identified by AceView, Entrez Gene ID(s) 2043	EPHA4	0,96
taspase 1	TASP1	0,96
hect domain and RLD 2 pseudogene 2	HERC2P2	0,96
regucalcin	RGN	0,96
amylase, alpha 1A (salivary); amylase, alpha 1C (salivary)	AMY1A; AMY1C	0,96
F-box protein 32	FBXO32	0,96
zinc finger, matrin-type 2	ZMAT2	0,96
acyl-CoA synthetase long-chain family member 3	ACSL3	0,96
selenium binding protein 1	SELENBP1	0,96
ER membrane protein complex subunit 6	EMC6	0,96
KN motif and ankyrin repeat domains 2	KANK2	0,96
ring finger protein 115	RNF115	0,96
SCP2 sterol-binding domain containing 1	SCP2D1	0,96
guanylate binding protein family, member 6	GBP6	0,96
DEAD (Asp-Glu-Ala-Asp) box polypeptide 28	DDX28	0,96
nudix hydrolase 1	NUDT1	0,96
coiled-coil domain containing 114	CCDC114	0,96
centrosomal protein 55kDa	CEP55	0,96

tumor necrosis factor receptor superfamily, member 10c, decoy without an intracellular domain	TNFRSF10C	0,96
PPP5 tetratricopeptide repeat domain containing 1	PPP5D1	0,96
zinc finger with KRAB and SCAN domains 4	ZKSCAN4	0,96
coiled-coil domain containing 102A	CCDC102A	0,96
ribosomal protein L7a	RPL7A	0,96
long intergenic non-protein coding RNA 1567	LINC01567	0,96
dual specificity tyrosine-(Y)-phosphorylation regulated kinase 1B	DYRK1B	0,96
olfactory receptor, family 3, subfamily A, member 2	OR3A2	0,96
keratin associated protein 4-3	KRTAP4-3	0,96
LIM domain only 3 (rhombotin-like 2)	LMO3	0,96
uncharacterized LOC100505478; putative novel transcript	LOC100505478; RP11-402G3.3	0,96
chemokine (C-X-C motif) receptor 4	CXCR4	0,96
MAGE family member H1	MAGEH1	0,96
coiled-coil domain containing 54	CCDC54	0,96
transmembrane protein 70; ribosomal protein S20 pseudogene 21	TMEM70; RPS20P21	0,96
glutamate receptor interacting protein 1	GRIP1	0,96
acetylcholinesterase (Yt blood group)	ACHE	0,96
SH3 domain containing 19	SH3D19	0,96
solute carrier family 25 (mitochondrial carrier; citrate transporter), member 1	SLC25A1	0,96
lysosomal-associated membrane protein 2	LAMP2	0,96
chromosome 14 open reading frame 177	C14orf177	0,96
TBP-like 1	TBPL1	0,96
polo-like kinase 1	PLK1	0,96
out at first homolog	OAF	0,96
aquaporin 9	AQP9	0,96
ADP-ribosylation factor like GTPase 8A	ARL8A	0,96
Transcript Identified by AceView, Entrez Gene ID(s) 153129	SLC38A9	0,96
interferon, lambda receptor 1	IFNLR1	0,96
NODAL modulator 3; NODAL modulator 2	NOMO3; NOMO2	0,96
Memczak2013 ALT_ACCEPTOR, ALT_DONOR, coding, INTERNAL, intronic best transcript NM_013314	BLNK	0,96
RNA binding motif protein, X-linked; small nucleolar RNA, C/D box 61	RBMX; SNORD61	0,96
Transcript Identified by AceView, Entrez Gene ID(s) 23028	KDM1A	0,96
H2A histone family, member B1; H2A histone family, member B2; H2A histone family, member B3	H2AFB1; H2AFB2; H2AFB3	0,96
KIAA1033	KIAA1033	0,96
microfibrillar associated protein 3	MFAP3	0,96

Transcript Identified by AceView, Entrez Gene ID(s) 23184; novel transcript, antisense to CEMIP	RP11-351M8.2; MESDC2; RP11-351M8.1	0,96
desmoplakin	DSP	0,96
SEC14-like lipid binding 3	SEC14L3	0,96
eukaryotic translation initiation factor 4E	EIF4E	0,96
RNA (guanine-7-) methyltransferase	RNMT	0,96
tetraspanin 33	TSPAN33	0,96
ATPase, H+ transporting, lysosomal 56/58kDa, V1 subunit B2	ATP6V1B2	0,96
ryanodine receptor 1 (skeletal)	RYR1	0,96
coiled-coil domain containing 64B	CCDC64B	0,96
ER membrane protein complex subunit 4	EMC4	0,96
solute carrier family 9, subfamily A (NHE9, cation proton antiporter 9), member 9	SLC9A9	0,96
epithelial mitogen	EPGN	0,96
erythrocyte membrane protein band 4.2	EPB42	0,96
DEAD/H (Asp-Glu-Ala-Asp/His) box polypeptide 12, pseudogene; DEAD/H (Asp-Glu-Ala-Asp/His) box helicase 11	DDX12P; DDX11	0,96
c-Maf inducing protein	CMIP	0,96
transmembrane 4 L six family member 20	TM4SF20	0,96
nuclear factor, interleukin 3 regulated	NFIL3	0,96
upper zone of growth plate and cartilage matrix associated	UCMA	0,96
mediator complex subunit 1	MED1	0,96
Ca++-dependent secretion activator 2	CADPS2	0,96
ATP binding cassette subfamily C member 5	ABCC5	0,96
FH2 domain containing 1	FHDC1	0,96
heterogeneous nuclear ribonucleoprotein A3; microRNA 4444-2; microRNA 4444-1	HNRNPA3; MIR4444-2; MIR4444-1	0,96
zinc and ring finger 1, E3 ubiquitin protein ligase	ZNRF1	0,96
vitamin K epoxide reductase complex subunit 1	VKORC1	0,96
phospholipase B domain containing 2	PLBD2	0,96
ER degradation enhancer, mannosidase alpha-like 3	EDEM3	0,96
LSM11, U7 small nuclear RNA associated	LSM11	0,96
SPOC domain containing 1	SPOCD1	0,96
alanine--glyoxylate aminotransferase 2	AGXT2	0,96
zinc finger protein, Y-linked	ZFY	0,96
lethal giant larvae homolog 1 (Drosophila)	LLGL1	0,96
ataxin 3	ATXN3	0,96
fucose-1-phosphate guanylyltransferase	FPGT	0,96
keratin associated protein 6-3	KRTAP6-3	0,96
topoisomerase (DNA) II alpha	TOP2A	0,96
LFNG O-fucosylpeptide 3-beta-N-acetylglucosaminyltransferase; microRNA 4648	LFNG; MIR4648	0,96
UNC homeobox	UNCX	0,96

kallikrein related peptidase 14	KLK14	0,96
phosducin like	PDCL	0,96
DEAD (Asp-Glu-Ala-Asp) box helicase 5; microRNA 3064; microRNA 5047	DDX5; MIR3064; MIR5047	0,96
KH domain containing, RNA binding, signal transduction associated 1	KHDRBS1	0,96
cancer/testis antigen 1B	CTAG1B	0,96
WWC2 antisense RNA 2	WWC2-AS2	0,96
dedicator of cytokinesis 9	DOCK9	0,96
cadherin 4, type 1, R-cadherin (retinal)	CDH4	0,96
alveolar soft part sarcoma chromosome region, candidate 1	ASPSCR1	0,96
integrin beta 1 binding protein 1	ITGB1BP1	0,96
homeobox B1	HOXB1	0,96
Rab geranylgeranyltransferase, beta subunit; small nucleolar RNA, C/D box 45B; small nucleolar RNA, C/D box 45A; small nucleolar RNA, C/D box 45C	RABGGTB; SNORD45B; SNORD45A; SNORD45C	0,96
A kinase (PRKA) anchor protein 8-like	AKAP8L	0,96
TATA box binding protein (TBP)-associated factor, RNA polymerase I, C, 110kDa	TAF1C	0,96
LSM14A mRNA processing body assembly factor	LSM14A	0,96
adrenoceptor beta 1	ADRB1	0,96
solute carrier family 19 (folate transporter), member 1	SLC19A1	0,96
pyridoxal-dependent decarboxylase domain containing 1	PDXDC1	0,96
major facilitator superfamily domain containing 12	MFSD12	0,96
dipeptidase 1 (renal)	DPEP1	0,96
squamous cell carcinoma antigen recognized by T-cells 3	SART3	0,96
tetraspanin 3	TSPAN3	0,96
proteasome activator subunit 3	PSME3	0,96
regulator of G-protein signaling 2	RGS2	0,96
spen family transcriptional repressor	SPEN	0,96
dynein, light chain, Tctex-type 3	DYNLT3	0,96
nuclear receptor subfamily 3, group C, member 1 (glucocorticoid receptor)	NR3C1	0,96
poly(A) polymerase alpha	PAPOLA	0,96
Memczak2013 ANTISENSE, coding, INTERNAL, intronic best transcript NM_001020819; novel transcript, antisense to MYADM	AC008753.6; MYADM	0,96
C-type lectin domain family 1, member B	CLEC1B	0,96
phospholipase A2-activating protein	PLAA	0,96
ubiquitin specific peptidase 17-like family member 19	USP17L19	0,96
inositol polyphosphate-5-phosphatase D	INPP5D	0,96
coiled-coil domain containing 122	CCDC122	0,96
H6 family homeobox 2	HMX2	0,96
trichohyalin	TCHH	0,96

synaptonemal complex central element protein 1-like	SYCE1L	0,96
papillary renal cell carcinoma (translocation-associated)	PRCC	0,96
homolog of rat pragma of Rnd2; Tyrosine-protein kinase SgK223 [Source:UniProtKB/Swiss-Prot;Acc:Q86YV5]	SGK223	0,96
cartilage associated protein	CRTAP	0,96
scavenger receptor class B, member 1	SCARB1	0,96
RecQ helicase-like 5	RECQL5	0,96
polymerase (DNA directed), epsilon 3, accessory subunit	POLE3	0,96
MLX, MAX dimerization protein	MLX	0,96
eukaryotic translation initiation factor 3, subunit J	EIF3J	0,96
cilia and flagella associated protein 36	CFAP36	0,96
SWI/SNF related, matrix associated, actin dependent regulator of chromatin, subfamily a, member 1	SMARCA1	0,96
histidine decarboxylase	HDC	0,96
FRAS1 related extracellular matrix protein 2	FREM2	0,96
natural killer cell cytotoxicity receptor 3 ligand 1	NCR3LG1	0,96
leucine rich repeat containing 37, member A5, pseudogene	LRRC37A5P	0,96
coiled-coil domain containing 12	CCDC12	0,96
heart development protein with EGF-like domains 1	HEG1	0,96
autophagy related 16-like 1	ATG16L1	0,96
myotubularin related protein 7	MTMR7	0,96
BCL2-like 14 (apoptosis facilitator)	BCL2L14	0,96
polymerase (RNA) II (DNA directed) polypeptide C, 33kDa	POLR2C	0,96
G-patch domain containing 2 like	GPATCH2L	0,96
transcription elongation factor A (SII)-like 4	TCEAL4	0,96
zinc finger protein 222	ZNF222	0,96
SLIT-ROBO Rho GTPase activating protein 2C	SRGAP2C	0,96
family with sequence similarity 13, member C	FAM13C	0,96
olfactory receptor, family 10, subfamily G, member 9	OR10G9	0,96
hemoglobin, zeta	HBZ	0,96
DENN/MADD domain containing 3	DENND3	0,96
OTU deubiquitinase 7B	OTUD7B	0,96
olfactory receptor, family 13, subfamily A, member 1	OR13A1	0,96
WD repeat domain 45B	WDR45B	0,96
MAP kinase interacting serine/threonine kinase 1	MKNK1	0,96
family with sequence similarity 175, member A	FAM175A	0,96
gamma-glutamyltransferase light chain 2	GGTLC2	0,96
olfactory receptor, family 4, subfamily A, member 8 (gene/pseudogene) [Source:HGNC Symbol;Acc:HGNC:15165]	OR4A8	0,96
chromosome 11 open reading frame 71	C11orf71	0,96
membrane-spanning 4-domains, subfamily A, member 4A	MS4A4A	0,96
protein arginine methyltransferase 7	PRMT7	0,96
mucolipin 3	MCOLN3	0,96
DnaJ (Hsp40) homolog, subfamily C, member 24	DNAJC24	0,96

leucine rich repeat containing 69; microRNA 4661	LRRC69; MIR4661	0,96
mitochondrial ribosomal protein L43	MRPL43	0,96
cytochrome c oxidase subunit IV isoform 2 (lung)	COX4I2	0,96
olfactory receptor, family 4, subfamily K, member 2	OR4K2	0,96
GH3 domain containing	GHDC	0,96
SLAIN motif family member 2	SLAIN2	0,96
ankyrin repeat domain 10	ANKRD10	0,96
Transcript Identified by AceView, Entrez Gene ID(s) 7511	XPNPEP1	0,96
DnaJ (Hsp40) homolog, subfamily B, member 6	DNAJB6	0,96
autophagy related 13	ATG13	0,96
protein kinase, DNA-activated, catalytic polypeptide	PRKDC	0,96
tudor and KH domain containing	TDRKH	0,96
ADP-ribosylation factor like GTPase 6	ARL6	0,96
dihydrolipoamide branched chain transacylase E2	DBT	0,96
RIC1 homolog, RAB6A GEF complex partner 1	RIC1	0,96
V-set and immunoglobulin domain containing 10	VSIG10	0,96
HIV-1 Tat specific factor 1	HTATSF1	0,96
bromodomain adjacent to zinc finger domain 1A	BAZ1A	0,96
DENN/MADD domain containing 2D	DENND2D	0,96
protein tyrosine phosphatase, non-receptor type 3	PTPN3	0,96
trace amine associated receptor 2	TAAR2	0,96
family with sequence similarity 63, member A	FAM63A	0,96
torsin family 1, member B (torsin B)	TOR1B	0,96
interleukin 23, alpha subunit p19	IL23A	0,96
SCAN domain containing 1	SCAND1	0,96
membrane associated ring finger 2	MARCH2	0,96
solute carrier family 22 (organic anion transporter), member 6	SLC22A6	0,96
uncharacterized LOC339803; Transcript Identified by AceView, Entrez Gene ID(s) 339803; novel transcript	LOC339803; AC016747.3	0,96
V-set and immunoglobulin domain containing 4	VSIG4	0,96
Transcript Identified by AceView, Entrez Gene ID(s) 26051	PPP1R16B	0,96
long intergenic non-protein coding RNA 1429	LINC01429	0,96
retinoid X receptor beta	RXRB	0,96
retinitis pigmentosa 2 (X-linked recessive)	RP2	0,96
sterol carrier protein 2	SCP2	0,96
REX4 homolog, 3'-5' exonuclease [Source:HGNC Symbol;Acc:HGNC:12820]	REXO4	0,96
proline-rich coiled-coil 1	PRRC1	0,96
zinc finger protein 852	ZNF852	0,96
retinol dehydrogenase 5 (11-cis/9-cis)	RDH5	0,96
cyclin C	CCNC	0,96
myeloid differentiation primary response 88	MYD88	0,96
chromosome 2 open reading frame 16	C2orf16	0,96
transmembrane protein 151B	TMEM151B	0,96

dihydrofolate reductase	DHFR	0,96
phospholipid phosphatase 1; ring finger protein 138, E3 ubiquitin protein ligase pseudogene 1	PLPP1; RNF138P1	0,96
tetratricopeptide repeat domain 39C	TTC39C	0,96
FUN14 domain containing 2	FUNDC2	0,96
proline rich 9	PRR9	0,96
deoxynucleotidyltransferase, terminal, interacting protein 2	DNTTIP2	0,96
chromosome 1 open reading frame 68	C1orf68	0,96
abhydrolase domain containing 16A	ABHD16A	0,96
surfeit 4	SURF4	0,96
ATPase, H+/K+ exchanging, beta polypeptide	ATP4B	0,96
thyroid hormone receptor associated protein 3	THRAP3	0,96
NUT midline carcinoma, family member 1	NUTM1	0,96
zinc finger, SWIM-type containing 1	ZSWIM1	0,96
signal-induced proliferation-associated 1 like 3	SIPA1L3	0,96
proteasome 26S subunit, non-ATPase 12	PSMD12	0,96
chromosome 2 open reading frame 70	C2orf70	0,96
aldo-keto reductase family 1, member C1	AKR1C1	0,96
carboxypeptidase O	CPO	0,96
FK506 binding protein 2	FKBP2	0,96
RAB7A, member RAS oncogene family	RAB7A	0,96
TMF1-regulated nuclear protein 1	TRNP1	0,96
cytochrome c oxidase subunit VIc	COX6C	0,96
Transcript Identified by AceView, Entrez Gene ID(s) 347088; adhesion G protein-coupled receptor D2 [Source:HGNC Symbol;Acc:HGNC:18651]	GPR144; ADGRD2	0,96
cleavage and polyadenylation specific factor 4	CPSF4	0,96
vacuole membrane protein 1; microRNA 21	VMP1; MIR21	0,96
urocortin	UCN	0,96
vitamin K epoxide reductase complex subunit 1 like 1	VKORC1L1	0,96
chromosome 11 open reading frame 97	C11orf97	0,96
coiled-coil domain containing 25	CCDC25	0,96
interleukin enhancer binding factor 3	ILF3	0,96
pancreatic lipase-related protein 1	PNLIPRP1	0,96
sarcoglycan gamma	SGCG	0,96
fibrillin 3	FBN3	0,96
spermatogenesis associated 3	SPATA3	0,96
tripartite motif containing 40	TRIM40	0,96
CTAGE family, member 6	CTAGE6	0,96
chromosome 2 open reading frame 88	C2orf88	0,96
SPT2 chromatin protein domain containing 1	SPTY2D1	0,96
septin 6	sept-06	0,96
ATP synthase mitochondrial F1 complex assembly factor 1	ATPAF1	0,96
GATA zinc finger domain containing 2A; microRNA 640	GATAD2A; MIR640	0,96

pleckstrin homology-like domain, family A, member 3	PHLDA3	0,96
snurportin 1	SNUPN	0,96
bone morphogenetic protein 7	BMP7	0,96
ATP synthase, H+ transporting, mitochondrial Fo complex subunit E	ATP5I	0,96
casein kinase 1, epsilon	CSNK1E	0,96
nudix hydrolase 16 like 1	NUDT16L1	0,96
MT-RNR2-like 13	MTRNR2L13	0,96
heat shock 27kDa protein 1	HSPB1	0,96
protein phosphatase 2A activator, regulatory subunit 4	PPP2R4	0,96
T-cell immunoreceptor with Ig and ITIM domains	TIGIT	0,96
dual specificity phosphatase 16	DUSP16	0,96
methionine sulfoxide reductase B3	MSRB3	0,96
aurora kinase C	AURKC	0,96
defensin, beta 105A	DEFB105A	0,96
major facilitator superfamily domain containing 10	MFSD10	0,96
kelch-like family member 36	KLHL36	0,96
olfactory receptor, family 5, subfamily H, member 14	OR5H14	0,96
chromosome 11 open reading frame 40	C11orf40	0,96
amyloid beta (A4) precursor-like protein 2	APLP2	0,96
deafness, autosomal recessive 31	DFNB31	0,96
ubiquitin-like modifier activating enzyme 5	UBA5	0,96
tetraspanin 1	TSPAN1	0,96
leucine rich adaptor protein 1-like	LURAP1L	0,96
H3 histone, family 3A; H3 histone, family 3A, pseudogene 4	H3F3A; H3F3AP4	0,96
olfactory receptor, family 13, subfamily C, member 3	OR13C3	0,96
keratin associated protein 3-1	KRTAP3-1	0,96
guanine nucleotide binding protein (G protein), gamma 13	GNG13	0,96
RALY heterogeneous nuclear ribonucleoprotein	RALY	0,96
tetratricopeptide repeat domain 9B	TTC9B	0,96
makorin ring finger protein 2	MKRN2	0,96
UBX domain protein 6	UBXN6	0,96
solute carrier family 26 (anion exchanger), member 2	SLC26A2	0,96
syntaxin 16	STX16	0,96
tubulin, alpha 1c	TUBA1C	0,96
transmembrane protein 240	TMEM240	0,96
sarcolemma associated protein	SLMAP	0,96
trefoil factor 3	TFF3	0,96
Memczak2013 ANTISENSE, coding, INTERNAL, intronic best transcript NM_004544	NDUFA10	0,96
thrombospondin-type laminin G domain and EAR repeats	TSPEAR	0,96
IKAROS family zinc finger 3	IKZF3	0,96
tumor necrosis factor receptor superfamily, member 11a, NFKB activator	TNFRSF11A	0,96

Rieske (Fe-S) domain containing	RFESD	0,96
kelch-like family member 34	KLHL34	0,96
PDGFA associated protein 1	PDAP1	0,96
coronin, actin binding protein, 1C	CORO1C	0,96
ZFP1 zinc finger protein	ZFP1	0,96
SET domain containing 6	SETD6	0,96
peptidase M20 domain containing 2	PM20D2	0,96
neural cell adhesion molecule 1	NCAM1	0,96
Rho guanine nucleotide exchange factor 17	ARHGEF17	0,96
zinc finger, DHHC-type containing 22	ZDHHC22	0,96
CD37 molecule	CD37	0,96
muscleblind-like splicing regulator 3	MBNL3	0,96
glucosidase, beta (bile acid) 2	GBA2	0,96
paired box 3	PAX3	0,96
chromosome 14 open reading frame 166	C14orf166	0,96
Transcript Identified by AceView, Entrez Gene ID(s) 225	ABCD2	0,96
smoothelin	SMTN	0,96
prolyl 3-hydroxylase family member 4 (non-enzymatic)	P3H4	0,96
calcium channel flower domain containing 1	CACFD1	0,96
osteoclast stimulating factor 1	OSTF1	0,96
serum response factor	SRF	0,96
centrobin, centrosomal BRCA2 interacting protein	CNTROB	0,96
tumor necrosis factor, alpha-induced protein 6	TNFAIP6	0,96
fibulin 2	FBLN2	0,96
DnaJ (Hsp40) homolog, subfamily A, member 2	DNAJA2	0,96
leucine rich repeat containing 43	LRRC43	0,96
small nuclear ribonucleoprotein polypeptide F	SNRPF	0,96
ubiquitin specific peptidase 18	USP18	0,96
olfactory receptor, family 4, subfamily K, member 5	OR4K5	0,96
integrin alpha V	ITGAV	0,96
zinc finger protein 488	ZNF488	0,96
teneurin transmembrane protein 4	TENM4	0,96
family with sequence similarity 179, member A	FAM179A	0,96
mitochondrial ribosomal protein S28	MRPS28	0,96
myosin VIIA	MYO7A	0,96
cAMP responsive element binding protein-like 2	CREBL2	0,96
interleukin 13 receptor, alpha 1	IL13RA1	0,96
neutrophil cytosolic factor 4	NCF4	0,96
uncharacterized LOC115110; novel transcript	LOC115110; RP3-395M20.8	0,96
out at first homolog	OAF	0,96
antagonist of mitotic exit network 1 homolog	AMN1	0,96
LEM domain containing 3	LEMD3	0,96
EH domain binding protein 1	EHBP1	0,96
apolipoprotein C-III	APOC3	0,96

chemokine (C-C motif) ligand 25	CCL25	0,96
transient receptor potential cation channel, subfamily A, member 1	TRPA1	0,96
interleukin 34	IL34	0,96
charged multivesicular body protein 5	CHMP5	0,96
aarF domain containing kinase 2	ADCK2	0,96
interferon-induced protein with tetratricopeptide repeats 1	IFIT1	0,96
nucleoporin 62kDa; interleukin 4 induced 1	NUP62; IL4I1	0,96
7-dehydrocholesterol reductase	DHCR7	0,96
biorientation of chromosomes in cell division 1-like 1	BOD1L1	0,96
small integral membrane protein 15	SMIM15	0,96
chromosome 14 open reading frame 80	C14orf80	0,96
extracellular matrix protein 2, female organ and adipocyte specific	ECM2	0,96
NADH dehydrogenase (ubiquinone) 1 beta subcomplex, 4, 15kDa	NDUFB4	0,96
aralkylamine N-acetyltransferase	AANAT	0,96
solute carrier family 25, member 44	SLC25A44	0,96
TAR DNA binding protein	TARDBP	0,96
family with sequence similarity 153, member B	FAM153B	0,96
splA/ryanodine receptor domain and SOCS box containing 4	SPSB4	0,96
ST3 beta-galactoside alpha-2,3-sialyltransferase 6	ST3GAL6	0,96
cathepsin Z	CTSZ	0,96
spindle and centriole associated protein 1	SPICE1	0,96
BCL2-like 14 (apoptosis facilitator)	BCL2L14	0,96
dual specificity phosphatase and pro isomerase domain containing 1	DUPD1	0,96
phosphorylase kinase, alpha 1 (muscle)	PHKA1	0,96
keratin 71, type II	KRT71	0,96
BCL2-like 1	BCL2L1	0,96
defensin, beta 135	DEFB135	0,96
tubulin, beta 2B class IIb	TUBB2B	0,96
MMS19 homolog, cytosolic iron-sulfur assembly component	MMS19	0,96
ribosomal protein L10; small nucleolar RNA, H/ACA box 70	RPL10; SNORA70	0,96
UbiA prenyltransferase domain containing 1	UBIAD1	0,96
arginyl aminopeptidase (aminopeptidase B)-like 1	RNPEPL1	0,96
ATPase, class I, type 8B, member 4	ATP8B4	0,96
sodium channel, voltage gated, type I alpha subunit	SCN1A	0,96
DNA replication and sister chromatid cohesion 1	DSCC1	0,96
exocyst complex component 3-like 1	EXOC3L1	0,96
solute carrier family 22, member 23	SLC22A23	0,96
t-complex 10-like 2	TCP10L2	0,96
proteasome activator subunit 1	PSME1	0,96
protocadherin beta 12	PCDHB12	0,96
KIAA1614	KIAA1614	0,96

centromere protein K	CENPK	0,96
keratinocyte associated protein 3	KRTCAP3	0,96
TATA box binding protein associated factor 1D; small nucleolar RNA, H/ACA box 8; small nucleolar RNA, H/ACA box 18; small nucleolar RNA, H/ACA box 40; small nucleolar RNA, H/ACA box 1; small nucleolar RNA, H/ACA box 25; small nucleolar RNA, H/ACA box 32; small nucleolar RNA, C/D box 5; microRNA 1304	TAF1D; SNORA8; SNORA18; SNORA40; SNORA1; SNORA25; SNORA32; SNORD5; MIR1304	0,96
3-oxoacid CoA-transferase 1	OXCT1	0,96
ventral anterior homeobox 1	VAX1	0,96
lecithin-cholesterol acyltransferase	LCAT	0,96
chromogranin A	CHGA	0,96
kinesin family member 13B	KIF13B	0,96
neuropilin (NRP) and tolloid (TLL)-like 1	NETO1	0,96
complement component 2	C2	0,96
cyclin L1	CCNL1	0,96
DENN/MADD domain containing 5A	DENND5A	0,96
Rho GTPase activating protein 9	ARHGAP9	0,96
interleukin 7	IL7	0,96
uncharacterized LOC388882; novel transcript	LOC388882; AP000345.1	0,96
leucine-rich single-pass membrane protein 2	LSMEM2	0,96
calcium binding protein 39	CAB39	0,96
arrestin, beta 1	ARRB1	0,96
calcium channel, voltage-dependent, alpha 2/delta subunit 2	CACNA2D2	0,96
oxoglutarate (alpha-ketoglutarate) dehydrogenase (lipoamide)	OGDH	0,96
complement component 1, q subcomponent-like 4	C1QL4	0,96
coiled-coil domain containing 158	CCDC158	0,96
inter-alpha-trypsin inhibitor heavy chain family, member 4	ITIH4	0,96
septin 11	sept-11	0,96
UFM1-specific peptidase 1 (inactive)	UFSP1	0,96
SRSF protein kinase 1	SRPK1	0,96
Jeck2013 ANTISENSE, CDS, coding, INTERNAL, intronic, OVCODE, OVEXON best transcript NM_181453	GCC2	0,96
peptidylprolyl isomerase H (cyclophilin H)	PPIH	0,96
chromosome 19 open reading frame 54	C19orf54	0,96
solute carrier family 38, member 10	SLC38A10	0,96
Transcript Identified by AceView, Entrez Gene ID(s) 10211	FLOT1	0,96
S100 calcium binding protein Z	S100Z	0,96
mitochondrial ribosomal protein S17	MRPS17	0,96
TAR (HIV-1) RNA binding protein 1	TARBP1	0,96

angiotensinogen (serpin peptidase inhibitor, clade A, member 8)	AGT	0,96
lipopolysaccharide-induced TNF factor	LITAF	0,96
polymerase (DNA directed), eta	POLH	0,96
tripartite motif containing 23	TRIM23	0,96
leucine zipper, putative tumor suppressor 1	LZTS1	0,96
sprouty RTK signaling antagonist 1	SPRY1	0,96
C-terminal binding protein 1	CTBP1	0,96
calsyntenin 1	CLSTN1	0,96
chromosome 12 open reading frame 4	C12orf4	0,96
hedgehog acyltransferase	HHAT	0,96
ligase IV, DNA, ATP-dependent	LIG4	0,96
pre-B-cell leukemia homeobox interacting protein 1	PBXIP1	0,96
chromosome 2 open reading frame 74	C2orf74	0,96
laminin, alpha 5; microRNA 4758	LAMA5; MIR4758	0,96
argininosuccinate synthase 1	ASS1	0,96
serine/threonine/tyrosine interacting-like 1	STYXL1	0,96
activated leukocyte cell adhesion molecule	ALCAM	0,96
small nuclear ribonucleoprotein D3 polypeptide	SNRPD3	0,96
zinc finger protein 404	ZNF404	0,96
sodium channel, voltage gated, type VII alpha subunit	SCN7A	0,96
coiled-coil domain containing 84	CCDC84	0,96
UDP glucuronosyltransferase 1 family, polypeptide A1; UDP glucuronosyltransferase 1 family, polypeptide A6; UDP glucuronosyltransferase 1 family, polypeptide A4; UDP glucuronosyltransferase 1 family, polypeptide A10; UDP glucuronosyltransferase 1 family, polypeptide A8; UDP glucuronosyltransferase 1 family, polypeptide A7; UDP glucuronosyltransferase 1 family, polypeptide A5; UDP glucuronosyltransferase 1 family, polypeptide A3; UDP glucuronosyltransferase 1 family, polypeptide A9	UGT1A1; UGT1A6; UGT1A4; UGT1A10; UGT1A8; UGT1A7; UGT1A5; UGT1A3; UGT1A9	0,96
ret finger protein-like 3	RFPL3	0,96
tetraspanin 14	TSPAN14	0,96
dachsous cadherin-related 1	DCHS1	0,96
deafness, autosomal recessive 59	DFNB59	0,96
glucuronidase, beta	GUSB	0,96
ribosomal protein S23	RPS23	0,96
thioredoxin-like 4A	TXNL4A	0,96
F-box protein 24	FBXO24	0,96
squalene epoxidase	SQLE	0,96
ribosomal protein S6 kinase, 90kDa, polypeptide 1	RPS6KA1	0,96
proline rich 15-like	PRR15L	0,96
ephrin-A5	EFNA5	0,95
pre-mRNA processing factor 8	PRPF8	0,95

TRAF-interacting protein with forkhead-associated domain, family member B	TIFAB	0,95
AKNA domain containing 1	AKNAD1	0,95
WW domain containing E3 ubiquitin protein ligase 2	WWP2	0,95
5-methyltetrahydrofolate-homocysteine methyltransferase reductase	MTRR	0,95
chromosome 7 open reading frame 61	C7orf61	0,95
karyopherin alpha 3 (importin alpha 4)	KPNA3	0,95
dopamine beta-hydroxylase (dopamine beta-monooxygenase)	DBH	0,95
zinc finger CCCH-type containing 12C	ZC3H12C	0,95
family with sequence similarity 210, member B	FAM210B	0,95
FPGT-TNNI3K readthrough; fucose-1-phosphate guanylyltransferase	FPGT-TNNI3K; FPGT	0,95
protein O-fucosyltransferase 2	POFUT2	0,95
ADP-ribosylation factor like GTPase 16	ARL16	0,95
PIK3CD antisense RNA 1	PIK3CD-AS1	0,95
solute carrier family 22, member 25	SLC22A25	0,95
transmembrane protein 126B	TMEM126B	0,95
HKR1, GLI-Kruppel zinc finger family member	HKR1	0,95
sirtuin 1	SIRT1	0,95
armadillo repeat containing 2	ARMC2	0,95
ANKH inorganic pyrophosphate transport regulator	ANKH	0,95
sushi domain containing 4	SUSD4	0,95
Memczak2013 ANTISENSE, CDS, coding, INTERNAL best transcript NM_003105	SORL1	0,95
zinc finger protein 780B	ZNF780B	0,95
zinc finger protein 780B	ZNF780B	0,95
nucleotide binding protein 2	NUBP2	0,95
ubiquitin specific peptidase 17-like family member 3; ubiquitin specific peptidase 17-like family member 8	USP17L3; USP17L8	0,95
Rho GTPase activating protein 27	ARHGAP27	0,95
small integral membrane protein 10	SMIM10	0,95
ATPase, class VI, type 11B	ATP11B	0,95
ubiquitin specific peptidase 24	USP24	0,95
C1q and tumor necrosis factor related protein 6	C1QTNF6	0,95
triosephosphate isomerase 1	TPI1	0,95
ELAV like neuron-specific RNA binding protein 4	ELAVL4	0,95
ets variant 2	ETV2	0,95
neuroblastoma breakpoint family, member 20	NBPF20	0,95
EPH receptor A10	EPHA10	0,95

Homo sapiens phosphoglycerate mutase 1 (brain), mRNA (cDNA clone MGC:8462 IMAGE:2821567), complete cds.; Homo sapiens phosphoglycerate mutase 1 (brain), mRNA (cDNA clone MGC:19732 IMAGE:3604026), complete cds.; Homo sapiens phosphoglycerate mutase 1 (brain), mRNA (cDNA clone MGC:15086 IMAGE:4299008), complete cds.; Homo sapiens phosphoglycerate mutase 1 (brain), mRNA (cDNA clone MGC:61519 IMAGE:5787678), complete cds.; Homo sapiens phosphoglycerate mutase 1 (brain), mRNA (cDNA clone MGC:71323 IMAGE:6584466), complete cds.; Homo sapiens phosphoglycerate mutase 1 (brain), mRNA (cDNA clone MGC:87445 IMAGE:5262935), complete cds.; Homo sapiens phosphoglycerate mutase 1 (brain), mRNA (cDNA clone MGC:88744 IMAGE:6297173), complete cds.; Homo sapiens phosphoglycerate mutase 1 (brain), mRNA (cDNA clone MGC:189702 IMAGE:8862699), complete cds.	PGAM1	0,95
diphosphoinositol pentakisphosphate kinase 2	PPIP5K2	0,95
transmembrane protein 128	TMEM128	0,95
PHD finger protein 7	PHF7	0,95
leucine zipper protein 2	LUZP2	0,95
poly(A)-specific ribonuclease	PARN	0,95
fms-related tyrosine kinase 1	FLT1	0,95
SH3-domain GRB2-like 2	SH3GL2	0,95
delta-like 2 homolog (Drosophila)	DLK2	0,95
flotillin 2	FLOT2	0,95
nuclear factor I/A	NFIA	0,95
interleukin 1 family, member 10 (theta)	IL1F10	0,95
growth regulation by estrogen in breast cancer 1	GREB1	0,95
Dab, mitogen-responsive phosphoprotein, homolog 2 (Drosophila)	DAB2	0,95
chymotrypsin-like elastase family, member 3A	CELA3A	0,95
pyridoxal-dependent decarboxylase domain containing 2, pseudogene	PDXDC2P	0,95
G protein-coupled receptor 182	GPR182	0,95
solute carrier family 39 (zinc transporter), member 7	SLC39A7	0,95
zinc finger protein 707	ZNF707	0,95
surfeit 4	SURF4	0,95
regulator of G-protein signaling 12	RGS12	0,95
H1 histone family, member 0	H1F0	0,95
alpha kinase 1	ALPK1	0,95
interleukin 6 signal transducer	IL6ST	0,95
transferrin receptor	TFRC	0,95
zinc finger protein 98	ZNF98	0,95
lipoprotein, Lp(a)	LPA	0,95
exportin 7	XPO7	0,95

atlastin GTPase 2	ATL2	0,95
breast carcinoma amplified sequence 4	BCAS4	0,95
ribosomal protein L7a	RPL7A	0,95
uncharacterized 100631378; Transcript Identified by AceView, Entrez Gene ID(s) 728853; novel transcript	LOC100631378; AC016582.2; LOC728853	0,95
UDP-N-acetylglucosamine pyrophosphorylase 1 like 1	UAP1L1	0,95
C-type lectin domain family 12, member B	CLEC12B	0,95
cellular retinoic acid binding protein 1	CRABP1	0,95
family with sequence similarity 53, member C	FAM53C	0,95
solute carrier family 39 (zinc transporter), member 5	SLC39A5	0,95
heparan sulfate (glucosamine) 3-O-sulfotransferase 1	HS3ST1	0,95
cyclin G2	CCNG2	0,95
penta-EF-hand domain containing 1	PEF1	0,95
zinc finger protein 354B	ZNF354B	0,95
myocyte enhancer factor 2D	MEF2D	0,95
forkhead box C1	FOXC1	0,95
olfactory receptor, family 2, subfamily T, member 34	OR2T34	0,95
glucose-6-phosphate isomerase	GPI	0,95
GTP binding protein 2	GTPBP2	0,95
SPARC related modular calcium binding 1	SMOC1	0,95
Memczak2013 ALT_ACCEPTOR, ALT_DONOR, coding, INTERNAL, intronic best transcript NM_018717	MAML3	0,95
speedy/RINGO cell cycle regulator family member E2; speedy/RINGO cell cycle regulator family member E2B	SPDYE2; SPDYE2B	0,95
speedy/RINGO cell cycle regulator family member E2B	SPDYE2B	0,95
golgin A8 family, member R	GOLGA8R	0,95
ADAM metallopeptidase domain 17	ADAM17	0,95
kinesin family member 26B	KIF26B	0,95
R-spondin 1	RSPO1	0,95
ribosomal protein S6 kinase, 90kDa, polypeptide 1	RPS6KA1	0,95
peptidylprolyl isomerase A (cyclophilin A)	PPIA	0,95
transmembrane and tetratricopeptide repeat containing 2	TMTC2	0,95
La ribonucleoprotein domain family, member 6	LARP6	0,95
protein tyrosine phosphatase, non-receptor type 12	PTPN12	0,95
adenosine monophosphate deaminase 2	AMPD2	0,95
hedgehog acyltransferase	HHAT	0,95
transmembrane and tetratricopeptide repeat containing 3	TMTC3	0,95
chromosome 11 open reading frame 95	C11orf95	0,95
ring finger protein 224	RNF224	0,95
ubiquitin specific peptidase 20	USP20	0,95
X-prolyl aminopeptidase (aminopeptidase P) 1, soluble	XPNPEP1	0,95
chromosome 6 open reading frame 15	C6orf15	0,95
cytochrome b5 type B (outer mitochondrial membrane)	CYB5B	0,95

X-prolyl aminopeptidase (aminopeptidase P) 2, membrane-bound	XPNPEP2	0,95
selectin P (granule membrane protein 140kDa, antigen CD62)	SELP	0,95
survival of motor neuron 1, telomeric	SMN1	0,95
Transcript Identified by AceView, Entrez Gene ID(s) 2891	GRIA2	0,95
chromosome 5 open reading frame 24	C5orf24	0,95
SEC14-like lipid binding 5	SEC14L5	0,95
SR-related CTD-associated factor 8; T-cell lymphoma invasion and metastasis 2	SCAF8; TIAM2	0,95
variable charge, X-linked; variable charge, X-linked 3A	VCX; VCX3A	0,95
Rho GTPase activating protein 11B	ARHGAP11B	0,95
lin-7 homolog C (C. elegans)	LIN7C	0,95
PHD finger protein 20-like 1	PHF20L1	0,95
staufen double-stranded RNA binding protein 1	STAU1	0,95
zinc finger, CCHC domain containing 2	ZCCHC2	0,95
Zhang2013 ALT_DONOR, coding, INTERNAL, intronic, OVERLAPTX, OVEXON best transcript NM_016648	LARP7	0,95
diacylglycerol kinase, delta 130kDa	DGKD	0,95
TBC1 domain family, member 5	TBC1D5	0,95
family with sequence similarity 149, member A	FAM149A	0,95
fibroblast growth factor receptor-like 1	FGFRL1	0,95
dermokine	DMKN	0,95
retinol binding protein 2, cellular	RBP2	0,95
family with sequence similarity 198, member B	FAM198B	0,95
ubiquitin conjugating enzyme E2 variant 2	UBE2V2	0,95
peripherin 2 (retinal degeneration, slow)	PRPH2	0,95
PR domain containing 8	PRDM8	0,95
saccharopine dehydrogenase (putative)	SCCPDH	0,95
tropomyosin 4	TPM4	0,95
ubiquitin conjugating enzyme E2B	UBE2B	0,95
zinc finger, SWIM-type containing 3	ZSWIM3	0,95
ubiquitin specific peptidase 3	USP3	0,95
T-cell leukemia/lymphoma 1B; T-cell leukemia/lymphoma 6 (non-protein coding)	TCL1B; TCL6	0,95
Yip1 domain family member 6	YIPF6	0,95
TP53RK binding protein	TPRKB	0,95
Sec23 homolog A, COPII coat complex component	SEC23A	0,95
IQ motif containing F5 pseudogene; Transcript Identified by AceView, Entrez Gene ID(s) 401067	IQCF4; IQCF3	0,95
adrenoceptor beta 2, surface	ADRB2	0,95
arginine/serine-rich coiled-coil 2	RSRC2	0,95
Zhang2013 ALT_ACCEPTOR, ALT_DONOR, coding, INTERNAL, intronic best transcript NM_030653	DDX11	0,95
makorin ring finger protein 3	MKRN3	0,95
acyl-CoA dehydrogenase, short/branched chain	ACADSB	0,95

kinase insert domain receptor	KDR	0,95
forkhead box J1	FOXJ1	0,95
fibrillarin	FBL	0,95
DEAD (Asp-Glu-Ala-Asp) box helicase 6	DDX6	0,95
interferon-induced protein with tetratricopeptide repeats 2	IFIT2	0,95
chromosome 1 open reading frame 56	C1orf56	0,95
guanine nucleotide binding protein-like 3 (nucleolar); small nucleolar RNA, C/D box 19B	GNL3; SNORD19B	0,95
RNA binding motif protein 45	RBM45	0,95
chromosome 6 open reading frame 106	C6orf106	0,95
cathepsin B	CTSB	0,95
MAP-kinase activating death domain	MADD	0,95
small nucleolar RNA, H/ACA box 17A; small nucleolar RNA, H/ACA box 17B; small nucleolar RNA host gene 7	SNORA17A; SNORA17B; SNHG7	0,95
target of myb1 membrane trafficking protein	TOM1	0,95
transmembrane protein 108	TMEM108	0,95
PHD finger protein 21A	PHF21A	0,95
crystallin alpha A	CRYAA	0,95
O-sialoglycoprotein endopeptidase-like 1	OSGEPL1	0,95
zinc finger protein 264	ZNF264	0,95
histone cluster 1, H4f	HIST1H4F	0,95
FERM, ARH/RhoGEF and pleckstrin domain protein 1; microRNA 3170	FARP1; MIR3170	0,95
gamma-aminobutyric acid (GABA) A receptor, alpha 6	GABRA6	0,95
chromosome 19 open reading frame 47	C19orf47	0,95
palmitoyl-protein thioesterase 2; EGF-like-domain, multiple 8; PPT2-EGFL8 readthrough (NMD candidate)	PPT2; EGFL8; PPT2-EGFL8	0,95
cyclin I	CCNI	0,95
SRY box 7	SOX7	0,95
transmembrane protein 259	TMEM259	0,95
serine incorporator 4	SERINC4	0,95
mitogen-activated protein kinase 8 interacting protein 2	MAPK8IP2	0,95
zinc finger protein 740	ZNF740	0,95
tumor protein p53 binding protein 2	TP53BP2	0,95
small glutamine-rich tetratricopeptide repeat (TPR)-containing, alpha	SGTA	0,95
kelch-like family member 33	KLHL33	0,95
zinc finger protein 248	ZNF248	0,95
phosphoserine phosphatase	PSPH	0,95
PAK1 interacting protein 1	PAK1IP1	0,95
ATP citrate lyase	ACLY	0,95
cannabinoid receptor 2	CNR2	0,95
zinc finger protein 384	ZNF384	0,95
lymphocyte antigen 6 complex, locus E	LY6E	0,95

histone cluster 1, H2ak	HIST1H2AK	0,95
claspin	CLSPN	0,95
chromosome 14 open reading frame 178	C14orf178	0,95
cilia and flagella associated protein 45	CFAP45	0,95
ras homolog family member V	RHOV	0,95
cysteinyl leukotriene receptor 2	CYSLTR2	0,95
solute carrier family 16, member 12	SLC16A12	0,95
matrix metallopeptidase 28	MMP28	0,95
nuclear factor I/B	NFIB	0,95
oligonucleotide/oligosaccharide-binding fold containing 1	OBFC1	0,95
Homo sapiens testis specific protein, Y-linked 3, mRNA (cDNA clone MGC:103998 IMAGE:30915406), complete cds.; Homo sapiens similar to testis specific protein, Y-linked 1, mRNA (cDNA clone MGC:150413 IMAGE:40120433), complete cds.; Homo sapiens testis specific protein, Y-linked 3, mRNA (cDNA clone MGC:150414 IMAGE:40120435), complete cds.	TSPY3; LOC728395	0,95
eukaryotic elongation factor 2 lysine methyltransferase	EEF2KMT	0,95
zinc finger, DHHC-type containing 2	ZDHHC2	0,95
olfactory receptor, family 2, subfamily T, member 4	OR2T4	0,95
DEAD (Asp-Glu-Ala-Asp) box helicase 1	DDX1	0,95
leucine-rich repeat containing G protein-coupled receptor 5	LGR5	0,95
keratin 40, type I	KRT40	0,95
heparan-alpha-glucosaminide N-acetyltransferase	HGSNAT	0,95
caspase recruitment domain family, member 18	CARD18	0,95
late cornified envelope 5A	LCE5A	0,95
male-enhanced antigen 1	MEA1	0,95
Morf4 family associated protein 1-like 1	MRFAP1L1	0,95
potassium channel, voltage gated shaker related subfamily A, member 2	KCNA2	0,95
transducin-like enhancer of split 4	TLE4	0,95
MAS-related GPR, member X4	MRGPRX4	0,95
elongation factor Tu GTP binding domain containing 2	EFTUD2	0,95
deoxyribonuclease I-like 2	DNASE1L2	0,95
S100 calcium binding protein A10	S100A10	0,95
keratin associated protein 9-2	KRTAP9-2	0,95
fumarylacetoacetate hydrolase domain containing 1	FAHD1	0,95
ATPase family, AAA domain containing 1	ATAD1	0,95
transmembrane protein 150B	TMEM150B	0,95
enoyl-CoA hydratase domain containing 3	ECHDC3	0,95
mediator complex subunit 24; microRNA 6884	MED24; MIR6884	0,95
purine-rich element binding protein A	PURA	0,95
ribophorin I	RPN1	0,95
pipecolic acid oxidase	PIPOX	0,95
carbonic anhydrase VI	CA6	0,95

apolipoprotein D	APOD	0,95
free fatty acid receptor 4	FFAR4	0,95
chemokine (C-C motif) receptor 4	CCR4	0,95
Memczak2013 ANTISENSE, coding, INTERNAL, intronic best transcript NM_052931	SLAMF6	0,95
polymerase (DNA directed) kappa	POLK	0,95
receptor-associated protein of the synapse	RAPSN	0,95
myeloma overexpressed	MYEOV	0,95
proteasome 26S subunit, non-ATPase 11	PSMD11	0,95
Kruppel-like factor 16	KLF16	0,95
chromosome 1 open reading frame 158	C1orf158	0,95
grancalcin, EF-hand calcium binding protein	GCA	0,95
Bruton agammaglobulinemia tyrosine kinase	BTK	0,95
keratin 80, type II	KRT80	0,95
leucine-rich repeat LGI family, member 2	LGI2	0,95
family with sequence similarity 204, member A	FAM204A	0,95
acyl-CoA oxidase 2, branched chain	ACOX2	0,95
protein phosphatase 1, regulatory (inhibitor) subunit 1B	PPP1R1B	0,95
aldehyde dehydrogenase 1 family, member A1	ALDH1A1	0,95
major histocompatibility complex, class I, B	HLA-B	0,95
membrane-spanning 4-domains, subfamily A, member 5	MS4A5	0,95
kelch-like family member 30	KLHL30	0,95
lectin, mannose-binding, 1	LMAN1	0,95
RNA binding motif protein, Y-linked, family 1, member E; RNA binding motif protein, Y-linked, family 1, member B	RBMY1E; RBMY1B	0,95
solute carrier family 13 (sodium/sulfate symporter), member 1	SLC13A1	0,95
recombination activating gene 2	RAG2	0,95
SMAD family member 4	SMAD4	0,95
nucleoporin 210kDa	NUP210	0,95
spermatogenesis associated 21	SPATA21	0,95
dystrophia myotonica, WD repeat containing	DMWD	0,95
KIAA1586	KIAA1586	0,95
ubiquitin associated and SH3 domain containing B	UBASH3B	0,95
charged multivesicular body protein 1A	CHMP1A	0,95
endothelin converting enzyme-like 1	ECEL1	0,95
glycine-N-acyltransferase-like 1	GLYATL1	0,95
THAP domain containing 4	THAP4	0,95
homeobox D10	HOXD10	0,95
tetratricopeptide repeat domain 28	TTC28	0,95
transmembrane protein 182	TMEM182	0,95
Transcript Identified by AceView, Entrez Gene ID(s) 60672	MIIP	0,95
ras homolog family member A	RHOA	0,95
surfeit 1	SURF1	0,95
MORN repeat containing 4	MORN4	0,95
carnosine synthase 1	CARNS1	0,95

CDK5 regulatory subunit associated protein 1	CDK5RAP1	0,95
small nucleolar RNA, C/D box 102; small nucleolar RNA, H/ACA box 27; ribosomal protein L21	SNORD102; SNORA27; RPL21	0,95
glutathione S-transferase theta 2 (gene/pseudogene)	GSTT2	0,95
ligand dependent nuclear receptor corepressor like	LCORL	0,95
interleukin 5	IL5	0,95
polymerase (RNA) II (DNA directed) polypeptide E, 25kDa	POLR2E	0,95
complement factor H	CFH	0,95
Transcript Identified by AceView, Entrez Gene ID(s) 79868	ALG13	0,95
G protein-coupled estrogen receptor 1	GPER1	0,95
glutathione S-transferase, C-terminal domain containing	GSTCD	0,95
tripartite motif containing 37	TRIM37	0,95
serpin peptidase inhibitor, clade D (heparin cofactor), member 1	SERPIND1	0,95
tyrosine kinase, non-receptor, 2	TNK2	0,95
COMM domain containing 5	COMMD5	0,95
Cas scaffolding protein family member 4	CASS4	0,95
interferon, alpha-inducible protein 27-like 1	IFI27L1	0,95
one cut homeobox 1	ONECUT1	0,95
chondroitin sulfate proteoglycan 4 pseudogene 1, Y-linked; dynamin 1 pseudogene 24	CSPG4P1Y; DNM1P24	0,95
gamma-glutamyl carboxylase	GGCX	0,95
mitochondrial calcium uptake 1	MICU1	0,95
peptidyl arginine deiminase, type II	PADI2	0,95
SEC22 homolog B, vesicle trafficking protein (gene/pseudogene)	SEC22B	0,95
proteolipid protein 1	PLP1	0,95
carcinoembryonic antigen-related cell adhesion molecule 1 (biliary glycoprotein)	CEACAM1	0,95
HECT and RLD domain containing E3 ubiquitin protein ligase 3	HERC3	0,95
proline rich 30	PRR30	0,95
immunoglobulin superfamily containing leucine-rich repeat 2	ISLR2	0,95
low density lipoprotein receptor; microRNA 6886	LDLR; MIR6886	0,95
mitochondrial genome maintenance exonuclease 1	MGME1	0,95
NODAL modulator 1	NOMO1	0,95
solute carrier family 45, member 4	SLC45A4	0,95
nucleoredoxin-like 1	NXNL1	0,95
ENTH domain containing 2	ENTHD2	0,95
period circadian clock 2	PER2	0,95
transmembrane protein 185A	TMEM185A	0,95
mitochondrial calcium uptake 2	MICU2	0,95
testis highly expressed protein 5; novel transcript; Transcript Identified by AceView	THEG5; AC011525.4; pleysey	0,95
kelch-like family member 24	KLHL24	0,95
YdjC homolog (bacterial)	YDJC	0,95

ring finger protein 11	RNF11	0,95
G-patch domain containing 2	GPATCH2	0,95
DIM1 dimethyladenosine transferase 1 homolog	DIMT1	0,95
ST3 beta-galactoside alpha-2,3-sialyltransferase 2	ST3GAL2	0,95
structural maintenance of chromosomes 4	SMC4	0,95
ankyrin repeat and SOCS box containing 3	ASB3	0,95
ATP binding cassette subfamily B member 9	ABCB9	0,95
vesicle associated membrane protein 5	VAMP5	0,95
eukaryotic translation initiation factor 2B, subunit 4 delta, 67kDa	EIF2B4	0,95
ankyrin repeat domain 54; microRNA 658	ANKRD54; MIR658	0,95
protein C (inactivator of coagulation factors Va and VIIIa)	PROC	0,95
zinc finger, MYM-type 3	ZMYM3	0,95
trans-golgi network vesicle protein 23 homolog C (S. cerevisiae); CMT1A duplicated region transcript 4; TVP23C-CDRT4 readthrough	TVP23C; CDRT4; TVP23C-CDRT4	0,95
coagulation factor II (thrombin) receptor-like 3	F2RL3	0,95
family with sequence similarity 167, member A	FAM167A	0,95
transformer 2 beta homolog (Drosophila)	TRA2B	0,95
involucrin	IVL	0,95
tripartite motif containing 16	TRIM16	0,95
kinesin family member 9	KIF9	0,95
lysophosphatidic acid receptor 4	LPAR4	0,95
Transcript Identified by AceView, Entrez Gene ID(s) 55040	EPN3	0,95
TNF receptor-associated protein 1	TRAP1	0,95
DEAH (Asp-Glu-Ala-His) box polypeptide 34	DHX34	0,95
solute carrier family 9, subfamily A (NHE3, cation proton antiporter 3), member 3 regulator 1; microRNA 3615	SLC9A3R1; MIR3615	0,95
zinc finger, FYVE domain containing 28	ZFYVE28	0,95
CD96 molecule	CD96	0,95
G protein-coupled receptor 12	GPR12	0,95
BTB (POZ) domain containing 17	BTBD17	0,95
ubiquinol-cytochrome c reductase hinge protein	UQCRH	0,95
cms1 ribosomal small subunit homolog (yeast)	CMSS1	0,95
C-x(9)-C motif containing 1	CMC1	0,95
ssu-2 homolog (C. elegans)	SSUH2	0,95
protein tyrosine phosphatase, receptor type, H	PTPRH	0,95
reticulon 2	RTN2	0,95
chromosome 17 open reading frame 78	C17orf78	0,95
keratin associated protein 4-2	KRTAP4-2	0,95
family with sequence similarity 122A	FAM122A	0,95
interleukin 1 receptor, type I	IL1R1	0,95
WD repeat domain 6	WDR6	0,95
BCL2-like 14 (apoptosis facilitator)	BCL2L14	0,95

regulator of G-protein signaling 8	RGS8	0,95
cadherin 9, type 2 (T1-cadherin)	CDH9	0,95
ariadne homolog 2 opposite strand	ARIH2OS	0,95
poliovirus receptor	PVR	0,95
Memczak2013 ANTISENSE, coding, INTERNAL, intronic best transcript NM_001146694	KDM4C	0,95
leucine rich repeat transmembrane neuronal 3	LRRTM3	0,95
Wolf-Hirschhorn syndrome candidate 1-like 1	WHSC1L1	0,95
N-myristoyltransferase 1	NMT1	0,95
Spi-B transcription factor (Spi-1/PU.1 related)	SPIB	0,95
HECT, UBA and WWE domain containing 1, E3 ubiquitin protein ligase	HUWE1	0,95
ephrin-B3	EFNB3	0,95
sonic hedgehog	SHH	0,95
CKLF-like MARVEL transmembrane domain containing 8	CMTM8	0,95
solute carrier family 25, member 42	SLC25A42	0,95
CD28 molecule	CD28	0,95
NAD synthetase 1	NADSYN1	0,95
zinc finger protein 181	ZNF181	0,95
abhydrolase domain containing 17C	ABHD17C	0,95
fibrillarin	FBL	0,95
dual specificity phosphatase 9	DUSP9	0,95
heparan sulfate 6-O-sulfotransferase 2	HS6ST2	0,95
ribosomal protein S29; ribosomal protein L32 pseudogene 29	RPS29; RPL32P29	0,95
FK506 binding protein 1A; microRNA 6869	FKBP1A; MIR6869	0,95
cactin, spliceosome C complex subunit	CACTIN	0,95
uncharacterized LOC389602; novel transcript	LOC389602; AC021218.2	0,95
ganglioside induced differentiation associated protein 2	GDAP2	0,95
zinc finger protein 41	ZNF41	0,95
THO complex 2	THOC2	0,95
serine/threonine kinase 19	STK19	0,95
solute carrier family 25 (mitochondrial carrier; ornithine transporter) member 15; microRNA 621	SLC25A15; MIR621	0,95
G-protein signaling modulator 1	GPSM1	0,95
actin gamma 1	ACTG1	0,95
KH domain containing, RNA binding, signal transduction associated 2	KHDRBS2	0,95
thioredoxin domain containing 12 (endoplasmic reticulum); KTI12 chromatin associated homolog	TXNDC12; KTI12	0,95
ADAM metallopeptidase domain 9	ADAM9	0,95
kinesin family member 12	KIF12	0,95

glucosaminyl (N-acetyl) transferase 6 [Source:HGNC Symbol;Acc:HGNC:21623]	GCNT6	0,95
polycomb group ring finger 5	PCGF5	0,95
formyl peptide receptor 3	FPR3	0,95
transcription elongation factor A (SII), 2	TCEA2	0,95
Ras-related associated with diabetes	RRAD	0,95
myosin light chain 6	MYL6	0,95
membrane-spanning 4-domains, subfamily A, member 3 (hematopoietic cell-specific)	MS4A3	0,95
PAN2 poly(A) specific ribonuclease subunit; canopy FGF signaling regulator 2	PAN2; CNPY2	0,95
Memczak2013 ALT_ACCEPTOR, ALT_DONOR, coding, INTERNAL, intronic best transcript NM_022172	PC	0,95
uncharacterized LOC400553; novel transcript	LOC400553; RP11-863P13.4	0,95
chromosome 12 open reading frame 56	C12orf56	0,95
tripartite motif containing 54	TRIM54	0,95
tripartite motif-containing 51	TRIM51	0,95
pleckstrin homology domain containing, family S member 1	PLEKHS1	0,95
THO complex 3	THOC3	0,95
growth differentiation factor 6	GDF6	0,95
ribosomal protein L11	RPL11	0,95
diacylglycerol kinase, delta 130kDa	DGKD	0,95
H2A histone family, member X	H2AFX	0,95
chromosome 9 open reading frame 135	C9orf135	0,95
diphthamide biosynthesis 3 pseudogene 1	DPH3P1	0,95
syndecan binding protein	SDCBP	0,95
interleukin 12 receptor, beta 1	IL12RB1	0,95
adenosine deaminase-like	ADAL	0,95
eukaryotic translation initiation factor 3, subunit I	EIF3I	0,95
nuclear factor related to kappaB binding protein	NFRKB	0,95
DDB1 and CUL4 associated factor 12	DCAF12	0,95
sushi domain containing 6	SUSD6	0,95
LY6/PLAUR domain containing 6	LYPD6	0,95
peptidyl arginine deiminase, type III	PADI3	0,95
calcium channel flower domain containing 1	CACFD1	0,95
aldo-keto reductase family 7, member A3 (aflatoxin aldehyde reductase)	AKR7A3	0,95
Rho guanine nucleotide exchange factor 38	ARHGEF38	0,95
insulin receptor substrate 1	IRS1	0,95
Rho GDP dissociation inhibitor (GDI) alpha	ARHGDIA	0,95
nicotinamide nucleotide adenylyltransferase 3	NMNAT3	0,95
cell cycle and apoptosis regulator 2	CCAR2	0,95

ZNF559-ZNF177 readthrough; zinc finger protein 177; zinc finger protein 559	ZNF559-ZNF177; ZNF177; ZNF559	0,95
odd-skipped related transciption factor 1	OSR1	0,95
zinc finger protein 33B	ZNF33B	0,95
carbohydrate (N-acetylglucosamine 6-O) sulfotransferase 7	CHST7	0,95
excision repair cross-complementation group 1	ERCC1	0,95
thymosin beta 10	TMSB10	0,95
olfactory receptor, family 10, subfamily C, member 1 (gene/pseudogene)	OR10C1	0,95
sterol regulatory element binding transcription factor 2	SREBF2	0,95
Ras-related GTP binding D	RRAGD	0,95
homeobox B8	HOXB8	0,95
activating signal cointegrator 1 complex subunit 3	ASCC3	0,95
chromosome 5 open reading frame 56	C5orf56	0,95
Transcript Identified by AceView, Entrez Gene ID(s) 94031	HTRA3	0,95
potassium channel, voltage gated modifier subfamily F, member 1	KCNF1	0,95
interleukin 2	IL2	0,95
zinc finger, ZZ-type with EF-hand domain 1	ZZEF1	0,95
histone cluster 2, H2ac	HIST2H2AC	0,95
cholinergic receptor, nicotinic alpha 4	CHRNA4	0,95
RAB11 family interacting protein 1 (class I)	RAB11FIP1	0,95
DiGeorge syndrome critical region gene 6-like	DGCR6L	0,95
CD53 molecule	CD53	0,95
kinetochore-localized astrin/SPAG5 binding protein	KNSTRN	0,95
armadillo repeat containing 3	ARMC3	0,95
transglutaminase 5	TGM5	0,95
AKT1 substrate 1 (proline rich)	AKT1S1	0,95
ADP-ribosylation factor like GTPase 13A	ARL13A	0,95
signal recognition particle 9kDa	SRP9	0,95
molybdenum cofactor synthesis 3	MOCS3	0,95
dihydrolipoamide S-succinyltransferase (E2 component of 2-oxo-glutarate complex)	DLST	0,95
tumor necrosis factor receptor superfamily, member 11b	TNFRSF11B	0,95
scratch family zinc finger 1	SCRT1	0,95
vezatin, adherens junctions transmembrane protein	VEZT	0,95
sulfotransferase family 1A member 1	SULT1A1	0,95
Transcript Identified by AceView, Entrez Gene ID(s) 8850	KAT2B	0,95
guanine nucleotide binding protein (G protein), gamma transducing activity polypeptide 2	GNGT2	0,95
ectonucleoside triphosphate diphosphohydrolase 6 (putative)	ENTPD6	0,95
zinc finger protein 2	ZNF2	0,95
neuroblastoma breakpoint family, member 15	NBPF15	0,95

eukaryotic translation initiation factor 4H	EIF4H	0,95
cyclin H	CCNH	0,95
family with sequence similarity 231, member C; family with sequence similarity 231, member A	FAM231C; FAM231A	0,95
chromatin licensing and DNA replication factor 1	CDT1	0,95
ubiquitin-conjugating enzyme E2, J1	UBE2J1	0,95
ST20-MTHFS readthrough	ST20-MTHFS	0,95
prostaglandin reductase 2	PTGR2	0,95
family with sequence similarity 71, member F2	FAM71F2	0,95
integrin beta 1	ITGB1	0,95
ankyrin repeat domain 20 family, member A3; ankyrin repeat domain 20 family, member A2	ANKRD20A3; ANKRD20A2	0,95
group-specific component (vitamin D binding protein)	GC	0,95
cardiomyopathy associated 5	CMYA5	0,95
TIMELESS interacting protein; ribosomal protein L9 pseudogene 25	TIPIN; RPL9P25	0,95
zonadhesin (gene/pseudogene)	ZAN	0,95
fibroblast growth factor 1 (acidic)	FGF1	0,95
radial spoke head 9 homolog (Chlamydomonas)	RSPH9	0,95
minichromosome maintenance 8 homologous recombination repair factor	MCM8	0,95
ASH1L antisense RNA 1	ASH1L-AS1	0,95
histone cluster 1, H1t	HIST1H1T	0,95
plasmalemma vesicle associated protein	PLVAP	0,95
tripartite motif containing 6	TRIM6	0,95
taste receptor, type 2, member 38	TAS2R38	0,95
glycoprotein A33 (transmembrane)	GPA33	0,95
microtubule associated tumor suppressor candidate 2	MTUS2	0,95
de-etiolated homolog 1 (Arabidopsis)	DET1	0,95
nucleoporin 107kDa	NUP107	0,95
mitogen-activated protein kinase kinase kinase 10	MAP3K10	0,95
ELF1 homolog, elongation factor 1	ELOF1	0,95
notum pectinacetylesterase homolog (Drosophila)	NOTUM	0,95
geminin, DNA replication inhibitor	GMNN	0,95
aprataxin	APTX	0,95
zinc finger protein 468	ZNF468	0,95
sodium channel, voltage gated, type II beta subunit	SCN2B	0,95
toll-like receptor 4	TLR4	0,95
tudor domain containing 1	TDRD1	0,95
regulator of chromosome condensation 2	RCC2	0,95
chemokine (C-C motif) receptor 10	CCR10	0,95
FAT atypical cadherin 1	FAT1	0,95
Down syndrome critical region 3	DSCR3	0,95
SCY1-like, kinase-like 2	SCYL2	0,95
TBC1 domain family, member 13	TBC1D13	0,95

cell division cycle associated 2	CDCA2	0,95
transmembrane protein 167B	TMEM167B	0,95
protein kinase, cAMP-dependent, regulatory, type I, beta	PRKAR1B	0,95
guanine nucleotide binding protein (G protein), gamma 12	GNG12	0,95
keratin associated protein 13-1	KRTAP13-1	0,95
scaffolding protein involved in DNA repair	SPIDR	0,95
pumilio RNA binding family member 2	PUM2	0,95
basic charge, Y-linked, 2C; basic charge, Y-linked, 2	BPY2C; BPY2	0,95
kelch-like family member 6	KLHL6	0,95
major facilitator superfamily domain containing 13A	MFSD13A	0,95
WD repeat domain 12	WDR12	0,95
golgi-associated, olfactory signaling regulator	GFY	0,95
family with sequence similarity 19 (chemokine (C-C motif)-like), member A3	FAM19A3	0,95
R3H domain containing 2	R3HDM2	0,95
serglycin	SRGN	0,95
G protein-coupled receptor 75	GPR75	0,95
WD repeat domain 36	WDR36	0,95
annexin A13	ANXA13	0,95
splicing factor 3b subunit 3	SF3B3	0,95
small nucleolar RNA, C/D box 89; ring finger protein 149	SNORD89; RNF149	0,95
torsin A interacting protein 2	TOR1AIP2	0,95
immediate early response 5-like	IER5L	0,95
branched chain ketoacid dehydrogenase kinase	BCKDK	0,95
transformation/transcription domain-associated protein	TRRAP	0,95
Yae1 domain containing 1	YAE1D1	0,95
SURP and G-patch domain containing 2	SUGP2	0,95
Jeck2013 ALT_ACCEPTOR, ALT_DONOR, coding, INTERNAL, intronic best transcript NM_057159; Salzman2013 ALT_ACCEPTOR, ALT_DONOR, coding, INTERNAL, intronic best transcript NM_057159	LPAR1	0,95
CD79a molecule, immunoglobulin-associated alpha	CD79A	0,95
AT rich interactive domain 1A (SWI-like)	ARID1A	0,95
DEAH (Asp-Glu-Ala-His) box helicase 9	DHX9	0,95
Memczak2013 ANTISENSE, CDS, coding, INTERNAL best transcript NM_033133	CNP	0,95
transmembrane protein 201	TMEM201	0,95
DDRGK domain containing 1	DDRGK1	0,95
mitochondrial ribosomal protein L51	MRPL51	0,95
pleckstrin homology domain containing, family B (evectins) member 2	PLEKHB2	0,95
immunoglobulin superfamily, member 9B	IGSF9B	0,95
ribosomal protein L14	RPL14	0,95
PR domain containing 10	PRDM10	0,95

regulator of G-protein signaling 19	RGS19	0,95
SH3-domain GRB2-like 1	SH3GL1	0,95
EP300 interacting inhibitor of differentiation 3	EID3	0,95
Ca++-dependent secretion activator	CADPS	0,95
POU class 3 homeobox 2	POU3F2	0,95
Transcript Identified by AceView, Entrez Gene ID(s) 4591	TRIM37	0,95
keratin associated protein 12-4	KRTAP12-4	0,95
prostaglandin reductase 1	PTGR1	0,95
neuralized E3 ubiquitin protein ligase 2	NEURL2	0,95
Transcript Identified by AceView, Entrez Gene ID(s) 8660	IRS2	0,95
oncomodulin	OCM	0,95
microtubule-associated protein 10	MAP10	0,95
signal transducer and activator of transcription 6, interleukin-4 induced	STAT6	0,95
zinc finger protein 512B	ZNF512B	0,95
SNF related kinase	SNRK	0,95
BAI1-associated protein 2	BAIAP2	0,95
nudix hydrolase 5	NUDT5	0,95
copine VIII	CPNE8	0,95
P antigen family, member 2B	PAGE2B	0,95
epidermal growth factor receptor pathway substrate 15	EPS15	0,95
developmentally regulated GTP binding protein 2	DRG2	0,95
ring finger protein 126	RNF126	0,95
dpy-19-like 2 (C. elegans)	DPY19L2	0,95
ubiquitin specific peptidase 46	USP46	0,95
zinc finger with KRAB and SCAN domains 7	ZKSCAN7	0,95
zinc finger, DHHC-type containing 6	ZDHHC6	0,95
cytoskeleton-associated protein 4	CKAP4	0,95
TROVE domain family, member 2	TROVE2	0,95
ATP synthase, H+ transporting, mitochondrial F1 complex, gamma polypeptide 1	ATP5C1	0,95
family with sequence similarity 98, member B	FAM98B	0,95
thyroid hormone receptor, beta	THRB	0,95
olfactory receptor, family 4, subfamily B, member 1	OR4B1	0,95
histone cluster 1, H2bh	HIST1H2BH	0,95
LIM and senescent cell antigen-like domains 3-like; LIM and senescent cell antigen-like domains 3	LIMS3L; LIMS3	0,95
mannose receptor, C type 2	MRC2	0,95
proline rich 26	PRR26	0,95
hdc homolog, cell cycle regulator	HECA	0,95
prenyl (decaprenyl) diphosphate synthase, subunit 2	PDSS2	0,95
MNAT CDK-activating kinase assembly factor 1	MNAT1	0,95
transmembrane 9 superfamily member 1	TM9SF1	0,95
forkhead box D1	FOXD1	0,95
ARP6 actin-related protein 6 homolog (yeast)	ACTR6	0,95

zinc finger protein 660	ZNF660	0,95
NLR family, CARD domain containing 5	NLRC5	0,95
H2A histone family, member B2	H2AFB2	0,95
solute carrier family 16 (monocarboxylate transporter), member 1	SLC16A1	0,95
interleukin 12B	IL12B	0,95
SLIT and NTRK-like family, member 1	SLITRK1	0,95
solute carrier family 37 (glucose-6-phosphate transporter), member 2	SLC37A2	0,95
U2 small nuclear RNA auxiliary factor 1	U2AF1	0,95
kinesin family member 1C	KIF1C	0,95
RNA binding motif protein 34	RBM34	0,95
glutamate receptor, metabotropic 2	GRM2	0,95
PERP, TP53 apoptosis effector	PERP	0,95
growth differentiation factor 11	GDF11	0,95
ninein (GSK3B interacting protein)	NIN	0,95
Fc fragment of IgG binding protein	FCGBP	0,95
chromosome 11 open reading frame 94	C11orf94	0,95
interferon, alpha 17	IFNA17	0,95
ATP synthase, H+ transporting, mitochondrial Fo complex subunit B1	ATP5F1	0,95
Vpr (HIV-1) binding protein	VPRBP	0,94
DEAD (Asp-Glu-Ala-Asp) box helicase 3, Y-linked	DDX3Y	0,94
interferon regulatory factor 9	IRF9	0,94
leucine rich repeat containing 66	LRRC66	0,94
hydroxymethylbilane synthase	HMBS	0,94
CKLF-like MARVEL transmembrane domain containing 5	CMTM5	0,94
matrix metallopeptidase 15 (membrane-inserted)	MMP15	0,94
signal recognition particle receptor (docking protein)	SRPR	0,94
methyltransferase like 15	METTL15	0,94
Transcript Identified by AceView, Entrez Gene ID(s) 10659	CELF2	0,94
abhydrolase domain containing 12	ABHD12	0,94
KIAA1191	KIAA1191	0,94
dachshund family transcription factor 2	DACH2	0,94
neuroblastoma breakpoint family, member 6; neuroblastoma breakpoint family, member 5, pseudogene	NBPF6; NBPF5P	0,94
adenosine deaminase, RNA-specific	ADAR	0,94
MINOS1-NBL1 readthrough	MINOS1-NBL1	0,94
ELOVL fatty acid elongase 2	ELOVL2	0,94
proteasome subunit beta 4	PSMB4	0,94
F-box and leucine-rich repeat protein 5	FBXL5	0,94
SH2 domain containing 7	SH2D7	0,94
rhophilin associated tail protein 1-like	ROPN1L	0,94
alanyl-tRNA synthetase 2, mitochondrial	AARS2	0,94
dopamine receptor D3	DRD3	0,94

ribosomal protein L36a	RPL36A	0,94
structural maintenance of chromosomes flexible hinge domain containing 1	SMCHD1	0,94
atlastin GTPase 3	ATL3	0,94
sirtuin 4	SIRT4	0,94
formin like 3	FMNL3	0,94
DDB1 and CUL4 associated factor 10	DCAF10	0,94
paraspeckle component 1	PSPC1	0,94
ankyrin repeat domain 29	ANKRD29	0,94
cancer susceptibility candidate 3; microRNA 6866	CASC3; MIR6866	0,94
gem nuclear organelle associated protein 4	GEMIN4	0,94
cytoskeleton associated protein 5; small nucleolar RNA, C/D box 67	CKAP5; SNORD67	0,94
transmembrane 6 superfamily member 1	TM6SF1	0,94
TAF6 RNA polymerase II, TATA box binding protein (TBP)-associated factor, 80kDa	TAF6	0,94
MAD2 mitotic arrest deficient-like 2 (yeast)	MAD2L2	0,94
ring finger protein 139	RNF139	0,94
ArfGAP with GTPase domain, ankyrin repeat and PH domain 2	AGAP2	0,94
patched 1	PTCH1	0,94
calcium/calmodulin-dependent protein kinase II beta	CAMK2B	0,94
ectonucleoside triphosphate diphosphohydrolase 4; lysyl oxidase-like 2	ENTPD4; LOXL2	0,94
solute carrier family 35, member E3	SLC35E3	0,94
metadherin	MTDH	0,94
coatomer protein complex subunit epsilon	COPE	0,94
prickle homolog 2	PRICKLE2	0,94
transferrin receptor 2	TFR2	0,94
defensin, alpha 5, Paneth cell-specific	DEFA5	0,94
carbonic anhydrase XI	CA11	0,94
calcium channel, voltage-dependent, beta 2 subunit	CACNB2	0,94
keratin associated protein 10-5	KRTAP10-5	0,94
potassium channel, voltage gated shaker related subfamily A, member 6; polypeptide N-acetylgalactosaminyltransferase 8	KCNA6; GALNT8	0,94
BRO1 domain and CAAX motif containing	BROX	0,94
Salzman2013 ANTISENSE, CDS, coding, INTERNAL, intronic, OVCODE, OVERLAPTX, OVEXON, UTR3 best transcript NM_015178; novel transcript, antisense to RHOBTB2	RP11-875O11.1; RHOBTB2	0,94
KIAA1549	KIAA1549	0,94
hydroxysteroid dehydrogenase like 2	HSDL2	0,94
Transcript Identified by AceView, Entrez Gene ID(s) 79658	ARHGAP10	0,94
mitofusin 2	MFN2	0,94
transcription elongation factor A (SII) N-terminal and central domain containing 2; microRNA 4781	TCEANC2; MIR4781	0,94

Sin3A associated protein 25kDa	SAP25	0,94
protein tyrosine phosphatase, non-receptor type 23	PTPN23	0,94
tonsoku-like, DNA repair protein	TONSL	0,94
INO80B-WBP1 readthrough (NMD candidate)	INO80B-WBP1	0,94
serpin peptidase inhibitor, clade A (alpha-1 antiproteinase, antitrypsin), member 13, pseudogene	SERPINA13P	0,94
mitogen-activated protein kinase kinase kinase kinase 5	MAP4K5	0,94
keratin 72, type II	KRT72	0,94
cancer/testis antigen family 47, member A5; cancer/testis antigen family 47, member A2; cancer/testis antigen family 47, member A1; cancer/testis antigen family 47, member A11; cancer/testis antigen family 47, member A12	CT47A5; CT47A2; CT47A1; CT47A11; CT47A12	0,94
Jeck2013 ANTISENSE, coding, INTERNAL, intronic best transcript NM_173688	NKAIN3	0,94
unc-45 myosin chaperone A	UNC45A	0,94
glyceraldehyde-3-phosphate dehydrogenase	GAPDH	0,94
COX10 heme A:farnesyltransferase cytochrome c oxidase assembly factor	COX10	0,94
apoptogenic 1, mitochondrial	APOPT1	0,94
phosphodiesterase 4C, cAMP-specific	PDE4C	0,94
zinc finger and SCAN domain containing 29	ZSCAN29	0,94
G protein-coupled receptor 39	GPR39	0,94
transmembrane protease, serine 2	TMPRSS2	0,94
cilia and flagella associated protein 73	CFAP73	0,94
dedicator of cytokinesis 2	DOCK2	0,94
fatty acid binding protein 1, liver	FABP1	0,94
runt-related transcription factor 1	RUNX1	0,94
PAP associated domain containing 7	PAPD7	0,94
ornithine decarboxylase antizyme 2	OAZ2	0,94
Ral GEF with PH domain and SH3 binding motif 2	RALGPS2	0,94
amyotrophic lateral sclerosis 2 chromosome region candidate 11	ALS2CR11	0,94
zinc finger protein 227	ZNF227	0,94
paralemmin	PALM	0,94
small integral membrane protein 10 like 1	SMIM10L1	0,94
actin-related protein T2	ACTRT2	0,94
matrix metallopeptidase 3	MMP3	0,94
family with sequence similarity 126, member A	FAM126A	0,94
ubiquitin specific peptidase 34	USP34	0,94
peptidylprolyl isomerase domain and WD repeat containing 1	PPWD1	0,94
alkB homolog 5, RNA demethylase	ALKBH5	0,94
complement component 5a receptor 1	C5AR1	0,94
protocadherin-related 15	PCDH15	0,94
zinc finger protein 429	ZNF429	0,94

NIPA-like domain containing 4	NIPAL4	0,94
long intergenic non-protein coding RNA 1620	LINC01620	0,94
complement component 3a receptor 1	C3AR1	0,94
nucleoporin 153kDa	NUP153	0,94
family with sequence similarity 212, member A	FAM212A	0,94
lipin 2	LPIN2	0,94
autophagy related 4B, cysteine peptidase	ATG4B	0,94
hydroxymethylbilane synthase	HMBS	0,94
apolipoprotein L domain containing 1; DEAD (Asp-Glu-Ala-Asp) box polypeptide 47	APOLD1; DDX47	0,94
ovochymase 1	OVCH1	0,94
nuclear pore complex interacting protein family, member B4	NPIPB4	0,94
Wolfram syndrome 1 (wolframin)	WFS1	0,94
TRIO and F-actin binding protein; nucleolar protein 12	TRIOBP; NOL12	0,94
chromobox homolog 3	CBX3	0,94
chordin-like 2	CHRDL2	0,94
SYF2 pre-mRNA-splicing factor	SYF2	0,94
succinate dehydrogenase complex subunit D, integral membrane protein	SDHD	0,94
zinc and ring finger 2, E3 ubiquitin protein ligase	ZNRF2	0,94
SLAIN motif family member 1	SLAIN1	0,94
neurobeachin like 1	NBEAL1	0,94
Homo sapiens olfactory receptor, family 4, subfamily M, member 2 (OR4M2), mRNA.; Homo sapiens olfactory receptor, family 4, subfamily M, member 2, mRNA (cDNA clone MGC:168600 IMAGE:9020977), complete cds.	OR4M2; AC171558.2	0,94
RAD51 paralog B	RAD51B	0,94
kallikrein related peptidase 10	KLK10	0,94
diphthamide biosynthesis 3	DPH3	0,94
heterogeneous nuclear ribonucleoprotein L-like	HNRNPLL	0,94
insulin-degrading enzyme	IDE	0,94
eukaryotic translation initiation factor 3, subunit H	EIF3H	0,94
KIAA0930	KIAA0930	0,94
leucine rich repeat neuronal 4	LRRN4	0,94
transmembrane protein 57	TMEM57	0,94
TLR4 interactor with leucine-rich repeats	TRIL	0,94
N-acetylgalactosaminidase, alpha-	NAGA	0,94
calcium binding protein 7	CABP7	0,94
B-cell CLL/lymphoma 7C	BCL7C	0,94
tetraspanin 7	TSPAN7	0,94
cytoplasmic FMR1 interacting protein 1	CYFIP1	0,94
signal peptide peptidase like 3	SPPL3	0,94
mannosyl (alpha-1,3-)-glycoprotein beta-1,4-N-acetylglucosaminyltransferase, isozyme B	MGAT4B	0,94

solute carrier family 25 (mitochondrial carrier; adenine nucleotide translocator), member 4	SLC25A4	0,94
TELO2 interacting protein 1	TTI1	0,94
zinc finger, MYND-type containing 10	ZMYND10	0,94
oxysterol binding protein-like 3	OSBPL3	0,94
Transcript Identified by AceView, Entrez Gene ID(s) 7813	EVI5	0,94
G protein-coupled receptor 34	GPR34	0,94
betaine--homocysteine S-methyltransferase	BHMT	0,94
RALBP1 associated Eps domain containing 1	REPS1	0,94
N(alpha)-acetyltransferase 40, NatD catalytic subunit	NAA40	0,94
CD38 molecule	CD38	0,94
statherin	STATH	0,94
premature ovarian failure, 1B	POF1B	0,94
proline rich 36	PRR36	0,94
zinc finger, CCHC domain containing 14	ZCCHC14	0,94
zinc finger protein 324	ZNF324	0,94
fatty acid hydroxylase domain containing 2	FAXDC2	0,94
transmembrane protein 185B	TMEM185B	0,94
2-5-oligoadenylate synthetase-like	OASL	0,94
early B-cell factor 4	EBF4	0,94
leukocyte cell-derived chemotaxin 2	LECT2	0,94
selenoprotein M; selenoprotein M [Source:EntrezGene;Acc:140606]; Salzman2013 ANNOTATED, CDS, coding, OVCODE, OVERLAPTX, OVEXON, UTR3 best transcript NM_080430; Salzman2013 ANNOTATED, CDS, coding, OVCODE, OVERLAPTX, OVEXON, UTR3, UTR5 best transcript NM_080430; Transcript Identified by AceView, Entrez Gene ID(s) 140606	SELM	0,94
speedy/RINGO cell cycle regulator family member E1	SPDYE1	0,94
general transcription factor IIE subunit 2	GTF2E2	0,94
CD151 molecule (Raph blood group)	CD151	0,94
family with sequence similarity 181, member B	FAM181B	0,94
CLOCK-interacting pacemaker	CIPC	0,94
proline rich membrane anchor 1	PRIMA1	0,94
trans-golgi network vesicle protein 23 homolog A (S. cerevisiae)	TVP23A	0,94
small nuclear ribonucleoprotein, U5 40kDa subunit	SNRNP40	0,94
egl-9 family hypoxia-inducible factor 1	EGLN1	0,94
zinc finger and SCAN domain containing 18	ZSCAN18	0,94
pseudouridylate synthase 7-like	PUS7L	0,94
keratin associated protein 9-7	KRTAP9-7	0,94
APOBEC1 complementation factor	A1CF	0,94
pyruvate dehydrogenase phosphatase regulatory subunit	PDPR	0,94
cell adhesion associated, oncogene regulated	CDON	0,94
myosin XVA	MYO15A	0,94
syntaxin 19	STX19	0,94

CD7 molecule	CD7	0,94
agmatinase	AGMAT	0,94
WDR86 antisense RNA 1	WDR86-AS1	0,94
family with sequence similarity 3, member C	FAM3C	0,94
Transcript Identified by AceView, Entrez Gene ID(s) 401067	IQCF3	0,94
inositol monophosphatase domain containing 1	IMPAD1	0,94
apolipoprotein B mRNA editing enzyme, catalytic polypeptide-like 3D	APOBEC3D	0,94
GC-rich promoter binding protein 1-like 1	GPBP1L1	0,94
ras homolog family member C	RHOC	0,94
VPS18 CORVET/HOPS core subunit	VPS18	0,94
serine/threonine kinase 32B	STK32B	0,94
glucosaminyl (N-acetyl) transferase 2, I-branching enzyme (I blood group)	GCNT2	0,94
olfactory receptor, family 4, subfamily Q, member 3	OR4Q3	0,94
exonuclease 3-5 domain containing 1	EXD1	0,94
serine/threonine kinase 24	STK24	0,94
Cdk5 and Abl enzyme substrate 1	CABLES1	0,94
tripeptidyl peptidase I	TPP1	0,94
3-oxoacid CoA-transferase 2 pseudogene 1	OXCT2P1	0,94
Transcript Identified by AceView, Entrez Gene ID(s) 3632	INPP5A	0,94
phosphatidylinositol glycan anchor biosynthesis class W	PIGW	0,94
Transcript Identified by AceView, Entrez Gene ID(s) 9320	TRIP12	0,94
barrier to autointegration factor 1	BANF1	0,94
tachykinin 3	TAC3	0,94
inositol polyphosphate-5-phosphatase D	INPP5D	0,94
purinergic receptor P2X, ligand gated ion channel, 1	P2RX1	0,94
required for meiotic nuclear division 1 homolog	RMND1	0,94
E2F transcription factor 1	E2F1	0,94
keratin 4, type II	KRT4	0,94
zinc finger, DHHC-type containing 5	ZDHHC5	0,94
milk fat globule-EGF factor 8 protein	MFGE8	0,94
intercellular adhesion molecule 2	ICAM2	0,94
jun B proto-oncogene	JUNB	0,94
ATP13A5 antisense RNA 1	ATP13A5-AS1	0,94
FUS RNA binding protein	FUS	0,94
dysbindin (dystrobrevin binding protein 1) domain containing 2	DBNDD2	0,94
aarF domain containing kinase 3	ADCK3	0,94
testis-specific kinase 1; microRNA 4667	TESK1; MIR4667	0,94
U2 small nuclear RNA auxiliary factor 1	U2AF1	0,94
ubiquitin-conjugating enzyme E2Q family member 2	UBE2Q2	0,94
digestive organ expansion factor homolog (zebrafish)	DIEXF	0,94
nuclear factor of activated T-cells, cytoplasmic, calcineurin-dependent 2 interacting protein; microRNA 4517	NFATC2IP; MIR4517	0,94

vasoactive intestinal peptide	VIP	0,94
docking protein 2	DOK2	0,94
fibronectin type III domain containing 5	FNDC5	0,94
zinc finger protein 585A	ZNF585A	0,94
WD repeat domain 93	WDR93	0,94
activity-regulated cytoskeleton-associated protein	ARC	0,94
complement component 1, r subcomponent-like	C1RL	0,94
interleukin enhancer binding factor 2	ILF2	0,94
formin 1	FMN1	0,94
non-POU domain containing, octamer-binding	NONO	0,94
secretoglobin, family 1D, member 1	SCGB1D1	0,94
EBNA1 binding protein 2	EBNA1BP2	0,94
stem-loop binding protein	SLBP	0,94
transmembrane protein 30C	TMEM30C	0,94
RAN binding protein 1	RANBP1	0,94
KIAA0368	KIAA0368	0,94
zinc finger protein 443	ZNF443	0,94
crystallin gamma A	CRYGA	0,94
ankyrin repeat domain 63	ANKRD63	0,94
1-acylglycerol-3-phosphate O-acyltransferase 1; microRNA 6721	AGPAT1; MIR6721	0,94
chromosome 2 open reading frame 42	C2orf42	0,94
defensin, beta 1	DEFB1	0,94
xenotropic and polytropic retrovirus receptor 1	XPR1	0,94
myosin regulatory light chain interacting protein; microRNA 4639	MYLIP; MIR4639	0,94
tetraspanin 17	TSPAN17	0,94
UDP glucuronosyltransferase 2 family, polypeptide A3	UGT2A3	0,94
EPH receptor B4	EPHB4	0,94
Memczak2013 ANTISENSE, coding, INTERNAL, UTR3 best transcript NM_130847	AMOTL1	0,94
transmembrane and coiled-coil domain family 1	TMCC1	0,94
RIC8 guanine nucleotide exchange factor B	RIC8B	0,94
pyrimidinergic receptor P2Y, G-protein coupled, 6	P2RY6	0,94
chromosome 1 open reading frame 204	C1orf204	0,94
cathepsin O	CTSO	0,94
N-acetylated alpha-linked acidic dipeptidase-like 1	NAALADL1	0,94
reprimo-like	RPRML	0,94
zinc finger, HIT-type containing 6	ZNHIT6	0,94
discs, large (Drosophila) homolog-associated protein 1	DLGAP1	0,94
COMM domain containing 10	COMMD10	0,94
paraoxonase 3	PON3	0,94
mutL homolog 1	MLH1	0,94
histone cluster 1, H2ai	HIST1H2AI	0,94
fibronectin 1	FN1	0,94

histamine receptor H1	HRH1	0,94
killin, p53-regulated DNA replication inhibitor	KLLN	0,94
TOPORS antisense RNA 1; GVQW motif containing 1	TOPORS-AS1; GVQW1	0,94
docking protein 5	DOK5	0,94
olfactory receptor, family 4, subfamily C, member 5 (gene/pseudogene)	OR4C5	0,94
transmembrane protein 72	TMEM72	0,94
cytochrome P450, family 4, subfamily A, polypeptide 11	CYP4A11	0,94
microtubule associated serine/threonine kinase 1	MAST1	0,94
Transcript Identified by AceView, Entrez Gene ID(s) 23355	VPS8	0,94
cadherin 3, type 1, P-cadherin (placental)	CDH3	0,94
topoisomerase (DNA) III alpha	TOP3A	0,94
SEC13 homolog, nuclear pore and COPII coat complex component	SEC13	0,94
solute carrier family 9, subfamily A (NHE2, cation proton antiporter 2), member 2	SLC9A2	0,94
tumor protein p63 regulated 1	TPRG1	0,94
calcium channel flower domain containing 1	CACFD1	0,94
tumor necrosis factor receptor superfamily, member 17	TNFRSF17	0,94
Transcript Identified by AceView, Entrez Gene ID(s) 162494	RHBDL3	0,94
Memczak2013 ALT_ACCEPTOR, ALT_DONOR, coding, INTERNAL, intronic best transcript NM_004152	OAZ1	0,94
neurexophilin 1	NXPH1	0,94
trafficking protein particle complex 6B	TRAPPC6B	0,94
ubiquitin specific peptidase 33	USP33	0,94
TRPM8 channel-associated factor 1	TCAF1	0,94
contactin 2 (axonal)	CNTN2	0,94
transforming growth factor beta receptor III	TGFBR3	0,94
atonal bHLH transcription factor 7	ATOH7	0,94
anaphase promoting complex subunit 2	ANAPC2	0,94
cofilin 2 (muscle)	CFL2	0,94
hyaluronoglucosaminidase 1	HYAL1	0,94
ubiquitin protein ligase E3B	UBE3B	0,94
germ cell associated 2 (haspin)	GSG2	0,94
homeobox D9	HOXD9	0,94
eukaryotic translation elongation factor 1 alpha 2	EEF1A2	0,94
bifunctional apoptosis regulator	BFAR	0,94
PR domain containing 13	PRDM13	0,94
histone cluster 1, H3h	HIST1H3H	0,94
ATP binding cassette subfamily A member 7	ABCA7	0,94
centrosomal protein 295kDa	CEP295	0,94
ribosomal protein L36a-like	RPL36AL	0,94
protein phosphatase 2, regulatory subunit B, epsilon isoform	PPP2R5E	0,94

DNA fragmentation factor, 40kDa, beta polypeptide (caspase-activated DNase)	DFFB	0,94
complement component 1, q subcomponent-like 1	C1QL1	0,94
calcium channel flower domain containing 1	CACFD1	0,94
	PRAMEF9	0,94
eukaryotic translation elongation factor 1 alpha 1 pseudogene 5	EEF1A1P5	0,94
CD72 molecule	CD72	0,94
protein phosphatase 4, regulatory subunit 2	PPP4R2	0,94
leucine rich repeat and fibronectin type III domain containing 1	LRFN1	0,94
catenin (cadherin-associated protein), alpha 1	CTNNA1	0,94
LIM homeobox 6	LHX6	0,94
lamin B2; microRNA 7108	LMNB2; MIR7108	0,94
actinin, alpha 4	ACTN4	0,94
family with sequence similarity 129, member C	FAM129C	0,94
ATP binding cassette subfamily B member 10	ABCB10	0,94
Myb/SANT-like DNA-binding domain containing 3	MSANTD3	0,94
eukaryotic translation initiation factor 2, subunit 1 alpha, 35kDa	EIF2S1	0,94
mab-21-like 1 (C. elegans)	MAB21L1	0,94
natural cytotoxicity triggering receptor 2	NCR2	0,94
defensin, beta 126	DEFB126	0,94
zinc finger protein 527	ZNF527	0,94
copine II	CPNE2	0,94
coiled-coil domain containing 15	CCDC15	0,94
complement factor D (adipsin)	CFD	0,94
ribosomal protein L12	RPL12	0,94
YME1-like 1 ATPase	YME1L1	0,94
eukaryotic translation initiation factor 4E family member 1B	EIF4E1B	0,94
ankyrin repeat domain 17	ANKRD17	0,94
small nuclear RNA activating complex polypeptide 5	SNAPC5	0,94
acireductone dioxygenase 1	ADI1	0,94
cysteine-serine-rich nuclear protein 1	CSRNP1	0,94
tumor protein p53 binding protein 1	TP53BP1	0,94
P antigen family, member 4 (prostate associated)	PAGE4	0,94
discs, large homolog 2 (Drosophila)	DLG2	0,94
neuregulin 3	NRG3	0,94
RAB1A, member RAS oncogene family	RAB1A	0,94
nuclear factor, erythroid 2	NFE2	0,94
peptide YY	PYY	0,94
lysocardiolipin acyltransferase 1	LCLAT1	0,94
baculoviral IAP repeat containing 2	BIRC2	0,94
solute carrier family 27 (fatty acid transporter), member 4	SLC27A4	0,94
keratin 74, type II	KRT74	0,94
translation machinery associated 7 homolog	TMA7	0,94
Cbl proto-oncogene-like 1, E3 ubiquitin protein ligase	CBLL1	0,94

phytanoyl-CoA 2-hydroxylase interacting protein-like	PHYHIPL	0,94
component of oligomeric golgi complex 2	COG2	0,94
CTD small phosphatase like 2	CTDSPL2	0,94
SET binding factor 1	SBF1	0,94
coilin	COIL	0,94
fructose-1,6-bisphosphatase 1	FBP1	0,94
sterile alpha motif domain containing 3	SAMD3	0,94
bactericidal/permeability-increasing protein	BPI	0,94
pregnancy specific beta-1-glycoprotein 2	PSG2	0,94
fms-related tyrosine kinase 3	FLT3	0,94
family with sequence similarity 193, member A	FAM193A	0,94
H2A histone family, member J	H2AFJ	0,94
zinc finger protein 883	ZNF883	0,94
transmembrane and ubiquitin-like domain containing 2	TMUB2	0,94
bicaudal D homolog 1 (Drosophila)	BICD1	0,94
ribosomal protein L34	RPL34	0,94
CTP synthase 2	CTPS2	0,94
SPT3 homolog, SAGA and STAGA complex component	SUPT3H	0,94
small integral membrane protein 2	SMIM2	0,94
transforming growth factor beta regulator 4; small nucleolar RNA, H/ACA box 5B	TBRG4; SNORA5B	0,94
dual oxidase 2	DUOX2	0,94
olfactory receptor, family 2, subfamily T, member 11 (gene/pseudogene)	OR2T11	0,94
lectin, galactoside-binding, soluble, 12	LGALS12	0,94
olfactory receptor, family 11, subfamily A, member 1	OR11A1	0,94
chromosome 11 open reading frame 72	C11orf72	0,94
MAGE family member D1	MAGED1	0,94
zinc finger, GATA-like protein 1	ZGLP1	0,94
lysosomal-associated membrane protein 1	LAMP1	0,94
Jeck2013 ANTISENSE, CDS, coding, INTERNAL, intronic, OVCODE, OVEXON best transcript NM_015466	PTPN23	0,94
GAR1 homolog, ribonucleoprotein	GAR1	0,94
sema domain, transmembrane domain (TM), and cytoplasmic domain, (semaphorin) 6D	SEMA6D	0,94
t-complex 10-like 2	TCP10L2	0,94
leucine zipper-EF-hand containing transmembrane protein 1	LETM1	0,94
phosphatidylinositol glycan anchor biosynthesis class N	PIGN	0,94
eukaryotic translation initiation factor 4 gamma, 1	EIF4G1	0,94
potassium channel tetramerization domain containing 11	KCTD11	0,94
polypyrimidine tract binding protein 1; microRNA 4745	PTBP1; MIR4745	0,94
Transcript Identified by AceView, Entrez Gene ID(s) 8395	PIP5K1B	0,94

selenoprotein O; selenoprotein O [Source:EntrezGene;Acc:83642]; Salzman2013 ANNOTATED, CDS, coding, INTERNAL, OVCODE, OVERLAPTX, OVEXON best transcript NM_031454; Salzman2013 ANNOTATED, CDS, coding, OVCODE, OVERLAPTX, OVEXON, UTR3 best transcript NM_031454; Transcript Identified by AceView, Entrez Gene ID(s) 83642, RefSeq ID(s) NM_031454	SELO	0,94
peptidylprolyl isomerase A (cyclophilin A)-like 4F; peptidylprolyl isomerase A (cyclophilin A)-like 4D	PPIAL4F; PPIAL4D	0,94
olfactory receptor, family 8, subfamily J, member 3	OR8J3	0,94
chromosome 15 open reading frame 56	C15orf56	0,94
abhydrolase domain containing 14B	ABHD14B	0,94
IQ motif containing GTPase activating protein 1	IQGAP1	0,94
multiple inositol-polyphosphate phosphatase 1	MINPP1	0,94
G-rich RNA sequence binding factor 1	GRSF1	0,94
prenylcysteine oxidase 1 like	PCYOX1L	0,94
potassium channel, inwardly rectifying subfamily J, member 15	KCNJ15	0,94
WD repeat domain 74	WDR74	0,94
prostate cancer susceptibility candidate 2	PRAC2	0,94
mesoderm specific transcript	MEST	0,94
nerve growth factor receptor (TNFRSF16) associated protein 1	NGFRAP1	0,94
translocase of inner mitochondrial membrane 23 homolog (yeast)	TIMM23	0,94
zinc finger protein 689	ZNF689	0,94
nodal growth differentiation factor	NODAL	0,94
translin	TSN	0,94
EH domain containing 1	EHD1	0,94
zinc finger protein 282	ZNF282	0,94
COP9 signalosome subunit 2	COPS2	0,94
coiled-coil-helix-coiled-coil-helix domain containing 1	CHCHD1	0,94
transforming growth factor alpha	TGFA	0,94
poly(ADP-ribose) polymerase family member 4	PARP4	0,94
CAP, adenylate cyclase-associated protein 1 (yeast)	CAP1	0,94
YEATS domain containing 2	YEATS2	0,94
zinc finger, BED-type containing 5	ZBED5	0,94
chromosome 14 open reading frame 159	C14orf159	0,94
SEC23 interacting protein	SEC23IP	0,94
Memczak2013 ANTISENSE, coding, INTERNAL, intronic best transcript NM_001161841	SULF2	0,94
zinc finger protein 852	ZNF852	0,94
BAI1-associated protein 2-like 2	BAIAP2L2	0,94
heterogeneous nuclear ribonucleoprotein C-like 1	HNRNPCL1	0,94
kallikrein 1	KLK1	0,94
ankyrin repeat domain 49	ANKRD49	0,94
calcium and integrin binding family member 2	CIB2	0,94

matrix metallopeptidase 25	MMP25	0,94
angiopoietin 1	ANGPT1	0,94
REX4 homolog, 3'-5' exonuclease [Source:HGNC Symbol;Acc:HGNC:12820]	REXO4	0,94
short chain dehydrogenase/reductase family 9C, member 7	SDR9C7	0,94
LON peptidase N-terminal domain and ring finger 1	LONRF1	0,94
zinc finger protein 260	ZNF260	0,94
LSM4 homolog, U6 small nuclear RNA and mRNA degradation associated	LSM4	0,94
mannose-binding lectin (protein C) 2, soluble	MBL2	0,94
tetratricopeptide repeat domain 14	TTC14	0,94
bromodomain adjacent to zinc finger domain 1B	BAZ1B	0,94
anaphase promoting complex subunit 10	ANAPC10	0,94
histone cluster 1, H3d; histone cluster 1, H2ad	HIST1H3D; HIST1H2AD	0,94
nuclear cap binding subunit 3	NCBP3	0,94
protocadherin 1	PCDH1	0,94
cullin 3	CUL3	0,94
DEAH (Asp-Glu-Ala-His) box helicase 15	DHX15	0,94
family with sequence similarity 162, member A	FAM162A	0,94
odorant binding protein 2B	OBP2B	0,94
etoposide induced 2.4	EI24	0,94
TYRO3 protein tyrosine kinase	TYRO3	0,94
zinc finger and BTB domain containing 43	ZBTB43	0,94
3-hydroxyanthranilate 3,4-dioxygenase	HAAO	0,94
glucosidase, alpha; acid	GAA	0,94
FGFR1OP N-terminal like	FOPNL	0,94
tripartite motif containing 26	TRIM26	0,94
spermatogenesis associated 32	SPATA32	0,94
fin bud initiation factor homolog (zebrafish)	FIBIN	0,94
chromosome 4 open reading frame 36	C4orf36	0,94
integrin beta 6	ITGB6	0,94
phospholipase C, epsilon 1	PLCE1	0,94
phospholipase D2	PLD2	0,94
olfactory receptor, family 2, subfamily L, member 8 (gene/pseudogene)	OR2L8	0,94
chromosome 2 open reading frame 69	C2orf69	0,94
Transcript Identified by AceView, Entrez Gene ID(s) 57553	MICAL3	0,94
microsomal triglyceride transfer protein	MTTP	0,94
zinc finger protein 445	ZNF445	0,94
VANGL planar cell polarity protein 2	VANGL2	0,94
matrix-remodelling associated 7	MXRA7	0,94
bone morphogenetic protein 5	BMP5	0,94
ubiquitin C-terminal hydrolase L3	UCHL3	0,94
lipoprotein lipase	LPL	0,94

microseminoprotein, prostate associated	MSMP	0,94
keratin 20, type I	KRT20	0,94
calreticulin	CALR	0,94
signal peptide, CUB domain, EGF-like 3	SCUBE3	0,94
RAB3A interacting protein (rabin3)-like 1	RAB3IL1	0,94
histamine receptor H3	HRH3	0,94
solute carrier family 37, member 3	SLC37A3	0,94
apolipoprotein B	APOB	0,94
mitogen-activated protein kinase kinase 7	MAP2K7	0,94
von Willebrand factor A domain containing 1	VWA1	0,94
fibrillarin	FBL	0,94
carbonic anhydrase XIII	CA13	0,94
shisa family member 2	SHISA2	0,94
smu-1 suppressor of mec-8 and unc-52 homolog (C. elegans)	SMU1	0,94
serine/threonine kinase 10	STK10	0,94
NADH dehydrogenase (ubiquinone) flavoprotein 1, 51kDa	NDUFV1	0,94
pantothenate kinase 2	PANK2	0,94
pleiotropic regulator 1	PLRG1	0,94
guanine nucleotide binding protein (G protein), alpha inhibiting activity polypeptide 3	GNAI3	0,94
fidgetin-like 1	FIGNL1	0,94
cAMP responsive element binding protein 3	CREB3	0,94
colorectal neoplasia differentially expressed (non-protein coding)	CRNDE	0,94
MYC associated factor X	MAX	0,94
ER membrane protein complex subunit 8	EMC8	0,94
cathepsin E	CTSE	0,94
polymerase (RNA) II (DNA directed) polypeptide J3	POLR2J3	0,94
uncharacterized LOC101928436; novel transcript; Transcript Identified by AceView	LOC101928436; RP11-153F1.1; RP11-347K2.2; teyfawbu	0,94
uncharacterized LOC401357; novel piRNA host transcript	LOC401357; RP13-580B18.4	0,94
olfactory receptor, family 2, subfamily A, member 14; olfactory receptor, family 2, subfamily A, member 2	OR2A14; OR2A2	0,94
proteasome subunit alpha 3	PSMA3	0,94
isocitrate dehydrogenase 2 (NADP+), mitochondrial	IDH2	0,94
Transcript Identified by AceView, Entrez Gene ID(s) 54715	A2BP1	0,94
activating transcription factor 1	ATF1	0,94
ER lipid raft associated 2	ERLIN2	0,94
NIMA-related kinase 5	NEK5	0,94
zinc finger protein 683	ZNF683	0,94
transforming growth factor beta receptor II	TGFBR2	0,94

integrator complex subunit 7	INTS7	0,94
fragile site, folic acid type, rare, fra(10)(q23.3) or fra(10)(q24.2) candidate 1	FRA10AC1	0,94
SH2 domain containing 3A	SH2D3A	0,94
NmrA-like family domain containing 1	NMRAL1	0,94
olfactory receptor, family 2, subfamily T, member 33	OR2T33	0,94
interleukin 4	IL4	0,94
hydroxysteroid (17-beta) dehydrogenase 3	HSD17B3	0,94
membrane associated ring finger 10	MARCH10	0,94
gamma-glutamyltransferase 2	GGT2	0,94
RAN, member RAS oncogene family	RAN	0,94
Rho GTPase activating protein 18	ARHGAP18	0,94
SRY box 3	SOX3	0,94
histone cluster 1, H2ab	HIST1H2AB	0,94
neuromedin B	NMB	0,94
secretoglobin, family 1C, member 1	SCGB1C1	0,94
transmembrane protein 62	TMEM62	0,94
activating transcription factor 7 interacting protein	ATF7IP	0,94
thioredoxin reductase 1	TXNRD1	0,94
RNA binding motif protein, Y-linked, family 1, member D; RNA binding motif protein, Y-linked, family 1, member B; RNA binding motif protein, Y-linked, family 1, member A1	RBMY1D; RBMY1B; RBMY1A1	0,94
DDB1 and CUL4 associated factor 16	DCAF16	0,94
olfactory receptor, family 52, subfamily I, member 2	OR52I2	0,94
small nuclear ribonucleoprotein, U4/U6.U5 27kDa subunit	SNRNP27	0,94
splicing factor 3b subunit 5	SF3B5	0,94
glutathione S-transferase omega 1	GSTO1	0,94
dual specificity phosphatase 16	DUSP16	0,94
phosphopantothenoylcysteine decarboxylase	PPCDC	0,94
keratin associated protein 19-2	KRTAP19-2	0,94
histidine triad nucleotide binding protein 3	HINT3	0,94
zinc finger with UFM1-specific peptidase domain	ZUFSP	0,94
immunoglobulin (CD79A) binding protein 1	IGBP1	0,94
heterogeneous nuclear ribonucleoprotein D like	HNRNPDL	0,94
fibroblast growth factor 21	FGF21	0,94
DNA-damage-inducible transcript 4-like	DDIT4L	0,94
long intergenic non-protein coding RNA 471	LINC00471	0,94
forkhead box J3	FOXJ3	0,94
histone deacetylase 5	HDAC5	0,94
cell division cycle 40	CDC40	0,94
coiled-coil-helix-coiled-coil-helix domain containing 6	CHCHD6	0,94
Sec61 translocon gamma subunit	SEC61G	0,94
tripartite motif family like 2	TRIML2	0,94
ubiquinol-cytochrome c reductase, complex III subunit X	UQCR10	0,94
Rh family, C glycoprotein	RHCG	0,94

guanine nucleotide binding protein (G protein), beta 5	GNB5	0,94
tRNA selenocysteine 1 associated protein 1	TRNAU1AP	0,94
NLR family, pyrin domain containing 6	NLRP6	0,94
phosphatidylinositol-4,5-bisphosphate 3-kinase, catalytic subunit beta	PIK3CB	0,94
cytochrome c oxidase subunit VIIIA (ubiquitous)	COX8A	0,94
wingless-type MMTV integration site family, member 3	WNT3	0,94
transmembrane protein 205	TMEM205	0,94
bone morphogenetic protein 15	BMP15	0,94
cystatin-like 1	CSTL1	0,94
patatin-like phospholipase domain containing 5	PNPLA5	0,94
junctophilin 2	JPH2	0,94
leucine rich repeat containing 16A	LRRC16A	0,94
GTP-binding protein 10 (putative)	GTPBP10	0,94
ubiquitin conjugating enzyme E2A	UBE2A	0,94
paraneoplastic Ma antigen family member 5	PNMA5	0,94
colony stimulating factor 2 receptor, alpha, low-affinity (granulocyte-macrophage)	CSF2RA	0,94
adhesion G protein-coupled receptor F3	ADGRF3	0,94
MAM domain containing 4	MAMDC4	0,94
phosphomannomutase 1	PMM1	0,94
15 kDa selenoprotein; Salzman2013 ANNOTATED, CDS, coding, INTERNAL, OVCODE, OVEXON best transcript NM_004261; Transcript Identified by AceView, Entrez Gene ID(s) 9403, RefSeq ID(s) NM_203341; Transcript Identified by AceView, Entrez Gene ID(s) 9403, RefSeq ID(s) NM_004261; 15 kDa selenoprotein [Source:EntrezGene;Acc:9403]	sept-15	0,94
olfactory receptor, family 8, subfamily H, member 2	OR8H2	0,94
glycerol-3-phosphate dehydrogenase 1-like	GPD1L	0,94
spalt-like transcription factor 1	SALL1	0,94
interferon gamma receptor 2 (interferon gamma transducer 1)	IFNGR2	0,94
fructose-1,6-bisphosphatase 2	FBP2	0,94
EH domain containing 4	EHD4	0,94
SLCO4A1 antisense RNA 1	SLCO4A1-AS1	0,94
ribosomal protein L7a	RPL7A	0,94
calcium/calmodulin-dependent protein kinase IG	CAMK1G	0,94
wingless-type MMTV integration site family member 2	WNT2	0,94
olfactory receptor, family 51, subfamily M, member 1	OR51M1	0,94
family with sequence similarity 161, member A	FAM161A	0,94
autophagy related 9A	ATG9A	0,94
chromosome 3 open reading frame 36	C3orf36	0,94
olfactory receptor, family 4, subfamily K, member 14	OR4K14	0,94
methyltransferase like 2A	METTL2A	0,94
potassium channel tetramerization domain containing 12	KCTD12	0,94
homeobox D3	HOXD3	0,94

transcriptional adaptor 1	TADA1	0,94
hes family bHLH transcription factor 2	HES2	0,94
baculoviral IAP repeat containing 8	BIRC8	0,94
forkhead box D4	FOXD4	0,94
acetylserotonin O-methyltransferase-like	ASMTL	0,94
acetylserotonin O-methyltransferase-like	ASMTL	0,94
tripartite motif containing 71, E3 ubiquitin protein ligase	TRIM71	0,94
chromosome 6 open reading frame 136	C6orf136	0,94
interferon, omega 1	IFNW1	0,94
nuclear assembly factor 1 ribonucleoprotein	NAF1	0,94
spondin 2, extracellular matrix protein	SPON2	0,94
solute carrier family 4, sodium borate transporter, member 11	SLC4A11	0,94
MIF antisense RNA 1	MIF-AS1	0,94
nudix hydrolase 21	NUDT21	0,94
keratin 12, type I	KRT12	0,94
four and a half LIM domains 3	FHL3	0,94
inturned planar cell polarity protein	INTU	0,94
hydroxysteroid (17-beta) dehydrogenase 7	HSD17B7	0,94
cilia and flagella associated protein 44	CFAP44	0,94
KIAA1161	KIAA1161	0,94
arylformamidase	AFMID	0,94
caspase 1	CASP1	0,94
lysophosphatidylcholine acyltransferase 3	LPCAT3	0,94
surfeit 1	SURF1	0,94
coiled-coil domain containing 120	CCDC120	0,94
membrane magnesium transporter 1	MMGT1	0,94
Transcript Identified by AceView, Entrez Gene ID(s) 669	BPGM	0,94
solute carrier organic anion transporter family, member 1B3	SLCO1B3	0,94
pleckstrin	PLEK	0,94
valyl-tRNA synthetase 2, mitochondrial	VARS2	0,94
Transcript Identified by AceView, Entrez Gene ID(s) 7752	ZNF200	0,94
F-box and leucine-rich repeat protein 3	FBXL3	0,94
RAB11 family interacting protein 2 (class I)	RAB11FIP2	0,94
von Willebrand factor A domain containing 5B2	VWA5B2	0,94
lines homolog 1	LINS1	0,94
ubiquitin specific peptidase 14 (tRNA-guanine transglycosylase)	USP14	0,94
Rho GDP dissociation inhibitor (GDI) beta	ARHGDIB	0,94
ankyrin repeat and SOCS box containing 1	ASB1	0,94
prostaglandin E synthase 2	PTGES2	0,94
tropomyosin 1 (alpha)	TPM1	0,94
THUMP domain containing 2	THUMPD2	0,94
StAR-related lipid transfer domain containing 5	STARD5	0,94
tudor domain containing 7	TDRD7	0,94
golgin A2	GOLGA2	0,94
MARVEL domain containing 3	MARVELD3	0,94

RNA methyltransferase like 1	RNMTL1	0,93
ribosomal protein S12	RPS12	0,93
ubiquitin-conjugating enzyme E2R 2	UBE2R2	0,93
small integral membrane protein 24	SMIM24	0,93
protein phosphatase 1, regulatory subunit 12A	PPP1R12A	0,93
R3H domain containing-like	R3HDML	0,93
DnaJ (Hsp40) homolog, subfamily B, member 11	DNAJB11	0,93
exosome component 8	EXOSC8	0,93
olfactory receptor, family 8, subfamily D, member 1	OR8D1	0,93
sulfiredoxin 1	SRXN1	0,93
A kinase (PRKA) anchor protein 2	AKAP2	0,93
sorting nexin 8; microRNA 6836	SNX8; MIR6836	0,93
intraflagellar transport 122	IFT122	0,93
mitochondrial ribosomal protein L4	MRPL4	0,93
BTAF1 RNA polymerase II, B-TFIID transcription factor-associated, 170kDa	BTAF1	0,93
olfactory receptor, family 5, subfamily D, member 14	OR5D14	0,93
aminomethyltransferase; nicolin 1	AMT; NICN1	0,93
MLX interacting protein	MLXIP	0,93
ankyrin repeat domain 52	ANKRD52	0,93
WD repeat domain 75	WDR75	0,93
proteoglycan 3	PRG3	0,93
DENN/MADD domain containing 4C	DENND4C	0,93
myeloid zinc finger 1	MZF1	0,93
K(lysine) acetyltransferase 5	KAT5	0,93
ring finger protein 19A, RBR E3 ubiquitin protein ligase	RNF19A	0,93
Rho guanine nucleotide exchange factor (GEF) 12	ARHGEF12	0,93
cholinergic receptor, nicotinic beta 4	CHRNB4	0,93
neurobeachin like 2	NBEAL2	0,93
ubiquitin specific peptidase 9, X-linked	USP9X	0,93
mitochondrial ribosomal protein L12	MRPL12	0,93
Transcript Identified by AceView, Entrez Gene ID(s) 23126	POGZ	0,93
dual oxidase maturation factor 2	DUOXA2	0,93
ubiquitin-like 3	UBL3	0,93
G protein-coupled receptor 89B	GPR89B	0,93
solute carrier family 30 (zinc transporter), member 9	SLC30A9	0,93
dipeptidyl-peptidase 4	DPP4	0,93
leucine rich repeat containing 36	LRRC36	0,93
teneurin transmembrane protein 1	TENM1	0,93
zinc finger and SCAN domain containing 5A	ZSCAN5A	0,93
long intergenic non-protein coding RNA 934	LINC00934	0,93
major facilitator superfamily domain containing 2A	MFSD2A	0,93
crystallin beta B1	CRYBB1	0,93
chitinase 3-like 1 (cartilage glycoprotein-39)	CHI3L1	0,93

mucin 3A, cell surface associated	MUC3A	0,93
FERM, ARH/RhoGEF and pleckstrin domain protein 2	FARP2	0,93
cytosolic thiouridylase subunit 1	CTU1	0,93
TNF receptor-associated factor 7, E3 ubiquitin protein ligase	TRAF7	0,93
uroplakin 2	UPK2	0,93
sortilin-related VPS10 domain containing receptor 3	SORCS3	0,93
dipeptidyl-peptidase 3	DPP3	0,93
hes family bHLH transcription factor 4	HES4	0,93
MRS2 magnesium transporter	MRS2	0,93
cyclin N-terminal domain containing 2	CNTD2	0,93
tripartite motif containing 69	TRIM69	0,93
chromosome 20 open reading frame 196	C20orf196	0,93
G protein-coupled receptor kinase 4	GRK4	0,93
	LRIG1	0,93
formin binding protein 1-like	FNBP1L	0,93
eukaryotic translation initiation factor 2, subunit 3 gamma, 52kDa	EIF2S3	0,93
Homo sapiens DiGeorge syndrome critical region gene 6 (DGCR6), mRNA.; protein DGCR6; Homo sapiens DiGeorge syndrome critical region gene 6, mRNA (cDNA clone MGC:54086 IMAGE:5229172), complete cds.; Protein DGCR6 [Source:UniProtKB/Swiss-Prot;Acc:Q14129]	DGCR6; LOC102724770	0,93
RANBP2-like and GRIP domain containing 4	RGPD4	0,93
BMS1 ribosome biogenesis factor	BMS1	0,93
transmembrane protease, serine 7	TMPRSS7	0,93
protein tyrosine phosphatase, receptor type, U	PTPRU	0,93
SPATA31 subfamily C, member 1	SPATA31C1	0,93
origin recognition complex subunit 5	ORC5	0,93
chromosome 14 open reading frame 79	C14orf79	0,93
prolyl 4-hydroxylase, transmembrane (endoplasmic reticulum)	P4HTM	0,93
WW domain binding protein 1-like	WBP1L	0,93
glucosidase, alpha; neutral AB	GANAB	0,93
proteinase 3	PRTN3	0,93
spectrin repeat containing, nuclear envelope family member 4	SYNE4	0,93
eukaryotic translation initiation factor 4E nuclear import factor 1	EIF4ENIF1	0,93
zinc finger protein 785	ZNF785	0,93
ataxin 2	ATXN2	0,93
cysteine rich C-terminal 1	CRCT1	0,93
TraB domain containing	TRABD	0,93
uncharacterized LOC100144595; novel transcript; Transcript Identified by AceView	LOC100144595; AC009227.2; tikure	0,93
gap junction protein gamma 2	GJC2	0,93
ADP-ribosylation factor like GTPase 11	ARL11	0,93

adhesion G protein-coupled receptor L3	ADGRL3	0,93
TBC1 domain family, member 3C	TBC1D3C	0,93
matrilin 2	MATN2	0,93
exosome component 10	EXOSC10	0,93
nuclear factor I/C (CCAAT-binding transcription factor)	NFIC	0,93
chromosome 3 open reading frame 79	C3orf79	0,93
serine/threonine/tyrosine kinase 1	STYK1	0,93
solute carrier family 17 (vesicular glutamate transporter), member 8	SLC17A8	0,93
Transcript Identified by AceView, Entrez Gene ID(s) 51008	ASCC1	0,93
crystallin beta B2	CRYBB2	0,93
protein phosphatase, Mg2+/Mn2+ dependent, 1E	PPM1E	0,93
bromodomain adjacent to zinc finger domain 2B	BAZ2B	0,93
zinc finger protein 451	ZNF451	0,93
cancer susceptibility candidate 4	CASC4	0,93
kelch-like family member 26	KLHL26	0,93
aminoacyl tRNA synthetase complex-interacting multifunctional protein 1	AIMP1	0,93
N-deacetylase/N-sulfotransferase (heparan glucosaminyl) 1	NDST1	0,93
butyrophilin, subfamily 2, member A2	BTN2A2	0,93
G-2 and S-phase expressed 1	GTSE1	0,93
keratin 35, type I	KRT35	0,93
glutathione peroxidase 6	GPX6	0,93
chromosome 9 open reading frame 139	C9orf139	0,93
receptor-interacting serine-threonine kinase 4	RIPK4	0,93
ST6 (alpha-N-acetyl-neuraminyl-2,3-beta-galactosyl-1,3)-N-acetylgalactosaminide alpha-2,6-sialyltransferase 3	ST6GALNAC3	0,93
solute carrier family 37 (glucose-6-phosphate transporter), member 4	SLC37A4	0,93
suppressor of variegation 3-9 homolog 2 (Drosophila)	SUV39H2	0,93
ubiquitin specific peptidase 17-like family member 13	USP17L13	0,93
par-6 family cell polarity regulator alpha	PARD6A	0,93
transmembrane protein 101	TMEM101	0,93
ubiquilin 2	UBQLN2	0,93
opsin 1 (cone pigments), medium-wave-sensitive 2	OPN1MW2	0,93
NGFI-A binding protein 1	NAB1	0,93
ubiquitin-like modifier activating enzyme 1	UBA1	0,93
dehydrogenase/reductase (SDR family) member 1	DHRS1	0,93
t-complex-associated-testis-expressed 3	TCTE3	0,93
G protein-coupled receptor 119	GPR119	0,93
tripartite motif containing 66	TRIM66	0,93
phospholipase A2 inhibitor and LY6/PLAUR domain containing	PINLYP	0,93
Transcript Identified by AceView, Entrez Gene ID(s) 1488	CTBP2	0,93
major histocompatibility complex, class II, DO beta	HLA-DOB	0,93
protein tyrosine phosphatase, non-receptor type 2	PTPN2	0,93

LDL receptor related protein 12	LRP12	0,93
pleckstrin homology-like domain, family A, member 2	PHLDA2	0,93
long intergenic non-protein coding RNA 260	LINC00260	0,93
vacuolar protein sorting 41 homolog (S. cerevisiae)	VPS41	0,93
peptidylprolyl cis/trans isomerase, NIMA-interacting 1	PIN1	0,93
clusterin-like 1 (retinal)	CLUL1	0,93
natural killer cell triggering receptor	NKTR	0,93
zinc finger, matrin-type 1	ZMAT1	0,93
Salzman2013 ANNOTATED, CDS, coding, INTERNAL, OVCODE, OVEXON best transcript NM_006219	PIK3CB	0,93
ankyrin repeat and IBR domain containing 1	ANKIB1	0,93
G protein-coupled receptor 160	GPR160	0,93
Transcript Identified by AceView, Entrez Gene ID(s) 26009	ZZZ3	0,93
disco-interacting protein 2 homolog A	DIP2A	0,93
protein phosphatase 1, regulatory subunit 16A	PPP1R16A	0,93
glypican 5	GPC5	0,93
glutamine and serine rich 1	QSER1	0,93
chromosome 11 open reading frame 96	C11orf96	0,93
mediator complex subunit 12	MED12	0,93
family with sequence similarity 183, member A	FAM183A	0,93
cyclin-dependent kinase 6	CDK6	0,93
protein interacting with cyclin A1	PROCA1	0,93
coiled-coil domain containing 152	CCDC152	0,93
NHS-like 2	NHSL2	0,93
olfactory receptor, family 5, subfamily L, member 2	OR5L2	0,93
acetylserotonin O-methyltransferase; A kinase (PRKA) anchor protein 17A	ASMT; AKAP17A	0,93
Homo sapiens acetylserotonin O-methyltransferase (ASMT), transcript variant 1, mRNA.; Homo sapiens acetylserotonin O-methyltransferase (ASMT), transcript variant 3, mRNA.; acetylserotonin O-methyltransferase [Source:HGNC Symbol;Acc:HGNC:750]; Homo sapiens acetylserotonin O-methyltransferase, mRNA (cDNA clone MGC:2145 IMAGE:3356470), complete cds.; Transcript Identified by AceView, Entrez Gene ID(s) 438; 8227, RefSeq ID(s) NM_001171038	ASMT; SFRS17AandASMT	0,93
molybdenum cofactor synthesis 2	MOCS2	0,93
fermitin family member 2	FERMT2	0,93
phosphatidylinositol 4-kinase, catalytic, alpha pseudogene 1	PI4KAP1	0,93
ring finger protein 167	RNF167	0,93
ubiquitin specific peptidase 26	USP26	0,93
tenascin XB	TNXB	0,93
olfactory receptor, family 2, subfamily F, member 1 (gene/pseudogene)	OR2F1	0,93
CCR4-NOT transcription complex subunit 2	CNOT2	0,93

KDEL (Lys-Asp-Glu-Leu) endoplasmic reticulum protein retention receptor 1	KDELR1	0,93
chromosome 7 open reading frame 60	C7orf60	0,93
deoxyribonuclease I-like 3	DNASE1L3	0,93
RIO kinase 2	RIOK2	0,93
formin 1	FMN1	0,93
solute carrier family 25 (S-adenosylmethionine carrier), member 26	SLC25A26	0,93
snail family zinc finger 3	SNAI3	0,93
F-box protein 34	FBXO34	0,93
tumor necrosis factor receptor superfamily, member 19	TNFRSF19	0,93
protein O-linked mannose N-acetylglucosaminyltransferase 2 (beta 1,4-)	POMGNT2	0,93
telomeric repeat binding factor 2, interacting protein	TERF2IP	0,93
mannose-P-dolichol utilization defect 1	MPDU1	0,93
PAN3 poly(A) specific ribonuclease subunit; RNA, U6 small nuclear 82, pseudogene	PAN3; RNU6-82P	0,93
ring finger protein 114	RNF114	0,93
C3 and PZP-like, alpha-2-macroglobulin domain containing 8	CPAMD8	0,93
formin 1	FMN1	0,93
zinc finger, FYVE domain containing 1	ZFYVE1	0,93
Transcript Identified by AceView, Entrez Gene ID(s) 404636	FAM45A	0,93
G protein-coupled receptor 179	GPR179	0,93
G protein-coupled receptor 156	GPR156	0,93
CDC42 effector protein (Rho GTPase binding) 3	CDC42EP3	0,93
vesicle amine transport 1	VAT1	0,93
acyl-CoA thioesterase 13	ACOT13	0,93
neuregulin 4	NRG4	0,93
striatin, calmodulin binding protein	STRN	0,93
coiled-coil domain containing 82	CCDC82	0,93
steroid-5-alpha-reductase, alpha polypeptide 2 (3-oxo-5 alpha-steroid delta 4-dehydrogenase alpha 2)	SRD5A2	0,93
solute carrier family 25 (mitochondrial carrier), member 16	SLC25A16	0,93
family with sequence similarity 168, member A	FAM168A	0,93
nuclear autoantigenic sperm protein (histone-binding)	NASP	0,93
histidyl-tRNA synthetase	HARS	0,93
synaptic vesicle glycoprotein 2B	SV2B	0,93
solute carrier family 12 (potassium/chloride transporter), member 4	SLC12A4	0,93
sperm associated antigen 11A; sperm associated antigen 11B	SPAG11A; SPAG11B	0,93
epidermal growth factor receptor	EGFR	0,93
cysteine-rich transmembrane module containing 1	CYSTM1	0,93
fibroblast growth factor 11	FGF11	0,93
synaptosome associated protein 47kDa	SNAP47	0,93

N-acetyltransferase 9 (GCN5-related, putative)	NAT9	0,93
DEP domain containing 5	DEPDC5	0,93
PRAME family member 4	PRAMEF4	0,93
G-patch domain and KOW motifs	GPKOW	0,93
four and a half LIM domains 5	FHL5	0,93
mesoderm induction early response 1, family member 3	MIER3	0,93
SWI/SNF related, matrix associated, actin dependent regulator of chromatin, subfamily e, member 1	SMARCE1	0,93
EGF containing fibulin-like extracellular matrix protein 2	EFEMP2	0,93
thioredoxin-related transmembrane protein 4	TMX4	0,93
rhotekin	RTKN	0,93
chemokine (C-X-C motif) ligand 5	CXCL5	0,93
BH3-like motif containing, cell death inducer; mir-100-let-7a-2 cluster host gene	BLID; MIR100HG	0,93
SUMO1/sentrin/SMT3 specific peptidase 2	SENP2	0,93
minichromosome maintenance 10 replication initiation factor	MCM10	0,93
olfactory receptor, family 10, subfamily G, member 3	OR10G3	0,93
pyruvate dehydrogenase (lipoamide) beta	PDHB	0,93
histone deacetylase 3	HDAC3	0,93
neuroblastoma RAS viral (v-ras) oncogene homolog	NRAS	0,93
synemin, intermediate filament protein	SYNM	0,93
damage-specific DNA binding protein 1	DDB1	0,93
period circadian clock 3	PER3	0,93
mitochondrial ribosomal protein L3	MRPL3	0,93
SIN3 transcription regulator family member A	SIN3A	0,93
shugoshin-like 1 (S. pombe)	SGOL1	0,93
zinc finger protein 366	ZNF366	0,93
RAB18, member RAS oncogene family	RAB18	0,93
syntrophin, beta 2 (dystrophin-associated protein A1, 59kDa, basic component 2)	SNTB2	0,93
mitochondrial ribosomal protein S10	MRPS10	0,93
von Willebrand factor A domain containing 9	VWA9	0,93
DnaJ (Hsp40) homolog, subfamily B, member 11	DNAJB11	0,93
RAD23 homolog A, nucleotide excision repair protein	RAD23A	0,93
galactosidase beta 1 like 3	GLB1L3	0,93
carboxypeptidase, vitellogenic-like	CPVL	0,93
Memczak2013 ANTISENSE, coding, INTERNAL, UTR3 best transcript NM_032434	ZNF512	0,93
syntaxin binding protein 4	STXBP4	0,93
neuroepithelial cell transforming 1	NET1	0,93
X-linked Kx blood group related 8	XKR8	0,93
sodium channel, voltage gated, type V alpha subunit	SCN5A	0,93
aquarius intron-binding spliceosomal factor	AQR	0,93
G protein-coupled receptor 4	GPR4	0,93
olfactory receptor, family 6, subfamily C, member 6	OR6C6	0,93

testis expressed 40	TEX40	0,93
MAP3K12 binding inhibitory protein 1	MBIP	0,93
zinc finger protein-like 1	ZFPL1	0,93
tumor necrosis factor (ligand) superfamily, member 14	TNFSF14	0,93
phospholipase C, eta 1	PLCH1	0,93
WAS/WASL interacting protein family, member 1	WIPF1	0,93
transmembrane protein 64	TMEM64	0,93
dishevelled segment polarity protein 2	DVL2	0,93
ZFP36 ring finger protein-like 1	ZFP36L1	0,93
hexokinase 1	HK1	0,93
ArfGAP with coiled-coil, ankyrin repeat and PH domains 3	ACAP3	0,93
potassium channel, inwardly rectifying subfamily J, member 3	KCNJ3	0,93
inositol polyphosphate-5-phosphatase D	INPP5D	0,93
yippee like 5	YPEL5	0,93
chromosome 2 open reading frame 83	C2orf83	0,93
cadherin 17, LI cadherin (liver-intestine)	CDH17	0,93
zinc finger, AN1-type domain 2B	ZFAND2B	0,93
uncharacterized LOC100506142; Memczak2013 ANTISENSE, coding, INTERNAL, intronic best transcript NM_012249; novel transcript	LOC100506142; RP11-417F21.1; RHOQ	0,93
otolin 1	OTOL1	0,93
RIC8 guanine nucleotide exchange factor A; microRNA 6743	RIC8A; MIR6743	0,93
T-cell leukemia/lymphoma 1A	TCL1A	0,93
palmitoyl-protein thioesterase 1	PPT1	0,93
ankyrin repeat domain 40	ANKRD40	0,93
terminal uridylyl transferase 1, U6 snRNA-specific	TUT1	0,93
sodium channel, non voltage gated 1 beta subunit	SCNN1B	0,93
olfactory receptor, family 2, subfamily A, member 1	OR2A1	0,93
oxysterol binding protein-like 2	OSBPL2	0,93
WW domain binding protein 4	WBP4	0,93
zinc finger protein 318	ZNF318	0,93
fatty acid binding protein 6, ileal	FABP6	0,93
fms-related tyrosine kinase 3 ligand	FLT3LG	0,93
golgin A7 family, member B	GOLGA7B	0,93
pleckstrin homology domain containing, family A (phosphoinositide binding specific) member 1	PLEKHA1	0,93
amine oxidase, copper containing 2 (retina-specific)	AOC2	0,93
autophagy related 12	ATG12	0,93
LDL receptor related protein 10	LRP10	0,93
neural EGFL like 1	NELL1	0,93
ribonucleic acid export 1	RAE1	0,93
sphingomyelin phosphodiesterase, acid-like 3B	SMPDL3B	0,93
kinesin family member 14	KIF14	0,93

cyclin-dependent kinase inhibitor 2B (p15, inhibits CDK4)	CDKN2B	0,93
superkiller viralicidic activity 2-like 2 (S. cerevisiae)	SKIV2L2	0,93
Zhang2013 ALT_ACCEPTOR, ALT_DONOR, coding, INTERNAL, intronic, OVERLAPTX best transcript NM_017623	CNNM3	0,93
leukemia inhibitory factor receptor alpha	LIFR	0,93
collagen, type VI, alpha 2	COL6A2	0,93
transmembrane protein 192	TMEM192	0,93
YjeF N-terminal domain containing 3	YJEFN3	0,93
olfactory receptor, family 11, subfamily H, member 12	OR11H12	0,93
proline rich Gla (G-carboxyglutamic acid) 1	PRRG1	0,93
GATA zinc finger domain containing 2B	GATAD2B	0,93
leukocyte immunoglobulin-like receptor, subfamily B (with TM and ITIM domains), member 5	LILRB5	0,93
v-crk avian sarcoma virus CT10 oncogene homolog-like	CRKL	0,93
sphingosine kinase 1	SPHK1	0,93
Transcript Identified by AceView, Entrez Gene ID(s) 7752	ZNF200	0,93
oculocutaneous albinism II	OCA2	0,93
chondroitin sulfate proteoglycan 5 (neuroglycan C)	CSPG5	0,93
Janus kinase and microtubule interacting protein 3	JAKMIP3	0,93
transmembrane protein 254	TMEM254	0,93
GATS protein-like 2	GATSL2	0,93
tumor necrosis factor receptor superfamily, member 13B	TNFRSF13B	0,93
transcription factor 21	TCF21	0,93
proteasome subunit beta 9	PSMB9	0,93
FXYD domain containing ion transport regulator 4	FXYD4	0,93
Transcript Identified by AceView, Entrez Gene ID(s) 23081	KDM4C	0,93
programmed cell death 7	PDCD7	0,93
heterogeneous nuclear ribonucleoprotein C-like 2	HNRNPCL2	0,93
calcyphosine 2	CAPS2	0,93
SAC1 suppressor of actin mutations 1-like (yeast)	SACM1L	0,93
iroquois homeobox 4	IRX4	0,93
MAGE family member A9B; MAGE family member A9	MAGEA9B; MAGEA9	0,93
cysteine-rich protein 1 (intestinal)	CRIP1	0,93
protein Z, vitamin K-dependent plasma glycoprotein	PROZ	0,93
mitochondrial ribosomal protein L49	MRPL49	0,93
pantothenate kinase 1	PANK1	0,93
myoneurin	MYNN	0,93
sialic acid binding Ig-like lectin 12 (gene/pseudogene)	SIGLEC12	0,93
glutamine-rich 1	QRICH1	0,93
oocyte expressed protein	OOEP	0,93
GTPase, IMAP family member 7	GIMAP7	0,93
RAB6C-like; Synthetic construct Homo sapiens clone IMAGE:100016523, MGC:184337 RAB6C-like (LOC150786) mRNA, encodes complete protein.	WTH3DI; LOC150786	0,93

eukaryotic translation elongation factor 1 alpha 1	EEF1A1	0,93
poly(rC) binding protein 2; PCBP2 overlapping transcript 1	PCBP2; PCBP2-OT1	0,93
neuro-oncological ventral antigen 1	NOVA1	0,93
coatomer protein complex subunit zeta 1	COPZ1	0,93
kelch-like family member 9	KLHL9	0,93
RNA binding motif protein 38	RBM38	0,93
ADP-ribosylation factor like GTPase 1	ARL1	0,93
keratin associated protein 19-8	KRTAP19-8	0,93
dihydrofolate reductase; dihydrofolate reductase pseudogene 1	DHFR; DHFRP1	0,93
solute carrier family 22 (organic anion/urate transporter), member 11	SLC22A11	0,93
poly(A) binding protein, cytoplasmic 4 (inducible form)	PABPC4	0,93
UBX domain protein 10	UBXN10	0,93
MRVI1 antisense RNA 1	MRVI1-AS1	0,93
guanine nucleotide binding protein (G protein), gamma 7	GNG7	0,93
integrator complex subunit 2	INTS2	0,93
ring finger and CCCH-type domains 1	RC3H1	0,93
tripartite motif containing 63, E3 ubiquitin protein ligase	TRIM63	0,93
thromboxane A synthase 1 (platelet)	TBXAS1	0,93
ADP-ribosylation factor like GTPase 10	ARL10	0,93
transgelin	TAGLN	0,93
chromosome 6 open reading frame 163	C6orf163	0,93
DnaJ (Hsp40) homolog, subfamily C, member 22	DNAJC22	0,93
Memczak2013 ALT_ACCEPTOR, ALT_DONOR, coding, INTERNAL, intronic best transcript NM_015986	CRLF3	0,93
retinoblastoma-like 2	RBL2	0,93
adaptor-related protein complex 2, mu 1 subunit	AP2M1	0,93
LDL receptor related protein associated protein 1	LRPAP1	0,93
centrosomal protein 250kDa	CEP250	0,93
pericentrin	PCNT	0,93
MORN repeat containing 3	MORN3	0,93
CBP80/20-dependent translation initiation factor; microRNA 4743	CTIF; MIR4743	0,93
SPT16 homolog, facilitates chromatin remodeling subunit	SUPT16H	0,93
RPL36A-HNRNPH2 readthrough	RPL36A-HNRNPH2	0,93
catsper channel auxiliary subunit delta	CATSPERD	0,93
ankyrin repeat domain 45	ANKRD45	0,93
SAYSVFN motif domain containing 1	SAYSD1	0,93
proteasome (prosome, macropain) assembly chaperone 4	PSMG4	0,93
msh homeobox 2	MSX2	0,93
cyclin Pas1/PHO80 domain containing 1	CNPPD1	0,93
erythrocyte membrane protein band 4.2	EPB42	0,93
olfactory receptor, family 52, subfamily E, member 8	OR52E8	0,93

arylsulfatase E (chondrodysplasia punctata 1)	ARSE	0,93
growth arrest-specific 2 like 1	GAS2L1	0,93
family with sequence similarity 228, member A	FAM228A	0,93
cytochrome P450, family 26, subfamily A, polypeptide 1	CYP26A1	0,93
peroxiredoxin 4	PRDX4	0,93
ribosomal protein S6 kinase, 70kDa, polypeptide 1	RPS6KB1	0,93
dihydrolipoamide dehydrogenase	DLD	0,93
leucine rich repeat and Ig domain containing 3	LINGO3	0,93
pleckstrin homology domain containing, family M (with RUN domain) member 1; microRNA 4315-1	PLEKHM1; MIR4315-1	0,93
lecithin retinol acyltransferase (phosphatidylcholine--retinol O-acyltransferase)	LRAT	0,93
G protein-coupled receptor 3	GPR3	0,93
CD8a molecule	CD8A	0,93
protein kinase C, beta	PRKCB	0,93
kelch-like family member 20	KLHL20	0,93
tuberous sclerosis 1	TSC1	0,93
MT-RNR2-like 8; microRNA 4485	MTRNR2L8; MIR4485	0,93
transportin 3	TNPO3	0,93
copine V	CPNE5	0,93
WD repeat domain 34	WDR34	0,93
solute carrier family 4 (sodium bicarbonate cotransporter), member 4	SLC4A4	0,93
transient receptor potential cation channel, subfamily C, member 4 associated protein	TRPC4AP	0,93
SNAP-associated protein	SNAPIN	0,93
alpha 1,3-galactosyltransferase 2	A3GALT2	0,93
Memczak2013 ANTISENSE, coding, INTERNAL, intronic best transcript NM_001198696	AIFM2	0,93
growth arrest-specific 7	GAS7	0,93
DCN1, defective in cullin neddylation 1, domain containing 2	DCUN1D2	0,93
THAP domain containing 9	THAP9	0,93
tRNA methyltransferase 10B	TRMT10B	0,93
KN motif and ankyrin repeat domains 1	KANK1	0,93
pleckstrin homology domain containing, family G (with RhoGef domain) member 3	PLEKHG3	0,93
G protein-coupled receptor 78	GPR78	0,93
cadherin 2, type 1, N-cadherin (neuronal)	CDH2	0,93
angiopoietin like 1	ANGPTL1	0,93
ubiquitin specific peptidase 47	USP47	0,93
cytochrome c oxidase assembly homolog 15 (yeast)	COX15	0,93
cytokine receptor-like factor 1	CRLF1	0,93
P450 (cytochrome) oxidoreductase; microRNA 4651	POR; MIR4651	0,93
adhesion G protein-coupled receptor V1	ADGRV1	0,93

early growth response 4	EGR4	0,93
kynurenine 3-monooxygenase (kynurenine 3-hydroxylase)	KMO	0,93
chromosome 14 open reading frame 37	C14orf37	0,93
tumor necrosis factor receptor superfamily, member 8	TNFRSF8	0,93
proline-rich nuclear receptor coactivator 2	PNRC2	0,93
chromosome 18 open reading frame 32	C18orf32	0,93
G protein-coupled receptor 6	GPR6	0,93
ubiquitin specific peptidase 19	USP19	0,93
nestin	NES	0,93
dual adaptor of phosphotyrosine and 3-phosphoinositides	DAPP1	0,93
ATP synthase, H+ transporting, mitochondrial F1 complex, alpha subunit 1, cardiac muscle	ATP5A1	0,93
glutathione synthetase	GSS	0,93
cAMP responsive element binding protein 3-like 2	CREB3L2	0,93
golgin A8 family, member T	GOLGA8T	0,93
Transcript Identified by AceView, Entrez Gene ID(s) 10587	TXNRD2	0,93
carboxypeptidase A3 (mast cell)	CPA3	0,93
dihydropyrimidinase-like 4	DPYSL4	0,93
gon-4-like (C. elegans)	GON4L	0,93
zinc finger protein 284	ZNF284	0,93
beta-transducin repeat containing E3 ubiquitin protein ligase	BTRC	0,93
thioredoxin domain containing 2 (spermatozoa)	TXNDC2	0,93
G protein-coupled receptor 87	GPR87	0,93
collagen beta(1-O)galactosyltransferase 1	COLGALT1	0,93
EPH receptor A8	EPHA8	0,93
par-6 family cell polarity regulator beta	PARD6B	0,93
RIC3 acetylcholine receptor chaperone	RIC3	0,93
poly(ADP-ribose) polymerase family member 8	PARP8	0,93
chromosome 16 open reading frame 78	C16orf78	0,93
methyltransferase like 4	METTL4	0,93
peptidylprolyl isomerase A (cyclophilin A) pseudogene 30	PPIAP30	0,93
ribonuclease, RNase A family, 12 (non-active); ribonuclease, RNase A family, 11 (non-active)	RNASE12; RNASE11	0,93
bone morphogenetic protein 4	BMP4	0,93
potassium channel, two pore domain subfamily K, member 4	KCNK4	0,93
REC8 meiotic recombination protein	REC8	0,93
family with sequence similarity 219, member B	FAM219B	0,93
translocase of outer mitochondrial membrane 6 homolog (yeast); prickle homolog 4	TOMM6; PRICKLE4	0,93
transmembrane protein 258; microRNA 611	TMEM258; MIR611	0,93
defensin, beta 115	DEFB115	0,93
coiled-coil domain containing 105	CCDC105	0,93
NMD3 ribosome export adaptor	NMD3	0,93
forkhead box A2	FOXA2	0,93

NMDA receptor synaptonuclear signaling and neuronal migration factor; microRNA 7114	NSMF; MIR7114	0,93
Transcript Identified by AceView, Entrez Gene ID(s) 221937	FOXK1	0,93
round spermatid basic protein 1-like	RSBN1L	0,93
TBC1 domain family, member 25	TBC1D25	0,93
HSPA (heat shock 70kDa) binding protein, cytoplasmic cochaperone 1	HSPBP1	0,93
histone cluster 1, H1a	HIST1H1A	0,93
sarcalumenin	SRL	0,93
synovial sarcoma, X breakpoint 6 (pseudogene)	SSX6	0,93
surfeit 4	SURF4	0,93
BSD domain containing 1	BSDC1	0,93
adhesion G protein-coupled receptor L2	ADGRL2	0,93
TEN1 CST complex subunit; cyclin-dependent kinase 3; TEN1-CDK3 readthrough (NMD candidate)	TEN1; CDK3; TEN1-CDK3	0,93
guanine nucleotide binding protein (G protein) alpha 12	GNA12	0,93
YEATS domain containing 4	YEATS4	0,93
protein phosphatase 4, regulatory subunit 3A	PPP4R3A	0,93
nuclear envelope integral membrane protein 1	NEMP1	0,93
dendrocyte expressed seven transmembrane protein	DCSTAMP	0,93
vasohibin 1	VASH1	0,93
tetratricopeptide repeat domain 32	TTC32	0,93
thioredoxin reductase 3 neighbor; thioredoxin reductase 3	TXNRD3NB; TXNRD3	0,93
chromosome 8 open reading frame 22	C8orf22	0,93
protein phosphatase, Mg2+/Mn2+ dependent, 1N (putative)	PPM1N	0,93
N(alpha)-acetyltransferase 10, NatA catalytic subunit	NAA10	0,93
pyruvate kinase, liver and RBC	PKLR	0,93
lactoperoxidase	LPO	0,93
HCLS1 binding protein 3	HS1BP3	0,93
lipase, hormone-sensitive	LIPE	0,93
T cell activation inhibitor, mitochondrial	TCAIM	0,93
epididymal sperm binding protein 1	ELSPBP1	0,93
retinoic acid receptor responder (tazarotene induced) 1	RARRES1	0,93
transforming growth factor, beta receptor 1	TGFBR1	0,93
secernin 2	SCRN2	0,93
EYA transcriptional coactivator and phosphatase 1	EYA1	0,93
neutral cholesterol ester hydrolase 1	NCEH1	0,93
interleukin 18 binding protein	IL18BP	0,93
scavenger receptor class A, member 3	SCARA3	0,93
acyl-CoA synthetase bubblegum family member 2	ACSBG2	0,93
Salzman2013 ALT_ACCEPTOR, ALT_DONOR, coding, INTERNAL, intronic best transcript NM_080476	PIGU	0,93
catenin (cadherin-associated protein), alpha-like 1	CTNNAL1	0,93
midkine (neurite growth-promoting factor 2)	MDK	0,93

keratin associated protein 2-3	KRTAP2-3	0,93
translocase of inner mitochondrial membrane 21 homolog (yeast)	TIMM21	0,93
elastin microfibril interfacer 3	EMILIN3	0,93
amyloid P component, serum	APCS	0,93
proteasome 26S subunit, ATPase 3	PSMC3	0,93
ADAM metallopeptidase with thrombospondin type 1 motif 13	ADAMTS13	0,93
C1q and tumor necrosis factor related protein 7	C1QTNF7	0,93
keratin associated protein 1-1	KRTAP1-1	0,93
histone cluster 1, H2bj	HIST1H2BJ	0,93
Kv channel interacting protein 3, calsenilin	KCNIP3	0,93
trace amine associated receptor 8	TAAR8	0,93
chloride channel, voltage-sensitive 6	CLCN6	0,93
LIM and cysteine-rich domains 1; long intergenic non-protein coding RNA 312	LMCD1; LINC00312	0,93
scratch family zinc finger 2	SCRT2	0,93
NADH dehydrogenase (ubiquinone) 1 alpha subcomplex, 5	NDUFA5	0,93
coiled-coil domain containing 174	CCDC174	0,93
cell division cycle 25A	CDC25A	0,93
MAM domain containing 2	MAMDC2	0,93
nanos homolog 1 (Drosophila)	NANOS1	0,93
sterile alpha motif domain containing 14	SAMD14	0,93
primase, DNA, polypeptide 2 (58kDa)	PRIM2	0,93
prostaglandin F2 receptor inhibitor	PTGFRN	0,93
kelch-like family member 18	KLHL18	0,93
fibrillarin	FBL	0,93
receptor accessory protein 2	REEP2	0,93
neuroblastoma breakpoint family, member 14	NBPF14	0,93
carbonyl reductase 3	CBR3	0,93
olfactory receptor, family 5, subfamily H, member 6 (gene/pseudogene)	OR5H6	0,93
golgi phosphoprotein 3 (coat-protein)	GOLPH3	0,93
PRAME family member 16; PRAME family member 17	PRAMEF16; PRAMEF17	0,93
mannose phosphate isomerase	MPI	0,93
Src-like-adaptor 2	SLA2	0,93
uncharacterized LOC100130357; novel transcript, antisense to PHACTR1	LOC100130357; RP1-257A7.4	0,93
6-phosphogluconolactonase	PGLS	0,93
EF-hand calcium binding domain 9	EFCAB9	0,93
meiotic kinetochore factor	MEIKIN	0,93
A kinase (PRKA) anchor protein 4	AKAP4	0,93
unc-80 homolog (C. elegans)	UNC80	0,93
regulator of chromosome condensation 2	RCC2	0,93
spalt-like transcription factor 3	SALL3	0,93

protein phosphatase 1, regulatory subunit 3C	PPP1R3C	0,93
emerin	EMD	0,93
oxysterol binding protein-like 10	OSBPL10	0,93
isthmin 2	ISM2	0,93
WD repeat domain 60	WDR60	0,93
nucleolar protein 10	NOL10	0,93
zinc finger and BTB domain containing 8A	ZBTB8A	0,93
BCL2-like 12 (proline rich)	BCL2L12	0,93
ADP-ribosylation factor like GTPase 2	ARL2	0,93
leucine rich repeat (in FLII) interacting protein 1	LRRFIP1	0,93
thioredoxin-related transmembrane protein 3	TMX3	0,93
DNA cross-link repair 1C	DCLRE1C	0,93
interleukin 4 receptor	IL4R	0,93
glucosaminyl (N-acetyl) transferase family member 7	GCNT7	0,93
thymine DNA glycosylase	TDG	0,93
pituitary tumor-transforming 1	PTTG1	0,93
zinc finger protein 280B	ZNF280B	0,93
RasGEF domain family member 1B	RASGEF1B	0,93
endothelial cell adhesion molecule	ESAM	0,93
RAN binding protein 9	RANBP9	0,93
cilia and flagella associated protein 206	CFAP206	0,93
zinc finger protein 529	ZNF529	0,93
ribosomal protein L39; small nucleolar RNA, H/ACA box 69	RPL39; SNORA69	0,93
heparan sulfate (glucosamine) 3-O-sulfotransferase 3A1	HS3ST3A1	0,93
insulin like growth factor binding protein 2	IGFBP2	0,93
sarcoma antigen 1	SAGE1	0,93
Ras association (RalGDS/AF-6) domain family (N-terminal) member 9	RASSF9	0,93
solute carrier family 26 (anion exchanger), member 7	SLC26A7	0,93
DEAD (Asp-Glu-Ala-Asp) box polypeptide 39B; small nucleolar RNA, C/D box 84	DDX39B; SNORD84	0,93
solute carrier family 39 (zinc transporter), member 6	SLC39A6	0,93
tachykinin receptor 3	TACR3	0,93
potassium channel tetramerization domain containing 6	KCTD6	0,93
polymeric immunoglobulin receptor	PIGR	0,93
LYR motif containing 9	LYRM9	0,93
DnaJ (Hsp40) homolog, subfamily C, member 17	DNAJC17	0,93
zinc finger protein 83	ZNF83	0,93
solute carrier family 37 (glucose-6-phosphate transporter), member 1	SLC37A1	0,93
alpha-1,4-N-acetylglucosaminyltransferase	A4GNT	0,93
CUB and Sushi multiple domains 3	CSMD3	0,93
translational activator of mitochondrially encoded cytochrome c oxidase I	TACO1	0,93

G protein-coupled receptor 141	GPR141	0,93
solute carrier family 17 (acidic sugar transporter), member 5	SLC17A5	0,93
olfactory receptor, family 2, subfamily J, member 1 (gene/pseudogene)	OR2J1	0,93
cytochrome b561	CYB561	0,93
transmembrane and coiled-coil domains 1	TMCO1	0,93
ATPase type 13A1	ATP13A1	0,93
prodynorphin	PDYN	0,93
hypoxia inducible factor 3, alpha subunit	HIF3A	0,93
phosphoribosyl pyrophosphate synthetase 1-like 1	PRPS1L1	0,93
AT rich interactive domain 4A (RBP1-like)	ARID4A	0,93
immunoglobulin superfamily, member 10	IGSF10	0,93
general transcription factor IIIC subunit 4	GTF3C4	0,93
NK2 homeobox 5	NKX2-5	0,93
endothelial PAS domain protein 1	EPAS1	0,93
fibrosin-like 1	FBRSL1	0,93
aldo-keto reductase family 1, member C3	AKR1C3	0,93
non-SMC condensin I complex subunit D2	NCAPD2	0,93
MT-RNR2-like 10	MTRNR2L10	0,93
forkhead box N3	FOXN3	0,93
sex comb on midleg-like 1 (Drosophila)	SCML1	0,93
SRY box 5	SOX5	0,93
ATPase, aminophospholipid transporter, class I, type 8B, member 3	ATP8B3	0,93
Kazal-type serine peptidase inhibitor domain 1	KAZALD1	0,93
protein kinase N3	PKN3	0,93
protein phosphatase 1, regulatory subunit 16B	PPP1R16B	0,93
MAS-related GPR, member X2	MRGPRX2	0,93
heterogeneous nuclear ribonucleoprotein U (scaffold attachment factor A)	HNRNPU	0,93
filamin B, beta	FLNB	0,93
kyphoscoliosis peptidase	KY	0,93
zinc finger protein 285	ZNF285	0,93
ADAM metallopeptidase with thrombospondin type 1 motif 13	ADAMTS13	0,93
secretoglobin, family 3A, member 1	SCGB3A1	0,93
zinc finger protein 827	ZNF827	0,93
family with sequence similarity 169, member A	FAM169A	0,93
interferon regulatory factor 5	IRF5	0,93
8-oxoguanine DNA glycosylase	OGG1	0,93
zinc finger protein 645	ZNF645	0,93
RAS protein activator like 2	RASAL2	0,93
Transcript Identified by AceView, Entrez Gene ID(s) 79041	TMEM38A	0,93
myotubularin 1	MTM1	0,93
WEE1 homolog 2 (S. pombe)	WEE2	0,93
exocyst complex component 1	EXOC1	0,93

caspase 8 associated protein 2	CASP8AP2	0,93
KIAA0895-like	KIAA0895L	0,93
disrupted in schizophrenia 1	DISC1	0,93
transcription elongation factor B polypeptide 3C (elongin A3)	TCEB3C	0,93
galactosidase beta 1 like	GLB1L	0,93
RAD51 paralog D	RAD51D	0,93
maltase-glucoamylase	MGAM	0,93
quiescin Q6 sulfhydryl oxidase 2	QSOX2	0,93
ferric-chelate reductase 1-like	FRRS1L	0,93
phospholipase A2, group III	PLA2G3	0,93
family with sequence similarity 132, member B	FAM132B	0,93
mitotic spindle positioning	MISP	0,93
phosphodiesterase 12	PDE12	0,93
peptidoglycan recognition protein 2	PGLYRP2	0,93
BarH-like homeobox 1	BARHL1	0,93
kelch-like family member 41	KLHL41	0,93
KDEL (Lys-Asp-Glu-Leu) containing 2	KDELC2	0,93
heat shock protein 90kDa alpha (cytosolic), class B member 1	HSP90AB1	0,93
transcription factor AP-2 alpha (activating enhancer binding protein 2 alpha)	TFAP2A	0,93
DND microRNA-mediated repression inhibitor 1	DND1	0,93
GRB2 associated regulator of MAPK1 2	GAREM2	0,93
MT-RNR2-like 5	MTRNR2L5	0,93
dynein, axonemal, intermediate chain 1	DNAI1	0,93
WD repeat domain, phosphoinositide interacting 2	WIPI2	0,92
olfactory receptor, family 51, subfamily A, member 2	OR51A2	0,92
secretoglobin, family 1C, member 2	SCGB1C2	0,92
sidekick cell adhesion molecule 2	SDK2	0,92
reversion-inducing-cysteine-rich protein with kazal motifs	RECK	0,92
laccase (multicopper oxidoreductase) domain containing 1	LACC1	0,92
fucosyltransferase 1 (galactoside 2-alpha-L-fucosyltransferase, H blood group)	FUT1	0,92
interleukin 17 receptor E-like	IL17REL	0,92
polycomb group ring finger 6	PCGF6	0,92
olfactory receptor, family 5, subfamily T, member 3	OR5T3	0,92
keratin associated protein 10-6	KRTAP10-6	0,92
transmembrane protein with EGF-like and two follistatin-like domains 2	TMEFF2	0,92
WD repeat domain 18	WDR18	0,92
BCL2 associated athanogene 6	BAG6	0,92
spinster homolog 1 (Drosophila)	SPNS1	0,92
ceramide kinase	CERK	0,92
keratin associated protein 8-1	KRTAP8-1	0,92
G2/M-phase specific E3 ubiquitin protein ligase	G2E3	0,92
ovo-like zinc finger 2	OVOL2	0,92

TBC1 domain family, member 24	TBC1D24	0,92
ubiquitin protein ligase E3 component n-recognin 2	UBR2	0,92
chromosome 17 open reading frame 96	C17orf96	0,92
PYM homolog 1, exon junction complex associated factor	PYM1	0,92
epithelial membrane protein 2	EMP2	0,92
thioredoxin domain containing 5 (endoplasmic reticulum)	TXNDC5	0,92
HUS1 checkpoint clamp component B	HUS1B	0,92
olfactory receptor, family 2, subfamily A, member 7; Rho guanine nucleotide exchange factor 34, pseudogene	OR2A7; ARHGEF34P	0,92
tumor necrosis factor receptor superfamily, member 14	TNFRSF14	0,92
SH3-domain GRB2-like 3	SH3GL3	0,92
mitochondrial translational initiation factor 3	MTIF3	0,92
MOB kinase activator 1B	MOB1B	0,92
keratin associated protein 10-9	KRTAP10-9	0,92
pantothenate kinase 2	PANK2	0,92
serine/threonine kinase 4	STK4	0,92
EF-hand calcium binding domain 14	EFCAB14	0,92
low density lipoprotein receptor class A domain containing 2	LDLRAD2	0,92
keratin 18, type I	KRT18	0,92
syntaxin 17	STX17	0,92
polypeptide N-acetylgalactosaminyltransferase 3	GALNT3	0,92
long intergenic non-protein coding RNA 961	LINC00961	0,92
signal recognition particle 72kDa	SRP72	0,92
phosphorylated adaptor for RNA export	PHAX	0,92
uncoupling protein 1 (mitochondrial, proton carrier)	UCP1	0,92
transmembrane protein 215	TMEM215	0,92
Ly6/neurotoxin 1	LYNX1	0,92
tight junction protein 1	TJP1	0,92
angel homolog 1 (Drosophila)	ANGEL1	0,92
claudin domain containing 2	CLDND2	0,92
espin	ESPN	0,92
family with sequence similarity 45, member A	FAM45A	0,92
late cornified envelope 3B	LCE3B	0,92
UPF3 regulator of nonsense transcripts homolog B (yeast)	UPF3B	0,92
oral-facial-digital syndrome 1	OFD1	0,92
MAX interactor 1, dimerization protein	MXI1	0,92
zinc finger CCHC-type and RNA binding motif 1; microRNA 7851	ZCRB1; MIR7851	0,92
retinoic acid early transcript 1G	RAET1G	0,92
p53-induced death domain protein 1	PIDD1	0,92
PMS1 homolog 2, mismatch repair system component	PMS2	0,92
tyrosyl-DNA phosphodiesterase 2	TDP2	0,92
ring finger protein 123	RNF123	0,92
LUC7-like 3 pre-mRNA splicing factor	LUC7L3	0,92

zinc finger protein 670; zinc finger protein 695; ZNF670-ZNF695 readthrough (NMD candidate)	ZNF670; ZNF695; ZNF670-ZNF695	0,92
cathepsin H	CTSH	0,92
lysine (K)-specific demethylase 4C	KDM4C	0,92
HBS1-like translational GTPase	HBS1L	0,92
katanin p60 subunit A-like 1	KATNAL1	0,92
arginyltransferase 1	ATE1	0,92
Bet1 golgi vesicular membrane trafficking protein	BET1	0,92
Memczak2013 ANTISENSE, coding, INTERNAL, intronic best transcript NM_014117	C16orf72	0,92
tRNA methyltransferase 1 like	TRMT1L	0,92
PRAME family member 25	PRAMEF25	0,92
PDS5 cohesin associated factor B	PDS5B	0,92
pelota homolog (Drosophila); integrin alpha 1	PELO; ITGA1	0,92
pre-B lymphocyte 1	VPREB1	0,92
small integral membrane protein 13	SMIM13	0,92
transient receptor potential cation channel, subfamily V, member 6	TRPV6	0,92
forkhead box I2	FOXI2	0,92
phospholipase A2, group VI (cytosolic, calcium-independent)	PLA2G6	0,92
Transcript Identified by AceView; Memczak2013 ANTISENSE, CDS, coding, INTERNAL, UTR3 best transcript NM_004526	goyborbu; MCM2	0,92
oligosaccharyltransferase complex subunit 4 (non-catalytic)	OST4	0,92
serine/threonine protein kinase 26	STK26	0,92
ribonucleotide reductase M2	RRM2	0,92
plexin B3	PLXNB3	0,92
PDLIM1 interacting kinase 1 like	PDIK1L	0,92
COP9 signalosome subunit 8	COPS8	0,92
zinc finger protein 385B	ZNF385B	0,92
chromosome 6 open reading frame 62	C6orf62	0,92
synaptopodin 2-like	SYNPO2L	0,92
tetratricopeptide repeat, ankyrin repeat and coiled-coil containing 1	TANC1	0,92
annexin A7	ANXA7	0,92
OTU deubiquitinase 5	OTUD5	0,92
hydroxymethylbilane synthase	HMBS	0,92
transmembrane protein 175	TMEM175	0,92
neurensin 2	NRSN2	0,92
tumor protein p53	TP53	0,92
serine hydroxymethyltransferase 1 (soluble); microRNA 6778	SHMT1; MIR6778	0,92
tripartite motif containing 43	TRIM43	0,92
cerberus 1, DAN family BMP antagonist	CER1	0,92

stereocilin	STRC	0,92
myozenin 3	MYOZ3	0,92
thiosulfate sulfurtransferase (rhodanese)-like domain containing 2	TSTD2	0,92
replication factor C subunit 1	RFC1	0,92
ST6 (alpha-N-acetyl-neuraminyl-2,3-beta-galactosyl-1,3)-N-acetylgalactosaminide alpha-2,6-sialyltransferase 1	ST6GALNAC1	0,92
chromosome 10 open reading frame 111	C10orf111	0,92
succinate dehydrogenase complex, subunit C, integral membrane protein, 15kDa	SDHC	0,92
2-oxoglutarate and iron-dependent oxygenase domain containing 2	OGFOD2	0,92
histocompatibility (minor) serpin domain containing	HMSD	0,92
RUN and SH3 domain containing 1	RUSC1	0,92
cullin 5	CUL5	0,92
metaxin 2	MTX2	0,92
arginine-glutamic acid dipeptide (RE) repeats	RERE	0,92
short stature homeobox	SHOX	0,92
potassium channel, voltage gated modifier subfamily G, member 2	KCNG2	0,92
sema domain, immunoglobulin domain (Ig), transmembrane domain (TM) and short cytoplasmic domain, (semaphorin) 4F	SEMA4F	0,92
ribosomal protein SA; small nucleolar RNA, H/ACA box 62; small nucleolar RNA, H/ACA box 6	RPSA; SNORA62; SNORA6	0,92
complement component (3d/Epstein Barr virus) receptor 2	CR2	0,92
LDL receptor related protein 6	LRP6	0,92
arginase 1	ARG1	0,92
relaxin/insulin-like family peptide receptor 4	RXFP4	0,92
Memczak2013 ALT_ACCEPTOR, ALT_DONOR, coding, INTERNAL, intronic best transcript NM_003502	AXIN1	0,92
5-hydroxymethylcytosine (hmC) binding, ES cell-specific	HMCES	0,92
chromosome 7 open reading frame 49	C7orf49	0,92
pleckstrin homology domain containing, family A member 8 pseudogene 1	PLEKHA8P1	0,92
histamine receptor H2	HRH2	0,92
phospholipase A2, group VII (platelet-activating factor acetylhydrolase, plasma)	PLA2G7	0,92
serum deprivation response	SDPR	0,92
histone cluster 1, H4j	HIST1H4J	0,92
potassium channel tetramerization domain containing 17	KCTD17	0,92
t-complex 10-like 2	TCP10L2	0,92
ubiquitin-like with PHD and ring finger domains 2, E3 ubiquitin protein ligase	UHRF2	0,92
glycosyltransferase 6 domain containing 1	GLT6D1	0,92

X-linked Kx blood group related, X-linked	XKRX	0,92
topoisomerase (DNA) II beta; microRNA 4442	TOP2B; MIR4442	0,92
X-ray repair complementing defective repair in Chinese hamster cells 5 (double-strand-break rejoining)	XRCC5	0,92
parathyroid hormone	PTH	0,92
transmembrane protein 145	TMEM145	0,92
scavenger receptor class B, member 2	SCARB2	0,92
peptidyl-tRNA hydrolase 1 homolog	PTRH1	0,92
Src homology 2 domain containing adaptor protein B	SHB	0,92
RBPJ interacting and tubulin associated 1	RITA1	0,92
myosin, heavy chain 2, skeletal muscle, adult	MYH2	0,92
S100 calcium binding protein A16	S100A16	0,92
pre-mRNA processing factor 38B	PRPF38B	0,92
breast cancer 1, early onset	BRCA1	0,92
olfactory receptor, family 1, subfamily L, member 4	OR1L4	0,92
PH domain and leucine rich repeat protein phosphatase 2	PHLPP2	0,92
polypeptide N-acetylgalactosaminyltransferase 2	GALNT2	0,92
lysozyme-like 4	LYZL4	0,92
family with sequence similarity 96, member A	FAM96A	0,92
solute carrier family 25 (glutamate carrier), member 18	SLC25A18	0,92
ectodermal-neural cortex 1 (with BTB domain)	ENC1	0,92
chondroadherin-like	CHADL	0,92
tumor protein D52	TPD52	0,92
ring finger protein 185	RNF185	0,92
short stature homeobox 2	SHOX2	0,92
ataxin 3-like	ATXN3L	0,92
cytokine receptor-like factor 3	CRLF3	0,92
cAMP-regulated phosphoprotein 21kDa	ARPP21	0,92
long intergenic non-protein coding RNA 1565	LINC01565	0,92
BPI fold containing family B, member 4	BPIFB4	0,92
NAD synthetase 1	NADSYN1	0,92
Kruppel-like factor 6	KLF6	0,92
acyl-CoA thioesterase 9	ACOT9	0,92
membrane-spanning 4-domains, subfamily A, member 18	MS4A18	0,92
endoplasmic reticulum protein 44	ERP44	0,92
glycosylphosphatidylinositol anchored molecule like	GML	0,92
alpha 1,4-galactosyltransferase	A4GALT	0,92
ARP2 actin-related protein 2 homolog (yeast)	ACTR2	0,92
potassium channel, voltage gated KQT-like subfamily Q, member 1	KCNQ1	0,92
delta-like 1 homolog (Drosophila)	DLK1	0,92
coiled-coil domain containing 146	CCDC146	0,92
zinc finger protein 346	ZNF346	0,92
aquaporin 11	AQP11	0,92

phospholipase C-like 1	PLCL1	0,92
zinc finger protein 770	ZNF770	0,92
ADP-ribosyltransferase 3	ART3	0,92
syntaxin binding protein 3	STXBP3	0,92
eukaryotic translation initiation factor 2-alpha kinase 1	EIF2AK1	0,92
naked cuticle homolog 1 (Drosophila)	NKD1	0,92
CDK2-associated, cullin domain 1	CACUL1	0,92
Transcript Identified by AceView, Entrez Gene ID(s) 143686	SESN3	0,92
actin related protein 2/3 complex subunit 5	ARPC5	0,92
bone morphogenetic protein receptor type IA	BMPR1A	0,92
zinc finger protein 606	ZNF606	0,92
long intergenic non-protein coding RNA 552	LINC00552	0,92
immunoglobulin superfamily, member 3	IGSF3	0,92
olfactory receptor, family 1, subfamily B, member 1 (gene/pseudogene)	OR1B1	0,92
kin of IRRE like 3 (Drosophila)	KIRREL3	0,92
BPI fold containing family C	BPIFC	0,92
sorting nexin 9	SNX9	0,92
GC-rich promoter binding protein 1	GPBP1	0,92
mitochondrial ribosomal protein L13	MRPL13	0,92
Ral GTPase activating protein, alpha subunit 2 (catalytic)	RALGAPA2	0,92
olfactory receptor, family 10, subfamily AC, member 1 (gene/pseudogene) [Source:HGNC Symbol;Acc:HGNC:14758]	OR10AC1	0,92
acyl-CoA binding domain containing 5	ACBD5	0,92
coiled-coil domain containing 87	CCDC87	0,92
La ribonucleoprotein domain family, member 4	LARP4	0,92
nardilysin convertase; microRNA 761	NRDC; MIR761	0,92
tachykinin, precursor 1	TAC1	0,92
inhibitor of growth family member 3	ING3	0,92
forkhead box R1	FOXR1	0,92
CTBP1 antisense RNA 2 (head to head)	CTBP1-AS2	0,92
WD repeat domain 7	WDR7	0,92
KDEL (Lys-Asp-Glu-Leu) endoplasmic reticulum protein retention receptor 3	KDELR3	0,92
NADH dehydrogenase, subunit 3 (complex I)	ND3	0,92
apolipoprotein L, 5	APOL5	0,92
CD6 molecule	CD6	0,92
B9 protein domain 2	B9D2	0,92
ERGIC and golgi 2	ERGIC2	0,92
ribosomal protein L23; small nucleolar RNA, H/ACA box 21	RPL23; SNORA21	0,92
BUB3 mitotic checkpoint protein	BUB3	0,92
centrosomal protein 162kDa	CEP162	0,92
SLX4 structure-specific endonuclease subunit	SLX4	0,92
peptidase (mitochondrial processing) beta	PMPCB	0,92

translocase of outer mitochondrial membrane 22 homolog (yeast)	TOMM22	0,92
interleukin 2 receptor, beta	IL2RB	0,92
short stature homeobox	SHOX	0,92
phosphatidylinositol-5-phosphate 4-kinase, type II, gamma	PIP4K2C	0,92
intracisternal A particle-promoted polypeptide	IPP	0,92
cholinergic receptor, nicotinic beta 1	CHRNB1	0,92
MAS-related GPR, member D	MRGPRD	0,92
claudin 7	CLDN7	0,92
centrosomal protein 19kDa	CEP19	0,92
replication timing regulatory factor 1	RIF1	0,92
lymphocyte antigen 6 complex, locus E	LY6E	0,92
ubiquitin-conjugating enzyme E2W (putative)	UBE2W	0,92
nuclear cap binding protein subunit 2-like	NCBP2L	0,92
transducin (beta)-like 1 X-linked receptor 1	TBL1XR1	0,92
zinc finger protein 12	ZNF12	0,92
glutathione S-transferase mu 3 (brain)	GSTM3	0,92
zinc finger protein 502	ZNF502	0,92
nurim (nuclear envelope membrane protein)	NRM	0,92
methyl-CpG binding domain protein 3-like 4	MBD3L4	0,92
oligosaccharyltransferase complex subunit pseudogene 1	OSTCP1	0,92
poly(A) binding protein, cytoplasmic 1-like 2B	PABPC1L2B	0,92
p21 protein (Cdc42/Rac)-activated kinase 7	PAK7	0,92
BEN domain containing 4	BEND4	0,92
Transcript Identified by AceView, Entrez Gene ID(s) 7881	KCNAB1	0,92
ets variant 4	ETV4	0,92
SLAM family member 8	SLAMF8	0,92
2-aminoethanethiol (cysteamine) dioxygenase	ADO	0,92
NLR family, pyrin domain containing 9	NLRP9	0,92
MFNG O-fucosylpeptide 3-beta-N-acetylglucosaminyltransferase	MFNG	0,92
membrane protein, palmitoylated 7	MPP7	0,92
ribosomal protein L9 pseudogene 7	RPL9P7	0,92
EDAR-associated death domain	EDARADD	0,92
stomatin (EPB72)-like 1	STOML1	0,92
UDP-N-acetylglucosamine pyrophosphorylase 1	UAP1	0,92
ATPase, H+ transporting, lysosomal accessory protein 1-like	ATP6AP1L	0,92
S-antigen; retina and pineal gland (arrestin)	SAG	0,92
CD300 molecule-like family member d	CD300LD	0,92
myostatin	MSTN	0,92
sulfatase 1	SULF1	0,92
CD320 molecule	CD320	0,92
nidogen 1	NID1	0,92
chromosome 19 open reading frame 60	C19orf60	0,92
proteasome subunit beta 7	PSMB7	0,92

Williams-Beuren syndrome chromosome region 17	WBSCR17	0,92
GRAM domain containing 1B	GRAMD1B	0,92
syntaxin 2	STX2	0,92
Memczak2013 ALT_ACCEPTOR, ALT_DONOR, coding, INTERNAL, intronic best transcript NM_001130110	SETBP1	0,92
protease, serine 45	PRSS45	0,92
PWWP domain containing 2A	PWWP2A	0,92
Fanconi anemia complementation group L	FANCL	0,92
pre-mRNA processing factor 38A	PRPF38A	0,92
ring finger protein 180	RNF180	0,92
solute carrier family 44, member 3	SLC44A3	0,92
keratin associated protein 12-1	KRTAP12-1	0,92
CD1d molecule	CD1D	0,92
synapsin I	SYN1	0,92
formin 2; alcohol dehydrogenase 5 (class III), chi polypeptide, pseudogene 3	FMN2; ADH5P3	0,92
eukaryotic translation initiation factor 1A domain containing	EIF1AD	0,92
replication factor C subunit 5	RFC5	0,92
chemokine (C-C motif) receptor 3	CCR3	0,92
olfactory receptor, family 2, subfamily A, member 5	OR2A5	0,92
heparanase 2 (inactive)	HPSE2	0,92
HECT domain containing E3 ubiquitin protein ligase 2	HECTD2	0,92
glutaminyl-tRNA synthetase; microRNA 6890	QARS; MIR6890	0,92
centromere protein N	CENPN	0,92
interferon, kappa	IFNK	0,92
proline rich 27	PRR27	0,92
basic leucine zipper nuclear factor 1	BLZF1	0,92
ectonucleotide pyrophosphatase/phosphodiesterase 3	ENPP3	0,92
Mix paired-like homeobox	MIXL1	0,92
olfactory receptor, family 7, subfamily A, member 5	OR7A5	0,92
chromosome 8 open reading frame 33	C8orf33	0,92
IgA-inducing protein	IGIP	0,92
zinc finger protein 575	ZNF575	0,92
CTAGE family, member 8	CTAGE8	0,92
inscuteable homolog (Drosophila)	INSC	0,92
EMSY BRCA2-interacting transcriptional repressor	EMSY	0,92
PRAME family member 25; PRAME family member 26	PRAMEF25; PRAMEF26	0,92
mannosyl (alpha-1,6-)-glycoprotein beta-1,6-N-acetyl-glucosaminyltransferase	MGAT5	0,92
fatty acid synthase	FASN	0,92
Eukaryotic translation initiation factor 3 subunit F [Source:UniProtKB/Swiss-Prot;Acc:O00303]	EIF3F	0,92
chromosome 2 open reading frame 40	C2orf40	0,92

Memczak2013 ANTISENSE, coding, INTERNAL, intronic best transcript NM_033360	KRAS	0,92
serine/arginine-rich splicing factor 11	SRSF11	0,92
methylthioadenosine phosphorylase	MTAP	0,92
G protein-coupled receptor 173	GPR173	0,92
ankyrin 3, node of Ranvier (ankyrin G)	ANK3	0,92
prokineticin receptor 2	PROKR2	0,92
small G protein signaling modulator 1	SGSM1	0,92
COMM domain containing 9	COMMD9	0,92
heterogeneous nuclear ribonucleoprotein U-like 2	HNRNPUL2	0,92
hydroxy-delta-5-steroid dehydrogenase, 3 beta- and steroid delta-isomerase 2	HSD3B2	0,92
interleukin 5 receptor, alpha	IL5RA	0,92
transmembrane protease, serine 4	TMPRSS4	0,92
cell division cycle associated 8	CDCA8	0,92
pancreatic lipase	PNLIP	0,92
adenylate kinase 4	AK4	0,92
interferon, alpha-inducible protein 27	IFI27	0,92
TAF4 RNA polymerase II, TATA box binding protein (TBP)-associated factor, 135kDa	TAF4	0,92
olfactory receptor, family 5, subfamily AP, member 2	OR5AP2	0,92
Memczak2013 ANTISENSE, coding, INTERNAL, intronic best transcript NM_024829	PLBD1	0,92
TSPY-like 2	TSPYL2	0,92
adhesion molecule, interacts with CXADR antigen 1	AMICA1	0,92
FER1L6 antisense RNA 1	FER1L6-AS1	0,92
radial spoke head 1 homolog (Chlamydomonas)	RSPH1	0,92
obscurin, cytoskeletal calmodulin and titin-interacting RhoGEF	OBSCN	0,92
dedicator of cytokinesis 1	DOCK1	0,92
UL16 binding protein 2	ULBP2	0,92
zinc finger protein 124	ZNF124	0,92
ras homolog family member T1	RHOT1	0,92
Transcript Identified by AceView, Entrez Gene ID(s) 375775	PNPLA7	0,92
nucleolar protein 7	NOL7	0,92
methyl-CpG binding domain protein 3	MBD3	0,92
tetratricopeptide repeat domain 22	TTC22	0,92
chemokine (C-C motif) ligand 4-like 2; chemokine (C-C motif) ligand 4; chemokine (C-C motif) ligand 4-like 1	CCL4L2; CCL4; CCL4L1	0,92
Rho-associated, coiled-coil containing protein kinase 2	ROCK2	0,92
LDL receptor related protein 6	LRP6	0,92
histone cluster 1, H2aj	HIST1H2AJ	0,92
ketohexokinase	KHK	0,92
pantothenate kinase 4	PANK4	0,92
phosphatidylethanolamine-binding protein 4	PEBP4	0,92

Zhang2013 ALT_ACCEPTOR, ALT_DONOR, coding, INTERNAL, intronic best transcript NM_003934	FUBP3	0,92
olfactory receptor, family 11, subfamily H, member 6	OR11H6	0,92
spermatogenesis associated 4	SPATA4	0,92
POC1 centriolar protein A	POC1A	0,92
RAD1 checkpoint DNA exonuclease	RAD1	0,92
hydroxysteroid (17-beta) dehydrogenase 2	HSD17B2	0,92
LIM and senescent cell antigen-like domains 2	LIMS2	0,92
adenylate kinase 3	AK3	0,92
olfactory receptor, family 4, subfamily F, member 17	OR4F17	0,92
solute carrier family 39 (zinc transporter), member 1	SLC39A1	0,92
pyruvate dehydrogenase (lipoamide) alpha 2	PDHA2	0,92
SP110 nuclear body protein	SP110	0,92
chromosome 1 open reading frame 145	C1orf145	0,92
ribosomal protein S6 kinase, 90kDa, polypeptide 1	RPS6KA1	0,92
protein-L-isoaspartate (D-aspartate) O-methyltransferase domain containing 2	PCMTD2	0,92
von Hippel-Lindau binding protein 1	VBP1	0,92
chromosome 11 open reading frame 45	C11orf45	0,92
TBC1 domain family, member 10A	TBC1D10A	0,92
F-box protein 31	FBXO31	0,92
SERTA domain containing 4	SERTAD4	0,92
spermatogenesis associated 33	SPATA33	0,92
ISY1 splicing factor homolog	ISY1	0,92
achaete-scute family bHLH transcription factor 5	ASCL5	0,92
calmodulin-like 4	CALML4	0,92
notch 3	NOTCH3	0,92
Dmx-like 2	DMXL2	0,92
immunoresponsive 1 homolog (mouse)	IRG1	0,92
Sec61 translocon alpha 1 subunit	SEC61A1	0,92
cysteine rich, DPF motif domain containing 1	CDPF1	0,92
hyaluronan synthase 1	HAS1	0,92
filamin A, alpha	FLNA	0,92
neural precursor cell expressed, developmentally down-regulated 9	NEDD9	0,92
cancer/testis antigen family 47, member A7; cancer/testis antigen family 47, member A5; cancer/testis antigen family 47, member A6	CT47A7; CT47A5; CT47A6	0,92
leucine rich repeat containing 59	LRRC59	0,92
long intergenic non-protein coding RNA 1272	LINC01272	0,92
bromodomain containing 2	BRD2	0,92
zinc finger protein 101	ZNF101	0,92
mediator complex subunit 29	MED29	0,92
cell death-inducing DFFA-like effector b	CIDEB	0,92
pyrimidinergic receptor P2Y, G-protein coupled, 4	P2RY4	0,92

ADP-ribosylation factor like GTPase 6 interacting protein 6	ARL6IP6	0,92
interferon, alpha 7	IFNA7	0,92
chromosome 17 open reading frame 50	C17orf50	0,92
SKI2 homolog, superkiller viralicidic activity 2-like	SKIV2L	0,92
SFT2 domain containing 3	SFT2D3	0,92
nuclear fragile X mental retardation protein interacting protein 2	NUFIP2	0,92
forkhead box M1	FOXM1	0,92
reticulon 4	RTN4	0,92
mex-3 RNA binding family member C	MEX3C	0,92
electron-transfer-flavoprotein, beta polypeptide	ETFB	0,92
PIH1 domain containing 1	PIH1D1	0,92
adenylosuccinate lyase	ADSL	0,92
kinesin family member 26A	KIF26A	0,92
multiple endocrine neoplasia I	MEN1	0,92
dynein, light chain, roadblock-type 2	DYNLRB2	0,92
Transcript Identified by AceView, Entrez Gene ID(s) 89910	UBE3B	0,92
FIC domain containing	FICD	0,92
UDP-GlcNAc:betaGal beta-1,3-N-acetylglucosaminyltransferase-like 1	B3GNTL1	0,92
ELOVL fatty acid elongase 7	ELOVL7	0,92
mastermind-like transcriptional coactivator 3	MAML3	0,92
laminin, gamma 3	LAMC3	0,92
DiGeorge syndrome critical region gene 5 (non-protein coding); DiGeorge syndrome critical region gene 9 (non-protein coding)	DGCR5; DGCR9	0,92
ATP synthase, H+ transporting, mitochondrial Fo complex subunit F2	ATP5J2	0,92
zinc finger protein 653	ZNF653	0,92
RNA binding protein with multiple splicing	RBPMS	0,92
microtubule-associated protein 1 light chain 3 beta	MAP1LC3B	0,92
transmembrane protein 19	TMEM19	0,92
cyclin Y like 1; microRNA 4775	CCNYL1; MIR4775	0,92
SET domain containing 5	SETD5	0,92
FEZ family zinc finger 2	FEZF2	0,92
TBC1 domain family, member 3L	TBC1D3L	0,92
Rho GTPase activating protein 15	ARHGAP15	0,92
solute carrier family 23 (ascorbic acid transporter), member 2	SLC23A2	0,92
heat shock 27kDa protein 3	HSPB3	0,92
lysine (K)-specific demethylase 5B	KDM5B	0,92
phosphoglucomutase 5	PGM5	0,92
RAB9B, member RAS oncogene family	RAB9B	0,92
SET domain containing 9	SETD9	0,92
TEK tyrosine kinase, endothelial	TEK	0,92
formyl peptide receptor 1	FPR1	0,92

Bardet-Biedl syndrome 9	BBS9	0,92
aspartyl-tRNA synthetase	DARS	0,92
family with sequence similarity 35, member A	FAM35A	0,92
lymphocyte expansion molecule	LEXM	0,92
bradykinin receptor B2	BDKRB2	0,92
family with sequence similarity 20, member B	FAM20B	0,92
keratin associated protein 22-1	KRTAP22-1	0,92
solute carrier family 4, sodium bicarbonate transporter, member 10	SLC4A10	0,92
adenylate cyclase 1 (brain)	ADCY1	0,92
myosin VIIA and Rab interacting protein	MYRIP	0,92
spermine synthase	SMS	0,92
prostaglandin E synthase 3 (cytosolic)	PTGES3	0,92
transmembrane protein 204	TMEM204	0,92
Transcript Identified by AceView, Entrez Gene ID(s) 23122	CLASP2	0,92
SAM and SH3 domain containing 1	SASH1	0,92
chromosome 17 open reading frame 51	C17orf51	0,92
ankyrin repeat domain 30B	ANKRD30B	0,92
TBC1 domain family, member 23	TBC1D23	0,92
adhesion G protein-coupled receptor A2	ADGRA2	0,92
trace amine associated receptor 6	TAAR6	0,92
reticulon 4 receptor	RTN4R	0,92
chondroitin sulfate N-acetylgalactosaminyltransferase 2	CSGALNACT2	0,92
MIR670 host gene; hydroxysteroid (17-beta) dehydrogenase 12	MIR670HG; HSD17B12	0,92
Rhox homeobox family, member 2; Rhox homeobox family, member 2B	RHOXF2; RHOXF2B	0,92
eukaryotic translation initiation factor 4E family member 3	EIF4E3	0,92
olfactory receptor, family 1, subfamily J, member 4	OR1J4	0,92
myosin, heavy chain 7, cardiac muscle, beta	MYH7	0,92
phospholipid transfer protein	PLTP	0,92
calcium channel, voltage-dependent, beta 1 subunit	CACNB1	0,92
Jeck2013 ALT_DONOR, coding, INTERNAL, intronic best transcript NM_147780	CTSB	0,92
glycine cleavage system protein H (aminomethyl carrier)	GCSH	0,92
Zhang2013 ALT_ACCEPTOR, ALT_DONOR, coding, INTERNAL, intronic best transcript NM_030653	DDX11	0,92
muscleblind-like splicing regulator 2	MBNL2	0,92
bromodomain containing 1	BRD1	0,92
membrane associated ring finger 11	MARCH11	0,92
long intergenic non-protein coding RNA 269	LINC00269	0,92
coatomer protein complex subunit zeta 2	COPZ2	0,92
complement component 8, gamma polypeptide	C8G	0,92
unc-93 homolog A (C. elegans)	UNC93A	0,92
discs, large homolog 4 (Drosophila)	DLG4	0,92

superoxide dismutase 2, mitochondrial	SOD2	0,92
LIM domain 7	LMO7	0,92
ZFP92 zinc finger protein	ZFP92	0,92
cornichon family AMPA receptor auxiliary protein 3	CNIH3	0,92
patatin-like phospholipase domain containing 8	PNPLA8	0,92
phospholipase B1	PLB1	0,92
empty spiracles homeobox 1	EMX1	0,92
purinergic receptor P2Y, G-protein coupled, 10	P2RY10	0,92
ribosomal protein S14	RPS14	0,92
Kirsten rat sarcoma viral oncogene homolog	KRAS	0,92
metaxin 3	MTX3	0,92
polymerase (RNA) I polypeptide B	POLR1B	0,92
family with sequence similarity 195, member A	FAM195A	0,92
CDC28 protein kinase regulatory subunit 2	CKS2	0,92
cyclin-dependent kinase 2 interacting protein	CINP	0,92
GA binding protein transcription factor alpha subunit	GABPA	0,92
CEP295 N-terminal like; TIMP metallopeptidase inhibitor 2	CEP295NL; TIMP2	0,92
V-set and transmembrane domain containing 2A; VSTM2A overlapping transcript 1	VSTM2A; VSTM2A-OT1	0,92
chemokine (C-X-C motif) ligand 11	CXCL11	0,92
CD101 molecule	CD101	0,92
calcium homeostasis endoplasmic reticulum protein	CHERP	0,92
DAN domain family member 5, BMP antagonist	DAND5	0,92
solute carrier family 25 (mitochondrial carnitine/acylcarnitine carrier), member 29	SLC25A29	0,92
CUB domain containing protein 2	CDCP2	0,92
tankyrase, TRF1-interacting ankyrin-related ADP-ribose polymerase	TNKS	0,92
tumor necrosis factor receptor superfamily, member 10b	TNFRSF10B	0,92
heat shock protein, alpha-crystallin-related, B9	HSPB9	0,92
ferritin, light polypeptide	FTL	0,92
SENP3-EIF4A1 readthrough (NMD candidate)	SENP3-EIF4A1	0,92
large tumor suppressor kinase 1	LATS1	0,92
striatin, calmodulin binding protein 4	STRN4	0,92
CD300 molecule-like family member g	CD300LG	0,92
ORMDL sphingolipid biosynthesis regulator 3	ORMDL3	0,92
lamin B1	LMNB1	0,92
zinc finger protein 347	ZNF347	0,92
nuclear casein kinase and cyclin-dependent kinase substrate 1	NUCKS1	0,92
defensin, beta 123	DEFB123	0,92
chemokine (C-X-C motif) receptor 6	CXCR6	0,92
solute carrier family 7 (cationic amino acid transporter, y+ system), member 2	SLC7A2	0,92
inter-alpha-trypsin inhibitor heavy chain 3	ITIH3	0,92

sterol O-acyltransferase 1	SOAT1	0,92
NIMA-related kinase 7	NEK7	0,92
zinc finger protein 598	ZNF598	0,92
transcription factor EC	TFEC	0,92
UFM1-specific ligase 1	UFL1	0,92
ribonuclease, RNase A family, k6	RNASE6	0,92
zinc finger and BTB domain containing 25	ZBTB25	0,92
Fas (TNFRSF6) associated factor 1	FAF1	0,92
solute carrier family 4 (anion exchanger), member 2	SLC4A2	0,92
SEC22 homolog C, vesicle trafficking protein	SEC22C	0,92
activating transcription factor 6	ATF6	0,92
sarcoglycan delta	SGCD	0,92
APC membrane recruitment protein 3	AMER3	0,92
syntaxin 4	STX4	0,92
autophagy related 16-like 1	ATG16L1	0,92
caspase recruitment domain family, member 14	CARD14	0,91
DNA (cytosine-5-)-methyltransferase 3-like	DNMT3L	0,91
ataxin 7-like 3B	ATXN7L3B	0,91
tetraspanin 9	TSPAN9	0,91
adaptor protein, phosphotyrosine interaction, PH domain and leucine zipper containing 1	APPL1	0,91
ring finger protein 166	RNF166	0,91
ring finger and CHY zinc finger domain containing 1, E3 ubiquitin protein ligase	RCHY1	0,91
centrosomal protein 70kDa	CEP70	0,91
signal peptide peptidase like 2B	SPPL2B	0,91
poly(rC) binding protein 1	PCBP1	0,91
ELOVL fatty acid elongase 6	ELOVL6	0,91
casein beta	CSN2	0,91
protein inhibitor of activated STAT 1	PIAS1	0,91
2,3-cyclic nucleotide 3 phosphodiesterase	CNP	0,91
hes family bHLH transcription factor 7	HES7	0,91
excision repair cross-complementation group 6-like	ERCC6L	0,91
receptor (G protein-coupled) activity modifying protein 3	RAMP3	0,91
mitogen-activated protein kinase 9	MAPK9	0,91
cysteine and glycine-rich protein 1	CSRP1	0,91
long intergenic non-protein coding RNA 1126	LINC01126	0,91
trafficking protein particle complex 3	TRAPPC3	0,91
Sep (O-phosphoserine) tRNA:Sec (selenocysteine) tRNA synthase	SEPSECS	0,91
phospholysine phosphohistidine inorganic pyrophosphate phosphatase	LHPP	0,91
WD repeat domain, phosphoinositide interacting 1	WIPI1	0,91
coiled-coil domain containing 84	CCDC84	0,91
interleukin 1 receptor accessory protein	IL1RAP	0,91

methyl-CpG binding domain protein 3-like 3	MBD3L3	0,91
protein phosphatase 1, regulatory (inhibitor) subunit 14D	PPP1R14D	0,91
inner centromere protein	INCENP	0,91
Rho/Rac guanine nucleotide exchange factor 2	ARHGEF2	0,91
ring-box 1, E3 ubiquitin protein ligase	RBX1	0,91
PDZ domain containing 11	PDZD11	0,91
R3H domain containing 4	R3HDM4	0,91
solute carrier family 25, member 53	SLC25A53	0,91
interleukin 20 receptor beta	IL20RB	0,91
visinin like 1	VSNL1	0,91
citron rho-interacting serine/threonine kinase; microRNA 1178	CIT; MIR1178	0,91
Memczak2013 ANTISENSE, coding, INTERNAL, UTR3 best transcript NM_014737	RASSF2	0,91
Transcript Identified by AceView, Entrez Gene ID(s) 130507	UBR3	0,91
phosphodiesterase 11A	PDE11A	0,91
annexin A6	ANXA6	0,91
Ral GTPase activating protein, alpha subunit 1 (catalytic)	RALGAPA1	0,91
aggrecan	ACAN	0,91
gastrin-releasing peptide	GRP	0,91
cordon-bleu WH2 repeat protein like 1	COBLL1	0,91
SPT6 homolog, histone chaperone	SUPT6H	0,91
F-box protein 47	FBXO47	0,91
upstream binding transcription factor, RNA polymerase I-like 1	UBTFL1	0,91
alcohol dehydrogenase 7 (class IV), mu or sigma polypeptide	ADH7	0,91
zinc finger protein 852	ZNF852	0,91
nicalin	NCLN	0,91
Rh-associated glycoprotein	RHAG	0,91
Ras association (RalGDS/AF-6) domain family (N-terminal) member 7	RASSF7	0,91
HMG box domain containing 4	HMGXB4	0,91
CHURC1-FNTB readthrough	CHURC1-FNTB	0,91
regulatory solute carrier protein, family 1, member 1; DNA-damage inducible 1 homolog 2	RSC1A1; DDI2	0,91
NADH dehydrogenase (ubiquinone) 1 alpha subcomplex, 7, 14.5kDa	NDUFA7	0,91
TATA box binding protein (TBP)-associated factor, RNA polymerase I, A, 48kDa	TAF1A	0,91
regulator of chromosome condensation 2	RCC2	0,91
Yip1 interacting factor homolog B (S. cerevisiae)	YIF1B	0,91
olfactory receptor, family 5, subfamily B, member 12	OR5B12	0,91
diaphanous-related formin 2	DIAPH2	0,91
homeobox A1	HOXA1	0,91
solute carrier family 35, member E3	SLC35E3	0,91
transcobalamin II	TCN2	0,91
Yip1 interacting factor homolog A (S. cerevisiae)	YIF1A	0,91

EPH receptor A6	EPHA6	0,91
BRICK1, SCAR/WAVE actin-nucleating complex subunit	BRK1	0,91
anoctamin 7	ANO7	0,91
ubiquitin specific peptidase 17-like family member 22; ubiquitin specific peptidase 17-like family member 20	USP17L22; USP17L20	0,91
uncharacterized LOC151121; novel transcript	LOC151121; AC079586.1	0,91
DEAD (Asp-Glu-Ala-Asp) box polypeptide 39A	DDX39A	0,91
nuclear cap binding protein subunit 2	NCBP2	0,91
acyl-CoA dehydrogenase, C-4 to C-12 straight chain	ACADM	0,91
protease, serine 53	PRSS53	0,91
kinocilin	KNCN	0,91
KIAA1841	KIAA1841	0,91
ectonucleotide pyrophosphatase/phosphodiesterase 6	ENPP6	0,91
serine peptidase inhibitor, Kazal type 13 (putative)	SPINK13	0,91
chromosome 14 open reading frame 119	C14orf119	0,91
transmembrane protein 218	TMEM218	0,91
transmembrane p24 trafficking protein 3	TMED3	0,91
small nuclear ribonucleoprotein polypeptide C	SNRPC	0,91
ras homolog family member B	RHOB	0,91
transcription factor CP2	TFCP2	0,91
hydroxysteroid (17-beta) dehydrogenase 1	HSD17B1	0,91
integrin alpha 11	ITGA11	0,91
solute carrier family 30 (zinc transporter), member 7	SLC30A7	0,91
DC-STAMP domain containing 2	DCST2	0,91
F-box protein 46	FBXO46	0,91
zinc finger protein 806	ZNF806	0,91
TP53 target 3C	TP53TG3C	0,91
calcium-binding protein, spermatid-specific 1	CABS1	0,91
dual specificity tyrosine-(Y)-phosphorylation regulated kinase 2	DYRK2	0,91
1-aminocyclopropane-1-carboxylate synthase homolog (inactive)	ACCS	0,91
nucleoporin 188kDa	NUP188	0,91
cytochrome c-1	CYC1	0,91
dynein, axonemal, assembly factor 3	DNAAF3	0,91
hedgehog interacting protein	HHIP	0,91
inversin	INVS	0,91
lysophosphatidylglycerol acyltransferase 1	LPGAT1	0,91
transmembrane protein 243, mitochondrial	TMEM243	0,91
GM2 ganglioside activator	GM2A	0,91
chromosome 2 open reading frame 68	C2orf68	0,91
family with sequence similarity 195, member A	FAM195A	0,91
leucine rich repeat containing 31	LRRC31	0,91
WD repeat containing, antisense to TP73	WRAP73	0,91
forkhead box Q1	FOXQ1	0,91

protein tyrosine phosphatase, receptor type, B	PTPRB	0,91
family with sequence similarity 127, member A	FAM127A	0,91
sec1 family domain containing 1	SCFD1	0,91
tankyrase, TRF1-interacting ankyrin-related ADP-ribose polymerase	TNKS	0,91
solute carrier family 25 (S-adenosylmethionine carrier), member 26	SLC25A26	0,91
chloride channel, voltage-sensitive 7	CLCN7	0,91
small nuclear RNA activating complex polypeptide 4	SNAPC4	0,91
interleukin 24	IL24	0,91
guanylate cyclase activator 1B (retina)	GUCA1B	0,91
nebulin-related anchoring protein	NRAP	0,91
neuropeptide Y receptor Y1	NPY1R	0,91
NHL repeat containing 3	NHLRC3	0,91
coagulation factor XI	F11	0,91
ubiquitination factor E4A	UBE4A	0,91
late cornified envelope 6A	LCE6A	0,91
plexin D1	PLXND1	0,91
NOP2/Sun domain family, member 4	NSUN4	0,91
protein kinase, AMP-activated, alpha 1 catalytic subunit	PRKAA1	0,91
somatostatin receptor 1	SSTR1	0,91
aspartoacylase	ASPA	0,91
naked cuticle homolog 2 (Drosophila)	NKD2	0,91
myosin, heavy chain 10, non-muscle	MYH10	0,91
potassium channel tetramerization domain containing 2	KCTD2	0,91
ADAM metallopeptidase with thrombospondin type 1 motif 13	ADAMTS13	0,91
septin 1	sept-01	0,91
msh homeobox 1	MSX1	0,91
zinc finger protein 704	ZNF704	0,91
LYR motif containing 5	LYRM5	0,91
zinc finger, DHHC-type containing 9	ZDHHC9	0,91
KRAB box domain containing 4	KRBOX4	0,91
chromosome 4 open reading frame 51	C4orf51	0,91
zyg-11 related, cell cycle regulator	ZER1	0,91
olfactory receptor, family 2, subfamily S, member 2 (gene/pseudogene)	OR2S2	0,91
formin homology 2 domain containing 1	FHOD1	0,91
CD24 molecule	CD24	0,91
kallikrein related peptidase 6	KLK6	0,91
glutamine rich 2	QRICH2	0,91
tubulin, beta 4A class IVa	TUBB4A	0,91
plasminogen-like B1; plasminogen-like B2	PLGLB1; PLGLB2	0,91
prostaglandin F receptor (FP)	PTGFR	0,91
hyaluronan binding protein 4	HABP4	0,91

speedy/RINGO cell cycle regulator family member E5	SPDYE5	0,91
NUF2, NDC80 kinetochore complex component	NUF2	0,91
vanin 3	VNN3	0,91
phosphodiesterase 7B	PDE7B	0,91
zinc finger CCCH-type domain-containing-like	LOC441155	0,91
myotubularin related protein 3	MTMR3	0,91
hCG1657980; Transcript Identified by AceView, Entrez Gene ID(s) 200726, RefSeq ID(s) NM_001102659; novel transcript	LOC200726; AC010731.4	0,91
xylosyltransferase I	XYLT1	0,91
FCH domain only 1	FCHO1	0,91
olfactory receptor, family 51, subfamily B, member 6	OR51B6	0,91
interferon induced transmembrane protein 10	IFITM10	0,91
C-type lectin domain family 11, member A	CLEC11A	0,91
Salzman2013 ALT_ACCEPTOR, ALT_DONOR, coding, INTERNAL, intronic best transcript NM_015092	SMG1	0,91
ribonucleoprotein, PTB-binding 1	RAVER1	0,91
FERM domain containing 3	FRMD3	0,91
CCR4-NOT transcription complex subunit 1; small nucleolar RNA, H/ACA box 50A	CNOT1; SNORA50A	0,91
zinc finger protein 225	ZNF225	0,91
retinol dehydrogenase 10 (all-trans)	RDH10	0,91
alcohol dehydrogenase 4 (class II), pi polypeptide	ADH4	0,91
transmembrane protein 18	TMEM18	0,91
nucleoporin 58kDa	NUP58	0,91
intercellular adhesion molecule 4 (Landsteiner-Wiener blood group)	ICAM4	0,91
neuronal tyrosine-phosphorylated phosphoinositide-3-kinase adaptor 1	NYAP1	0,91
nucleotide binding protein 1	NUBP1	0,91
NLR family, pyrin domain containing 4	NLRP4	0,91
chondroitin sulfate synthase 1	CHSY1	0,91
yrdC N(6)-threonylcarbamoyltransferase domain containing	YRDC	0,91
SLIT and NTRK-like family, member 4	SLITRK4	0,91
fibroblast growth factor 8 (androgen-induced)	FGF8	0,91
NIMA-related kinase 6	NEK6	0,91
cilia and flagella associated protein 43	CFAP43	0,91
neuritin 1	NRN1	0,91
DEAD (Asp-Glu-Ala-Asp) box helicase 17	DDX17	0,91
calcium homeostasis modulator 2	CALHM2	0,91
neurensin 1	NRSN1	0,91
HYDIN, axonemal central pair apparatus protein	HYDIN	0,91
zinc finger protein 45	ZNF45	0,91
chromosome 17 open reading frame 47	C17orf47	0,91
membrane-spanning 4-domains, subfamily A, member 7	MS4A7	0,91
beta-1,4-N-acetyl-galactosaminyl transferase 3	B4GALNT3	0,91

S-phase kinase-associated protein 2, E3 ubiquitin protein ligase	SKP2	0,91
autophagy related 2B	ATG2B	0,91
STEAP family member 2, metalloreductase	STEAP2	0,91
transmembrane protein 42	TMEM42	0,91
galanin receptor 1	GALR1	0,91
anoctamin 3	ANO3	0,91
zinc finger protein 853	ZNF853	0,91
ES cell expressed Ras	ERAS	0,91
ATP/GTP binding protein-like 3	AGBL3	0,91
zinc finger, MYM-type 5	ZMYM5	0,91
proline and serine rich 3	PROSER3	0,91
chromosome 9 open reading frame 47; sphingosine-1-phosphate receptor 3	C9orf47; S1PR3	0,91
sorting nexin 14	SNX14	0,91
nucleotide-binding oligomerization domain containing 2	NOD2	0,91
E2F transcription factor 6	E2F6	0,91
SEH1-like nucleoporin	SEH1L	0,91
NFKB inhibitor interacting Ras-like 2	NKIRAS2	0,91
PAX3 and PAX7 binding protein 1	PAXBP1	0,91
phosphatidylinositol-4-phosphate 5-kinase-like 1	PIP5KL1	0,91
eukaryotic elongation factor, selenocysteine-tRNA-specific	EEFSEC	0,91
phenylalanyl-tRNA synthetase alpha subunit	FARSA	0,91
myoferlin	MYOF	0,91
Memczak2013 ANTISENSE, coding, INTERNAL, intronic best transcript NM_020235	BBX	0,91
prostate androgen-regulated mucin-like protein 1	PARM1	0,91
IQ motif and ubiquitin domain containing	IQUB	0,91
secreted frizzled-related protein 2	SFRP2	0,91
myelin protein zero	MPZ	0,91
peripheral myelin protein 2	PMP2	0,91
neuropeptide Y	NPY	0,91
MOB kinase activator 2	MOB2	0,91
CTD small phosphatase 1	CTDSP1	0,91
geranylgeranyl diphosphate synthase 1	GGPS1	0,91
family with sequence similarity 150, member B	FAM150B	0,91
AU RNA binding protein/enoyl-CoA hydratase	AUH	0,91
glyoxylate reductase/hydroxypyruvate reductase	GRHPR	0,91
ubiquitin specific peptidase 29	USP29	0,91
microtubule associated serine/threonine kinase 3	MAST3	0,91
double homeobox A	DUXA	0,91
OTU domain containing 6B	OTUD6B	0,91
CD8b molecule	CD8B	0,91
sterile alpha motif domain containing 8	SAMD8	0,91
kinesin family member 23	KIF23	0,91
vesicle associated membrane protein 3	VAMP3	0,91

Eukaryotic translation initiation factor 3 subunit F [Source:UniProtKB/Swiss-Prot;Acc:O00303]	EIF3F	0,91
tumor necrosis factor, alpha-induced protein 8-like 1	TNFAIP8L1	0,91
hes-related family bHLH transcription factor with YRPW motif-like	HEYL	0,91
exosome component 3	EXOSC3	0,91
solute carrier family 35, member F3	SLC35F3	0,91
misshapen-like kinase 1	MINK1	0,91
THUMP domain containing 1	THUMPD1	0,91
	LRIG1	0,91
tetratricopeptide repeat domain 39B	TTC39B	0,91
tubby like protein 3	TULP3	0,91
CD109 molecule	CD109	0,91
NOP58 ribonucleoprotein	NOP58	0,91
tetratricopeptide repeat domain 5	TTC5	0,91
EPH receptor A5	EPHA5	0,91
ATPase, H+ transporting, lysosomal 13kDa, V1 subunit G1	ATP6V1G1	0,91
iron responsive element binding protein 2	IREB2	0,91
angiogenic factor with G-patch and FHA domains 1	AGGF1	0,91
solute carrier family 25 (mitochondrial carrier; ornithine transporter) member 2	SLC25A2	0,91
phorbol-12-myristate-13-acetate-induced protein 1	PMAIP1	0,91
uncharacterized LOC340074; Transcript Identified by AceView, Entrez Gene ID(s) 340074, RefSeq ID(s) NR_027127; novel transcript	LOC340074; CTC-321K16.4	0,91
ubiquitin specific peptidase 50	USP50	0,91
sialic acid binding Ig-like lectin 6	SIGLEC6	0,91
zinc finger protein 429	ZNF429	0,91
serpin peptidase inhibitor, clade A (alpha-1 antiproteinase, antitrypsin), member 12	SERPINA12	0,91
ankyrin repeat and kinase domain containing 1	ANKK1	0,91
cytochrome c oxidase subunit VIIa polypeptide 2 (liver)	COX7A2	0,91
Transcript Identified by AceView, Entrez Gene ID(s) 23126	POGZ	0,91
zinc finger, DHHC-type containing 18	ZDHHC18	0,91
odorant binding protein 2A	OBP2A	0,91
unc-13 homolog D (C. elegans)	UNC13D	0,91
homeobox B9	HOXB9	0,91
defensin, beta 4A	DEFB4A	0,91
interferon, beta 1, fibroblast	IFNB1	0,91
olfactory receptor, family 10, subfamily R, member 2	OR10R2	0,91
interferon induced transmembrane protein 2	IFITM2	0,91
G protein-coupled receptor 132	GPR132	0,91
YY1 transcription factor; microRNA 6764	YY1; MIR6764	0,91
Memczak2013 ANTISENSE, CDS, coding, INTERNAL best transcript NM_022118	RBM26	0,91

autophagy related 16-like 1	ATG16L1	0,91
Ras-related GTP binding A	RRAGA	0,91
testis expressed 35	TEX35	0,91
chromosome 5 open reading frame 38	C5orf38	0,91
protamine 1	PRM1	0,91
LCK proto-oncogene, Src family tyrosine kinase	LCK	0,91
keratin associated protein 7-1 (gene/pseudogene)	KRTAP7-1	0,91
T-box 3	TBX3	0,91
OTU deubiquitinase, ubiquitin aldehyde binding 1	OTUB1	0,91
MACRO domain containing 2; ribosomal protein S3 pseudogene 1	MACROD2; RPS3P1	0,91
t-complex 11 family, X-linked 2	TCP11X2	0,91
growth differentiation factor 15	GDF15	0,91
Salzman2013 ANNOTATED, CDS, coding, OVCODE, OVEXON, UTR3 best transcript NM_000947	PRIM2	0,91
SET and MYND domain containing 4	SMYD4	0,91
FOS-like antigen 1	FOSL1	0,91
abhydrolase domain containing 17A	ABHD17A	0,91
beta-1,4-N-acetyl-galactosaminyl transferase 2	B4GALNT2	0,91
neuronal differentiation 1	NEUROD1	0,91
DLC1 Rho GTPase activating protein	DLC1	0,91
KDEL (Lys-Asp-Glu-Leu) endoplasmic reticulum protein retention receptor 2	KDELR2	0,91
Transcript Identified by AceView, Entrez Gene ID(s) 51752	ERAP1	0,91
GRIP and coiled-coil domain containing 1	GCC1	0,91
solute carrier family 10, member 7	SLC10A7	0,91
cyclin-dependent kinase-like 2 (CDC2-related kinase)	CDKL2	0,91
adhesion G protein-coupled receptor F5	ADGRF5	0,91
cyclin E1	CCNE1	0,91
progestin and adipoQ receptor family member IV	PAQR4	0,91
sorting nexin family member 27	SNX27	0,91
NADH dehydrogenase (ubiquinone) 1 beta subcomplex, 6, 17kDa	NDUFB6	0,91
GINS complex subunit 3 (Psf3 homolog)	GINS3	0,91
BCL6 corepressor	BCOR	0,91
glutamate decarboxylase 1	GAD1	0,91
mitogen-activated protein kinase kinase kinase 14	MAP3K14	0,91
REX4 homolog, 3'-5' exonuclease [Source:HGNC Symbol;Acc:HGNC:12820]	REXO4	0,91
enolase 2 (gamma, neuronal)	ENO2	0,91
scaffold attachment factor B2	SAFB2	0,91
cysteine conjugate-beta lyase 2; RNA binding motif protein, X-linked-like 1	CCBL2; RBMXL1	0,91
solute carrier family 7, member 14	SLC7A14	0,91
sidekick cell adhesion molecule 1	SDK1	0,91
crystallin alpha A	CRYAA	0,91

perforin 1 (pore forming protein)	PRF1	0,91
olfactory receptor, family 52, subfamily I, member 1	OR52I1	0,91
leucine-rich repeat kinase 2	LRRK2	0,91
AP2 associated kinase 1	AAK1	0,91
exocyst complex component 4	EXOC4	0,91
histone cluster 1, H2ac	HIST1H2AC	0,91
androgen receptor	AR	0,91
collagen triple helix repeat containing 1	CTHRC1	0,91
G protein-coupled receptor kinase 5	GRK5	0,91
chromosome 19 open reading frame 53	C19orf53	0,91
mal, T-cell differentiation protein	MAL	0,91
solute carrier family 22, member 31	SLC22A31	0,91
protein phosphatase 2C-like domain containing 1	PP2D1	0,91
histocompatibility (minor) HA-1	HMHA1	0,91
UEV and lactate/malate dehyrogenase domains	UEVLD	0,91
nudix hydrolase 2	NUDT2	0,91
Rho GTPase activating protein 19	ARHGAP19	0,91
small ubiquitin-like modifier 3	SUMO3	0,91
Memczak2013 ALT_ACCEPTOR, ALT_DONOR, coding, INTERNAL, intronic best transcript NM_001142333	RBFOX1	0,91
G-patch domain containing 8	GPATCH8	0,91
Jeck2013 ALT_ACCEPTOR, ALT_DONOR, coding, INTERNAL, intronic best transcript NM_006042	HS3ST3A1	0,91
glycerol-3-phosphate dehydrogenase 1	GPD1	0,91
FLYWCH family member 2	FLYWCH2	0,91
transcription factor 24	TCF24	0,91
MAP kinase interacting serine/threonine kinase 2	MKNK2	0,91
testis specific protein, Y-linked 1	TSPY1	0,91
prolactin releasing hormone receptor	PRLHR	0,91
hypocretin (orexin) receptor 1	HCRTR1	0,91
Memczak2013 ANTISENSE, CDS, coding, INTERNAL best transcript NM_004719	SCAF11	0,91
crystallin zeta	CRYZ	0,91
centromere protein T	CENPT	0,91
paired immunoglobin-like type 2 receptor beta; stromal antigen 3-like 5 pseudogene; poliovirus receptor related immunoglobulin domain containing 2, pseudogene; microRNA 6840; STAG3L5P-PVRIG2P-PILRB readthrough	PILRB; STAG3L5P; PVRIG2P; MIR6840; STAG3L5P-PVRIG2P-PILRB	0,91
programmed cell death 10	PDCD10	0,91
translocase of outer mitochondrial membrane 40 homolog (yeast)	TOMM40	0,91
BLK proto-oncogene, Src family tyrosine kinase	BLK	0,91
protease, serine, 56	PRSS56	0,91

DENN/MADD domain containing 6A	DENND6A	0,91
HAUS augmin like complex subunit 5	HAUS5	0,91
DnaJ (Hsp40) homolog, subfamily C, member 12	DNAJC12	0,91
kinesin light chain 3	KLC3	0,91
synaptonemal complex central element protein 1; shadow of prion protein homolog (zebrafish) pseudogene 1	SYCE1; SPRNP1	0,91
enkurin domain containing 1	ENKD1	0,91
endonuclease domain containing 1	ENDOD1	0,91
family with sequence similarity 153, member A	FAM153A	0,91
cullin 7	CUL7	0,91
cytochrome P450, family 4, subfamily F, polypeptide 8	CYP4F8	0,91
chromosome 16 open reading frame 97	C16orf97	0,91
DAB2 interacting protein	DAB2IP	0,91
phosphoglucomutase 3	PGM3	0,91
solute carrier family 11 (proton-coupled divalent metal ion transporter), member 2	SLC11A2	0,91
SRC proto-oncogene, non-receptor tyrosine kinase	SRC	0,91
transmembrane protein 253	TMEM253	0,91
chromosome 8 open reading frame 31	C8orf31	0,91
ubiquitin specific peptidase 16	USP16	0,91
THUMPD3 antisense RNA 1	THUMPD3-AS1	0,91
solute carrier family 26 (anion exchanger), member 4	SLC26A4	0,91
glycoprotein hormones, alpha polypeptide	CGA	0,91
serine/arginine repetitive matrix 3	SRRM3	0,91
single-pass membrane protein with coiled-coil domains 1	SMCO1	0,91
death effector domain containing 2	DEDD2	0,91
cap methyltransferase 1	CMTR1	0,91
SEC16 homolog A, endoplasmic reticulum export factor	SEC16A	0,91
tubby like protein 4	TULP4	0,91
transmembrane protein 200A	TMEM200A	0,91
ring finger protein 34, E3 ubiquitin protein ligase	RNF34	0,91
fibrillarin	FBL	0,91
family with sequence similarity 78, member A	FAM78A	0,91
DAZ interacting zinc finger protein 1	DZIP1	0,91
malic enzyme 1, NADP(+)-dependent, cytosolic	ME1	0,91
StAR-related lipid transfer domain containing 7	STARD7	0,91
zinc finger protein 454	ZNF454	0,91
lemur tyrosine kinase 3	LMTK3	0,91
phosphoribosylaminoimidazole carboxylase, phosphoribosylaminoimidazole succinocarboxamide synthetase	PAICS	0,91
lysine (K)-specific demethylase 3B	KDM3B	0,91
solute carrier family 35 (GDP-fucose transporter), member C1	SLC35C1	0,91
chromodomain helicase DNA binding protein 1	CHD1	0,91
cancer/testis antigen 55	CT55	0,91
SUMO1/sentrin specific peptidase 1	SENP1	0,91

frizzled class receptor 5	FZD5	0,91
polymerase (DNA directed), alpha 1, catalytic subunit	POLA1	0,91
histone cluster 3, H3	HIST3H3	0,91
MYCBP-associated, testis expressed 1	MAATS1	0,91
peptidylprolyl isomerase A (cyclophilin A)-like 4C	PPIAL4C	0,91
CCAAT/enhancer binding protein (C/EBP), alpha	CEBPA	0,91
chromosome 9 open reading frame 69	C9orf69	0,91
pleiomorphic adenoma gene-like 2	PLAGL2	0,91
ermin	ERMN	0,91
RNA binding motif protein, Y-linked, family 1, member J; RNA binding motif protein, Y-linked, family 1, member F	RBMY1J; RBMY1F	0,91
hydroxylysine kinase	HYKK	0,91
oxidoreductase NAD-binding domain containing 1	OXNAD1	0,91
zinc finger protein 707	ZNF707	0,91
minichromosome maintenance complex component 5	MCM5	0,91
olfactory receptor, family 2, subfamily C, member 3; GCSAML antisense RNA 1	OR2C3; GCSAML-AS1	0,91
mitochondrial ribosomal protein L19	MRPL19	0,91
histone cluster 1, H2be	HIST1H2BE	0,91
integrin-binding sialoprotein	IBSP	0,91
Transcript Identified by AceView, Entrez Gene ID(s) 7837	PXDN	0,91
integrin beta 5	ITGB5	0,91
SCL/TAL1 interrupting locus	STIL	0,91
cytoskeleton associated protein 2-like	CKAP2L	0,91
neighbor of BRCA1 gene 1	NBR1	0,91
SH3-domain binding protein 1; pyridoxal (pyridoxine, vitamin B6) phosphatase	SH3BP1; PDXP	0,91
kelch domain containing 10	KLHDC10	0,91
KIAA1715	KIAA1715	0,91
ribonuclease, RNase A family, 10 (non-active)	RNASE10	0,91
cyclin-dependent kinase 8	CDK8	0,91
protocadherin beta 7	PCDHB7	0,91
testis expressed 2	TEX2	0,91
mitochondrial ribosomal protein L57	MRPL57	0,91
aryl hydrocarbon receptor interacting protein-like 1	AIPL1	0,91
TAF5 RNA polymerase II, TATA box binding protein (TBP)-associated factor, 100kDa	TAF5	0,91
ubiquitin specific peptidase 40	USP40	0,91
solute carrier family 5 (sodium/monocarboxylate cotransporter), member 8	SLC5A8	0,91
choline kinase alpha	CHKA	0,91
prolyl 4-hydroxylase, alpha polypeptide II	P4HA2	0,91
golgi glycoprotein 1	GLG1	0,91
UDP-GlcNAc:betaGal beta-1,3-N-acetylglucosaminyltransferase 2	B3GNT2	0,91

FKBP1A-SDCBP2 readthrough (NMD candidate)	FKBP1A-SDCBP2	0,91
neuropeptide FF receptor 2	NPFFR2	0,91
TLC domain containing 1	TLCD1	0,91
RNA binding motif protein, X-linked 2	RBMX2	0,91
5-nucleotidase, cytosolic II	NT5C2	0,91
zinc finger protein 705A; family with sequence similarity 66, member C	ZNF705A; FAM66C	0,91
potassium channel, voltage gated shaker related subfamily A, member 10	KCNA10	0,91
zinc finger protein 600	ZNF600	0,91
zinc finger protein 560	ZNF560	0,91
feline leukemia virus subgroup C cellular receptor family, member 2	FLVCR2	0,91
MKRN2 opposite strand	MKRN2OS	0,91
olfactory receptor, family 7, subfamily G, member 2	OR7G2	0,91
long intergenic non-protein coding RNA 494	LINC00494	0,91
gap junction protein beta 4	GJB4	0,91
cholinergic receptor, nicotinic delta	CHRND	0,91
immunoglobulin superfamily, member 9	IGSF9	0,91
nuclear protein, ataxia-telangiectasia locus	NPAT	0,91
colony stimulating factor 2 receptor, alpha, low-affinity (granulocyte-macrophage)	CSF2RA	0,91
storkhead box 2	STOX2	0,91
protein kinase D3	PRKD3	0,91
BRX1, biogenesis of ribosomes	BRIX1	0,91
uncharacterized LOC101929372; novel transcript	LOC101929372; AC004019.13	0,91
vav 3 guanine nucleotide exchange factor	VAV3	0,91
MAGE family member A12; CSAG family, member 4 (pseudogene)	MAGEA12; CSAG4	0,91
centromere protein P	CENPP	0,91
zinc finger protein 202	ZNF202	0,91
BCL2-associated athanogene 5	BAG5	0,91
solute carrier family 35 (adenosine 3-phospho 5-phosphosulfate transporter), member B3	SLC35B3	0,91
cystatin F (leukocystatin)	CST7	0,91
fibroblast growth factor 19	FGF19	0,91
IQ motif containing F3	IQCF3	0,91
forty-two-three domain containing 1	FYTTD1	0,91
zinc finger CCCH-type containing 6	ZC3H6	0,91
DEAH (Asp-Glu-Ala-His) box polypeptide 37	DHX37	0,91
basic helix-loop-helix family, member e22	BHLHE22	0,91
dysbindin (dystrobrevin binding protein 1) domain containing 1	DBNDD1	0,91

methylmalonic aciduria (cobalamin deficiency) cblC type, with homocystinuria	MMACHC	0,91
olfactory receptor, family 10, subfamily H, member 5	OR10H5	0,91
MAGE family member L2	MAGEL2	0,91
PR domain containing 7	PRDM7	0,91
breast cancer 2, early onset	BRCA2	0,91
TANK-binding kinase 1	TBK1	0,91
olfactory receptor, family 1, subfamily C, member 1	OR1C1	0,91
autophagy related 16-like 1	ATG16L1	0,91
hypoxia up-regulated 1	HYOU1	0,91
N-acetylglucosamine kinase	NAGK	0,91
CD93 molecule	CD93	0,91
potassium channel, voltage gated subfamily A regulatory beta subunit 1	KCNAB1	0,91
ephrin-A2	EFNA2	0,91
cyclin and CBS domain divalent metal cation transport mediator 3	CNNM3	0,91
zinc finger protein 502	ZNF502	0,91
ribonuclease P/MRP 21kDa subunit	RPP21	0,91
neuropeptide FF receptor 1	NPFFR1	0,91
UV stimulated scaffold protein A	UVSSA	0,91
cytoplasmic FMR1 interacting protein 2	CYFIP2	0,91
solute carrier family 35, member A4	SLC35A4	0,91
olfactory receptor, family 11, subfamily G, member 2	OR11G2	0,91
tripartite motif containing 13; potassium channel regulator	TRIM13; KCNRG	0,91
complement component 1, q subcomponent, C chain	C1QC	0,91
Fanconi anemia complementation group C	FANCC	0,91
CD44 molecule (Indian blood group)	CD44	0,91
immunity-related GTPase family, Q	IRGQ	0,91
glucagon	GCG	0,91
hyaluronan and proteoglycan link protein 3	HAPLN3	0,91
mitogen-activated protein kinase 7	MAPK7	0,91
H2A histone family, member Z	H2AFZ	0,91
ADP-ribosylation factor like GTPase 14 effector protein	ARL14EP	0,91
UDP-Gal:betaGlcNAc beta 1,4- galactosyltransferase, polypeptide 3	B4GALT3	0,91
protein kinase C and casein kinase substrate in neurons 3	PACSIN3	0,91
ring finger protein 170; microRNA 4469	RNF170; MIR4469	0,91
tripartite motif containing 8	TRIM8	0,91
phosphoglycolate phosphatase	PGP	0,91
olfactory receptor, family 2, subfamily K, member 2	OR2K2	0,91
aurora kinase A and ninein interacting protein	AUNIP	0,91
DEAH (Asp-Glu-Ala-His) box polypeptide 8	DHX8	0,91

Myb/SANT-like DNA-binding domain containing 4 with coiled-coils	MSANTD4	0,91
heparin-binding EGF-like growth factor	HBEGF	0,91
caspase recruitment domain family, member 10	CARD10	0,91
TAF2 RNA polymerase II, TATA box binding protein (TBP)-associated factor, 150kDa	TAF2	0,91
filamin A interacting protein 1	FILIP1	0,91
pseudouridine 5-phosphatase	PUDP	0,91
Memczak2013 ALT_ACCEPTOR, ALT_DONOR, coding, INTERNAL, intronic best transcript NM_001099272	BTBD9	0,91
ets variant 3-like	ETV3L	0,91
Transcript Identified by AceView, Entrez Gene ID(s) 7082	TJP1	0,91
HMG-box transcription factor 1	HBP1	0,91
phosphatidylinositol-4-phosphate 5-kinase, type I, alpha	PIP5K1A	0,91
family with sequence similarity 184, member A	FAM184A	0,91
RPTOR independent companion of MTOR, complex 2	RICTOR	0,91
sulfatase modifying factor 1	SUMF1	0,91
peroxisomal biogenesis factor 1	PEX1	0,91
piccolo presynaptic cytomatrix protein	PCLO	0,91
cadherin-like and PC-esterase domain containing 1	CPED1	0,90
ceramide-1-phosphate transfer protein	CPTP	0,90
FLYWCH-type zinc finger 1	FLYWCH1	0,90
coiled-coil domain containing 138	CCDC138	0,90
nudix hydrolase 11	NUDT11	0,90
v-maf avian musculoaponeurotic fibrosarcoma oncogene homolog K	MAFK	0,90
potassium voltage-gated channel, modifier subfamily S, member 2	KCNS2	0,90
intelectin 2	ITLN2	0,90
Rap guanine nucleotide exchange factor like 1	RAPGEFL1	0,90
WD repeat domain 89	WDR89	0,90
kininogen 1	KNG1	0,90
cat eye syndrome chromosome region, candidate 2	CECR2	0,90
5-hydroxytryptamine (serotonin) receptor 1A, G protein-coupled	HTR1A	0,90
annexin A4	ANXA4	0,90
oxytocin/neurophysin I prepropeptide	OXT	0,90
sushi domain containing 2	SUSD2	0,90
Rho guanine nucleotide exchange factor 33	ARHGEF33	0,90
prostaglandin E receptor 4 (subtype EP4)	PTGER4	0,90
transglutaminase 4	TGM4	0,90
spermatogenesis associated 7	SPATA7	0,90
zinc finger protein 418	ZNF418	0,90
chromosome 3 open reading frame 58	C3orf58	0,90
leucine rich repeat containing 15	LRRC15	0,90

leukocyte immunoglobulin-like receptor, subfamily A (with TM domain), member 5	LILRA5	0,90
thymus, brain and testes associated	TBATA	0,90
B-cell CLL/lymphoma 11B (zinc finger protein)	BCL11B	0,90
peroxisomal biogenesis factor 6	PEX6	0,90
potassium channel, calcium activated intermediate/small conductance subfamily N alpha, member 2	KCNN2	0,90
protein kinase domain containing, cytoplasmic	PKDCC	0,90
ephrin-B2	EFNB2	0,90
DNA damage-induced apoptosis suppressor	DDIAS	0,90
procollagen-lysine, 2-oxoglutarate 5-dioxygenase 1	PLOD1	0,90
sorting nexin 11	SNX11	0,90
exosome component 9	EXOSC9	0,90
male-specific lethal 1 homolog (Drosophila)	MSL1	0,90
nuclear receptor subfamily 2, group F, member 6	NR2F6	0,90
exonuclease 3-5 domain containing 2	EXD2	0,90
zinc finger protein 792	ZNF792	0,90
translocase of inner mitochondrial membrane 10 homolog B (yeast)	TIMM10B	0,90
Cbp/p300-interacting transactivator, with Glu/Asp rich carboxy-terminal domain, 2	CITED2	0,90
kelch-like family member 35	KLHL35	0,90
hydroxysteroid (17-beta) dehydrogenase 11	HSD17B11	0,90
pygopus family PHD finger 2	PYGO2	0,90
adaptor-related protein complex 1, mu 2 subunit	AP1M2	0,90
negative regulator of reactive oxygen species	NRROS	0,90
keratin 27, type I	KRT27	0,90
B-Raf proto-oncogene, serine/threonine kinase	BRAF	0,90
ankyrin repeat domain 9	ANKRD9	0,90
IMP (inosine 5-monophosphate) dehydrogenase 1	IMPDH1	0,90
leucine-rich alpha-2-glycoprotein 1	LRG1	0,90
C7orf55-LUC7L2 readthrough; LUC7-like 2 pre-mRNA splicing factor; chromosome 7 open reading frame 55	C7orf55-LUC7L2; LUC7L2; C7orf55	0,90
ubiquitin specific peptidase 36	USP36	0,90
opioid growth factor receptor	OGFR	0,90
ubiquitin specific peptidase 31	USP31	0,90
transcription factor 7-like 2 (T-cell specific, HMG-box)	TCF7L2	0,90
anoctamin 9	ANO9	0,90
SRY box 11	SOX11	0,90
sialic acid binding Ig-like lectin 8	SIGLEC8	0,90
thiamin pyrophosphokinase 1	TPK1	0,90
LanC lantibiotic synthetase component C-like 1 (bacterial)	LANCL1	0,90
PSMC3 interacting protein	PSMC3IP	0,90

epithelial stromal interaction 1 (breast)	EPSTI1	0,90
major histocompatibility complex, class II, DO alpha	HLA-DOA	0,90
H1 histone family, member X	H1FX	0,90
cytochrome P450, family 2, subfamily A, polypeptide 13	CYP2A13	0,90
bromodomain, testis-specific	BRDT	0,90
GIPC PDZ domain containing family, member 1	GIPC1	0,90
carbohydrate (chondroitin 4) sulfotransferase 13	CHST13	0,90
NPL4 homolog, ubiquitin recognition factor	NPLOC4	0,90
solute carrier family 10, member 5	SLC10A5	0,90
SPEG complex locus	SPEG	0,90
Transcript Identified by AceView, Entrez Gene ID(s) 10777	ARPP21	0,90
ribonuclease, RNase A family, 1 (pancreatic)	RNASE1	0,90
protein phosphatase 2, regulatory subunit B, beta	PPP2R2B	0,90
NADH dehydrogenase (ubiquinone) 1 alpha subcomplex, 10, 42kDa	NDUFA10	0,90
transthyretin	TTR	0,90
bromodomain adjacent to zinc finger domain 2A	BAZ2A	0,90
transmembrane p24 trafficking protein 2	TMED2	0,90
NSL1, MIS12 kinetochore complex component	NSL1	0,90
kinetochore associated 1	KNTC1	0,90
solute carrier family 44 (choline transporter), member 1	SLC44A1	0,90
family with sequence similarity 215, member A (non-protein coding)	FAM215A	0,90
abhydrolase domain containing 13	ABHD13	0,90
ectonucleoside triphosphate diphosphohydrolase 2	ENTPD2	0,90
villin-like	VILL	0,90
ADP-ribosyltransferase 1	ART1	0,90
Rho guanine nucleotide exchange factor 4	ARHGEF4	0,90
nuclear protein 2, transcriptional regulator	NUPR2	0,90
trafficking protein particle complex 4	TRAPPC4	0,90
NUS1 dehydrodolichyl diphosphate synthase subunit	NUS1	0,90
HFM1, ATP-dependent DNA helicase homolog	HFM1	0,90
coiled-coil domain containing 150	CCDC150	0,90
uroplakin 3B-like; polymerase (RNA) II (DNA directed) polypeptide J2	UPK3BL; POLR2J2	0,90
methionyl aminopeptidase 1	METAP1	0,90
PX domain containing 1	PXDC1	0,90
calcium binding and coiled-coil domain 1	CALCOCO1	0,90
proline rich 5 like	PRR5L	0,90
protein inhibitor of activated STAT 4	PIAS4	0,90
GRIP and coiled-coil domain containing 1	GCC1	0,90
ATPase, H+ transporting, lysosomal 42kDa, V1 subunit C1	ATP6V1C1	0,90
solute carrier family 27 (fatty acid transporter), member 6	SLC27A6	0,90
protein kinase D2	PRKD2	0,90
tetratricopeptide repeat domain 7B	TTC7B	0,90

hepatoma-derived growth factor	HDGF	0,90
DCC netrin 1 receptor	DCC	0,90
Memczak2013 ANTISENSE, coding, INTERNAL, intronic best transcript NM_006721	ADK	0,90
oligodendrocyte lineage transcription factor 2	OLIG2	0,90
troponin I type 1 (skeletal, slow)	TNNI1	0,90
NME/NM23 nucleoside diphosphate kinase 3	NME3	0,90
immunoglobulin superfamily, DCC subclass, member 3	IGDCC3	0,90
calcium binding protein 2	CABP2	0,90
ring finger protein 39	RNF39	0,90
Transcript Identified by AceView, Entrez Gene ID(s) 50515	CHST11	0,90
growth factor independent 1B transcription repressor	GFI1B	0,90
metallophosphoesterase 1	MPPE1	0,90
Transcript Identified by AceView, Entrez Gene ID(s) 10777	ARPP21	0,90
lectin, galactoside-binding, soluble, 1	LGALS1	0,90
TRAF family member-associated NFKB activator	TANK	0,90
iduronate 2-sulfatase	IDS	0,90
Zhang2013 ALT_ACCEPTOR, ALT_DONOR, coding, INTERNAL, intronic best transcript NM_173595	ANKRD52	0,90
polycystin (PKD) family receptor for egg jelly	PKDREJ	0,90
tripartite motif containing 21	TRIM21	0,90
PDZ domain containing 1	PDZK1	0,90
ankyrin repeat and death domain containing 1B	ANKDD1B	0,90
spectrin repeat containing, nuclear envelope 1	SYNE1	0,90
transmembrane protein 139	TMEM139	0,90
cerebellar degeneration related protein 1	CDR1	0,90
ER membrane protein complex subunit 10	EMC10	0,90
histone cluster 1, H2ah	HIST1H2AH	0,90
retinoblastoma binding protein 8; microRNA 4741	RBBP8; MIR4741	0,90
microfibrillar associated protein 1	MFAP1	0,90
ribosomal protein S6 kinase, 90kDa, polypeptide 1	RPS6KA1	0,90
cytochrome c, somatic	CYCS	0,90
C1GALT1-specific chaperone 1 like	C1GALT1C1L	0,90
vaccinia related kinase 1	VRK1	0,90
lectin, galactoside-binding, soluble, 3 binding protein	LGALS3BP	0,90
myotubularin related protein 12	MTMR12	0,90
carnitine O-acetyltransferase	CRAT	0,90
WD repeat domain 55	WDR55	0,90
Transcript Identified by AceView, Entrez Gene ID(s) 108	ADCY2	0,90
ATPase family, AAA domain containing 5	ATAD5	0,90
eukaryotic translation initiation factor 4A1; small nucleolar RNA, C/D box 10; small nucleolar RNA, H/ACA box 67; small nucleolar RNA, H/ACA box 48	EIF4A1; SNORD10; SNORA67; SNORA48	0,90

mitochondrial trans-2-enoyl-CoA reductase	MECR	0,90
noncompact myelin associated protein	NCMAP	0,90
trafficking protein particle complex 4	TRAPPC4	0,90
ribonuclease H2, subunit B	RNASEH2B	0,90
free fatty acid receptor 3	FFAR3	0,90
collagen, type XX, alpha 1	COL20A1	0,90
cytochrome c oxidase subunit I	COX1	0,90
protein kinase, X-linked	PRKX	0,90
high mobility group box 4	HMGB4	0,90
RNA polymerase II associated protein 3	RPAP3	0,90
histone cluster 1, H2ae	HIST1H2AE	0,90
actin, beta-like 2	ACTBL2	0,90
Memczak2013 ANTISENSE, coding, INTERNAL, UTR3 best transcript NM_001001349	NKIRAS2	0,90
serologically defined colon cancer antigen 3	SDCCAG3	0,90
KDEL (Lys-Asp-Glu-Leu) containing 1	KDELC1	0,90
LYR motif containing 7	LYRM7	0,90
MYCBP associated protein	MYCBPAP	0,90
keratin associated protein 9-8	KRTAP9-8	0,90
sperm flagellar 1	SPEF1	0,90
COMM domain containing 4	COMMD4	0,90
endoplasmic reticulum lectin 1	ERLEC1	0,90
ZFP28 zinc finger protein	ZFP28	0,90
trafficking protein, kinesin binding 1	TRAK1	0,90
sorting nexin family member 21	SNX21	0,90
phosphofructokinase, liver	PFKL	0,90
Jeck2013 ANTISENSE, coding, INTERNAL, intronic best transcript NM_001103184	FMN1	0,90
lipoma HMGIC fusion partner-like 3	LHFPL3	0,90
protein phosphatase 1, regulatory subunit 13 like	PPP1R13L	0,90
doublecortin domain containing 2	DCDC2	0,90
transcription factor 20 (AR1)	TCF20	0,90
transcription factor 20 (AR1)	TCF20	0,90
chromosome X open reading frame 40A	CXorf40A	0,90
protein tyrosine phosphatase, receptor type, F	PTPRF	0,90
EF-hand domain family, member B	EFHB	0,90
proteasomal ATPase-associated factor 1	PAAF1	0,90
izumo sperm-egg fusion 1	IZUMO1	0,90
Rac/Cdc42 guanine nucleotide exchange factor 6	ARHGEF6	0,90
alpha-2-glycoprotein 1, zinc-binding	AZGP1	0,90
K(lysine) acetyltransferase 8	KAT8	0,90
minichromosome maintenance complex component 4	MCM4	0,90
H2B histone family, member M	H2BFM	0,90
transmembrane protein 223	TMEM223	0,90
KIAA1324-like	KIAA1324L	0,90

chaperonin containing TCP1, subunit 8 (theta)	CCT8	0,90
phosphatidylinositol glycan anchor biosynthesis class X	PIGX	0,90
peroxisomal biogenesis factor 26	PEX26	0,90
NADH dehydrogenase (ubiquinone) flavoprotein 2, 24kDa	NDUFV2	0,90
transmembrane protein 167A	TMEM167A	0,90
phosphatidylinositol glycan anchor biosynthesis class K	PIGK	0,90
SUN domain containing ossification factor	SUCO	0,90
kelch-like family member 31	KLHL31	0,90
meiosis-specific nuclear structural 1	MNS1	0,90
lysophosphatidylcholine acyltransferase 1	LPCAT1	0,90
synaptic Ras GTPase activating protein 1; microRNA 5004	SYNGAP1; MIR5004	0,90
chromosome 7 open reading frame 50	C7orf50	0,90
Dexi homolog (mouse)	DEXI	0,90
phospholipase C, gamma 2 (phosphatidylinositol-specific)	PLCG2	0,90
RecQ mediated genome instability 1	RMI1	0,90
dermcidin	DCD	0,90
POU class 3 homeobox 4	POU3F4	0,90
potassium channel, inwardly rectifying subfamily J, member 16	KCNJ16	0,90
chromosome 17 open reading frame 77	C17orf77	0,90
protein tyrosine phosphatase, mitochondrial 1	PTPMT1	0,90
serine/arginine-rich splicing factor 1	SRSF1	0,90
Jeck2013 ALT_ACCEPTOR, ALT_DONOR, coding, INTERNAL, intronic best transcript NM_006734	HIVEP2	0,90
sodium channel, voltage gated, type IX alpha subunit	SCN9A	0,90
testis expressed 11	TEX11	0,90
solute carrier family 38, member 11	SLC38A11	0,90
GTP cyclohydrolase 1	GCH1	0,90
kinesin family member 5C	KIF5C	0,90
NSE2/MMS21 homolog, SMC5-SMC6 complex SUMO ligase	NSMCE2	0,90
cardiotrophin-like cytokine factor 1	CLCF1	0,90
pre-mRNA processing factor 31	PRPF31	0,90
neuroblastoma breakpoint family, member 8	NBPF8	0,90
PARK2 co-regulated	PACRG	0,90
alcohol dehydrogenase 5 (class III), chi polypeptide	ADH5	0,90
tripartite motif containing 56	TRIM56	0,90
Transcript Identified by AceView, Entrez Gene ID(s) 4646	MYO6	0,90
zinc finger protein 3	ZNF3	0,90
immediate early response 5	IER5	0,90
SOS Ras/Rac guanine nucleotide exchange factor 1	SOS1	0,90
CWF19-like 2, cell cycle control (S. pombe)	CWF19L2	0,90
tetratricopeptide repeat domain 38	TTC38	0,90
ets variant 1	ETV1	0,90
glycoprotein V (platelet)	GP5	0,90
long intergenic non-protein coding RNA 174	LINC00174	0,90

dual specificity phosphatase 18	DUSP18	0,90
regulator of G-protein signaling 6	RGS6	0,90
MAX dimerization protein 1	MXD1	0,90
Memczak2013 ALT_ACCEPTOR, ALT_DONOR, coding, INTERNAL, intronic best transcript NM_001190821	SMAD7	0,90
inositol 1,3,4,5,6-pentakisphosphate 2-kinase	IPPK	0,90
RAB guanine nucleotide exchange factor (GEF) 1	RABGEF1	0,90
hydroxymethylbilane synthase	HMBS	0,90
mitogen-activated protein kinase kinase kinase 19	MAP3K19	0,90
small integral membrane protein 1 (Vel blood group)	SMIM1	0,90
ST8 alpha-N-acetyl-neuraminide alpha-2,8-sialyltransferase 1	ST8SIA1	0,90
Zhang2013 ALT_ACCEPTOR, ALT_DONOR, coding, INTERNAL, intronic best transcript NM_182706	SCRIB	0,90
amnion associated transmembrane protein	AMN	0,90
PAS domain containing 1	PASD1	0,90
dihydropyrimidinase-like 2	DPYSL2	0,90
oral cancer overexpressed 1	ORAOV1	0,90
family with sequence similarity 169, member B	FAM169B	0,90
inhibitor of kappa light polypeptide gene enhancer in B-cells, kinase epsilon	IKBKE	0,90
docking protein 1	DOK1	0,90
prepronociceptin	PNOC	0,90
echinoderm microtubule associated protein like 3	EML3	0,90
WD repeat domain 24	WDR24	0,90
casein kinase 1, alpha 1	CSNK1A1	0,90
BCL2-like 13 (apoptosis facilitator)	BCL2L13	0,90
solute carrier family 27 (fatty acid transporter), member 3	SLC27A3	0,90
Homo sapiens uncharacterized LOC100133669 (LOC100133669), long non-coding RNA.; Transcript Identified by AceView, Entrez Gene ID(s) 100133669; novel transcript	LOC100133669; RP11-273G15.2	0,90
ADP-ribosylation factor like GTPase 14	ARL14	0,90
chromosome 11 open reading frame 65	C11orf65	0,90
myogenic factor 6 (herculin)	MYF6	0,90
fat storage-inducing transmembrane protein 1	FITM1	0,90
urotensin 2B	UTS2B	0,90
Memczak2013 ANTISENSE, CDS, coding, INTERNAL best transcript NM_014709	USP34	0,90
histidine triad nucleotide binding protein 1	HINT1	0,90
zinc finger protein 614	ZNF614	0,90
interleukin 1 receptor, type II	IL1R2	0,90
nicastrin	NCSTN	0,90
thymosin beta 15B	TMSB15B	0,90
heat shock 70kDa protein 2	HSPA2	0,90
zinc finger protein 780B	ZNF780B	0,90
zinc finger protein 35	ZNF35	0,90

peroxisomal membrane protein 2	PXMP2	0,90
pseudouridylate synthase-like 1	PUSL1	0,90
proline rich 23 domain containing 2; proline rich 23 domain containing 1	PRR23D2; PRR23D1	0,90
tumor suppressor candidate 5	TUSC5	0,90
solute carrier family 25 (mitochondrial carrier: glutamate), member 22	SLC25A22	0,90
tumor necrosis factor (ligand) superfamily, member 9	TNFSF9	0,90
ATPase, H+ transporting, lysosomal 31kDa, V1 subunit E2	ATP6V1E2	0,90
neuronal PAS domain protein 1	NPAS1	0,90
solute carrier family 34 (type II sodium/phosphate cotransporter), member 1	SLC34A1	0,90
fibroblast growth factor 7	FGF7	0,90
claudin 18	CLDN18	0,90
kinesin family member 5B	KIF5B	0,90
PRAME family member 25	PRAMEF25	0,90
PRAME family member 25	PRAMEF25	0,90
enhancer of zeste 2 polycomb repressive complex 2 subunit	EZH2	0,90
serpin peptidase inhibitor, clade H (heat shock protein 47), member 1, (collagen binding protein 1)	SERPINH1	0,90
cyclin-dependent kinase 2	CDK2	0,90
sialic acid binding Ig-like lectin 7	SIGLEC7	0,90
plasminogen-like B2	PLGLB2	0,90
tweety family member 3	TTYH3	0,90
uncharacterized LOC100505549; Salzman2013 ANNOTATED, CDS, coding, OVCODE, OVERLAPTX, OVEXON, UTR3, UTR5 best transcript NM_001242804; novel transcript, antisense to ATP8B1	LOC100505549; RP11-35G9.3	0,90
autophagy related 16-like 1	ATG16L1	0,90
troponin C type 1 (slow)	TNNC1	0,90
acyl-CoA oxidase 3, pristanoyl	ACOX3	0,90
potassium channel, two pore domain subfamily K, member 1	KCNK1	0,90
tetratricopeptide repeat domain 37	TTC37	0,90
cadherin, EGF LAG seven-pass G-type receptor 2	CELSR2	0,90
hydroxysteroid (17-beta) dehydrogenase 14	HSD17B14	0,90
Transcript Identified by AceView, Entrez Gene ID(s) 58155	PTBP2	0,90
adiponectin, C1Q and collagen domain containing	ADIPOQ	0,90
UDP-Gal:betaGal beta 1,3-galactosyltransferase 6	B3GALT6	0,90
S100 calcium binding protein A7A	S100A7A	0,90
Rho GTPase activating protein 20	ARHGAP20	0,90
family with sequence similarity 177, member A1	FAM177A1	0,90
insulin receptor-related receptor	INSRR	0,90
dimethylarginine dimethylaminohydrolase 1	DDAH1	0,90
transcription elongation factor B polypeptide 3B (elongin A2)	TCEB3B	0,90
KIRREL3 antisense RNA 3	KIRREL3-AS3	0,90

chromosome 3 open reading frame 14	C3orf14	0,90
Rho GTPase activating protein 1	ARHGAP1	0,90
high mobility group 20A	HMG20A	0,90
receptor-interacting serine-threonine kinase 3	RIPK3	0,90
autophagy related 7	ATG7	0,90
glyoxalase I	GLO1	0,90
meiotic double-stranded break formation protein 1	MEI1	0,90
leucine rich repeat containing 19	LRRC19	0,90
solute carrier family 35, member E1	SLC35E1	0,90
myosin IE	MYO1E	0,90
DET1 and DDB1 associated 1	DDA1	0,90
VANGL planar cell polarity protein 1	VANGL1	0,90
chaperonin containing TCP1, subunit 2 (beta)	CCT2	0,90
glycosylated lysosomal membrane protein	GLMP	0,90
wingless-type MMTV integration site family, member 3A	WNT3A	0,90
cytochrome P450, family 51, subfamily A, polypeptide 1	CYP51A1	0,90
sosondowah ankyrin repeat domain family member C	SOWAHC	0,90
paralemmin 3	PALM3	0,90
acyl-CoA dehydrogenase family, member 11	ACAD11	0,90
transmembrane protein 107; small nucleolar RNA, C/D box 118	TMEM107; SNORD118	0,90
orthodenticle homeobox 2	OTX2	0,90
cerebellar degeneration related protein 2	CDR2	0,90
SEC31 homolog A, COPII coat complex component	SEC31A	0,90
uracil DNA glycosylase	UNG	0,90
synaptic vesicle glycoprotein 2A	SV2A	0,90
cereblon	CRBN	0,90
v-akt murine thymoma viral oncogene homolog 2	AKT2	0,90
BCL2-associated athanogene 3	BAG3	0,90
cyclin J	CCNJ	0,90
lymphotoxin beta (TNF superfamily, member 3)	LTB	0,90
heme binding protein 1	HEBP1	0,90
protein phosphatase, Mg2+/Mn2+ dependent, 1K	PPM1K	0,90
transmembrane protease, serine 11A	TMPRSS11A	0,90
ATPase, class V, type 10B	ATP10B	0,90
lysyl oxidase-like 3	LOXL3	0,90
methylsterol monooxygenase 1	MSMO1	0,90
NK3 homeobox 1	NKX3-1	0,90
spindlin family, member 3	SPIN3	0,90
retinoblastoma-like 1	RBL1	0,90
MED14 opposite strand	MED14OS	0,90
suppression of tumorigenicity 18, zinc finger	ST18	0,90
family with sequence similarity 216, member B	FAM216B	0,90
non-SMC condensin I complex subunit H	NCAPH	0,90
zinc finger protein 98	ZNF98	0,90

histatin 3	HTN3	0,90
ubiquitin specific peptidase 11	USP11	0,90
UDP-GlcNAc:betaGal beta-1,3-N-acetylglucosaminyltransferase 6	B3GNT6	0,90
myosin binding protein C, cardiac	MYBPC3	0,90
protocadherin beta 4	PCDHB4	0,90
PARP1 binding protein	PARPBP	0,90
lactate dehydrogenase A	LDHA	0,90
CD69 molecule	CD69	0,90
matrix metallopeptidase 12	MMP12	0,90
monoacylglycerol O-acyltransferase 3	MOGAT3	0,90
tight junction protein 2	TJP2	0,90
ligand of numb-protein X 2	LNX2	0,90
SWI/SNF related, matrix associated, actin dependent regulator of chromatin, subfamily a, member 2	SMARCA2	0,90
synapse associated protein 1	SYAP1	0,90
IZUMO family member 4	IZUMO4	0,90
SIGLEC family like 1	SIGLECL1	0,90
calcium release activated channel regulator 2B	CRACR2B	0,90
leucine rich repeat containing 58	LRRC58	0,90
mastermind-like transcriptional coactivator 2	MAML2	0,90
leukocyte receptor cluster (LRC) member 9	LENG9	0,90
solute carrier family 31 (copper transporter), member 2	SLC31A2	0,90
solute carrier family 9, member C2 (putative)	SLC9C2	0,90
protease-associated domain containing 1	PRADC1	0,90
neuropeptide W	NPW	0,90
WAS protein family homolog 1	WASH1	0,90
immunity-related GTPase family, cinema	IRGC	0,90
actin, gamma 2, smooth muscle, enteric	ACTG2	0,90
glucose-6-phosphatase, catalytic, 2	G6PC2	0,90
chromosome 3 open reading frame 70	C3orf70	0,90
cytochrome b5 domain containing 1	CYB5D1	0,90
PHD finger protein 3	PHF3	0,90
CWF19-like 1, cell cycle control (S. pombe); small nucleolar RNA, H/ACA box 12	CWF19L1; SNORA12	0,90
ring finger and WD repeat domain 3	RFWD3	0,90
Z-DNA binding protein 1	ZBP1	0,90
ADP-dependent glucokinase	ADPGK	0,90
SWI/SNF-related, matrix-associated actin-dependent regulator of chromatin, subfamily a, containing DEAD/H box 1	SMARCAD1	0,90
DNA-damage regulated autophagy modulator 1	DRAM1	0,90
olfactory receptor, family 4, subfamily F, member 29; olfactory receptor, family 4, subfamily F, member 3; olfactory receptor, family 4, subfamily F, member 16	OR4F29; OR4F3; OR4F16	0,90

olfactory receptor, family 4, subfamily F, member 16; olfactory receptor, family 4, subfamily F, member 29	OR4F16; OR4F29	0,90
bone morphogenetic protein/retinoic acid inducible neural-specific 2	BRINP2	0,90
HOXB cluster antisense RNA 3	HOXB-AS3	0,90
cold shock domain containing E1, RNA binding	CSDE1	0,90
ARHGAP19-SLIT1 readthrough (NMD candidate)	ARHGAP19-SLIT1	0,90
glutathione S-transferase zeta 1	GSTZ1	0,90
ectonucleotide pyrophosphatase/phosphodiesterase 1	ENPP1	0,90
ADAM metallopeptidase with thrombospondin type 1 motif 6	ADAMTS6	0,90
golgin A6 family, member A	GOLGA6A	0,90
zinc finger and SCAN domain containing 26	ZSCAN26	0,90
cholinergic receptor, nicotinic beta 2	CHRNB2	0,90
RERG/RAS-like	RERGL	0,90
nicotinamide N-methyltransferase	NNMT	0,90
archain 1	ARCN1	0,90
leucine rich repeat containing 3C	LRRC3C	0,90
synaptotagmin VIII	SYT8	0,90
stress-associated endoplasmic reticulum protein 1	SERP1	0,90
aurora kinase B	AURKB	0,90
progesterone immunomodulatory binding factor 1	PIBF1	0,90
forkhead box N2	FOXN2	0,90
cathepsin L	CTSL	0,90
fucosyltransferase 2 (secretor status included)	FUT2	0,90
splA/ryanodine receptor domain and SOCS box containing 3	SPSB3	0,90
neural precursor cell expressed, developmentally down-regulated 4, E3 ubiquitin protein ligase	NEDD4	0,90
ring finger protein 26	RNF26	0,90
keratin associated protein 5-4	KRTAP5-4	0,90
ubiquitin specific peptidase like 1	USPL1	0,90
KIAA1549-like	KIAA1549L	0,90
mitochondrial ribosomal protein L42	MRPL42	0,90
cytochrome P450, family 2, subfamily C, polypeptide 9	CYP2C9	0,90
chromosome 12 open reading frame 80	C12orf80	0,90
SH3 and multiple ankyrin repeat domains 1	SHANK1	0,90
alpha-fetoprotein	AFP	0,90
RAS guanyl releasing protein 3 (calcium and DAG-regulated)	RASGRP3	0,90
chromosome 22 open reading frame 46	C22orf46	0,90
G protein-coupled receptor kinase 4	GRK4	0,90
platelet-derived growth factor receptor, alpha polypeptide	PDGFRA	0,90
DnaJ (Hsp40) homolog, subfamily C, member 5 gamma	DNAJC5G	0,90
amino-terminal enhancer of split	AES	0,90
SH2 domain containing 1B	SH2D1B	0,90
thioesterase superfamily member 5	THEM5	0,90

pleckstrin homology domain containing, family G (with RhoGef domain) member 2	PLEKHG2	0,90
Tctex1 domain containing 4	TCTEX1D4	0,90
neurotrophin 3	NTF3	0,90
GTP binding protein 1	GTPBP1	0,90
Rieske (Fe-S) domain containing	RFESD	0,90
protein tyrosine phosphatase type IVA, member 2	PTP4A2	0,90
activating transcription factor 5; microRNA 4751	ATF5; MIR4751	0,90
olfactory receptor, family 13, subfamily C, member 5	OR13C5	0,90
dematin actin binding protein	DMTN	0,90
basic helix-loop-helix family, member e41	BHLHE41	0,90
chromosome 22 open reading frame 29; guanine nucleotide binding protein (G protein), beta polypeptide 1-like	C22orf29; GNB1L	0,90
zinc finger protein 93	ZNF93	0,90
proteolipid protein 2 (colonic epithelium-enriched)	PLP2	0,90
ADAMTS like 4	ADAMTSL4	0,90
Rho guanine nucleotide exchange factor 35	ARHGEF35	0,90
oncostatin M receptor	OSMR	0,90
glucosamine-6-phosphate deaminase 2	GNPDA2	0,90
uroporphyrinogen decarboxylase	UROD	0,90
X-prolyl aminopeptidase 3, mitochondrial	XPNPEP3	0,90
Na+/K+ transporting ATPase interacting 1	NKAIN1	0,90
ring finger protein 41, E3 ubiquitin protein ligase	RNF41	0,90
KAT8 regulatory NSL complex subunit 1	KANSL1	0,90
tumor protein p63 regulated 1-like	TPRG1L	0,89
leucine zipper and CTNNBIP1 domain containing	LZIC	0,89
trehalase	TREH	0,89
ribulose-5-phosphate-3-epimerase	RPE	0,89
Transcript Identified by AceView, Entrez Gene ID(s) 23126	POGZ	0,89
par-3 family cell polarity regulator beta	PARD3B	0,89
DEAD (Asp-Glu-Ala-Asp) box polypeptide 50	DDX50	0,89
TBCC domain containing 1	TBCCD1	0,89
TATA element modulatory factor 1	TMF1	0,89
carcinoembryonic antigen-related cell adhesion molecule 7	CEACAM7	0,89
deoxyuridine triphosphatase	DUT	0,89
spermatogenesis and centriole associated 1-like	SPATC1L	0,89
anthrax toxin receptor-like	ANTXRL	0,89
cilia and flagella associated protein 70	CFAP70	0,89
transmembrane protein 245	TMEM245	0,89
poly(A) binding protein, cytoplasmic 1; microRNA 7705	PABPC1; MIR7705	0,89
sirtuin 2	SIRT2	0,89
GTP cyclohydrolase I feedback regulator	GCHFR	0,89

SYNJ2BP-COX16 readthrough; COX16 cytochrome c oxidase assembly homolog; synaptojanin 2 binding protein	SYNJ2BP-COX16; COX16; SYNJ2BP	0,89
neurexophilin 4	NXPH4	0,89
gamma-aminobutyric acid (GABA) A receptor, gamma 2	GABRG2	0,89
phospholipid phosphatase related 5	PLPPR5	0,89
ribosomal protein S27	RPS27	0,89
colony stimulating factor 2 (granulocyte-macrophage)	CSF2	0,89
bladder cancer associated transcript 1 (non-protein coding); LEM domain containing 1	BLACAT1; LEMD1	0,89
katanin p60 (ATPase containing) subunit A 1	KATNA1	0,89
sedoheptulokinase; transient receptor potential cation channel, subfamily V, member 1	SHPK; TRPV1	0,89
pleckstrin homology domain interacting protein	PHIP	0,89
RAS p21 protein activator 3	RASA3	0,89
prostate and testis expressed 4	PATE4	0,89
family with sequence similarity 64, member A	FAM64A	0,89
DEAD (Asp-Glu-Ala-Asp) box polypeptide 31	DDX31	0,89
ribonuclease P/MRP 14kDa subunit	RPP14	0,89
EPS8-like 1	EPS8L1	0,89
Transcript Identified by AceView, Entrez Gene ID(s) 79719	AAGAB	0,89
thyroglobulin	TG	0,89
myosin light chain 10	MYL10	0,89
RAB40A, member RAS oncogene family	RAB40A	0,89
phosphoribosyl pyrophosphate amidotransferase	PPAT	0,89
peroxisomal biogenesis factor 19	PEX19	0,89
transmembrane protein 232	TMEM232	0,89
ATP/GTP binding protein-like 1	AGBL1	0,89
required for meiotic nuclear division 5 homolog B	RMND5B	0,89
aminocarboxymuconate semialdehyde decarboxylase	ACMSD	0,89
RAN binding protein 17	RANBP17	0,89
breast cancer anti-estrogen resistance 3	BCAR3	0,89
left-right determination factor 1	LEFTY1	0,89
mitotic spindle organizing protein 1	MZT1	0,89
monoamine oxidase B	MAOB	0,89
nerve growth factor (beta polypeptide)	NGF	0,89
zinc finger, NFX1-type containing 1	ZNFX1	0,89
acyl-CoA dehydrogenase family, member 9	ACAD9	0,89
intersectin 1	ITSN1	0,89
X antigen family, member 1E; X antigen family, member 1B	XAGE1E; XAGE1B	0,89
torsin family 2, member A	TOR2A	0,89
bone marrow stromal cell antigen 2	BST2	0,89
coiled-coil domain containing 140	CCDC140	0,89
matrix metallopeptidase 26	MMP26	0,89

family with sequence similarity 26, member E	FAM26E	0,89
chromosome 10 open reading frame 88	C10orf88	0,89
CACTIN antisense RNA 1	CACTIN-AS1	0,89
S100 calcium binding protein A4	S100A4	0,89
inducible T-cell co-stimulator ligand	ICOSLG	0,89
Salzman2013 ALT_ACCEPTOR, CDS, coding, INTERNAL, OVCODE, OVEXON best transcript NM_004892; Salzman2013 ANNOTATED, CDS, coding, OVCODE, OVEXON, UTR3 best transcript NM_004892	SEC22B	0,89
ariadne RBR E3 ubiquitin protein ligase 1; microRNA 630	ARIH1; MIR630	0,89
olfactory receptor, family 52, subfamily N, member 1	OR52N1	0,89
Transcript Identified by AceView, Entrez Gene ID(s) 8242	KDM5C	0,89
lectin, galactoside-binding, soluble, 14	LGALS14	0,89
TPX2, microtubule-associated	TPX2	0,89
transcription factor B2, mitochondrial	TFB2M	0,89
DnaJ (Hsp40) homolog, subfamily B, member 9	DNAJB9	0,89
zinc finger protein 780B	ZNF780B	0,89
synaptotagmin XV	SYT15	0,89
transducin-like enhancer of split 3	TLE3	0,89
heterogeneous nuclear ribonucleoprotein C-like 4	HNRNPCL4	0,89
ribosomal protein L7-like 1	RPL7L1	0,89
hexose-6-phosphate dehydrogenase (glucose 1-dehydrogenase)	H6PD	0,89
oligosaccharyltransferase complex subunit (non-catalytic)	OSTC	0,89
sushi-repeat containing protein, X-linked	SRPX	0,89
trafficking protein particle complex 12	TRAPPC12	0,89
olfactory receptor, family 2, subfamily M, member 2	OR2M2	0,89
cell division cycle and apoptosis regulator 1; small nucleolar RNA, C/D box 98	CCAR1; SNORD98	0,89
transmembrane protein 132C	TMEM132C	0,89
olfactory receptor, family 1, subfamily A, member 1	OR1A1	0,89
WD repeat containing, antisense to TP53	WRAP53	0,89
small ArfGAP 1	SMAP1	0,89
histone cluster 1, H4l	HIST1H4L	0,89
dynein, axonemal, heavy chain 6	DNAH6	0,89
thymidine phosphorylase; SCO2 cytochrome c oxidase assembly protein	TYMP; SCO2	0,89
leucyl-tRNA synthetase	LARS	0,89
gap junction protein delta 3	GJD3	0,89
oligodendrocyte myelin glycoprotein	OMG	0,89
olfactory receptor, family 5, subfamily H, member 15; olfactory receptor, family 5, subfamily H, member 5 pseudogene	OR5H15; OR5H5P	0,89
caspase 7	CASP7	0,89
vacuolar protein sorting 37 homolog D (S. cerevisiae)	VPS37D	0,89
sprouty RTK signaling antagonist 2	SPRY2	0,89
teashirt zinc finger homeobox 1	TSHZ1	0,89

paraneoplastic Ma antigen 2	PNMA2	0,89
solute carrier family 25 (mitochondrial carrier; phosphate carrier), member 24	SLC25A24	0,89
intermediate filament family orphan 1	IFFO1	0,89
outer dense fiber of sperm tails 3	ODF3	0,89
tumor protein p53 inducible protein 11	TP53I11	0,89
isopentenyl-diphosphate delta isomerase 1	IDI1	0,89
RAS protein activator like 1 (GAP1 like)	RASAL1	0,89
phosphoinositide-3-kinase, regulatory subunit 4	PIK3R4	0,89
Memczak2013 ALT_ACCEPTOR, ALT_DONOR, coding, INTERNAL, intronic best transcript NM_001174072	SERINC5	0,89
nudix hydrolase 6	NUDT6	0,89
Sjogren syndrome nuclear autoantigen 1	SSNA1	0,89
phosphatidylinositol-4-phosphate 3-kinase, catalytic subunit type 2 alpha	PIK3C2A	0,89
zinc finger protein 628	ZNF628	0,89
WD repeat and HMG-box DNA binding protein 1	WDHD1	0,89
inositol 1,4,5-trisphosphate receptor interacting protein	ITPRIP	0,89
solute carrier family 6 (neurotransmitter transporter), member 7	SLC6A7	0,89
5-hydroxytryptamine (serotonin) receptor 6, G protein-coupled	HTR6	0,89
Transcript Identified by AceView, Entrez Gene ID(s) 56474	CTPS2	0,89
remodeling and spacing factor 1	RSF1	0,89
tetratricopeptide repeat domain 21B	TTC21B	0,89
glutamate receptor, ionotropic, AMPA 4	GRIA4	0,89
myosin XIX	MYO19	0,89
INO80 complex subunit B	INO80B	0,89
peptidyl arginine deiminase, type I	PADI1	0,89
transcription factor AP-2 beta (activating enhancer binding protein 2 beta)	TFAP2B	0,89
protein-O-mannose kinase	POMK	0,89
HECT domain containing E3 ubiquitin protein ligase 3	HECTD3	0,89
histone cluster 1, H3g	HIST1H3G	0,89
alkylglycerone phosphate synthase	AGPS	0,89
N(alpha)-acetyltransferase 30, NatC catalytic subunit	NAA30	0,89
leucine-rich repeat, immunoglobulin-like and transmembrane domains 2	LRIT2	0,89
protocadherin 11 Y-linked	PCDH11Y	0,89
peroxisomal biogenesis factor 11 gamma	PEX11G	0,89
presenilin 2	PSEN2	0,89
leucine zipper, putative tumor suppressor family member 3; Leucine zipper putative tumor suppressor 3 [Source:UniProtKB/Swiss-Prot;Acc:O60299]; Salzman2013 ANNOTATED, CDS, coding, OVCODE, OVEXON, UTR5 best transcript NM_014731	LZTS3; ProSAPiP1	0,89

beta-1,4-N-acetyl-galactosaminyl transferase 1	B4GALNT1	0,89
eukaryotic translation termination factor 1	ETF1	0,89
zinc finger, FYVE domain containing 16	ZFYVE16	0,89
Transcript Identified by AceView, Entrez Gene ID(s) 23355	VPS8	0,89
POTE ankyrin domain family, member B3	POTEB3	0,89
solute carrier family 26 (anion exchanger), member 11	SLC26A11	0,89
glutathione S-transferase theta 2 (gene/pseudogene); glutathione S-transferase theta 2B (gene/pseudogene)	GSTT2; GSTT2B	0,89
lipopolysaccharide binding protein	LBP	0,89
translin-associated factor X	TSNAX	0,89
poly(A) polymerase beta	PAPOLB	0,89
ADAM metallopeptidase domain 7	ADAM7	0,89
angio-associated migratory cell protein	AAMP	0,89
mediator complex subunit 23	MED23	0,89
methyltransferase like 23	METTL23	0,89
follistatin	FST	0,89
netrin 5	NTN5	0,89
oxysterol binding protein-like 8	OSBPL8	0,89
Transcript Identified by AceView, Entrez Gene ID(s) 7184	HSP90B1	0,89
vacuolar protein sorting 25 homolog (S. cerevisiae)	VPS25	0,89
transcription elongation factor A (SII) N-terminal and central domain containing	TCEANC	0,89
olfactory receptor, family 2, subfamily M, member 7	OR2M7	0,89
protein kinase, cAMP-dependent, catalytic, alpha	PRKACA	0,89
SKI proto-oncogene	SKI	0,89
chromosome 9 open reading frame 24	C9orf24	0,89
DMRT-like family C2	DMRTC2	0,89
polypeptide N-acetylgalactosaminyltransferase 13	GALNT13	0,89
ADAM metallopeptidase domain 29	ADAM29	0,89
zyg-11 family member B, cell cycle regulator	ZYG11B	0,89
olfactory receptor, family 51, subfamily I, member 1	OR51I1	0,89
asparaginase	ASPG	0,89
enhancer of rudimentary homolog (Drosophila)	ERH	0,89
membrane protein, palmitoylated 5	MPP5	0,89
oligophrenin 1	OPHN1	0,89
acid sensing ion channel 3	ASIC3	0,89
KIAA1147	KIAA1147	0,89
solute carrier family 25, member 30	SLC25A30	0,89
mitochondrial transcription termination factor 3	MTERF3	0,89
LSM1 homolog, mRNA degradation associated	LSM1	0,89
astrotactin 1	ASTN1	0,89
neural precursor cell expressed, developmentally down-regulated 4-like, E3 ubiquitin protein ligase	NEDD4L	0,89
cysteine-rich protein 2	CRIP2	0,89
COP9 signalosome subunit 4	COPS4	0,89

phosphoinositide-3-kinase, regulatory subunit 5	PIK3R5	0,89
RNA binding motif protein, X-linked-like 2	RBMXL2	0,89
Rho GTPase activating protein 44	ARHGAP44	0,89
vacuolar protein sorting 33 homolog B (yeast)	VPS33B	0,89
NIM1 serine/threonine protein kinase	NIM1K	0,89
phosphatidylinositol-specific phospholipase C, X domain containing 1	PLCXD1	0,89
KIAA0430; microRNA 6506	KIAA0430; MIR6506	0,89
hydroxymethylbilane synthase	HMBS	0,89
cytochrome c oxidase subunit IV isoform 1	COX4I1	0,89
centromere protein Q	CENPQ	0,89
uncharacterized LOC101928877; novel transcript; Transcript Identified by AceView, Entrez Gene ID(s) 291	LOC101928877; RP11-386B13.3; SLC25A4	0,89
UDP glucuronosyltransferase 2 family, polypeptide B28	UGT2B28	0,89
Cbp/p300-interacting transactivator, with Glu/Asp rich carboxy-terminal domain, 1	CITED1	0,89
ADAM metallopeptidase with thrombospondin type 1 motif 13	ADAMTS13	0,89
Jeck2013 ALT_ACCEPTOR, ALT_DONOR, coding, INTERNAL, intronic best transcript NM_032531	KIRREL3	0,89
D4, zinc and double PHD fingers family 1	DPF1	0,89
DEP domain containing MTOR-interacting protein	DEPTOR	0,89
Transcript Identified by AceView, Entrez Gene ID(s) 57181	SLC39A10	0,89
additional sex combs like transcriptional regulator 3	ASXL3	0,89
RAD21 antisense RNA 1	RAD21-AS1	0,89
protein phosphatase 2, regulatory subunit B, gamma	PPP2R2C	0,89
solute carrier family 26 (anion exchanger), member 9	SLC26A9	0,89
synovial sarcoma, X breakpoint 9	SSX9	0,89
ribosomal protein L7a	RPL7A	0,89
membrane-spanning 4-domains, subfamily A, member 12	MS4A12	0,89
ribosomal protein L18	RPL18	0,89
claudin 15	CLDN15	0,89
chromosome 6 open reading frame 183	C6orf183	0,89
fucosyltransferase 6 (alpha (1,3) fucosyltransferase)	FUT6	0,89
glycine N-methyltransferase	GNMT	0,89
membrane bound transcription factor peptidase, site 2; YY2 transcription factor	MBTPS2; YY2	0,89
signal-regulatory protein beta 2	SIRPB2	0,89
TNF receptor-associated factor 2	TRAF2	0,89
pannexin 3	PANX3	0,89
ATP binding cassette subfamily A member 8	ABCA8	0,89
SPARC related modular calcium binding 2	SMOC2	0,89

ARMCX5-GPRASP2 readthrough	ARMCX5-GPRASP2	0,89
RAB6B, member RAS oncogene family	RAB6B	0,89
ras homolog family member T2	RHOT2	0,89
rhomboid 5 homolog 1 (Drosophila)	RHBDF1	0,89
v-maf avian musculoaponeurotic fibrosarcoma oncogene homolog G	MAFG	0,89
HD domain containing 2	HDDC2	0,89
chromosome 11 open reading frame 54	C11orf54	0,89
cornulin	CRNN	0,89
NPR2-like, GATOR1 complex subunit	NPRL2	0,89
exophilin 5	EXPH5	0,89
Transcript Identified by AceView, Entrez Gene ID(s) 22985	ACIN1	0,89
Transcript Identified by AceView, Entrez Gene ID(s) 387535	HCRP1	0,89
Transcript Identified by AceView, Entrez Gene ID(s) 3069	HDLBP	0,89
uncharacterized LOC389831; Salzman2013 ANNOTATED, CDS, coding, OVCODE, OVEXON, UTR3 best transcript NM_001242480; Salzman2013 ANNOTATED, CDS, coding, OVCODE, OVEXON, UTR3, UTR5 best transcript NM_001242480; Salzman2013 ANNOTATED, ncRNA, OVEXON best transcript TCONS_00030034; Salzman2013 ANNOTATED, ncRNA, OVCODE, OVEXON best transcript TCONS_00030034; Salzman2013 ALT_ACCEPTOR, ncRNA, OVCODE, OVEXON, upstream_start best transcript TCONS_00030034; Salzman2013 ALT_DONOR, downstream_end, ncRNA, OVEXON best transcript TCONS_00030035; Salzman2013 ALT_DONOR, coding, intronic, OVEXON, UTR5 best transcript NM_001242480; uncharacterized protein LOC389831 [Source:RefSeq peptide;Acc:NP_001229409]	LOC389831; TCONS_00030034; TCONS_00030035; AC011043.1	0,89
CDC42 effector protein (Rho GTPase binding) 4	CDC42EP4	0,89
collagen, type I, alpha 1	COL1A1	0,89
cytochrome c oxidase subunit Va	COX5A	0,89
olfactory receptor family 10 subfamily A member 6 (gene/pseudogene) [Source:HGNC Symbol;Acc:HGNC:15132]	OR10A6	0,89
centrosomal protein 152kDa	CEP152	0,89
GATS protein-like 3	GATSL3	0,89
podocan	PODN	0,89
coiled-coil and C2 domain containing 2B	CC2D2B	0,89
potassium channel, voltage gated Shal related subfamily D, member 1	KCND1	0,89
Jeck2013 ANTISENSE, CDS, coding, INTERNAL, intronic, OVCODE, OVEXON best transcript NM_002473	MYH9	0,89
ankyrin repeat domain 7	ANKRD7	0,89
Transcript Identified by AceView, Entrez Gene ID(s) 51684	SUFU	0,89
galanin receptor 3	GALR3	0,89

peptidyl arginine deiminase, type IV	PADI4	0,89
sucrase-isomaltase (alpha-glucosidase)	SI	0,89
eosinophil peroxidase	EPX	0,89
tumor necrosis factor (ligand) superfamily, member 10	TNFSF10	0,89
zinc finger E-box binding homeobox 1	ZEB1	0,89
LSM8 homolog, U6 small nuclear RNA associated	LSM8	0,89
cell division cycle 14B	CDC14B	0,89
G protein-coupled receptor 88	GPR88	0,89
olfactory receptor, family 52, subfamily H, member 1	OR52H1	0,89
achaete-scute family bHLH transcription factor 1	ASCL1	0,89
olfactory receptor, family 9, subfamily G, member 1; olfactory receptor, family 9, subfamily G, member 9	OR9G1; OR9G9	0,89
ribosomal L1 domain containing 1	RSL1D1	0,89
nuclear envelope integral membrane protein 2	NEMP2	0,89
N-acetylneuraminate pyruvate lyase (dihydrodipicolinate synthase)	NPL	0,89
interferon-induced protein 44-like	IFI44L	0,89
chromosome 15 open reading frame 62	C15orf62	0,89
adaptor-related protein complex 4, epsilon 1 subunit	AP4E1	0,89
nuclear receptor coactivator 4	NCOA4	0,89
glycine receptor alpha 2	GLRA2	0,89
G protein-coupled receptor 107	GPR107	0,89
single-pass membrane protein with aspartate-rich tail 1	SMDT1	0,89
alcohol dehydrogenase 1A (class I), alpha polypeptide	ADH1A	0,89
oxysterol binding protein-like 1A	OSBPL1A	0,89
ring finger protein, LIM domain interacting	RLIM	0,89
solute carrier family 24 (sodium/potassium/calcium exchanger), member 4	SLC24A4	0,89
WD repeat domain 49	WDR49	0,89
gamma-glutamyltransferase light chain 1	GGTLC1	0,89
Ras converting CAAX endopeptidase 1; chromosome 11 open reading frame 80	RCE1; C11orf80	0,89
olfactory receptor, family 51, subfamily T, member 1	OR51T1	0,89
S100P binding protein	S100PBP	0,89
zinc finger protein 791	ZNF791	0,89
zinc finger protein 311	ZNF311	0,89
DnaJ (Hsp40) homolog, subfamily A, member 3	DNAJA3	0,89
kaptin (actin binding protein)	KPTN	0,89
olfactory receptor, family 1, subfamily G, member 1	OR1G1	0,89
nucleoporin 37kDa	NUP37	0,89
ATPase, Na+/K+ transporting, beta 2 polypeptide	ATP1B2	0,89
leukocyte receptor cluster (LRC) member 1	LENG1	0,89
transmembrane protein 247	TMEM247	0,89
major histocompatibility complex, class II, DP beta 1	HLA-DPB1	0,89
coiled-coil domain containing 62	CCDC62	0,89

connector enhancer of kinase suppressor of Ras 2	CNKSR2	0,89
major histocompatibility complex, class I, V (pseudogene)	HLA-V	0,89
family with sequence similarity 111, member B	FAM111B	0,89
transgelin 2	TAGLN2	0,89
phospholipase A2 receptor 1	PLA2R1	0,89
Transcript Identified by AceView, Entrez Gene ID(s) 27301	APEX2	0,89
zinc finger protein 385D	ZNF385D	0,89
YTH N(6)-methyladenosine RNA binding protein 3	YTHDF3	0,89
zinc finger protein 276	ZNF276	0,89
TIMP metallopeptidase inhibitor 3	TIMP3	0,89
glia maturation factor, beta	GMFB	0,89
hepatoma-derived growth factor-related protein 2; Hepatoma-derived growth factor-related protein 2 [Source:UniProtKB/Swiss-Prot;Acc:Q7Z4V5]; Salzman2013 ANNOTATED, CDS, coding, OVCODE, OVEXON, UTR5 best transcript NM_001001520; Salzman2013 ANNOTATED, CDS, coding, OVCODE, OVERLAPTX, OVEXON, UTR3, UTR5 best transcript NM_001001520; Salzman2013 ANNOTATED, CDS, coding, INTERNAL, OVCODE, OVEXON best transcript NM_001001520; Salzman2013 ALT_ACCEPTOR, CDS, coding, INTERNAL, intronic, OVCODE, OVEXON best transcript NM_001001520; Salzman2013 ANNOTATED, CDS, coding, OVCODE, OVERLAPTX, OVEXON, UTR3 best transcript NM_001001520; Salzman2013 ALT_DONOR, CDS, coding, INTERNAL, OVCODE, OVERLAPTX, OVEXON, UTR3 best transcript NM_001001520	HDGFRP2	0,89
ring finger and FYVE-like domain containing E3 ubiquitin protein ligase	RFFL	0,89
epithelial splicing regulatory protein 1	ESRP1	0,89
cytochrome P450, family 2, subfamily J, polypeptide 2	CYP2J2	0,89
PHD finger protein 19	PHF19	0,89
fatty acid binding protein 5 (psoriasis-associated)	FABP5	0,89
RAB3D, member RAS oncogene family	RAB3D	0,89
peroxisomal biogenesis factor 12	PEX12	0,89
serine/threonine kinase-like domain containing 1 [Source:HGNC Symbol;Acc:HGNC:28669]	STKLD1	0,89
PAS domain containing serine/threonine kinase	PASK	0,89
mesoderm posterior bHLH transcription factor 1	MESP1	0,89
Rac GTPase activating protein 1	RACGAP1	0,89
M-phase phosphoprotein 9	MPHOSPH9	0,89
leucine rich repeat transmembrane neuronal 2	LRRTM2	0,89
mitogen-activated protein kinase kinase kinase 4	MAP3K4	0,89
olfactory receptor, family 2, subfamily J, member 2	OR2J2	0,89
golgin A7	GOLGA7	0,89
serine/arginine repetitive matrix 1	SRRM1	0,89

prolactin receptor	PRLR	0,89
frizzled class receptor 9	FZD9	0,89
ethylmalonyl-CoA decarboxylase 1	ECHDC1	0,89
mannan-binding lectin serine peptidase 2	MASP2	0,89
forkhead box O3	FOXO3	0,89
spastin	SPAST	0,89
E2F transcription factor 7	E2F7	0,89
shroom family member 4	SHROOM4	0,89
vascular endothelial growth factor C	VEGFC	0,89
RAB14, member RAS oncogene family	RAB14	0,89
C-type lectin domain family 3, member A	CLEC3A	0,89
transmembrane protein 225-like; Jeck2013 ALT_ACCEPTOR, ALT_DONOR, ncRNA, OVCODE, OVEXON, upstream_start best transcript TCONS_l2_00026125; Salzman2013 ANNOTATED, INTERNAL, ncRNA, OVCODE, OVEXON best transcript TCONS_l2_00026126; novel transcript; Transcript Identified by AceView, Entrez Gene ID(s) 79027	GS1-259H13.2; TCONS_l2_00026125; TCONS_l2_00026126; ZNF655	0,89
solute carrier family 29 (equilibrative nucleoside transporter), member 3	SLC29A3	0,89
feline leukemia virus subgroup C cellular receptor 1	FLVCR1	0,89
hyaluronan and proteoglycan link protein 2	HAPLN2	0,89
minichromosome maintenance complex component 3	MCM3	0,89
TRK-fused gene	TFG	0,89
opticin	OPTC	0,89
protein regulator of cytokinesis 1	PRC1	0,89
Rho GTPase activating protein 11B	ARHGAP11B	0,89
protein phosphatase 1, catalytic subunit, beta isozyme	PPP1CB	0,89
U6 snRNA biogenesis 1	USB1	0,89
growth differentiation factor 2	GDF2	0,89
proline rich 33	PRR33	0,89
POU class 4 homeobox 2	POU4F2	0,89
germ cell associated 1	GSG1	0,89
reactive oxygen species modulator 1	ROMO1	0,89
olfactory receptor, family 5, subfamily M, member 9	OR5M9	0,89
trafficking protein particle complex 4	TRAPPC4	0,89
uncharacterized LOC651430; uncharacterized LOC651430 [Source:EntrezGene;Acc:651430]; novel transcript	FLJ38576; RP11-173M11.6	0,89
dynein, axonemal, light intermediate chain 1	DNALI1	0,89
sorting nexin 25	SNX25	0,89
ribosomal protein S6 kinase, 90kDa, polypeptide 3	RPS6KA3	0,89
Transcript Identified by AceView, Entrez Gene ID(s) 1837	DTNA	0,89
unc-51 like autophagy activating kinase 2	ULK2	0,89
ADAM metallopeptidase with thrombospondin type 1 motif 14	ADAMTS14	0,89
seizure related 6 homolog (mouse)	SEZ6	0,89

RANBP2-like and GRIP domain containing 6; RANBP2-like and GRIP domain containing 5	RGPD6; RGPD5	0,89
family with sequence similarity 195, member B	FAM195B	0,89
chromosome 8 open reading frame 58	C8orf58	0,89
REX4 homolog, 3'-5' exonuclease [Source:HGNC Symbol;Acc:HGNC:12820]	REXO4	0,89
nucleoredoxin-like 2	NXNL2	0,89
MGAT4 family, member E, pseudogene	MGAT4EP	0,89
chromosome 1 open reading frame 186	C1orf186	0,89
transmembrane protein 132E	TMEM132E	0,89
zinc finger CCCH-type, antiviral 1	ZC3HAV1	0,89
family with sequence similarity 187, member B	FAM187B	0,89
progestin and adipoQ receptor family member V	PAQR5	0,89
aldehyde dehydrogenase 3 family, member B1	ALDH3B1	0,89
piggyBac transposable element derived 4	PGBD4	0,89
Memczak2013 ANTISENSE, CDS, coding, INTERNAL best transcript NM_020175	DUS3L	0,89
ring finger protein 169	RNF169	0,89
plasminogen	PLG	0,89
leucine rich repeat containing 23	LRRC23	0,89
succinate-CoA ligase, GDP-forming, beta subunit	SUCLG2	0,89
HIG1 hypoxia inducible domain family, member 1A	HIGD1A	0,89
adenylosuccinate synthase like 1	ADSSL1	0,89
phosphatidylinositol-specific phospholipase C, X domain containing 1	PLCXD1	0,89
Homo sapiens similar to testis specific protein, Y-linked 1, mRNA (cDNA clone MGC:150413 IMAGE:40120433), complete cds.; Homo sapiens testis specific protein, Y-linked 3, mRNA (cDNA clone MGC:150414 IMAGE:40120435), complete cds.	LOC728395; TSPY3	0,89
THO complex 6	THOC6	0,89
BPI fold containing family B, member 3	BPIFB3	0,89
fibroblast growth factor 16	FGF16	0,89
keratin associated protein 4-5	KRTAP4-5	0,89
chromosome 11 open reading frame 98; LBH domain containing 1	C11orf98; LBHD1	0,89
GATA zinc finger domain containing 1	GATAD1	0,89
solute carrier family 39, member 9	SLC39A9	0,89
astacin-like metallo-endopeptidase (M12 family)	ASTL	0,89
transmembrane protein 214	TMEM214	0,89
dual specificity phosphatase 11	DUSP11	0,89
non-SMC condensin II complex subunit H2	NCAPH2	0,89
capping protein (actin filament) muscle Z-line, alpha 3	CAPZA3	0,89
small nuclear ribonucleoprotein polypeptides B and B1	SNRPB	0,89
potassium channel tetramerization domain containing 7	KCTD7	0,89

Homo sapiens TMLHE antisense RNA 1 (TMLHE-AS1), long non-coding RNA.; uncharacterized LOC101927830; Transcript Identified by AceView, Entrez Gene ID(s) 1193; novel transcript	TMLHE-AS1; LOC101927830; CLIC2; RP13-228J13.1	0,89
Transcript Identified by AceView; Jeck2013 ALT_ACCEPTOR, ALT_DONOR, coding, INTERNAL, intronic best transcript NM_001003679; putative novel transcript	boyboy; RP4-630A11.3; LEPR	0,89
staufen double-stranded RNA binding protein 2	STAU2	0,89
MHC class I polypeptide-related sequence B	MICB	0,89
transglutaminase 4	TGM4	0,89
chromosome 2 open reading frame 78	C2orf78	0,89
chemokine (C-X3-C motif) ligand 1	CX3CL1	0,89
complement component 1, r subcomponent	C1R	0,89
pericentriolar material 1	PCM1	0,89
tripartite motif containing 62	TRIM62	0,89
olfactory receptor family 4 subfamily E member 1 (gene/pseudogene) [Source:HGNC Symbol;Acc:HGNC:8296]	OR4E1	0,89
hexosaminidase A (alpha polypeptide)	HEXA	0,89
integrin beta like 1	ITGBL1	0,89
lamin B receptor	LBR	0,89
Transcript Identified by AceView, Entrez Gene ID(s) 3707	ITPKB	0,89
F-box protein 36	FBXO36	0,89
Transcript Identified by AceView, Entrez Gene ID(s) 8470	SORBS2	0,89
histone cluster 1, H2bb	HIST1H2BB	0,89
protein-kinase, interferon-inducible double stranded RNA dependent inhibitor, repressor of (P58 repressor)	PRKRIR	0,89
zinc finger protein 503	ZNF503	0,89
dynein, axonemal, heavy chain 14	DNAH14	0,89
novel transcript; Transcript Identified by AceView, Entrez Gene ID(s) 54453	RP1-122P22.2; RIN2	0,89
ribosomal protein L7a	RPL7A	0,89
signal sequence receptor, alpha	SSR1	0,89
vomeronasal 1 receptor 1	VN1R1	0,89
sorting nexin 19	SNX19	0,89
collagen, type IV, alpha 2	COL4A2	0,89
CREB binding protein	CREBBP	0,89
transmembrane 4 L six family member 5	TM4SF5	0,89
late endosomal/lysosomal adaptor, MAPK and MTOR activator 4	LAMTOR4	0,89
doublecortin-like kinase 1	DCLK1	0,89
INTS3 and NABP interacting protein	INIP	0,89
calcium channel, voltage-dependent, beta 3 subunit	CACNB3	0,89
polymerase (RNA) II (DNA directed) polypeptide B, 140kDa	POLR2B	0,89
mevalonate (diphospho) decarboxylase	MVD	0,89
anillin actin binding protein	ANLN	0,89

B-cell CLL/lymphoma 2	BCL2	0,89
cholinergic receptor, muscarinic 4	CHRM4	0,89
albumin	ALB	0,89
Myb-related transcription factor, partner of profilin	MYPOP	0,89
chromosome 17 open reading frame 98	C17orf98	0,88
leucine-rich repeat containing G protein-coupled receptor 4	LGR4	0,88
X antigen family, member 3	XAGE3	0,88
cilia and flagella associated protein 97	CFAP97	0,88
protein phosphatase 1, regulatory subunit 12C	PPP1R12C	0,88
myosin IIIB	MYO3B	0,88
contactin associated protein-like 5	CNTNAP5	0,88
NANOG neighbor homeobox	NANOGNB	0,88
coiled-coil domain containing 36	CCDC36	0,88
zinc finger protein 620	ZNF620	0,88
uridine monophosphate synthetase	UMPS	0,88
paired box 7	PAX7	0,88
centrosomal protein 41kDa	CEP41	0,88
non-specific cytotoxic cell receptor protein 1 homolog (zebrafish)	NCCRP1	0,88
nanos homolog 3 (Drosophila)	NANOS3	0,88
SLU7 homolog, splicing factor	SLU7	0,88
dachshund family transcription factor 1	DACH1	0,88
poly(A) binding protein, cytoplasmic 1-like 2A	PABPC1L2A	0,88
centromere protein O	CENPO	0,88
transmembrane protein 266	TMEM266	0,88
DEAD (Asp-Glu-Ala-Asp) box polypeptide 23	DDX23	0,88
proline/histidine/glycine-rich 1	PHGR1	0,88
FK506 binding protein 1B	FKBP1B	0,88
Rho GDP dissociation inhibitor (GDI) gamma	ARHGDIG	0,88
thyroid stimulating hormone, beta	TSHB	0,88
histone cluster 1, H2bg	HIST1H2BG	0,88
selenoprotein K; selenoprotein K [Source:EntrezGene;Acc:58515]; Transcript Identified by AceView, Entrez Gene ID(s) 58515, RefSeq ID(s) NM_021237	SELK	0,88
HSPE1-MOB4 readthrough	HSPE1-MOB4	0,88
SCY1-like, kinase-like 3	SCYL3	0,88
sentan, cilia apical structure protein	SNTN	0,88
VPS35 retromer complex component	VPS35	0,88
polycystic kidney disease 1 like 1	PKD1L1	0,88
chromosome 11 open reading frame 87	C11orf87	0,88
forkhead box O4	FOXO4	0,88
gap junction protein alpha 1	GJA1	0,88
apolipoprotein A-II	APOA2	0,88
apolipoprotein O-like	APOOL	0,88
DSN1 homolog, MIS12 kinetochore complex component	DSN1	0,88

interleukin 19	IL19	0,88
zinc finger protein 681	ZNF681	0,88
tripartite motif containing 28	TRIM28	0,88
G elongation factor, mitochondrial 1	GFM1	0,88
ankyrin repeat domain 53	ANKRD53	0,88
family with sequence similarity 9, member A	FAM9A	0,88
coiled-coil domain containing 47	CCDC47	0,88
KH homology domain containing 1	KHDC1	0,88
polypeptide N-acetylgalactosaminyltransferase 9	GALNT9	0,88
transmembrane protease, serine 11E	TMPRSS11E	0,88
GRAM domain containing 3	GRAMD3	0,88
tubulin tyrosine ligase-like family member 12	TTLL12	0,88
SUMO1/sentrin specific peptidase 5	SENP5	0,88
CD180 molecule	CD180	0,88
golgi SNAP receptor complex member 2	GOSR2	0,88
insulinoma-associated 2	INSM2	0,88
complement factor H-related 3	CFHR3	0,88
nuclear factor of activated T-cells, cytoplasmic, calcineurin-dependent 3	NFATC3	0,88
protein phosphatase 1, regulatory (inhibitor) subunit 11	PPP1R11	0,88
G protein-coupled receptor 161	GPR161	0,88
patched domain containing 3	PTCHD3	0,88
olfactory receptor, family 52, subfamily B, member 4 (gene/pseudogene)	OR52B4	0,88
ribosomal protein S6 kinase, 90kDa, polypeptide 1	RPS6KA1	0,88
latent transforming growth factor beta binding protein 4	LTBP4	0,88
olfactory receptor, family 2, subfamily L, member 5	OR2L5	0,88
basic charge, Y-linked, 2B	BPY2B	0,88
stratifin	SFN	0,88
spermatogenesis associated 6	SPATA6	0,88
chromosome 9 open reading frame 92	C9orf92	0,88
phospholipase C, beta 1 (phosphoinositide-specific)	PLCB1	0,88
acid phosphatase 1, soluble	ACP1	0,88
minichromosome maintenance complex component 2	MCM2	0,88
adaptor-related protein complex 4, beta 1 subunit	AP4B1	0,88
family with sequence similarity 127, member C	FAM127C	0,88
transmembrane protein 39A	TMEM39A	0,88
chromosome X open reading frame 23	CXorf23	0,88
ubiquitin conjugating enzyme E2D 1	UBE2D1	0,88
integrin alpha D	ITGAD	0,88
chromosome 5 open reading frame 51	C5orf51	0,88
AAR2 splicing factor homolog	AAR2	0,88
transmembrane protein 186	TMEM186	0,88
ST8 alpha-N-acetyl-neuraminide alpha-2,8-sialyltransferase 3	ST8SIA3	0,88
activin A receptor type IC	ACVR1C	0,88

TBC1D3P1-DHX40P1 readthrough transcribed pseudogene	TBC1D3P1-DHX40P1	0,88
pyruvate kinase, muscle	PKM	0,88
ubiquitin specific peptidase 41	USP41	0,88
actin, alpha 1, skeletal muscle	ACTA1	0,88
dual specificity phosphatase 26 (putative)	DUSP26	0,88
microRNA 1199	MIR1199	0,88
N(alpha)-acetyltransferase 38, NatC auxiliary subunit	NAA38	0,88
golgin A3	GOLGA3	0,88
sodium channel, voltage gated, type XI alpha subunit	SCN11A	0,88
myeloid leukemia factor 2	MLF2	0,88
polypeptide N-acetylgalactosaminyltransferase 8	GALNT8	0,88
Salzman2013 ANTISENSE, coding, INTERNAL, intronic best transcript NM_001034845	GALNTL6	0,88
peptidylprolyl isomerase G (cyclophilin G)	PPIG	0,88
myosin light chain kinase 2	MYLK2	0,88
Memczak2013 ANTISENSE, coding, INTERNAL, intronic best transcript NM_006322	TUBGCP3	0,88
Wilms tumor 1	WT1	0,88
ring finger protein 111	RNF111	0,88
transportin 2	TNPO2	0,88
p21 protein (Cdc42/Rac)-activated kinase 4	PAK4	0,88
Homo sapiens chromosome X open reading frame 51A (CXorf51A), mRNA.; Homo sapiens chromosome X open reading frame 51B (CXorf51B), mRNA.; Transcript Identified by AceView, Entrez Gene ID(s) 100129239, RefSeq ID(s) NM_001144064; chromosome X open reading frame 51A [Source:HGNC Symbol;Acc:HGNC:30533]; chromosome X open reading frame 51	CXorf51A; CXorf51B; CXorf51	0,88
four jointed box 1	FJX1	0,88
MORC family CW-type zinc finger 1	MORC1	0,88
Treacher Collins-Franceschetti syndrome 1	TCOF1	0,88
secretoglobin, family 1A, member 1 (uteroglobin)	SCGB1A1	0,88
zinc finger and BTB domain containing 45	ZBTB45	0,88
ring finger protein 133	RNF133	0,88
lectin, galactoside-binding, soluble, 9C; lectin, galactoside-binding, soluble, 9B	LGALS9C; LGALS9B	0,88
Kruppel-like factor 5 (intestinal)	KLF5	0,88
protein phosphatase 1, regulatory subunit 36	PPP1R36	0,88
myo-inositol oxygenase	MIOX	0,88
glucoside xylosyltransferase 1	GXYLT1	0,88
Memczak2013 ANTISENSE, CDS, coding, INTERNAL best transcript NM_018031	WDR6	0,88
ankyrin repeat domain 13A	ANKRD13A	0,88
dual specificity phosphatase 6	DUSP6	0,88

ADAM metallopeptidase with thrombospondin type 1 motif 13	ADAMTS13	0,88
mediator complex subunit 20	MED20	0,88
zinc finger and BTB domain containing 38	ZBTB38	0,88
glucosamine (UDP-N-acetyl)-2-epimerase/N-acetylmannosamine kinase	GNE	0,88
sortilin-related receptor, L(DLR class) A repeats containing	SORL1	0,88
sprouty-related, EVH1 domain containing 1	SPRED1	0,88
Memczak2013 ALT_ACCEPTOR, ALT_DONOR, coding, INTERNAL, intronic best transcript NM_018460	ARHGAP15	0,88
ATP binding cassette subfamily G member 1	ABCG1	0,88
PR domain containing 11	PRDM11	0,88
cytochrome b561 family, member D1	CYB561D1	0,88
family with sequence similarity 210, member A	FAM210A	0,88
olfactory receptor, family 1, subfamily E, member 1	OR1E1	0,88
mucin-like 1	MUCL1	0,88
cell adhesion molecule L1-like	CHL1	0,88
suppression of tumorigenicity 14 (colon carcinoma)	ST14	0,88
family with sequence similarity 126, member B	FAM126B	0,88
FCF1 rRNA-processing protein	FCF1	0,88
secreted LY6/PLAUR domain containing 1	SLURP1	0,88
PCOLCE antisense RNA 1	PCOLCE-AS1	0,88
THAP domain containing 6	THAP6	0,88
leptin receptor; leptin receptor overlapping transcript	LEPR; LEPROT	0,88
Transcript Identified by AceView, Entrez Gene ID(s) 8565	YARS	0,88
prolactin releasing hormone	PRLH	0,88
membrane metallo-endopeptidase	MME	0,88
taxilin gamma pseudogene, Y-linked	TXLNGY	0,88
apolipoprotein B mRNA editing enzyme, catalytic polypeptide-like 3C	APOBEC3C	0,88
glutathione S-transferase omega 2	GSTO2	0,88
transmembrane protein 263	TMEM263	0,88
glutathione peroxidase 5	GPX5	0,88
small muscle protein, X-linked	SMPX	0,88
phosphodiesterase 10A	PDE10A	0,88
arylsulfatase B	ARSB	0,88
keratin associated protein 9-9	KRTAP9-9	0,88
embryonal Fyn-associated substrate	EFS	0,88
ATP synthase, H+ transporting, mitochondrial Fo complex subunit s (factor B)	ATP5S	0,88
high mobility group nucleosomal binding domain 4	HMGN4	0,88
echinoderm microtubule associated protein like 4	EML4	0,88
dynein, axonemal, light chain 1	DNAL1	0,88
olfactory receptor, family 10, subfamily G, member 2	OR10G2	0,88
methyl-CpG binding domain 4 DNA glycosylase	MBD4	0,88
zinc finger, RAN-binding domain containing 3	ZRANB3	0,88

ATP binding cassette subfamily E member 1	ABCE1	0,88
transmembrane 6 superfamily member 2	TM6SF2	0,88
family with sequence similarity 156, member B; family with sequence similarity 156, member A	FAM156B; FAM156A	0,88
MAP7 domain containing 3	MAP7D3	0,88
pregnancy specific beta-1-glycoprotein 6	PSG6	0,88
olfactory receptor, family 10, subfamily W, member 1	OR10W1	0,88
cytidine monophosphate (UMP-CMP) kinase 1, cytosolic	CMPK1	0,88
Transcript Identified by AceView, Entrez Gene ID(s) 5800	PTPRO	0,88
LAS1-like, ribosome biogenesis factor	LAS1L	0,88
paired box 5	PAX5	0,88
Memczak2013 ALT_ACCEPTOR, ALT_DONOR, coding, INTERNAL, intronic best transcript NM_001166007	EPB41	0,88
golgin A8 family, member D, pseudogene	GOLGA8DP	0,88
KIAA0895	KIAA0895	0,88
lactation elevated 1	LACE1	0,88
BCL2-associated athanogene	BAG1	0,88
secretin receptor	SCTR	0,88
cerebral endothelial cell adhesion molecule	CERCAM	0,88
olfactory receptor, family 2, subfamily W, member 5 (gene/pseudogene)	OR2W5	0,88
kelch-like family member 11	KLHL11	0,88
Memczak2013 ALT_ACCEPTOR, ALT_DONOR, coding, INTERNAL, intronic best transcript NM_014957	DENND3	0,88
MMS22-like, DNA repair protein	MMS22L	0,88
5-nucleotidase domain containing 2	NT5DC2	0,88
ribosomal protein S4, Y-linked 2	RPS4Y2	0,88
cysteine conjugate-beta lyase, cytoplasmic	CCBL1	0,88
tripartite motif containing 15	TRIM15	0,88
coiled-coil alpha-helical rod protein 1	CCHCR1	0,88
SKI-like proto-oncogene	SKIL	0,88
serine/arginine-rich splicing factor 3	SRSF3	0,88
kinectin 1 (kinesin receptor)	KTN1	0,88
solute carrier family 22 (organic anion transporter), member 8	SLC22A8	0,88
alpha-2-macroglobulin-like 1	A2ML1	0,88
primase, DNA, polypeptide 1 (49kDa)	PRIM1	0,88
XRCC6 binding protein 1	XRCC6BP1	0,88
forkhead box L1-like; novel transcript; Transcript Identified by AceView	WI2-2373I1.2; pajoyby	0,88
coatomer protein complex subunit beta 2 (beta prime)	COPB2	0,88
mediator complex subunit 25; microRNA 6800	MED25; MIR6800	0,88
phosphatidylinositol glycan anchor biosynthesis class O	PIGO	0,88
AT hook containing transcription factor 1	AHCTF1	0,88
snail family zinc finger 1	SNAI1	0,88

POZ (BTB) and AT hook containing zinc finger 1	PATZ1	0,88
CD248 molecule, endosialin	CD248	0,88
Transcript Identified by AceView, Entrez Gene ID(s) 3839	KPNA3	0,88
u3 small nucleolar ribonucleoprotein protein MPP10-like	LOC643802	0,88
caspase recruitment domain family, member 19	CARD19	0,88
olfactory receptor, family 13, subfamily C, member 4	OR13C4	0,88
keratin associated protein 9-4	KRTAP9-4	0,88
R-spondin 4	RSPO4	0,88
chromosome 19 open reading frame 48; small nucleolar RNA, C/D box 88B; small nucleolar RNA, C/D box 88C	C19orf48; SNORD88B; SNORD88C	0,88
RPGRIP1-like	RPGRIP1L	0,88
cholinergic receptor, nicotinic alpha 9	CHRNA9	0,88
hippocalcin-like 1	HPCAL1	0,88
E2F transcription factor 8	E2F8	0,88
tropomodulin 1	TMOD1	0,88
chloride intracellular channel 5	CLIC5	0,88
zinc finger, CCHC domain containing 3	ZCCHC3	0,88
ladybird homeobox 2	LBX2	0,88
family with sequence similarity 71, member B	FAM71B	0,88
chromosome 1 open reading frame 198	C1orf198	0,88
transmembrane channel like 5	TMC5	0,88
dual specificity phosphatase 1	DUSP1	0,88
meprin A, alpha (PABA peptide hydrolase)	MEP1A	0,88
granzyme M	GZMM	0,88
split hand/foot malformation (ectrodactyly) type 1	SHFM1	0,88
vacuolar protein sorting 37 homolog B (S. cerevisiae)	VPS37B	0,88
translocase of inner mitochondrial membrane 8 homolog A (yeast)	TIMM8A	0,88
adenosine monophosphate deaminase 3	AMPD3	0,88
solute carrier family 25 (mitochondrial carrier, brain), member 14	SLC25A14	0,88
protein phosphatase 1, regulatory subunit 3A	PPP1R3A	0,88
lectin, galactoside-binding, soluble, 7; lectin, galactoside-binding, soluble, 7B	LGALS7; LGALS7B	0,88
acyl-CoA thioesterase 2	ACOT2	0,88
DDB1 and CUL4 associated factor 11	DCAF11	0,88
Rab acceptor 1 (prenylated)	RABAC1	0,88
sorting nexin 4	SNX4	0,88
PDZ domain containing 3	PDZD3	0,88
Memczak2013 ANTISENSE, coding, INTERNAL, intronic best transcript NM_001144382	PLCL2	0,88
ral guanine nucleotide dissociation stimulator-like 3	RGL3	0,88

novel transcript; olfactory receptor, family 5, subfamily H, member 8 (gene/pseudogene) [Source:HGNC Symbol;Acc:HGNC:14773]	RP11-325B23.2; OR5H8	0,88
spermatogenesis associated 20	SPATA20	0,88
jumonji domain containing 6	JMJD6	0,88
synuclein alpha	SNCA	0,88
JAZF zinc finger 1	JAZF1	0,88
cyclin F	CCNF	0,88
NK2 homeobox 1	NKX2-1	0,88
LEO1 homolog, Paf1/RNA polymerase II complex component	LEO1	0,88
heterogeneous nuclear ribonucleoprotein C-like 2 [Source:HGNC Symbol;Acc:HGNC:48813]	HNRNPCL2	0,88
hydroxypyruvate isomerase (putative)	HYI	0,88
solute carrier family 35, member F5	SLC35F5	0,88
ankyrin repeat domain 37	ANKRD37	0,88
nucleosome assembly protein 1-like 4	NAP1L4	0,88
Salzman2013 ANTISENSE, coding, INTERNAL, intronic best transcript NM_032968	PCDH11X	0,88
chromosome 16 open reading frame 70	C16orf70	0,88
raftlin, lipid raft linker 1	RFTN1	0,88
SRY box 8	SOX8	0,88
integrin beta 6	ITGB6	0,88
CD68 molecule	CD68	0,88
S-antigen; retina and pineal gland (arrestin)	SAG	0,88
sortilin 1	SORT1	0,88
dendrin	DDN	0,88
KIAA1143 [Source:HGNC Symbol;Acc:HGNC:29198]	KIAA1143	0,88
flap structure-specific endonuclease 1	FEN1	0,88
forkhead box S1	FOXS1	0,88
3-hydroxy-3-methylglutaryl-CoA synthase 2 (mitochondrial)	HMGCS2	0,88
ADAM metallopeptidase domain 33	ADAM33	0,88
ankyrin repeat and sterile alpha motif domain containing 6	ANKS6	0,88
mannosidase, alpha, class 1B, member 1	MAN1B1	0,88
nuclear receptor coactivator 7	NCOA7	0,88
adrenocortical dysplasia homolog (mouse)	ACD	0,88
trafficking protein particle complex 4	TRAPPC4	0,88
family with sequence similarity 89, member B	FAM89B	0,88
oxidoreductase-like domain containing 1	OXLD1	0,88
Transcript Identified by AceView, Entrez Gene ID(s) 91694	LONRF1	0,88
T cell-interacting, activating receptor on myeloid cells 1	TARM1	0,88
metallothionein 1H-like 1	MT1HL1	0,88
signal transducing adaptor molecule (SH3 domain and ITAM motif) 1	STAM	0,88
ring finger protein 150	RNF150	0,88
motile sperm domain containing 1	MOSPD1	0,88

apelin receptor	APLNR	0,88
neural precursor cell expressed, developmentally down-regulated 1	NEDD1	0,88
synapse differentiation inducing 1-like	SYNDIG1L	0,88
argonaute RISC catalytic component 2	ago-02	0,88
Fc receptor-like B	FCRLB	0,88
MANSC domain containing 4	MANSC4	0,88
chloride channel accessory 1	CLCA1	0,88
zinc finger protein 132	ZNF132	0,88
kinesin family member 18A	KIF18A	0,88
zinc finger protein 391	ZNF391	0,88
HAUS augmin like complex subunit 1	HAUS1	0,88
olfactory receptor, family 8, subfamily J, member 1	OR8J1	0,88
KIAA2013	KIAA2013	0,88
heparan sulfate (glucosamine) 3-O-sulfotransferase 2	HS3ST2	0,88
neuroblastoma breakpoint family, member 1	NBPF1	0,88
mannosyl (alpha-1,3-)-glycoprotein beta-1,4-N-acetylglucosaminyltransferase, isozyme A	MGAT4A	0,88
olfactory receptor, family 2, subfamily AP, member 1	OR2AP1	0,88
cysteine rich hydrophobic domain 2	CHIC2	0,88
butyrophilin, subfamily 1, member A1	BTN1A1	0,88
G0/G1 switch 2	G0S2	0,88
Jeck2013 ALT_ACCEPTOR, ALT_DONOR, coding, INTERNAL, intronic best transcript NM_004529	MLLT3	0,88
surfeit 6	SURF6	0,88
ubiquitin conjugating enzyme E2 variant 1	UBE2V1	0,88
Smad nuclear interacting protein 1	SNIP1	0,88
transmembrane protein 51	TMEM51	0,88
olfactory receptor, family 11, subfamily H, member 4	OR11H4	0,88
fatty acyl CoA reductase 1	FAR1	0,88
NOP9 nucleolar protein	NOP9	0,88
coiled-coil domain containing 126	CCDC126	0,88
sphingomyelin phosphodiesterase 1, acid lysosomal	SMPD1	0,88
protein associated with topoisomerase II homolog 1 (yeast)	PATL1	0,88
GS homeobox 2	GSX2	0,88
late cornified envelope 1F	LCE1F	0,88
heat shock 10kDa protein 1	HSPE1	0,88
K(lysine) acetyltransferase 2B	KAT2B	0,88
DEAD (Asp-Glu-Ala-Asp) box polypeptide 18	DDX18	0,88
coagulation factor V (proaccelerin, labile factor)	F5	0,88
RAB27A, member RAS oncogene family	RAB27A	0,88
chromosome 5 open reading frame 15	C5orf15	0,88
afamin	AFM	0,88
chromosome 17 open reading frame 74	C17orf74	0,88
secretoglobin, family 3A, member 2	SCGB3A2	0,88

protocadherin 10	PCDH10	0,88
solute carrier family 1 (glial high affinity glutamate transporter), member 3	SLC1A3	0,88
GTP binding protein 4	GTPBP4	0,88
protein tyrosine phosphatase, receptor type, N polypeptide 2	PTPRN2	0,88
solute carrier family 16 (monocarboxylate transporter), member 1	SLC16A1	0,88
chromosome 9 open reading frame 131	C9orf131	0,88
WW and C2 domain containing 2	WWC2	0,88
parkin RBR E3 ubiquitin protein ligase	PARK2	0,88
actin, alpha, cardiac muscle 1	ACTC1	0,88
zinc finger protein 547; trafficking protein particle complex 2B; trafficking protein particle complex 2	ZNF547; TRAPPC2B; TRAPPC2	0,88
seizure related 6 homolog (mouse)-like 2	SEZ6L2	0,88
long intergenic non-protein coding RNA 1465	LINC01465	0,88
KIAA1429	KIAA1429	0,88
cation channel, sperm associated 3	CATSPER3	0,88
Transcript Identified by AceView, Entrez Gene ID(s) 8615	USO1	0,88
solute carrier family 16, member 6	SLC16A6	0,88
non imprinted in Prader-Willi/Angelman syndrome 2	NIPA2	0,88
hydroxymethylbilane synthase	HMBS	0,88
carcinoembryonic antigen-related cell adhesion molecule 18	CEACAM18	0,88
solute carrier family 39 (zinc transporter), member 13	SLC39A13	0,88
cortexin 1	CTXN1	0,88
chromodomain helicase DNA binding protein 9	CHD9	0,88
nuclear transport factor 2-like export factor 1	NXT1	0,88
leucine rich repeat containing 7	LRRC7	0,88
glucoside xylosyltransferase 2	GXYLT2	0,88
EPPIN-WFDC6 readthrough	EPPIN-WFDC6	0,88
thymosin beta 4, X-linked	TMSB4X	0,88
Jeck2013 ALT_ACCEPTOR, ALT_DONOR, coding, INTERNAL, intronic best transcript NM_001167740	SMYD3	0,88
amelogenin, X-linked	AMELX	0,88
DNA-damage inducible 1 homolog 1	DDI1	0,88
ankyrin repeat domain 27 (VPS9 domain)	ANKRD27	0,88
Rho guanine nucleotide exchange factor (GEF) 40	ARHGEF40	0,88
serine peptidase inhibitor, Kazal type 7 (putative)	SPINK7	0,88
regulator of G-protein signaling 7 binding protein	RGS7BP	0,88
secreted and transmembrane 1	SECTM1	0,88
CD163 molecule-like 1	CD163L1	0,88
POU class 2 associating factor 1	POU2AF1	0,88
tetratricopeptide repeat domain 33	TTC33	0,88
protein kinase, AMP-activated, gamma 2 non-catalytic subunit	PRKAG2	0,88
minichromosome maintenance complex component 6	MCM6	0,88

BCL2-like 14 (apoptosis facilitator)	BCL2L14	0,88
pre-mRNA processing factor 19	PRPF19	0,88
WD repeat domain 48	WDR48	0,88
coiled-coil domain containing 85B	CCDC85B	0,88
dual specificity tyrosine-(Y)-phosphorylation regulated kinase 1B	DYRK1B	0,88
abhydrolase domain containing 17B	ABHD17B	0,88
histone cluster 2, H2aa3; histone cluster 2, H2aa4	HIST2H2AA3; HIST2H2AA4	0,88
chromosome 17 open reading frame 58	C17orf58	0,88
ornithine carbamoyltransferase	OTC	0,88
tubulin, beta class I	TUBB	0,87
protease, serine, 1 (trypsin 1)	PRSS1	0,87
tripartite motif containing 29	TRIM29	0,87
monocyte to macrophage differentiation-associated	MMD	0,87
Janus kinase 1	JAK1	0,87
profilin 2	PFN2	0,87
vanin 1	VNN1	0,87
NIPA-like domain containing 3	NIPAL3	0,87
RCAN family member 3	RCAN3	0,87
tubulin, alpha 3e	TUBA3E	0,87
osteoglycin	OGN	0,87
prolactin regulatory element binding	PREB	0,87
endoplasmic reticulum aminopeptidase 2	ERAP2	0,87
olfactory receptor, family 2, subfamily L, member 2	OR2L2	0,87
nudix hydrolase 17	NUDT17	0,87
mitochondrial ribosomal protein L28	MRPL28	0,87
Transcript Identified by AceView, Entrez Gene ID(s) 131034	CPNE4	0,87
ADAM metallopeptidase with thrombospondin type 1 motif 19	ADAMTS19	0,87
H2A histone family, member B3	H2AFB3	0,87
ankyrin repeat and SOCS box containing 15	ASB15	0,87
calcium/calmodulin-dependent protein kinase II inhibitor 2	CAMK2N2	0,87
C5orf66 antisense RNA 1	C5orf66-AS1	0,87
biogenesis of lysosomal organelles complex-1, subunit 6, pallidin	BLOC1S6	0,87
agrin	AGRN	0,87
solute carrier family 7 (amino acid transporter light chain, y+L system), member 7	SLC7A7	0,87
Memczak2013 ALT_ACCEPTOR, ALT_DONOR, coding, INTERNAL, intronic best transcript NM_139207	NAP1L1	0,87
runt-related transcription factor 2	RUNX2	0,87
zinc finger protein 154	ZNF154	0,87
keratin 23, type I	KRT23	0,87
Transcript Identified by AceView, Entrez Gene ID(s) 93190	C1orf158	0,87
SEC31 homolog B, COPII coat complex component	SEC31B	0,87
zinc finger, MIZ-type containing 2	ZMIZ2	0,87

non-SMC condensin I complex subunit G	NCAPG	0,87
RNA binding motif protein 46	RBM46	0,87
olfactory receptor, family 13, subfamily C, member 7 (gene/pseudogene) [Source:HGNC Symbol;Acc:HGNC:15102]	OR13C7	0,87
casein kinase 1, gamma 2	CSNK1G2	0,87
neuroblastoma breakpoint family, member 10	NBPF10	0,87
mediator complex subunit 10	MED10	0,87
ethanolaminephosphotransferase 1	EPT1	0,87
protein tyrosine phosphatase, receptor type, f polypeptide (PTPRF), interacting protein (liprin), alpha 1	PPFIA1	0,87
aarF domain containing kinase 5	ADCK5	0,87
DEAD (Asp-Glu-Ala-Asp) box helicase 3, X-linked	DDX3X	0,87
WNK lysine deficient protein kinase 1	WNK1	0,87
heparan sulfate (glucosamine) 3-O-sulfotransferase 3B1	HS3ST3B1	0,87
wingless-type MMTV integration site family, member 4	WNT4	0,87
HIRA interacting protein 3	HIRIP3	0,87
Transcript Identified by AceView, Entrez Gene ID(s) 23355	VPS8	0,87
RNA binding motif protein 47	RBM47	0,87
family with sequence similarity 120C	FAM120C	0,87
prion protein (testis specific)	PRNT	0,87
arginine vasopressin receptor 1B	AVPR1B	0,87
SLC2A4 regulator	SLC2A4RG	0,87
transmembrane protein 211	TMEM211	0,87
solute carrier family 36 (proton/amino acid symporter), member 2	SLC36A2	0,87
solute carrier family 28 (concentrative nucleoside transporter), member 1	SLC28A1	0,87
myelin basic protein	MBP	0,87
Zhang2013 ALT_ACCEPTOR, ALT_DONOR, coding, INTERNAL, intronic best transcript NM_133374	ZNF618	0,87
Transcript Identified by AceView, Entrez Gene ID(s) 2146	EZH2	0,87
chromosome 1 open reading frame 101	C1orf101	0,87
zinc finger protein 530	ZNF530	0,87
ORAI calcium release-activated calcium modulator 3	ORAI3	0,87
SEC22 homolog A, vesicle trafficking protein	SEC22A	0,87
scribbled planar cell polarity protein; microRNA 937	SCRIB; MIR937	0,87
transmembrane protein 135	TMEM135	0,87
cullin 4B	CUL4B	0,87
DNA cross-link repair 1A	DCLRE1A	0,87
late endosomal/lysosomal adaptor, MAPK and MTOR activator 5	LAMTOR5	0,87
Transcript Identified by AceView, Entrez Gene ID(s) 90485	ZNF835	0,87
xanthine dehydrogenase	XDH	0,87
pre-B-cell leukemia homeobox 3	PBX3	0,87
SOS Ras/Rho guanine nucleotide exchange factor 2	SOS2	0,87

Memczak2013 ALT_DONOR, coding, INTERNAL, intronic best transcript NM_014739	BCLAF1	0,87
basal body orientation factor 1	BBOF1	0,87
phospholipase C, beta 4	PLCB4	0,87
vacuolar protein sorting 52 homolog (S. cerevisiae)	VPS52	0,87
neuronal regeneration related protein	NREP	0,87
SH3 domain and tetratricopeptide repeats 2	SH3TC2	0,87
von Hippel-Lindau tumor suppressor, E3 ubiquitin protein ligase	VHL	0,87
plastin 1	PLS1	0,87
zinc finger with KRAB and SCAN domains 5	ZKSCAN5	0,87
zinc finger protein 697	ZNF697	0,87
chymotrypsinogen B2	CTRB2	0,87
keratin associated protein 1-5	KRTAP1-5	0,87
hes-related family bHLH transcription factor with YRPW motif 2	HEY2	0,87
kallikrein B1	KLKB1	0,87
glutamate rich 6B	ERICH6B	0,87
adrenoceptor alpha 1A	ADRA1A	0,87
ADP-ribosylarginine hydrolase	ADPRH	0,87
leucine-rich repeats and IQ motif containing 3	LRRIQ3	0,87
centrosomal protein 95kDa	CEP95	0,87
HNF1 homeobox B	HNF1B	0,87
neuron-derived neurotrophic factor	NDNF	0,87
complement factor H-related 2	CFHR2	0,87
distal-less homeobox 4	DLX4	0,87
ring finger and CCCH-type domains 2	RC3H2	0,87
YTH domain containing 2	YTHDC2	0,87
proteasome 26S subunit, ATPase 4 [Source:HGNC Symbol;Acc:HGNC:9551]	PSMC4	0,87
zinc finger protein 705C, pseudogene	ZNF705CP	0,87
phospholipase D1, phosphatidylcholine-specific	PLD1	0,87
carbamoyl-phosphate synthase 1	CPS1	0,87
DEAF1 transcription factor	DEAF1	0,87
NLR family, pyrin domain containing 3	NLRP3	0,87
zinc finger protein 658B, pseudogene	ZNF658B	0,87
short coiled-coil protein	SCOC	0,87
methionyl aminopeptidase 2	METAP2	0,87
oocyte secreted protein 2	OOSP2	0,87
cerebellin 4 precursor	CBLN4	0,87
Transcript Identified by AceView, Entrez Gene ID(s) 26064	RAI14	0,87
signal peptidase complex subunit 1	SPCS1	0,87
plastin 3	PLS3	0,87
death-domain associated protein	DAXX	0,87
Transcript Identified by AceView, Entrez Gene ID(s) 10580	SORBS1	0,87
UDP-Gal:betaGlcNAc beta 1,4- galactosyltransferase, polypeptide 2	B4GALT2	0,87

creatine kinase, brain	CKB	0,87
folylpolyglutamate synthase	FPGS	0,87
calcium/calmodulin-dependent protein kinase I	CAMK1	0,87
ataxin 7-like 1	ATXN7L1	0,87
chromosome 8 open reading frame 37	C8orf37	0,87
RANBP2-like and GRIP domain containing 3	RGPD3	0,87
transmembrane protein 38A	TMEM38A	0,87
solute carrier family 35, member G1	SLC35G1	0,87
Fas ligand (TNF superfamily, member 6)	FASLG	0,87
La ribonucleoprotein domain family, member 4B	LARP4B	0,87
secretion associated, Ras related GTPase 1A	SAR1A	0,87
pleckstrin homology domain containing, family G (with RhoGef domain) member 1	PLEKHG1	0,87
dimethylglycine dehydrogenase	DMGDH	0,87
gonadotropin releasing hormone 2	GNRH2	0,87
optic atrophy 1 (autosomal dominant)	OPA1	0,87
family with sequence similarity 160, member A1	FAM160A1	0,87
olfactory receptor, family 8, subfamily K, member 5	OR8K5	0,87
complement component 4 binding protein, alpha	C4BPA	0,87
tet methylcytosine dioxygenase 3	TET3	0,87
Transcript Identified by AceView, Entrez Gene ID(s) 10915	TCERG1	0,87
retinoic acid early transcript 1L	RAET1L	0,87
stanniocalcin 1	STC1	0,87
COMM domain containing 2	COMMD2	0,87
thrombomodulin	THBD	0,87
amine oxidase, copper containing 3	AOC3	0,87
synuclein alpha interacting protein	SNCAIP	0,87
KRR1, small subunit (SSU) processome component, homolog (yeast)	KRR1	0,87
protease, serine 50	PRSS50	0,87
calcium channel flower domain containing 1	CACFD1	0,87
NADH dehydrogenase (ubiquinone) 1, subcomplex unknown, 1, 6kDa	NDUFC1	0,87
M-phase phosphoprotein 8	MPHOSPH8	0,87
Transcript Identified by AceView, Entrez Gene ID(s) 51011	FAHD2A	0,87
biogenesis of lysosomal organelles complex-1, subunit 5, muted	BLOC1S5	0,87
Cbl proto-oncogene, E3 ubiquitin protein ligase	CBL	0,87
solute carrier family 18 (vesicular monoamine transporter), member 1	SLC18A1	0,87
osteopetrosis associated transmembrane protein 1	OSTM1	0,87
zinc finger protein 660	ZNF660	0,87
protein phosphatase 3, catalytic subunit, gamma isozyme	PPP3CC	0,87
calumenin	CALU	0,87
family with sequence similarity 76, member B	FAM76B	0,87
taxilin gamma	TXLNG	0,87

centrosomal protein 57kDa	CEP57	0,87
trafficking protein particle complex 5	TRAPPC5	0,87
phosphodiesterase 2A, cGMP-stimulated	PDE2A	0,87
cleavage and polyadenylation specific factor 7	CPSF7	0,87
CDC42 binding protein kinase alpha (DMPK-like)	CDC42BPA	0,87
tissue factor pathway inhibitor (lipoprotein-associated coagulation inhibitor)	TFPI	0,87
small nuclear RNA activating complex polypeptide 3	SNAPC3	0,87
profilin 3	PFN3	0,87
mitochondrial ribosomal protein L17	MRPL17	0,87
holocytochrome c synthase	HCCS	0,87
additional sex combs like transcriptional regulator 2	ASXL2	0,87
synovial sarcoma, X breakpoint 4B	SSX4B	0,87
chromodomain protein, Y-linked, 1	CDY1	0,87
family with sequence similarity 120B	FAM120B	0,87
pyruvate dehydrogenase kinase, isozyme 3	PDK3	0,87
UDP glucuronosyltransferase 2 family, polypeptide B15	UGT2B15	0,87
family with sequence similarity 71, member E2	FAM71E2	0,87
centrosomal protein 85kDa-like	CEP85L	0,87
REX4 homolog, 3'-5' exonuclease [Source:HGNC Symbol;Acc:HGNC:12820]	REXO4	0,87
glutamate rich 2	ERICH2	0,87
signal transducer and activator of transcription 2	STAT2	0,87
zinc finger protein 609	ZNF609	0,87
zinc finger protein 486	ZNF486	0,87
Bet1 golgi vesicular membrane trafficking protein-like	BET1L	0,87
chromosome 1 open reading frame 159	C1orf159	0,87
formin 1	FMN1	0,87
olfactory receptor, family 8, subfamily G, member 1 (gene/pseudogene)	OR8G1	0,87
high mobility group box 2	HMGB2	0,87
praja ring finger 2, E3 ubiquitin protein ligase	PJA2	0,87
deoxycytidine kinase	DCK	0,87
bone marrow stromal cell antigen 1	BST1	0,87
ras homolog family member D	RHOD	0,87
zinc finger protein 561	ZNF561	0,87
solute carrier family 52 (riboflavin transporter), member 2	SLC52A2	0,87
family with sequence similarity 47, member E; FAM47E-STBD1 readthrough; starch binding domain 1	FAM47E; FAM47E-STBD1; STBD1	0,87
keratin associated protein 4-9	KRTAP4-9	0,87
achaete-scute family bHLH transcription factor 2	ASCL2	0,87
reticulocalbin 2, EF-hand calcium binding domain	RCN2	0,87
ankyrin repeat and BTB (POZ) domain containing 2	ABTB2	0,87
FYN binding protein	FYB	0,87

S-antigen; retina and pineal gland (arrestin)	SAG	0,87
SMC5-SMC6 complex localization factor 2	SLF2	0,87
essential meiotic structure-specific endonuclease 1	EME1	0,87
shisa family member 7	SHISA7	0,87
C-type lectin domain family 2, member B	CLEC2B	0,87
neuralized E3 ubiquitin protein ligase 4	NEURL4	0,87
transmembrane protein 35	TMEM35	0,87
ZMYM6 neighbor; zinc finger, MYM-type 6	ZMYM6NB; ZMYM6	0,87
BTB and CNC homology 1, basic leucine zipper transcription factor 2	BACH2	0,87
carboxypeptidase A2 (pancreatic)	CPA2	0,87
cyclin G2	CCNG2	0,87
olfactory receptor, family 4, subfamily C, member 11	OR4C11	0,87
inducible T-cell co-stimulator ligand	ICOSLG	0,87
enolase 3 (beta, muscle)	ENO3	0,87
lysosomal protein transmembrane 4 beta	LAPTM4B	0,87
zinc finger protein 263	ZNF263	0,87
kelch-like ECH-associated protein 1	KEAP1	0,87
synaptotagmin IX	SYT9	0,87
SP140 nuclear body protein-like	SP140L	0,87
family with sequence similarity 166, member A	FAM166A	0,87
adhesion G protein-coupled receptor D1	ADGRD1	0,87
inhibitor of kappa light polypeptide gene enhancer in B-cells, kinase gamma	IKBKG	0,87
replication factor C subunit 3	RFC3	0,87
autophagy related 16-like 1	ATG16L1	0,87
Transcript Identified by AceView, Entrez Gene ID(s) 9088	PKMYT1	0,87
inositol-3-phosphate synthase 1	ISYNA1	0,87
phosphatidylinositol glycan anchor biosynthesis class V	PIGV	0,87
dedicator of cytokinesis 8	DOCK8	0,87
transmembrane protein 181	TMEM181	0,87
MDM2 binding protein	MTBP	0,87
BCL2-like 14 (apoptosis facilitator)	BCL2L14	0,87
chromosome 3 open reading frame 20	C3orf20	0,87
gamma-aminobutyric acid (GABA) A receptor, beta 3	GABRB3	0,87
long intergenic non-protein coding RNA 1521	LINC01521	0,87
chromobox homolog 2	CBX2	0,87
atypical chemokine receptor 3	ACKR3	0,87
sperm-tail PG-rich repeat containing 2	STPG2	0,87
MAS1 proto-oncogene, G protein-coupled receptor	MAS1	0,87
Transcript Identified by AceView, Entrez Gene ID(s) 23358	USP24	0,87
olfactory receptor, family 4, subfamily Q, member 2 (gene/pseudogene)	OR4Q2	0,87
fucosyltransferase 11 (alpha (1,3) fucosyltransferase)	FUT11	0,87

adenylate cyclase 6; microRNA 4701	ADCY6; MIR4701	0,87
outer dense fiber of sperm tails 3-like 2	ODF3L2	0,87
coatomer protein complex subunit beta 1	COPB1	0,87
carbamoyl-phosphate synthetase 2, aspartate transcarbamylase, and dihydroorotase	CAD	0,87
cadherin 24, type 2	CDH24	0,87
olfactory receptor, family 13, subfamily C, member 9	OR13C9	0,87
opsin 1 (cone pigments), medium-wave-sensitive	OPN1MW	0,87
EF-hand calcium binding domain 10	EFCAB10	0,87
ankyrin repeat domain 50	ANKRD50	0,87
zinc finger protein 107; microRNA 6839	ZNF107; MIR6839	0,87
carboxypeptidase A5	CPA5	0,87
tudor domain containing 9	TDRD9	0,87
mab-21-like 3 (C. elegans)	MAB21L3	0,87
olfactory receptor, family 8, subfamily J, member 2 (gene/pseudogene)	OR8J2	0,87
zinc finger protein 500	ZNF500	0,87
zinc finger protein 25	ZNF25	0,87
zinc finger protein 430	ZNF430	0,87
5-hydroxytryptamine (serotonin) receptor 4, G protein-coupled	HTR4	0,87
NGFI-A binding protein 2 (EGR1 binding protein 2)	NAB2	0,87
ribosomal protein L7a	RPL7A	0,87
mitogen-activated protein kinase kinase kinase kinase 2	MAP4K2	0,87
NFKB activating protein	NKAP	0,87
biogenesis of lysosomal organelles complex-1, subunit 4, cappuccino	BLOC1S4	0,87
disco-interacting protein 2 homolog C	DIP2C	0,87
olfactory receptor, family 56, subfamily A, member 4	OR56A4	0,87
heterogeneous nuclear ribonucleoprotein C-like 3; heterogeneous nuclear ribonucleoprotein C-like 4	HNRNPCL3; HNRNPCL4	0,87
serpin peptidase inhibitor, clade A (alpha-1 antiproteinase, antitrypsin), member 10	SERPINA10	0,87
sex comb on midleg-like 2 (Drosophila)	SCML2	0,87
butyrophilin, subfamily 3, member A3	BTN3A3	0,87
splicing factor 3b, subunit 1, 155kDa	SF3B1	0,87
3-hydroxybutyrate dehydrogenase, type 1	BDH1	0,87
enoyl-CoA, hydratase/3-hydroxyacyl CoA dehydrogenase	EHHADH	0,87
GTPase activating protein and VPS9 domains 1	GAPVD1	0,87
DEAD (Asp-Glu-Ala-Asp) box polypeptide 60	DDX60	0,87
vav 1 guanine nucleotide exchange factor	VAV1	0,87
Salzman2013 ALT_ACCEPTOR, ALT_DONOR, coding, INTERNAL, intronic best transcript NM_152740	HIBADH	0,87
olfactory receptor, family 7, subfamily A, member 10	OR7A10	0,87

NLR family, pyrin domain containing 10	NLRP10	0,87
Memczak2013 ANTISENSE, coding, INTERNAL, intronic best transcript NM_079423	MYL6	0,87
nuclear receptor subfamily 5, group A, member 1	NR5A1	0,87
cysteine rich hydrophobic domain 1	CHIC1	0,87
endogenous retrovirus group MER34, member 1	ERVMER34-1	0,87
NAD kinase 2, mitochondrial	NADK2	0,87
peroxisomal membrane protein 4	PXMP4	0,87
mahogunin ring finger 1, E3 ubiquitin protein ligase	MGRN1	0,87
kinesin family member 15	KIF15	0,87
FSHD region gene 1 pseudogene	LOC283788; AL592183.1	0,87
cyclin N-terminal domain containing 1	CNTD1	0,87
chromobox homolog 6	CBX6	0,87
diacylglycerol O-acyltransferase 2-like 6	DGAT2L6	0,87
tectorin alpha	TECTA	0,87
aurora kinase A interacting protein 1	AURKAIP1	0,87
ESF1 nucleolar pre-rRNA processing protein homolog	ESF1	0,87
dopamine receptor D5	DRD5	0,87
Transcript Identified by AceView, Entrez Gene ID(s) 254251	LCORL	0,87
K(lysine) acetyltransferase 6B	KAT6B	0,87
centrosomal protein 97kDa	CEP97	0,87
G protein-coupled receptor 89A	GPR89A	0,87
uncharacterized LOC100134391; novel transcript	LOC100134391; CTD-2532D12.4	0,87
v-ral simian leukemia viral oncogene homolog A (ras related)	RALA	0,87
protein kinase, AMP-activated, beta 2 non-catalytic subunit	PRKAB2	0,87
GRAM domain containing 1A	GRAMD1A	0,87
chromosome 1 open reading frame 115	C1orf115	0,87
ATP binding cassette subfamily C member 3	ABCC3	0,87
homeobox D4	HOXD4	0,87
Transcript Identified by AceView, Entrez Gene ID(s) 10580	SORBS1	0,87
Transcript Identified by AceView, Entrez Gene ID(s) 23122	CLASP2	0,87
RecQ mediated genome instability 2	RMI2	0,87
vasodilator-stimulated phosphoprotein	VASP	0,87
cyclin D-type binding-protein 1	CCNDBP1	0,87
creatine kinase, mitochondrial 1A	CKMT1A	0,87
tryptophan hydroxylase 1	TPH1	0,87
SMYD family member 5	SMYD5	0,87
cytochrome P450, family 2, subfamily A, polypeptide 6	CYP2A6	0,87
cell division cycle 25C	CDC25C	0,87
keratocan	KERA	0,87
transmembrane protein 143	TMEM143	0,87
elastin microfibril interfacer 1	EMILIN1	0,87

peptidyl arginine deiminase, type IV	PADI4	0,87
zinc finger and BTB domain containing 16	ZBTB16	0,87
Transcript Identified by AceView, Entrez Gene ID(s) 23378	RRP8	0,87
potassium channel, voltage gated subfamily A regulatory beta subunit 3	KCNAB3	0,87
zinc finger and SCAN domain containing 1	ZSCAN1	0,87
chromosome X open reading frame 49B	CXorf49B	0,87
zinc finger protein 804B	ZNF804B	0,87
chromosome 17 open reading frame 102	C17orf102	0,86
bromodomain adjacent to zinc finger domain 2B	BAZ2B	0,86
hepatitis A virus cellular receptor 2	HAVCR2	0,86
Homo sapiens uncharacterized LOC150527 (TISP43), transcript variant 1, non-coding RNA.; Homo sapiens uncharacterized LOC150527 (TISP43), transcript variant 2, non-coding RNA.; Homo sapiens uncharacterized LOC646743 (LOC646743), non-coding RNA.; Transcript Identified by AceView, Entrez Gene ID(s) 150527; Transcript Identified by AceView, Entrez Gene ID(s) 150527, RefSeq ID(s) NR_027313; uncharacterized LOC150527 [Source:EntrezGene;Acc:150527]	TISP43; LOC646743; LOC150527	0,86
kinesin family member 16B	KIF16B	0,86
phosphodiesterase 4A, cAMP-specific	PDE4A	0,86
FYVE, RhoGEF and PH domain containing 5	FGD5	0,86
ladybird homeobox 1	LBX1	0,86
calcyon neuron-specific vesicular protein	CALY	0,86
nuclear factor of kappa light polypeptide gene enhancer in B-cells inhibitor, delta	NFKBID	0,86
Transcript Identified by AceView, Entrez Gene ID(s) 51555	PEX5L	0,86
transmembrane protein 31	TMEM31	0,86
NEDD4 binding protein 2-like 1	N4BP2L1	0,86
ER degradation enhancer, mannosidase alpha-like 1	EDEM1	0,86
long intergenic non-protein coding RNA 615	LINC00615	0,86
solute carrier family 25 (S-adenosylmethionine carrier), member 26	SLC25A26	0,86
trafficking protein particle complex 13	TRAPPC13	0,86
DNA methyltransferase 1 associated protein 1	DMAP1	0,86
synaptotagmin VII	SYT7	0,86
tripartite motif containing 64B	TRIM64B	0,86
solute carrier family 38, member 8	SLC38A8	0,86
2-phosphoxylose phosphatase 1	PXYLP1	0,86
family with sequence similarity 168, member B	FAM168B	0,86
olfactory receptor, family 2, subfamily J, member 3	OR2J3	0,86
G protein-coupled receptor 18	GPR18	0,86
colorectal cancer associated 1	COLCA1	0,86
adenylate kinase 1	AK1	0,86
zinc finger protein 546	ZNF546	0,86

chromosome 18 open reading frame 25	C18orf25	0,86
ADP-ribosylation factor like GTPase 2 binding protein	ARL2BP	0,86
nuclear GTPase, germinal center associated	NUGGC	0,86
methylmalonyl CoA epimerase	MCEE	0,86
olfactory receptor, family 4, subfamily F, member 15	OR4F15	0,86
endothelial cell surface expressed chemotaxis and apoptosis regulator	ECSCR	0,86
golgi-associated PDZ and coiled-coil motif containing	GOPC	0,86
CSNK1G2 antisense RNA 1	CSNK1G2-AS1	0,86
zinc finger protein 121	ZNF121	0,86
solute carrier family 26 (anion exchanger), member 3	SLC26A3	0,86
tubulin, delta 1	TUBD1	0,86
transmembrane protein 69	TMEM69	0,86
zinc finger protein 780B	ZNF780B	0,86
phospholipase A2, group IID	PLA2G2D	0,86
mitogen-activated protein kinase kinase kinase 2	MAP3K2	0,86
ADP-ribose/CDP-alcohol diphosphatase, manganese-dependent	ADPRM	0,86
reprimo, TP53 dependent G2 arrest mediator candidate	RPRM	0,86
translocase of outer mitochondrial membrane 70 homolog A (S. cerevisiae)	TOMM70A	0,86
chromosome 19 open reading frame 52	C19orf52	0,86
cytotoxic and regulatory T-cell molecule	CRTAM	0,86
arsenite methyltransferase	AS3MT	0,86
cysteine-rich secretory protein 1	CRISP1	0,86
ribosomal RNA processing 1	RRP1	0,86
zinc finger protein 385D	ZNF385D	0,86
retinol binding protein 7, cellular	RBP7	0,86
LLP homolog, long-term synaptic facilitation (Aplysia)	LLPH	0,86
SH2 domain containing 3C	SH2D3C	0,86
capicua transcriptional repressor	CIC	0,86
chromosome 9 open reading frame 64	C9orf64	0,86
protein phosphatase 2, regulatory subunit A, alpha	PPP2R1A	0,86
GTPase, IMAP family member 2	GIMAP2	0,86
transmembrane and tetratricopeptide repeat containing 4	TMTC4	0,86
RAP1B, member of RAS oncogene family	RAP1B	0,86
golgin A8 family, member O	GOLGA8O	0,86
cyclin-dependent kinase inhibitor 2C (p18, inhibits CDK4)	CDKN2C	0,86
3-hydroxyacyl-CoA dehydratase 3	HACD3	0,86
olfactory receptor, family 5, subfamily H, member 1	OR5H1	0,86
leucine rich repeat neuronal 1	LRRN1	0,86
elongator acetyltransferase complex subunit 6	ELP6	0,86
solute carrier family 16 (monocarboxylate transporter), member 7	SLC16A7	0,86
family with sequence similarity 98, member A	FAM98A	0,86
glutamate rich 3	ERICH3	0,86

desmoglein 3	DSG3	0,86
N-acetyltransferase 8 (GCN5-related, putative)	NAT8	0,86
ankyrin repeat domain 66	ANKRD66	0,86
carboxylesterase 3; carboxylesterase 2	CES3; CES2	0,86
chemokine (C-X-C motif) receptor 5	CXCR5	0,86
PRKC, apoptosis, WT1, regulator	PAWR	0,86
clathrin interactor 1	CLINT1	0,86
procollagen-lysine, 2-oxoglutarate 5-dioxygenase 2	PLOD2	0,86
nuclear factor, erythroid 2-like 3	NFE2L3	0,86
nuclear receptor subfamily 1, group H, member 2	NR1H2	0,86
oligodendrocyte transcription factor 1	OLIG1	0,86
cadherin 6, type 2, K-cadherin (fetal kidney)	CDH6	0,86
interleukin 17C	IL17C	0,86
pancreatic progenitor cell differentiation and proliferation factor	PPDPF	0,86
glutathione peroxidase 7	GPX7	0,86
Fc fragment of IgG, receptor, transporter, alpha	FCGRT	0,86
GINS complex subunit 2 (Psf2 homolog)	GINS2	0,86
trypsinogen-like pseudogene	TRY2P	0,86
transmembrane protein 158 (gene/pseudogene)	TMEM158	0,86
olfactory receptor, family 8, subfamily D, member 4	OR8D4	0,86
golgin A6 family, member B	GOLGA6B	0,86
mitogen-activated protein kinase 1	MAPK1	0,86
slit guidance ligand 3	SLIT3	0,86
sorcin	SRI	0,86
chloride intracellular channel 2	CLIC2	0,86
solute carrier family 2 (facilitated glucose transporter), member 1	SLC2A1	0,86
immunoglobulin-like and fibronectin type III domain containing 1	IGFN1	0,86
SET and MYND domain containing 2	SMYD2	0,86
MT-RNR2-like 6	MTRNR2L6	0,86
single immunoglobulin and toll-interleukin 1 receptor (TIR) domain	SIGIRR	0,86
v-ets avian erythroblastosis virus E26 oncogene homolog 2	ETS2	0,86
EH domain containing 2	EHD2	0,86
HECT domain and ankyrin repeat containing E3 ubiquitin protein ligase 1	HACE1	0,86
angiopoietin like 3	ANGPTL3	0,86
zinc finger, SWIM-type containing 2	ZSWIM2	0,86
protocadherin 9	PCDH9	0,86
serine peptidase inhibitor, Kazal type 8 (putative)	SPINK8	0,86
GAS8 antisense RNA 1	GAS8-AS1	0,86
glutamate receptor, ionotropic, delta 2 (Grid2) interacting protein	GRID2IP	0,86

dolichyldiphosphatase 1	DOLPP1	0,86
ubiquinol-cytochrome c reductase complex assembly factor 3	UQCC3	0,86
nuclear receptor subfamily 1, group D, member 2	NR1D2	0,86
RAD54 homolog B (S. cerevisiae); fibrinogen silencer binding protein	RAD54B; FSBP	0,86
solute carrier family 17 (organic anion transporter), member 1	SLC17A1	0,86
wntless Wnt ligand secretion mediator	WLS	0,86
glucan (1,4-alpha-), branching enzyme 1	GBE1	0,86
IKAROS family zinc finger 1	IKZF1	0,86
PRAME family member 10	PRAMEF10	0,86
X-linked Kx blood group related 9	XKR9	0,86
SERPINE1 mRNA binding protein 1	SERBP1	0,86
capping protein (actin filament), gelsolin-like	CAPG	0,86
REST corepressor 3	RCOR3	0,86
oncomodulin 2	OCM2	0,86
chromosome 15 open reading frame 43	C15orf43	0,86
phospholipid phosphatase related 3	PLPPR3	0,86
Pim-1 proto-oncogene, serine/threonine kinase	PIM1	0,86
WW domain containing transcription regulator 1	WWTR1	0,86
lipoma HMGIC fusion partner	LHFP	0,86
MAGE family member B3	MAGEB3	0,86
solute carrier family 36 (proton/amino acid symporter), member 4	SLC36A4	0,86
transmembrane protein 110	TMEM110	0,86
transmembrane protein 237	TMEM237	0,86
metallothionein 4	MT4	0,86
glyoxalase domain containing 4	GLOD4	0,86
cell division cycle associated 5	CDCA5	0,86
glutathione peroxidase 8 (putative)	GPX8	0,86
synaptotagmin XVII	SYT17	0,86
glial cells missing homolog 1 (Drosophila)	GCM1	0,86
CUE domain containing 2	CUEDC2	0,86
neuroligin 4, X-linked	NLGN4X	0,86
sterile alpha motif domain containing 1	SAMD1	0,86
sperm associated antigen 5	SPAG5	0,86
serine/arginine repetitive matrix 4	SRRM4	0,86
choroideremia (Rab escort protein 1)	CHM	0,86
interferon regulatory factor 2 binding protein-like	IRF2BPL	0,86
C-type lectin domain family 12, member A	CLEC12A	0,86
solute carrier family 22 (organic anion transporter), member 9	SLC22A9	0,86
kinesin family member 2C	KIF2C	0,86
ring finger protein 128, E3 ubiquitin protein ligase	RNF128	0,86
zinc finger, DHHC-type containing 3	ZDHHC3	0,86
elongation factor RNA polymerase II	ELL	0,86
WD repeat and SOCS box containing 2	WSB2	0,86

TCDD-inducible poly(ADP-ribose) polymerase	TIPARP	0,86
histone cluster 1, H3e	HIST1H3E	0,86
chaperonin containing TCP1, subunit 4 (delta)	CCT4	0,86
ferritin mitochondrial	FTMT	0,86
endoplasmic reticulum oxidoreductase alpha	ERO1A	0,86
adhesion G protein-coupled receptor G6	ADGRG6	0,86
chromosome 5 open reading frame 34	C5orf34	0,86
protease, serine, 58	PRSS58	0,86
homer scaffolding protein 1	HOMER1	0,86
phosphoglycerate mutase 1 (brain)	PGAM1	0,86
sterile alpha motif and leucine zipper containing kinase AZK; Mitogen-activated protein kinase kinase kinase MLT [Source:UniProtKB/Swiss-Prot;Acc:Q9NYL2]; Salzman2013 ANNOTATED, CDS, coding, INTERNAL, OVCODE, OVERLAPTX, OVEXON best transcript NM_133646; Salzman2013 ANNOTATED, CDS, coding, OVCODE, OVERLAPTX, OVEXON, UTR3 best transcript NM_016653; Salzman2013 ANNOTATED, CDS, coding, OVCODE, OVERLAPTX, OVEXON, UTR3 best transcript NM_133646; Salzman2013 ANNOTATED, CDS, coding, INTERNAL, OVCODE, OVERLAPTX, OVEXON best transcript NM_016653; Transcript Identified by AceView, Entrez Gene ID(s) 51776	ZAK; pk	0,86
transmembrane protein 236	TMEM236	0,86
Transcript Identified by AceView, Entrez Gene ID(s) 51128	SAR1B	0,86
teratocarcinoma-derived growth factor 1	TDGF1	0,86
ST8 alpha-N-acetyl-neuraminide alpha-2,8-sialyltransferase 6	ST8SIA6	0,86
caveolin 1	CAV1	0,86
SMG9 nonsense mediated mRNA decay factor	SMG9	0,86
HEAT repeat containing 3	HEATR3	0,86
echinoderm microtubule associated protein like 5	EML5	0,86
extra spindle pole bodies like 1, separase	ESPL1	0,86
transmembrane protein 121	TMEM121	0,86
retinoblastoma binding protein 9	RBBP9	0,86
Rap guanine nucleotide exchange factor 5	RAPGEF5	0,86
Josephin domain containing 1	JOSD1	0,86
inositol polyphosphate-4-phosphatase type I A	INPP4A	0,86
olfactory receptor, family 51, subfamily A, member 4	OR51A4	0,86
G protein-coupled receptor 176	GPR176	0,86
Fc receptor-like A	FCRLA	0,86
potassium channel tetramerization domain containing 9	KCTD9	0,86
cell division cycle 23	CDC23	0,86
regulatory factor X, 7	RFX7	0,86
epsin 2; EPN2 intronic transcript 1	EPN2; EPN2-IT1	0,86

olfactory receptor, family 10, subfamily A, member 6 (gene/pseudogene)	OR10A6	0,86
ERBB receptor feedback inhibitor 1	ERRFI1	0,86
chromosome 4 open reading frame 3	C4orf3	0,86
olfactory receptor, family 5, subfamily AU, member 1	OR5AU1	0,86
forkhead box D4-like 6	FOXD4L6	0,86
caspase 14	CASP14	0,86
LDL receptor related protein 5	LRP5	0,86
proteasome (prosome, macropain) assembly chaperone 4	PSMG4	0,86
RAS, dexamethasone-induced 1	RASD1	0,86
peptidoglycan recognition protein 1	PGLYRP1	0,86
WD repeat domain 78	WDR78	0,86
aquaporin 12A	AQP12A	0,86
solute carrier family 41 (magnesium transporter), member 1	SLC41A1	0,86
5-nucleotidase, cytosolic IB; NT5C1B-RDH14 readthrough; retinol dehydrogenase 14 (all-trans/9-cis/11-cis)	NT5C1B; NT5C1B-RDH14; RDH14	0,86
polymerase (RNA) II (DNA directed) polypeptide H	POLR2H	0,86
D-amino-acid oxidase	DAO	0,86
protein tyrosine phosphatase, receptor-type, Z polypeptide 1	PTPRZ1	0,86
Transcript Identified by AceView, Entrez Gene ID(s) 8438	RAD54L	0,86
guanylate cyclase activator 1C	GUCA1C	0,86
cathepsin V	CTSV	0,86
impact RWD domain protein	IMPACT	0,86
zinc finger, DHHC-type containing 24	ZDHHC24	0,86
S1 RNA binding domain 1	SRBD1	0,86
Zic family member 3	ZIC3	0,86
leucine rich repeat containing 3	LRRC3	0,86
occludin/ELL domain containing 1	OCEL1	0,86
keratin associated protein 10-3	KRTAP10-3	0,86
solute carrier family 16 (monocarboxylate transporter), member 1	SLC16A1	0,86
leucine-rich repeat kinase 1	LRRK1	0,86
phosphatidylinositol glycan anchor biosynthesis class A	PIGA	0,86
KIAA1211-like	KIAA1211L	0,86
follistatin like 1; microRNA 198	FSTL1; MIR198	0,86
submaxillary gland androgen regulated protein 3B	SMR3B	0,86
paired related homeobox 2	PRRX2	0,86
V-set and transmembrane domain containing 1	VSTM1	0,86
zinc finger protein 277	ZNF277	0,86
natural cytotoxicity triggering receptor 1	NCR1	0,86
toll-like receptor 1	TLR1	0,86
chromosome 19 open reading frame 38	C19orf38	0,86
Memczak2013 ALT_ACCEPTOR, ALT_DONOR, coding, INTERNAL, intronic best transcript NM_002919	RFX3	0,86

zyxin	ZYX	0,86
protein phosphatase 1, regulatory subunit 37	PPP1R37	0,86
serine/threonine kinase 35	STK35	0,86
guanidinoacetate N-methyltransferase	GAMT	0,86
selenoprotein W, 1	SEPW1	0,86
cyclin-dependent kinase 1	CDK1	0,86
Fas apoptotic inhibitory molecule 2	FAIM2	0,86
ethanolamine-phosphate phospho-lyase	ETNPPL	0,86
Memczak2013 ANTISENSE, coding, INTERNAL, intronic best transcript NM_016463	CXXC5	0,86
zinc finger protein 682	ZNF682	0,86
poly(ADP-ribose) polymerase family member 3	PARP3	0,86
MON2 homolog, regulator of endosome-to-Golgi trafficking	MON2	0,86
adhesion G protein-coupled receptor B2	ADGRB2	0,86
CAS1 domain containing 1	CASD1	0,86
dual specificity phosphatase 16	DUSP16	0,86
ubiquitin protein ligase E3C	UBE3C	0,86
v-maf avian musculoaponeurotic fibrosarcoma oncogene homolog	MAF	0,86
interferon, lambda 2	IFNL2	0,86
RAD9 checkpoint clamp component A	RAD9A	0,86
phosphatidylinositol glycan anchor biosynthesis class Z	PIGZ	0,86
protease, serine 35	PRSS35	0,86
BCL2-associated transcription factor 1	BCLAF1	0,86
caspase 9	CASP9	0,86
TP53 regulated inhibitor of apoptosis 1	TRIAP1	0,86
MKL/myocardin-like 2; TVP23C pseudogene 2	MKL2; TVP23CP2	0,86
SIVA1, apoptosis-inducing factor	SIVA1	0,86
hexamethylene bis-acetamide inducible 1	HEXIM1	0,86
PR domain containing 15	PRDM15	0,86
CD226 molecule	CD226	0,86
coiled-coil domain containing 155	CCDC155	0,86
zinc finger protein 546	ZNF546	0,86
thioredoxin domain containing 11	TXNDC11	0,86
schlafen family member 12	SLFN12	0,86
homeobox A13	HOXA13	0,86
sperm specific antigen 2	SSFA2	0,86
olfactory receptor, family 51, subfamily F, member 1 (gene/pseudogene)	OR51F1	0,86
purinergic receptor P2Y, G-protein coupled, 2	P2RY2	0,86
diacylglycerol kinase, delta 130kDa	DGKD	0,86
histone cluster 1, H4c	HIST1H4C	0,86
guanine nucleotide binding protein (G protein), alpha 11 (Gq class)	GNA11	0,86

centromere protein I	CENPI	0,86
chromosome 16 open reading frame 58	C16orf58	0,86
keratin associated protein 9-1	KRTAP9-1	0,86
chromosome 17 open reading frame 75	C17orf75	0,86
apolipoprotein B mRNA editing enzyme, catalytic polypeptide-like 3F	APOBEC3F	0,86
zinc finger protein 714; vomeronasal 1 receptor 81 pseudogene	ZNF714; VN1R81P	0,86
UBX domain protein 7	UBXN7	0,86
Memczak2013 ANTISENSE, CDS, coding, INTERNAL best transcript NM_004396	DDX5	0,86
Transcript Identified by AceView, Entrez Gene ID(s) 100288237; 7700	ZNF141	0,86
histone cluster 1, H2bm	HIST1H2BM	0,86
ligand dependent nuclear receptor interacting factor 1	LRIF1	0,86
phospholipase B domain containing 1	PLBD1	0,85
breast cancer anti-estrogen resistance 1	BCAR1	0,85
GC-rich sequence DNA-binding factor 2	GCFC2	0,85
erbb2 interacting protein	ERBB2IP	0,85
laminin, gamma 2	LAMC2	0,85
tenascin C	TNC	0,85
SERTA domain containing 1	SERTAD1	0,85
pleckstrin homology domain containing, family A (phosphoinositide binding specific) member 8	PLEKHA8	0,85
elongator acetyltransferase complex subunit 2	ELP2	0,85
zinc finger protein 580; zinc finger protein 581	ZNF580; ZNF581	0,85
RAB41, member RAS oncogene family	RAB41	0,85
transmembrane protein 231	TMEM231	0,85
glycophorin B (MNS blood group)	GYPB	0,85
dual specificity tyrosine-(Y)-phosphorylation regulated kinase 1B	DYRK1B	0,85
general transcription factor IIH subunit 3	GTF2H3	0,85
RAD50 interactor 1	RINT1	0,85
Bardet-Biedl syndrome 7	BBS7	0,85
solute carrier family 25 (mitochondrial carrier; oxoglutarate carrier), member 11	SLC25A11	0,85
polycystic kidney disease 2-like 2	PKD2L2	0,85
RUN and SH3 domain containing 2	RUSC2	0,85
VCP-interacting membrane selenoprotein	VIMP	0,85
protein kinase C and casein kinase substrate in neurons 1	PACSIN1	0,85
MACRO domain containing 1	MACROD1	0,85
myosin XVB	MYO15B	0,85
matrix metallopeptidase 11	MMP11	0,85
claudin 10	CLDN10	0,85
DEAH (Asp-Glu-Ala-His) box polypeptide 33	DHX33	0,85

C2 calcium-dependent domain containing 4B	C2CD4B	0,85
Transcript Identified by AceView, Entrez Gene ID(s) 3988	LIPA	0,85
cilia and flagella associated protein 47	CFAP47	0,85
X-linked Kx blood group related 3	XKR3	0,85
TYRO protein tyrosine kinase binding protein	TYROBP	0,85
Sp4 transcription factor	SP4	0,85
SERTA domain containing 4	SERTAD4	0,85
KIAA0101; casein kinase 1, gamma 1	KIAA0101; CSNK1G1	0,85
crooked neck pre-mRNA splicing factor 1	CRNKL1	0,85
heat shock 70kDa protein 6 (HSP70B)	HSPA6	0,85
UDP glucuronosyltransferase 2 family, polypeptide B17	UGT2B17	0,85
RAN binding protein 6	RANBP6	0,85
ubiquitin specific peptidase 17-like family member 21	USP17L21	0,85
histone cluster 2, H2aa4; histone cluster 2, H2aa3	HIST2H2AA4; HIST2H2AA3	0,85
fibroblast growth factor 3	FGF3	0,85
proline-serine-threonine phosphatase interacting protein 1	PSTPIP1	0,85
pepsinogen 3, group I (pepsinogen A); pepsinogen 4, group I (pepsinogen A)	PGA3; PGA4	0,85
cathelicidin antimicrobial peptide	CAMP	0,85
RAB30, member RAS oncogene family	RAB30	0,85
solute carrier family 25 (S-adenosylmethionine carrier), member 26	SLC25A26	0,85
transmembrane protein 251	TMEM251	0,85
TBC1 domain family, member 4	TBC1D4	0,85
Transcript Identified by AceView, Entrez Gene ID(s) 1785	DNM2	0,85
Xg blood group; Xg pseudogene, Y-linked 2	XG; XGY2	0,85
olfactory receptor, family 4, subfamily S, member 1	OR4S1	0,85
ADP-ribosylation factor like GTPase 8B	ARL8B	0,85
olfactory receptor, family 4, subfamily F, member 21	OR4F21	0,85
Transcript Identified by AceView, Entrez Gene ID(s) 3082	HGF	0,85
ELK1, member of ETS oncogene family	ELK1	0,85
glutamate receptor, metabotropic 3	GRM3	0,85
ATR interacting protein; three prime repair exonuclease 1	ATRIP; TREX1	0,85

Homo sapiens TBC1 domain family, member 3H (TBC1D3H), mRNA.; Homo sapiens TBC1 domain family, member 3L (TBC1D3L), mRNA.; Homo sapiens TBC1 domain family, member 3I (TBC1D3I), mRNA.; Homo sapiens TBC1 domain family member-like (LOC101060389), mRNA.; TBC1 domain family member-like; Homo sapiens TBC1 domain family, member 3C, mRNA (cDNA clone MGC:87891 IMAGE:5165385), complete cds.; Homo sapiens TBC1 domain family, member 3, mRNA (cDNA clone MGC:87893 IMAGE:5742850), complete cds.; TBC1 domain family, member 3D [Source:HGNC Symbol;Acc:HGNC:28944]	TBC1D3H; TBC1D3L; TBC1D3I; LOC101060389; TBC1D3C; TBC1D3; TBC1D3D	0,85
promyelocytic leukemia	PML	0,85
ubiquitin specific peptidase 42	USP42	0,85
StAR-related lipid transfer domain containing 4	STARD4	0,85
SPANX family, member N4	SPANXN4	0,85
NME/NM23 family member 5	NME5	0,85
purine-rich element binding protein G	PURG	0,85
solute carrier family 37 (glucose-6-phosphate transporter), member 1	SLC37A1	0,85
ventral anterior homeobox 2	VAX2	0,85
neurogenin 3	NEUROG3	0,85
coiled-coil domain containing 153	CCDC153	0,85
PSMB8 antisense RNA 1 (head to head)	PSMB8-AS1	0,85
neurotensin	NTS	0,85
cannabinoid receptor interacting protein 1	CNRIP1	0,85
Memczak2013 ANTISENSE, CDS, coding, INTERNAL best transcript NM_025268	TMEM121	0,85
adhesion G protein-coupled receptor F4	ADGRF4	0,85
triokinase/FMN cyclase	TKFC	0,85
GDNF family receptor alpha like	GFRAL	0,85
t-SNARE domain containing 1	TSNARE1	0,85
adhesion G protein-coupled receptor L3	ADGRL3	0,85
eukaryotic translation initiation factor 5A2	EIF5A2	0,85
urocortin 3	UCN3	0,85
peptidylprolyl isomerase (cyclophilin)-like 4	PPIL4	0,85
dynein, axonemal, heavy chain 8	DNAH8	0,85
zinc finger and SCAN domain containing 9	ZSCAN9	0,85
regulator of chromosome condensation 1; small nucleolar RNA, H/ACA box 73A; small nucleolar RNA host gene 3; small nucleolar RNA, H/ACA box 73B	RCC1; SNORA73A; SNHG3; SNORA73B	0,85
Jeck2013 ALT_ACCEPTOR, ALT_DONOR, coding, INTERNAL, intronic best transcript NM_001080396	FAM155A	0,85
derlin 2	DERL2	0,85
chromosome 10 open reading frame 131	C10orf131	0,85

desmocollin 1	DSC1	0,85
IQ motif containing with AAA domain 1	IQCA1	0,85
keratin associated protein 11-1	KRTAP11-1	0,85
chibby homolog 1 (Drosophila)	CBY1	0,85
transmembrane protein 82	TMEM82	0,85
sideroflexin 1	SFXN1	0,85
basic helix-loop-helix domain containing, class B, 9	BHLHB9	0,85
FAD-dependent oxidoreductase domain containing 2	FOXRED2	0,85
serine peptidase inhibitor, Kunitz type 4	SPINT4	0,85
dual specificity phosphatase 7	DUSP7	0,85
kelch repeat and BTB (POZ) domain containing 7	KBTBD7	0,85
collagen, type IX, alpha 3	COL9A3	0,85
nucleoporin 43kDa	NUP43	0,85
zinc finger protein 655	ZNF655	0,85
phosphate cytidylyltransferase 1, choline, beta	PCYT1B	0,85
chromosome 6 open reading frame 47	C6orf47	0,85
Memczak2013 ANTISENSE, CDS, coding, INTERNAL best transcript NM_014959; Transcript Identified by AceView; novel transcript, antisense to CARD8	CTC-453G23.8; CARD8; nekera	0,85
Memczak2013 ANTISENSE, CDS, coding, INTERNAL, UTR3 best transcript NM_004308	ARHGAP1	0,85
Jeck2013 ANTISENSE, coding, INTERNAL, intronic best transcript NM_001130100	KIFC3	0,85
protamine 2	PRM2	0,85
regulator of G-protein signaling 18	RGS18	0,85
mannosidase, alpha, class 1A, member 1	MAN1A1	0,85
MOB family member 4, phocein	MOB4	0,85
male-specific lethal 3 homolog (Drosophila)	MSL3	0,85
ADP-ribosylation factor like GTPase 5B	ARL5B	0,85
solute carrier family 13 (sodium-dependent citrate transporter), member 5	SLC13A5	0,85
glycerol-3-phosphate dehydrogenase 2	GPD2	0,85
low density lipoprotein receptor class A domain containing 3	LDLRAD3	0,85
Transcript Identified by AceView, Entrez Gene ID(s) 3670	ISL1	0,85
cut-like homeobox 2	CUX2	0,85
zinc finger and BTB domain containing 9	ZBTB9	0,85
UTP23, small subunit (SSU) processome component, homolog (yeast)	UTP23	0,85
Ras and Rab interactor 1	RIN1	0,85
ribosomal protein S6 kinase, 90kDa, polypeptide 1	RPS6KA1	0,85
sterile alpha motif domain containing 9	SAMD9	0,85
RAS p21 protein activator 4B; RAS p21 protein activator 4	RASA4B; RASA4	0,85
thyroid hormone receptor interactor 4	TRIP4	0,85
taste receptor, type 2, member 46	TAS2R46	0,85

DMRT-like family A1	DMRTA1	0,85
interleukin 18 receptor 1	IL18R1	0,85
heat-responsive protein 12	HRSP12	0,85
G protein-coupled receptor 157	GPR157	0,85
lemur tyrosine kinase 2	LMTK2	0,85
proline-rich coiled-coil 2A	PRRC2A	0,85
MORN repeat containing 5	MORN5	0,85
transmembrane protein 43	TMEM43	0,85
leukocyte immunoglobulin-like receptor, subfamily B (with TM and ITIM domains), member 4	LILRB4	0,85
paternally expressed 10	PEG10	0,85
pregnancy up-regulated nonubiquitous CaM kinase	PNCK	0,85
zinc finger protein 316	ZNF316	0,85
torsin family 1, member A (torsin A)	TOR1A	0,85
chromosome 7 open reading frame 26	C7orf26	0,85
cell division cycle 27	CDC27	0,85
Memczak2013 ALT_ACCEPTOR, ALT_DONOR, coding, INTERNAL, intronic best transcript NM_001145114	RRP12	0,85
hepatocellular carcinoma, down-regulated 1	HEPN1	0,85
NK1 homeobox 2	NKX1-2	0,85
adhesion G protein-coupled receptor F1	ADGRF1	0,85
retinoblastoma 1	RB1	0,85
kin of IRRE like (Drosophila)	KIRREL	0,85
zinc finger protein 35	ZNF35	0,85
TruB pseudouridine (psi) synthase family member 1	TRUB1	0,85
hepatoma derived growth factor-like 1	HDGFL1	0,85
growth factor receptor bound protein 7	GRB7	0,85
hepatic leukemia factor	HLF	0,85
retinitis pigmentosa 1 (autosomal dominant)	RP1	0,85
Fanconi anemia complementation group A	FANCA	0,85
ELOVL fatty acid elongase 4	ELOVL4	0,85
claudin 24	CLDN24	0,85
CD164 molecule, sialomucin	CD164	0,85
fascin actin-bundling protein 2, retinal	FSCN2	0,85
acyl-CoA wax alcohol acyltransferase 2	AWAT2	0,85
BCL2-like 14 (apoptosis facilitator)	BCL2L14	0,85
T cell receptor associated transmembrane adaptor 1	TRAT1	0,85
TBC1 domain family, member 21	TBC1D21	0,85
coiled-coil domain containing 42	CCDC42	0,85
family with sequence similarity 9, member B	FAM9B	0,85
anti-silencing function 1B histone chaperone	ASF1B	0,85
actin, beta	ACTB	0,85
chromosome 16 open reading frame 47	C16orf47	0,85
BORCS7-ASMT readthrough (NMD candidate)	BORCS7-ASMT	0,85
chemokine (C-C motif) ligand 13	CCL13	0,85

follistatin-like 4	FSTL4	0,85
F-box and WD repeat domain containing 10	FBXW10	0,85
ral guanine nucleotide dissociation stimulator	RALGDS	0,85
serine/arginine-rich splicing factor 2; microRNA 636	SRSF2; MIR636	0,85
uncharacterized LOC283731; novel transcript	LOC283731; RP11-247C2.2	0,85
chromosome 5 open reading frame 46	C5orf46	0,85
Rho family GTPase 1	RND1	0,85
poly(rC) binding protein 3	PCBP3	0,85
kinesin family member 4B	KIF4B	0,85
ring finger protein 17	RNF17	0,85
mannosidase, alpha, class 2A, member 1	MAN2A1	0,85
ribonuclease H2, subunit A	RNASEH2A	0,85
RecQ helicase-like	RECQL	0,85
enhancer of mRNA decapping 4	EDC4	0,85
Transcript Identified by AceView, Entrez Gene ID(s) 4674	NAP1L2	0,85
sema domain, immunoglobulin domain (Ig), transmembrane domain (TM) and short cytoplasmic domain, (semaphorin) 4G	SEMA4G	0,85
ubiquitin specific peptidase 32	USP32	0,85
clustered mitochondria (cluA/CLU1) homolog	CLUH	0,85
G protein-coupled receptor 137C	GPR137C	0,85
solute carrier family 3 (amino acid transporter heavy chain), member 1	SLC3A1	0,85
coiled-coil domain containing 18	CCDC18	0,85
azurocidin 1	AZU1	0,85
chromatin assembly factor 1, subunit B (p60)	CHAF1B	0,85
zinc finger protein 546	ZNF546	0,85
tachykinin receptor 1	TACR1	0,85
chromosome 4 open reading frame 50	C4orf50	0,85
regulator of G-protein signaling 5	RGS5	0,85
tubulin tyrosine ligase-like family member 11	TTLL11	0,85
myc target 1	MYCT1	0,85
plexin B2	PLXNB2	0,85
muscleblind like splicing regulator 1	MBNL1	0,85
cyclin D1	CCND1	0,85
coiled-coil domain containing 107	CCDC107	0,85
acidic nuclear phosphoprotein 32 family member C	ANP32C	0,85
FAM83H antisense RNA 1 (head to head)	FAM83H-AS1	0,85
cyclin A2	CCNA2	0,85
zinc finger and BTB domain containing 7A	ZBTB7A	0,85
major histocompatibility complex, class I, L (pseudogene)	HLA-L	0,85
olfactory receptor, family 56, subfamily A, member 5	OR56A5	0,85
FAT atypical cadherin 2	FAT2	0,85
neurocan	NCAN	0,85
tumor protein p63 regulated 1	TPRG1	0,85

galactosylceramidase	GALC	0,85
crystallin beta B3	CRYBB3	0,85
transmembrane channel like 1	TMC1	0,85
family with sequence similarity 19 (chemokine (C-C motif)-like), member A2	FAM19A2	0,85
keratin associated protein 19-6	KRTAP19-6	0,85
essential meiotic structure-specific endonuclease subunit 2	EME2	0,85
copper chaperone for superoxide dismutase	CCS	0,85
harakiri, BCL2 interacting protein	HRK	0,85
NDRG family member 2; microRNA 6717	NDRG2; MIR6717	0,85
calcium modulating ligand	CAMLG	0,85
B9 protein domain 1	B9D1	0,85
BPI fold containing family B, member 2	BPIFB2	0,85
carboxylesterase 1	CES1	0,85
zinc finger, RAN-binding domain containing 1	ZRANB1	0,85
glutamate receptor, metabotropic 1	GRM1	0,85
TGF-beta activated kinase 1/MAP3K7 binding protein 2	TAB2	0,85
ret finger protein-like 4A-like 1	RFPL4AL1	0,85
cytochrome P450, family 27, subfamily B, polypeptide 1	CYP27B1	0,85
ubiquitin-fold modifier 1	UFM1	0,85
adhesion G protein-coupled receptor E1	ADGRE1	0,85
solute carrier family 25 (S-adenosylmethionine carrier), member 26	SLC25A26	0,85
POTE ankyrin domain family, member E	POTEE	0,85
crumbs family member 2	CRB2	0,85
ubiquinol-cytochrome c reductase hinge protein like	UQCRHL	0,85
endogenous retrovirus group 48, member 1	ERVH48-1	0,85
solute carrier family 9, subfamily A (NHE3, cation proton antiporter 3), member 3 regulator 2	SLC9A3R2	0,85
protein serine kinase H1	PSKH1	0,85
aldehyde dehydrogenase 3 family, member B2	ALDH3B2	0,85
Opa interacting protein 5	OIP5	0,85
leucine rich repeat containing 4	LRRC4	0,85
family with sequence similarity 160, member B2	FAM160B2	0,85
recombination signal binding protein for immunoglobulin kappa J region	RBPJ	0,85
phospholipid phosphatase related 1	PLPPR1	0,85
GTF2I repeat domain containing 2B	GTF2IRD2B	0,85
CCCTC-binding factor (zinc finger protein)-like	CTCFL	0,85
Jeck2013 ANTISENSE, CDS, coding, INTERNAL, intronic, OVCODE, OVEXON best transcript NM_012335	MYO1F	0,85
KIF1 binding protein	KIF1BP	0,84
ALG10B, alpha-1,2-glucosyltransferase	ALG10B	0,84
cold shock domain containing C2, RNA binding	CSDC2	0,84

synaptic vesicle glycoprotein 2C	SV2C	0,84
patatin-like phospholipase domain containing 2	PNPLA2	0,84
Memczak2013 ANTISENSE, CDS, coding, INTERNAL, intronic best transcript NM_139235	NOL6	0,84
SCY1-like, kinase-like 1	SCYL1	0,84
sphingosine kinase 2	SPHK2	0,84
olfactory receptor, family 10, subfamily K, member 2	OR10K2	0,84
isoprenoid synthase domain containing	ISPD	0,84
sosondowah ankyrin repeat domain family member A	SOWAHA	0,84
myosin ID	MYO1D	0,84
collagen, type XXVI, alpha 1	COL26A1	0,84
T-box 6	TBX6	0,84
coiled-coil domain containing 117	CCDC117	0,84
doublecortin	DCX	0,84
age-related maculopathy susceptibility 2	ARMS2	0,84
C1q and tumor necrosis factor related protein 6	C1QTNF6	0,84
peptidyl arginine deiminase, type IV	PADI4	0,84
platelet derived growth factor C	PDGFC	0,84
kinesin family member 15	KIF15	0,84
gamma-aminobutyric acid (GABA) A receptor, beta 1	GABRB1	0,84
NADH dehydrogenase (ubiquinone) complex I, assembly factor 6	NDUFAF6	0,84
SAM and SH3 domain containing 3	SASH3	0,84
G protein-coupled receptor 19	GPR19	0,84
family with sequence similarity 234, member B	FAM234B	0,84
family with sequence similarity 135, member A	FAM135A	0,84
zinc finger protein 891	ZNF891	0,84
histone cluster 2, H2bf	HIST2H2BF	0,84
platelet-activating factor acetylhydrolase 1b, regulatory subunit 1 (45kDa)	PAFAH1B1	0,84
tribbles pseudokinase 1	TRIB1	0,84
	PRAMEF9	0,84
mitochondrial ribosomal protein S33	MRPS33	0,84
lysosomal-associated membrane protein family, member 5	LAMP5	0,84
TATA box binding protein associated factor 3	TAF3	0,84
limb development membrane protein 1	LMBR1	0,84
Sad1 and UNC84 domain containing 2	SUN2	0,84
otoraplin	OTOR	0,84
Transcript Identified by AceView, Entrez Gene ID(s) 127435	PODN	0,84
arachidonate 5-lipoxygenase-activating protein	ALOX5AP	0,84
selectin E	SELE	0,84
serpin peptidase inhibitor, clade B (ovalbumin), member 8	SERPINB8	0,84
caspase activity and apoptosis inhibitor 1	CAAP1	0,84
NOC3-like DNA replication regulator	NOC3L	0,84
purinergic receptor P2Y, G-protein coupled, 12	P2RY12	0,84
leucine rich repeat containing 34	LRRC34	0,84

EPH receptor A2	EPHA2	0,84
thiosulfate sulfurtransferase (rhodanese)	TST	0,84
FCH domain only 2	FCHO2	0,84
cAMP-regulated phosphoprotein 19kDa	ARPP19	0,84
xyloside xylosyltransferase 1	XXYLT1	0,84
vasorin	VASN	0,84
cullin-associated and neddylation-dissociated 1	CAND1	0,84
sirtuin 6	SIRT6	0,84
otogelin	OTOG	0,84
chromosome 14 open reading frame 132	C14orf132	0,84
homeobox A11	HOXA11	0,84
high mobility group 20B	HMG20B	0,84
component of oligomeric golgi complex 4	COG4	0,84
microtubule associated tumor suppressor 1	MTUS1	0,84
zinc finger protein, FOG family member 2	ZFPM2	0,84
RAB11A, member RAS oncogene family	RAB11A	0,84
solute carrier family 2 (facilitated glucose transporter), member 9	SLC2A9	0,84
trinucleotide repeat containing 6C	TNRC6C	0,84
D-dopachrome tautomerase	DDT	0,84
Zhang2013 ALT_DONOR, coding, INTERNAL, intronic best transcript NM_007184	NISCH	0,84
cholinergic receptor, nicotinic alpha 2	CHRNA2	0,84
transmembrane protein 26	TMEM26	0,84
polypyrimidine tract binding protein 3	PTBP3	0,84
RIO kinase 3	RIOK3	0,84
Transcript Identified by AceView, Entrez Gene ID(s) 23040	MYT1L	0,84
apolipoprotein B receptor	APOBR	0,84
zinc finger protein 667	ZNF667	0,84
N-deacetylase/N-sulfotransferase (heparan glucosaminyl) 4	NDST4	0,84
zinc finger protein 513	ZNF513	0,84
myoregulin	MRLN	0,84
Memczak2013 ANTISENSE, coding, INTERNAL, intronic best transcript NM_014371; TEC	AC006128.2; AKAP8L	0,84
sex hormone-binding globulin	SHBG	0,84
sterol-C5-desaturase	SC5D	0,84
poly(A)-specific ribonuclease (PARN)-like domain containing 1	PNLDC1	0,84
cytochrome P450, family 26, subfamily C, polypeptide 1	CYP26C1	0,84
SIN3 transcription regulator family member B	SIN3B	0,84
zinc finger protein 830	ZNF830	0,84
psoriasis susceptibility 1 candidate 1	PSORS1C1	0,84
SDS3 homolog, SIN3A corepressor complex component	SUDS3	0,84
exocyst complex component 4	EXOC4	0,84

olfactory receptor, family 4, subfamily N, member 4; olfactory receptor, family 4, subfamily N, member 3 pseudogene; olfactory receptor, family 4, subfamily M, member 2	OR4N4; OR4N3P; OR4M2	0,84
Jeck2013 ANTISENSE, coding, INTERNAL, intronic best transcript NM_022765	MICAL1	0,84
solute carrier family 50 (sugar efflux transporter), member 1	SLC50A1	0,84
MGAT4 family, member C	MGAT4C	0,84
galactokinase 1	GALK1	0,84
BPI fold containing family B, member 1	BPIFB1	0,84
serine dehydratase	SDS	0,84
anoctamin 1, calcium activated chloride channel	ANO1	0,84
1-acylglycerol-3-phosphate O-acyltransferase 3	AGPAT3	0,84
kelch domain containing 8B	KLHDC8B	0,84
NACC family member 2, BEN and BTB (POZ) domain containing	NACC2	0,84
insulin like growth factor binding protein 3	IGFBP3	0,84
proteasome 26S subunit, non-ATPase 10	PSMD10	0,84
zinc finger protein 473	ZNF473	0,84
ABHD14A-ACY1 readthrough	ABHD14A-ACY1	0,84
TP53 target 3B	TP53TG3B	0,84
Homo sapiens TP53 target 3B (TP53TG3B), transcript variant 1, mRNA.; Homo sapiens TP53 target 3C (TP53TG3C), transcript variant 1, mRNA.; Homo sapiens TP53 target 3 (TP53TG3), transcript variant 1, mRNA.; Homo sapiens TP53 target 3 (TP53TG3), transcript variant 3, non-coding RNA.; Homo sapiens TP53 target 3B (TP53TG3B), transcript variant 3, non-coding RNA.; Homo sapiens TP53 target 3 (TP53TG3), transcript variant 4, non-coding RNA.; Homo sapiens TP53 target 3C (TP53TG3C), transcript variant 3, non-coding RNA.; TP53-target gene 3 protein; Homo sapiens TP53 target 3, mRNA (cDNA clone MGC:119889 IMAGE:40015196), complete cds.; Homo sapiens TP53 target 3, mRNA (cDNA clone MGC:119888 IMAGE:40015195), complete cds.; TP53-target gene 3 protein [Source:UniProtKB/Swiss-Prot;Acc:Q9ULZ0]	TP53TG3B; TP53TG3C; TP53TG3; LOC102723655; RP11-23E10.6	0,84

Homo sapiens TP53 target 3B (TP53TG3B), transcript variant 1, mRNA.; Homo sapiens TP53 target 3C (TP53TG3C), transcript variant 1, mRNA.; Homo sapiens TP53 target 3 (TP53TG3), transcript variant 1, mRNA.; Homo sapiens TP53 target 3 (TP53TG3), transcript variant 3, non-coding RNA.; Homo sapiens TP53 target 3B (TP53TG3B), transcript variant 3, non-coding RNA.; Homo sapiens TP53 target 3 (TP53TG3), transcript variant 4, non-coding RNA.; Homo sapiens TP53 target 3C (TP53TG3C), transcript variant 3, non-coding RNA.; TP53-target gene 3 protein; Homo sapiens TP53 target 3, mRNA (cDNA clone MGC:119889 IMAGE:40015196), complete cds.; Homo sapiens TP53 target 3, mRNA (cDNA clone MGC:119888 IMAGE:40015195), complete cds.; Transcript Identified by AceView; TP53-target gene 3 protein [Source:UniProtKB/Swiss-Prot;Acc:Q9ULZ0]	TP53TG3B; TP53TG3C; TP53TG3; LOC102723655; hotema; RP11-1277H1.5	0,84
Salzman2013 ANTISENSE, coding, INTERNAL, intronic, OVERLAPTX best transcript NM_152516; novel transcript	AC018462.2; COMMD1	0,84
FK506 binding protein 10	FKBP10	0,84
sema domain, immunoglobulin domain (Ig), transmembrane domain (TM) and short cytoplasmic domain, (semaphorin) 4C	SEMA4C	0,84
defensin, beta 109, pseudogene 1B	DEFB109P1B	0,84
zinc finger protein 664	ZNF664	0,84
peptidase inhibitor 3, skin-derived	PI3	0,84
neuroligin 1	NLGN1	0,84
zinc finger protein 532	ZNF532	0,84
REST corepressor 2	RCOR2	0,84
Fc receptor-like 2	FCRL2	0,84
muscle-related coiled-coil protein	MURC	0,84
c-fos induced growth factor (vascular endothelial growth factor D)	FIGF	0,84
interleukin 1 receptor antagonist	IL1RN	0,84
tyrosine kinase 2	TYK2	0,84
transforming growth factor beta 1 induced transcript 1	TGFB1I1	0,84
G protein-coupled receptor 135	GPR135	0,84
ankyrin repeat domain 20 family, member A1	ANKRD20A1	0,84
chondroitin polymerizing factor	CHPF	0,84
glutaryl-CoA dehydrogenase	GCDH	0,84
human immunodeficiency virus type I enhancer binding protein 2	HIVEP2	0,84
uroplakin 1A	UPK1A	0,84
chemokine (C-C motif) ligand 11	CCL11	0,84
replication protein A3	RPA3	0,84
shugoshin-like 2 (S. pombe)	SGOL2	0,84
serine racemase	SRR	0,84
hippocalcin	HPCA	0,84

phospholipid scramblase 4	PLSCR4	0,84
chromosome 12 open reading frame 42	C12orf42	0,84
phosphatidylinositol glycan anchor biosynthesis class U	PIGU	0,84
S100 calcium binding protein A13	S100A13	0,84
zinc finger protein 138	ZNF138	0,84
Jeck2013 ANTISENSE, INTERNAL, intronic, ncRNA, OVEXON best transcript NR_036569	PILRB	0,84
zinc finger, DHHC-type containing 3	ZDHHC3	0,84
testis expressed 10	TEX10	0,84
SPATA31 subfamily A, member 3	SPATA31A3	0,84
UBA-like domain containing 1	UBALD1	0,84
lin-7 homolog A (C. elegans)	LIN7A	0,84
torsin family 4, member A	TOR4A	0,84
melanocortin 2 receptor (adrenocorticotropic hormone)	MC2R	0,84
angiogenin, ribonuclease, RNase A family, 5; ribonuclease, RNase A family, 4	ANG; RNASE4	0,84
diaphanous-related formin 3	DIAPH3	0,84
zinc finger protein 630	ZNF630	0,84
ribosomal protein L7a	RPL7A	0,84
NIPA-like domain containing 1	NIPAL1	0,84
transmembrane protein 217	TMEM217	0,84
G protein-coupled receptor 62	GPR62	0,84
protein tyrosine phosphatase, non-receptor type 20	PTPN20	0,84
histone cluster 1, H2bo	HIST1H2BO	0,84
mitochondrial calcium uptake 1	MICU1	0,84
signaling threshold regulating transmembrane adaptor 1	SIT1	0,84
myelin protein zero-like 3	MPZL3	0,84
glomulin, FKBP associated protein	GLMN	0,84
TWIST neighbor	TWISTNB	0,84
ceramide kinase like	CERKL	0,84
helicase, lymphoid-specific	HELLS	0,84
ribosomal RNA processing 12 homolog	RRP12	0,84
MOB kinase activator 1A	MOB1A	0,84
zinc finger, C2HC-type containing 1B	ZC2HC1B	0,84
lysine (K)-specific methyltransferase 2E	KMT2E	0,84
wingless-type MMTV integration site family, member 8A	WNT8A	0,84
zinc finger protein 524	ZNF524	0,84
solute carrier family 5 (sodium/sugar cotransporter), member 9	SLC5A9	0,84
ubiquitin conjugating enzyme E2Q family-like 1	UBE2QL1	0,84
cardiotrophin 1	CTF1	0,84
archaelysin family metallopeptidase 1	AMZ1	0,84
TRIM6-TRIM34 readthrough	TRIM6-TRIM34	0,84
PRAME family member 2	PRAMEF2	0,84
protein kinase C, epsilon	PRKCE	0,84
cysteine/tyrosine-rich 1	CYYR1	0,84

ornithine aminotransferase	OAT	0,84
olfactory receptor, family 6, subfamily C, member 65	OR6C65	0,84
pumilio RNA binding family member 1	PUM1	0,84
Rho guanine nucleotide exchange factor 1	ARHGEF1	0,84
galactose-3-O-sulfotransferase 1	GAL3ST1	0,84
FLT3-interacting zinc finger 1	FIZ1	0,84
sprouty-related, EVH1 domain containing 2	SPRED2	0,84
anti-Mullerian hormone receptor, type II	AMHR2	0,84
down-regulator of transcription 1	DR1	0,84
kelch repeat and BTB (POZ) domain containing 2	KBTBD2	0,84
SERTA domain containing 4	SERTAD4	0,84
Transcript Identified by AceView, Entrez Gene ID(s) 240	ALOX5	0,84
amyloid beta precursor protein (cytoplasmic tail) binding protein 2	APPBP2	0,84
zinc finger protein 516	ZNF516	0,84
tyrosine 3-monooxygenase/tryptophan 5-monooxygenase activation protein, epsilon pseudogene 7	YWHAEP7	0,84
ubiquitin specific peptidase 48	USP48	0,84
fibroblast growth factor binding protein 1	FGFBP1	0,84
perilipin 3	PLIN3	0,84
zinc finger and BTB domain containing 8B	ZBTB8B	0,84
SAGA complex associated factor 29 pseudogene; Transcript Identified by AceView, Entrez Gene ID(s) 388242; Uncharacterized protein [Source:UniProtKB/TrEMBL;Acc:H0YHM3]	LOC388242; LOC613038; RP11-345J4.3	0,84
arylacetamide deacetylase-like 4	AADACL4	0,84
Transcript Identified by AceView, Entrez Gene ID(s) 5825	ABCD3	0,84
pleckstrin homology domain containing, family D (with coiled-coil domains) member 1	PLEKHD1	0,84
sprouty RTK signaling antagonist 4	SPRY4	0,84
olfactory receptor, family 6, subfamily C, member 68	OR6C68	0,84
purinergic receptor P2Y, G-protein coupled, 1	P2RY1	0,84
RecQ helicase-like 4	RECQL4	0,84
Transcript Identified by AceView, Entrez Gene ID(s) 219333	USP12	0,84
VMA21 vacuolar H+-ATPase homolog (S. cerevisiae)	VMA21	0,84
coiled-coil domain containing 182	CCDC182	0,84
glycoprotein hormone alpha 2	GPHA2	0,84
RUN domain containing 3B	RUNDC3B	0,84
solute carrier family 1 (glial high affinity glutamate transporter), member 2	SLC1A2	0,84
potassium channel, subfamily U, member 1	KCNU1	0,84
late endosomal/lysosomal adaptor, MAPK and MTOR activator 3	LAMTOR3	0,84
SUMO1/sentrin/SMT3 specific peptidase 3	SENP3	0,84
Transcript Identified by AceView, Entrez Gene ID(s) 56261	GPCPD1	0,84

family with sequence similarity 216, member A	FAM216A	0,84
solute carrier family 2 (facilitated glucose transporter), member 6 [Source:HGNC Symbol;Acc:HGNC:11011]	SLC2A6	0,84
macrophage stimulating 1	MST1	0,84
ALG10, alpha-1,2-glucosyltransferase	ALG10	0,84
pituitary tumor-transforming 1 interacting protein	PTTG1IP	0,84
myelin regulatory factor	MYRF	0,84
lectin, galactoside-binding, soluble, 8	LGALS8	0,84
midline 1	MID1	0,84
kelch-like family member 8	KLHL8	0,84
small proline-rich protein 2G	SPRR2G	0,84
epithelial cell transforming 2	ECT2	0,84
family with sequence similarity 57, member A	FAM57A	0,84
zinc finger and BTB domain containing 44	ZBTB44	0,84
tubulin polymerization promoting protein	TPPP	0,84
inhibitor of DNA binding 3, dominant negative helix-loop-helix protein	ID3	0,84
alpha-2-macroglobulin	A2M	0,84
Memczak2013 ALT_ACCEPTOR, ALT_DONOR, coding, INTERNAL, intronic best transcript NM_006828	ASCC3	0,84
Homo sapiens uncharacterized LOC102724238 (LOC102724238), long non-coding RNA.; Homo sapiens uncharacterized LOC554249 (LOC554249), long non-coding RNA.; Homo sapiens hypothetical LOC554249, mRNA (cDNA clone MGC:99799 IMAGE:6378300), complete cds.; novel transcript	LOC102724238; LOC554249; RP11-111F5.4	0,84
Homo sapiens POTE ankyrin domain family, member B3 (POTEB3), transcript variant 1, mRNA.; Homo sapiens POTE ankyrin domain family, member B, mRNA (cDNA clone MGC:119371 IMAGE:40006483), complete cds.; Homo sapiens POTE ankyrin domain family, member B, mRNA (cDNA clone MGC:119372 IMAGE:40006485), complete cds.; Homo sapiens POTE ankyrin domain family, member B, mRNA (cDNA clone MGC:119373 IMAGE:40006489), complete cds.; Homo sapiens POTE ankyrin domain family, member B, mRNA (cDNA clone MGC:156116 IMAGE:40006490), complete cds.	POTEB3; POTEB; AC136352.4	0,84
lipase maturation factor 1	LMF1	0,84
insulin-like growth factor 2; INS-IGF2 readthrough	IGF2; INS-IGF2	0,84
Transcript Identified by AceView, Entrez Gene ID(s) 6764	ST5	0,84
family with sequence similarity 156, member A	FAM156A	0,84
sparc/osteonectin, cwcv and kazal-like domains proteoglycan (testican) 1	SPOCK1	0,84
transmembrane protein 50A	TMEM50A	0,84
abhydrolase domain containing 6	ABHD6	0,84
pannexin 1	PANX1	0,84
tektin 4 pseudogene 2	TEKT4P2	0,84

carnitine palmitoyltransferase 2	CPT2	0,84
family with sequence similarity 150, member A	FAM150A	0,84
sperm associated antigen 8	SPAG8	0,84
lysine (K)-specific demethylase 1B	KDM1B	0,84
dolichyl-diphosphooligosaccharide--protein glycosyltransferase subunit (non-catalytic)	DDOST	0,84
actin binding Rho activating protein	ABRA	0,84
MCF.2 cell line derived transforming sequence-like 2	MCF2L2	0,84
cysteine-rich tail protein 1	CYSRT1	0,84
zinc finger protein 233	ZNF233	0,84
methyltransferase like 21B	METTL21B	0,84
pleckstrin homology domain containing, family H (with MyTH4 domain) member 3	PLEKHH3	0,84
polycystic kidney disease 1-like 2 (gene/pseudogene)	PKD1L2	0,84
glycerophosphodiester phosphodiesterase domain containing 4	GDPD4	0,84
retrotransposon gag domain containing 1	RGAG1	0,84
transmembrane protein 71	TMEM71	0,84
Rho GTPase activating protein 31	ARHGAP31	0,84
granzyme H	GZMH	0,84
phospholipase A2, group IB (pancreas)	PLA2G1B	0,84
transition protein 2 (during histone to protamine replacement)	TNP2	0,84
RNA binding motif (RNP1, RRM) protein 3	RBM3	0,84
collectin sub-family member 10 (C-type lectin)	COLEC10	0,84
C-type lectin domain family 6, member A	CLEC6A	0,84
paired immunoglobin-like type 2 receptor alpha	PILRA	0,84
tudor domain containing 10	TDRD10	0,84
versican	VCAN	0,84
protein phosphatase 1, regulatory (inhibitor) subunit 1A	PPP1R1A	0,84
dimethylarginine dimethylaminohydrolase 2	DDAH2	0,83
methyl-CpG binding protein 2	MECP2	0,83
TMEM147 antisense RNA 1	TMEM147-AS1	0,83
neuromedin S	NMS	0,83
moesin	MSN	0,83
lysozyme G-like 1	LYG1	0,83
sulfotransferase family 1C member 4	SULT1C4	0,83
F-box protein 27	FBXO27	0,83
transient receptor potential cation channel, subfamily M, member 1	TRPM1	0,83
heterogeneous nuclear ribonucleoprotein A0	HNRNPA0	0,83
dystrobrevin binding protein 1	DTNBP1	0,83
family with sequence similarity 222, member B	FAM222B	0,83
methyltransferase like 13	METTL13	0,83
thyrotropin-releasing hormone	TRH	0,83
adenosylmethionine decarboxylase 1	AMD1	0,83
hedgehog acyltransferase	HHAT	0,83

SH2 domain containing 5	SH2D5	0,83
stimulated by retinoic acid 13	STRA13	0,83
cadherin 1, type 1	CDH1	0,83
LY75-CD302 readthrough; CD302 molecule; lymphocyte antigen 75	LY75-CD302; CD302; LY75	0,83
KIAA1211	KIAA1211	0,83
Wilms tumor 1 interacting protein	WTIP	0,83
transcriptional regulating factor 1	TRERF1	0,83
casein kinase 1, gamma 3	CSNK1G3	0,83
IKBKB interacting protein	IKBIP	0,83
tetratricopeptide repeat domain 12	TTC12	0,83
C1q and tumor necrosis factor related protein 8	C1QTNF8	0,83
POTE ankyrin domain family, member B2; POTE ankyrin domain family, member B; POTE ankyrin domain family, member B3	POTEB2; POTEB; POTEB3	0,83
N-myristoyltransferase 2	NMT2	0,83
testis expressed 43	TEX43	0,83
glutaminyl-peptide cyclotransferase	QPCT	0,83
NudC domain containing 2	NUDCD2	0,83
olfactory receptor, family 52, subfamily N, member 5	OR52N5	0,83
myosin VI	MYO6	0,83
regulator of calcineurin 2	RCAN2	0,83
leucine rich repeat containing 20	LRRC20	0,83
olfactory receptor, family 10, subfamily A, member 6 (gene/pseudogene)	OR10A6	0,83
olfactory receptor family 10 subfamily A member 6 (gene/pseudogene) [Source:HGNC Symbol;Acc:HGNC:15132]	OR10A6	0,83
ciliary rootlet coiled-coil, rootletin family member 2 [Source:HGNC Symbol;Acc:HGNC:51677]	CROCC2	0,83
sulfotransferase family 1C member 2	SULT1C2	0,83
interleukin 13 receptor, alpha 2	IL13RA2	0,83
bile acid-CoA:amino acid N-acyltransferase	BAAT	0,83
chromosome 10 open reading frame 25	C10orf25	0,83
retinoic acid receptor, beta	RARB	0,83
zinc finger protein 91	ZNF91	0,83
polypeptide N-acetylgalactosaminyltransferase 7	GALNT7	0,83
myosin light chain 4	MYL4	0,83
Memczak2013 ALT_ACCEPTOR, ALT_DONOR, coding, INTERNAL, intronic best transcript NM_006667	PGRMC1	0,83
v-rel avian reticuloendotheliosis viral oncogene homolog	REL	0,83
butyrophilin-like 10	BTNL10	0,83
protein kinase C, delta binding protein	PRKCDBP	0,83
Transcript Identified by AceView, Entrez Gene ID(s) 9793	CKAP5	0,83
guanine nucleotide binding protein (G protein), gamma 4	GNG4	0,83
D-dopachrome tautomerase-like; D-dopachrome tautomerase	DDTL; DDT	0,83

R-spondin 2	RSPO2	0,83
alkaline ceramidase 3	ACER3	0,83
RAN binding protein 2	RANBP2	0,83
family with sequence similarity 189, member B	FAM189B	0,83
FK506 binding protein 8	FKBP8	0,83
histone cluster 1, H3b	HIST1H3B	0,83
t-complex 11, testis-specific	TCP11	0,83
glutathione S-transferase mu 1	GSTM1	0,83
lysophosphatidic acid receptor 6	LPAR6	0,83
retinoid X receptor gamma	RXRG	0,83
pleiomorphic adenoma gene 1	PLAG1	0,83
chromobox homolog 7	CBX7	0,83
potassium channel, inwardly rectifying subfamily J, member 9	KCNJ9	0,83
mitochondrial amidoxime reducing component 2	MARC2	0,83
G kinase anchoring protein 1	GKAP1	0,83
RAP2B, member of RAS oncogene family	RAP2B	0,83
acyl-CoA synthetase medium-chain family member 2B	ACSM2B	0,83
pyroglutamyl-peptidase I	PGPEP1	0,83
CUB and zona pellucida-like domains 1	CUZD1	0,83
protein kinase, membrane associated tyrosine/threonine 1	PKMYT1	0,83
solute carrier family 7 (neutral amino acid transporter light chain, asc system), member 10	SLC7A10	0,83
Memczak2013 ANTISENSE, coding, INTERNAL, intronic best transcript NM_005225	E2F1	0,83
Memczak2013 ANTISENSE, CDS, coding, INTERNAL best transcript NM_000250	MPO	0,83
nuclear factor, erythroid 2-like 1	NFE2L1	0,83
lin-37 DREAM MuvB core complex component	LIN37	0,83
matrilin 3	MATN3	0,83
SLAM family member 6	SLAMF6	0,83
ADP-ribosylation factor GTPase activating protein 3	ARFGAP3	0,83
polymerase (DNA directed), epsilon 2, accessory subunit	POLE2	0,83
uncharacterized LOC643355; putative novel transcript; Transcript Identified by AceView	LOC643355; RP11-88H9.2; toyfawbu	0,83
FYVE, RhoGEF and PH domain containing 1	FGD1	0,83
coatomer protein complex subunit alpha	COPA	0,83
peptidylprolyl isomerase A (cyclophilin A)-like 4G	PPIAL4G	0,83
zinc finger protein 408	ZNF408	0,83
keratin associated protein 1-3	KRTAP1-3	0,83
MAGE family member A9; MAGE family member A9B	MAGEA9; MAGEA9B	0,83
sphingomyelin synthase 2	SGMS2	0,83
retinoid X receptor alpha; microRNA 4669	RXRA; MIR4669	0,83

surfeit 6	SURF6	0,83
LSM7 homolog, U6 small nuclear RNA and mRNA degradation associated	LSM7	0,83
Nedd4 family interacting protein 2	NDFIP2	0,83
N-methylpurine DNA glycosylase	MPG	0,83
synapse defective 1, Rho GTPase, homolog 1 (C. elegans)	SYDE1	0,83
zinc finger protein 677	ZNF677	0,83
amylase, alpha 1C (salivary); amylase, alpha 1A (salivary)	AMY1C; AMY1A	0,83
Transcript Identified by AceView, Entrez Gene ID(s) 4846	NOS3	0,83
zinc finger protein 746	ZNF746	0,83
TP53 target 3D	TP53TG3D	0,83
oviductal glycoprotein 1	OVGP1	0,83
dynein, axonemal, heavy chain 3	DNAH3	0,83
F-box protein 3	FBXO3	0,83
Transcript Identified by AceView, Entrez Gene ID(s) 161436	EML5	0,83
chloride channel, voltage-sensitive Ka	CLCNKA	0,83
zinc finger protein 470	ZNF470	0,83
HAUS augmin like complex subunit 7; three prime repair exonuclease 2	HAUS7; TREX2	0,83
NADH dehydrogenase (ubiquinone) 1 alpha subcomplex, 12	NDUFA12	0,83
G protein-coupled receptor 35	GPR35	0,83
Fc fragment of IgE, high affinity I, receptor for; alpha polypeptide	FCER1A	0,83
Transcript Identified by AceView, Entrez Gene ID(s) 8829	NRP1	0,83
methyltransferase like 17	METTL17	0,83
leucine-rich repeats and death domain containing 1	LRRD1	0,83
forkhead box G1	FOXG1	0,83
choline/ethanolamine phosphotransferase 1	CEPT1	0,83
phospholipase A2, group IIA (platelets, synovial fluid)	PLA2G2A	0,83
mesenchyme homeobox 1	MEOX1	0,83
aryl-hydrocarbon receptor repressor; programmed cell death 6	AHRR; PDCD6	0,83
fetuin B	FETUB	0,83
serpin peptidase inhibitor, clade B (ovalbumin), member 7	SERPINB7	0,83
solute carrier family 4, sodium bicarbonate cotransporter, member 8	SLC4A8	0,83
enolase 1, (alpha)	ENO1	0,83
cysteine-rich secretory protein LCCL domain containing 1	CRISPLD1	0,83
inhibin beta C	INHBC	0,83
hydroxymethylbilane synthase	HMBS	0,83
nudix hydrolase 12	NUDT12	0,83
chromosome 2 open reading frame 80	C2orf80	0,83
maestro heat-like repeat family member 5	MROH5	0,83
Transcript Identified by AceView, Entrez Gene ID(s) 84267	C9orf64	0,83
hydroxymethylbilane synthase	HMBS	0,83

ectonucleotide pyrophosphatase/phosphodiesterase 5 (putative)	ENPP5	0,83
family with sequence similarity 172, member A; POU domain class 5, transcription factor 2; microRNA 2277	FAM172A; POU5F2; MIR2277	0,83
spermatogenesis associated 41 (non-protein coding)	SPATA41	0,83
glypican 1	GPC1	0,83
ArfGAP with FG repeats 2	AGFG2	0,83
chromosome 4 open reading frame 46	C4orf46	0,83
solute carrier family 25, member 40	SLC25A40	0,83
olfactory receptor, family 7, subfamily D, member 4	OR7D4	0,83
hydroxymethylbilane synthase	HMBS	0,83
atypical chemokine receptor 2	ACKR2	0,83
Zhang2013 ALT_ACCEPTOR, ALT_DONOR, coding, INTERNAL, intronic best transcript NM_198253	TERT	0,83
PRAME family member 25	PRAMEF25	0,83
protocadherin gamma subfamily C, 3; protocadherin gamma subfamily A, 12; protocadherin gamma subfamily B, 4; protocadherin gamma subfamily A, 8; protocadherin gamma subfamily A, 1; protocadherin gamma subfamily A, 10; protocadherin gamma subfamily A, 11; protocadherin gamma subfamily A, 2; protocadherin gamma subfamily A, 3; protocadherin gamma subfamily A, 4; protocadherin gamma subfamily A, 5; protocadherin gamma subfamily A, 6; protocadherin gamma subfamily A, 7; protocadherin gamma subfamily A, 9; protocadherin gamma subfamily B, 1; protocadherin gamma subfamily B, 2; protocadherin gamma subfamily B, 3; protocadherin gamma subfamily B, 5; protocadherin gamma subfamily B, 6; protocadherin gamma subfamily B, 7; protocadherin gamma subfamily C, 4; protocadherin gamma subfamily C, 5	PCDHGC3; PCDHGA12; PCDHGB4; PCDHGA8; PCDHGA1; PCDHGA10; PCDHGA11; PCDHGA2; PCDHGA3; PCDHGA4; PCDHGA5; PCDHGA6; PCDHGA7; PCDHGA9; PCDHGB1; PCDHGB2; PCDHGB3; PCDHGB5; PCDHGB6; PCDHGB7; PCDHGC4; PCDHGC5	0,83
interferon stimulated exonuclease gene 20kDa	ISG20	0,83
interferon, alpha-inducible protein 6	IFI6	0,83
olfactory receptor, family 10, subfamily J, member 3	OR10J3	0,83
PTPRF interacting protein, binding protein 2 (liprin beta 2)	PPFIBP2	0,83
dysferlin	DYSF	0,83
jagged 2	JAG2	0,83
zinc finger, BED-type containing 5	ZBED5	0,83
X-linked Kx blood group related 4	XKR4	0,83
highly divergent homeobox	HDX	0,83

ZFP41 zinc finger protein	ZFP41	0,83
sulfotransferase family 4A member 1	SULT4A1	0,83
late cornified envelope 2B	LCE2B	0,83
thioesterase superfamily member 6	THEM6	0,83
tousled-like kinase 1	TLK1	0,83
ADP-ribosylation factor like GTPase 4D	ARL4D	0,83
MT-RNR2-like 1	MTRNR2L1	0,83
dentin sialophosphoprotein	DSPP	0,83
fibroblast growth factor 5	FGF5	0,83
arginine vasopressin receptor 1A	AVPR1A	0,83
DENN/MADD domain containing 1B	DENND1B	0,83
matrix metallopeptidase 16 (membrane-inserted)	MMP16	0,83
neurexophilin and PC-esterase domain family, member 2	NXPE2	0,83
olfactory receptor, family 2, subfamily G, member 3	OR2G3	0,83
pre-mRNA processing factor 39	PRPF39	0,83
valyl-tRNA synthetase	VARS	0,83
eukaryotic translation initiation factor 2A, 65kDa	EIF2A	0,83
chorionic somatomammotropin hormone 1 (placental lactogen)	CSH1	0,83
DEP domain containing 1	DEPDC1	0,83
transmembrane protein 184A	TMEM184A	0,83
potassium channel, voltage gated Shaw related subfamily C, member 4	KCNC4	0,83
CDC42 small effector 1	CDC42SE1	0,83
TNNI3 interacting kinase	TNNI3K	0,83
REV1, DNA directed polymerase	REV1	0,83
gremlin 1, DAN family BMP antagonist [Source:HGNC Symbol;Acc:HGNC:2001]	GREM1	0,83
solute carrier family 25 (mitochondrial folate carrier), member 32	SLC25A32	0,83
protein phosphatase 6, regulatory subunit 2	PPP6R2	0,83
transmembrane protein 133	TMEM133	0,83
diffuse panbronchiolitis critical region 1	DPCR1	0,83
protocadherin 19	PCDH19	0,83
major histocompatibility complex, class I, C	HLA-C	0,83
zinc finger protein 467	ZNF467	0,83
hydroxycarboxylic acid receptor 2	HCAR2	0,83
small proline-rich protein 4	SPRR4	0,83
l(3)mbt-like 2 (Drosophila)	L3MBTL2	0,83
tumor necrosis factor	TNF	0,83
NEDD4 binding protein 2	N4BP2	0,83
PTPN13-like, Y-linked pseudogene 3	PRYP3	0,83
family with sequence similarity 81, member B	FAM81B	0,83
zinc finger, CCHC domain containing 18	ZCCHC18	0,83
MT-RNR2-like 7	MTRNR2L7	0,83
carboxypeptidase E	CPE	0,83

structural maintenance of chromosomes 1B	SMC1B	0,83
PTPN13-like, Y-linked 2; PTPN13-like, Y-linked	PRY2; PRY	0,83
dolichyl-phosphate mannosyltransferase polypeptide 3	DPM3	0,83
Memczak2013 ANTISENSE, CDS, coding, INTERNAL best transcript NM_001130967	ZNF385A	0,83
MALT1 paracaspase	MALT1	0,83
F-box protein 48	FBXO48	0,83
dickkopf WNT signaling pathway inhibitor 3	DKK3	0,83
MAGE family member B2	MAGEB2	0,83
ubiquinol-cytochrome c reductase, complex III subunit XI	UQCR11	0,83
insulin-like growth factor 2 mRNA binding protein 3	IGF2BP3	0,83
WD repeat domain 88	WDR88	0,83
coagulation factor III (thromboplastin, tissue factor)	F3	0,83
ADP-ribosyltransferase 4 (Dombrock blood group)	ART4	0,83
Homo sapiens coiled-coil domain containing 101 pseudogene (LOC388242), non-coding RNA.; Homo sapiens SAGA complex associated factor 29 pseudogene (LOC613038), non-coding RNA.; Transcript Identified by AceView, Entrez Gene ID(s) 613038, RefSeq ID(s) NR_002557; Transcript Identified by AceView, Entrez Gene ID(s) 613038; Protein LOC101929849 [Source:UniProtKB/TrEMBL;Acc:H0YHN6]	LOC388242; LOC613038; RP11-347C12.3	0,83
ubiquitously transcribed tetratricopeptide repeat containing, Y-linked	UTY	0,83
F-box protein 21	FBXO21	0,83
syntaxin 5	STX5	0,83
Transcript Identified by AceView, Entrez Gene ID(s) 23126	POGZ	0,83
GSK3B interacting protein	GSKIP	0,83
chemokine (C-X-C motif) receptor 1	CXCR1	0,83
sorting nexin 10	SNX10	0,83
cyclic nucleotide gated channel alpha 4	CNGA4	0,83
SPECC1L-ADORA2A readthrough (NMD candidate)	SPECC1L-ADORA2A	0,83
mediator complex subunit 18	MED18	0,83
protein tyrosine phosphatase, receptor type, M	PTPRM	0,83
olfactory receptor, family 4, subfamily C, member 15	OR4C15	0,83
jumonji domain containing 1C	JMJD1C	0,82
fibronectin type III domain containing 1	FNDC1	0,82
steroid sulfatase (microsomal), isozyme S	STS	0,82
ubiquitin specific peptidase 17-like family member 12	USP17L12	0,82
SEC24 homolog A, COPII coat complex component	SEC24A	0,82
lysozyme-like 6	LYZL6	0,82
UDP-galactose-4-epimerase	GALE	0,82
mitochondrial fission process 1	MTFP1	0,82
F-box and leucine-rich repeat protein 6	FBXL6	0,82
Rho GTPase activating protein 23	ARHGAP23	0,82

histone cluster 1, H4k	HIST1H4K	0,82
poly(A) binding protein interacting protein 1	PAIP1	0,82
nucleosome assembly protein 1-like 6	NAP1L6	0,82
interleukin 3	IL3	0,82
nucleolar and spindle associated protein 1	NUSAP1	0,82
apoptosis-associated tyrosine kinase	AATK	0,82
Rho guanine nucleotide exchange factor 25	ARHGEF25	0,82
SH2B adaptor protein 1	SH2B1	0,82
proline rich 34	PRR34	0,82
Zhang2013 ALT_ACCEPTOR, ALT_DONOR, coding, INTERNAL, intronic best transcript NM_001145297	EXOC7	0,82
ATP synthase, H+ transporting, mitochondrial Fo complex subunit G2	ATP5L2	0,82
phospholipase C-like 1	PLCL1	0,82
chromosome 8 open reading frame 59	C8orf59	0,82
MIS18 binding protein 1	MIS18BP1	0,82
centromere protein J	CENPJ	0,82
leucine rich repeat containing 29	LRRC29	0,82
RAB15 effector protein	REP15	0,82
abhydrolase domain containing 5	ABHD5	0,82
acid phosphatase 2, lysosomal	ACP2	0,82
solute carrier family 38, member 9	SLC38A9	0,82
guanine nucleotide binding protein-like 2 (nucleolar)	GNL2	0,82
ATPase family, AAA domain containing 3C	ATAD3C	0,82
POP7 homolog, ribonuclease P/MRP subunit	POP7	0,82
COP9 signalosome subunit 7A	COPS7A	0,82
SMAD family member 3	SMAD3	0,82
MT-RNR2-like 9	MTRNR2L9	0,82
brain expressed, associated with NEDD4, 1	BEAN1	0,82
olfactory receptor, family 2, subfamily AE, member 1	OR2AE1	0,82
chromosome 11 open reading frame 85	C11orf85	0,82
phospholipase A2, group X	PLA2G10	0,82
glypican 6	GPC6	0,82
chromosome 3 open reading frame 35	C3orf35	0,82
CD1e molecule	CD1E	0,82
protein phosphatase methylesterase 1	PPME1	0,82
zinc finger protein 639	ZNF639	0,82
tigger transposable element derived 5	TIGD5	0,82
Sp2 transcription factor	SP2	0,82
carnosine synthase 1	CARNS1	0,82
parvin, gamma	PARVG	0,82
insulin like growth factor binding protein 5	IGFBP5	0,82
TP53 target 3B	TP53TG3B	0,82
long intergenic non-protein coding RNA 668	LINC00668	0,82
platelet factor 4 variant 1	PF4V1	0,82

peptidylprolyl isomerase A (cyclophilin A)-like 4F	PPIAL4F	0,82
RAB2B, member RAS oncogene family	RAB2B	0,82
zinc finger protein 780B	ZNF780B	0,82
histone cluster 1, H3i	HIST1H3I	0,82
importin 5	IPO5	0,82
Jeck2013 ALT_ACCEPTOR, ALT_DONOR, coding, INTERNAL, intronic best transcript NM_145177	DHRSX	0,82
indolethylamine N-methyltransferase; family with sequence similarity 188, member B; INMT-FAM188B readthrough (NMD candidate)	INMT; FAM188B; INMT-FAM188B	0,82
zinc finger protein 160	ZNF160	0,82
ADAM metallopeptidase domain 10	ADAM10	0,82
leucine-rich repeat LGI family, member 4	LGI4	0,82
pleckstrin homology domain containing, family A member 6	PLEKHA6	0,82
zinc finger protein 175	ZNF175	0,82
Transcript Identified by AceView, Entrez Gene ID(s) 3551	IKBKB	0,82
phosphatidylinositol-4,5-bisphosphate 3-kinase, catalytic subunit delta	PIK3CD	0,82
ribosomal protein S6 kinase, 90kDa, polypeptide 1	RPS6KA1	0,82
Memczak2013 ANTISENSE, CDS, coding, INTERNAL best transcript NM_001195422	GTPBP3	0,82
Jeck2013 ANTISENSE, coding, INTERNAL, intronic best transcript NM_005336	HDLBP	0,82
heparan sulfate (glucosamine) 3-O-sulfotransferase 5	HS3ST5	0,82
DEAH (Asp-Glu-Ala-His) box polypeptide 40	DHX40	0,82
GIPC PDZ domain containing family, member 2	GIPC2	0,82
integrin alpha 10	ITGA10	0,82
Transcript Identified by AceView, Entrez Gene ID(s) 5426	POLE	0,82
solute carrier family 22 (organic cation/zwitterion transporter), member 4	SLC22A4	0,82
carboxyl ester lipase	CEL	0,82
uncoupling protein 3 (mitochondrial, proton carrier)	UCP3	0,82
syntaxin binding protein 2	STXBP2	0,82
zinc finger protein 747	ZNF747	0,82
solute carrier family 38, member 7	SLC38A7	0,82
angiopoietin like 8	ANGPTL8	0,82
fem-1 homolog c (C. elegans)	FEM1C	0,82
nudE neurodevelopment protein 1; microRNA 484	NDE1; MIR484	0,82
epidermal growth factor	EGF	0,82
deleted in azoospermia 4; deleted in azoospermia 1	DAZ4; DAZ1	0,82
solute carrier family 39 (zinc transporter), member 2	SLC39A2	0,82
myelin protein zero-like 2	MPZL2	0,82
DOT1-like histone H3K79 methyltransferase	DOT1L	0,82
BCL2/adenovirus E1B 19kDa interacting protein 3	BNIP3	0,82

Memczak2013 ANTISENSE, CDS, coding, INTERNAL, UTR3 best transcript NM_005999	TSNAX	0,82
unc-5 netrin receptor B	UNC5B	0,82
late cornified envelope 2C	LCE2C	0,82
eukaryotic translation initiation factor 2-alpha kinase 3	EIF2AK3	0,82
solute carrier family 25, member 27	SLC25A27	0,82
lymphocyte antigen 6 complex, locus G6F	LY6G6F	0,82
WD repeat domain 13	WDR13	0,82
transmembrane and coiled-coil domains 4	TMCO4	0,82
Kruppel-like factor 9	KLF9	0,82
G protein-coupled receptor, class C, group 5, member C	GPRC5C	0,82
gastrokine 2	GKN2	0,82
gastrokine 1	GKN1	0,82
PRAME family member 15; PRAME family member 9	PRAMEF15; PRAMEF9	0,82
deleted in lymphocytic leukemia 2 (non-protein coding); microRNA 15a; microRNA 16-1; microRNA 3613	DLEU2; MIR15A; MIR16-1; MIR3613	0,82
protein kinase, cAMP-dependent, regulatory, type I, alpha	PRKAR1A	0,82
prokineticin receptor 1	PROKR1	0,82
gamma-glutamyltransferase 7	GGT7	0,82
protein O-fucosyltransferase 1; microRNA 1825	POFUT1; MIR1825	0,82
G protein-coupled receptor 155	GPR155	0,82
golgin A8 family, member E, pseudogene; golgin A8 family, member D, pseudogene	GOLGA8EP; GOLGA8DP	0,82
YY1 associated factor 2	YAF2	0,82
Transcript Identified by AceView, Entrez Gene ID(s) 5783	PTPN13	0,82
myotubularin related protein 6	MTMR6	0,82
cytochrome c oxidase subunit VIIa polypeptide 1 (muscle)	COX7A1	0,82
sterile alpha motif domain containing 7	SAMD7	0,82
MORC family CW-type zinc finger 2	MORC2	0,82
BTB (POZ) domain containing 18	BTBD18	0,82
retinal pigment epithelium-specific protein 65kDa	RPE65	0,82
family with sequence similarity 186, member A	FAM186A	0,82
glucuronic acid epimerase	GLCE	0,82
proline-rich nuclear receptor coactivator 1	PNRC1	0,82
Gilles de la Tourette syndrome chromosome region, candidate 1 (non-protein coding)	GTSCR1	0,82
KIAA1524	KIAA1524	0,82
solute carrier family 30 (zinc transporter), member 5	SLC30A5	0,82
novel transcript; Transcript Identified by AceView, Entrez Gene ID(s) 64116	RP11-499E18.1; SLC39A8	0,82

myosin IIIA	MYO3A	0,82
golgin, RAB6-interacting	GORAB	0,82
CD81 molecule	CD81	0,82
beta-1,3-glucuronyltransferase 2	B3GAT2	0,82
zinc finger protein 286A	ZNF286A	0,82
MAM domain containing glycosylphosphatidylinositol anchor 1	MDGA1	0,82
cytochrome b5 reductase 3	CYB5R3	0,82
ubiquitin-like with PHD and ring finger domains 1	UHRF1	0,82
intraflagellar transport 27	IFT27	0,82
transglutaminase 2	TGM2	0,82
family with sequence similarity 231, member A	FAM231A	0,82
cytochrome P450, family 2, subfamily R, polypeptide 1	CYP2R1	0,82
coenzyme Q3 methyltransferase	COQ3	0,82
nuclear receptor interacting protein 3	NRIP3	0,82
regulator of G-protein signaling 21	RGS21	0,82
vomeronasal 1 receptor 4	VN1R4	0,82
novel transcript; Transcript Identified by AceView, Entrez Gene ID(s) 79634	AC010894.3; SCRN3	0,82
urocortin 2	UCN2	0,82
family with sequence similarity 155, member A	FAM155A	0,82
gamma-aminobutyric acid (GABA) A receptor, gamma 1	GABRG1	0,82
cadherin 5, type 2 (vascular endothelium)	CDH5	0,82
oxidized low density lipoprotein (lectin-like) receptor 1	OLR1	0,82
MAGE family member F1	MAGEF1	0,82
methylthioribose-1-phosphate isomerase 1	MRI1	0,82
Memczak2013 ANTISENSE, CDS, coding, INTERNAL, intronic, UTR3 best transcript NM_001099678	LRRC58	0,82
chromosome 6 open reading frame 201	C6orf201	0,82
carcinoembryonic antigen-related cell adhesion molecule 3	CEACAM3	0,82
H2A histone family, member Y2	H2AFY2	0,82
progesterone receptor	PGR	0,82
leucine rich repeat containing 71	LRRC71	0,82
sterile alpha motif domain containing 9-like	SAMD9L	0,82
notch 4	NOTCH4	0,82
post-GPI attachment to proteins 2	PGAP2	0,82
protein phosphatase 4, regulatory subunit 3B	PPP4R3B	0,82
circadian associated repressor of transcription	CIART	0,82
Transcript Identified by AceView, Entrez Gene ID(s) 9361	LONP1	0,82
frizzled-related protein	FRZB	0,82
family with sequence similarity 188, member A	FAM188A	0,82
oculomedin	OCLM	0,82
globoside alpha-1,3-N-acetylgalactosaminyltransferase 1	GBGT1	0,82
dehydrogenase/reductase (SDR family) member 13	DHRS13	0,82
Kruppel-like factor 2	KLF2	0,82
casein alpha s1	CSN1S1	0,82

seizure related 6 homolog (mouse)-like	SEZ6L	0,82
fer-1-like family member 6	FER1L6	0,82
hydroxyacid oxidase (glycolate oxidase) 1	HAO1	0,82
DEAD (Asp-Glu-Ala-Asp) box polypeptide 43	DDX43	0,82
SLIT-ROBO Rho GTPase activating protein 3	SRGAP3	0,82
VPS9 domain containing 1	VPS9D1	0,82
transient receptor potential cation channel, subfamily M, member 6	TRPM6	0,82
armadillo repeat containing, X-linked 4	ARMCX4	0,82
glutamate receptor, metabotropic 6	GRM6	0,82
transmembrane protein 140	TMEM140	0,82
acyl-CoA synthetase medium-chain family member 5	ACSM5	0,82
neurotensin receptor 2	NTSR2	0,82
Memczak2013 ANTISENSE, CDS, coding, INTERNAL, UTR3 best transcript NM_004521	KIF5B	0,82
leucine rich repeat containing 37B	LRRC37B	0,82
uncharacterized LOC103611081; uncharacterized LOC100507431; Transcript Identified by AceView; novel transcript	LOC103611081; LOC100507431; RP11-890B15.3; RP11-890B15.2; klerglaw	0,82
cilia and flagella associated protein 100	CFAP100	0,82
microtubule-associated protein 1S	MAP1S	0,82
kelch-like family member 3	KLHL3	0,82
cysteine-rich protein 3	CRIP3	0,82
Transcript Identified by AceView, Entrez Gene ID(s) 6453	ITSN1	0,82
solute carrier family 6 (neurotransmitter transporter), member 2	SLC6A2	0,82
granzyme B	GZMB	0,82
zinc finger CCCH-type containing 14	ZC3H14	0,81
olfactory receptor, family 5, subfamily M, member 10	OR5M10	0,81
heat shock 70kDa protein 1A; heat shock 70kDa protein 1B	HSPA1A; HSPA1B	0,81
poly(A) binding protein, cytoplasmic 3	PABPC3	0,81
zinc finger protein 395	ZNF395	0,81
peptidylprolyl isomerase A (cyclophilin A)-like 4E	PPIAL4E	0,81
talin 2	TLN2	0,81
polymerase (RNA) II (DNA directed) polypeptide J4, pseudogene	POLR2J4	0,81
phosphodiesterase 9A	PDE9A	0,81
forkhead box O6	FOXO6	0,81
peptidyl arginine deiminase, type I	PADI1	0,81
membrane bound O-acyltransferase domain containing 7	MBOAT7	0,81

eukaryotic translation initiation factor 3, subunit E	EIF3E	0,81
zinc finger protein 275	ZNF275	0,81
acyl-CoA binding domain containing 3	ACBD3	0,81
Ras association (RalGDS/AF-6) domain family (N-terminal) member 8	RASSF8	0,81
histone cluster 1, H2am; histone cluster 1, H3j	HIST1H2AM; HIST1H3J	0,81
APC membrane recruitment protein 2	AMER2	0,81
chromosome 5 open reading frame 60	C5orf60	0,81
core-binding factor, runt domain, alpha subunit 2; translocated to, 2	CBFA2T2	0,81
sarcoglycan zeta	SGCZ	0,81
prosaposin-like 1 (gene/pseudogene)	PSAPL1	0,81
amyloid beta (A4) precursor protein-binding, family A, member 1	APBA1	0,81
V-set and immunoglobulin domain containing 2	VSIG2	0,81
amylase, alpha 1B (salivary); amylase, alpha 1A (salivary)	AMY1B; AMY1A	0,81
hyaluronan-mediated motility receptor (RHAMM)	HMMR	0,81
Ras homolog enriched in brain	RHEB	0,81
mediator complex subunit 21	MED21	0,81
transcription elongation factor A (SII)-like 8	TCEAL8	0,81
Fc fragment of IgG, low affinity IIIa, receptor (CD16a)	FCGR3A	0,81
inositol polyphosphate-5-phosphatase J	INPP5J	0,81
cystic fibrosis transmembrane conductance regulator	CFTR	0,81
v-ets avian erythroblastosis virus E26 oncogene homolog	ERG	0,81
mitochondrial elongation factor 1	MIEF1	0,81
epiphycan	EPYC	0,81
olfactory receptor, family 8, subfamily B, member 4 (gene/pseudogene)	OR8B4	0,81
olfactory receptor, family 52, subfamily L, member 1	OR52L1	0,81
MOB kinase activator 3A	MOB3A	0,81
cytochrome P450, family 1, subfamily A, polypeptide 2	CYP1A2	0,81
histone cluster 1, H3f	HIST1H3F	0,81
synaptotagmin XVI	SYT16	0,81
tenascin R	TNR	0,81
proprotein convertase subtilisin/kexin type 9	PCSK9	0,81
alpha hemoglobin stabilizing protein	AHSP	0,81
TM4SF19 antisense RNA 1	TM4SF19-AS1	0,81
GRP1 (general receptor for phosphoinositides 1)-associated scaffold protein	GRASP	0,81
claudin 23	CLDN23	0,81
tumor protein p63 regulated 1	TPRG1	0,81
collagen, type I, alpha 2	COL1A2	0,81
Transcript Identified by AceView, Entrez Gene ID(s) 51339	DACT1	0,81

TEA domain family member 4	TEAD4	0,81
meiosis specific with coiled-coil domain	MEIOC	0,81
neuropilin 2	NRP2	0,81
Transcript Identified by AceView, Entrez Gene ID(s) 54875	CNTLN	0,81
zinc finger protein 492	ZNF492	0,81
doublecortin domain containing 1	DCDC1	0,81
leucine-rich repeats and immunoglobulin-like domains 2	LRIG2	0,81
cyclic nucleotide binding domain containing 1	CNBD1	0,81
annexin A2	ANXA2	0,81
abnormal spindle microtubule assembly	ASPM	0,81
RCC1 domain containing 1	RCCD1	0,81
beta-carotene oxygenase 2; ribosomal protein S12 pseudogene 21	BCO2; RPS12P21	0,81
cytochrome P450, family 2, subfamily D, polypeptide 6	CYP2D6	0,81
rogdi homolog	ROGDI	0,81
cathepsin S	CTSS	0,81
PRAME family member 2	PRAMEF2	0,81
olfactory receptor, family 1, subfamily J, member 1	OR1J1	0,81
Transcript Identified by AceView, Entrez Gene ID(s) 5992	RFX4	0,81
gametogenetin	GGN	0,81
Ras-like without CAAX 1	RIT1	0,81
prostaglandin E receptor 1	PTGER1	0,81
idnK, gluconokinase homolog (E. coli)	IDNK	0,81
dehydrogenase/reductase (SDR family) member 7B	DHRS7B	0,81
ARL2-SNX15 readthrough (NMD candidate)	ARL2-SNX15	0,81
phosphoinositide-3-kinase interacting protein 1	PIK3IP1	0,81
chromosome 10 open reading frame 126	C10orf126	0,81
cysteine rich transmembrane BMP regulator 1 (chordin-like)	CRIM1	0,81
immediate early response 3	IER3	0,81
THO complex 1	THOC1	0,81
pregnancy-associated plasma protein A, pappalysin 1	PAPPA	0,81
Memczak2013 ANTISENSE, CDS, coding, INTERNAL best transcript NM_012335	MYO1F	0,81
phosphodiesterase 4D, cAMP-specific	PDE4D	0,81
aldehyde dehydrogenase 16 family, member A1	ALDH16A1	0,81
amyotrophic lateral sclerosis 2 chromosome region candidate 12	ALS2CR12	0,81
aldehyde dehydrogenase 9 family, member A1	ALDH9A1	0,81
olfactory receptor, family 6, subfamily C, member 76	OR6C76	0,81
phosphoribosyl transferase domain containing 1	PRTFDC1	0,81
semenogelin II	SEMG2	0,81
ubiquitin specific peptidase 44	USP44	0,81
solute carrier family 35, member D3	SLC35D3	0,81
proline-rich transmembrane protein 1	PRRT1	0,81
chemokine (C-C motif) ligand 20	CCL20	0,81

TNF receptor-associated factor 4	TRAF4	0,81
keratin associated protein 10-10	KRTAP10-10	0,81
G protein-coupled receptor 63	GPR63	0,81
CUGBP, Elav-like family member 2	CELF2	0,81
Transcript Identified by AceView, Entrez Gene ID(s) 5771	PTPN2	0,81
FK506 binding protein like	FKBPL	0,81
solute carrier family 2 (facilitated glucose transporter), member 2	SLC2A2	0,81
LysM, putative peptidoglycan-binding, domain containing 3	LYSMD3	0,81
renin binding protein	RENBP	0,81
protein phosphatase, Mg2+/Mn2+ dependent, 1H	PPM1H	0,81
t-complex 10-like	TCP10L	0,81
glycosylphosphatidylinositol anchor attachment 1	GPAA1	0,81
SET and MYND domain containing 1	SMYD1	0,81
vacuolar protein sorting 37 homolog A (S. cerevisiae)	VPS37A	0,81
EF-hand calcium binding domain 12	EFCAB12	0,81
CD5 molecule-like	CD5L	0,81
dedicator of cytokinesis 11	DOCK11	0,81
guanine deaminase	GDA	0,81
flavin containing monooxygenase 5	FMO5	0,81
estrogen-related receptor beta	ESRRB	0,81
aldehyde dehydrogenase 4 family, member A1	ALDH4A1	0,81
CDGSH iron sulfur domain 3	CISD3	0,81
testis and ovary specific PAZ domain containing 1	TOPAZ1	0,81
BEN domain containing 3	BEND3	0,81
family with sequence similarity 91, member A1	FAM91A1	0,81
Zhang2013 ALT_ACCEPTOR, ALT_DONOR, coding, INTERNAL, intronic best transcript NM_001042576	RRBP1	0,81
ribosomal protein L4	RPL4	0,81
cyclin-dependent kinase 18	CDK18	0,81
Transcript Identified by AceView, Entrez Gene ID(s) 10265; novel transcript	CTD-3032H12.2; IRX5	0,81
nuclear receptor subfamily 1, group I, member 3	NR1I3	0,81
histone cluster 1, H2ba	HIST1H2BA	0,81
solute carrier family 25, member 52	SLC25A52	0,81
cyclin G associated kinase	GAK	0,81
olfactory receptor, family 10, subfamily H, member 1	OR10H1	0,81
Kruppel-like factor 10	KLF10	0,81
ALG6, alpha-1,3-glucosyltransferase	ALG6	0,81
cyclin-dependent kinase-like 3	CDKL3	0,81
transmembrane protein 74	TMEM74	0,81
MAS-related GPR, member G	MRGPRG	0,81
zinc activated ligand-gated ion channel	ZACN	0,81
apolipoprotein A-V	APOA5	0,81

brain abundant, membrane attached signal protein 1	BASP1	0,81
TSPY-like 6	TSPYL6	0,81
coiled-coil domain containing 186; microRNA 2110	CCDC186; MIR2110	0,81
uncharacterized LOC101928137; novel transcript	LOC101928137; RP11-314D7.2	0,81
prostaglandin E receptor 3 (subtype EP3)	PTGER3	0,81
CTAGE family, member 8; CTAGE family, member 4	CTAGE8; CTAGE4	0,81
RAB9A, member RAS oncogene family	RAB9A	0,81
acyl-CoA synthetase long-chain family member 6	ACSL6	0,81
olfactory receptor, family 11, subfamily L, member 1	OR11L1	0,81
RNA binding motif protein 25	RBM25	0,81
HMP19 protein; Neuron-specific protein family member 2 [Source:UniProtKB/Swiss-Prot;Acc:Q9Y328]; Transcript Identified by AceView, Entrez Gene ID(s) 51617	HMP19	0,81
growth differentiation factor 7	GDF7	0,81
solute carrier family 19 (thiamine transporter), member 3	SLC19A3	0,81
histone cluster 2, H3a	HIST2H3A	0,81
translocase of outer mitochondrial membrane 40 homolog (yeast)-like; microRNA 5187	TOMM40L; MIR5187	0,81
Transcript Identified by AceView, Entrez Gene ID(s) 3836	KPNA1	0,81
zinc finger protein 26	ZNF26	0,81
keratin associated protein 5-7	KRTAP5-7	0,81
thiosulfate sulfurtransferase (rhodanese)-like domain containing 1	TSTD1	0,81
dyslexia susceptibility 1 candidate 1	DYX1C1	0,81
haptoglobin-related protein	HPR	0,81
sarcolemma associated protein	SLMAP	0,81
interleukin 31 receptor A	IL31RA	0,81
chromosome 2 open reading frame 91	C2orf91	0,81
potassium channel, voltage gated modifier subfamily G, member 3	KCNG3	0,81
netrin 3	NTN3	0,81
gamma-aminobutyric acid (GABA) A receptor, alpha 3	GABRA3	0,81
galectin-related inter-fiber protein	GRIFIN	0,81
TBC1 domain family, member 8B (with GRAM domain)	TBC1D8B	0,81
intercellular adhesion molecule 3	ICAM3	0,81
chromosome 18 open reading frame 42	C18orf42	0,81
histone cluster 1, H2al; histone cluster 1, H2bn	HIST1H2AL; HIST1H2BN	0,81
KIAA1462	KIAA1462	0,81
sorting nexin 2	SNX2	0,80
desmoglein 1	DSG1	0,80
docking protein 3	DOK3	0,80

ring finger protein 24	RNF24	0,80
ATPase, class V, type 10A	ATP10A	0,80
chromosome 15 open reading frame 40	C15orf40	0,80
phosphorylase kinase, alpha 2 (liver)	PHKA2	0,80
transient receptor potential cation channel, subfamily C, member 4	TRPC4	0,80
olfactory receptor, family 12, subfamily D, member 3	OR12D3	0,80
fibroblast growth factor receptor 3	FGFR3	0,80
proline dehydrogenase (oxidase) 2	PRODH2	0,80
spire-type actin nucleation factor 2	SPIRE2	0,80
phosphodiesterase 4B, cAMP-specific	PDE4B	0,80
protease, serine, 3	PRSS3	0,80
syntaxin binding protein 6 (amisyn)	STXBP6	0,80
Memczak2013 ANTISENSE, CDS, coding, INTERNAL best transcript NM_198525	KIF7	0,80
LDL receptor related protein 6	LRP6	0,80
glia maturation factor, gamma	GMFG	0,80
solute carrier family 26 (anion exchanger), member 6; microRNA 6824	SLC26A6; MIR6824	0,80
angiopoietin 2	ANGPT2	0,80
MT-RNR2-like 3	MTRNR2L3	0,80
proline rich 7 (synaptic)	PRR7	0,80
coiled-coil-helix-coiled-coil-helix domain containing 10	CHCHD10	0,80
glycerophosphodiester phosphodiesterase domain containing 5	GDPD5	0,80
dynamin 1-like	DNM1L	0,80
dermatan sulfate epimerase	DSE	0,80
CART prepropeptide	CARTPT	0,80
frizzled class receptor 2	FZD2	0,80
electron-transferring-flavoprotein dehydrogenase	ETFDH	0,80
adenylate kinase 2	AK2	0,80
peroxisomal biogenesis factor 10	PEX10	0,80
RAD51 associated protein 1	RAD51AP1	0,80
phosphoinositide-interacting regulator of transient receptor potential channels	PIRT	0,80
chromobox homolog 8	CBX8	0,80
translation machinery associated 16 homolog	TMA16	0,80
MDM2 proto-oncogene, E3 ubiquitin protein ligase	MDM2	0,80
olfactory receptor, family 4, subfamily C, member 3	OR4C3	0,80
G protein-coupled receptor 50	GPR50	0,80
solute carrier family 10 (sodium/bile acid cotransporter), member 2	SLC10A2	0,80
tubulin tyrosine ligase-like family member 10	TTLL10	0,80
complement component 7	C7	0,80
PTPN13-like, Y-linked; PTPN13-like, Y-linked 2	PRY; PRY2	0,80
ATP binding cassette subfamily C member 10	ABCC10	0,80

guanine nucleotide binding protein (G protein), gamma 3	GNG3	0,80
adenylate cyclase 8 (brain)	ADCY8	0,80
Memczak2013 ANTISENSE, coding, INTERNAL, UTR5 best transcript NM_003494; Transcript Identified by AceView	DYSF; kydor	0,80
small integral membrane protein 3	SMIM3	0,80
brain and acute leukemia, cytoplasmic	BAALC	0,80
allograft inflammatory factor 1	AIF1	0,80
quiescin Q6 sulfhydryl oxidase 1	QSOX1	0,80
ankyrin repeat domain 42	ANKRD42	0,80
pseudouridylate synthase 10	PUS10	0,80
Transcript Identified by AceView, Entrez Gene ID(s) 6764	ST5	0,80
wingless-type MMTV integration site family, member 7B	WNT7B	0,80
UDP-GlcNAc:betaGal beta-1,3-N-acetylglucosaminyltransferase 5	B3GNT5	0,80
epoxide hydrolase 3	EPHX3	0,80
HID1 domain containing	HID1	0,80
desmin	DES	0,80
hexokinase 2	HK2	0,80
BCL2/adenovirus E1B 19kDa interacting protein 3-like	BNIP3L	0,80
RanBP-type and C3HC4-type zinc finger containing 1	RBCK1	0,80
dual specificity phosphatase 10	DUSP10	0,80
serpin peptidase inhibitor, clade B (ovalbumin), member 13	SERPINB13	0,80
sperm associated antigen 17	SPAG17	0,80
DnaJ (Hsp40) homolog, subfamily C, member 14	DNAJC14	0,80
recombination activating gene 1	RAG1	0,80
smoothelin-like 2	SMTNL2	0,80
prolyl 3-hydroxylase 3	P3H3	0,80
kinesin family member 15	KIF15	0,80
synaptosome associated protein 25kDa	SNAP25	0,80
one cut homeobox 2	ONECUT2	0,80
solute carrier family 16 (monocarboxylate transporter), member 3; microRNA 6787	SLC16A3; MIR6787	0,80
serpin peptidase inhibitor, clade A (alpha-1 antiproteinase, antitrypsin), member 11	SERPINA11	0,80
DBF4 zinc finger	DBF4	0,80
ATPase family, AAA domain containing 2	ATAD2	0,80
lin-7 homolog B (C. elegans)	LIN7B	0,80
solute carrier family 6 (neutral amino acid transporter), member 18	SLC6A18	0,80
olfactory receptor, family 8, subfamily K, member 1	OR8K1	0,80
insulin induced gene 2	INSIG2	0,80
chromosome 9 open reading frame 57	C9orf57	0,80
sema domain, immunoglobulin domain (Ig), short basic domain, secreted, (semaphorin) 3B; microRNA 6872	SEMA3B; MIR6872	0,80
insulin-like growth factor 2	IGF2	0,80

FK506 binding protein 14	FKBP14	0,80
APC membrane recruitment protein 1	AMER1	0,80
KIAA1257	KIAA1257	0,80
solute carrier family 26 (anion exchanger), member 8	SLC26A8	0,80
leucine rich adaptor protein 1	LURAP1	0,80
olfactory receptor, family 12, subfamily D, member 1 (gene/pseudogene) [Source:HGNC Symbol;Acc:HGNC:8177]	OR12D1	0,80
chronic lymphocytic leukemia up-regulated 1 opposite strand	CLLU1OS	0,80
family with sequence similarity 19 (chemokine (C-C motif)-like), member A5	FAM19A5	0,80
ribosomal protein S6 kinase, 52kDa, polypeptide 1	RPS6KC1	0,80
olfactory receptor, family 7, subfamily G, member 1	OR7G1	0,80
solute carrier family 16 (monocarboxylate transporter), member 8	SLC16A8	0,80
laminin, beta 2 (laminin S)	LAMB2	0,80
proline-rich basic protein 1	PROB1	0,80
complement component (3b/4b) receptor 1-like	CR1L	0,80
solute carrier family 6 (amino acid transporter), member 14	SLC6A14	0,80
melanoma antigen family D4; melanoma antigen family D4B; small nucleolar RNA, H/ACA box 11D; small nucleolar RNA, H/ACA box 11E	MAGED4; MAGED4B; SNORA11D; SNORA11E	0,80
zinc finger protein 335	ZNF335	0,80
ArfGAP with SH3 domain, ankyrin repeat and PH domain 2	ASAP2	0,80
melanocortin 2 receptor accessory protein	MRAP	0,80
mitogen-activated protein kinase kinase kinase 3	MAP3K3	0,80
PHD finger protein 1	PHF1	0,80
ribosomal RNA processing 8, methyltransferase, homolog (yeast)	RRP8	0,80
carboxypeptidase N, polypeptide 1	CPN1	0,80
mannosyl (alpha-1,6-)-glycoprotein beta-1,2-N-acetylglucosaminyltransferase	MGAT2	0,80
RNA binding motif, single stranded interacting protein 1; microRNA 4785	RBMS1; MIR4785	0,80
melanoma antigen family D4B; melanoma antigen family D4; small nucleolar RNA, H/ACA box 11D	MAGED4B; MAGED4; SNORA11D	0,80
family with sequence similarity 46, member C	FAM46C	0,80
transcription elongation factor B (SIII), polypeptide 2 (18kDa, elongin B)	TCEB2	0,80
leucine rich repeat containing 53	LRRC53	0,80
sialic acid binding Ig-like lectin 1, sialoadhesin	SIGLEC1	0,80
potassium channel tetramerization domain containing 19	KCTD19	0,80
tumor suppressor candidate 1	TUSC1	0,80

Memczak2013 ANTISENSE, CDS, coding, INTERNAL best transcript NM_005319	HIST1H1C	0,80
glutamic-oxaloacetic transaminase 1-like 1	GOT1L1	0,80
phospholipase A2, group IVA (cytosolic, calcium-dependent)	PLA2G4A	0,80
yippee like 1	YPEL1	0,80
olfactory receptor, family 4, subfamily D, member 9	OR4D9	0,80
coiled-coil domain containing 190	CCDC190	0,80
transmembrane protein 86A	TMEM86A	0,80
WAP four-disulfide core domain 10A	WFDC10A	0,80
Pim-2 proto-oncogene, serine/threonine kinase	PIM2	0,80
Memczak2013 ALT_ACCEPTOR, ALT_DONOR, coding, INTERNAL, intronic best transcript NM_004972	JAK2	0,80
TBC1 domain family, member 30	TBC1D30	0,80
distal-less homeobox 2	DLX2	0,80
olfactory receptor, family 2, subfamily Y, member 1	OR2Y1	0,80
DDB1 and CUL4 associated factor 8-like 1	DCAF8L1	0,80
paired box 9	PAX9	0,80
cellular repressor of E1A-stimulated genes 2	CREG2	0,80
kelch-like family member 1	KLHL1	0,80
arylsulfatase family, member K	ARSK	0,80
integrin alpha 2b	ITGA2B	0,79
RAD21 cohesin complex component like 1	RAD21L1	0,79
olfactory receptor, family 52, subfamily N, member 4 (gene/pseudogene)	OR52N4	0,79
CASP2 and RIPK1 domain containing adaptor with death domain	CRADD	0,79
long intergenic non-protein coding RNA 649	LINC00649	0,79
repetin	RPTN	0,79
equatorin, sperm acrosome associated	EQTN	0,79
pleckstrin and Sec7 domain containing 4	PSD4	0,79
sialidase 3 (membrane sialidase)	NEU3	0,79
coiled-coil domain containing 142; mitochondrial ribosomal protein L53	CCDC142; MRPL53	0,79
EF-hand domain family member D2	EFHD2	0,79
AhpC/TSA antioxidant enzyme domain containing 1	AAED1	0,79
GATA binding protein 2	GATA2	0,79
mitogen-activated protein kinase kinase 5	MAP2K5	0,79
agouti related neuropeptide	AGRP	0,79
nudix hydrolase 18	NUDT18	0,79
colony stimulating factor 3 receptor	CSF3R	0,79
GRIP and coiled-coil domain containing 2	GCC2	0,79
Transcript Identified by AceView, Entrez Gene ID(s) 9659	PDE4DIP	0,79
transmembrane protein 161A	TMEM161A	0,79
Jeck2013 ALT_ACCEPTOR, ALT_DONOR, coding, INTERNAL, intronic best transcript NM_004787	SLIT2	0,79
armadillo repeat containing 12	ARMC12	0,79

complexin 4	CPLX4	0,79
contactin associated protein-like 3B	CNTNAP3B	0,79
olfactory receptor, family 52, subfamily N, member 2	OR52N2	0,79
galactokinase 2	GALK2	0,79
sideroflexin 3	SFXN3	0,79
serum/glucocorticoid regulated kinase family, member 3; C8orf44-SGK3 readthrough	SGK3; C8orf44-SGK3	0,79
V-set and immunoglobulin domain containing 10 like	VSIG10L	0,79
MUS81 structure-specific endonuclease subunit	MUS81	0,79
InaF-motif containing 2	INAFM2	0,79
achaete-scute family bHLH transcription factor 3	ASCL3	0,79
solute carrier family 2 (facilitated glucose transporter), member 10	SLC2A10	0,79
BTB (POZ) domain containing 11	BTBD11	0,79
uncharacterized LOC100129697; novel transcript, antisense to CBFA2T3	LOC100129697; RP11-830F9.6	0,79
zinc finger protein 35	ZNF35	0,79
Transcript Identified by AceView, Entrez Gene ID(s) 10371	SEMA3A	0,79
DIRAS family, GTP-binding RAS-like 1	DIRAS1	0,79
myeloma overexpressed 2	MYEOV2	0,79
RAD51 associated protein 2	RAD51AP2	0,79
von Willebrand factor C and EGF domains	VWCE	0,79
cell migration inducing protein, hyaluronan binding	CEMIP	0,79
family with sequence similarity 98, member C	FAM98C	0,79
solute carrier family 25 (mitochondrial oxoadipate carrier), member 21	SLC25A21	0,79
TBC1 domain family, member 12	TBC1D12	0,79
Transcript Identified by AceView, Entrez Gene ID(s) 84312	BRMS1L	0,79
mitogen-activated protein kinase 6	MAPK6	0,79
ubiquitin specific peptidase 38	USP38	0,79
aminoacylase 1	ACY1	0,79
aldolase C, fructose-bisphosphate	ALDOC	0,79
insulin receptor	INSR	0,79
double homeobox 1	DUX1	0,79
Transcript Identified by AceView, Entrez Gene ID(s) 10978; novel transcript, sense overlapping CLP1	CLP1; AP000662.4	0,79
serpin peptidase inhibitor, clade B (ovalbumin), member 9	SERPINB9	0,79
zinc finger protein 573	ZNF573	0,79
wingless-type MMTV integration site family, member 3	WNT3	0,79
triadin	TRDN	0,79
adenylate cyclase 10 (soluble)	ADCY10	0,79
olfactory receptor, family 6, subfamily B, member 1	OR6B1	0,79
myosin XVI	MYO16	0,79
ADAM metallopeptidase with thrombospondin type 1 motif 13	ADAMTS13	0,79
transmembrane protein 255A	TMEM255A	0,79

abhydrolase domain containing 11	ABHD11	0,79
hydroxymethylbilane synthase	HMBS	0,79
synovial sarcoma translocation gene on chromosome 18-like 1	SS18L1	0,79
histone cluster 2, H3a; histone cluster 2, H3c	HIST2H3A; HIST2H3C	0,79
DCN1, defective in cullin neddylation 1, domain containing 1	DCUN1D1	0,79
calmegin	CLGN	0,79
centromere protein A	CENPA	0,79
doublesex and mab-3 related transcription factor 1	DMRT1	0,79
immunoglobin superfamily, member 21	IGSF21	0,79
gap junction protein gamma 1	GJC1	0,79
trichorhinophalangeal syndrome I	TRPS1	0,79
Memczak2013 ANTISENSE, CDS, coding, INTERNAL best transcript NM_012433	SF3B1	0,79
DnaJ (Hsp40) homolog, subfamily C, member 9	DNAJC9	0,79
Transcript Identified by AceView, Entrez Gene ID(s) 998	CDC42	0,79
leucine twenty homeobox	LEUTX	0,79
Sp5 transcription factor	SP5	0,79
hes family bHLH transcription factor 6	HES6	0,79
AT rich interactive domain 5B (MRF1-like)	ARID5B	0,79
lipoic acid synthetase	LIAS	0,79
interferon induced transmembrane protein 1	IFITM1	0,79
autophagy related 9B	ATG9B	0,79
F-box protein 38	FBXO38	0,79
epidermal growth factor receptor pathway substrate 15-like 1	EPS15L1	0,79
calcium channel, voltage-dependent, beta subunit associated regulatory protein	CBARP	0,79
ELKS/RAB6-interacting/CAST family member 1	ERC1	0,79
FXYD domain containing ion transport regulator 6; FXYD6-FXYD2 readthrough; FXYD domain containing ion transport regulator 2	FXYD6; FXYD6-FXYD2; FXYD2	0,79
interleukin 17F	IL17F	0,79
Jeck2013 ANTISENSE, CDS, coding, INTERNAL, OVCODE, OVEXON best transcript NM_130803	MEN1	0,79
cornichon family AMPA receptor auxiliary protein 2	CNIH2	0,79
polypeptide N-acetylgalactosaminyltransferase 1	GALNT1	0,79
long intergenic non-protein coding RNA 982	LINC00982	0,79
Transcript Identified by AceView, Entrez Gene ID(s) 2890	GRIA1	0,79
tumor-associated calcium signal transducer 2	TACSTD2	0,79
glycine amidinotransferase (L-arginine:glycine amidinotransferase)	GATM	0,79
transient receptor potential cation channel, subfamily V, member 1	TRPV1	0,79
cell division cycle 37-like 1	CDC37L1	0,79
inhibitor of DNA binding 1, dominant negative helix-loop-helix protein	ID1	0,79

v-crk avian sarcoma virus CT10 oncogene homolog	CRK	0,79
HECT and RLD domain containing E3 ubiquitin protein ligase 5	HERC5	0,79
olfactory receptor, family 8, subfamily G, member 2	OR8G2	0,79
WD repeat domain 47	WDR47	0,79
cytochrome P450, family 11, subfamily B, polypeptide 2	CYP11B2	0,79
ring finger protein 1	RING1	0,79
HESX homeobox 1	HESX1	0,79
regulator of G-protein signaling 4	RGS4	0,79
transmembrane protein 229B	TMEM229B	0,79
tetraspanin 11	TSPAN11	0,79
histone cluster 1, H2bc	HIST1H2BC	0,79
ZW10 interacting kinetochore protein	ZWINT	0,79
NLR family, pyrin domain containing 10	NLRP10	0,79
inositol polyphosphate-5-phosphatase K	INPP5K	0,79
coiled-coil domain containing 39	CCDC39	0,79
ubiquitin specific peptidase 17-like family member 17	USP17L17	0,79
nicotinamide nucleotide adenylyltransferase 2	NMNAT2	0,79
pancreatic lipase-related protein 3	PNLIPRP3	0,79
rabaptin, RAB GTPase binding effector protein 2	RABEP2	0,79
CCR4-NOT transcription complex subunit 8	CNOT8	0,79
melanin-concentrating hormone receptor 2	MCHR2	0,79
defensin, alpha 6, Paneth cell-specific	DEFA6	0,79
hedgehog acyltransferase	HHAT	0,79
transcription elongation regulator 1-like	TCERG1L	0,79
cell death-inducing DFFA-like effector a	CIDEA	0,79
zinc finger and BTB domain containing 4	ZBTB4	0,79
TRAF-interacting protein with forkhead-associated domain	TIFA	0,78
secretory carrier membrane protein 4; adenosine deaminase, tRNA-specific 3	SCAMP4; ADAT3	0,78
cytochrome b561 family, member D2	CYB561D2	0,78
WAP four-disulfide core domain 5	WFDC5	0,78
hypoxia up-regulated 1	HYOU1	0,78
potassium channel tetramerization domain containing 10	KCTD10	0,78
olfactory receptor, family 1, subfamily N, member 1	OR1N1	0,78
roundabout guidance receptor 2	ROBO2	0,78
monoacylglycerol O-acyltransferase 2	MOGAT2	0,78
zinc finger protein 99	ZNF99	0,78
Jeck2013 ALT_ACCEPTOR, ALT_DONOR, coding, INTERNAL, intronic best transcript NM_005119	THRAP3	0,78
putative uncharacterized protein MGC39545; novel transcript, antisense to FEZ1	LOC403312; AP000708.1	0,78
zinc finger protein 716	ZNF716	0,78
EF-hand calcium binding domain 11	EFCAB11	0,78
RAR-related orphan receptor A	RORA	0,78
leucine rich repeat and Ig domain containing 4	LINGO4	0,78

ARRDC1 antisense RNA 1	ARRDC1-AS1	0,78
surfeit 1	SURF1	0,78
non-SMC condensin II complex subunit D3	NCAPD3	0,78
zinc finger, DBF-type containing 2	ZDBF2	0,78
miR-17-92 cluster host gene; microRNA 17; microRNA 18a; microRNA 19a; microRNA 19b-1; microRNA 20a; microRNA 92a-1	MIR17HG; MIR17; MIR18A; MIR19A; MIR19B1; MIR20A; MIR92A1	0,78
zinc finger and SCAN domain containing 12	ZSCAN12	0,78
apolipoprotein M	APOM	0,78
tumor necrosis factor, alpha-induced protein 8	TNFAIP8	0,78
acyl-CoA synthetase family member 3	ACSF3	0,78
surfeit 1	SURF1	0,78
transmembrane protein 45A	TMEM45A	0,78
speckle-type POZ protein	SPOP	0,78
tetratricopeptide repeat domain 23	TTC23	0,78
lymphocyte cytosolic protein 2	LCP2	0,78
CDC like kinase 1	CLK1	0,78
transmembrane protein 229B	TMEM229B	0,78
melanocortin 4 receptor	MC4R	0,78
Cbl proto-oncogene C, E3 ubiquitin protein ligase	CBLC	0,78
family with sequence similarity 127, member B	FAM127B	0,78
phosphoglycerate kinase 1	PGK1	0,78
inositol polyphosphate-5-phosphatase D	INPP5D	0,78
developmental pluripotency associated 3	DPPA3	0,78
achaete-scute family bHLH transcription factor 4	ASCL4	0,78
cleavage and polyadenylation specific factor 4-like	CPSF4L	0,78
PALM2-AKAP2 readthrough	PALM2-AKAP2	0,78
RIMS binding protein 3C	RIMBP3C	0,78
neurotrimin	NTM	0,78
adaptor-related protein complex 5, beta 1 subunit	AP5B1	0,78
zinc finger protein 674	ZNF674	0,78
folate receptor 1 (adult)	FOLR1	0,78
ceramide synthase 3	CERS3	0,78
Jeck2013 ALT_ACCEPTOR, ALT_DONOR, coding, INTERNAL, intronic best transcript NM_134261	RORA	0,78
chromosome 9 open reading frame 66	C9orf66	0,78
exoribonuclease 1	ERI1	0,78
lymphocyte antigen 96	LY96	0,78
zinc finger protein 615	ZNF615	0,78
aquaporin 6, kidney specific	AQP6	0,78
hydroxysteroid (17-beta) dehydrogenase 13	HSD17B13	0,78

X-linked Kx blood group	XK	0,78
natriuretic peptide B	NPPB	0,78
paraoxonase 1	PON1	0,78
Fc receptor, IgA, IgM, high affinity	FCAMR	0,78
fatty acid binding protein 7, brain	FABP7	0,78
coproporphyrinogen oxidase	CPOX	0,78
Memczak2013 ANTISENSE, coding, INTERNAL, UTR3 best transcript NM_001428	ENO1	0,78
rearranged L-myc fusion	RLF	0,78
transmembrane protein 45B	TMEM45B	0,78
trafficking protein particle complex 4	TRAPPC4	0,78
ubiquitin-like modifier activating enzyme 6	UBA6	0,78
histone deacetylase 11	HDAC11	0,78
zinc finger protein 514	ZNF514	0,78
zinc finger, DHHC-type containing 15	ZDHHC15	0,78
cell division cycle 45	CDC45	0,78
ecto-NOX disulfide-thiol exchanger 2	ENOX2	0,78
solute carrier family 14 (urea transporter), member 1 (Kidd blood group)	SLC14A1	0,78
matrix-remodelling associated 8	MXRA8	0,78
MAN1B1 antisense RNA 1 (head to head)	MAN1B1-AS1	0,78
sorting nexin 12	SNX12	0,78
solute carrier family 2 (facilitated glucose/fructose transporter), member 5	SLC2A5	0,78
leucine rich repeat containing 74B	LRRC74B	0,78
translocase of outer mitochondrial membrane 20 homolog (yeast)	TOMM20	0,78
asparaginase like 1	ASRGL1	0,78
uncharacterized LOC101928694; novel transcript, antisense to LIPC	LOC101928694; RP11-355N15.1	0,78
Memczak2013 ALT_ACCEPTOR, ALT_DONOR, coding, INTERNAL, intronic best transcript NM_020397	CAMK1D	0,78
cadherin 8, type 2	CDH8	0,78
tissue factor pathway inhibitor 2	TFPI2	0,78
F-box protein 39	FBXO39	0,78
spire-type actin nucleation factor 1	SPIRE1	0,78
CTAGE family, member 15	CTAGE15	0,78
macrophage scavenger receptor 1	MSR1	0,78
ATP binding cassette subfamily A member 12	ABCA12	0,78
solute carrier family 28 (concentrative nucleoside transporter), member 3	SLC28A3	0,78
Na+/K+ transporting ATPase interacting 4	NKAIN4	0,78
collagen, type XVI, alpha 1	COL16A1	0,78
acyl-CoA thioesterase 1	ACOT1	0,78

Fanconi anemia complementation group I	FANCI	0,78
akirin 1	AKIRIN1	0,78
ISL LIM homeobox 1	ISL1	0,78
apolipoprotein B mRNA editing enzyme, catalytic polypeptide-like 3B	APOBEC3B	0,78
ubiquitin specific peptidase 17-like family member 4	USP17L4	0,78
zinc finger protein 215	ZNF215	0,78
integrin alpha 9	ITGA9	0,78
somatostatin	SST	0,78
matrix-remodelling associated 5	MXRA5	0,78
F-box and leucine-rich repeat protein 19	FBXL19	0,78
CD40 ligand	CD40LG	0,78
olfactory receptor, family 5, subfamily AS, member 1	OR5AS1	0,78
cyclin E2	CCNE2	0,78
gamma-aminobutyric acid (GABA) A receptor, epsilon; microRNA 224; microRNA 452	GABRE; MIR224; MIR452	0,78
zinc finger protein 358	ZNF358	0,78
family with sequence similarity 151, member A	FAM151A	0,78
zinc finger, CCHC domain containing 10	ZCCHC10	0,77
alkB homolog 7	ALKBH7	0,77
integrin, alpha 2 (CD49B, alpha 2 subunit of VLA-2 receptor)	ITGA2	0,77
xylosylprotein beta 1,4-galactosyltransferase, polypeptide 7	B4GALT7	0,77
olfactory receptor, family 4, subfamily C, member 16 (gene/pseudogene)	OR4C16	0,77
round spermatid basic protein 1	RSBN1	0,77
ATH1, acid trehalase-like 1 (yeast)	ATHL1	0,77
meiosis specific with OB domains; long intergenic non-protein coding RNA 254	MEIOB; LINC00254	0,77
mannan-binding lectin serine peptidase 1 (C4/C2 activating component of Ra-reactive factor)	MASP1	0,77
Yip1 domain family member 2	YIPF2	0,77
ribosomal modification protein rimK-like family member A	RIMKLA	0,77
Transcript Identified by AceView, Entrez Gene ID(s) 55604	LRRC16A	0,77
proline rich 12	PRR12	0,77
Homo sapiens serine/threonine kinase 19 (STK19), transcript variant 1, mRNA.; Homo sapiens serine/threonine kinase 19 (STK19), transcript variant 2, mRNA.; Homo sapiens serine/threonine kinase 19 (STK19), transcript variant 3, non-coding RNA.; Homo sapiens serine/threonine kinase 19, mRNA (cDNA clone MGC:117388 IMAGE:5165123), complete cds.	STK19	0,77
apolipoprotein F	APOF	0,77
olfactory receptor, family 10, subfamily J, member 1	OR10J1	0,77
tumor necrosis factor receptor superfamily, member 10d, decoy with truncated death domain	TNFRSF10D	0,77

spindlin family, member 4	SPIN4	0,77
hydroxyprostaglandin dehydrogenase 15-(NAD)	HPGD	0,77
UV radiation resistance associated	UVRAG	0,77
defensin, beta 105B; defensin, beta 105A	DEFB105B; DEFB105A	0,77
ATP binding cassette subfamily A member 13	ABCA13	0,77
zinc finger, DHHC-type containing 20	ZDHHC20	0,77
chromosome 4 open reading frame 36	C4orf36	0,77
aldolase B, fructose-bisphosphate	ALDOB	0,77
GTP binding protein 1	GTPBP1	0,77
potassium channel tetramerization domain containing 16	KCTD16	0,77
ADAM metallopeptidase domain 30	ADAM30	0,77
Transcript Identified by AceView, Entrez Gene ID(s) 8425	LTBP4	0,77
GPN-loop GTPase 3	GPN3	0,77
serum/glucocorticoid regulated kinase 1	SGK1	0,77
Memczak2013 ANTISENSE, coding, INTERNAL, intronic best transcript NM_020821	VPS13C	0,77
Transcript Identified by AceView, Entrez Gene ID(s) 10777	ARPP21	0,77
vertebrae development associated	VRTN	0,77
GTP binding protein 6 (putative)	GTPBP6	0,77
thrombospondin type 1 domain containing 7B	THSD7B	0,77
vacuolar protein sorting 51 homolog (S. cerevisiae)	VPS51	0,77
chromosome 17 open reading frame 100	C17orf100	0,77
Fas-activated serine/threonine kinase	FASTK	0,77
Eukaryotic translation initiation factor 3 subunit F [Source:UniProtKB/Swiss-Prot;Acc:O00303]	EIF3F	0,77
olfactory receptor, family 6, subfamily X, member 1	OR6X1	0,77
sulfotransferase family 2A member 1	SULT2A1	0,77
selenoprotein P, plasma, 1	SEPP1	0,77
SUMO1/sentrin specific peptidase 6	SENP6	0,77
synaptojanin 1	SYNJ1	0,77
WAP four-disulfide core domain 10B	WFDC10B	0,77
protein phosphatase 1, regulatory subunit 18	PPP1R18	0,77
peroxisome proliferator-activated receptor gamma	PPARG	0,77
cortexin 3	CTXN3	0,77
chromosome 16 open reading frame 91	C16orf91	0,77
olfactory receptor, family 2, subfamily T, member 3	OR2T3	0,77
collagen, type V, alpha 3	COL5A3	0,77
olfactory receptor, family 1, subfamily L, member 6	OR1L6	0,77
obscurin-like 1	OBSL1	0,77
seven transmembrane helix receptor; Transcript Identified by AceView, Entrez Gene ID(s) 650293, RefSeq ID(s) NM_001040071	LOC650293	0,77
solute carrier family 16, member 2 (thyroid hormone transporter)	SLC16A2	0,77

MIR22 host gene	MIR22HG	0,77
Transcript Identified by AceView, Entrez Gene ID(s) 23126	POGZ	0,77
guanine nucleotide binding protein-like 1	GNL1	0,77
lactase	LCT	0,77
pituitary tumor-transforming 2	PTTG2	0,77
histidine ammonia-lyase	HAL	0,77
ribosomal protein S25	RPS25	0,77
KIAA0825	KIAA0825	0,77
AGPAT4 intronic transcript 1	AGPAT4-IT1	0,77
RAD51 recombinase	RAD51	0,77
olfactory receptor, family 6, subfamily C, member 4	OR6C4	0,77
Rho guanine nucleotide exchange factor 39	ARHGEF39	0,77
crystallin gamma C	CRYGC	0,77
plexin A3	PLXNA3	0,77
N-acetylglucosaminidase, alpha	NAGLU	0,77
primary cilia formation	PIFO	0,77
glutathione S-transferase mu 2 (muscle)	GSTM2	0,77
collagen, type XI, alpha 2	COL11A2	0,77
B-cell CLL/lymphoma 7B	BCL7B	0,77
ZFP3 zinc finger protein	ZFP3	0,77
interleukin 1 receptor-like 1	IL1RL1	0,77
serpin peptidase inhibitor, clade G (C1 inhibitor), member 1	SERPING1	0,77
exosome component 6	EXOSC6	0,77
ARP8 actin-related protein 8 homolog (yeast)	ACTR8	0,77
Memczak2013 ALT_ACCEPTOR, ALT_DONOR, coding, INTERNAL, intronic best transcript NM_003574	VAPA	0,77
perilipin 2	PLIN2	0,77
cystic fibrosis transmembrane conductance regulator	CFTR	0,77
Memczak2013 ANTISENSE, coding, INTERNAL, intronic best transcript NM_001202514	MXD1	0,77
Transcript Identified by AceView, Entrez Gene ID(s) 23126	POGZ	0,77
tet methylcytosine dioxygenase 1	TET1	0,77
runt-related transcription factor 1; translocated to, 1 (cyclin D-related)	RUNX1T1	0,77
lactotransferrin	LTF	0,77
cilia and flagella associated protein 74	CFAP74	0,77
histone cluster 1, H2bi	HIST1H2BI	0,77
interleukin 1 receptor accessory protein-like 1	IL1RAPL1	0,76
neurotrophic tyrosine kinase, receptor, type 3	NTRK3	0,76
Transcript Identified by AceView, Entrez Gene ID(s) 138050	HGSNAT	0,76
deoxyhypusine hydroxylase/monooxygenase	DOHH	0,76
Ras homolog enriched in brain like 1	RHEBL1	0,76
Fc fragment of IgM receptor	FCMR	0,76
purinergic receptor P2X, ligand gated ion channel, 2	P2RX2	0,76
pleckstrin homology domain containing, family O member 1	PLEKHO1	0,76

monoglyceride lipase	MGLL	0,76
zinc finger protein 292	ZNF292	0,76
mucin 22	MUC22	0,76
catenin (cadherin-associated protein), alpha 2	CTNNA2	0,76
epiregulin	EREG	0,76
cell division cycle associated 3	CDCA3	0,76
non-SMC condensin II complex subunit G2	NCAPG2	0,76
cathepsin K	CTSK	0,76
lipase, family member N	LIPN	0,76
Rho GTPase activating protein 24	ARHGAP24	0,76
reticulon 4 receptor-like 2	RTN4RL2	0,76
Jeck2013 ANTISENSE, coding, INTERNAL, intronic best transcript NM_003380	VIM	0,76
syntaxin binding protein 5 (tomosyn)	STXBP5	0,76
F-box protein 17	FBXO17	0,76
SR-related CTD-associated factor 11	SCAF11	0,76
melanoma inhibitory activity; RAB4B, member RAS oncogene family; MIA-RAB4B readthrough (NMD candidate)	MIA; RAB4B; MIA-RAB4B	0,76
solute carrier family 35, member G2	SLC35G2	0,76
zinc finger protein 672	ZNF672	0,76
glutathione peroxidase 4	GPX4	0,76
cytochrome P450, family 2, subfamily U, polypeptide 1	CYP2U1	0,76
Salzman2013 ALT_ACCEPTOR, ALT_DONOR, coding, INTERNAL, intronic best transcript NM_004540	NCAM2	0,76
ephrin-A1	EFNA1	0,76
hydroxymethylbilane synthase	HMBS	0,76
chromosome 10 open reading frame 53	C10orf53	0,76
transmembrane protein 86B	TMEM86B	0,76
chromosome 14 open reading frame 28	C14orf28	0,76
KIAA2026	KIAA2026	0,76
CENPB DNA-binding domain containing 1	CENPBD1	0,76
Memczak2013 ALT_ACCEPTOR, ALT_DONOR, coding, INTERNAL, intronic best transcript NM_152701	ABCA13	0,76
trichoplein, keratin filament binding	TCHP	0,76
ADP-ribosylation factor guanine nucleotide-exchange factor 1 (brefeldin A-inhibited)	ARFGEF1	0,76
solute carrier organic anion transporter family, member 1A2	SLCO1A2	0,76
ninjurin 2	NINJ2	0,76
ATP binding cassette subfamily D member 4	ABCD4	0,76
formin 1	FMN1	0,76
CDP-diacylglycerol synthase 1	CDS1	0,76
nerve growth factor receptor	NGFR	0,76
short chain dehydrogenase/reductase family 16C, member 5	SDR16C5	0,76
F-box and leucine-rich repeat protein 21 (gene/pseudogene)	FBXL21	0,76
cilia and flagella associated protein 126	CFAP126	0,76

lipin 3	LPIN3	0,76
NADH dehydrogenase (ubiquinone) 1 alpha subcomplex, 10, 42kDa	NDUFA10	0,76
prominin 1	PROM1	0,76
integrin beta 7	ITGB7	0,76
zinc finger protein 35	ZNF35	0,76
ecotropic viral integration site 5	EVI5	0,76
tensin 4	TNS4	0,76
platelet-derived growth factor receptor, beta polypeptide	PDGFRB	0,76
frizzled class receptor 6	FZD6	0,76
anomalous homeobox	ANHX	0,76
WAP, follistatin/kazal, immunoglobulin, kunitz and netrin domain containing 1	WFIKKN1	0,76
uncharacterized LOC399886; Transcript Identified by AceView, Entrez Gene ID(s) 399886; novel transcript	LOC399886; RP11-430H10.2; FLJ41423	0,76
fukutin	FKTN	0,76
Down syndrome critical region 8	DSCR8	0,76
Memczak2013 ANTISENSE, coding, INTERNAL, UTR3 best transcript NM_175920	LNPEP	0,76
solute carrier family 43, member 3	SLC43A3	0,76
KIAA1024	KIAA1024	0,76
histone cluster 3, H2a	HIST3H2A	0,76
diacylglycerol kinase, delta 130kDa	DGKD	0,76
chromosome 21 open reading frame 62	C21orf62	0,76
NUT family member 2A	NUTM2A	0,76
zwilch kinetochore protein	ZWILCH	0,76
cilia and flagella associated protein 221	CFAP221	0,76
maternal embryonic leucine zipper kinase	MELK	0,76
zinc finger protein 213	ZNF213	0,76
scavenger receptor class A, member 5	SCARA5	0,76
tumor suppressor candidate 3	TUSC3	0,76
guanine nucleotide binding protein, alpha transducing 3	GNAT3	0,76
olfactory receptor, family 2, subfamily T, member 6	OR2T6	0,76
chemokine (C-C motif) ligand 16	CCL16	0,76
t-complex 11, testis-specific-like 2	TCP11L2	0,76
defensin, beta 131	DEFB131	0,76
immunoglobulin superfamily, member 1	IGSF1	0,76
mitochondrial ribosomal protein L2	MRPL2	0,76
ZFP36 ring finger protein	ZFP36	0,75
family with sequence similarity 110, member A	FAM110A	0,75
WNK lysine deficient protein kinase 4	WNK4	0,75
zinc finger and SCAN domain containing 32	ZSCAN32	0,75

E74-like factor 3 (ets domain transcription factor, epithelial-specific)	ELF3	0,75
family with sequence similarity 129, member B	FAM129B	0,75
zinc finger protein 846	ZNF846	0,75
aminolevulinate dehydratase	ALAD	0,75
collagen, type XV, alpha 1	COL15A1	0,75
cholesteryl ester transfer protein, plasma	CETP	0,75
transmembrane 9 superfamily member 3	TM9SF3	0,75
protein C receptor, endothelial	PROCR	0,75
PRAME family member 10	PRAMEF10	0,75
LINGO1 antisense RNA 1	LINGO1-AS1	0,75
PTPN13-like, Y-linked pseudogene 4	PRYP4	0,75
Salzman2013 ANNOTATED, CDS, coding, INTERNAL, OVCODE, OVEXON best transcript NM_001201380	CNTNAP3B	0,75
hepatic and glial cell adhesion molecule	HEPACAM	0,75
calcyphosine-like	CAPSL	0,75
zinc finger, CCHC domain containing 11	ZCCHC11	0,75
SAGA complex associated factor 29	SGF29	0,75
DR1-associated protein 1 (negative cofactor 2 alpha)	DRAP1	0,75
CD2 (cytoplasmic tail) binding protein 2	CD2BP2	0,75
solute carrier family 9, subfamily A (NHE4, cation proton antiporter 4), member 4	SLC9A4	0,75
TIMP metallopeptidase inhibitor 4	TIMP4	0,75
apoptotic peptidase activating factor 1	APAF1	0,75
Jeck2013 ALT_ACCEPTOR, ALT_DONOR, coding, INTERNAL, intronic best transcript NM_015049	TRAK2	0,75
prolyl 4-hydroxylase, alpha polypeptide I	P4HA1	0,75
ubiquitin specific peptidase 37	USP37	0,75
membrane-spanning 4-domains, subfamily A, member 15	MS4A15	0,75
Transcript Identified by AceView, Entrez Gene ID(s) 26084	SGEF	0,75
prostaglandin D2 receptor 2	PTGDR2	0,75
Transcript Identified by AceView, Entrez Gene ID(s) 4683	NBN	0,75
UPF0607 protein ENSP00000381418-like; Putative UPF0607 protein ENSP00000381418 [Source:UniProtKB/Swiss-Prot;Acc:A8MU76]	LOC729159; RP11-457D20.2	0,75
chondroitin sulfate N-acetylgalactosaminyltransferase 1	CSGALNACT1	0,75
LDL receptor related protein 3	LRP3	0,75
zinc finger protein 703	ZNF703	0,75
scinderin	SCIN	0,75
hyperpolarization activated cyclic nucleotide gated potassium channel 2	HCN2	0,75
sideroflexin 2	SFXN2	0,75
succinate dehydrogenase complex assembly factor 4	SDHAF4	0,75
BAI1-associated protein 3	BAIAP3	0,75
ethanolamine kinase 1	ETNK1	0,75

fibromodulin	FMOD	0,75
claudin 6	CLDN6	0,75
Transcript Identified by AceView, Entrez Gene ID(s) 1768	DNAH6	0,75
TBC1 domain family, member 10B	TBC1D10B	0,75
carcinoembryonic antigen-related cell adhesion molecule 4	CEACAM4	0,75
Transcript Identified by AceView, Entrez Gene ID(s) 9229	DLGAP1	0,75
lysine-rich coiled-coil 1	KRCC1	0,75
coiled-coil domain containing 177	CCDC177	0,75
SH3 domain binding kinase 1	SBK1	0,75
tafazzin	TAZ	0,75
SWI5-dependent homologous recombination repair protein 1	SFR1	0,75
olfactory receptor, family 8, subfamily A, member 1	OR8A1	0,75
transmembrane channel like 2	TMC2	0,75
glutamyl aminopeptidase (aminopeptidase A)	ENPEP	0,75
zinc fingers and homeoboxes 2	ZHX2	0,75
ubiquitin specific peptidase 53	USP53	0,75
serpin peptidase inhibitor, clade A (alpha-1 antiproteinase, antitrypsin), member 9	SERPINA9	0,75
cancer antigen 1	CAGE1	0,75
amidohydrolase domain containing 2	AMDHD2	0,74
myosin IG	MYO1G	0,74
Mab-21 domain containing 2	MB21D2	0,74
olfactory receptor, family 2, subfamily G, member 2	OR2G2	0,74
thioredoxin interacting protein	TXNIP	0,74
carbohydrate (chondroitin 4) sulfotransferase 12	CHST12	0,74
breast carcinoma amplified sequence 1	BCAS1	0,74
hemoglobin, alpha 2	HBA2	0,74
BRCA1 associated RING domain 1	BARD1	0,74
gastrin	GAST	0,74
DEAQ box RNA-dependent ATPase 1	DQX1	0,74
glucagon receptor	GCGR	0,74
pecanex-like 4 (Drosophila)	PCNXL4	0,74
homeobox D12	HOXD12	0,74
zinc finger protein 287	ZNF287	0,74
chromosome 10 open reading frame 10	C10orf10	0,74
ripply transcriptional repressor 2	RIPPLY2	0,74
diphthamine biosynthesis 6	DPH6	0,74
ankyrin repeat domain 1 (cardiac muscle)	ANKRD1	0,74
tetraspanin 16	TSPAN16	0,74
natriuretic peptide receptor 3	NPR3	0,74
arginine vasopressin-induced 1	AVPI1	0,74
chromosome 16 open reading frame 62	C16orf62	0,74
hemoglobin, alpha 1	HBA1	0,74
phospholipid phosphatase related 2	PLPPR2	0,74

Memczak2013 ANTISENSE, coding, INTERNAL, UTR3 best transcript NM_000565	IL6R	0,74
heterogeneous nuclear ribonucleoprotein H1 (H)	HNRNPH1	0,74
pro-melanin-concentrating hormone	PMCH	0,74
zinc finger protein 658	ZNF658	0,74
zinc finger, RAN-binding domain containing 2	ZRANB2	0,74
syndecan 3	SDC3	0,74
ras homolog family member H	RHOH	0,74
leucine rich repeat containing 61	LRRC61	0,74
chromosome 5 open reading frame 22	C5orf22	0,74
tetratricopeptide repeat domain 39A	TTC39A	0,74
tumor necrosis factor (ligand) superfamily, member 8	TNFSF8	0,74
SHANK-associated RH domain interactor	SHARPIN	0,74
zinc finger, MYM-type 1	ZMYM1	0,74
ARFGEF family member 3	ARFGEF3	0,74
Jeck2013 ANTISENSE, CDS, coding, INTERNAL, intronic, OVCODE, OVEXON best transcript NM_003131	SRF	0,74
MKRN2 opposite strand	MKRN2OS	0,74
olfactory receptor, family 6, subfamily C, member 74	OR6C74	0,74
angiopoietin like 2	ANGPTL2	0,74
chromosome 2 open reading frame 72	C2orf72	0,74
ubiquitin specific peptidase 17-like family member 22	USP17L22	0,74
solute carrier family 52 (riboflavin transporter), member 1	SLC52A1	0,74
sperm autoantigenic protein 17	SPA17	0,74
family with sequence similarity 83, member G	FAM83G	0,74
UDP-GlcNAc:betaGal beta-1,3-N-acetylglucosaminyltransferase 3	B3GNT3	0,74
phosphoenolpyruvate carboxykinase 1 (soluble)	PCK1	0,74
Transcript Identified by AceView, Entrez Gene ID(s) 85442	KNDC1	0,74
chromosome 8 open reading frame 74	C8orf74	0,74
tetraspanin 12	TSPAN12	0,74
peptidylglycine alpha-amidating monooxygenase	PAM	0,74
lymphocyte antigen 86	LY86	0,74
tumor necrosis factor (ligand) superfamily, member 4	TNFSF4	0,74
chromosome 9 open reading frame 72	C9orf72	0,74
liver expressed antimicrobial peptide 2	LEAP2	0,74
X-ray repair complementing defective repair in Chinese hamster cells 3	XRCC3	0,74
uroplakin 3A	UPK3A	0,74
selenoprotein T; SELT protein; Selenoprotein T; cDNA FLJ90525 fis, clone NT2RP4001001, highly similar to Selenoprotein T [Source:UniProtKB/TrEMBL;Acc:Q6IAK0]; Transcript Identified by AceView, Entrez Gene ID(s) 51714	SELT; SELT.1	0,74
zinc finger protein 540; ZNF571 antisense RNA 1	ZNF540; ZNF571-AS1	0,74

NCK interacting protein with SH3 domain	NCKIPSD	0,74
glutathione peroxidase 3	GPX3	0,74
ankyrin repeat and sterile alpha motif domain containing 1B	ANKS1B	0,74
lectin, galactoside-binding, soluble, 9	LGALS9	0,74
solute carrier family 39 (zinc transporter), member 8	SLC39A8	0,74
hydroxycarboxylic acid receptor 1	HCAR1	0,74
protein tyrosine phosphatase, receptor type, N	PTPRN	0,74
procollagen C-endopeptidase enhancer 2	PCOLCE2	0,74
Transcript Identified by AceView, Entrez Gene ID(s) 116441	TM4SF18	0,74
MYB binding protein (P160) 1a	MYBBP1A	0,74
family with sequence similarity 208, member B	FAM208B	0,74
tyrosylprotein sulfotransferase 1	TPST1	0,74
thimet oligopeptidase 1	THOP1	0,74
interferon regulatory factor 2 binding protein 2	IRF2BP2	0,74
MAGE family member E1	MAGEE1	0,73
abhydrolase domain containing 10	ABHD10	0,73
F-box and leucine-rich repeat protein 12	FBXL12	0,73
CWC25 spliceosome-associated protein homolog	CWC25	0,73
binder of sperm protein homolog 1	BSPH1	0,73
armadillo repeat containing, X-linked 3	ARMCX3	0,73
olfactory receptor, family 4, subfamily X, member 1 (gene/pseudogene)	OR4X1	0,73
Memczak2013 ALT_ACCEPTOR, ALT_DONOR, coding, INTERNAL, intronic best transcript NM_033028	BBS4	0,73
Memczak2013 ANTISENSE, CDS, coding, INTERNAL best transcript NM_001206844	TGOLN2	0,73
olfactory receptor, family 1, subfamily E, member 2	OR1E2	0,73
sperm associated antigen 1	SPAG1	0,73
fasciculation and elongation protein zeta 2 (zygin II)	FEZ2	0,73
synaptotagmin-like 4	SYTL4	0,73
growth hormone 1	GH1	0,73
potassium voltage-gated channel, modifier subfamily S, member 3	KCNS3	0,73
ZNF436 antisense RNA 1	ZNF436-AS1	0,73
Usher syndrome 1C binding protein 1	USHBP1	0,73
guanylate binding protein 1, interferon-inducible	GBP1	0,73
interferon, alpha 16	IFNA16	0,73
golgi-associated, gamma adaptin ear containing, ARF binding protein 1	GGA1	0,73
synaptonemal complex central element protein 3	SYCE3	0,73
DEP domain containing 4	DEPDC4	0,73
hyaluronan binding protein 2	HABP2	0,73
Rap guanine nucleotide exchange factor 4	RAPGEF4	0,73
beta-1,4-N-acetyl-galactosaminyl transferase 4	B4GALNT4	0,73
ring finger protein 183	RNF183	0,73

serine/threonine kinase 32A	STK32A	0,73
platelet factor 4	PF4	0,73
family with sequence similarity 186, member B	FAM186B	0,73
acyl-CoA synthetase long-chain family member 4	ACSL4	0,73
nuclear transport factor 2-like export factor 2	NXT2	0,73
long intergenic non-protein coding RNA 473	LINC00473	0,73
transmembrane protein 260	TMEM260	0,73
ATP binding cassette subfamily D member 1	ABCD1	0,73
solute carrier family 22 (organic anion transporter), member 7	SLC22A7	0,73
pleckstrin homology domain containing, family A (phosphoinositide binding specific) member 2	PLEKHA2	0,73
proteasome subunit alpha 8	PSMA8	0,73
homeobox D1; microRNA 7704	HOXD1; MIR7704	0,73
RELT-like 2	RELL2	0,73
IGF like family member 1	IGFL1	0,73
cytochrome P450, family 19, subfamily A, polypeptide 1	CYP19A1	0,73
zinc finger protein 521	ZNF521	0,73
zinc finger, DHHC-type containing 14	ZDHHC14	0,73
Rho guanine nucleotide exchange factor 10 like	ARHGEF10L	0,73
keratin associated protein 23-1	KRTAP23-1	0,72
DENN/MADD domain containing 6B	DENND6B	0,72
TXK tyrosine kinase	TXK	0,72
leucine rich repeat and coiled-coil centrosomal protein 1	LRRCC1	0,72
zinc finger, CCHC domain containing 13	ZCCHC13	0,72
Homo sapiens GTP binding protein 6 (putative) (GTPBP6), mRNA.; GTP binding protein 6 (putative) [Source:HGNC Symbol;Acc:HGNC:30189]; Homo sapiens GTP binding protein 6 (putative), mRNA (cDNA clone MGC:1170 IMAGE:3528598), complete cds.; Salzman2013 ANNOTATED, CDS, coding, INTERNAL, OVCODE, OVERLAPTX, OVEXON best transcript NM_012227	GTPBP6	0,72
lin-54 DREAM MuvB core complex component	LIN54	0,72
zinc finger, CCHC domain containing 5	ZCCHC5	0,72
G protein-coupled receptor 65	GPR65	0,72
zinc finger, DHHC-type containing 3	ZDHHC3	0,72
solute carrier family 38, member 2	SLC38A2	0,72
ring finger protein 215	RNF215	0,72
SH3 and multiple ankyrin repeat domains 3	SHANK3	0,72
retinoic acid induced 1	RAI1	0,72
family with sequence similarity 13, member A	FAM13A	0,72
Zhang2013 ALT_ACCEPTOR, ALT_DONOR, coding, INTERNAL, intronic best transcript NM_002473	MYH9	0,72
G protein-coupled receptor 83	GPR83	0,72
coiled-coil domain containing 68	CCDC68	0,72

Memczak2013 ALT_ACCEPTOR, ALT_DONOR, INTERNAL, intronic, ncRNA best transcript NR_045602	GALNT14	0,72
chromosome 8 open reading frame 4	C8orf4	0,72
testis, prostate and placenta expressed	TEPP	0,72
gamma-glutamylamine cyclotransferase	GGACT	0,72
EPH receptor A4	EPHA4	0,72
Transcript Identified by AceView, Entrez Gene ID(s) 79677	SMC6	0,72
hydroxycarboxylic acid receptor 3	HCAR3	0,72
lectin, galactoside-binding, soluble, 3	LGALS3	0,72
amelogenin, Y-linked	AMELY	0,72
Memczak2013 ANTISENSE, CDS, coding, INTERNAL best transcript NM_153341	RNF19B	0,72
Jeck2013 ANTISENSE, CDS, coding, INTERNAL, intronic, OVCODE, OVEXON best transcript NM_058175	COL6A2	0,72
chromosome 21 open reading frame 140	C21orf140	0,72
chromosome 21 open reading frame 140	C21orf140	0,72
DDB1 and CUL4 associated factor 12-like 1	DCAF12L1	0,72
TBC1 domain family, member 3 pseudogene 5	TBC1D3P5	0,72
platelet/endothelial cell adhesion molecule 1	PECAM1	0,72
replication factor C subunit 2	RFC2	0,72
immunoglobulin superfamily, member 23	IGSF23	0,72
hes-related family bHLH transcription factor with YRPW motif 1	HEY1	0,71
inositol 1,4,5-trisphosphate receptor interacting protein-like 2	ITPRIPL2	0,71
coiled-coil domain containing 148	CCDC148	0,71
pyruvate dehydrogenase kinase, isozyme 1	PDK1	0,71
TBC1 domain family, member 3K	TBC1D3K	0,71
junctional sarcoplasmic reticulum protein 1	JSRP1	0,71
peptidase domain containing associated with muscle regeneration 1	PAMR1	0,71
lamin tail domain containing 1	LMNTD1	0,71
glutamic-pyruvate transaminase (alanine aminotransferase)	GPT	0,71
WD repeat and SOCS box containing 1	WSB1	0,71
chromosome 18 open reading frame 54	C18orf54	0,71
forkhead box A3	FOXA3	0,71
Transcript Identified by AceView, Entrez Gene ID(s) 5019	OXCT1	0,71
keratin associated protein 10-7; keratin associated protein 10-8	KRTAP10-7; KRTAP10-8	0,71
NUT family member 2D	NUTM2D	0,71
family with sequence similarity 114, member A1	FAM114A1	0,71
solute carrier family 19 (thiamine transporter), member 2	SLC19A2	0,71
FXYD domain containing ion transport regulator 3; microRNA 6887	FXYD3; MIR6887	0,71
Memczak2013 ANTISENSE, CDS, coding, INTERNAL best transcript NM_001127491	ITGB2	0,71
olfactory receptor, family 8, subfamily B, member 12	OR8B12	0,71

hair growth associated	HR	0,71
TRPM8 channel-associated factor 2	TCAF2	0,71
solute carrier family 22, member 10	SLC22A10	0,71
chromosome 20 open reading frame 203	C20orf203	0,71
G protein-coupled receptor 32	GPR32	0,71
Memczak2013 ANTISENSE, CDS, coding, INTERNAL best transcript NM_014931	PPP6R1	0,71
sodium channel, voltage gated, type III alpha subunit	SCN3A	0,71
iroquois homeobox 3	IRX3	0,71
thrombospondin 2	THBS2	0,71
basic helix-loop-helix family, member e40	BHLHE40	0,71
growth arrest and DNA-damage-inducible, beta	GADD45B	0,71
heme oxygenase 1	HMOX1	0,71
cysteinyl leukotriene receptor 1	CYSLTR1	0,71
carboxypeptidase Z; G protein-coupled receptor 78	CPZ; GPR78	0,70
carbonic anhydrase VIII	CA8	0,70
cell growth regulator with EF-hand domain 1	CGREF1	0,70
potassium channel, voltage gated eag related subfamily H, member 2	KCNH2	0,70
zinc finger, DHHC-type containing 19	ZDHHC19	0,70
Transcript Identified by AceView, Entrez Gene ID(s) 9665	KIAA0430	0,70
shroom family member 2	SHROOM2	0,70
immunoglobulin mu binding protein 2	IGHMBP2	0,70
RAB4B, member RAS oncogene family; MIA-RAB4B readthrough (NMD candidate); RAB4B-EGLN2 readthrough (NMD candidate)	RAB4B; MIA-RAB4B; RAB4B-EGLN2	0,70
sialidase 4	NEU4	0,70
splicing factor 1	SF1	0,70
hematopoietically expressed homeobox	HHEX	0,70
cytochrome P450, family 8, subfamily B, polypeptide 1	CYP8B1	0,70
X-linked Kx blood group related 6	XKR6	0,70
integrin beta 8	ITGB8	0,70
olfactory receptor, family 1, subfamily E, member 3 (gene/pseudogene)	OR1E3	0,70
angiotensin II receptor, type 1	AGTR1	0,70
BRCA1 interacting protein C-terminal helicase 1	BRIP1	0,70
olfactory receptor, family 4, subfamily C, member 12	OR4C12	0,70
cerebellin 2 precursor	CBLN2	0,70
zinc finger protein 705D	ZNF705D	0,70
Transcript Identified by AceView, Entrez Gene ID(s) 31	ACACA	0,70
transmembrane phosphatase with tensin homology	TPTE	0,70
tumor protein p53 inducible nuclear protein 2	TP53INP2	0,70
ring finger protein 175	RNF175	0,70
mucolipin 2	MCOLN2	0,70
solute carrier family 39 (zinc transporter), member 10	SLC39A10	0,70

siah E3 ubiquitin protein ligase 1	SIAH1	0,70
TBC1 domain family, member 3G	TBC1D3G	0,69
doublecortin-like kinase 2	DCLK2	0,69
coiled-coil-helix-coiled-coil-helix domain containing 5	CHCHD5	0,69
platelet-activating factor receptor	PTAFR	0,69
sodium channel, voltage gated, type I beta subunit	SCN1B	0,69
histone cluster 2, H4a; histone cluster 2, H4b	HIST2H4A; HIST2H4B	0,69
olfactory receptor, family 8, subfamily B, member 3	OR8B3	0,69
collagen, type IV, alpha 5	COL4A5	0,69
G protein-coupled receptor 15	GPR15	0,69
uronyl-2-sulfotransferase	UST	0,69
NUT family member 2B	NUTM2B	0,69
forkhead box E3	FOXE3	0,69
olfactory receptor, family 2, subfamily T, member 29	OR2T29	0,69
olfactory receptor, family 6, subfamily N, member 2	OR6N2	0,69
dendritic cell-associated nuclear protein	DCANP1	0,69
Transcript Identified by AceView, Entrez Gene ID(s) 1767	DNAH5	0,69
BRCA1 associated protein 1	BAP1	0,69
histone cluster 2, H4b; histone cluster 2, H4a	HIST2H4B; HIST2H4A	0,69
olfactory receptor, family 56, subfamily B, member 1	OR56B1	0,69
Memczak2013 ANTISENSE, coding, INTERNAL, UTR3 best transcript NM_001195737	FAM213B	0,69
leukocyte immunoglobulin-like receptor, subfamily A (with TM domain), member 4	LILRA4	0,69
apolipoprotein L, 2	APOL2	0,69
hCG2028352-like; novel transcript	LOC729970; RP4-639F20.1	0,69
ssu-2 homolog (C. elegans)	SSUH2	0,69
Jeck2013 ALT_ACCEPTOR, ALT_DONOR, coding, INTERNAL, intronic best transcript NM_020177	FEM1C	0,69
transient receptor potential cation channel, subfamily V, member 4	TRPV4	0,69
tenascin N	TNN	0,69
ephrin-A3	EFNA3	0,69
transition protein 1 (during histone to protamine replacement)	TNP1	0,69
interleukin 22 receptor, alpha 1	IL22RA1	0,69
dimethylglycine dehydrogenase	DMGDH	0,68
Memczak2013 ANTISENSE, CDS, coding, INTERNAL best transcript NM_022047	DEF6	0,68
phosphoglycerate mutase family member 4	PGAM4	0,68
Memczak2013 ANTISENSE, coding, INTERNAL, UTR3 best transcript NM_015354	NUP188	0,68
RAB26, member RAS oncogene family	RAB26	0,68

lysine (K)-specific demethylase 3A	KDM3A	0,68
Charcot-Leyden crystal galectin	CLC	0,68
tripartite motif containing 49	TRIM49	0,68
poly(A) binding protein interacting protein 2 [Source:EntrezGene;Acc:51247]; novel transcript, antisense to PAIP2	PAIP2; CTB-43P18.1	0,68
Zhang2013 ALT_ACCEPTOR, ALT_DONOR, coding, INTERNAL, intronic best transcript NM_018198	DNAJC11	0,68
glycerol kinase	GK	0,68
family with sequence similarity 213, member B	FAM213B	0,68
filamin A interacting protein 1-like	FILIP1L	0,68
BCL2-related ovarian killer	BOK	0,68
large tumor suppressor kinase 2	LATS2	0,68
Memczak2013 ANTISENSE, CDS, coding, downstream_end, upstream_start, UTR3, UTR5 best transcript NM_004065; CDR1 antisense RNA; CDR1 antisense RNA [Source:HGNC Symbol;Acc:HGNC:48926]	CDR1; CDR1-AS	0,68
chromosome 16 open reading frame 54	C16orf54	0,68
hepsin	HPN	0,68
egl-9 family hypoxia-inducible factor 3	EGLN3	0,68
Purkinje cell protein 2	PCP2	0,68
ankyrin repeat domain 30A	ANKRD30A	0,68
transmembrane protease, serine 6	TMPRSS6	0,68
axin 1	AXIN1	0,68
leucine rich repeat containing 39	LRRC39	0,68
vasohibin 2	VASH2	0,67
Memczak2013 ANTISENSE, coding, INTERNAL, intronic best transcript NM_152756	RICTOR	0,67
SPO11 meiotic protein covalently bound to DSB	SPO11	0,67
transmembrane protease, serine 11D	TMPRSS11D	0,67
Transcript Identified by AceView, Entrez Gene ID(s) 23670	TMEM2	0,67
four and a half LIM domains 2	FHL2	0,67
cutaneous T-cell lymphoma-associated antigen 1	CTAGE1	0,67
Memczak2013 ANTISENSE, CDS, coding, INTERNAL, intronic best transcript NM_004475	FLOT2	0,67
polymerase I and transcript release factor	PTRF	0,67
docking protein 7	DOK7	0,67
FXYD domain containing ion transport regulator 2	FXYD2	0,67
sorbin and SH3 domain containing 3	SORBS3	0,67
ATP binding cassette subfamily C member 12	ABCC12	0,67
acid phosphatase 5, tartrate resistant	ACP5	0,67
calmodulin-like 5	CALML5	0,67
PDZ domain containing 7	PDZD7	0,67
mannosyl (alpha-1,3-)-glycoprotein beta-1,2-N-acetylglucosaminyltransferase	MGAT1	0,67

Memczak2013 ALT_ACCEPTOR, ALT_DONOR, coding, INTERNAL, intronic best transcript NM_021156	TMX4	0,67
family with sequence similarity 19 (chemokine (C-C motif)-like), member A4	FAM19A4	0,67
EH domain containing 3	EHD3	0,67
establishment of sister chromatid cohesion N-acetyltransferase 2	ESCO2	0,67
Transcript Identified by AceView, Entrez Gene ID(s) 10777	ARPP21	0,67
selenoprotein N, 1	SEPN1	0,67
Memczak2013 ALT_ACCEPTOR, ALT_DONOR, coding, INTERNAL, intronic best transcript NM_003959	HIP1R	0,66
LIM domain only 2 (rhombotin-like 1)	LMO2	0,66
Memczak2013 ALT_ACCEPTOR, ALT_DONOR, coding, INTERNAL, intronic best transcript NM_024766	CAMKMT	0,66
chemokine (C-C motif) receptor 6	CCR6	0,66
asparaginyl-tRNA synthetase 2, mitochondrial (putative)	NARS2	0,66
PDZ and LIM domain 2 (mystique)	PDLIM2	0,66
THAP domain containing, apoptosis associated protein 2	THAP2	0,66
guanine nucleotide binding protein (G protein), beta polypeptide 4	GNB4	0,66
Transcript Identified by AceView, Entrez Gene ID(s) 6872	TAF1	0,66
ferredoxin-fold anticodon binding domain containing 1	FDXACB1	0,66
olfactory receptor, family 5, subfamily B, member 17	OR5B17	0,66
family with sequence similarity 53, member C	FAM53C	0,66
transmembrane protease, serine 2	TMPRSS2	0,66
outer dense fiber of sperm tails 1	ODF1	0,65
tubulointerstitial nephritis antigen-like 1	TINAGL1	0,65
solute carrier family 26 (anion exchanger), member 5	SLC26A5	0,65
solute carrier family 51, alpha subunit	SLC51A	0,65
olfactory receptor, family 2, subfamily C, member 1	OR2C1	0,65
6-phosphofructo-2-kinase/fructose-2,6-biphosphatase 3	PFKFB3	0,65
sema domain, immunoglobulin domain (Ig), short basic domain, secreted, (semaphorin) 3A	SEMA3A	0,65
BCL2 binding component 3; microRNA 3191	BBC3; MIR3191	0,65
Memczak2013 ANTISENSE, coding, INTERNAL, intronic best transcript NM_152996	ST6GALNAC3	0,65
Transcript Identified by AceView, Entrez Gene ID(s) 26249	KLHL3	0,65
MAS-related GPR, member E	MRGPRE	0,65
collagen, type III, alpha 1; microRNA 3606	COL3A1; MIR3606	0,65
collagen, type XXVIII, alpha 1	COL28A1	0,64
N-myc downstream regulated 1	NDRG1	0,64
UDP-Gal:betaGlcNAc beta 1,3-galactosyltransferase 1	B3GALT1	0,64
apolipoprotein L1	APOL1	0,64

tigger transposable element derived 4	TIGD4	0,64
potassium channel, voltage gated Shaw related subfamily C, member 1	KCNC1	0,64
tumor protein p53 inducible protein 13	TP53I13	0,64
Transcript Identified by AceView, Entrez Gene ID(s) 10060	ABCC9	0,64
uncharacterized LOC388282; novel transcript	LOC388282; CTD-2600O9.1	0,63
kinesin family member 25	KIF25	0,63
ribonuclease T2	RNASET2	0,63
triple QxxK/R motif containing	TRIQK	0,63
ribosomal protein S6 kinase, 90kDa, polypeptide 2	RPS6KA2	0,63
Transcript Identified by AceView, Entrez Gene ID(s) 10426	TUBGCP3	0,63
cyclic nucleotide binding domain containing 2	CNBD2	0,63
1-acylglycerol-3-phosphate O-acyltransferase 2	AGPAT2	0,63
Memczak2013 ANTISENSE, coding, INTERNAL, intronic best transcript NM_001033556	PPM1B	0,62
trefoil factor 1	TFF1	0,62
transmembrane protein 141	TMEM141	0,62
disrupted in renal carcinoma 3	DIRC3	0,62
Transcript Identified by AceView, Entrez Gene ID(s) 54556	ING3	0,62
Transcript Identified by AceView, Entrez Gene ID(s) 85444	LRRCC1	0,62
Janus kinase 3	JAK3	0,62
sex determining region Y	SRY	0,62
zinc finger and SCAN domain containing 5A	ZSCAN5A	0,61
protocadherin beta 5	PCDHB5	0,61
IGF like family member 2	IGFL2	0,61
Zhang2013 ALT_ACCEPTOR, ALT_DONOR, coding, INTERNAL, intronic best transcript NM_006877	GMPR	0,61
WD repeat domain 81	WDR81	0,61
transmembrane protein 91	TMEM91	0,61
single stranded DNA binding protein 4	SSBP4	0,61
solute carrier family 25 (mitochondrial carrier; phosphate carrier), member 23	SLC25A23	0,60
ankyrin repeat and SOCS box containing 6	ASB6	0,60
stimulated by retinoic acid 8	STRA8	0,60
UDP glucuronosyltransferase 2 family, polypeptide A2; UDP glucuronosyltransferase 2 family, polypeptide A1, complex locus	UGT2A2; UGT2A1	0,60
lipocalin 15	LCN15	0,60
boule homolog, RNA binding protein	BOLL	0,59
serine/threonine kinase-like domain containing 1 [Source:HGNC Symbol;Acc:HGNC:28669]	STKLD1	0,59
carbonic anhydrase IX	CA9	0,59
latexin	LXN	0,58
serine incorporator 2	SERINC2	0,57
RAB42, member RAS oncogene family	RAB42	0,57

arylsulfatase A	ARSA	0,57
connective tissue growth factor	CTGF	0,57
hydroxymethylbilane synthase	HMBS	0,56
Jeck2013 ANTISENSE, CDS, coding, INTERNAL, OVCODE, OVEXON, UTR3 best transcript NM_003542	HIST1H4C	0,56
endonuclease V	ENDOV	0,55
Transcript Identified by AceView, Entrez Gene ID(s) 153129	SLC38A9	0,55
NADH dehydrogenase (ubiquinone) Fe-S protein 7, 20kDa (NADH-coenzyme Q reductase)	NDUFS7	0,55
solute carrier family 2 (facilitated glucose transporter), member 14	SLC2A14	0,54
ceruloplasmin (ferroxidase)	CP	0,53
sperm associated antigen 4	SPAG4	0,52
glutathione peroxidase 3	GPX3	0,51
WD repeat domain 54	WDR54	0,51
solute carrier family 2 (facilitated glucose transporter), member 3	SLC2A3	0,50
glutathione peroxidase 1	GPX1	0,50
histone cluster 1, H4a	HIST1H4A	0,50
6-phosphofructo-2-kinase/fructose-2,6-biphosphatase 4; microRNA 6823	PFKFB4; MIR6823	0,50
insulin like growth factor binding protein 1	IGFBP1	0,47
solute carrier family 6 (neurotransmitter transporter), member 8	SLC6A8	0,46
serpin peptidase inhibitor, clade E (nexin, plasminogen activator inhibitor type 1), member 1	SERPINE1	0,46
hepatitis A virus cellular receptor 1	HAVCR1	0,45
erythropoietin	EPO	0,45
glucose-6-phosphatase, catalytic subunit	G6PC	0,42
FXYD domain containing ion transport regulator 1	FXYD1	0,35
lysyl oxidase	LOX	0,35
tubulin, beta 1 class VI	TUBB1	0,34
microRNA 6809; tensin 1	MIR6809; TNS1	0,33
placenta specific 8	PLAC8	0,32
carbohydrate (N-acetylgalactosamine 4-sulfate 6-O) sulfotransferase 15	CHST15	0,30
